# Supplementary material for: RUFY3 and RUFY4 are ARL8 effectors that promote coupling of endolysosomes to dynein-dynactin
Source: Nat Commun. 2022 Mar 21;13:1506. doi: 10.1038/s41467-022-28952-y (PMC8938451; doi:10.1038/s41467-022-28952-y)
Supplement: Supplementary file 1 — Supplementary Information [file 41467_2022_28952_MOESM1_ESM.pdf]

Supplementary Information

RUFY3 and RUFY4 are ARL8 effectors that promote coupling of endolysosomes to dynein-dynactin

Tal Keren-Kaplan[1], Amra Sarić[1], Saikat Ghosh[1], Chad D. Williamson[1], Rui Jia[1], Yan Li[2] and Juan S. Bonifacino[1*]

[1] Neurosciences and Cellular and Structural Biology Division, *Eunice Kennedy Shriver* National Institute of Child Health and Human Development, National Institutes of Health, Bethesda, Maryland, USA.

[2] Proteomics Core Facility, National Institute of Neurological Disorders and Stroke, National Institutes of Health, Bethesda, Maryland, USA.

*Corresponding author: juan.bonifacino@nih.gov

Short title: RUFY3 and RUFY4 are ARL8 effectors

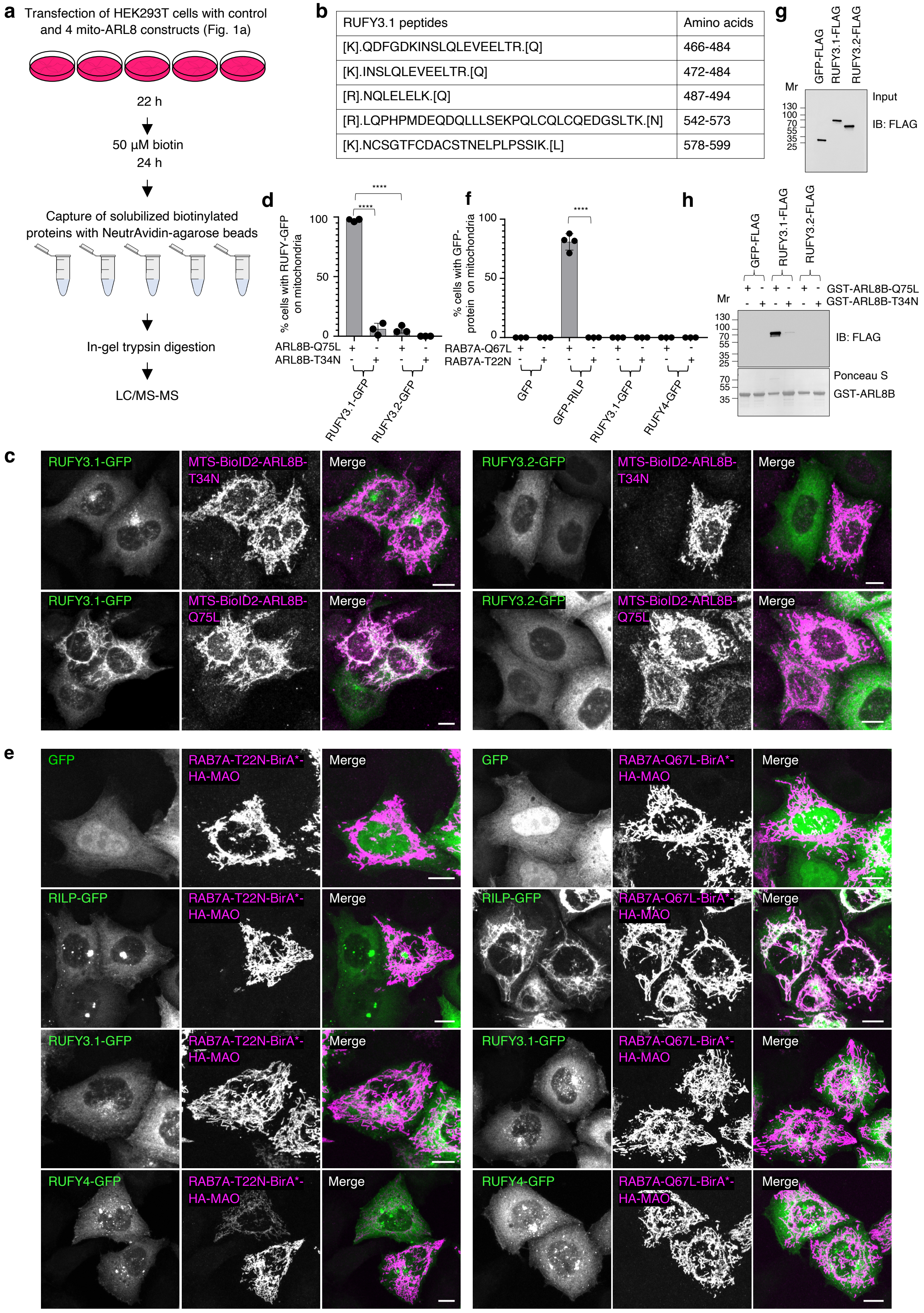

RUFY3.1 peptides	Amino acids
[K].QDFGDKINSLQLEVEELTR.[Q]	466-484
[K].INSLQLEVEELTR.[Q]	472-484
[R].NQLELELK.[Q]	487-494
[R].LQPHPMDEQDQLLLSEKPQLCQLCQEDGSLTK.[N]	542-573
[K].NCSGTFCDACSTNELPLPSSIK.[L]	578-599

Supplementary Fig. 1: MitoID procedure and identification of RUFY3.1 as a specific ARL8 effector.

a Workflow of the MitoID procedure. **b** Peptides and amino-acid numbers specific to the RUFY3.1 spliceform identified by mass spectrometry. **c** Immunofluorescence microscopy of HeLa cells co-expressing RUFY3.1-GFP or RUFY3.2-GFP fusion proteins (green) with MTS-BioID2-ALR8B-T34N or MTS-BioID2-ALR8B-Q75L. Fixed cells were stained with antibody to BioID2 (magenta), and imaged by confocal microscopy. Single channels are shown in grayscale. Scale bars: 10 μm. Images are representative of 3 independent experiments with similar results. **d** Quantification of the percentage of cells in which RUFY proteins were re-localized to mitochondria in experiments such as that in panel **c**. Values are the mean ± SD from 3 independent experiments scoring a minimum of 300 cells per condition. Statistical significance was calculated using one-way ANOVA with multiple comparisons between the groups using Tukey's test, **** p<0.0001. **e** Immunofluorescence microscopy of HeLa cells co-expressing GFP, GFP-RILP, RUFY3-GFP or RUFY4-GFP (green) with RAB7A-T22N-BirA*-HA-MAO or RAB7A-Q67L-BirA*-HA-MAO[1]. Fixed cells were stained with antibody to the HA epitope tag (magenta), and imaged by confocal microscopy. Single channels are shown in grayscale. Scale bars: 10 μm. Images are representative of 3 independent experiments with similar results. **f** Quantification of the percentage of cells in which RUFY proteins were re-localized to mitochondria in experiments such as that in panel **e**. Values are the mean ± SD from 3 independent experiments scoring a minimum of 300 cells per condition. Statistical significance was calculated by one-way ANOVA with multiple comparisons between the groups using Tukey's test, **** p<0.0001. **g, h** Recombinant GST-ARL8B-Q75L and GST-ARL8B-T34N were purified using the GST tag and used to pull down the indicated FLAG-tagged proteins expressed by transfection in HEK293T cells. FLAG-tagged proteins were identified by immunoblotting (IB) and GST proteins by Ponceau S staining. The positions of molecular mass markers (in kDa) in panels **g** and **h** are indicated at left.

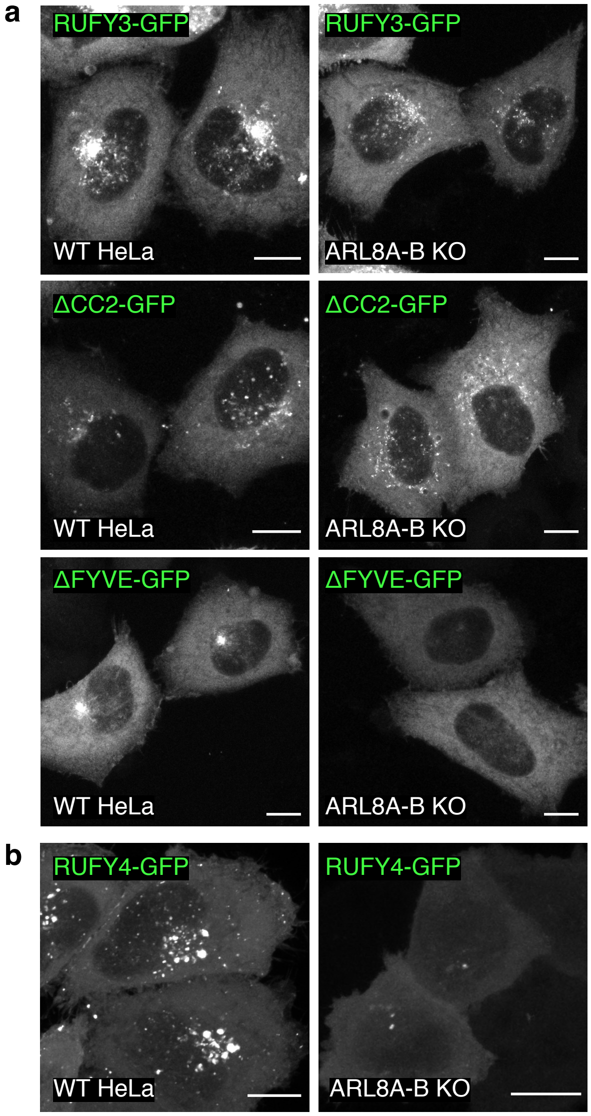
a
RUFY3-GFP
WT HeLa
RUFY3-GFP
ARL8A-B KO
ΔCC2-GFP
WT HeLa
ΔCC2-GFP
ARL8A-B KO
ΔFYVE-GFP
WT HeLa
ΔFYVE-GFP
ARL8A-B KO
b
RUFY4-GFP
WT HeLa
RUFY4-GFP
ARL8A-B KO

Supplementary Fig. 2: Distribution of full-length RUFY3-GFP and RUFY4-GFP, and domain-deleted RUFY3-GFP constructs, in WT and ARL8A-B-KO cells.

a Live-cell imaging of WT and ARL8A-B-KO HeLa cells transfected with plasmids encoding the indicated RUFY3-GFP full-length and deletion constructs (for schemes of the constructs, see Fig. 2c). **b** Live-cell imaging of WT and ARL8A-B-KO HeLa cells transfected with a plasmid encoding RUFY4-GFP. Images are shown in grayscale. Scale bars: 10 μm. Images are representative of 2 independent experiments with similar results.

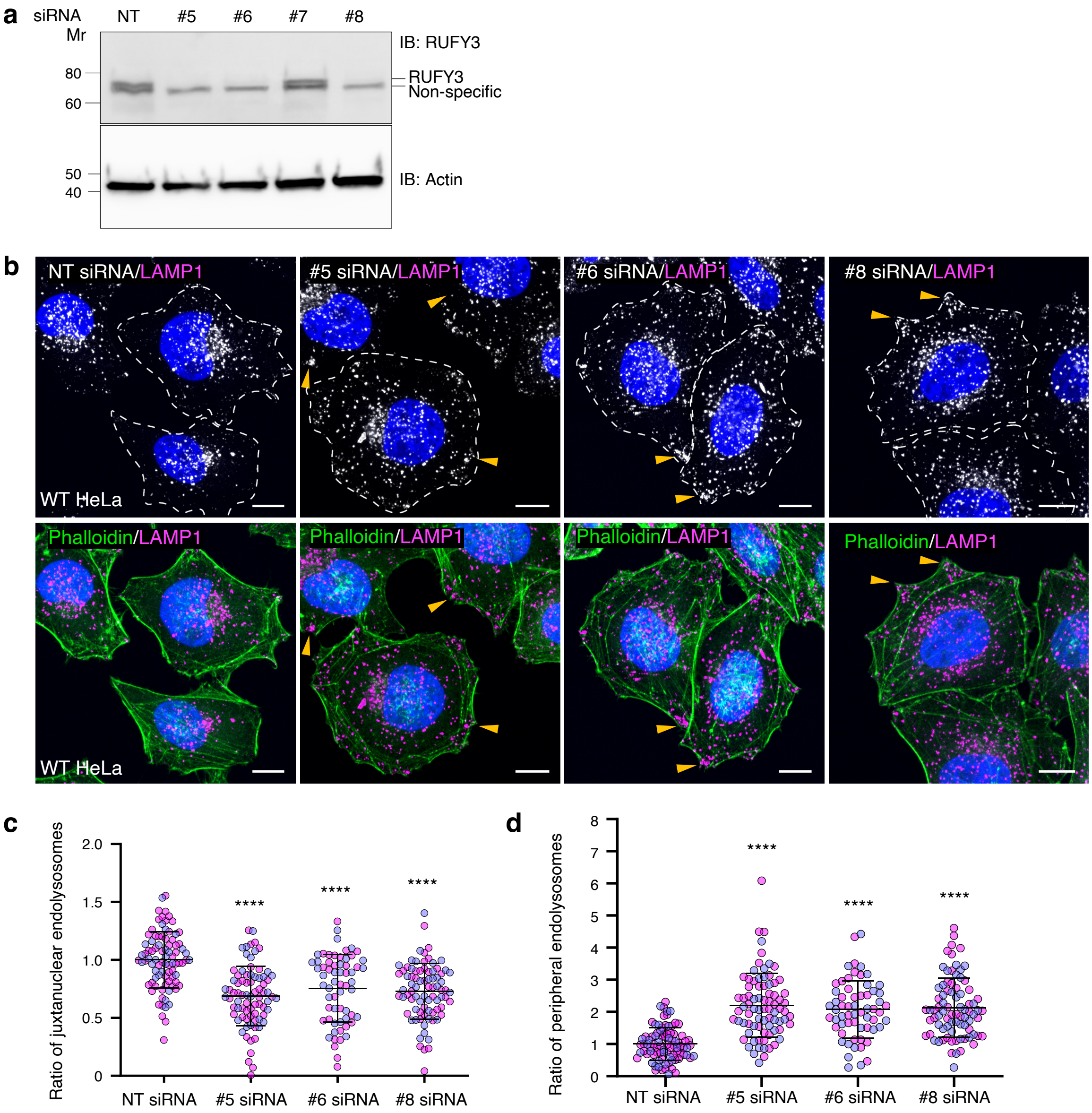

a
siRNA
NT
#5
#6
#7
#8
Mr
IB: RUFY3
80
60
RUFY3
Non-specific
50
40
IB: Actin
b
NT siRNA/LAMP1
#5 siRNA/LAMP1
#6 siRNA/LAMP1
#8 siRNA/LAMP1
WT HeLa
Phalloidin/LAMP1
Phalloidin/LAMP1
Phalloidin/LAMP1
Phalloidin/LAMP1
WT HeLa
c
Ratio of juxtanuclear endolysosomes
2.0
1.5
1.0
0.5
0
****
****
****
NT siRNA
#5 siRNA
#6 siRNA
#8 siRNA
d
Ratio of peripheral endolysosomes
8
7
6
5
4
3
2
1
0
****
****
****
NT siRNA
#5 siRNA
#6 siRNA
#8 siRNA

Supplementary Fig. 3: Endolysosome dispersal caused by RUFY3 KD using individual siRNAs.

a HeLa cells were treated with non-targeting (NT) or individual siRNAs (#5-8) for RUFY3 from the SMARTpool, and extracts were immunoblotted (IB) for endogenous RUFY3. Actin was used as a loading control. RUFY3 is the upper band and a non-specific protein is the lower band in the doublet. The positions of molecular mass markers (in kDa) are indicated at left. Notice that 3 of the siRNAs (#5, #6 and #8) were effective at knocking down RUFY3; these were used in subsequent immunofluorescence microscopy experiments. **b** Immunofluorescence microscopy of HeLa cells treated with non-targeting (NT) or individual RUFY3 siRNAs (#5, #6 and #8), and stained with antibodies to endogenous LAMP1 (grayscale and magenta) and Alexa fluor 633-conjugated phalloidin (green). Nuclei were stained with DAPI (blue). Cell edges are highlighted with dashed lines in grayscale images and with Alexa fluor 633-conjugated phalloidin (green) in colored images. Arrowheads indicate accumulation of endolysosomes at cell vertices. Scale bars: 10 μm. Images are representative from two independent experiments with similar results. **c, d** Quantification of the ratio of juxtanuclear (c) or peripheral (d) LAMP1 to total LAMP1 calculated by shell analysis from the experiment shown in panel b. Colors represent results from two independent experiments. Values are represented as the mean ± SD of all individual points. Statistical significance was calculated using one-way ANOVA with multiple comparisons to the NT siRNA control using Dunnett's test. **** $p<0.0001$.

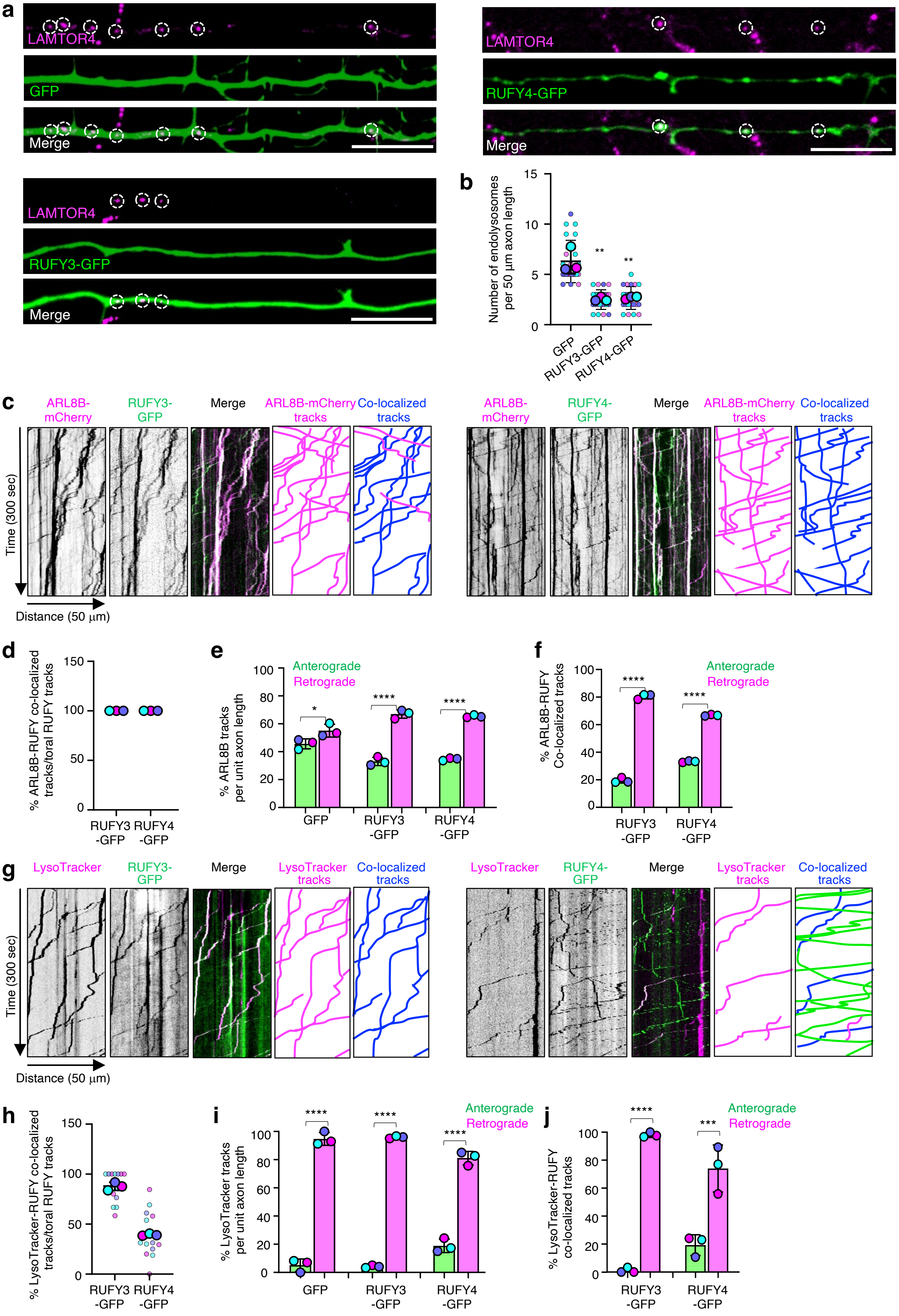

a
LAMTOR4
GFP
Merge
LAMTOR4
RUFY3-GFP
Merge
LAMTOR4
RUFY4-GFP
Merge
b
Number of endolysosomes per 50 µm axon length
15
10
5
0
**
**
GFP
RUFY3-GFP
RUFY4-GFP
c
ARL8B-mCherry
RUFY3-GFP
Merge
ARL8B-mCherry tracks
Co-localized tracks
ARL8B-mCherry
RUFY4-GFP
Merge
ARL8B-mCherry tracks
Co-localized tracks
Time (300 sec)
Distance (50 µm)
d
% ARL8B-RUFY co-localized tracks/toral RUFY tracks
150
100
50
0
RUFY3 -GFP
RUFY4 -GFP
e
% ARL8B tracks per unit axon length
Anterograde
Retrograde
100
80
60
40
20
0
*
****
****
GFP
RUFY3 -GFP
RUFY4 -GFP
f
% ARL8B-RUFY Co-localized tracks
Anterograde
Retrograde
****
****
RUFY3 -GFP
RUFY4 -GFP
g
LysoTracker
RUFY3-GFP
Merge
LysoTracker tracks
Co-localized tracks
LysoTracker
RUFY4-GFP
Merge
LysoTracker tracks
Co-localized tracks
Time (300 sec)
Distance (50 µm)
h
% LysoTracker-RUFY co-localized tracks/toral RUFY tracks
150
100
50
0
RUFY3 -GFP
RUFY4 -GFP
i
% LysoTracker tracks per unit axon length
Anterograde
Retrograde
****
****
****
GFP
RUFY3 -GFP
RUFY4 -GFP
j
% LysoTracker-RUFY co-localized tracks
Anterograde
Retrograde
****
***
RUFY3 -GFP
RUFY4 -GFP

Supplementary Fig. 4: Co-movement of RUFY3 and RUFY4 with ARL8B, and relationship to acidic organelles, in the axon of rat hippocampal neurons.

a Immunofluorescence microscopy of the axon of rat hippocampal neurons transfected with plasmids encoding GFP, RUFY3-GFP or RUFY4-GFP (green). Neurons were fixed and stained with antibody to endogenous LAMTOR4 (magenta). Axonal endolysosomes labeled for LAMTOR4 are highlighted with white circles. Images are representative from 3 independent experiments with similar results. **b** Quantification of LAMTOR4-positive endolysosomes within a 50 μm axon length from experiments such as that in panel **a**. Data are represented as SuperPlots, in which big circles represent the mean, and small dots the individual data points, from each experiment. Horizonal lines indicate the mean ± SD of the means from 3 independent experiments. Experiments are color coded. Statistical significance was calculated using one-way ANOVA with multiple comparisons to the GFP control using Dunnett's test. ** $p<0.01$. **c** Live-cell imaging of the axon of rat hippocampal neurons that were transfected with plasmids encoding ARL8B-mCherry (magenta) along with GFP, RUFY3-GFP or RUFY4-GFP (green). Axons were imaged using a spinning-disk confocal microscopy, and trajectories of fluorescent particles were represented as kymographs. Single channels are represented in grayscale. ARL8B-mCherry-only tracks and co-localized tracks with RUFY3-GFP or RUFY4-GFP are highlighted in separate kymographs. **d** Quantification of the percentage of co-localized ARL8B-mCherry–RUFY-GFP tracks relative to total RUFY-GFP tracks from experiments such as that in panel c. Values are the mean ± SD from 3 independent experiments. **e** Quantification of the percentage of anterograde and retrograde ARL8B-mCherry tracks in the presence of GFP, RUFY3-GFP or RUFY4-GFP from experiments such as that in panel c. Values are the mean ± SD of ARL8B-mCherry tracks from 3 independent experiments. Statistical significance was calculated using one-way ANOVA with multiple comparisons using Tukey's test. *$p<0.05$, ****$p<0.0001$. **f** Quantification of the percentage of anterograde or retrograde ARL8B-mCherry–RUFY-GFP co-localized tracks from experiments such as that in panel c. Values are the mean ± SD of co-localized tracks from 3 independent experiments. Statistical significance was calculated using one-way ANOVA with multiple comparisons using Tukey's test. ****$p<0.0001$. **g** Same as panel c, except that endolysosomes were labeled with LysoTracker red (magenta) instead of ARL8B-mCherry. **h** Quantification of the percentage of co-localized LysoTracker–RUFY-GFP tracks relative to total RUFY-GFP tracks from experiments such as that in panel g. Values are the mean ± SD from 3 independent experiments. **i** Quantification of the percentage of anterograde and retrograde LysoTracker tracks in the presence of GFP, RUFY3-GFP or RUFY4-GFP from experiments such as that in panel g. Values are the mean ± SD of ARL8B-mCherry tracks from 3 independent experiments. Statistical significance was calculated using one-way

ANOVA with multiple comparisons using Tukey's test. ****p<0.0001. **j** Quantification of the percentage of anterograde and retrograde LysoTracker-RUFY-GFP co-localized tracks from experiments such as that in panel g. Values are the mean ± SD of co-localized tracks from 3 independent experiments. Statistical significance was calculated using one-way ANOVA with multiple comparisons using Tukey's test. *** p<0.001, ****p<0.0001.

Supplementary Table 1: Abbreviations used in this study.

ARF	ADP-Ribosylation Factor
ARL8	ARF-Like GTPase 8
BICD2	Bicaudal D cargo adaptor 2
BORC	BLOC One Related Complex
CC1	Coiled Coil 1
CC2	Coiled Coil 2
CRISPR	Clustered Regularly Interspaced Short Palindromic Repeats
CT	C-terminal
DIC	Dynein Intermediate Chain
DHC	Dynein Heavy Chain
FOS	FLAG-One-Strep
HOPS	Homotypic Protein Sorting Complex
IB	Immunoblotting
GEF	Guanine Nucleotide Exchange Factor
GFP	Green Fluorescent Protein
kDa	Kilodalton
KO	Knock Out
LAMP1	Lysosome-Associated Membrane Protein 1
MTS	Mitochondrial Targeting Sequence
Mr	Relative molecular mass
ns	Not significant
NT	Non-Targeting
RUFY	RUN- and FYVE-domain-containing
RUN	RPIP8, UNC-14, and NESCA
RILP	RAB-Interacting Lysosomal Protein
SKIP	Sif-A and Kinesin-Interacting Protein
SNX	Sorting Nexin
TCEP	Tris (2-carboxyethyl)phosphine

Supplementary Table 2: Oligonucleotides used in this study.

Name	Sequence 5'->3'	Purpose
TKK1F	GACCCAAGCTGGCTAGCCACCATGGTGGGTC GGAACAGCGCCATCGCCGCCGGTGTATGCGG GGCCCTTTTCATTGGGTACTGCATCTACTTCG ACCGCAAAAGACGAAGTGGCGCGGGTGCAG AACAAAAACTCATCTCAGAAGAG	To make Mito-BioID2 (QuikChange mutagenesis) TOM20 seq
TKK2R	CTCTTCTGAGATGAGTTTTTGTTCTGCACCCG CGCCACTTCGTCTTTTGCGGTCGAAGTAGATG CAGTACCCAATGAAAAGGGCCCCGCATACA CCGGCGGCGATGGCGCTGTTCCGACCCACCA TGGTGGCTAGCCAGCTTGGGTC	To make Mito-BioID2 (QuikChange mutagenesis)
TKK3F	GCCGTACCGCTCGAGGGCGCGGGTGCAAAG GAAGAGATGGAGCTGACGCTC	To make Mito-BioID2-ARL8B with XhoI and BamHI sites
TKK4R	GCGCGGGATCCTCAGCTTCTCCGTGACTTCGA GTG	To make Mito-BioID2-ARL8B with XhoI and BamHI sites
TKK5F	GCCGTACCGCTCGAGGGCGCGGGTGCAAAG GAGGAGATGGAGCTCACGCTG	To make Mito-BioID2-ARL8A with XhoI and BamHI sites
TKK6R	GCGCGGGATCCTCAGCTTCTTCTAGATTTTGA ATGC	To make Mito-BioID2-ARL8A with XhoI and BamHI sites
TKK7F	CAGGGGGGTGGAGGTGGTACCATGTCTGCTC TGACGCCTCCGACC	To make His6-StrepII-sfGFP-RUFY3 (Gibson assembly)
TKK8R	GGTGGTGCTCGAGTGCGGCCGCTTATGATGG GCTGGTAGAATATTGC	To make His6-StrepII-sfGFP-RUFY3 (Gibson assembly)
TKK9F	GATCTCGAGCTCAAGCTTCGATGGCCGACCG GGAAGGC	To make RUFY1-GFP (Gibson assembly)
TKK10R	GGTACCGTCGACTGCAGAATTTTGGAGGCCG TGGAGGAGCAG	To make RUFY1-GFP (Gibson assembly)
TKK11F	GATCTCGAGCTCAAGCTTCGATGACTTTTCAG GTTTGGGGGTG	To make RUFY2-GFP (Gibson assembly)
TKK12R	GGTACCGTCGACTGCAGAATTTTGGGCAAGT TAGATGAGCATC	To make RUFY2-GFP (Gibson assembly)
TKK13F	GATCTCGAGCTCAAGCTTCGATGTCTGCTCTG ACGCCTCCGAC	To make RUFY3.1-GFP, RUFY3.1-mCherry and RUFY3.2-GFP (Gibson assembly)
TKK14R	GGTACCGTCGACTGCAGAATTTTTGATGGGCT GGTAGAATATTGC	To make RUFY3.1-GFP and RUFY3.1-mCherry (Gibson method)
TKK15R	GGTACCGTCGACTGCAGAATTTTTCCAAAGT CCTGTTTGAAG	To make RUFY3.2-GFP (Gibson assembly)

TKK16F	GATCTCGAGCTCAAGCTTCGATGGCAGAAGAGGGAGCCATC	To make RUFY4-GFP and mCherry (Gibson assembly)
TKK17R	GGTACCGTCGACTGCAGAATTTTGGTGACCTGGGCTTCTCTTC	To make RUFY4-GFP and mCherry (Gibson assembly)
TKK18R	TCTGTCTTGCTTGGGACCCTTCC	To make ΔCC2ΔFYVE (KLD mutagenesis)
TKK19R	ACCGTCTCCTTCAGTACCTTTACTGC	To make ΔCC1 (KLD mutagenesis)
TKK20F	ACTGCAGAAGGGCAAGCACTAAGTGAAGC	To make ΔCC1, CC2, ΔRUNΔCC1 (KLD mutagenesis)
TKK21R	TCCAGGGGCTTTTTAGTTTAAC	To make CC2 (KLD mutagenesis)
TKK22R	CATAGGGTGGGGTTGCAGC	To make ΔFYVE (KLD mutagenesis)
TKK23R	CATCGAAGCTTGAGCTCGAGATCTG	To make ΔRUN, ΔRUNΔCC1 (KLD mutagenesis)
TKK24F	AAAATTCTGCAGTCGACGGTACCGC	To make ΔFYVE, ΔCC2ΔFYVE-GFP, CC2-GFP, RUN-GFP (KLD mutagenesis)
TKK25F	GATTACAAGGATGACGACGATAAGTGA	To make ΔFYVE, ΔCC2ΔFYVE-FLAG, RUN-FLAG (KLD mutagenesis)
TKK26R	TTCTCCTTTCATACAGAAATTGGC	To make RUN-RUFY3 (KLD mutagenesis)
TKK27F	GATGAACAGGATCAGCTGCTG	To make FYVE-RUFY3 (KLD mutagenesis)
TKK28R	CATGGTGGCGGATCCGAGCTCGGTACC	To make ΔRUN, ΔFYVE-FLAG (KLD mutagenesis)
TKK29F	GACTTGGACTCTCAGGTTGG	To make ΔRUN-RUFY3 (KLD mutagenesis)
TKK30R	ATGGTGGCGAGCTCGAGCTCCTCCAAGGACCGTGCCCAGGAAGATGCATTTCTTTTTGTGGCGTTTCAGAAAATTCCATACAGACCTCAG	To make PEX3$_{1-42}$-FKBP-mRFP (KLD mutagenesis)
TKK31F	TATATATTCTGGGGAAATATGGACAGAAGAAAATCAGAGAAATACAGGAAGGTAGCGGCAGCGGTAGCATGGGAGTGCAGGTGGAAACC	To make PEX3$_{1-42}$-FKBP-mRFP (KLD mutagenesis)
TKK32F	CGCGGATCCCGAGCTCATGGAGGTGAAGCGGCTGTCCCAC	To make BICD2$_{25-400}$-FRB-EGFP (restriction cloning)
TKK33R	CATGGTCGACCGGAGGGCACTAAGGTTCTC	To make BICD2$_{25-400}$-FRB-EGFP (restriction cloning)

AS9F	CGGGGATCCATGGCGGCCGTGGGGCGAGTCG GCTCC	To make GST-LIC1 (restriction cloning)
AS10R	GCGGCTCGAGTCAAGAAGCTTCTCCTTCCGT AGGAG	To make GST-LIC1 (restriction cloning)

Supplementary Table 3: Plasmids used in this study

Name	Source	Catalog #
pmCherry-N1-ARL8B-mCherry, human	Ref. 2	N/A
pmCherry-N1-ARL8B-mCherry-Q75L, human	Ref. 3	N/A
pEGFP-N1-GFP	Clontech	N/A
pcDNA3.1-myc-BioID2-MCS	Ref. 4, gift from Kyle Roux	Addgene #74223
pcDNA3.1+/C-(K)-DYK-RUFY1-FLAG, human	GenScript	OHu19866D
pcDNA3.1+/C-(K)-DYK-RUFY2-FLAG, human	GenScript	OHu02933D
pcDNA3.1+/C-(K)-DYK-RUFY3.1-FLAG, human	GenScript	OHu24594D
pcDNA3.1+/C-(K)-DYK-RUFY3.2-FLAG, human	GenScript	OHu24610D
pcDNA3.1+/C-(K)-DYK-RUFY4-FLAG, human	GenScript	OHu55786D
pEGFP-C1-GFP-FLAG	Ref. 5, gift from Steve Jackson	Addgene #46956
LAMP1-RFP, Rat, C-RFP	Ref. 6, gift from Walter Mothes	Addgene #1817
pet28-His$_6$-StrepII-sfGFP-BICD2$_{25-400}$, mouse	Ref. 7, gift from Ronald Vale	N/A
pet28-His$_6$-StrepII-sfGFP-RUFY3.1, human	This work	N/A
pet28-His$_6$-StrepII-sfGFP	This work	N/A
pGST-parallel-1	Ref. 8	N/A
pGEX-6P-3-GST-LIC1, human	This work	N/A
pGEX-6P-3-GST-LIC1-CT$_{389-523}$, human	Ref. 9	N/A
pEGFP-p150^{Glued}-CC1, chicken	This work	N/A
pmCherry-N1-RUFY3-mCherry, human	This work	N/A
pmCherry-N1-RUFY4-mCherry, human	This work	N/A
pEGFP-N1-PEX3$_{1-42}$-FKBP-RFP	This work	N/A
pEGFP-N1-RUFY3-FRB-EGFP, human	This work	N/A
pEGFP-N1-RUFY4-FRB-EGFP, human	This work	N/A
pEGFP-N1-BICD2$_{25-400}$-FRB-EGFP, mouse	This work	N/A
pcDNA3.1-SKIP-FOS, human	This work	N/A
pMSCV-N-FLAG-HA-HOOK1, human	Ref. 10, gift from J. Wade Harper	N/A

pEGFP-N1-RUFY3-ΔRUN-GFP, human	This work	N/A
pEGFP-N1-RUFY3-ΔCC1-GFP, human	This work	N/A
pEGFP-N1-RUFY3-ΔCC2-GFP, human	This work	N/A
pEGFP-N1-RUFY3-ΔFYVE-GFP, human	This work	N/A
pEGFP-N1-RUFY3-ΔRUNΔCC1-GFP, human	This work	N/A
pEGFP-N1-RUFY3-ΔCC2ΔFYVE-GFP, human	This work	N/A
pEGFP-N1-RUFY3-CC2-GFP, human	This work	N/A
pEGFP-N1-RUFY3-FYVE-GFP, human	This work	N/A
pcDNA3.1+/C-(K)-DYK-RUFY3-ΔRUN-FLAG, human	This work	N/A
pcDNA3.1+/C-(K)-DYK-RUFY3-ΔCC1-FLAG, human	This work	N/A
pcDNA3.1+/C-(K)-DYK-RUFY3-ΔCC2-FLAG, human	This work	N/A
pcDNA3.1+/C-(K)-DYK-RUFY3-ΔFYVE-FLAG, human	This work	N/A
pcDNA3.1+/C-(K)-DYK-RUFY3-ΔRUNΔCC1-FLAG, human	This work	N/A
pcDNA3.1+/C-(K)-DYK-RUFY3-ΔCC2ΔFYVE-FLAG, human	This work	N/A
pcDNA3.1+/C-(K)-DYK-RUFY3-RUN-FLAG, human	This work	N/A
pcDNA3.1+/C-(K)-DYK-RUFY3-CC2-FLAG, human	This work	N/A
pcDNA3.1+/C-(K)-DYK-RUFY3-FYVE-FLAG, human	This work	N/A
pEGFP-N1-RUFY1-GFP, human	This work	N/A
pEGFP-N1-RUFY2-GFP, human	This work	N/A
pEGFP-N1-RUFY3.1-GFP, human	This work	N/A
pEGFP-N1-RUFY4-GFP, human	This work	N/A
pcDNA3.1-Mito-BioID2-ARL8A-Q75L, human	This work	N/A
pcDNA3.1-Mito-BioID2-ARL8B-Q75L, human	This work	N/A
pcDNA3.1-Mito-BioID2-ARL8A-T34N, human	This work	N/A
pcDNA3.1-Mito-BioID2-ARL8B-T34N, human	This work	N/A
pcDNA3.1-Mito-BioID2	This work	N/A
pcDNA3.1-RAB7A-T22N-BirA*-HA-MAO	Ref. 1, gift from Sean Munro	Addgene #128905
pcDNA3.1-RAB7A-Q67L-BirA*-HA-MAO	Ref. 1, gift from Sean Munro	Addgene #128904

pOPINE-GFPnanobody	Ref. 11, gift from Brett Collins	Addgene #49172
pEGFP-C1-GFP-RILP, human	Ref. 12, gift from Cecilia Bucci	N/A
pEGFP-C2-GFP-BICD2, mouse	Ref. 13, gift from Anna Akhmanova	N/A

N/A: not applicable

Supplementary References

1 Gillingham, A. K., Bertram, J., Begum, F. & Munro, S. In vivo identification of GTPase interactors by mitochondrial relocalization and proximity biotinylation. *Elife* **8**, e45916 (2019).

2 Farías, G. G., Guardia, C. M., De Pace, R., Britt, D. J. & Bonifacino, J. S. BORC/kinesin-1 ensemble drives polarized transport of lysosomes into the axon. *Proc. Natl. Acad. Sci. U. S. A.* **114**, E2955-E2964 (2017).

3 Keren-Kaplan, T. & Bonifacino, J. S. ARL8 Relieves SKIP Autoinhibition to Enable Coupling of Lysosomes to Kinesin-1. *Curr. Biol.* **31**, 540-554 e545 (2021).

4 Roux, K. J., Kim, D. I. & Burke, B. BioID: a screen for protein-protein interactions. *Curr. Protoc. Protein Sci.* **74**, 19.23.1-19.23.14 (2013).

5 Britton, S., Coates, J. & Jackson, S. P. A new method for high-resolution imaging of Ku foci to decipher mechanisms of DNA double-strand break repair. *J. Cell Biol.* **202**, 579-595 (2013).

6 Sherer, N. M. *et al.* Visualization of retroviral replication in living cells reveals budding into multivesicular bodies. *Traffic* **4**, 785-801 (2003).

7 Huynh, W. & Vale, R. D. Disease-associated mutations in human BICD2 hyperactivate motility of dynein-dynactin. *J. Cell Biol.* **216**, 3051-3060 (2017).

8 Sheffield, P., Garrard, S. & Derewenda, Z. Overcoming expression and purification problems of RhoGDI using a family of "parallel" expression vectors. *Protein Expr. Purif.* **15**, 34-39 (1999).

9 Saric, A. *et al.* SNX19 restricts endolysosome motility through contacts with the endoplasmic reticulum. *Nat. Commun.* **12**, 4552 (2021).

10 Xu, L. *et al.* An FTS/Hook/p107(FHIP) complex interacts with and promotes endosomal clustering by the homotypic vacuolar protein sorting complex. *Mol. Biol. Cell* **19**, 5059-5071 (2008).

11 Kubala, M. H., Kovtun, O., Alexandrov, K. & Collins, B. M. Structural and thermodynamic analysis of the GFP:GFP-nanobody complex. *Protein Sci.* **19**, 2389-2401 (2010).

12 Colucci, A. M., Campana, M. C., Bellopede, M. & Bucci, C. The Rab-interacting lysosomal protein, a Rab7 and Rab34 effector, is capable of self-interaction. *Biochem. Biophys. Res. Commun.* **334**, 128-133 (2005).

13 Splinter, D. *et al.* BICD2, dynactin, and LIS1 cooperate in regulating dynein recruitment to cellular structures. *Mol. Biol. Cell* **23**, 4226-4241 (2012).
