# Supplementary material for: RUFY3 and RUFY4 are ARL8 effectors that promote coupling of endolysosomes to dynein-dynactin
Source: Nat Commun. 2022 Mar 21;13:1506. doi: 10.1038/s41467-022-28952-y (PMC8938451; doi:10.1038/s41467-022-28952-y)
Supplement: Supplementary file 6 — Reporting Summary [file 41467_2022_28952_MOESM6_ESM.pdf]

nature portfolio

Corresponding author(s): Juan S. Bonifacino

Last updated by author(s): 12/27/2021

Reporting Summary

Nature Portfolio wishes to improve the reproducibility of the work that we publish. This form provides structure for consistency and transparency in reporting. For further information on Nature Portfolio policies, see our Editorial Policies and the Editorial Policy Checklist.

Statistics

For all statistical analyses, confirm that the following items are present in the figure legend, table legend, main text, or Methods section.

n/a	Confirmed	
☐	☒	The exact sample size (*n*) for each experimental group/condition, given as a discrete number and unit of measurement
☐	☒	A statement on whether measurements were taken from distinct samples or whether the same sample was measured repeatedly
☐	☒	The statistical test(s) used AND whether they are one- or two-sided<br>*Only common tests should be described solely by name; describe more complex techniques in the Methods section.*
☒	☐	A description of all covariates tested
☒	☐	A description of any assumptions or corrections, such as tests of normality and adjustment for multiple comparisons
☐	☒	A full description of the statistical parameters including central tendency (e.g. means) or other basic estimates (e.g. regression coefficient) AND variation (e.g. standard deviation) or associated estimates of uncertainty (e.g. confidence intervals)
☐	☒	For null hypothesis testing, the test statistic (e.g. *F*, *t*, *r*) with confidence intervals, effect sizes, degrees of freedom and *P* value noted<br>*Give P values as exact values whenever suitable.*
☒	☐	For Bayesian analysis, information on the choice of priors and Markov chain Monte Carlo settings
☒	☐	For hierarchical and complex designs, identification of the appropriate level for tests and full reporting of outcomes
☐	☒	Estimates of effect sizes (e.g. Cohen's *d*, Pearson's *r*), indicating how they were calculated

Our web collection on statistics for biologists contains articles on many of the points above.

Software and code

Policy information about availability of computer code

Data collection	Microscopy images were acquired using Zeiss ZEN Black software: Zen 2012 SP5 FP3 release version 14.0.22.201. And Zen 2.3 SP1 FP3 release version 14.0.25.201. Images were also acquired with NIS-Elements AR microscope imaging software using a high-speed EMCCD camera (iXon Life 897, Andor).<br>MS/MS data were acquired on an Orbitrap Lumos mass spectrometer (Thermo Fisher Scientific). Database search and label-free quantification were performed using Proteome Discoverer 2.4 software.<br>qPCR was performed on the AriaMx Real-Time PCR system using AriaMx software version 1.3 (Agilent Technologies)
Data analysis	Microscopy images were analyzed by the open-source software Fiji vl.52p (NIH, Bethesda, MD). Statistical tests were done using the commercially available software Prism v9 GraphPad Software (San Diego, CA, USA).

For manuscripts utilizing custom algorithms or software that are central to the research but not yet described in published literature, software must be made available to editors and reviewers. We strongly encourage code deposition in a community repository (e.g. GitHub). See the Nature Portfolio guidelines for submitting code & software for further information.

Data

Policy information about availability of data

All manuscripts must include a data availability statement. This statement should provide the following information, where applicable:

- Accession codes, unique identifiers, or web links for publicly available datasets
- A description of any restrictions on data availability
- For clinical datasets or third party data, please ensure that the statement adheres to our policy

Data availability
The mass spectrometry raw data generated in this study have been deposited in the MassIVE database and can be accessed at ftp://massive.ucds.edu/MSV000087741. The processed mass spectrometry data are available as Supplementary Data 1. Microscopy data that support our findings are available on reasonable request from the corresponding author. All other source data are provided with the paper in Source Data file.

Field-specific reporting

Please select the one below that is the best fit for your research. If you are not sure, read the appropriate sections before making your selection.

☒ Life sciences ☐ Behavioural & social sciences ☐ Ecological, evolutionary & environmental sciences

For a reference copy of the document with all sections, see nature.com/documents/nr-reporting-summary-flat.pdf

Life sciences study design

All studies must disclose on these points even when the disclosure is negative.

Sample size	Sample size was not predetermined, but was based on what is considered accepted in the field for similar experiments and the capacity of the researcher and experimental settings. See: (https://doi.org/10.1091/mbc.E15-02-0076)<br>For experiments in neurons, 5-8 cells per condition per experiment in 3 independent experiments (total of 15-24 cells per condition) were analyzed.<br>For HeLa cells, ~100 cells were manually scored per condition per experiment in 2-3 independent experiments (total of 200-300 cells per condition). Because the cells were scored manually, we could use a larger sample size.<br>For other experiments, number of cells analyzed varied, but it was no less than 11 cells per condition per experiment in typically 3 independent experiments to allow a sufficient sample size for statistical analyses. The exact number of cells analyzed can be found in the Source Data file. The exact number of experiments is indicated in the figure legend for each experiments.
Data exclusions	Except when overexpression was intended, the localization of transgenic proteins was analyzed in transfected cells exhibiting low-to moderate expression levels ("pre selection criteria").<br>Quantification of endolysosome positioning using "shell" analysis was performed in cells with a relatively round shape and centered nucleus. Cells that did not meet these criteria were excluded from analysis.
Replication	Most experiments were performed in replicates as indicated in the manuscript.<br>siRNA KD of RUFY3 with individual siRNAs (Supplemental figure 3) was done twice and the SMARTpool combining all 4 individual siRNA was replicated multiple times with similar results (n=3 independent experiments in Fig. 9b, n=3 independent experiments in Fig. 5d).<br>The pulldown in Fig. 7C was done once, but the interaction between RUFY3 and dynein-dynactin was analyzed by additional methods with similar conclusions. This was because of availability of time in the lab and of staff during COVID-19 limitations.<br>All other experiments in the paper were replicated at least n=2 independent experiments and in most cases n=3 independent experiments and showed similar results. The exact number each replicate experiments is indicated for each experiment in the figure legends.
Randomization	Samples were not randomized. They were all collected and analyzed the same way.
Blinding	Blinding was not done, as cells were prepared and analyzed by the same investigator.

Reporting for specific materials, systems and methods

We require information from authors about some types of materials, experimental systems and methods used in many studies. Here, indicate whether each material, system or method listed is relevant to your study. If you are not sure if a list item applies to your research, read the appropriate section before selecting a response.

Materials & experimental systems

n/a	Involved in the study
☐	☒ Antibodies
☐	☒ Eukaryotic cell lines
☒	☐ Palaeontology and archaeology
☐	☒ Animals and other organisms
☒	☐ Human research participants
☒	☐ Clinical data
☒	☐ Dual use research of concern

Methods

n/a	Involved in the study
☒	☐ ChIP-seq
☒	☐ Flow cytometry
☒	☐ MRI-based neuroimaging

Antibodies

Antibodies used

Primary antibodies (catalog numbers, names, animal species, working dilutions and sources in parentheses): FLAG-HRP (Cat# A8592, RRID:AB_439702, mouse, 1:5,000-1:6,000, Millipore-Sigma), ARL8A (Cat# 17060-1-AP, RRID:AB_2058998, rabbit, 1:500, Proteintech), ARL8B (Cat# C13049-1-AP, RRID:AB_2059000, rabbit, 1:500, Proteintech), TOM20 (Cat# 11802-1-AP, RRID:AB_2207530, rabbit, 1:500, Proteintech), BioID2 (Cat# BID2-CP-100, chicken, 1:2000, BioFront Technologies), p150Glued (Cat# 610473, RRID:AB_397845, mouse, 1:300, BD Biosciences), DIC (Cat# MAB1618, RRID: AB_224605, mouse,1:200, Millipore-Sigma), Streptavidin-HRP (Cat# 21130, 1:10,000, Pierce), GFP-HRP (Cat# 130-091-833, RRID:AB_247003 mouse, 1:2,000, Miltenyi Biotec), LAMTOR4 (C7orf59) (D4P6O) (Cat# 13140, RRID:AB_2798129, rabbit, 1:200, Cell Signaling Technology), LAMP1 (DSHB Hybridoma Product H4A3, mouse, 1:500, deposited by J.T. August and J.E.K. Hildreth), FLAG (Cat# F1804, RRID:AB_262044, mouse, 1:200, Millipore-Sigma), RUFY3 (Cat# NBP1-89614, RRID:AB_11022810, rabbit, 1:500, Novus Biological). HA (Cat# 11867423001, RRID: AB_390918, rat, 1:300, Roche). Pan-Neurofascin extracellular (Cat# A12/18, RRID:AB_2877334, mouse, 1:100, UC Davis/NIH NeuroMab Facility)

Secondary antibodies: HRP-conjugated goat anti-rabbit IgG (H+L), (Cat# 111-035-003, RRID:AB_2313567, 1:10,000, Jackson ImmunoResearch), HRP-conjugated donkey anti-mouse IgG (H+L) (Cat# 715-035-150, RRID:AB_2340770, 1:10,000, Jackson ImmunoResearch), donkey-anti-mouse IgG Alexa Fluor 488 (Cat# A21202, RRID:AB_141607, 1:2,000, Thermo Fisher), donkey-anti-mouse IgG Alexa Fluor 555 (Cat# A31570, RRID:AB_2536180, 1:2000, Thermo Fisher), goat anti-Chicken IgY (H+L) Alexa Fluor 555 (Cat# A21437, RRID:AB_1500593, 1:1000, Thermo Fisher), donkey anti-mouse IgG Alexa Fluor 647 (Cat# A31571, RRID:AB_162542, 1:1,000, Thermo Fisher). We also used Alexa Fluor 546-phalloidin (Cat# A22283, 1:2000, Thermo Fisher) and Alexa Fluor 633-phalloidin (Cat# A22284, 1:400, Thermo Fisher).

Validation

Antibodies were chosen based on suppliers' recommendations and previous publications, and all antibodies detected proteins of expected molecular weights. The specificity of the RUFY3 antibody used for immunoblotting was validated by using RUFY3 KD cells as a negative control (Supplementary figure 3a).
ARL8A and ARL8B were validated with a ARL8A-B-KO cell line (Keren-Kaplan and Bonifacino, Current Biology, 2021)
TOM20 validated by supplier
BIOID2 validated by supplier
DIC validated by supplier
P150 glued (cited multiple times, single band in correct molecular weight)
HA rat validated by manufacturer.
LAMP1 validated by multiple publications available in manufacturer's site
LAMTOR4 (Pu et al., JCB, 2017)
GFP-HRP - validated in (Keren-Kaplan and Bonifacino, Current Biology, 2021)
FLAG-HRP- validated in multiple citations in supplier's website

Eukaryotic cell lines

Policy information about cell lines

Cell line source(s)	HeLa (CCL-2, ATCC) and HEK293T (Takara Bio #632180)
Authentication	Providers' authenticated cell lines. In addition, we assessed the cell morphology by microscopy.
Mycoplasma contamination	Cells were not specifically tested for mycoplasma. However, routine staining with DAPI revealed no obvious bacterial or mycoplasma contamination.
Commonly misidentified lines (See ICLAC register)	We did not use misidentified cell lines.

Animals and other organisms

Policy information about studies involving animals; ARRIVE guidelines recommended for reporting animal research

Laboratory animals	Rats: Sprague Dawley® outbred, ENVIGO Stock No: Sprague Dawley® SD®
Wild animals	We did not use wild animals in the study

Field-collected samples	We did not use field-collected samples in the study
Ethics oversight	All research conducted in this study has been approved by the Intramural Research Program of NICHD. The animal procedure of rats was conducted following the NIH Guide for the Care and Use of Laboratory Animals, under protocols #19-011 and approved by the Animal Care and Use Committee of NICHD.

Note that full information on the approval of the study protocol must also be provided in the manuscript.
