# Supplementary material for: RUFY3 and RUFY4 are ARL8 effectors that promote coupling of endolysosomes to dynein-dynactin
Source: Nat Commun. 2022 Mar 21;13:1506. doi: 10.1038/s41467-022-28952-y (PMC8938451; doi:10.1038/s41467-022-28952-y)
Supplement: Supplementary file 3 — Description of Additional Supplementary Files [file 41467_2022_28952_MOESM3_ESM.pdf]

Description of Additional Supplementary Files

File name: Supplementary Data 1

Description: Proteins identified in the MitoID mass spectrometry.

A-DN: Mito-BioID2-ARL8A-T34N, A-CA: Mito-BioID2-ARL8A-Q75L, B-DNN: Mito-BioID2-ARL8B-T34N. B-CAN: Mito-BioID2-ARL8B-Q75L, Ctrl: MitoID-BioID2 control.

File name: Supplementary Movie 1

Description: RUFY3 and RUFY4 co-move with LAMP1-labeled endolysosomes in the axon.

DIV5 rat hippocampal neurons co-expressing LAMP1-RFP with RUFY3-GFP or RUFY4-GFP were imaged using a spinning-disk confocal microscopy. Dual-color images of 50 µm adjacent to the axon initial segment (AIS) at a speed of 15 frames per second for 300 seconds without any delay. Notice the comovement of LAMP1-RFP with RUFY3-GFP or RUFY4-GFP.

±ⁿ£╘TD⌐9╙ª╖g═≥5□ô╝L-¬à□⌐2d#└═┘□╒q
╓_ü«└■±v z▌ □¿&Z╣B╙Nδ'└. ÿò╕□╫≡□+kε
²ñ║i╢│qY▌₧ó«□□┴$ò«~s≤·h(╡0▄δ□Jó╢jÿÜ"½╒□·A▄ïóB±ô□εA⌠<"S.τ2□1⌡£)Ωêªh□ùO□□─s
:ƒq¥àΦ⌠Γ╤╔$≈&Dç<δ=¿⌠⌡6─î±ó¼j≈è╞äTè█□Çò╨≥<@µ╕╝E¢>Eƒ%▄*"]k╢Q□└g3ε│ÉqP₧#┤w¿└

?□ÿà/A°□∩

~á¬□f□¡Xüb│É─#□W╧qe□tåät╧&¼£
s□□⌐╥(□▄åU₧q⌐≈□P
╓M╙d^ÿ▀┌Θ□.Bé$ï9□∞+±Φj┼≤□□è╓Äδ□╛ □⌐□ëGd□□4ßⁿÖ□G$ε□≈ç□Θ5╧C╞▓╥^ß

□u╦që ▌Bù°'P8Gt□c%?Ç╟□K╒P(Ω┐□╨d─□⌡k2ì╜└■íeÄ█PB{Ü╗xL□╣J≥□ÿ≡╢áT₧√╢╘Ñ¢o4¬╣πÖ
Q¡]□▌óèYΣI(iC$ú□yP╥□╙∞╤┘%8╗<□:pvπ□t÷π∞□£]¿□@□g╫YÇNK╞ΓlX¡τ8□⌐╜cîj▌Üm╧o╦áêÑ
ùτ2dY1K<H₧V g
FΦS╕┬╓□°□╒Ü₧╬_╪v╖╫□Ç▄□8z«δ6□╖n□E÷╙╛ □╟fπφ└µ+>/ïΦπ3╧3□Γ²9Θ┘x╥│ÖjQ▀ 4=kgµⁿπ
□U≤Iô□'K M├░s`δ#>□}2█1□M¥^ÿƒ┌□SJc□╫╬╪
▌╗¥Ä├

«¥╬Γ□$NvX4≤⌠;2J□□═□┘;ä?□c□8{íæ╢

╓ñ8□x-u□ⁿ□PN└□ lµèst╟▌@┤X╥ëB=c£╫├E°áI$.φ.□┼åü╜∞□»¼□ç▐
P'▀R█─J≤╥╤dk⌐Ω□╧│□▓êò¢±9□)Ω╣`Γ"□□'╕4φ╤Σ⌠W□¡Ñ≡║₧`,⌐□▙αi╨□$Å□Ö┬╥t▓
╥½·hhëìw±eMs

î>□╚m>Ωá□╩CcCⁿG¥I□î√□B┼7□ óªdTl6σ≥□¼□□▄╓≤ç1□?C┘DLFZ╨ ╪■╤k<c
è·|t╞□┘]ëe]u├ ≡□┬K□æâLp}□Ek¿Å}PªoG¿K╥πεxt
x□-f│xü┴X.⌠□≡□□ì\■/x!5í%▀╒i,O╓PRü;SσZƒZ]c4╛è□□╖Q d`≤'<a)□■□í□ │
àú═¬l*fⁿk₧╞kfî╞æ□Çíÿ]ôq8≤□□ó□°=_lZ'╟ªñ¢▌é~ª□n□╗ûiè!□□)! Ocå[□╒»
▒╖B▀╕»¢9J□_(
D$ƒ□«dd5ΦVB₧[
t$ñ□})α█ƒ⌠{□╛▌¿/⌡ú√l□d╡É÷ñ/□«█~╧└╖P░&·x)▀□Öï~~T∩─_zXV□▐π▓-╓>¼Φ7├~O╫╖TfΘ╖=
δÜì⌠V9á'.onEM┴;á2 fë

\√□>╖K°~i□}M|φx┬□
]?{□╠╗~├
`½_□□ær>¿ù≡GnD╝EÅ□□₧<¡ô6┼5uè╥è}Nh╛ □□ ▌□±9e□╨G□&□«7½□π ╘╣Üa«f□/q

┴fδç□□½N*d░éYS─█`∫□oΩ9r;tF6{H□u┬≤ë@r⌠┴6P¬□u7▀°Ω□□AM□Ç│ì¢[▄ëÄ□|B┴îul▌╦^□4
Ö▄só■₧□]<Ü°ånZ`U\▌□▒+*+
ùYH╓±GÑVB□╒╥:m ┴╫m▐Y□^╘±V£│=□W╥A(

▒□3Σ{°⌐
□:Qs╕-8ε╩²öW╩□*≡÷ÄV□:¿
□□òW5▐êσò□ûk}□╝ZdöIéx□σ╞£∩⌐

```
Å╤□$█║╩□□┬σ╙c
□endstream
□endobj
□5 0 obj
□<</Type/Font/Subtype/TrueType/Name/F1/BaseFont/ABCDEE+Calibri/Encoding/W
inAnsiEncoding/FontDescriptor 6 0 R/FirstChar 32/LastChar 121/Widths 33 0
R>>
□endobj
□6 0 obj
□<</Type/FontDescriptor/FontName/ABCDEE+Calibri/Flags 32/ItalicAngle
0/Ascent 750/Descent -250/CapHeight 750/AvgWidth 521/MaxWidth
1743/FontWeight 400/XHeight 250/StemV 52/FontBBox[ -503 -250 1240 750]
/FontFile2 31 0 R>>
□endobj
□7 0 obj
□<</Type/ExtGState/BM/Normal/ca 1>>
□endobj
□8 0 obj
□<</Type/ExtGState/BM/Normal/CA 1>>
□endobj
□9 0 obj
□<</Type/Font/Subtype/TrueType/Name/F2/BaseFont/ABCDEE+Calibri,Bold/Encod
ing/WinAnsiEncoding/FontDescriptor 10 0 R/FirstChar 32/LastChar
121/Widths 34 0 R>>
□endobj
□10 0 obj
□<</Type/FontDescriptor/FontName/ABCDEE+Calibri,Bold/Flags 32/ItalicAngle
0/Ascent 750/Descent -250/CapHeight 750/AvgWidth 536/MaxWidth
1781/FontWeight 700/XHeight 250/StemV 53/FontBBox[ -519 -250 1263 750]
/FontFile2 35 0 R>>
□endobj
□11 0 obj
□<</Type/Font/Subtype/Type0/BaseFont/ABCDEE+Calibri/Encoding/Identity-
H/DescendantFonts 12 0 R/ToUnicode 30 0 R>>
□endobj
□12 0 obj
□[ 13 0 R]
□endobj
□13 0 obj
□<</BaseFont/ABCDEE+Calibri/Subtype/CIDFontType2/Type/Font/CIDToGIDMap/Id
entity/DW 1000/CIDSystemInfo 14 0 R/FontDescriptor 15 0 R/W 32 0 R>>
□endobj
□14 0 obj
□<</Ordering(Identity) /Registry(Adobe) /Supplement 0>>
□endobj
□15 0 obj
□<</Type/FontDescriptor/FontName/ABCDEE+Calibri/Flags 32/ItalicAngle
0/Ascent 750/Descent -250/CapHeight 750/AvgWidth 521/MaxWidth
1743/FontWeight 400/XHeight 250/StemV 52/FontBBox[ -503 -250 1240 750]
/FontFile2 31 0 R>>
□endobj
□16 0 obj
□<</Author(Kristen Sharp) /Creator(■ □M□i□c□r□o□s□o□f□t□«□ □W□o□r□d□
□2□0□1□6) /CreationDate(D:20220202155358-05'00')
```

/ModDate(D:20220202155358-05'00') /Producer(■ □M□i□c□r□o□s□o□f□t□«□
□W□o□r□d□ □2□0□1□6) >>
□endobj
□23 0 obj
□<</Type/ObjStm/N 12/First 81/Filter/FlateDecode/Length 352>>
□stream
□x£¥S╥è┬0□▌
■├²â<┌┤□D□Fe□Qñ□f!.b╜Sïm#1□²√I┌

vßb:¢£▄╟97╜çr□□╕□é□□└(□-□□-≡□8⌡üOl^□Å└úü═Ç'B`!°~□l□┬°└"□C[π□F□L°dδ·(─$![▓
{\æ$F╫⌐Y□XÆ╒-Φ□╚6□╧⌡╠fπ╤(l8à□°x├)■pè□N ^Q¼
□Ej≤r□╬█╪╢█Çh!h!la╥BΣα□¥vOk°□cÑ

ëUükyuª╖⌐v&VM╒·∩2n mez╒
▐╞□└:ΘÑ╒¬öA▓q╟ó:=â¥m=¬;I05Σ□σ
u{w£▀√gUΣ□&gΘ^Φ□oòUÉ&WU□kô□K{ió/Ñ/GÑ.d«╥║┤oj2╖3ói╫║û⌐V╜°²l╧^<╧eí▓^"
)≥□÷z█9╢-╙▓$╤<½5v▀║⌐╥█▐²^ⁿ╣▌ ╕2-²□□⌐□▌
□endstream
□endobj
□30 0 obj
□<</Filter/FlateDecode/Length 342>>
□stream
□x£]Æ╧nâ0

╞∩<EÄ▌ íé└h¿äÉ:Z$

√ú▒=□ML□iä(╨□○┐`│nj$ê~▓?τs£░¼Å╡╤□
▀▄ □ÿX°ìr0

W'ü¥ßóM└cª┤£V┬┐∞[□ä^▄╟π□}m║!╚s□╛√α8╣Öm

j8├C□╛:□N¢
█|ûìτµjφ7⌠`&□□E┴□t╛╨sk_┌-Xê▓m¡|\O≤╓k■2>f
,FµdF

□F█Jp¡╣@ÉG~□,»ⁿ*□0Ω.╬W┘╣ô_¡├⌠─°GQ□□₧x□#φ8□Åê□ó□╤#╤ë(Eè3ó□R‖Ω□╤¬╦êÄD{$┐!□ê
*ó□¡»-oÄ□;Σ"]╥┐╪Q÷q═ªxv╫ □δü□-æòH"F┌ôQA
ε╔¿á□K*/╨v£ßÖ╔S╡Pró*   ^ZRqó⌠┐φσεù'r□¼╝:τgè∩□ç╣îQ□╕=5;╪E╡|?╥ú╢┬
□endstream
□endobj
□31 0 obj
□<</Filter/FlateDecode/Length 216331/Length1 607596>>
□stream
□x£∞}□`T┼┌÷;τ∞ªùM%dIv├ÆP6□Z □╔B□□□□Æ┼Mh
       )┤PCh□╞□h□╦╡w∞□╘═é□¼α┼«╪√U▒δ□,W⌠zQ╙ ╠yw
APε╜ □²ε≈∩¢<τyµ¥Ö≈╠╠ÖÖ3ïë!ADë╕ÿ¿2┐┤ht▀╧úk(┤{+Q╩╢éQ·ewσ┐▒å┤.¢êΓ?.□uj▐τ╗2«#
-a

Q░gt~Aßεg▀▄Kµ□½ëé>□]<▒⌠¼▓µ□2╫UÆ╛k≥ΦR≈¿oma┘ñ⌡□!╜≈ÆëÑY²»□|╨~"±□εZY=┐j╤α≈≤□□
═;@d÷U/[j▀▒Φ¡ló[v"²p▌óY≤╫╛»□&Z|'QñsVU├"J%□εƒâ·ûY⌡+δ>╗°ΩRó╗p -
?Ö][U≤╙°ò^─»G■á┘pD▐╙E▐∩Vñ╗═₧┐t┼φ₧╓ƒ□ih £-
≤jù,8x÷┴OH╝ü■Fε½_X]ò■┴°ùô╪TëΩôτW¡X╘'2#□⌡ƒF}√ⁿ┌ÑU╫£╡i□i²╬A·£□U≤ko8°├

□ƒ ~▀åE
□û┤║Yi=┌ù$╦/ZR╗(nV╫╬Dk_├φ┐"9╓AC?∞▓≤▓'gD□ ü:çÉ┤ç╛Z²╝Σ7ïûOⁿ·αíª╨}!âÉ

%ì╪P/ê

ô╪□╢Θτâ□7àε3"╡│╫[ñ╟┌ï«$
¡ □5-öE□êb□ß╛□ruôS\Lf□1_m╞êïTf²%Z»Q□i╤fM╙L║f·ä·┤φñn°□-
Çì/╡█╔E⌠│ë█□|âûa'╤&≤⌠φµ(┘Sè7E□mìx□╙Θ&<°□╥L□┤┼öOU╟═█G[■┘xF
∩í-
µ□Ü≥[e4╚┐□█ê?φ°uâ▐━}{²δq█¢⌐+╟1╧ñjô°cL⌠o⌠q█⌡□E╖O¢╖╥▌'}┐fΩzó<─M²╝··□╠╛□╥L&║
I□Äµ7»ûnΩ□┐⌐c·äφ(ª¢LgQ²»Γ¡8Z_∞√φX╘ÅQZ□ßÅ√

╫╤÷─┐nP□ε{╔±≤LwS▌╔┤]Ö■□╟1╡P¥╛ ÿqÿHE╟¡SN]:▄s#▌x╥≈;DiA#h≡»ⁿ╧╙
²∞▀□«·δ4⌡d∩╒í▐L¬8₧?x!U□╜□□╬7
ñ╨

⌡~ªi'□_[LΘJ¢6Sz╨╡ö-≥:Ñ¢å⌡ û□-;╣r*₧,□è{Σ²·-2╧┤ ¿/╕□Ñδ╗)√W▒ÄΘ½▀w╡╥Γ
Z≈{φA□ú╝)ò╬□□m{]Bª⌡[:─╢·xuâjΦΩ÷≈√U[rÄ ╠NX╛],φ┘Äq⌉4*9^□≤╝□²┌╜ö╓!µgöfj∞Φ;ε╜
Q╞□Gi┴π0┐▀▄²≥▓

┌{┘∩òSª_O]=¡┐~å·rΩ⌐▀xΓ²⌠xªmí|â'P╛÷)ì╤Zi┤╪E▌┤+⌐ù÷%╒ïj¬□≤█▐B║^L°z╙d¬╫>3PÇ≥╞
~'~D║/ì□ôC╓╤╓æM å2╡╡╘C[O╢□ª= ¡åyMb╧▌è £╓≈╬─
▌å÷f¬;±▐□░Ç² h┌╡"∞äyò┤┐}□ƒ▒z□■n⌠Éf╞g╗ Çi
xç□']~>¡□V²╨∩ósÇU┐WεdL╧ª≤ òz /╟t□═╙₧#ç╛Åf□%&7u╒▀Σ3èεúl╙ ¬6╨E╤□α^` 0
░□╡■╝j`Æí≤hû╢æ:δg╥T╜ü╧⌡-öí╧µ╧└z□eΘ≈╙8C▀H┼└Fá□ÿ

□f·≤ª□%╟m_Åôn_▀π╡□τ▓1Γ'£!╝4N╖çFj∩R║v;╧□φ□Ω»}╙φkoΓa¬□< N]φz╩□?P?m□
╫è¿╖6ûΓ╡BΩo╘═í«┌i|~>┘r□
X└■X3φ·τ■φπxª²@]┤╧ΘB=ê*⌠qtíß╖□Θ|■,*ngƒßƒB□Üµ#»æ6□Θ╖

]í]K°iMTê╜┴ñ╟C□L╔GΩ£▀±▀N□□░Ç□,`□
X└□□░Ç□,` {M~╞4°s■,⌐>g□·$>g−±π≤ª±Y╙ 9≤?╙·Ç□,`□
X└□□░Ç□,`□
X└□□░Ç□╧Dα'U□□░Ç□,`□
X└□□░Ç□,`□
X└□÷?╠┤E□

□╥ü

□Φß╧│□± ÷=Ωh□0□ÿΦGá─ƒ7□p¥┐v█¥ ε²□□░Ç□,`□
X└□□░Ç□,`□
X└□□░Ç□,`□
X└□□░Ç□,`□;┘╡=°G╖ `□√âM≈ú
%)▒□)(²#2ëùα(#□Öìƒ-ëñ«╘ô·P6σ╨P╨°╤TD□h2òS□-óò┤ëεí¡┤C⌠╙□δ□°dª⌠IΘ¢2
%;eh╧H{¿▌b_do┤»░»│ƒk┐0²·ƒMm╞▀ìBT;⌡áLΩKâ□u$□"Ω⌐TlD¡ª%FT▀qóµñî≡G]j_n_c▀`▀ê¿
ä¿óφ□┤□2≡0╛R░╬≈╢}f▄+ì *B[╡÷ä^¿Åá∞╢□çƒ·jC√»²▌?₧·±z·E⌠±║Å╫}p□æ ol□□ôⁿúVl\'í
≈╥ª╙èô□φJ?╧òú«Å╒»╘▌·□▌C&èíXJó□□╫□:
╜»@╘□ÜM
┬"ÆE¬Φ!èEàÿ&µêz▒P4èebì8O\ .□╫ê√┼N▒K<)₧2Θ&ô╪k2¢éL┴ª□S¿)L8E1ZΣêq□$~4ε²π▒□3

i═ □╞4 ·mπÜF╦Å:⌡╡ ·□□s_H▀°∩╫┐╓┐╤┐╒┐CΩ{²□«□ ƒG¬┐D□╘□╫,├╦╝°Ä²7rÄ·┐┼
┤≤╖╞□╣╟î╠∩⌠δÅ7²Σè╔°y≥AE]`□ /[┼Σ¬X┐ni├Æ┼ï□.ÿ_?oε£┘│Ωjkf╬ÿ>mΩöèrÅ╗¼tRI±─
πO□7╢h╠Φ┬éⁿ╝Q#]╣#N□>lΦÉ£┴â▓│·⌠╬∞ææ▐═╤╒û□cëÄ

2¢tMPfüú░╥ε=¿⌠Ü2□c╞⌠ûiG□□Uφ□ò^;\à□╦xφòF1{╟Æ.ö¼;ªñïK║Äö□□√p□▐;╙^α░{_╙w╪[EE
ë□zc╛ú▄ε▌oΦ±å6e□ëH$╥╥P├^É4;▀ε□ò÷□oß▓┘=□ò·ê╫□-ûτ╙½
δ¥I-aßÉßP▐-ÄE-
ó╟□a□¡G┴╨□ìB"σm╜zzAUì╖╕─SÉoMK+7|ög─≥□σyâìX÷9▓═t╛╜%sg≤□¡□ÜYΘî¿q╘TM⌡x⌡*Tj╓
Ü¢7xc£▐₧Ä|o╧Uƒ$í╦╡▐LG~ü╫Θ@░qôÄ▄@x═Θ□ç╜·□Bπ□√≈u⌠T·=AΘûHJ┘┼#├ä|Ñ mC
╤┐┤4┘û≤[]4□
oSëç╙vÜi⌡æ+╦Yε╒*e╬Nòôαû9M*τH⌡JGÜ|T□ò■∩e│ô╝M3φ╜31·╞w:╛æo≈Ω□ò3½gK«¬mv
Στ≤╕òy╝«|□Wò┐»□-}│P╛¬□¥ÿ#çí─π=r,≥╞;Fq□8∞≥□╠)⌡□Uⁿ╒╜±y^¬¼÷╫≥f□Σ╦v┘
Ü+≤╣ü2ûú─│â□┤φm□h╖n□@□⌐\╢├¢ÿççÆQ╨∞⌐⌐≤┌*¡5ÿƒuvÅ5=δ*╟≡ò;<╡σ≥)9,▐₧{q╗4πÄF-
⌠φÿ╥¬░∞ypzê▌úY⌡r·┤α░□ΓΓ□5□□□<.#)ƒΦ¿ßvÅ░Æ*å╗°KH╒!

□zz▐□ÖÑ╥¬yc¼iσil┐╤$½┐MµtoH╖X□8Ä┤ë∩s┬ªqi┘á₧÷é┌ⁿv
∞□╘∞oá?┌±█⌐╔░≡▀□5BΣπ□ú▓⌠t¼\°4ä1\≥)&┘╜Tl≈8j□σ

╠!W▒G÷MÄ╡±|╟ò:╞òTxî°φƒ%e□R£ƒ├)/Ñ![%┤<╠┴B°U=V#=┌HIÄ9&╗He;d╗Ü¢kZHOùS┘┌"

a╬;┐■;╤Yε≡╬t:╥d;{g╢äPDZYe-╓j!╢;Gaò□/│┬µ¬╓╢ªÖ═-
.W≤óé╩┘C▒.Ü□E5═ÄR╧p½╤°I₧5╓U≥▌▒4Nî+□àP□ìjqêsKZ\Γ■╥□╧

æ²▄2ÅO□Z^σ¿≥ûn╚≤∞░π□`x5ΘòNÖ░╥äî4   ë□ú╝uçï¿╔╚5□

#]▌*╚≡à(ƒáΩVì}□╛Qåq#□╬é┌¡&╬q⌐╥&°B╪╫─Ñ{°Kç ╟"s-$╝H╚╚dk!9└«0│+─□Ωè╨"5

⌐t·αy□eC□mì□æ┬┌éÿô

w½hj  uYw□æ&·K6íñ⌠5□±íσ▓X╗@╕w▄}┤□ε□╧╓□B|πè□úña□&═╞□┬√ñ└^#τ▀Ω≥┘═òσr≈áD╠U|
»pî »µ□ü□□Ex├□╡ú╝ßÄQ╥ƒ+²╣∞□Æ■`╠|æ(≡░σª█\Θ└Fî□π!½α╡ª╦É÷╓╢╢2O┌
╓²σiXKSü□Å7╘ëù¢9},╨ìû¿ä{┤╖⌐║J╢â▄-Y78╜¿║□δR□Dæ"o("ä·#áDíQG«7T¬╞\½r□□nl□Mσ▐
r°╝⌐gN╣▒^-^□π□Ω
╩αÿµ

yú¼≥µXG□c≤┴Z□K▀
)□múR□{¼HΓfσ<H┴□hy╡□Y╒òv₧#ÑX╤ⁿ▓□│▓°□{╛)ú╓@ÿ╒ƒI▓[zzxdÿ7┤□□Γ[Ω≡>r╧1°□ùùsπì╘
□□□▄█Γ
Gï2┌
Ñ┐□F□YE▓-°▐Ç ª╩ó╗dÿÆVÜΣXü¡S6┌ê□îlodzQ□▐n\?□-GÄ¬□"7┴p□î▌∞
û=Å└┐cKhm╗├▒2¡¥a∩Éo?9 ╚║□
ò╩¢Åux°8{gå□δì4▄══!æ╟»└π□□yä
°û^-▀□`9ßî·f/É»J╟╪□mé╙`ap≤X□▐ Z║□

::ûOÜ╜ª\ûBôïì╜∞äàD╗B≥5m□o╢

S)ßO±├l÷╬Ωÿ£}$Y(ü├`z-
>Cá+r»┼\Ök⌡╔cf¬"≥ë╪¢φ□╟Pç╝□òGKTΓ!□Y□ÿ■ÿur╤4U█=31┘□░▓╣░Y-Q½½ⁿ├µ┐ôwü│CH¼
ü╔â@▓;▌ªb{e╣╜□GSQΓIK│b5éφu8°:¬Σ½áÿ√S\a□U¬Üσ□'£T╩¡▌`╝ÿΩ¬j□ixâxσ

─ú/█h≥/□▓67;Ü╝╞║-
Daä╧└▓+Æä∩ENGU¡<B╫╔□t¡Q╖□═5FGF┃□8░ûkß6╞□□ç¡oª╝T7╦□·┤J'F"ª9╢┘>ñ□[≡4╝=L□╒ô+
±¬Æo$╗±¿½¼Ha□èd¬□ü╕`h║,╚K@╣f╛┃eZp·QÅ±╝╨╔àCî¿h┘$Å╖X□1╓ô□ï¥^¡S

2eτ┼ñ□Å┌°tÖ]äßuaVYem╖W+≤°ÅQ┐HV╡¬□╞╒α1▐!■⌡uΣmú▐CS¡□╙□·±r╨GûjOkOΓ├«M{╩╧∩QÄ÷

╣╡╖┴oé▀≥≤□α╫┴»ü_□┐□~□ⁿ□°Q≡#αç╔M&φ]□□ö□·□U□▄□╝□ÿi-"
□G}A±┌πö□╘□Kü╤□3╩>è╝[□QÉ];g[hÆ□ï□z╢□g)qª□MJ£í─Z%╓(▒Zë╙òXÑ─J%V(▒\ëeJ
4*▒Të□%□+▒HëàJ,Pb╛□⌡J╟Sb«□söÿ¡─,%Ωö¿UóFëj%f*QÑDÑ□3öÿ«─4%ª*1Eë□%╩ò≡(qÜ□ôòp
+QªD⌐□ôö(QóXëëJLPb╝□°*1Në▒J□)1Fë╤J□*QáD╛□yJîRbñ□.%rò□í─)J

Wbÿ□Cò□óDÄ□âò□ñD╣□□ò□áD□%·)╤Wë,%·(╤[ëL%£J⌠Ró°□=öΦ«Då□ΘJtS┬íDW%╥ö░+aS"Uë□%
║(aU"Yë╬J$)╤IëD%□öêW"NëX%bö░(□¡Dö□æJD(□«Dÿ□íJä(□¼DÉ□f%LJΦJhJ□%╚/D¢□çò8ñ─/
Jⁿ¼─A%■í─OJⁿ]ë-
ò°Aë□J|»─▀ö°Nëoò°Fë»ò╪»─>%╛RΓ»J|⌐─□J|«─gJ|¬─'J|¼─GJ|¿─^%>PΓ}%▐SΓ/J╝½─;J╝¡
─[J╝⌐─□J╝«─kJ╝¬─+J╝¼─KJ╝¿──%^PΓy%₧SΓY%₧QΓi%₧RΓI%₧Pb╖□□VΓq%v)▒Së╟öxTëGöxXë
çöxPë□J┤*▒]ë□ö╕_ëmJlU┬°Dï□^%εSΓ^%εQbï□¢ò╕[ë╗ö╕Së;ö╕]ë█ö╕Uë[ö╕Yë¢ö╪ñ─ìJ▄á─
⌡J\°─┤J\ú─╒J\Ñ─òJ\í─σJ\ª─ÑJⁿIëKö╕Xëïö╕PëìJ\á─·J4+q₧□τ*▒Aë⌡J¼SB□{ä:÷□u∞□Ω╪
#╘▒G¿cÅP╟-íÄ=B□{ä:÷□u∞□Ω╪#╘▒G¿cÅP╟-íÄ=B□{ä:÷ê%J¿≤ÅPτ-
í╬?B¥□ä: □u■□Ωⁿ#╘·G¿≤ÅPτí╬?B¥□ä: □u■□Ωⁿ#╘·G¿≤ÅPτí╬?B¥□ä: □u■□Ωⁿ#╘·G¿≤ÅPτ-
í╬?B¥□ä: □u■□Ωⁿ#╘·G¿cÅP╟-íÄ=B¥vä:φ□u┌□Ω┤#╘iG¿╙ÄP°□íN;B¥vD▐V)Z╡s|⌐#l83√R□@
gqΩL_ΩPP□°╬`ZδKì□¡ß╘jª╙ÖV1¡⌠Ñî□¡≡ÑΣüû3-cjΣ╝Ñ£j`Z┬╬┼╛öQáEL
Ö□pæ·L⌡L≤|]□@sÖµ0═fÜ┼TτδÆ□¬σT
S5╙Lª*ªJª□L╙╣▐4NMeÜ┬T┴T╬Σa:ìi2ô¢⌐î⌐öi□S S1╙Dª LπÖNe□╟4╓g-
□□1ì±Y╟éF3□·¼π@□>δ⌐á|ª<ªQ£7Æδ╣ÿr╣▐□ªSÿås╓aLC╣·□ª□ª┴Lâÿ▓9╪@ª□□Ñ?S?ª╛□,ï⌐□╫
δ═ö╓ΣdΩ┼╘ô⌐□Sw

¥┴ö╬1╗19ÿ║rΦ4&;╫│1Ñ2Ñ0ua▓2%√Æ'Ç:3%·Æ'é:1%▓3ü)₧¥qL▒L1£gaèfg□S$S□τà3à1àr^□S
0SÉ»s1╚∞δ\□21Θ∞╘8%ÿ╚ ╤╞t╪("

qΩ□ªƒÖ

r▌?8⌡□╙▀Ö~d·┴ùT□:αK*□}╧┌┐1}╟⌠-
τ}├⌐»Ö÷3φπ╜»ÿ■╜╚╬/Ö╛`·£Θ3.≥)°>ß╘╟£·êΘCª╜£≈□╙√∞|ÅΘ/L∩2╜├E▐µ╘[Lo·:¥□z├╫i2Φuª
╫╪·*╙+L/3╜─E^d┌├╬□ÿ₧gzÄΘY.≥

╙╙∞|èΘIª'ÿv3²ÖK>╬⌐]L;Ö-π╝GÖ-aτ├L□1=╚┤â⌐òKnτ╘□L≈3mc┌ΩK╟□·|ëS@-L^ª√ÿεe║çi
╙fª╗}ë╪»┼]□σNª;8∩vª█ÿne║àΘfª¢ÿ61▌╚┴nα(╫3]╟y╫2]├t5╙U\ßJN]┴t9╙e£w)G·□╙%£w1╙
EL□2md║ÇK₧╧⌐fª≤ÿ╬e┌└┤▐ùP□ZτKÿ :çΘl_B□Φ,ª3} nPô/□¢▒8├ù0□┤ûi
W_=⌡NgZσK¿□¡ΣΩ+ÿû3-cjdZ╩╘└íùp⌡┼Lï|
╒áà□l□ù£╧T╧4Åi.╙□«7¢i□╗¼Ä½╫2╒p╓jªÖLULòL3ÿªs°°q╦ª2MßNWpΦr╛æçΘ4nεd╛æ¢
úö1ò2Mb*±┼╗@┼╛xyçë╛x9╜'°Γ╧□ì≈┼≈□¥╩E╞1ì⌡┼π\ è85åi4;
}±kA□╛°
á|_ⁿ□á<_|□hö/╢□4Æ╓┼ö╦4┬□ï≈╗8àS├}1σáaLC}1rj

a╨±┼î□
÷┼x@â|1□ál╬□╚4└□ôΩ╧%√·bd╟· ·bΣ┌╠bΩ├╒{≤□2Ö£□¼□SO

╓â⌐;S□S║/FÄR7&□╟∞╩1╙8ÿ¥ú╪ÿR╣^□S□&+S2Sgƒe□(╓gÖ

ΩΣ┃╠□%2%0┼3┼1┼rà□«`ag4S□S$S□ù

τÆa∞

e□a□f□ΓÆf.ib°╬ñ1  &r╡E╧┤I□Ä«┤║□è«▒²□²3p□°□|?┴≈wαGα□α□ⁿ▀□□C▐wH□
|□|
∞ç□≡□≥■è⌠ù└□└τ└gQ│lƒF═╣}□|

|□|□■^≡□L√L{H □n.≡

≡6≡VΣ<█¢æ²loÇ Å¼╖╜□Öa{□x□·σH°φ%αE`□≥_Ç∩·╚·╢τáƒà~□·Θ╚╣╢°"τ╪₧î£m{"rûm7Ω■□±-
□v□«╢¥╕>□<□<□▒╪÷p─□█C□
╢□#û┌v□¡└v°□εG▐6Σmà╧□┤□^α╛≡ò╢{├W┘ε_m█□╛╞╢9|¡φnα.αNα

αvα╢≡▐╟[┴╖□7ú╬MαMß≤l7B▀□}=p□⌠╡êu
b]ìXW┴w%p□p9p□p)≡'╘╗□±.

¢`╗(lóφ┬░Y╢ìa╖┘.□╗├╢NO╖¥úτ╪╬□9╢│▄Mε377╣╧p»q»▌╝╞□╛Fä»▒«□╗µ⌠5¢╫╝╗╞□□□╢┌╜╧}·
µUεòεσε□¢ù╗╘╓S¥╢╬5▄┘lsú█╘□▀╕┤Q?╨(67èⁿF╤╖Qh╘hi┤7Ω□K▌K▄
¢ù╕iI±Æª%▐%ªa▐%{ùh┤Dä╡╢φ▄║─ÜZ□v¡^□i)\∞^Φ^┤yí{A▌|≈\4pN╬,≈∞═│▄u95ε┌═5εΩ£Öε¬
£J≈î£iεΘ¢°╣°µT╕°l«pùτx▄°íⁿΣ£2╖{sÖ╗4°──isë{b╬□≈□°╟τîsƒ║y£{l╬□w╤µ1ε╤9àε□t₧║
X║╪╗Φ□┘Ç ]╨□▓èQ}¡.δ^δ╗V□Y╝╔¥V=6:┘û¼⌡îε,≥&v□
;ƒ╤·ó╬zt╥ïIÜ+⌐gfat°□;}╨Θ¢Nª8W°₧}□)╤ÆhO╘□d▀□╟ù□□£¢╧▄/█Φ½─╤æQ□¥ ó□l Z┴7
b=Θ┬.äⁿaP╗╨CPf¢H░□ΩÅ□·π~f□Γb*sÄk
íIπ╝!┼S╝Γ\oz⌐╝║J*╝Aτz╔]1┼╙"─àσ╞═$xπσ□ò□Θu□7R╨¿q▐öRÅO▀┤)eT·8oô╘.ùí█ñ&□)wNo
hlpz\°P╞▐ÿoc⌠ä╟,/Z┤Φh□□▌□¡╣ó╤°Φ([ö&/mQ║+¬▀α┬ΦH[ñ&/mæzó+□─┘┐ε□┼eà╤ß╢p═¥□>1
\sàτµ□║┬{≈─ⁿU?╗╨~≥¥¥K°π2╜a⌐╙°F¬\4╩ñSzσw├RñσWúæ&τo□□□═hÇ-
U╬Ñ┐]δ□║ë?║□ ²╞?╔3▓M;çj┤│ü│Ç3ü&α

`–░□X
£

¼□V□+Çσ└2á□X□4□ïüE└B`□0¿□µ□sü9└l`□P□╘□5@50□¿□*ü□└t`□0□ÿ□T□σÇ□8
ÿ

╕ü2á□ÿ□ö□┼└D`□0-8□□□î□èÇ1└há□(□≥ü<`□0□p□╣└□α□`80

□rÇ┴└ □□□

□·□²Ç╛@□╨□Φ
d□Ná□╨□Φ□t□2Çtá□α□║□iÇ□░□⌐@□╨□░□╔@g Φ□$□ @<□□─□1Ç□ê□óÇH □□□┬ÇP
□□□é□3`□┘å½

hÇ□êj□|Γ0p□°□°□8□$^{n}$□° °;≡#≡□p□°-°□≡□≡-≡
≡5░╪□|□$^{n}$□°□°□°□°

°□°□°□°□°□╪
|□╝□╝□n□x□x□x□x
x□x□x□x
x□x□x□x x□╪□╝□<□<□<
<□<
<□< <□∞□■

<

∞□v□Å□Å□Å□□□□□□□□;ÇV`;≡□p?░
╪□°Ç□└
▄□▄
▄□l□6□w□w□w□w□╖□╖□╖□╖□7□7□¢Ç□ü□ÇδüδÇkükÇ½ü½Ç+ü+Ç╤ü╤ÇKü?□ù□□□□□□□□□ü
Ç≤üfα<α\`□░-XG5#¢□╓┐└·□X □δ_`²
¼□ü⌡/░▀□╓┐└·□X □δ_`²
¼□ü⌡/░▀□╓┐└·□K□∞□□{Ç└─ ░□□∞□□{Ç└─ ░□□∞□□{Ç└─ ░□□∞□□{Ç└─ ░□□∞□□{Ç└─ ░□□∞□□
{Ç└─ ░□□∞□□{Ç└─ ░□□∞□□{Ç└─ ░□□¼□ü⌡/░▀□╓╛└┌□X√□k_`φ
¼}ü╡/░÷□╓╛└┌ ú≈ß r+ ú□≡_n╘╨╨ε`&-
i╞t"□╛üΦ≡Ñ□~SÑÿµR□5ßk=mñKΘ1zùf╥┘PW╙&║¥ε"/φógΦ═ôⁿ═ùô▓├+═≤)B▀NA□G╘v░m ß█üVs
T;╧ÑH┼Ö∞G=mû╢»Å±}}°╥6╤ß╓áX□3ΩFj»└√╝8╘v□»\ñ█□╓┤╢□:┌¿±]≡
ç∩;|╟1cPB□4àª╥4¬ñ*⌠_▀F╬□î╠<¬°·┤└H-
@▌,\δÉÜaⁿ╓RìíÅûZHïÇ%┤ö□i□╛□A7°S2o▒ænñσ°ZA+i□¥N½iì ║▄≡¼F╬*#╜□XKgα╓£Ig□J1{╬
ªsh□₧┌□:ù╬√═╘yGT3¥O□α9_H□¥Po∞É║□_ù╨ƒ0-
.ú╤Θ□║□≤ΓZ║ε□∩òå □║ün─£æyù├súídε╞⌠$▌O≈╥}⌠Ç1û╒□5-□5.u╞□╩▀/[ì-₧▌«┼<~╤Åî╓Z⌠]
÷¡┘▀╙□≡ƒ╒«╞2 8╨Ægú$Gßτ ú¼9f$.FX-
φ□°.7·□╘█~T~╤½╞π║v#s¡æÆΩX∩ë⌠□t=VαM╕╩QòΩfhV7□║╜ å#e7□Θ[ΦV║
╧Γ

C)f╧φ╨w╨¥X█w╙f┌é»ú║╜b╛ùε1₧£ùZ╚G[i□₧Σ□┤¥Z
οσ□╧┐ ╒∩≈□±∞á□Θ!╟ÉGi'vÜ╟±Ñ<Å└≈ÿ▀╗█≡q·q·3╥▓□°₧ñ°░C=K╧╤≤⌠"=ü╘-π·4R/╤+⌠*╜)"í
^ª/q=D/Ö?í(□ëÅ □b£»úΘ4² µεv¼Öô)ü6╡²╚╢╜φ'}

╒ë2□ ╖α)mú
≡ë}┴╤Æ┬FaªÅ(₧╢╡²¿O□≈8⌠Äy÷ß¢█╛!3v═□²□∞r:□╙□□O□ΦJ∩:°τaè━)%æåè√∩O╚╧□Θ□ⁿ(N
□┘qå  !!≥\╤&-r{rr«c{v╨F=ª¿U⌠▐û□╝□°≤▄C∩┌ôuΦ²²░C▓÷ï¼≈>| C╦w{båd
°≡╡□√⌡╡║Γô#╖╫új╢c{}╢-┤░^Å╓ò⌡]í⌡╣.-xc=é$σ:ô≈8≈d9≈8□╞┘╖_╣êIï1□Ñ□□╟□9║÷╤▓╗g

□0á □-{`åúköf°□

□<B╨?U╙πògä&╙B□σù□}Γí m¡#w≥□sjrt|dÉYδÆ□█{x║ÑtJ·≡>)┴zpÉn

ε1xT╫q⌡□]▀ ÄIIHLë
ëMILHë
>⌠«9Ωα▀╟Q?τÖΩ□╛L□□65╖¢~UXêf□□jMMΩ▄kXZ╤ΣΦ8ï)<╬□ô□□□□□╤#□Ωí⌡ ]dî.
□δ╨x

°úφái¡9₧‖R□²EÄ√

Ω╓÷┼║□ï8 ╤╤Ω□□¡m▀n
ç□W"

┬ò,U║E^#ìkäqu⌡□Θ2;3\î∩µ╚H?□□-æ╘5┼□□)□M□□aë╨εs<µx╤í;"□□▒)ôb▌f7σµµ╞

□Æò5mZL°!1É1□,√√╟

Φ╫W8°·▀▄N°╒òèÉ□Θ□Ω█╟l'I□:□╞ë(xxΘëëA╞□δ«°ΘQ║úkF╞á┴éS°`ç₧fj

□ût¢-
=.╘┤≡╨gs⌡░8Gùö⌠h□"|ª╚╬▌Sφ╜ÆúL°ï□─π°$ZúLzpD¿□v°Ö╨╙Pô9╧Üh≥àGàΦzHt°╞C°c6o!2
╟δTrR

²]Ä¡+┘ûd□πmûhyë─%)□□;FJ■ùwWÅΣ□□≥□\╚OH□╧öà3eßLY8S□╫öà3─çhj█y?4e

└s┌èÆαo╖F√9╥α╖F□ⁿ┼╥p╓Ü┼□╢)|g╕□₧▄²@┐~┴▌î□δ/□╪*┬[é╦(w□«▒bå꼼i□C▌ 5'
╡□£CX╦□□û▄»√üzä░╚□█Ω-%┴2è»-a░prì□CΣÜëÅ29╥║fd╠

□4
c¥ □O¬.□÷╤□Ä□╣rΓÄJô░σL¼^\t° ▌N={v□□K/½εƒΦ□┘+{jAÅ├çÆs*╞·vτM□╘yB·Φy%{

≤ΣeêåSfM□╤+┴╓▌tVw[f┘¬±}╩Fτ─åeOZáë¼S│╗□₧µ□6±╨{C=├mçs║

₧$□║¬φ[Sä9□√ì▒╫lφB├£■Qt·G□╝OÄ"°k9èN (:╒□`ùM□YöF□"╙□WjzH⌠ólΩ+·┤äN╞µ≤┌~
æ┼├eyc7F¼%-
⌐Udm¡Oï╦h□Ö█ΩπJ│M¡ó╫╓·∞╨╛≥?:╒ú&□n╖SBN╫°¿áv;GPé□'æ{LB|¬&GKN]Säf

ëw═8╜hφs□ì/╜Γσ3rµV□ZC╠║)$<$¬ ─┼□'o¼□£]}±ö±
%□úâ├é⌠φûñ╪¿°₧▌¡e╖~w²M┐▄75┴▐╦□□ù□□▀%.┤{V≈é⌡╗Vƒ■╚□#3▓2ébRσ □A╬σï0ùc╔FW□39%
7M─╔·□'τg\<F*.□├□ùä1è{H╬OJµ□M÷Åh▓□^&√τe▓□Dô-
╥b(□#□ßï*▒╢èî□3╧E5é»⌐y7═┌□àaî╪V □H·□≡6«j▌yif$ì-ú╤cF∩≈├▓-
[r∞╙O·□[╢σ8╢□;Icτa;Åf╥$mB₧ñi□□ö╥□M
-σuÇ□=\ ë¥ÿ□┌₧∩ª-□┬φwO╬ß□-üKΘ-
¿┤£ShÜD╡{╧îd9Å╥Bⁿ}╥d4{╧▐k²k¡□¡╜g⌠C*xσIQÑαMå□╢□jT□░ε-
²·[O□▐ö`□·µδ_□Ü¡█▀Σ▒∩₧8°Σφi∞▒o^··░

á▒»╛■Φû┘ú}ù┘╓├ □É≥φ½∩ß╤┴╟#╙▌p▐'(N┴°╟èSf┼)│ΓöYqs□;½s!n□□F|èπ□r

ì₧≥□ ╨┘)æ)qûUî* 1┬┴KgEp¡^<#JW□ùVè@╥□┘ó□ü├bÇ)╬□_NhtT!î>GΘ4ät£í=^;∩7╙1□╓-
¥=╥9Y║ÉÑî

τ`╒W~OΘ(ò□|□▀ëx@d□≤╛σΩ¢─^ò□iC^ò⌡φt□x¿o-Ω¢ç■ê╫0≡□╟ò□s═ΦÉg#¿7Æë¼ïα□â"%â"%âb
M□┼Ü

èö

pf|ó

¡□j╒╟°²ΘDδ╙¿□░□
□;Ö-1□[9æ□àh□□┼╨BS¦╙·±±│%τ□Ñτç}£□a'¬VpxJTÑ5shlFLÅ$`O'┼─¿îû│q╢\ó
∞¬╛üà év'╓Öà-l ▌□ b/A3□│xΓε╒[ƒⁿH█Æ²▀Ün>░¿p₧e   5ê□ƒ╫ZM□SπÜ
S╡Åⁿ϶½úπ▀╙╧?╨wdzë]CLp.Ä

Wçù▐≤╟÷â╧□φr╣╨}■ P□M□¥ª□g□╗ⁿ<3■φ╖Ä?÷▐≈╓█□1╗-
æ□ê«□□┌é¼çÜ8╙f□┤}╫å#èä□E┬êéCD┴!óH□y□XùµΩsg,ΦÇ╞8,àZ4□ƒ7⌐S╥I□2┘δ□ß$╗□
║Æ6√□ê-╡┼g□|f┌

f□└┬╨─/ïGpö□é≈âQ┌σQ~-
▒╚├│(├│(├│(├│(├│⎣² ê┌0lÖCπ'H╔≡╤─╣Rñ9e□&ßO'ER╢⌠Γ(eCWF)Y□ ▌OÇX|σ¼-F¢²<□*·2

╬²f□°□╓²¥Γp/}E═:e╔Æq`█╞╚□J□7«k▌╤èΘjjlëäªÜτφs╨üA°╗â╡

úü□óü□óü□óü□óü-Q□╡□"vFÇ*
╓□iy¢.┴╫VôPtsΦÉgEæ□╡Ö□kI□□□π.α.╞╥□¢nIñRÉ□ì□□{├>°·N□¿3ÇB╨□╙□□Xα2$÷âª
□Æ□I╞i│G░·8□+ñp¡┼e╢╕=Z¼╨â□'"≡^Ä¬tl÷╓□y5║Gà-╙┌=aa¢┴┴1≤¿╪tΘ8Ñíp□D□@O-
¥□ó"╚╪úÄ╤c°□ε□A½µ\□Y□Ç□▓H
≥ä□ë#□âY□╗⌠mP╛u╥≈[P∞fE∞fI∞nMuu□è=╓□α□╕0idα□╕$
/1"ε┼├ÜjCä□á?à□ôd□┼|¥ö□)Ho⌠╫4αò□EÖ╨ó

ç#□½┎r□ü‖q[*\åOΓÉ╬b╫5╪#üÇÑ░┘█ε─0îµ<<∩1╤ò÷aW─πb╤FW}▓ûG1□ⁿ"X╜&‖╟

□╗╓òî`┐I□┤)√H▀σ *9Ω'ú~ì-µ╣≥ú║╓uπë┴□□─₧□|ö□.êé±□D¡□□▓¥H

·Æ□╖éñ"UR□3⌐ÇÖT└L*R%í╚l¼
è╘□æ∞22:4τé╝╙□╖Ä"l□:WÆd□Ç±¥▓

1e□M□¿▒,ªæ≡ΩY□\nü╫╧êRâkcZα┌@Fö□oⁿ┼╟₧ ▒≈!5τ□áUW╪QK┼└ûm╡╪l╙╪xσù>┐tSw□□h²π╖5
□¬Kp□□ólmk÷ì
▐Z°┐r1┌3    ƒè┐┌«·╕╧□óy□≥î□╫4>S□╬5□τ□à<&□yL□┬'□µ¢╤
^gì≤░□w&□□&□□&òáûTéZR□a□>□f≥itU0·╪FBD□tn:□╟╬ƒâóJù¿=|

K□i═ÇF6╪□$óá]å╨Θ□$τ°ô□ù±╝²□ö□$╓*E`□▓£'*⌠Z%╤δ2¬□f¡aH≤¬]G[k-
Öⁿ╟□|¬½∩°»Å□Ω▀∩╧r░╓è▐█▓Q3]°vΣû╧φ╪±╢╡░≡Ω╧▌╡≤░ëΦφ6□K·┌V7╖+Gƒ°█ù╖°▌╡ú_ ╥□åÄ-
▌Q╒▄Θ7p□∞7╖²αSKG>²╘µ█ƒ╜w`·²?Dd⌠□ZÇ╛zñ
·í$e╖░Ümáüh□áö□$D5@⌐7@17□y¥ë┴╟'╓╞B┘é#Væ1½└öU`╩*2fß÷^g╡q

ÑO∩╚áÖî¡□ák╓7dS\)□Σx╛$Φ▓L□□·du□6¥□AC-
lyZTÜBS/e/E□□┴½±δ0j╡╣q%¢░qV+Z□Ää├E╛⌐%═A╖▌g╓□{,U¡╥¢ε(ó□≡O«╢▌▐□╟╥HácM┌[W□5∩
╥╙à+]╥ä╢╘â▀∞Ü∞≡□gJ□S□n¼╢n¼-pσ□%T□~ª┬uïG╖w╢o□l4δπ═Kk
┐
║≡Åσ╢╪(▓É≤5-□^╡τj-ƒ□8∩Eúr4n□⌐╝□$τφè8█□1╖+>╡]□k√□VÖë'3£□═%3,╚αô┴$παa[□

i

ú□~Ç&

¿:╟SX-îk°□□πxεöá|¢σ∩╙□□═!L⌡╙h□i□ñ.£╤▓▐□┤!úe╨□
w~hαQ□█└Z¢!·mw¿b#V`□'T□φ□Ω╩┃0ƒÅ╟╟ìy#t1Pï%]┬-
JdßDC5▄Å'▓Ç÷à╧êR»1╪φ□QΩW□;□9â╟□Aδ╕╥⌡<□$èå$╫t¬╓¢$`$>┘╣τ+πφ█╟ÜlZ@□i}j┘╬╛┼π¥
┴Σ≡û█6□°Ü╢<╕<>6╨╟æ□åôZJ¢Φ□o¼_VgOÄ▄z█¡#)tδ-
≈üΣ╓δτC-½╤D·ú□w├▓T├╥ª┌Tδ≥¥âCwÄV□□□°ey╬□≥2g└σ¬Θ□╒/mNªZFvBfh□^■τ└╬ⁿ╙aë□≥□╙╙
Y(≈□╙□?░╤çΣä╜·▄,┤3╥□╙□ùΓ╒ôÇ*╛-
ë≈·╕±,ö±I╥eÆr□W╤Å'τ□ôÆü°è.I"f?□─î.□/2IpñúU*≡ü-
Ñ□Aô≤æK_,í|□═:9N«Rüy>y5O∞□▄2Ä╟╩>{]□Ωà▐├
╜ë□┬╥
Öû□"□>GÜaæ

á╗Håâ□┼êU□àU□àU□àU□àU□à⌡)╠□┘≥)╙û!<╒á
Mx╪8∞ÿ╟$Σ╧□Φ╬╟πeTz□^□□╙yÉ╡òσ□e|Uq╥e

a▀Æ├s╗╖~∩Pù£¡qtσ╙ε █▄█ CqIj>Nì■·#▀?▄╤║∩⌠-<Pö╘σ┐¼>╢¬¬rσæ1▄V"π@b~αü7□ë□æc▓─é╨
·Fâ¿□~çφh╘ååuhÑÇV≥¿0°8□Θ□║f╛x□-dL≡ö└
|8Σ□µU&9ç0Ñ█X□*¢□£=2>ÄÄÅÅ╞╟πÄ3Ñ╦xΘ:ΦH%·I@╬T__F:ôV+Iag□╜□qY}<╦Pxa□ìÜó~°╧ñ&
╨;Pt
N□WΩ Ωp┌
+l(íó╡4qR¬┴╤:=Ñgë6x-╓αα▄▟ □≈1#□╙N┘≈!"QbÇΩmΦ□□U□ª=└äå□ñiÿTfîû{¢3Ñ▀J╙Dτ-`~ª
6╪R⌡⌡
\i╛╜rébí
ƒ╤¬

□ƒ;d╒¬N I;f½□fp-τ╖□cFò□²[íñj⌠U∞ù6╖₧
@□_╕w╤«ª⌠╬□⌠#□=E¿⌠v+ê║F0∩ Käü╬ú≥╟gy[ä δµ04ú╢à╜α╜6¼Ö├Ü□!

ç\□æw□╞ΣÜ6mVmû½»
ÿ`úBé?□□╕ES┌n|U>Çî╪□Z0æw┼·6rÑ5□3Θ□σ╒êÅZX^%ñ≥*■K□7å}╛ÉÖ╞╟□ÖaB├□¥«Ç-ú
╤╌
□├G▄BÇ7iiⁿú╪w╤M═V0S£d╘·?¬□¿Z°□□^½°p□W□_{╕áü∩ìⁿ□°°*ßU*¼□%₧□N=ìßê□±`╓S░ä:ç╡
Ç·jφæ▀╓╓Rí╫îS⌐w¿Mσò╬≤□└┴□πàñ╝.P□·¡T╓

╜&□°¿╘;"╕·□□4±□□4±»:SKVL╣□■°9□4ú╤╡çF+╣`}
>É÷ ┘R╡ñ·[│Θ÷¿Ñ╔╤0╥⌡╠»□uÑ\h¬ntI╥ot·≡»·\■«╔÷HWcòPts«Φ\ë>□hîZ
╧:¬Ü
²±Äj╛≡5k╝□b~█╒╖≡╕ë□d□▓□Jß$ÅDµ░╓îå▒^J╕┌\ÿ╤?çÜ@Φ▌ê╝δ¡¡┌┼j+τ╨E'¿-░X9Ptsù>□Q:□
òº]╓Kóïò□hDvc-÷«XK┴δOèá┴55╩□òæ¢╒(±╗i{]∩xâx≥╬εPts├°──X_ô]
□DJ□n□╧t▀1TÖ□▌╙█2╓□╒æ┤□ £╦g≈9╣PtsO╛tΣ«ƒ▐╫gt·∞□ƒ╔╬╥Ptsá╗a╚#π□-ÖJ╣□n□$±□≈□□ù□□`
m▓U^9Γ░4,?bµîZ═ ╘O9.¬6□+è≥□□úτ/è·)òπó¿┌┤░é□╕q□□┐▄{╧Å>}IR#{╧┐▌▌⌡╝ΦèÅï□>░±╪
¬J╠s∩OÅ╡╥□[r⌠┘C╞≈nj╝ⁿfφ⌠gínα°⌠`|ò╙J       ívá□s╞¼÷r^

Q[redacted]

ô┬╖║⌐╚╕Σ<.╤─9ΦY□Tra√▀DpÖNxW╘Mæ└□╔2P.,ê╟cra¥Pè├zé╥ÆW■□τÇÖ(-□£ëû*¼C7QZ╕r□Ä-
E┐□╕□╤□ñM╔≤íî

ôI0╨àƒRF;╟□F¬≡u╩(H3╗ ·-÷□ÿY□Y&═L┼┬ÖÖ£Z¡□q:T□Y╔F\≥N┘6/,x□°e`U□Epìù╕$JW-
H□╔≈½sZ▒╖

å┬>t□╓└I0dß□Üâa╥L□⌡\4□≡_□╜à□┌(p&;□√rZ₧,ì ╪τ≥┴▒ïW▀▁▀$ÆH□┘!╒□▄n□□ƒ-
Dóå9lqF│(≡Äá□□5□°□EπF3░ç□5[□
A▌╚□□<$,>f┤Bα□Qj╨□[╠êì□k`¢ôb═ûó«`¼╝Y╓1OOÿ`EüñΣzM╤┤≡7UöÜ0╘µ╢σF∩Y_╫0·╓íΩ⌡æ?
□uê«╡zì¼o┘≥╤╪¥/▌█;°└K□:o_╤`╓α≈r

#φ□╣Üo}x╒åG6-▓ZP7PT)σ≥□&=.╨dτ┤╣{_╪□τ╧-□┤x<£G╤-
áma$ü¼ût□`αCû┥á□╛╨□▒UL□íâP_□BñóJR╓≡⌐èi&(√æδ├╞┬·T╣éK5╜╥!■s┌□┤δ5S□╖Tç□z□┤ê□
╝f□²-e÷J┴╘VÆ╞
à╞Γ1■ù□╬'╤/□Åò□ó²`å□$¿≡□03ìqZÜ
╨-£Ç⌠┐¢ûF√ïcQ├□Ç▒öFÇ □)-
æ≡ì╛,╕╙□╒╟░0≥□αûH,╟J∩Σ╜·□·%p (R7□□ù>0═C▀¼┌"WBσq□ªU╝ΣüU[èEP□EΣé|e~l_▓/^█c»
ë╣└`@■íªh│#hwGm□ .╗▌╤╤h⌡ΦmK+)¡^╦□m□½¢Ñ□âÄ
6e░_ö□/πα╙`öïæ yö□l▌LUòuqΩ□¼□d-Z╠îX□
6Ö╤!╓Φ┤_╦:°┘□-Σ□¢±φΣà□î▓Æíö_ñ¼╓û╦7é▐□□╧:¡□jQ□□█O╙\@p□m:Uß╨uê╪N□¼^▐ετ╘
±R□╛üε!i□τ)α+p└ñ0÷╧ƒΘδ□UXä■□£┼ßYR½g╡à;□jZ»╙(>□√□É□_╘□□¡Ç╥M□□╫ƒ
6□½╓PW║i□₧=)□¢µ
╓Σ
Ω╒╪/îåé╟□£7╞}û□üç┬□Z□╓σ╕(╓!δA5
|φbΣV8éÖJKU□@ΓjFφ╫%4UU■:
ⁿï°□MUY╡╕+<σ┌lT□Q¬v&MΘ□áë4░OV¬âd

╫^^¼
_[?V╪▌√╒Å¡□╒4┼ymé╫DaàO□ü¿┼iRπàG1╨Σ□□Åë□≤ó°╥╟½╤□ü&□┴□sn□é≤V│τ≥Qå□`%±âù?Y:√
ó■

╟WΩ░╣+∞Z»┐Φƒ▐□□iBrÆ□≥Öα├└Nóf

2 Çó┃~Z[aâQ□■T

E┼3e┤σ┐/@!
Ç\█5╤½?╫╟Vó≤┌°╝2±╖ⁿ÷;<acß⌡Φ`□E1öb¥V▌□g{ÉuÿY║□_□├P≡Å49m╝ï%;ⁿ^Å□╙÷?û≤
≈⌡≈·»<S>W┌└□
┴í»

GW¼□ìó∩H╡ □µu□▓±Ω¢D□êZ□□A▓p ▌╧"f¼ 8□7°╘ □j8i╪□ÿC
è (□₧□□□£¥□
□U≡τÆ#)ÅGEOB]S;$║┌∩ⁿ┴■²g

4w□■┴■▌│□3'}}{W«▄╫≡÷â∩²9µ>≥┐-\┌⌡±-
;tεüÑ]╟^╕□σCbsf√CC╖<▓¡⌐c╟├É°□ì▌□□∞□l}⌐╟╒╓°ü≈`┴α¢ü╩╪╚;*□□·½eè┘\^P.▓□U□□X□c
²Uö.·{%dX₧ú╧kr°¡uô≈O-
/║ï0ÅΩ□]▐╞[2■S□¡ûä⌡3_lΩ¡□░▀Å□╣%Qx░\%$┼ñûN≈e7░*Ua¢°í_╓┼□ΓYáï□ÆF╓I□Bφe├≡▌

êú□╛Oî⌡¬Ö8□╢un"°E0@'g≤┼·/α

J╤□\gìKΩ▒ndαÑ°Dx-▀v6>_φU∞∩□╒^ÇGX}╝Fi╧Æz-╖or╨-
;<░ ╠╛ÆεLíz ó╜φz}ß▀KZ∞□▀□r■5□╖Ñ║Ñ-`
v}Γ'╟

²□h≥π/-φ<░uu░║▌Bå░■ò╟╖□¡~f┘Ü╧è═□;-R┤·8╨j□0⌐)┘7j0╦L¡1╬╔┴╫Uä¢$Rhp╞┘╫ÜÜlΘ┐B¢
ô╝R1□╗Éä9X·?Ñ≡`è7▒»ëαJo·»órφìK▓æ□ödK┘ÿ
êñ,#├°-!°├g╤αúå`M{▌ª"□□]┤»√╪-5«E╣ZGU╚g\Ñí■d⌐Θ╧<|_δ╥ñ└Q└□ßj╜÷/□]
{a░äèƒ°\ßεMφ0W3j}5ÖΦ │
╪»□═qí≡□!□▀.▄{⌡M∞2└G?rTûK□fÜ
╫àδ⌠.°v

D□\ò.úN╖^tu¬Γ□ü√fO{╣□

πÇ_╫I□,%g@4╥n□IH28æû┌Ω─t╝⌡ó(5τ`√S"□yé«h▐gσ"τ┘°=°┬√╘7▒╦M□∩□I¡=-2R*

□▄Y[╒╜╛╣*╫αëw»-_▌SQ╖µ`╢║b╕│V/²«ª╘▒ûßT$S╓W÷¼₧X▌SëF·v
VÜ□Nú╓h1Ü]f╡+α▓╞Ü┬▒ûD¿"╡d}{fK_╟h□

Zû7r ┐|╗∞ûP╩□o¡ÄDô]□╨╧;□╛Z□╛╝HúΣ3□□└Θö╒@□Aî=σÿ╥lV╩ög▀~-V'
□ⁿaFö~Ö»NÆ7/N╢□⌠à
jôO░{╟tßB1□┬^ç║╞□□≥]>R╥·!Ü□┘ÉâÑ`P□ú√ÆöEäüG╦╙Ö°□πÇ7╢bµYMx┌8φÿw┼mEW<
□ÇE┬y/ⁿAïä°¢-╖}q├┌╟╖7□°≥vG□û¼M°'║|4τσ]-ÄB█⌡┘-
ïS╙□▀ëφ(Rê+Å»ƒε≥√╗&Wb█╦ÿ¥□H÷□0v?╥/╒

□□Ç╫ƒg²6»╞f□⌐PFú╡╣ª¡*à□├ΩùT□Æ
CRUΦLΘ≈k□}╪M□}╖□□[╪π»α□ó≡yÆ0□╜₧ÇÖ┬╤$□□ü┌∞w╣|:ér╥□Θ⌠

■╝╒íô6┌]■□╛FúSè|(╥p⌡=Æ□coF÷I⌡MuB├ ═55Lr

}+úifl╝.□□0■9╟Üayªa║b║&á┼╦ô□@qJ3□□░╬┼□σcS┌ñÉúk█□╫ü╦gè□≡δµ╟Ñ8egír□gO╝FΦ∞Q╖
?╬k±▀ατ      åÅz╝q;□┼ íPS╪δ÷q□▄▀╪ƒp┌Σs9²&□□□²□NsP*zîTñbd░≈«¿□├5□╥\~□-
╤ΩαY¥·≥┐╩╟ä▌a□╥¬□Ü~□H+à∞ûQ□─□#v$è!□MRH%φα□╤├╫µ≡îT d□╞□

3U╙aå≤Lsσt^Hñ□v-□@┬□≡G□¢4╨╘╝A9⌐Ü▀z□íµ≈^ûë╦6_□E±╖

╕)µ±ä¡□╒∩╡┌▀□Zs└εëÜp=ZY°□ú2E□.┐Eú·àü9Oh8└û├□R[°]½¥╫⌐p@ö╨Å┌là╗iX7╘±<·*·□⌐Ü
□╙uß╦v;:□+èñ▐n.ñül`╒⌠

⌐j┌#{i□╞¥╘1v°6ƒ Å└╪Ñf<╙<iÜ&ïfÉx;²p■╨□J?²¥□≡<

0A=·m□0

C□┬~w╚ó&.c

α⌠ç⌠*-·píΣs╨C╪á\
□⌐L
·2¡%  ┬ └è∩Ü½y╝
▒□{ *┘ü╫╨ßΘHtαZ╡¡Äa╨ü:╕6R□ùEΩî░₧_7ç■-úG"□□é2□\=A□ò□└FeùEúR

o,«□4╬at╞╟┌₧GΩîuX╙su(Rç╓╒U╖W╠í└D^÷ú~?ßzú║»σ□fÇ@□J
k\┌«5╛sb╝┐Å∩l|b□□□ñσX`_□πÄîNkCδl╧ï░?┐╘íUDⁿ¿ò□}V╗▐□½√ÿûWD╪/ƒP□^k'╞σ▌[π2_'!
£□-*#F⌐E□□R╬□Æ≤ñΣ╚fàσ□╜═Φt╪=·ª□çzε□¬j▌⌡═-□¡╡K╙─
δ{k□ÜQ□öúctc▌·O,□□φ╙]S□₧U╥┌╖╖≡

CÆ

┃║¡;╘╝▒=╖ú/╘]╖læ□D5┌(□□ù=αΓ*W□Z~╓V╒□δ-ΘΦ□°Z
tΣ┼_B□!?ÆΓùSZ□òù#~ú,C╝>□ù"╩□UDY°ê(╖□"èB└≈□░Ad

╙ft =¬□■α╔htYOp

┼f╕>ⁿÅ╡pσO¡╥┬≥.yB=□□D</}ö╢═¥U6fd□Å≡□QεÇâ=£□╣╛ZⁿÅ"∞d□vóå╜£□A7□òÉ¬╛7▐ÜJ╩─ü\
P⌠⌡b*Jhε_ÖX╚⌠ó÷¥Å«è□u-Γ╒$f╥□"=+□≈▄Θ╥î7°G█Γ

▄/⌠/¼└ΩäÉ╥ö9pj≈╟₧▌▀d┤√y=╟¢"- ╘wµ;cw»î□π□Üs╔R%□Ñ┌ï∞F╬K╠{╙┌í[ß[Uk□å□τ□z±T$▓
╖ⁿ4z□í□δ╥f∞kπ·φ=mìâìXM.ô├□sì╣₧║╫ôS┘-0┼îf⌡□Γ─²9}NÇr├√ñx
¥W╛-%»>ÅÅ+╣□,c$□}■ⁿ□V^□±nÅτ┼φ=åFO#åΣî9î┴Ñ▐°┌^□A ½Ñ□0bΘ

╕ □▄C□n≡&±╢ö╝

É=.gd▓╫(O>%·R≤'┬aà¿□7□┐eüÆ¼╢pXQ□n!÷÷▄▀┐ ·@┐┬
▓r▐cª15=╡¡□û╨RΩ╣╤┌Çíel▒#X%ΘJ□J□Ñ□]¡h½Ét□5█╘?&i6#▐╖╘R╔╛╟╡□█□[R∩╫ß⌡²╜-
□?╣■╧+p¥

nC

□□]┴ò+«|¬xå°▀□µ⌐╧╞┌□j⌠¼¥ìx▄AÅ¼πÇñcïQ01B╚)íßΦ□≈ª)╩┘Y┘q√≥Z□Ñ╒δ□□□│□□{É_J□□]
ÜY□1α g¼╗₧┴"╙4┬□□Xæìÿxzç□ⁿ-╒<ì
□zV

ßY:¡Umφ╡τ'z┌bâ1¼ª)╙ä┼ÜbM⌡╒»√F▓⌡@K│╝□lNòôRòr□┤)·¢□ î░╝⌡□c□$BR╧□÷╝8╤cêyb□╥d
l□╕Éz⌐~]□²≈┬□£□ò;H ╬B□┤╓Y▀ì¬b äΩτ
i ╨ó □═Rfƒ├□α⌡dßΦ5·≈'µ⌡┐√«□í□t□e□ⁿ╜□é┤▐Px□▌╬hñ
-8ÑSú□)Φ«┼└Γ□ë□□□ù_E?ó╤⌐q£╥¬□▐Xx¬□b-2.T╧¬╢#çΣτYfÉ=[□q□îl├
▄=~1úM╡ñ□┴▀-sx5tª╓=m├â├X═Tf□□₧□₧Z;÷ç╛â┘╡╨r╒╗□ñ°╝┘%
╛□1[5╨Ö°╗Ñ┬□□D▓□□RB/?xÆ2^H□_8□┴xûò≡`7

{å1d╩8□@ ⌡□p∞□"╕├φ╥-t"╕G
ƒ□┴]¬αm2j▒jÇε╠ïαVRì□ #╣□╨Gö╘èY,èNa>l-â□í□∩öφ╠Ñ╕°ü▒â5X═╒╖□□□·Φpⁿ5□□Yπk
▌╢á╙B½h□°⌠╬H╥╤3Öq∩1ÿ□╡Ä┌#Tu─ó□╒é╗F¡┬Lî.┤╕Σ<èÄ╛<,□≡

ZΓ°îú+▐▒}╕║z⌠«□□□kτé▐é{τZ╡F¡╥≤&╖_°╙Rí■;6á∩yâ Oñ·Ü╟□□╬dw┼Γíñ▐$öCG

□µ≥`□á│ÿä□¢u □?«┌åäæ4≥□ë/y┌ÜP¡#
YR□ε
I├▌Mi╙ï╥É4Ña□AÉä□⌐□JÇN(□:í0°ä□á□sÿ&□□i╖6□q□·□°▓,┘□P.Γö~@≥#É□Iò╪□╧5@.□¿Éª╪
Éç-gD┘O□█╬êRcÿ╨□·sM]╢£⌡□KƒO□K>B╥l□■8┼:═≡·»₧GoÖ╝w,Ü▄≡α┌┴╖3ö┘├
^ô·ë╬Åv╡¡l□,uúφ┘ûLwDáσà5z╧└Φ╘'6∞z·h╤ÆNL[▄□ze╔╚X≤ååÖ«#╙-ªè╬Z ▌q
▌G□□ì#u╚□Æt+□⌡m⌡█δq╬
fÅ≡┬ç%8_%▄;V Ñ+?╘$±R└4.╬v┼┐□╟απ7│≡±¢:B!Hä┬âñ kÑoÖÿ□P▐>_σïçë□□∞9□}Ö@
┬Öx%▄╟┐▒N┐CÅΘ╒o8□ö2è─Iw▐^$ú╔Wπ2!Æ₧lÆ□α'*_□?"⌡□N╝"å√⌠ⁿ□"ó7Ω1□«w¬▀□¥2
□é□TbóπÑò╧¢Z□°□ñ^╥□à?□□«£tw∩□╧L⌡&□Éäπ□≡]⌡ú;3█┐q{c≤╬/O▐·≡║¬'≡}{Z╓┤·1

ï° ·≈ÄV[∞□J/ÿt£ü╤□<╫║□n «∩▀╡ñδÄ╧»ΣÄ□»╫M7└h□║ ·−v

D├fΣ□R6o5Bé)□Kç▓ß□~Kés(; □□p□≡□ñ5□í╣½/gLF□═à4· ·-{8_ô⌡µîY)□&! ëƒM╜-
s╙□▄*Éaδ5y□\Y□╬ï╩╡R(K╢]╖╥YεóJï□≤~I^σ─Ä□└¡P□w╟□¬≤Ω_ó╡jò╔≡□,n≡
0▌i4┬B°¥ü∞╢╛@G□&x□╬ªW⌐╡j>5╘╕A÷□ù X

0╕Ev□п□ìΘ

τP$E<°┌ç¼ù≈F═⌠⌠°GéP□╒î□F□╧r┐╔oB╥⌐j:┐║g$;ÿoδ

X□·t6ûsµ□I□@-╨√CÖ£M¥à^9%--(ï┴╬┌t^äm█□≤"l¥NΣ┼R{ià%₧ä.╜¡ê
┼□▀HJ╛¢□L▐Ç:▀╪7□ÜxNúú-₧¿╜»σ%┘oùñX□»òg0╗╡°▓├ó□□à¡╜áU3z<□╬╞;-└½π*ú╫
t▒q╟Σ⌡"╛^▄+╞?6□3¿ o╬δR├vcδτ╧): IÇ╓m╙πÆ

R⌐□╕övqf╡█ ⌐à╩╪V╒□╛N□⌠tNsé□╞I╥µ╛╡▌æⁿHOCg~ ▐Æ½╩□□;ôß┘ª□╓╧ÑΣé2Tëⁿd╨

Φb│╘ç]ä¥îD≥"Φ□□s▒¼#I-□v█èU≈╨═·7I~8h█□Γ┼*╘₧$e-
ÿ)kë}KJ!╘ñ:USkk]V═~_V▌≈»UX┼╪▒q√ΓT╠ª╟Qè⌡┌ßoÇ╙»:É≤    ∩╞╣ƒ╟q≡)╘
7▄╘T╓ì[□#(è╥╨□F#¡;]·╜Lτ□╜╤□`;□╦y╫╠'>1y|□·ε□##¡□c0≤Ü|x▓

Üæ║òi¥□□;ΓsΦ╗□ùg «□╟│□Σ□wOìφ╚∩╧▐Ü□╧
dm÷t.ö½□¬9c∩c╗{≥¬-)≈Æ╥»y;âJUΦ6dW□├Æ≈æ(□τ>₧=ⁿ@^ä¥∩▀æ□□voç²g┤"╝â¬'/é{H╣║W╝x□
┘□í%□òU*┴■²D∞∩□ßCX/VÇzu□╜Z⌡ä┤b*⌐╡o╒┴~mé;

L4_▌[╙zá
□□>ñCA░T5╘Z□XLO□
%X├S╓╬ -┌ªñs⌡╡:□▀□ε□□╫¿ a╤P├?∞□ +□ÿ┬O□Lq╙□y/╬ë%╙╙╪mê□±□?░b╙
╤d⌐»⌐□╩

Σ¢{╝ò·zâ¬>□╬ Æ±f?g╟□1r!·Ω█τ/$^{n}$

"┬:öm-╙ïα··╧╝h╙□[@+?o?□O.╪EQè╓°□P▄éèç□ε╫┴O½¡□╖+b╙hl□ù;bU¢▐G
=[║¼òAçå$0á□╓-r.i─(╖@ⁿ╘□å=ä¥╬ÉáV
íK╡∩'O╔Ñ□┤Zú5≥¼╫I╤□≡╧

^û¿ΩmIó□æôÆ¥.[V╡□╩qªr╝RDµ░φ°5òα/φü«÷αÜ*)εu╢ñ≈f
*Uτ«ⁿd╧Ü∞╨|owò7¥∩╠ªrEaùΓ-p¼τè"/µ┴?ô∞Rv╢Ö□Φ*□·Ü▄ò□ao╜+≤"∞»3¥□K=.êäáí²▄□╓╔√
¿ç╕AL╝^g¬╖╒╢r¥┘$¥IVU╪·≈l╙S4íÿ
à╨ná╥¬□P⌐□╙Φ@÷□v,i□*╡□`ò▐╟\t╫□╤□k╝≡ÉbC¢ÉG$□□Sl¿□8τM~ ª╓$╘1fφh¡1oÖäj╬«═/φI
╢µ|┘┼9αúë□┬á∞{ôèz┘TJ╓≡9αjü]╜□⌡ΩÉ¢□╒fù«═ïáçlk^ä}□░ôô"ΦE÷«╓óJA?7╫¬▓■h╡╧+] ñ
╒ ¡¢□═▒áS½┬1é╘Éj!µqU╣

î□╛wy═«æj╨H¡5□ì&çæRk╕¬▐^╝·C┘dß!┼&∩G₧ùl≥╚æë√╓B√█Ω□‖┌

t5□■ô¼-åè≥▐?╤>╤₧▄╝}δ▓╘â≈e∩╔Φ^;╝5┐█á┌¥¥╬¡╠⌡□YƒY<X¥¡╚
ÑÉ)√BháèΩ╬□Ö)╘^□=ò·<pO^ä╝ε▐*·G╢▀┼░c□+a╫BE^□µcÑ∞6í¡▐Xí¬¢¿ë╝ßF╡□─ö»7┘+:hz|e
PÉ⌠¿úX□ΦQ2=□□kTå□N□(□l>╗½╩m°□╓¡□╙k□±≥ì╡|9ù-□┼`2@áÉ□É▓□_÷íM]µZ¬
Ç#oùfî¥ΘΩJ┤ffA/"╦□
□FH$□╪VbkéÜ├─╙l□ⁿ-
□╠a├□°0▒r·Γⁿ TOvy~"╗4╫Ü¡╙æA╞¥czæεΓ:^)Ñ+□+ëV╝¥╝P□ô⌐┼y□t3▒</^▀□?▀S1▀√ ¼}
x□WÖΦ£□ìFú□I3zî▐╥Φ-Yûd·²è%╟Ä-·Ö8Iô╥8ÅÆöÆ*ë□°-
ÉRΦï╢t╗É6iΦ□ûφ^va┐╗l¢6/Z╕╦~□z)É~└╢]□Ñ[╛▌
à~║└r/-
Uε9gF╥╪▒╙┤l□K╓hµ╟£ u■≈Y₧¬°x□□½80▀âεLmBhB₧EW╪φ□K╚|°U░└□
6∞┐┤Φk≈╘æI□□«¡½Mzg
║+íj╨⌠":·╟¼ΓQdWu]╛{UZ╒₧}P□<B╝¼─□∩!+g>╡k╫└MâHç.Ñ╙R,çtΦü╩└├╧@□▀MpHôû>,
└ƒÆ□í7F╟MòεµΦOûo
V□Å▀T·@u√╪`«T¥+¡ƒΩÜèò─åY╘X«
□╒4jj╤□╒╦⌡h%=3í

}8X¡á┴╖□áZA├╧ò¬òe7P╠Ñ╞Ω]╕é├·=½╚½/σ□⌡|□▒□|□[╪╙$□╝ÜCrαs□9\╡Ä╝B□(zyWB▓Pu╜□lA
NnHè

ú┘

I±□╧¡εΣ~Víöw°%/ô□ÉÉVΩτ½R¬Ba╘□í╟╪H▄êeF0□□3ó5fú;éû□{_Gnr╟V-îG□çå╗D╫□╗å

²BC□8&╟□╢j□₧]w`α≤]
CyÖìⁿ^MbΩï□□τb█╫²'≡%N¡Tñφ┐B ▌╝U⌐ù/▓¢7╖σéA

├φ╞ε\n≡□÷K▄║½
□╥?V□<

□y├°«╥u╒⌐▒╢╚`u¼╘=Ñühô·□`U£□□╕ó¬%╥ç╟nÇ□FúL]W¡áq╞□½□═H«σ\÷n□■▐·è■╪:≤P□≤8■Dµ
ü□KjîZ≈□!w∞╣▄zU<□┐╥²□Σä}─├X.N 7□┐/□!:≈φπ╟╢w□H╥J□?â4-╟«Θb⌐ú╘▀/e¬╛±
é»J%=v┘╖+Ωpíá¼û□Å□□□¡⌡2ûS╖≡□₧Jc□_ªZAcH|╡éGq⌐□TåI/╧□Y□5Z╖i□9∩□Σ□√/sô
Äÿêì□xåó⌠□┌`G^╒

┘□-1á╕»15ⁿ

òßZ-
?∩.I1ƒâü'ΘX!É╩Iπ{ï~*╗å#U□┐╓∩·R╝≤─Kj∞╞8_Å▌°├éÖ╒╟&ùfH│é□²╧!ù}Ü°.ûOCC₧□Σ}>ô╪
▒├T1#
╒3δ·≡]ê┼ZL□L-°ô°□æO'>\¬öûû≥7T╖Ä╧öJ╒₧▒╗╝µD5_□M┘°εE╓
3ì╒`ä░åëZP<GÜ╕▄σ«ú¿:≥╓□¬□4v□\Φ≡Φ·D╡óî╧▄ï-□fZ╤à□2□&k╝6D≈.-
╘╒pⁿεP» 9+□!▓╞÷□□W╥iæ╖<1□ÇL╣╟╢

ÄG╟æ□=bB⌠┴Ω□╗□□Θ□□╘á□P─Å╢□?├⌠aç⌠í╫]°EΦòyunUb┘╢s■▌m)│Y=

/└ç▀┐6
íJ╧.h▀▐O}ï□"fê]@┬□┌a═îúH▌╕üç/▓`□Sπ□à≤ù▐Bíìé□úâ∩»¥E_□ÿY°g╤d▒é⌐Y»╬╥Fu0

è (        8

≥ìó ■æΘ`╝^ª#úC▒ôb'□₧lG╖╪.
≡▓φ-▒"□▀cû6åΩ¥°□┐·uçcw/⌡ï┴Rï╝■σ▐ëδ^ûg╒□▒é╥¼Ω%□∩□╣t╟E□4qB*Dσ╥"<(\L├ Θ·
V╜≡╕ⁿ─Å*╝├▒·⌡□□|É·E□
▀╖■σJ∩ä|▌╤□y╢^NVP╥áägQXPì#DΩ□w<íçûÿΣT{T╘
╡-□5εε⌐╟Ä%'┤╨@g╝æ□àèWπëäÖR?Q≈█,wD|φ≤-
ƒΘy┐╫Ω□ε~cda.█y╙▀,-x⌠·V!öù≤╣÷X0┌╣πÄ⌐╘x□□óX½φ¢o□╧9≈]ù/σ£¢wm·à£r▒w▀2╣o╚K□ë□
ú█r3┌▄Ωù¼┘@$K□╔╨║k□å□╡µc┼k;CC╜□n≈Tδ║▌±╪ⁿ·ΘÅl╔░åPφ7;>
≈ûô╫▐□∞)╜╜|┐@□▄ÖT╥1<Γo□┬æ]Hƒú╛M¼âÜ╧╜8■╒─╪êñ
a6□c(NhJ·ë╣▐r√╨╞Ç.2îv.╧L@[ù:□Ör╜A+hSó|╪v■Θ□,#pⁿ▐ñ╣2â.=]╔LD╨┼E╟□Öó]o
Th□)JäV▒Åc½┘╜]╤╧·Hgâ┼/Å╪÷T-½Σ▀┐╣█n@╛

ΣΦ-□p┤╕g╜£Ü□O╘â╕⌐±
π)â□□wEµ▓0n∞└ú╗[9½├d□∞╝□₧as█<δ÷MφK⌡E-╙w=~²╥╙wìï▒ü╘□VI{fkoΓ└na├¥{□¡⌐æ<ZK-
àZσ_╥ïD;±y,╡
¥áÑ┘µN
⋆j·▀⌐²≡αzδ

p(¼╬íH/çb╛□

≈rΦ;#QD╒≈ü□□▐9ùÖêÄ5□:Éâ@NmRª$

Γ□·S-ε

Äπ░ò╞Θè2j}çp═Jç>⌡ù╥<Ω╖ì*kóìal╩ß±Où▀wσp□▌[»╟iFHHΓ₧K
6╤9┬Aäê/)²┌"│æC□JR≤+ùu£░ß≈╫Vtª P:Q<C.□>┬▒V█4□∞

□╨│╞
Ω□è6K;φ□╨□å/U╙jñ[═2P┌'║╤Ig*╨Y□t╧ª█V+m┤!⌡□I□("└╙J╪Z□·╙Φ╖□□ΩnFü□□┌·[R}≡Wí□
há}MSa ▐:mT░^a⌐>╙Ω□ûù▌ZsGH¼□□╔□¡□≈A■?
í^&╢□{□*²*1A.<╣1╣□mP{M`□m├Äñ□

NqP□╝ □(□□-∩┌îávφt╢▄╖R□╝╘

ì"▄B╢║)□^°⌐á□Ésì□ª╤□g+hêH▀¬Bß┘÷t}áwÆ
ù% ≈4üí┴    ¡·¢zá≤╞┐╕1┐s6∩dHRG□⌡lj├▐╤┬Äuü≡å▒ë°╡╖Ä□Ω+║═╝>τφ[7Ü¿½Ço□
Γ▒%=0╨BÄóW⌠I□□ó]┤ÿφf╤
àå□
ìδc▌Qqαå√ª╔Ä:∞▀▄╖■]#Q1Uh#Å╘Å5(á■KÉù^¼=

÷BⁿDë6Γq£¡7█Ä║□πäV°■ê╢c⌡─

┕>□□y

í.=□ΩyÜå□□φ7:w ö□▌nó=ï°
±±T2X╢#╡PA&Σ□□ï8{Aß□─□°ß5╓,f
x□m╟z₧è╖g╙□╛Φeìüñσ□□╤□eSá╕w\╬╕X□á□û╤G£í\└\╫╟luH[÷-▌Æ6□Mó╒d⌡□

m┴ö╨╘ ╕£ü□Çß╢ rPT⌐/9┼ú⌐₧à3ì/¢₧Jí¿{□■j·D«b□∩≡ΦW~buà╕
«□¥─SX7π
▌ ò□·ó□Lτ╧_ ·-FW^MΦ╔#|≥°□'⌠Σƒ!□DÿαU¼≥Ω□┬½ΦµU,≤h╤≡HÖ

üÉ¼,-Rÿúôe▀X├σà▌\□αræ□pªJ√ku┘7□yV{╢K=²¬KÅ□□□u[╙┼t≈JΘ□╢□╘╟G╓ä2-
□ÜVw□5ùÄ╒□9u┘ !Ω 65║~-┬]?Wo^╙Σ ,c╟Ω2╢┘σs∞╜
┌µ│*OE□□ΩY□A□π┘kEφ_q╧▀╕Ç□y%\Φua

î]▌□WΘ8ªΘ¢√╥å₧□□H≡╧@á]Θÿë{gΓ╢ÖX]@-¼s□Q°ìC
uXMVⁿ»Wd═c□%₧□oAàE□·'''ó8╧rxbh,╙[╬L╣5╘óφ3▄°÷êâûí┌σ□i□x╧8∩⌐Iñx£«LN

π╤╠òσ├╔╓I¡┌╜Æ*▓ûnΓPkETéú┐°¿(Pj╖Äf√ûpå□≥╓H¡#┘╛#
ì□□□K~üÖ·T╣≈┌╤6!│ir<║φûr░┬╖D·VΦ.ùi«°╖n¥⌡Σåô·╤□□TjªΩ·-
─z;q-c▌ó`□╜¿¬αJ╠«╤□□b┌□αPÄÑó□"█JQ□▒n□┐?°*àX╦3f&Z▄╤r□]╙¬jhà⌡-ô*å╝°□┼É½h«QB
□∩êÅσα_[5l□·Σ⌠;¿å╦Ç
ü╕□iå¿□ΣU□ET╗²□åú»É□I+Hë¿□W£□q□ê3áà□)□□╘d╙Ç□╘Ç*<□j6d@□j□□7ü£□□φ¿#Ü□
ü╘Ä≥-φ¿iÑ□┴╒■4iD=·╬YêΘ□êN7┌┌╒2□9□╚S⌠┤Z&>»é╡^&é$ª·O)-□°+û
T<N┬àe·¬ï╟┌W√ù╛r°╨_∞ε[·╗%°▐≤≈▐í²│╨ö y
√gK√Ge≡┐□~⌡□ôδo?}□╛O└≈█╚‾w^▀╫╣δ╬Θë;≈⌠uε╝□A∩╤┌qΩE□╜□hπ¥┬▌¡áx
u□UZ3¬┤f¼╦Aú□-
#6C□i□Æ4□I□≈2L#└ñ□∞X┬a∞ε□ΘΦ╢≤Ç>□ƒ≡ûàY¿╞╤*h□□┼ä╫66U□v╧)ù┼╤uPτS«ñ╤Ñ
□□□
╙\]aV!;à▌δpcD%□N╜╪±■çv&GçïQ
²┘□^+ôÜÜ▐ö╢■ô▐Æ□∩Φ╕ª(□A;o⌠##C╫÷x└/n·┌]πB╕3R□¬Km▌/Ωª▌ç[åRÄ┌╗-
┐y├□{□m╨║½²╖═█□≈▐ªp8·%\IªΣD.tü╕E□i│U╖□Zi□s
□¡U╙d□┴ÿ≡@ê╟èlz"nq╙e╟□íèY╝,°¢╢▄┌4>╤Xi₧ΘR%Φè▄╤╒x□□MO~ë╘│□â╙-
u╕█║·#+956▄▀τ7àó~^G□Ωz) ▓,k░g°z▐~Γr^╜½{4aí

F#kF□76]¬Æ╘CÿöüáΦ4╡╔┬Σ∞Σ╟&ƒñ5m4□°╢╧─\:îè╖l+┌kΓ╢ÜαòbPΘÑë╖h"í°╢╥D
╕êk╜Oâ▀ß□╤Fñ▐≡ENmC□çπ□°╟yÆ╧■ñ╟°å╕Q▄-.êö╥2≤╟¿»σä⌠║B¼ìfÖj½╟y╘áP╙*Sc
□c=┘ƒTDπ□□B□DYñ╞ö┌.≤╟╕Wµ□-
╜^'πFúLö·ⁿ^zeÆ╧w∞╜sªm█å6╔¿C╜0╙àkz[F█╜ëΓ╞¡¢èë╘▄╤╢h⌐?σ`(□╟Ω╟▌σ\K1σH□τ╡n.&Çy
C□Rë╙mÅ□mP□⌡╧^kñ;□∩L□├Θík□╗÷ö[y½Cα-ÆÇ:TIn╔□i≤%║Ær╕ep
í`ô>@"-"~½⌠═Φ□»□√ê□□µ├─□xφt4e;z□2$·-n╤üß}├6ï┼6╝O7}□1}┤□¼▐<╓╖c ╪Σ□s□τv╧-
╟Q┘╡∞▄║ÄτΓ√'╢╜>6}ÅÑΩ.▌Å4rVæ¿┌Γ□□9□√p Φà
V┼
hφSΩD^E
.ö≥┘╬úÑ¢â╒èrú╡IêÖ9aN₧âÿ┴≈┌▀±\□▐ml█δ□x?╗ÑZqù╪√▒ó╧¬≥xyòHZT⌠□m╔▐¬╡
ΣJ|9«ê_ME╤□«i·□⌐c°`□G□□G-
V╘δ≤#ε∞·Trñ═□±□Éì┴å╗&┤H╛2ëd6▐4ΣN[%g█Ä╗╢╟▌╢Ñσg¿sh=xí:°EIΣ8K▌=¡¡□╔ÅÑè□>9░□u
⌠_Ö╢·≈lêδ⌡«R|²íM╥□TÜ

jTK +≥Çε+D?q?ûƒ)BîdTÖÉQeEFò□□ue╧¿r5â□SNSª□)·MUg)■

gT/"┴┘í╓ë\╝Ç
7ß╨╒□<╫YtÜ¬□gë╔k├□iÅp▒░¼;▀eßç5░F-0□r*δ□█[⌠▀«α∞úusατ(□
í▌3εî·∞□ÜÑùç□□X_┴ëÅañ√□²!b/q□»▀s├├φ{;╨Σ▌3╛x;╤-å?ªφ3{K;wΩ;Γ3╒φÑ-□Y3ûª[°|%⌐
¬W□d□πAí□□Ö
jdτóÜ}ÅC:□uêφ3╒╩÷Æ2è⌐ó

úù¬□²x}uFí□4□Æi╥â0¬;vì╕ΩòÏ¼Ω@ª·#Ñ□σ≡□¬T└!╕t□Ä░<°┫╛]_«ji
X╫╞□u«Y╚ÇC*Vnì▓□m□.$«ë$╘¢

bà·□¥#πα╤□A0dî$^{n}$
┤a□>■□ñΦa%ôα╔▄░Ç,╤t É╣ )╓S]ΘßÆÉ«

tòÉ╖ß⌐╪4½ö8]ä▓□ΣpZ(≥ÿaîÿα⌐]Θjeá╪Uè┘qU□>W5ßl\£?ñφÑ□⌡P■r@¬ù.╓rW
I»τÅ'▀═╝T*■╛εçpΘ°□□.f□ù'╙3█q

¡i╜╔□□ê«⌠□bª4\□□ÉKm%▓┤▌£«vò¼êQc╙;4

Äh·┬╝□¬╝Ç┬╨ì□B□tne□ó$öHÄ*um7#@B0ZU02;û▒?"i□╒(¡□¢ö.½RZ
ó═╞{ΓjrB≈}â¿Dë
ü┌░□α$┼X□╔╒A

■íεL┬uäV≤╧╗╟□Qτδⁿ!┴lTí«Aåh□M&╙ZΦ□á▐□╖viuy├∞å≥µ╧ëτ1ª«?2é0╡≤Ä□□

¡┐y╝αA(ï╟∩îoZ▀□ùñx╫·M4qπ╬ú□Å-╝╤X╜oⁿÄ╥æ╥H┬││z#¬□╫=╣}□Ñ╕₧□£nTE)°kW"□□sH")δ╗
&▄\╟ñ□□|ƒ▒Zi

}π╬jσ╞╥÷qî╩φ╙âhn│□⌡□JUpZ
7□╘□ÆWSW╡:δ\5Ci╨▀êL3╗u4½g□8F□┤4*│¼!%Ω▄¬┴&#å□½PçA□╝#mÇ□P┤:6□6╜sm╫□L{
ò╝¼%-,VqHZòê·=X"₧V$"M╓%ó~□r■~┼╟·dph□
┬2φ√=√ττ≈¢)∩

╨□ZƒG>╕'c ▌═h▌ qε¥.M
ò≥ÑtZεmδ%{g
o5V╥!□αPUEU□□ö╡-INLCÿx≡╬F{±PüJs,óWΦàΓá76K─╝ ╒J¼Σ╨a)α¿kéM□╨╙8y≈╪╕ □ü

÷iQ,□╫É╕M□ô#~'╬.B▌<s□▓i□□*╗F□╔òÉ╕╢D╫ªá@<-GU└╘╫□▐¼`í□p
dò%ÉUû@□╥□╪∩ÿ□░â□ⁿ■¼b▀□UM.¿jr≡²-
l□ú?₧┬¢°⌐&rP╡√é╕□╩û)'8┌]Ä₧□t│□XiI¿Üd/h□Ä▐"½^`ÄΓJαf□≡è^Öì·▀□aúεf╘ê·□c⌡;£~Q
?²□v[╒CA╬\⌐mΦΦ□╞-Dü ╢ß═║uδ╠α□ε┐₧

7b< wv╫Hl√V≥µf─
wÿ°ÄB(╢□ô┌□⌠╥[E'r■□
Φ5□□□σÅ□ÉTh8╘w{╙%î▀¡ì==.²║╪â6□□A\□□$i□N┬□δ┬ □□!⌠g!□ó! πú2ê╧
a□╖ä@□□»▓óú□Æí╜□B}δY¿dçP╒1·ä≡□Bπ≤≡┬P▓□Γ<en¬┘╒=ì÷ù£╟^»┤≥-
u│WwPtsD]φ╙▐3D□□4╛□□o╘□CΘⁿôå∞ª□gª▒┘Æ&╥_∩q□ ⌐úÇñ╚┌E▄
*Ét¢u╡τu4┌⌡╟ΘÅ╪X]MG²ü4┌B^g@d¿/ΦX#╧ⁿ±oQ≈'¥┴lñ╢±VûétO┬□÷m□╧ô?C□ìH□ç≡╥uΘ?Θ╗!
^6Ç╝éù±K▀(«â@ΦEi2⌐^╨â▐cY□□ü╕

ΓA□□Ç╕ $|  ⌐□) □⌠□Çü~0É□â¡@É□`ZP□╘Φ╝häΣ/╙p□┴ó−F∩╕U╛□□╢

ù±y□∞□aV8$|L╨
E½T□:╧▒r °[A+·«□y6□¢T·@δ¡¡Σ□x╘9à⌡╟□□╞τ/□□□!╞□╟47□P╢□P■aö□²├eï□□╨¡t╝
rƒ"╛╤╞V@ß¢XßMΓ¡▌¡$ IU°▄□b∞E╘F!╜
▌ Ωƒ;τò┬#}□u⌡NUJ[»U░¿·ô╛[G╫▐ñL╬d
╪Γµ⌐»ôΣπö╓ô□□□≡Sφ≈P╢A²╘□åèτ╦$·┐H╓□y.h5É BéùH╓□≥╕ⁿ□╙î▌╥─3·
╦╛╜╘─║┼╬░□D:céHgYêtö╕□E▀█«·'╥`ä□Éé£9 ) G|QíÇ<äéê·□ ╓ûE2m
\É□╬ó╛P.αTÑùT?$□□qJ
è+ík□ ╨□□▌□αdΣ║Gxµ╕|[¬□ßD□Yl╕τò□!rì▌ □π(╝□1┼iO_╓□¼▐┌ÆZ╡5ÿª1╪ê┴û□□"

N≈├□╤qÄ░╧□□
\╡7
└ûÉ²□╗Qw±{:ú□⌠·cVÆ¡²╛╒lπi□u╤┌W√,jαG≤638□╛d╢Ötö▌╚╘NüY=┌¡î│[j;æ\½□°n├□Wu≈□/
äD□ÆI^É≥□□

u╣@▄▄m&□,≡ 7]┐□╕{□X▌
Xv□meπññûÿTCL□(T╥è8Ab□5^╥₧ñ□Ä□(Bö□ë-[□uáφl4╨|)%Rvå∞°É>▀εæER□□+P╡□0□╤@
lgi□¿╖⌠bX÷EE}φî ╥╝▌

·tV#╡├ß2╙ö┴bz;K╛dπh┤&"Å·(·M▓H{ë

╤Oⁿ□╓h□G yp═i□σ╙₧□╫□²û╪ Y÷:Ä╓Y╨û-
f╓l╓Φ=æ\∞y╪xäZR√Yá«□U□≈j╒&M┼Σ╪ë□╝8δ8V!▓B÷╫Yè°α⌡I∩ëJr╤╪≤p□Åí╢╡P╗z5{┐ç╫∞Φ╒t
=i□zæEo Σë═≈╖Nv□ôôòæ-ª`G<6ÿ
□LV≤└▌uú≤}₧O╟%□Γ╓÷╓╓Böⁿ7₧τLm▒ö╘Zh╓n╙H□oï╧duê□ƒ═-p·╗°sτ%YJ$ó
H□□V.┌M┤□sΩ■□▒≤αôEï╤q╥-
■îeæz┤5·9µêv∩W╝ÖBQ≥;NVⁿû≡g*û┼VΩ╤J+ôⁿ\□₧¿▌F□∩(╤ÿù╞y¬W&ç⌐Çtæ:&ⁿ╛üO|2=yπÉ=¥î
;9=àHÿ1&
í±⌐╓ë⌠p£c□hàuÜ¼&ú+⌠╙â│KôQ='èF│╒╠╛¡F]╙╣{╧εδⁿ□VD5i%8½ÅΦEHδ]Jù≈'Yw╫3`;Tw3α■ó
□□╕Y*·ä┤╪■Y^â±>ÑùÑéh□>IJ>Qæ□·÷╧Vx-
Z√□═■□W╫¼□ó≥#εÉ(Y⌠╣=âδ»δ≤╙├╗□·╣$c±╪φ-A□_r<□à□□hÅG╤Y≥▀y□JÄ□╬σs│□□[ÜM╟π
K□t□ö¢tms6+wÄDóc]ít□ó·q8τâÉΩcDû╕
τTdíµ|∩i»(zπτ┴╢óô≡┌Ä¢═l÷ÿîZü╕R□╓ï∞╫æ·▌

ïìM┼□)α┼á┘v╝□»╤e!▒δÇùé╫╔⌐ç*≥óï=Qq□il╔Ç¿\╗├AúoêΣXF□═«!ΣAÅ¡v╟ÜZƒÅ□┌CFú┴□Nτ{
Σ□'□□7iÄAe·^▌å╤Hg╘FΩ□Å;▒«EΓ,╝═πs¢yû~Φ─╪ΓLKrlg╖86ΘLv□Éå□%┐□₧╔√ênΓ□∞½┤Z
│t-l/è-±░ßæ║âßô╥╔ûC╛%≤!lπVò─≈▀┤_P□ªφmåG*m□[┬'+-
□ⁿU╬─╞¬6$╪≡ƒI½╒üt7sí┴╙ñNOΘ╙Nd□▄╟¢M▄¡z┐╫εÇ╚₧ß ù╬8≤□φ╬╝âÑI·ƒ═V#iΓ╜-■^ù╧∩¬□
Ωu□ α]~ƒ½╖g«╤c`
&;A□-αMrΣ┘Abè╕Ä°ÖΓ⌡▀□Fë$a□sD□¬H¢╧σ╙≡'µ]w-l~Æ!ªƒ□█□/▒□\SL╞t╟√

%7□/:6:HGΘÿ%╥P▌pΘпy╡x¼{Q ▌□┤║□+╩@F-≈

\I╛à(ñτ½ï□ì@□«·╥|╡Om□≈┬+¿7ÿ╥,∞°╕od1▀°;^ü7pl:^!□□╝□╧ùÄU≡]^├wΘ.-½á√@ÆÆüë╥▄╚
àòM╡□
╝UZxv>▌╫Φ%rσ□αε╦RÇß/sy□░^»~"≈Ö╪I½94tMW░╫j4┼σç│S¥╛H·╨dΘåß@k┬'G<Æ;<┤¡├ùs£σ╕
»≈≈xS^S□°/φ5e╗r≈F\ôúΘ■êE≈c╗dK╗▓ÑvÅë7:□½ï╘ôÄxo89╥ΘùΓ]rr8`╩y"□N⌐/¥+ux⌡┤δ
m=ó?aoδ□ⁿ╤┌■@Ç╘y□RD╢╕d╝ï□·□≥v╜¡.ùO%¡□â>éâΣl!|b╥i>ò^

p.╤K⌡─╥>mSqxF┌│¬╥8°₧M¬
┌Bδ*~σlR≥vF≡┌m─üεΘìöR┤á╚*╝≥Ng▀╫╓┐⌐]" ╝A¡╜Ñ±\ªvó■Ö≥╤¿ç2│⌐=╫Æî

mεâÆj□ΣSÆ■□╬$-*╘+ü-
┴╔□pè¿9éτ┤[X└≤z╡┘τ□%xz╬V▄Eⁿ□£ÄτyD□╢╥₧╝Gô╣÷ hï╟Q□bç╟B¢┬m┘P8█□j>3Θ╥□⌠$
_╫┤□□⌐û`Ç /²□╝ x°îa(M'U;□ⁿΦ╔□Oáe─ ¡C╧┘╕□┴■(}
Æƒ°9/(□□o=Å┤°J╝Φ_áφÅVhL╫Ωπ6□î▌Jⁿ⌐{⌡□□ä5SΩû▓□|>╪ág-

1e,^Ü

╬σBæl.D>╫6╙□╨3

o□|Nèª2}dτe┃Q¿i□ñªNΓZ4ùaÄ╚â□╨-□□α□£╪□¿+□┴y┤╙□╧$[≡□□δtÑÆò▓╫□╬□╒₧í╓5kd□P½ÉS
O3╫î▒I□╣ç▒·$╔'▓⌠□V╥╘i¥╤Ω┴Θ□¼·ï▀ß

□Γ╩╘■y□┬□┐¼7Ω)4câ▌·Dφ
½EÖ1±[8cMåε╢╙Fß□₧U=C≈Ç2âU⌐Φ╖+ƒ½·□═{½£J²□«╗c─A╠⌐▌□╕╪>Ö□□□í°ê1╕≥┌ìµS├ï≥⌐╛┼┴
εT√Bj╔⌐ü«Ü°ò√iⁿÅ□∞□å|;╝╪'ƒ¬,┐`E&╓□╕xσgDQJ□É╘¿Mⁿ%£□ªª╬.y$⌐╖xl

»àì∩□»o░╣;□q╢∩╠ù╖║╜□╡£½□&o_¢╒W~&]<ⁿ╖┘#█ô≤┼▌\t▌\»JëG!┤Z╒¥□óbS«Ö
ƒ·ë─b╘)/╘□ñdF!⌐åAcNÿƒ¿h╬╨ΣA]□□═┘#ëv□!╘□°┌▌□□O╓üαÄä▄φ╗;□µ≥╦ñY□MΘ°eS┬
ôüÆl□╚╣-â|▒A¡K▌í╦

εG╗V@─ô5║▒,F>CY▒Cù┼²□TÜΘ╚g*⌠æ½L7Ω&K═-.═.ö├ë⌐¢g&

ûc□Zbδ▓-δÆv⌠>│òzsda.ôÿ:0>rhSkj≥@99▌□≡uÄ╖╢îu·wó°¡Ç╖╙╧├°ì□=─⌡è°╖═ê└∩
□Pτ╖□Ä╓'┬ï╞╢\PG{□ä¢;N)ÅÄ□â╫w¼└xÉ┬¡OT┤°╥□°Ωô┴gkg┤25¿ü
F═Xs(ëAΣτCà¥δ<¡-Ig¥□i│$ä=□{□ï∩δ⌡ⁿ╣)╪-ìò2╓▒d┤#(PoÄ/╬ªY¢▀^{¢F{□Φí-UE∞
Qöo╦═▐4□□φÆ╙¥_╧dâ¥#□φü∩c╗p

gjç=ä□) ╒╝╟x!▒□╢8□
ÄÑfV╧o.(]OM πàJ≤√½╙σQ□N┘"□|Ü7┤üâ□▄□⌡ I╥[«ûx─f

I

╘╟□ ║╠

¡°9W╥_√≥rè□□&¥□¥AoF=╧╫ô▀□U8ïé╥□∩½D-
╪rFnò[y≈y░╡Φ'°ûc»σ□¥'≤▌□╣√Φ╪ó±╪7─∩ëñ(=D╤ε╝0┐|δàb,▀r¼óló□δ~¿é»□ì╟p□
ö□"-=ñΓXi□ê7`ÿ_=kª□Ü╡║σqg╒εâ□⌐F
╫v╔□┘ »°hFg⌠'╗cÖíûír!%≈mj□t$<□
┐í⌡R4□lç╩≈Día║5╝>πΓ,□▐Θ0┘xZ░Z┬
_╚ΘL□╗□âiëσMF°ì╚╙&┴ö≥□".)åw□î@x=N?F┤+V┴SD$ÿ@X□l□.x(q╖=¥┤□J?╩(\z□□5_
°=7_─V«#x╘û8Yq█è6εd┼vêI?¬□╣8□í▐i □8+⌡ e6□2w╤1≡╕▐(□Bû▌[f8Äπ°⌡¬⌡≈□ⁿ─=
╖xΓz¥₧&)Ar┴e_╖c'ê#² ú┤ü╓Θα╦G▒u≡□≥φ□□g┼4²M≥v┌

╡□e°:6óÿ╣~$O┼□KÑ□£
≥□
#╥╤ΓW┘□@=üùƒ╨ÿ╖ì□]┐û+^Zµë'owG¼N□▌╢»c`S^╥Cì╤ε□⌠=}íR¬.l□µl;□₧`J»░¼╛÷¡±r.□⋆⌡
╧pNA≥╗╜╛▒□ u □k□¬~≈¥╢à

!╕╢╛»╚1r(d≥,ÿûê□eæ□ε£╟à∞╓╞■
ì∩ò%BmΓ□╒Å║∩□┌µ╥]ƒd╨ε╡□(▌╙ö╤−÷·"

# ⌡

M┃é╧. ·¼zΩ!Æ║Å4□^┏

5
▌b¬Ö
¿■╪└□└□≡"oáI<¢]6□xî1Φ)8ÅV≥╖╘?┴yî□┬Öεr(/σr÷

£Læ
┘¡□v□│n¥╝Ç<R"c∩^╚¡│S ▌Σéw⌐>3e{½╞V

x√å ·^

⌡²K-+»╙εs╡r╥ë╡τ»·ô║□Æ$CΘ╛H□l!Å7Σ`╔âÇ▄G▒v⌠╔nñ■JG1ó╟ε⌠ë

·aÆ╝□0é█ßp¢⌡╘╟I≥00ê□ÿ8ïY□ª}<_√l□hfük□ìτ┴c□□□⌠╡yN²ä╕7
⌡à¡□å9bIY_├α4ß"óp╜b│.°C89■<°│╙□ùπîτ┴9h□╓□.╡□ßh é╪P#0□┐⌐!Æå╒èuZφ·□□.
É╘%,íεⁿ┌┘cK─U░9UÑûa╚ì4□dÅ+`3É·(┼B▌╓#
·»ⁿw=░╙-╖_4PGû(â┼δ≡□-ñ■
Σ□,╧ΦH╚╬■.

□s8ç□≡5â –╘3⌠╫ °

yy□□√□─
╨┌┐□B ñx▓É&{÷)â┴Φ<□ε?□ÆdV▓ƒ□□□yúΣ[p░û□÷0uï¬:-
▀╓Dæ≥F═Y«åâV▌□¡°çj·°5;W╠PΘ□°▀□t╙»0└□≡╕áµ«;N~é╘ï~ù+`□4i1q:â╓xèö,v^G2<W╗Ö□□
2F╙R£M@·□A¥├□ä□┴□╓·╬Çï°⌡,àzÅ╛zQQ╦O│T□≈□⌡╝z▒Θ3Uî"░⌐^áW{\wQ-
'¬¥BcδdOI▀│|∞[±╪{Wî╝wì▒'[√z[╥}╝Θ┌□:╓ôN⌡⌠┬▒/\║T▀íÄ╘□ äπσÅ╓Äδ6R_#ªê├Xµ:ZQX<
dDòxEs{;□j7Üz╦±▓w6ç▓╩M╙#┌╝ÿz-
□╥≈δ¢╢5.0í+NWL╙╠╚≥Σ□MIÅNqÇá╫□σp\nm¡É╧óªë»n#cv╙!√û╝B·╙c²≤AÆ╥üò%2╫╧ú╢¥j!¥┴□
t .□╥&4zªé2□Uô≥║-e9!┴òN·Z3□╗□W²÷í█║ió¬oN⌡à□□\┐x'─□0^·□xà▌
╒╬□□├□st╠;-á□N?A-

╧╥▒"■îîⁿƒ<»UÄ⌐xú<m╣║

_
Jç├geD`pD|▐ê├`f▌╓`0σbYW*□L║Yps²a⌐°y+Oδíα■C_(φσ8o:□╩╕9╬¥AöZ╜T□Åδvß'∞UΣÄDε%
d┬A÷¥σä□°╝$α├□□ΩZ²Yt░ΦE╗-y╨±e□qτZ□}□Z⌠

╔+Φü¿╖E}▐░ìaY)Ω≈┼¥,δî√ⁿQë□]¿╖8□_╚K╝`ñi╬┬ Q÷'\□τJ°²I╖╤ΦNB¬l⌐╝□ûê╫□/ß╟½&τ⌠□
┬
¿██)«□ åδåτó□bg╘MX{lìGYé┌Ñx?m▓╣mó╙□t≈p«¿╟□urƒ□vf3ετ□#úá□░}▄+#½Xåpzµ╥¢αAΩ□
«ⁿhWv▒▓ƒ'Å₧3□"ε)┌□╣·bß"N²@ B╟è□╞┌≡≡*6Å╕□>□"

╨Iä┴ñî0╕≥3%╤¡□{¡r8â▐3o'C╨□êN□╧{2□2'ßS-äÉßêö≥î·≤ù╛qû□╙P:L□Φ□╙ ê└─□⌐)ⁿpΩvë
╤p074ÿE┐□╞s┘
≡□Iü
α4Ö%╫□p╝Q║±3\UGα□Äæñ╤q╒□┌█Ñß□WF├█cf¡bm°□■□□e0▒4°}"□î╟□z╤□Ñ═□r□Ä{æd¿├P┌|□▐
Θ₧┌ù└oΘ□êê□πóπ □≈%≡mTτ╛sf4#i┤═î╢╤╛┌ûlKûeI╢σE≥╛'vlgq∞∞½ú`B┬□ûä@BKK/ö¡t{ÑÑà
û-
$N└@{[□□^í⌠](□.Å■Z┌{o√zK{{[nJ□╦≈£3#[v£□∩÷²-□Åuµ╟0:s╬≈ □╦·>□╣:)□ZOq¿ä=e÷hÄ
□□□ñ%□p?ë>ï□□S┼@□R░╬□*èR8[é□]α□□'6«°ü▐e□∞F-
òZUτ⌠╘»¬□É÷X¼NÄñ╖ⁿ(┐ε╞[·▒W┤╝ååÉN∩xφφƒφ█≈ε?╜╛S┴0□├rh,«àO°k°ä>óMÆ╜é□□!╙æ┬Φx
□=⌐Çè─ W,agÑ'«ê╦Åî□ΣGF3┤α1M
╔□9Å╣□□ⁿ┌Y7öó┤F╗`wΘ□=╛a├□□╔9¡f'»"w^I┌÷²∞φ╫v╨*åñ5╝÷e≡╨[g└C?Rs,|ZF±j~
%|▐ús□≡óΓ□□╤ÿd□Σ└²□C╪ßoêR$u□

(üé ▐▀xµ=\╜∩□ⁿ,V□Å°i┘±|«┐╖┐Gísïé═¿í*[#□1┌Z

òC╗□Γ°B±w□Σ┐}ⁿD■æo▒<K#¼-
y⌠°⌠°─┌'-□üÜ□E│(ù╟╡≡Θ₧┴O□ùF╙□n8i6□╧Æ□!□4ⁿ`░│°±α├q╥≤┴□▒Ω#□╧Müg¼ò¡òñFÇ»█¡□è
▐₧ε~□epY!▀Sæσ¡Q□╝7>}ⁿQ°0p°α3~□¼>q□¼}@¡gi□□└╚#O£és⌠╪£□╬╚▀É

uêÿÇƒ»àƒ□ì?&&á\°,╕│âⁿ□}e▒äwövq]P╥╖èE□φ╙Γ╧H╥╖□ $ß
÷Å%-□3y3├Y□A40V╓Σ│è>ô□Σ?╡¿-VJ▌R□±α□□■╨W/nπ0□┘8≈╛"⌐¿Y\q┤□W□φ─□G-'
πP▀░£á7^Xq╘r*g□G¢╞,'s≡ⁿ╟«8Ül╝ε┘C7=uU-:-Ö╣¬÷d░ Üßü²+C┴■½ç□

¼

æ╞╜/▌7╢Ω«ù«╟íπ¥/□^≤à╦│ì{ε\│µ▐}≡x□╥iτ╬æî"□╫[#ûhV═

iƒ&x¡f□┤N;╫Cp═df_┼uaæd╙f¥Φ╠╒□>àö╝y(É¿âÆa$╖Imël-
$□╓∞╡Z╝f═9□¡êètüràûç3█#(mj─πò□ûZw╖□═w▐╞kÖ∩ô4
□R!Ö░□«ñ[ß°6□□ñYl!w?YS□□ê·□≥Φ)ì╫[∩ÿ□⌡Yu□oíÿΦ8W?□╥'ÿ
□φ1A~&╛-Mq╣^<.'Ω¡ÅΓK4╣┬5

‖Φd

^%ó]"╚g□/+|╖B=D⌠¡ûö╬bΣh?êà╕XΓ¡4kP╧╓δ,zòBm╨□s╫XìQ¼ε½i▌╓□╙0□╚╚i□▀░µè╓╤úδ½∞φ
√╫■□Y¡2░t╖α□╘J▌─Ü╜6ú·╖ì¢□;|e┘¿▌[µeá□╒[8□□⌠ïe²S¥ë-
╖»Ωⁿ╛Z╩□?ö╧S□ß°î□╖KπSC÷duâúí┴╓╨α`¿òB╒4w?EΦìM╞&▒n□êY╢o4:τ≈╙}πΓ

░¥á7K[ú¬▐»τñ
9╪á#àXB5□≤□|u╛\ù□⌡Gτr°□¿Üª
N▌═≥>¿¬□y'╘┼≥8αÆú≤Q
àûσ╞╙∞ª¿â
W<<╒▒□M¥□¬7H∩╫$VMu┤nm≈W

∞┐VkP+hòAsyδε₧2{r0┘░Ñ7╬2j¿ (Tµå╤╝Ö▒OÅE╝═δ╙Ö╥å½

}vWú┼π╤Φ╠nï╤f`|Ñ^□≤ΩÜ╘┌î_╔┘═F□╟□2δRß₧ö'□

╨£├b░≡zSI└□□╣▓│i≈¬:-
I╟W]□W╜□ÄU─□ÑD9□òj█₧î ·╕□Æ9UªP□æ□Æ~╨R┴·╓G□e3└zÜ¢`7)&□9σ□J▌□°#ó3hU=%uΣP╧'sr
WQ╬i'°ma□üy~₧£ƒìà¥≈<"□□αS─ì┬ÅQßK╤gT╬╫h□□âÆ¡é┐╨&_─□¿vδ□l░Σwæ·□x│╥_r□╥0ÜåÜ⌠
□8┴¼&Å╙╔Q-j□X¼2¿╧▀□áJ╫τÑ¥a□«K▄LΣñ□V
▐:□èçΓZ╟

┘uÆ╨zg□1¥N╙⌐□╨p▓r╠▓0⌐µk□/TfMú▐°s░{%Ω□*W9F[è'Q┴ _▓ⁿ-½·(ùàé╢¿v⌐ñmRô□7<╣│~╟P
╥──╥hôTIδ÷▐╠₧ò□□▀5½=UeAúS⌠╕H╖Z»íM╞|ú»;8⌡ì▌5°≈<0Ug0ïª2-
gπ╘ó╙Ωm▀▌ôÖhvS┤┬^Br^»╧Φ4□C·{□Tj≤°Ñ8Bû:□G┼C┤Kú┬æ╗ª□╞╧╧É╜╙äòµg@π┤}L│I.┐:»:ƒ
ñφΦ▄⌐□>╣Pü╡□□ëπ╗ñw,}AΩ□E½Ö|5ú□□

_)□□≡¢┘{╘□5m□╙?Φ═□å:#╕∞v²__E
BëB╖zYúS(+a□'╧▄▓uε}Ωu┌□□*It□{$M$J┌□□□"mY╓m□╕M≡çM=K╢CΦ╧ÆφY-
[╙@W╟┌╓╡═╧└╡¿¬WQUK□∞h½ÿ═┘╓╤m│≤(╡─Eîµ½─qµ□8\▀╪╟é@═ÜJ□ß╖-
Σ╒JQ»7]±αΣ╓»φk□□∞φl£╙·¬╖□q╟û█'ó╛û

]ù≈à▐┘?╢g┐ú~M╙÷╤*□□;;2¢2₧O□;|
Φ╝y,□-║fe╙Ä╒}~O╠αx¬φΩ▒Ü¬í╝═⌐ì#▌₧@∩ΦFr≤╙µ-
úemM⌡εÜ□g┐-φ╦6√╝M¡=ò¢''Q▄□□»ùûbz□╞⌠

î⊖V ╙¡╥b║╡Ç⊖╓Oä⊖╘K╔╣╟

□·╬╢pl∩cçnⁿ╬÷≡□b╙εí ▌]-.▒□▌ ñÉô1}∩ï□╙ τí╡_ÿ╧ñ'∩Z+ß╝|□╛≡»)┬P.
I≤2L┌│□ ╞kα□!□à└·0£□ª□B■∞UdCÉ7╒ qd□▓░
â·,□ó²g».□├□▀2pÅ≈├P_cX¥rv\⌐╒0

□(@┐□√□Q□╜□≤[¿X╙φhG!□╛F┴╫½╓□├$└╨ï╝å·Aü□£┐^═█╤èâz

⌡□°V╥Æ╫      1ü=OV□α□æ£!□₧╥X¬□8ⁿ⌡╙σπ\▓ê□╙□╖ê□└Äσ¿τΘ□∞╧$□A□qeô┼╕_zA┼╟□╪ □
╫πlRo6()╓á□b∩X5╖yk╙╓╛╕Ä╓¿i╓Æ□█ƒYwl]Ñ¡²└╪√d□ó╓R╚╧l-∞

îyC>□∩4┌}û`└□Ω╥┤╓nƒöß-□;!┌▀ ╟`m□φ½╓▌¼n┼pΘèlΘè□ÑYJ□e≥$D√□╛ü╖ñ0┌≈

W╬y╜t╧╕σcú=╛║'%í²░╖r.ço░XP_

φS ù`□g≤üç≈┤∞[¢6¿□J»S'ç°┌[╖╡√+å□

\□╟K╔h⌠Ω}□Ω□C╔⌠µ■8ï"V)Z⌐OÅ∞o□√⌠z□⌡c
mSâæúk∩╪Ykv╗
zô╦□txJ=■µ╤Ü╘║□á≈g╫┌å╗S-?□zâ├┬[□¡>□t,□╝21┤□±{/ö≤┐öæ~├□╥[eñ□-
@ñg}δ╦□Q┤≥□□┐í─»Y□Φq?

u<¥ôzB±_≤ëq₧·ÑI╕Se⌠ß╨d│?åΩ)â÷
â≈□&o─πïy

wrûⁿ² ▀□■a ╬╗¡&╖]╘Q²J-Ä¢V¥┐u-τ╖C£┐□πⁿ╡ïq-1╙ε╙ÉA□┼╘|áü¿$╥□┬! ½□[B
9ël□ß²)x□□·ô9□□" S9|▌à╜▒ÿo/G□²╟EA ▐╬□OΣ□s#I

╣╝T□%[▐╡╗╗φ≥íh┘╨⌡½¢╓ûb╨oB,█$Σ]ü₧╪╘âS⌡'w}}*-
╪D¥Ä╖≤éâW┘▄6Oδ«▐µì□Åv1Φ╙dr≤gα□màsß8□!╫b╟7C╟▀9Mÿi□a╛╕,µï□╞|±Æÿ?o┌íÄc╚Å╥□k╨
ε/σI□ⁿv÷.ú□□■□\□≡C┴R

≈□▒□ó╫3□φkë. ╟╕°╨╞□-⌠°╨

□dBI╬╚-╔BKó╨Æ(┤╘ ╗
□□jd√K□▓║□╚üM= VΦ□+lΘ)n┴i½b3É[╪L!l[□ß
C≥▀^x64CèY;b-n□Ñd┴┐□□⌐├}Ω╨.□│

┕ß
σFta▌3d□A╠╝1
□╫□6α∞N?ÿ6╓GN>JYá~ÇK╝╡óx}□▌ú5□o┌ZxΦ╓┌C╖╧□▌:C╢ey□E¡│╓&:□ P╟"□$∩CzC┌°Rö;□-
╕ó,((¼¥¿É ü4 ╠╠□┴4⌐πô╥ñé╙√B₧⌠Lπ╛□≈l√-
ùÑC}ùu4ÄC₧┤□≤ñJTR▓k¬»∞mW▌p27□ÖRπ÷\╣┐cg{fcôτ╪╤□o□²#7ÅE╦W]3
3Ñí±T√╒k!S║,S│añ╟ïÖ╥╞≥÷ÿ
qÑ╞zOΓ╨∞7ó}-M>O3µJ{á|▄ g█╗K╣R-µJ╡ÿ+ΘO□v@2ñ?A∩╛É+Θ!W┌ü╕Æ-□â▌ƒ+╝[□□├╜□ΣÆΘ╤-
╛□□-
□umI╖oo≤ùumi@GR<≥ô;·[Ä╛r█æƒ▄▐ƒ=·π╖□|uS(=y∩8<å□&∩Eë~µ╬üw□eäÅH╧█?î╙>l hÖvn╟
e▌g_D
□
≡ôZd■h9ò├g╨*~±"╓ÅB£□xùàΩÿ╒cV²3g╓B□ûU}¿1Z□éΦßò"vb"A╝Θ□KΩ\V▐╚k□_V¬%w&
|╛q╙x*□qóæÿ*0₧═
╢Å¢□╢Å─ô5□&║□□>Æ'ÿ¥Æßú■ΓåÅDV¥ô«@fÅ$Σ>;e│G²≥fÅ┌K¢=,≡!U:╒∞°⌡f□C│éε⌡÷╡5&kñ-
Z3Æ⌐Púφ#P-
τS+6'W▀░*lo9░■!≡«└w≥vA═@⌠5╗mV▌kφSc²>□CÑΦ≡█Q5N¥ë╫qnù╢▓o[}b█■[╫|□φ4X□τ[
f@7□□P▌□

(¢╡□(╨çÉ■ⁿ□▒ù□û□Ñ□xiεô╡╔ΣτCD|~àë╧

┌≥aaz.f=α┐=zJ╥╢/m«▌╨SmP*H¡FS▐:╤ö^╫Φå□hτ□ê"+X¥*ùXô±□╦[½j╔vDP*□4á\-
□╨□W□à▌╔■Hπª╒ (∩╛jUä│┌Ω□□ô╫□□=^╒□*∩ê┌□╜=$╪⌠┤=╔□÷╔åE╗▀NδmFâÖ╙□▄NSámKs|┤-
┬Rtyδ:╕¬Kτ╬S╟□A□⌡1┘╛□≤□f╘▄⌐2Ü&¬f└w▓□KÖ#vV□±q□δ█╬ε┬□OP-
D╠g□φ╔rÉ≤─╤µè║èà╛É·□b▓/={▄$&>╟□├Wò&Å□y□≤òÉh╙HC□╖2&Oà█-
uδ╛□ù□ô □Ö┐□Lé□ƒ ╜é╙δ=ÜsëéS┤Ω╔!ì₧ò6å]¡Σ1Σα∞/□√┘<≈□Jä╪▐*╒XC±P□╬│ƒ┤─~Ωf□ù╒V
6
£□ò█─ó·t□Γâ/αr▐µ□╔ßKhq╤<Æ□□Xû≤ö^hΦ@kÆù≈Hè□√┬┤:╢‖-
óc□X^░e⌡â5¡¢▓₧█n▒ç□─╬j▓█┴∩P□□àZ½╬∩╙Yφ.aⁿÄ¡I░z═▒±*
ob5╝▌╚Y⌡J▐┬{δVF6ìQ4%z└╙Nç□½dFu■wÇ□áb%JïV5wÄ╥/f?-
2√iB∞□jî╔iq¢f╫2∞□¥â∞□¥\Ä²$□│=J□Æ┐Rí╖□1╛2₧□▀Ü=╤≤□G▐ü▄G╘;é╙f╒₧□LâlUZNCφ.
□æ╜

┘: ·µ~Oφå2□▒ƒ>ΓJ╔╔□#Ilδ
ïl□u╧Æf□S¡ñ□Γ}]kS2□bΦè7m;║▐öæ□τ5[╞αa*⌠╢U╝Ö│φá╗▐£╟«┼┘╟.a⌡É░¼□╪ed█¥┌u▀û-
wnëy▓□[ t█"πƒ▌8vtMàÿ□n╚┬╫√╩ªm±▐ÿ╒□[┘░eì╫V;╔┌:Z
Ö·Ωª╢uI3╨┤L÷åJ:65F□╗│-[}╢»▓nkoEIδx]Eokâ╚╝╪║□ⁿ>3`.Ix▄±╨J[d}₧+¡½Ä┘]╡ëä╙[□▓:
+±NáA8Æ+ßH"∩Ks┴{¥éCµ%═Y╡╓qû▀Q≥ù%á₧╒≥Ä│9~□]≥ùe≡\qQ<_Y>~≈d╟e#MÑ|x²▌{v}~,ⁿÿ=
5ö╩

V ÄΣPmf(╨ô┬u/▀▒╥ù┘p≡«╒╫ ΦÄò╝ƒ}·3√∩▀□m╬▌|□-#M╣{α,²□£□ò□╙ú─╧é²Cx╩═≤ä¿1B

₧}2░=╟□ ú$≥m╝ÇE□ro□│□╘#╥µ`-
å `-□ï▄□≤╓ÅÜïZ?*i╡åÖ}å╓ár□□·τ┐Γ=Z□╓░*áó⌡é]□▌□≤}%<█"╪

JÑ┴&□6NI■τMj`≡XyïAC?@)(□|»<┐è¿╣9b;ⁿV °.□Ω╒(□├□êéQêé

⌡Ü□╜½4\¢╣
φ╤ááÑ~□╥δΦYE┼╟ô╫Q(»+■□≥Z╥SsF├□╡╤g□=Fe>úß╨

%5□«g□wÑ□σ╧=ó3τg╙ⁿC`=(≈·^âΩ╝B□□╜F□£ó╓%Z┤Σ□
/      ░┘▌^▓o÷Md□üÆ:R,⌐□áñ.Oö'Éñ«ô$u□KΩh≤»á╢□▌&~≡QÆ:Üû$╡┐·W9|    -
~≡▒%⌡r6i,¬e²4╥░≈K¢□7vûk╨µC□□╒■╞⌡-
Θë╓á╖uG╖°:□@┬ZD┼«╒8ap~▄▐-┌p╟╞□□Y}l}ò▐dRky╖╓`╤)Mó╓û□¬½Ωî┘□□╩Γ□3PXC1╬;□u■≈$
Y1x9B│
p>┤,╢Ngeδt│l¥NN█ùò╒vIV█/)½ï¼╙-≡¥╥·²┤

□k□□╟Ço╬■□□Gτ⌡Σ□t<;/«╤hò▓╕fì

A□╪□1□Ñ╟□£┘□óÜh)╙δr0G□DÉ$▓¼Sτq□≡G□□ûñá≡i$⌐,»è7╓Vò□□¬∞MδÄ╟□Ω#Σuí╖╡∞═£u□ò9ô
úû╙kfYq═\┌F=≡Γ≈╫▌╢)εi₧hN
&□□kn□╖qU┘Σ▐µ
□╧Kc□&╓¢ú}⌐╒½╜Ä║╒
╓■jqrj╧$0▀±□_δ╢╢╪PWôC¼oΘ»l╪┌]^┌>₧\},µll_□~ô□□╚║k¬*¼Ñ[≥µ@}<f╖V┼□ⁿ}Câ□□3╘ε!
£□╜╥hÖ╓*80-▓Ω$┴┌g□{╥ÇΩu│'Φ╓▓l~ß=╓≈b@gí╞5Lú≤PπZ}Qì╦|í╦9sΦ{7\□·¬║ªC▀=|□<₧,_
yáwφ5╜┘≡α■┘5╫⌠·╚#≈ⁿ·╤ì½┐s÷δ≈¥═┘q⌡├g∩╫▐±≥æåü[┐╖O>□|╬┤□Ω\5╥╠⌠æ|Vmσ5Zìs□=èé╖
^┼uäÉ□R½╔ΓF□┴⌡7q5k╒è╬╗5ö╓m□░½□2-Ç╥Θ□<═pd¢ëδ□┌╓·□mδ╞°4|]╜ñpE▒┬5î□«╨
fñXßZjk~J║□⌐]QYφ□╞jW□¬]#ï╘«σ

╧╞ÅßpFûτ║é├·▄┌▌╕╝│v(æ▌╘]┼┬∩LôîÜO»╛¼y├τ&óû«ú{_%½É±╣Wp□╒J╬╗m1╣¡V□`╟∩╝fKE┼@┌∩
/≤⌐xùYoσ⌡\I╨₧□┐╢ú··;-▀wF-╙Y?⌐âpöè,╨ë
⌠»▒"
┤□[á ä⌠¿a
ⁿ(O╤Åkü╓H□Φ?aEl□Yá5≤□≈o¡ï□¼▀≈╚╝û╤╫╘≤*Ü╥Θ5ë┴╤:
╬µâ□
⌠╚╝│ysoìN╓┼î╓5S-δo]p6â╓í╧∞l0╣╜z¥╓m8<K}═□Ñ?╗╢□·Ü²a□▀╫Y□âα
:¬F

t5φ━¬╫Ét|D≥5ƒS(h□╞÷□
╪«+`√_°━╪·

□Z├╞∞U∞ΦEß}Z▓@k▓Ü\Q∩σ□╛(┃╤E=╫□ô≡▓╨ä-
╤¬┘□¥≈8┐L¢|Qwá┌ú□┘hD-τ⌡α!p$Σ╔ □!â□┼ ·□╫e5z£

₧ⁿ□*)Æ╙·ƒ|ΣÖ┘>yûM╥zê±W□┤▒□µ1>>»ìUf╒╤µ?!└-□□O¡ï└<╛F
a■O□µçÉ□z±|·─H╧,Φd╪√£Ö\YÑGAXP¬░í╢φ]ù≡?≤-oëê<╨αü=□L╒sV½FktÜ8□°▓:E□█do≤å&Å□
;í
>»Z└÷Φ{I□ÇΣûOK┘l¿Cp£èp~T╞·$┬y╚g#╙÷!═Φ28Å╬A£G' &^h┼
é╦a╙ ⌡'ZNE1:döV□¥╞╥Rdûå£U÷Bô

⌡Cé(xYí$1□□@â#□mKÆ}ù□Æ}GPóÜ□dyÄÇó4▐('╛
σñ2!├░ID□=KFÉè"Ñ□⌡╩åk»£K▀+'ªé╟▀á╚═
┌ƒNF▓j╓K─ê,Aí⌠╪Y5╝óè]╓Æ□╬Öd└i÷▒□σ-å□░□□⌐tHH[Rä┤(╗
╢□úñ.▄{□┼y╪▒9╕(c╕ú□┼%□┼□ë□EH¼á^¬┌√─æk-
┌Q□╦=qπu≡°ä▐Q╤8□□¥l▓╕[┤w╫ì6àD5·Ö{■≤─µ5▀9{ ▐ g±±╤═_║j┤╓6x█wsƒ□σ╞t░m├□╟êé╟□"
r□h±[□□▌ Φ□A'□8@╨

é6Ç□║XA□┐ □□σ╕ ëßè`Φà─□ü□ƒ□╦Y[├≥Éçσ\`ay╚├r□¥≡

─{╜[D□ë□⌠[├╥V|x─V}^╢Γ□╡ □▌□°┐R├+ετ□Åö╣╟t`Uÿ¢□╧□□#}NNI⌡j┼
□5╕─E┼?╙ª°à▄H□□e/s:□o┴á{,α±rΩ▛▄┘M╜}□+9╖9DC□□□5µ»╖#g╖∞δ╞\Σeⁿ Ntß□¿pâ░
eú╩ó┴╖ó┴╧□
Z
□∞□▒xqÄ"22OZñ7R

yÿ╨HC¿A╣°4¿¬└╟&3╚╔!≤ÖéS□gÖ·□Omû8╒ ║fw╜αL

&Ü6≈Tkæ«H╥*▒a▌₧□ë ┌▄2⌡*Ysij□≥½□╖┘`ß⌠µ`@─╘µ║█Å_ü⌐
╓,▀°εD▄□tI╥t

┤□
⌠□¿V┴í¼Fb°□Åv5□φj(f│,"?+D#□╙ó¼jÑ░K)Jß5╧çT\!â9╛╥ü«t╙╦□ÆÑ╙i£Dè└╒}át╤╥╙^/»$=
z▌F°≥⌠
¿░K□J&╓_╒□≡▓ÉùçΣ█·□ö
°₧┬┘├▐ÿ≈ba□å╨{□⌡
-,┤╤╕□h¿OJ╚□▓╜²□ë□ □ò1=:ò-
Ä□□W¼╗}G¡┘σ╤π░□°╖□r▓╤xφ┌l□'[W□B±□!□□`Ç$╪□gJtΣJÖô)ôâ╙δ□â╕√Sëô□□Ö£Múöq□PV
ée X□J£á╘□□X|ûêá─□J-á╘

JM□é'£$A□□□á┬□░,□$Y□▒ê≡□ïùôδ₧I⌡╫~±□¬çµîF╣Ö╣≤Y□∞┴íe╧í9┼í─î□□A

à≤s╧Æ<QF($I¬Ç□åû╜□ºªâ°□èX╒Ñ╔ó£í¼B╬⌐]□τQmFx\X·K■·█RJΩ°(╕┴$φ$Ü²¡û╙A
åUé╫iú╗╥φ½vsw≥µⁿ╫%Jy╣»4 ∩à─îÇc8╖hD╛$J@¢sh╙+╧┐□ ╧lZèoxƒ║□rÑfΓ╝$S╦jAY□□□í░
L}R□⌐╡▓▄¼E╛a
\V╡╧└l
┴ù□é¡!┤□C·ò±⌐°ß8□wí□ßB/┬à□╖
-n╫3d
A└╕╚¼ß4«╟d£A%FP%@úÿ┬<┤2²g»-
áÇê%⌠uBΓ»□Ç;#»╬□&▐É□¬⌠□╨{ÿÅ¡¿╟≥9□·╧9┬□╫)╛█□┤□□&°(f√╤□□)├'ë░□≈∩·╞TÜ╖ëz-
o□x□bß▓{█w╬çX,PZ9─□╟X£úÄcF√Æ

µíLeyΦτ9ë□ⁿF□zÿ^≡rΩJxⁿ
₧ß□pjLnªp□╟I⌐□±U£|□>¡A┘7»Σ╨rdΣ─¢╛┬,≡üóPé╖q□üYf!E□□±=ß±□°ß5╪□▓êlK4□┐▒□9Sf!
aªlg√H■-σ ║TH□═Ω╒RP╚□ 6¿e■□é□çQ-≤o9.□≥∩□Φs7·□·

²kR⌐á!█°2l É»Ç+Θ□å-î▄╥G╛Fε─}örK
╜j□nQ╒-%Σ+Σi·τ░E-╖t└>#⌠;░àò[Γ░╧&▄ó¥┐≤
Σ}°>:╕║e□┘
╢2ù├□AnY
[╞qï□╡`¥!L╛C÷πhâ·L□╙8S╞Iö)πy├┴└≤⌠⌡□d╩x>g8H□₧╧┴S□Ü]r!=^d≡"▀)[u├Ω╤δ□KCCΦ╕▓∞
.{U{e╝ú▄Φê╡W─█+ä∩Ä▀=Yƒ▄y╧╞▒{&╙⌐¥≈l-
₧jsòu∩jüGgi≈.£7`.□«${□{,₧&|α±S╪▄5□î╙╬kΦδΣ=□Æ╔K▌ca<ò├°µ≈X╨ùè2╕R%8╨÷□σ=züE6
vσ3ö□▓□ôM°ÿQ¬iêåj%y`PI▓(á]╟╥S@A□┤□□>a▀\è▄
╟4%┘□ƒå#·τ╙□_─G╘╟É¡YVm};tP¢x₧║V□°,╤╖≡╪ΩB╓╖s≡4òx>□;Hⁿ□?/│$~αóßô□r°Vƒ
/╫ ╫₧J╟~1▐Σg│
%
□»Jíf(F(oΦ

e66{t╤5=ô`à╓p╖╥¡╨Zx╫bΣ5≈╞VdSbUú╓bb

V╬Γ□lf╜╖nE$╨l║½};╬Φ╓□g═:°
W□b□"α⌡¼«│'╪Y□∞∞

╓Qz█

°S╓NΦ{╥⌡□{w·±□t ┬M║▌t∞╢ûâû╨□□
µè·eb□□╜ε≥π9┬═╢I
□/kë=ùk9H[~8?ßd□\TMÅ)╝▌┤ä□Ñè╜┐≤|□Uªª╙u□+≈wV⌡╫zæ▌ë╒2▐x[╕□òX▌□δV▒╚┤┬*;å╫66□
3╒>¿éÉ□¡¡lΦ.m₧hr¡□□u$£µ║╡ì^-╧+5□½`q□&╛▒╓Uσσ□=d┼&-
╙┌□M□¡F╤Ñ□tj¡╒ñw&║*║╖q$σ¬╬┬□X2□#OôφK<Mαæé5Ω&Σi□ú╧╓Lα□÷║KyÜD.·\«¿δ□╥≡±""⌐□
≥┤Vwòè├ïÇ╓?åvCÆ┤Æ□.°fâ6g╙ª╝Jú╧ +yε╝╒εⁿb!G─□□≡√□lf□
₧bö░M □l╚
╛£╟zq□£5#d□≥à±B<ΣæBD□x-k ⌠■┐G╗-▐è\-
>╖0EûñC1ª²□Åv;DRoσ"W╙Γs┼Só"÷╓"-σî7ΣHdx□gá%Y¬U((öAJi
g¬╨Zóó⌐ó½ª4a□

F
╪□yôB»╥ ocT∞■╒ß»╬ni
¿⌠<╥Bò□Γâ╥ Φ
■T╚□≤ΦUé□

XM*╜U∩≥₧"ü°a□ìA□╛ φMp

<Rå╡ °

ⁿçΣ≤□□Fò┴(ß-√╒ÜδûZù▓j╨.╝ò├°.σ∩ù▒ì▄DQ*E■□ö╬Φ6;╜□□D≤ƒ╙i)åe└
╛╤ñBo□□═∞□Tj□è7¥è<αqB□°óuV∞≤□ô≈┴⌡φ!*ëFΓ░ä
Ñα1é#╝α▒,k╫╕∞□ⁿQEP₧□' X╘é□Y°¬"N□□.@■{□□-54ñ=ir.
╥ÅRΦ]N∞C ]t╧□╝╪□x4g>HÑ-
═Q≤o┤□╞+g□\∞L▓Xè¥Iï|IΣ}_√╗╔=}íukKjKM■÷¥φφ[3ε■₧╤ëç¢3┘fâ/^r└f,k□ò$²\╧@□□╪|□
Ñ}∞┌□µ├Uu╛╩ñ█^▌QP₧┘□

o□m╤╨X╪□≡║╣║ⁿ≈le□┐╤Φ
ûY□╒UΦ¥n╧□[╓7
√B╤«╒Cx╫Ωìh╫*xd┌∞╤□%2/,l[=ë□─╟
Rå□ö1▒É╨b±╢╒M
Θ╞:□╨êfâ┘á&□╔□/□□>á╥Yy┴ªÑ╙°«:w╙═□=ê□;⌐á□═çÄ▄▌~⌠╙ß

1□≥J°tkß╙ìπ°K□v¡-┬╗VßδD╗Vo£6╪YΘ±╨╢Uî≡'Qï⌠|%
√V□╡)$Hï≈¡Ä
üñƒR□,zï□U┌··□Æ╘èF▐ógÇ?□4■░²µ#çÜßôæHí╕÷├ú7¥╗□Ñ╦çbö╟□>r□╧┐║╣?Æ█╔{□□HV0□nû│
□█q├a□£K╟Θ¢αⁿü╙g¿zεìτd□r<g8L□Äτα╔Å□σHn≈wεΘε┘╒Ω±╡∩ΘY╣'k┐ì≤ÑJ□ -
gä▀#Tπ╤ü«üCδΓ╤57

÷\?ûH¡┐╢°nM┌σ¼□«k_ƒ4╗□åßêV╧¥□7ôwB□RW` ╟|,ª τ¥çΘ#ï°GûE□Σ|

ƒ╕ Σ

╧y÷q|èwÿì

─│□□Çé⌡YàZgµ╟6□═úΣS$╨ü÷ù□JJmΓa;½╕

É□└   Ç╪G#AÉv8₧)bñ└>▐x□▒Å□ó-
è,°µ¼└z<tXτIÇ─qΩF4╢q<╕En6─CÄτ`'*q<□√╟≤ÉÆO┬C∞:6┐Uk@[¬5Üc%q╖.□⌡'╦∞Éδ╥□¡/K╢□
α╚:°pOφFα╥δÆN□Σ!FâY0¿»
$óò╢▓8gDP╧¢L£I╨:Γφa_ªm 2êyH□╬□

~╫!Γ≤□-2¥╒u□□╗╙┴εε`ÜB╣╖▐╠z m2□µb ÷p¢'

┬■÷□

&Åçn;∞1□╙#≥⌠┬8Y┼╝┐□■[≡u,ëdäwiï=£≤ä┐¥+▄ü6=Rÿé╥
û□▐+.a⌐I--╜□;
Σ%\¿{G│?ô(10j╡╨Y▐X-¿÷□ä▓µ╨V%+%!kΘΩM╘╗□a□úÇΣ□P4□L╡ò╓¡¬u□□5εPS╪≥tñ7ßRδy╬fw□
□^╧┘|<╘vt(W╖┴¿Q╘D□U£╤á╨□⌡□âV┼□y¥¡ó⌐─□□╗T□{□G-
Öµ╬æMΣτ0?┘╢└On)≡ô├Y┴Rµ≡DA⌠aÖw□bÅ,Pö}╦F├<£+Ω√19è▒êúPêú4i5}r□Te■ù╚□□□Jµwö▐□
□¥e6╢Å╒²Ä|ΘG3vτ>ΣUDIî÷)átΓ,□╟éIàRJNù?`□□·╫%t■#i$?E╘I5¿₧&¬┴ß╙┴╨`Ñ╓1□gûHh╤Ü
y□□R

╬Å╟├7x,└≥≡╝xBde▀□╢ΓN<öâ²├UÅτ┬7╨ûçτσ╒%weû."+ûE\┼□ε█¥-oë:í□á□Fm-
MG<ëRKwO┤ ▐╩□î&0ó╤i╡·□ìU\πDï ╡─p┌ú╓ΘX½□9□ú5huÄrW<í2ÿÇ▌╚ï.ùπ│□╪b▌rò
╥□G`!&□ⁿTµ'g▓Z┬Ls□□-□o╨áW

_ε"ÄBï┬π9|n□GæS[-□Iä □REƒÑ4F┤3ï#□Ç╬┐ñ╤Θ4α4.,0¼3±□¢╫"q°PkU`╬εrïö╞

┘I    |Kq╕ΩQäK[! T98Jÿê
8è"□}RDbⁿY≡□èp□Åf□6RKseá∞[╢├₧□╨≥`╤K╖□#ü□╪╝ò|□ª[-,zk⌡KT▌ïr□ö╥sQ0bèî{[╖╡╖n
╬z═Ñu□w╟/8□6┤╡¼»|w7v

□%┌ÿ«N6ÜJ□□╧·kⁿ╓ÉçW;πí╓ΣúíÄ╕╙≥_∞} x\Gòn▌█√╛¿7╡ñV½%╡ûû╡╥Æ%Y▓,[Æ-
█RΣEïùDVK▓Γ╢ñhs,Γ0då%□é□□`

|,ô╟äu₧m

&□6(□`B□ä□□!!lI□□Æ IHÉε√δ▄█¡û∞äσπ╝y|#ƒ╧□╫¡[╥╬⌐sN¥¬‖¡«èl(L/-

█mÖy┼□í├╥

_ñ6╗rGFfùá╬.,╚╬\ôjt°ª-~+%α≈[═╛┤L°#α╡F░ε»□o□□5□X≈╗╨Ñ▀Ä£2±-
┬?jRæπVrΩ┼╖ï~*πQr╓α);σxò□ù°□q╜╞à□ƒÆSÄ2)□□Y¥¬ΣäP╞F9iJN.╧öS=Θ<□ô╚!╓°▐½▐┴t╟╞
╝ÿeµ▒□V═□Y+δ`▌∞Z6╟╞╪Q÷fa□═□F;□┼v┼jn╝┌■ªⁿ±⌐ó⌐αu╤£¿┘m¢y□█░I╜╓^ZΘ¬î▌4□▌╡┌▓r╙
╣Φ╘M1]z╧>_·╓ëÖ□3□gon╣╣ⁿ·╤╡ú■╛□ü□╬«=₧=bmâ╢┴XXl-₧╢y⌠└₧åΓΓå=□FoP₧╤àç

å┬¼ΣüÆ□□╩íZ╩Y□o

□□┬·ù<┴□¼µ»ôoCÿ·Jⁿ□⌐ê╘□│CUò□σy╨gè≥ΘU>π≈u+«W~«╝»≤,┐╬]Q□£ƒΩß╥╨╨╥≈sx╣ó¼ó,ç°□
½╦±∩≤□ee□b□╟□?╧□ 1Qvß
Ñòσσ9BYeeÖ0╧o.επ°2/²~₧R}á£□□e□ï?¼¿({□□┬□æ╪├k{□@╕┐╖╝ñjí
⌐□)-¡□âJíE□□O≤╟~TYZYî□|5]|@|X≤î¿╒fíwz╖ï▀□?ª·□«╧╥-c}Γ²Γù4┐c□╨)à╠□µτ2╪-
□e╫û=QªJ+K++╙ÜsPts□▀vª`N/o^)╩*ö\ûÅhH▒òeû}¼LeæK;│µb╝ⁿY}┴\L»lb╢∞+¡▓-T| t□
□!╠─ô7Q»ª}ä┼/a ,l∞,┌±□{+*≈▀▓úi¬╨n░Ü

~ô□W}Igm╓°`FuI«┘ε6ÿ╟¬]┴

│╬δuTD▀wφ┴□╟╓à▓¡!□╓9:{0╖φ·═∩z¢┴b╫□═-Y□□ë╧ô.╛L║╕□║j╥ⁿéÑ){\j,|╟D│╥└\Ü9ï┼í₧≤
¥□▀z╨A;*
·9√□Θ└l╤╟┼P╞°₧ï┼K·□□ù¥UÉ┤Mg≥`(6□□ï╧Kvï┼.□≥┬□vB╘a╘▄Öæ·ñ'+#┤°¿5%┼*~=╙ò□Ö√─9
±q═∩ ≤]è=□|d▀{X╝MMÜgq²Uσ·A╒□|╟□²y║~7┌°4]_Pε_□o&□▄O□s╤'-T▌¿y

½ù□v-φ7Uö-raq⌡NLÿî┬╗╛□*J╧╘╟σ┴╛ _⌠┌µ2╣C|[·σ█σç/╧!┴ ¿
à≥á□^lâ┴¢╣!╙6□╦$W≡█┐¥°Q█èσLè╝.û╖£S|├φ┬□äòÖx¢½┤|«╛|▄¥R┌Q_□M╡√7n½⌐j≤Ä£α55°?
]╝≈□}ƒ■ⁿ╛Ω═)□çAÑ▐W?╘^╕ f√`⌡·a■9┤8¢πK╔┌╨▓{┐5╖ C╫╫~τ╛{G#╣z¡▐Ωé

║íâcñâïΣ□-Φ□╡╨┴fy°φ»`└φ9¢SûSf±ƒ□▐╛┴┬,╢□[àw▌⌐z┐ªÇw

»▓üqΓ⌠h8å|£ßeZzP±éu°b╩□g5▌─6╞ëúí□δ╝0-
Üy┐□¿öw□yKc2uûx_æ▀□@;╡à[□δΩ≈°[¥□ò╫Σ7¢2≤J3Ω┌╝ßè┤£╢║£▄ì}k╙¬èsLF╝╟∞5╣□╓¼¡≥µò
°τl¡═U¥Y╫╗>3═«╖┌=ÄfçNg╖□δ*²y□┐┘□«j_[┘╣6]oK1□=«MVì)ú*∞□gªΓ▌┌¡╨¥□║δ"▌═ô?uCw
_ □ⁿ□Θ╥Å√▀╨ⁿ□cb¡╝□┤┴Å▐q┌Ñ▓≥NóÜîƒ-
╞;╤WP:·ûo┼«æy¬½□jt┐.½└□pÜT/δ╡µP╨σ│□T▀ⁿ║Jg≤{╙2L□¥°lèÖ┐6□┐Ω≥ÖU<╡≡E░▌h╒½Tfƒ¢
╥╙cΣ□d╟Æ≈□?π╓g1□·4û)ÉΣ,╙╖UjÆIMΓ≥¡RK-XxÇ╢E²□₧^Q─╖┤+j╝ú'□╧Tû┼$·;3├ßα≈î:S0α÷
█
ΩßÆ╥□╡┴µK å░vQïï?╖□

6!C╕╙φ┃≡]É⌠?¥₧X\○╤½!9ƒ╘─ú;
▒\y~√σ£╣∞

│┘Ö□Y╬8□╗Æb-xY^╗τ╟┼V□≡┼KP£J∞▀ûùò╪▌KyÖΩ╒aì≥□ì`□°|Θv¡zπΓ»ΩE╝#▌τ╦4
□┴(□░╟≈□□Fq╫╨w─ù¼vâ(ht┌3°°-i*╜├">«3¿EQm╘₧\|□≤^e,b□□┴□à÷«=πgßè√änfbÖ
┬¡╠═è0□YÖ╔₧√n╡║P^«╧¥│q╔+µ⌠□K»▄·□w□äb□mUδ⌠ùçτb¼\≡¿≡ê-w.╞-
:½»└x4q┼δv┌W9-ÅW■¬6█-ƒτæHèK╓.»o-·₧-╒o=╪╢□}╜Ω╧O
╔Σ√ε▒ñ□ew_□╟≈[è¥9k≤□▌□êäRT°r□╩├~KèO╝╧ùÆV╤^Æ^□╔6±≈≥┬:w╙g■╢3│p±▐⌠Håφg╓î□nUe
\bN⌠Üì≥j □±ù1┐╨{╞É:gß□≡╧i'öo▒╨t■τïûTî@╝¥Z \L;▒ⁿ-
Rr√Φ5R╢âZ≤|σ╨√□∩┤ªÑY┐8⌠₧δ╨nO½φ┘┤woSw]@=<⌠»âσ□√^_JU⌠▌√¬□┌≥□~□┌|êf
4□aåK;■£IO╖·╬ }°Y╛ì╧-╟ò□ì╤7□□ò□τ╓±□ΘÜ+I□ ├ ╙╘ü□üM
□□

·µb(╛╬8□π□£-q═┼JûN²ß ô▐ç%vTò¥⌠₧ÿ╢⌐Nzcª□-
τ;□δCδ:½k≈m□σ6φ]□¿≈□╬hT┴)□ô╓f╒ZrjΩ╓□°∩▐╓
┐╖gMGCí]½┘erÜ╒▐toß╓ß║µß╢□½⌡\(dsÖΦ╫½ï▀t°£-¢~▌╙?∩=≡í├⌡vOjN«<□αc1╝╢P╢□□·ÿæ┘□
□öπ .S─P2Σ□!Æ╬□P□]éOkOKq□°µù□gJÜ]+>╓7φπ▀╞ë/┼S─Å╞zµù▀ä₧╥ w
╝Xìxα-┬£à▄□<
ª╚<k□α!▓cPtZ·%□yç▓□ªWpkΓ»□n╖ÑΘmi.□í╤ⁿpaRg╟é▄c╒┴+-S□!9hÄ┴ΩΣ∩{N¡I='⌠£╬.5≥□û
]┼}▓╪kR□≥y*0╣tµ□à╥ç/ù█/ùôÉUW+╣lƒΘä╢□√Kg'¥⌐!√╖7Ñé□□≥±□O≡-
úS╥¼║g□â═c│{¼□ß'éá│√▄|┐≡@Jï7ÿj╫~S⌡}¥╙¥Ω▄jL1□─ƒúq°□}oX╕G┼ ñA¡U#²⌡D■#~7¬p,╝
Z£~¢VcvX°o╦Σ┘□[/┐½<e¬8'∞9╜>▀u╖░□a«□å▒□°åx MM▐2║Σr·├è□«(ô╝M┤▄ôσæ0ÅƒRΣ╕▓φ□ò
╖"\╣ΣNΓ╙□╠□┬╬4ƒ╧÷É='EPï:½▀σ≥Yu□Gì+═φ2}┬ûεOu
X█ⁿn╗▀ª□ ╚□F⌐sd·äwdl*¬╝&□qoⁿ@0±Qƒ[kMMY|(□#┌ÜèµLßs┤╖4?ªDî╧'Y:+PtÉúσ^Θ`□□

Fµ╚Ö╙j═┘sn>XÜôg╥ùπ3i-╞(öqg╧┼ΓÑû═ñ╣

V╢[¥□╙n╬m=╕yù▐╨OΣ▒Θⁿ Ä╧ñò6╡ûñ▐₧□Yπ┘▒-\□r¬□□□6τ-
>ù0τú⌐.╡5\│╡*╖┬°[ⁿú;╖□mQµàl-█$ƒüpª╕æU#└¥)Hot≡╡ü/╜╕qÄ9□~tA╨±ñπw

ì├ßmÿ
rδ┼╧□QN°├ ╡0g╟□Y-ú2è□ì□úû=kPßΘ`├\lΘyƒ|Ü□¼nê,;■□s┬+⌠αQ

¬┬Φσ⌡╥¢□>MT□«cj│╟i╧H│h╖□╝□àΘ5ⁿD874öf√lá└V╥VµsΣ╓d╗2╙}û□âµb(╧□Hmφ╨*
┌─'d□□⌡wÑùf°,▌ùP▄c>°J╨ç¬6□Σ5ûµÿ⌡i9Ñü╧{£░EÖIÑz╪┴G4!>K─<Ñ6╛3«╨{╓τ╨:τ2û□í□╙└
□-¡╬j3£s▒î$πù_┼≥ë8O∩▀║°ª▒□h□<X94□≥□≥ê±j1ùvh²ö=k╙-L'─~ù

–
óÜ¥ƒt«╟Z·æftnææ□ß│□µ□ÜXX|+Å┘g╥µösJΓCε▌±[g5isësI╕¿╧·÷è¡▒ô║σ□─┬□7┤6┼╢□iφΘn'
"¼7R□╬½+≡j□■□W:&¿ ╒6▐æù╕u¼Mx6▐┼□δ+╖W·²σ█╨àο&║□Z░╡Å■F;ìá╥╖ö╧'£B₧╕ =N▀H╤«□Γ
Ñ31¢&√n±□ï1ì°=╓□ß}δO~▌┤?╪z├5¥ú¢33[nΦΩ□█£·&wQSQES╪µ) ┌ê╧\½°╘╡-[_□√╙└u-
-½»9ⁿæ├√▐▄Ö]╢gªe∩¢;s╧÷□σ▓V
k┼□u

□╤√Eï7MeÆ□Qæ
σ%□\Æ─F╞ⁿg┤t▄Üⁿ:Ç▐ûï5□╙bï╤f5}ß╤─╕╤|I∩O═≥|└d□□R]«T┐[╜εÉ┘òΘuδn╞▄┬┐Pδ□╫\a¥╪«
>╨JXµ□╗*öBZ□⌐╓$3τ□idε╤╛□Γ╫±πÑΣ/ä─vïu1╒æbu▌Q╒Rα╪╓₧╜┘,█α╓Y
·╡¡·M╫5f║*·Z▐─□O□b┘4wf █·┐¬÷┤╓Ñ¡kw°║1╖2δ▄n[h▌╢H▐Ä┘├═╖B╧░É%nQO░-¼■tY}□
╣)ä5╙Ñ
)1[(3T□║64□╥äBÜz¢╞□7ßCñ9-h"δxgID
■]\ΓÅH┬┘±úCVⁿXEiÖ'■G╡Γû5;g╢□┤╓ΣΩî□°>T╤▓µ└┴δ°wÜ□vSgßµèt{¿*□▐Xæú7□╨┌é
┌╢é□gSï□s╤:╓fê┘Ü╜□r,).¥▐ò┘&┼ù╥┘╘║#┼∩K±ΣUçⁿkB.O¬╟Θ≈z░□uYç√≤Ü½r⌠ó:½b3≈╒Ç░F▄
⌐─┴°□d9gⁿNSÉ7▌□SexL&¥GwÄ█⌐$Θδ□┴┼┐\æ╧;Uî&$<Eò¡╩□wªv□22ⁿïτM6╝A░Φ├àk□:S-
□□□&z╓▄┴_╥▀ß╠╩╟q□Å9,vδ┬à≤ï□ΦL□!(╢¬≈|□Vp¬$
r£iòöhr#dö\ì;Y·%╩°┤L±kô4ƒⁿ²□□≡ëo?Z╫∞:║╡╕¡"S½╫Θîå⌠ΓµÆ□úâS╣⌡ÖN╗█)╝55u±£»6c╤
Φû\▒bπPK«╓jSk|Θ

Å=a╖f∩╢

â3U╪ßrùò²L□Cδ╗!wÜP(εRd^µ>¡raIpΘt╟ÑhNv

,>Θ%╥▓]Ñ<Γ.âq±Æ.ÿπ⌡¢D╡░e±á═l╡□»ª8╘%■ÉkßÉ├L□L°Y,·╥\|/□ù□□≈!▓ñ▒|V╔□7□R
yyEE*G&Θ╙í*Å≈«äÄ*Φ▌OΓDäΩÑúí»÷□□ß°)èk┼}-
x ¡ □ì÷ε╔j-n┘|░1cO∩P⌠÷¡█w┤9┬δï▐φδ╣«{╟ε}═╖D▌╪╟≡p√⌡k≥□7□╡╫dfö╖Σ7□□□□□{+□□╓·
□ssR6,₧¬┘^É█Y╜┘y#≈║□÷ÿS]O□2ê□┘d$▒mΓúL├ Φæ╟ⁿ□ô□T□2LµÑ╒]█╝qo¡▀_╥□╙╞²╡■a,□s
╫fZ£9╒╢æ╡Ö&╤╥±û}σ%╜o┘┘q
╛Ñw█⌡□□·m□╡█F°τA■
mvù¿S
"Jª¥2{╧ë?<¥□)□└√{#"eεƒêö:ô~±╝□│┘pΓCNï╬t°╓δ╨p▐ñ7=Θv8]≡»?∞19}.°÷ÉZ¡╪/£vr┐∩`
≈è^╒□V╞≥╛h□╜YNÖkû°ΦiâX$_êè□æ□-1s]□f└⌠z□□[m&╟¡EδB╓⌡
üèⁿ

╜Ck╥àJδ▓╓vVÑ┌#[kgä⌡₧_□┌²■t█¡kZδ╦<%δ□nGü├e╨:□µ┤╥
9Y□╢⌠¼¢ I?+zTçY3½8╜┴Å9≤Ñ/┼ⁿ~MImß}$e-□╨┼;ÇMsÄ[░"B"ùD╓²□Arφ╒c$¥$&zr7-
l□╒ò□0∩░Θ╥#╡╣;«ΘΘ▀d░YîM-=û⌠ó`fu□7⌡h^vi}Vth{c╫~ßhq√┌

ô═üΘ╡ 7╫₧b▀╕╢«╔µq┘ ¢╫y┬Θv°█i≤8S⌠┌□ç⌐╗ccÅU╘≈└?ï┘Y▒Z5@Q1╟ú"∩__Ä□┼╗──
2□╥Gπ□║├öΣ╕X!V{vy²iε┼╟LV¥■□}0/?kùτÆ≡█τäÖ≡¡zúQ□½─
=5`□▐i5a╕▌)▄┤°O┤█/√îΦRφcÑ¼ΣT▒fóbq▒&°@╓x

d╣RπWëìo□□╥╢δ║r[□╓τ╒□·▒□╡Ω|ßÜ╝□{w÷gV°lV°Mhv╗□□τ*⌡□¢□▐S╜╗.`░ÿUÜ□o╨l57okl╥┘
▄□\╝á≡.qäK▌╧NïÖ¬□╠┼|°U

╨$¼#í╕σaæ□┼xL╠╘Θ□%¡?Ég╒
Ä┼;ípß

╗UUΘIw,ⁿ╚jtX┼Üê╦πα╤p□╝1î(Æ╩r╕v6□

▐∞∞ⁿ|ò=Cë'v╕úè□+}XÄ'W
ëK□q┘╫»╥πa°µ┘ë¢vεzo□Z┐εÜ*▀{w∩<║~CcC²ûi╫ûÄ╢ª╓÷--
┬╚╝â{v7tç¢b╣ì┼~_amΦHSx╖□,¬¼,*¿
V.■gIcvfSIEM5="ÑúΩ□ ┐Ö9NÖ┼╖┼ï□╘,₧g<∞╣½╕uΦ½□5□1□ε╛n░≥╓Oª:ⁿ*[0═┴□ΘN╤╗╒v═
ÿ1[OiM≡îS1-#├+▀°≤»═T/Z±∩╡∩ª°k^p°gx╥*±Σ]║≈êÑ║▀C9z~╙

ƒ□¬▓▄Y-Γ╠┬;u┐┬¼÷½2
▒╫#▒Vⁿ▐□⌐:▐Ç.⌐O¼áƒ┐□i╓'æñ²╠ƒ&▌Z╨3·╧╚d°Φ□□□□}µjd╩_Fw¼$s╞δ╥'-
┴$□WΦ±+╓Zg╜□'[═╤
+Φ&ó□m□┌╗@∩["GÜπ□I⌠Γ╒╔╢□⌠DJL&ù-ë

(t╧╒╙φwfèô°╤≤╤òΣ□{}≥·ù(5▌ ƒƒV□ ·àLΘ v%e╘□╜□x$╙éƒ╛Æ▓>y%à&Cô┘╖σLτ~*▄,S^$∩%N□╬
┬Pé-èSdÿΦ≥J*□ü^Xs√Ü°╓<Uⁿ.óGû¿Σí╥⌐d*Ky□ ·□'¼□ë*Æ■U-TΦEÖ¬╛ Zui%¡φ«.¬i½┘╡Æ╓╒¼{
∞jT√∩uw╞⌐╛╡■₧½╙·.╨╜

╒
τ□ï6□6▄┤║ß°M¢¢εk·»ì∩▄°|s[≤7Ü┐░)▓Θδ¢╦7□ñà╡|▓5╗⌡□
⌠Ñ╓_ⁿ╖╨o■6╘v█*²□BÅ'╙ûjàn□═myε/°¡i[s«J╙[êS{z√□óΘ÷πo@□■-
h█Ümo▐n█▀¡□□v\┌q⌐#½π+□_Θ⌠v■╙5┌k╛┌U┘u□╫²;}╗:@ƒ▀╡░√φ{j≈£Θ▐□·`O=Φâ╜b»▒╫┘√Ä>?
hl»

4╝≈ï√Z≈│²│□¼áΦü╪ü╪╡Ñ╫╛z▌┘■o□╝cαΦ└╤hN┤□T□mê╢F;ú╝╤âá├╤⌐□□Å╛-
·─`Θα'åZçε□n□╝≈P~éÄ-Ü Äîìⁿ≥·□-╢□╛□⌠d∞□Φû╪╗b∩Å}4÷⌐╪□Ç╬┼.¼╥▀?ìZ $yF□ú-
□╝□⌠╥ΦΓΦΓÿû╚:µ□
Ç┬c╗s╝a╝e|╟°╟ë□ViòViòViò■'╥
√@O▀≡⌠─:ó9╨-
9Mnÿ╝□¬Eí╧M_3²ë□qòViòViòViòVΘ∩£∞½┤J½┤J½┤J½┤J½⌠7ó₧Ö≈═ⁿΩΦ╨╤═▌¿□¡┐±□□Æ■≤X■▒π
╟₧ƒ¡₧²g╨▄¢r□┌D╘┐J½┤J½┤J½┤J½┤J½┤J½┤J½┤J□□M¼╥*²╧%┌-A`╟≥4□─

3c□ß╟L═£╥≤└<┬^Θ□`T║

xHz□8)²;pV‖₧⌐àô╥╖üτ%<%╠K?dj╒nf□÷03p□58ÿZ·
0*}□8)²□8+═3çÉ╧≤à□a1a
a+a;ßI┬≤ä□ymαé┤j╖─≈å╨ô~
∞%î2#s" eµ□╟°ÇQHε□╟°ü│╥Åÿ□w┐ ∞ü$aöy7╨ëg├TOÿΩ
│□╥╧YX□Q2,╪┴%,°┴1,□PgX8N9'ñ╟Y□╡U□{ñFû□^/□'
gQ2□2 □xAz□xQz□8Åⁿ□p|□ÿ'=□∞ò╛□îBô
╨φ├└IΘ□pVΩe
╨╧3└□a1a
a+a;ßIh╛□╝N□/HτÇ≤╥)╓□+8X7┌8□îB'▌$[7j■=δFδ-□┌Ñ□²╥Oü□╪«[╚ç╒║à□a1a
a+a;ßq*y□₧╨-£ñ£yÄ╨å□∞Y|
8!}¥⌡é∩C@n±^≡²-δ□:Y□päu□/▓L╓
9GÇof□╓G╢Φâf.□{í≤>╪Γù└(Ñ'í·>╥mñ² ≡╕⌠□≡╝⌠+α□Θ
α<G°₧æ□@█ƒ□Fí·□xûπ,Ω9Ç≥╧□/H╧□τ!█□H√2╝G
□ÅBåGÇy╥²└^Θ"≡□⌠□□Ñ│(⌠å2╨[□╨□KE!╓V`'⌠□□åñ╖□° g□Å╙▌[)}□ß
i□xû╥τÑ;Ç□ñ╧□/Jƒ□╬K ╞ó╨d1░[Ü□÷HU└ ≡=□ ┐╧

æτB■O╪$r>

Σz¢ä╠┐□÷BcôÉ·Eα$3░I╚|;╨.²+╨/}□□ÉN□;ÑÅ□çñ∩□°
g□ÅC₧I╚╠╙╖□₧Çl ôÉÖ°τß·ôÉ□÷Çä_□÷H□└ Θ├l□≥□□:Ñ{üyä╝╥}└□╨÷,$Ö□┌Ñ~á_:

@ç┃≡≤»□#ä┼ä5ä¡äφä¥╥□p?╝n□2□□8"=□£ª⌠

ßqΦv▯Æ≤⌠mä'╨╟g╤G-▯₧Ñ£≤╥▌ └
TτEΘ.α╝⌠U6ï▯!óíEC└-)▯8!┘K▯!█ï└▯a1a
a+a;ßIΘ2≡╝⌠Sα▯Θ▯αE╩Öù~,╪╤ΩτÇ╟ü▯╘÷[`ä░ÿ░å░ò░¥≡ñ⌠{αyΘ%α▯┬ï╥╧Ç≤╥▯
·¿φQá]·>╨/]▯▯ñ▯ÇêÑ└▯a1a
a+a;a°4▯<.²▯xB·%≡<|▯/0-
p₧▯à|Φß▯`Åt#pB·&▯}Sê▯▀▯±ì▯▀▯±ì▯▀▯±ì▯▀▯±ì▯▀▯±ì▯▀▯±ì▯▀▯±ì▯▀▯±ì▯▀▯±ì▯▀▯±φ▯▀-
▯φ╨s'°^▯▯á╔NDÆ▯αqh╗▯▯√Ö╨ëHΓ▯·╚^}d»>▓W┘½Å∞╒G÷Ω#{⌡æ╝·╚^}d»>▓W

□□┌Ñ∩□²╥7Ç□Θ>αqΘnα öîíåùÇgÑ_ 1H{Fÿ&╛╙─wÜ°N□▀iΓ;M|°ë∩4±¥&╛╙─wÜ°╬P
3T├

╒0C5╠P
3T├

╒0C5╠P
3T├

╒p□·/□/H»□/┬^╟æ {ß8t√ÿp+Ω□

□!,&¼!l%l'<üv▌□./□╧S·□ßEΘç└yñoâ~-□┌Ñ°Ç~Θ□└□lz□¼╟╙╟Ñ'Ç□üτaú█`e□É[·6H╥
∞æ╨ü□≡è□ÉΩ20BXLXC╪J╪NxRz□x-V>A≥£Ç<╧□τ╤«ôÉτ╫@;í-
%OB₧gü╟ß{'±∞╧àôαuA8ÅÆO□y╔≤|L□□□≤!ßyîz□ï k□[ █ yδ╬ú╬╦└□╨╧yî}Fαyf□^
£τê6÷□{ñ-αä⌠Ép□|ƒ□┌┴δ□°■□□ ╞ç _□_Ä┼ä5ä¡äφä¥TÆ≤╝□╛<}□3¡
─≈□°Ωü£∩□╥φ□≡à□Ç∩□äïα√□╨.}□Φù-□□ño□Åú□]Dm/□Oé╦Ex≥oäïxΩk┬<₧·□╨

■₧╟SO□□╨╪<ñ5□#ä┼ä5ä¡äφä¥⌠╘qzè≈öy╥╥<I;O╥╬ô'╠C┌Q`Åt

8!}╜W═gu□»▀□£öªü│╥□╒nX≡□□╤╒╠

tJ?□µ□÷J╧□ú╥0≡É⌠[αñ4□£ò6⌐zαWÅ□#ä┼ä5ä¡äφä¥╠□─Φ□</¥□^Éε□╬≤4ñzÅ□cÄ⌠a╒□Å█└□a1
a
a+a;ßy┬ïÉmé╧QU7ú~#pä5¬^R╜"]□.HO¬^RδxZmF·eΣ<°z□ΘτT»¿^Æ^□╛&╜□\É╛úzE¡ß9j#╧AÖ
∩¿■Çⁿ çT□@·!╒½¬WÖ□°Ü┤□\Çû^U□x

╩#□e~áz
u.□_û^□╛□⌐^S²ü╥»íµ╫Φ⌐╫└□e╘Z₧□┘PF¡°┤æùíz□P╧S└ùY6≡□Θ1α-
$┤G⌡*┤╖@2,¿□ÿ□╕□╗,áNöWkyy╘ë≥¿□σ╒□^₧$\P¢yy╡□σ□Qτ+└□Θqα"□Uïx□9αÄ□╡àëj
╫!□:Tk╕

ü╨íZ╤u¿╓r□¬u\ç@Φ□□□¬u\ç@#╧ß:Tδ╣

╒z«C╡üδ□□·ü╨å┌└u□4≥□▐v╡æδ□□□□íCα(
□□·SF«Cáûτs□□⌡ö6≥2TÅÖδ□□□□íC t□ä

ü\□3╫!□:T¢╣

üZ^₧δ□¿τσ╣

ü\B3╫!□:T[╕

ü╨!□:T[╕

üf₧├uê╡▐□1─□░Γ╥>╤N½?□¡□¡tÑó²╧¡Ω ¡ñU,G}ƒÆV'ò╤0ƒ·)%¡M╩╫▒□⌡½JZ╧□57+i□
ΩnQ╥F±πë≥&╢G≈I%mfà║Wö┤┼¬╒╟σ┤▓¡(ú∞ε'Φ=·JZ`:o⌐Æ□Ö╬≈□JZ┼|╛╖+iuR□
3√>ªñ╡I·:Vτ√¼Æ╓3╖°DI□ÿ▌≈+%m□:□σM,Γ{II¢Ö;5KI[t¬╘╡J┌╩rQF┼□~┌°Φ╘î+iY╧rZ╓│£û⌡
,°╒Ied=╤imR╛¼g9-δYN╤zû╙▓₧σ┤¼g9-δYN[¼╛α:%-
δ·╙,╚╩Y)+c5Hm╟ΩlÇM░1¼%╞╪□¢B^3R□l£░-
9#Hì▓b▄ib1PÉu!o□δò)<┼»□±9ê╥3└(JZX□R□æ3╚ÄóD□j□D□╗╪1J□┘6╘|

⌡N□╟□R├$I□ ╟Pµ□₧ì≤□&d.e□|o¿─U5+"■²¿a□eâα█□>╝Ä□vX)╖□WçÉ╦∩NC╛╔D{v!□ä┌□{]yåH
□A╡□╫□qçτ÷ô□û╖Q«gLiiÉ╕Lπε□╡7«▌úxvérªQ*JZ
" □σmg[ □╫╬□=7Jz¡úτ□⌐─ ;□₧â┤^σ□T$èù
R■$┘t□▓─¡╖╘

~□□Rîα╔IhíÖZ3B-□I┤ú Åα YB╣║=²━#¿╪z□5≥Z√QÄ╫u

WGæÜ";Ló}□æÄæL□ñ
▌▌□α░ó)╣╓)jô╟söZ4@ÆÄ□ùI▓╙□▓╩□r╕?Nô□'⌐▐A┼□#╘&Y□ôΣ□ô¿╡_±Wn▒q%?╬σ□Ωëæ~╞□)Gæs
ä╕╧uNÆªû$α□╟⌐-
r▀êδVû=F^├=ßÉΓ╣\¬#(█□■St5J╢Ä√╡¼3Öïl╟QÑ]cñ█âTrIΓΣ□q¡▌H╤╔¡>îδbΩ╗╔╔╟ú┌ÄP
╟H□╙J/M╓w▄√F□Oµφùφ2A▐□≈╤A▓5≈▄±Dkd□çò2ô╕ÜUjƒB+d
═$¼╘O>┬{└æeφèG₧□H╥Oⁿ□□■┼□]å╓Vⁿ╬ò±¬÷èV∩Q<'ε·kQK9"╟δ{·□±îÆ'r.ç□6XΩÖW╞╓a┼»╟□
Ñ╣τ╩□E·A≥¥ 7±╓╕□q n"ε6H2└≥⌐ù□(≈â¼ò╝bî$¢□±xU╤J@Q╥-□≥╚□▐S¼°\
╥╟╚çå╓ï╕mÄ!╖▓╦:Ä╫*╫□#□╕□C$¡□τΣ║«µúôΣτπ╘vY
±τ╕U{ëç□iÄæªe═L%¼□/□Å
□J∞µ╝╝êt└╦ì+^æ□°╟I»úJ|Ék□T«√òÿ<H□eäZ(KwÉΣê[yÑ┼ªö'd Ö╕"g(╤åó?+□╚úBöt:Ñî>r
ö·□%°¼lü□EÅÆ₧□¿?]MgGòûÄPOïQƒÆ{■ò║τ╧╚#K>╩□,≤α½╫.╦≡╫Ω6╣□╚ú{P□ƒ°╚r□╦╞╓ò-
X□□W╩UùΣ□╝%r[Σ┘B<VN$f-Q□{G)Ä⌠┐nKe▀δ_µUr<□SPnò£₧ª■"╟°(ìc#Jlæδß%c□²_▀Gσ(>¬X
f⌐÷x□□IÜU□óx7óΦÖGu
┼╥AÑ
±□F\╦╦╝║ê,╙OΘ(ï╧»V╞╡ò=!□E\□ñ8}öf□#d}n╒~Σq

úDⁿ^ëRτ╡+bgü╥{ùó┼╥l
.=_2:²ÖúA0}E□█Γu□3□▐|=≥d;┼╜F₧¥─öQd╔╗▀häï{σδÅr▄r¥ë₧3Ö4□æφ-
{┴á┬KÄ╪úè▌ï¿=□╩Φ□ƒW╚≤óa┼╬q?û²j\Ö∩╚□╞h▐▌Oφî{J?[□σW╞│
╢Hh¿ƒ┌╬⌡6ó─·¿╥W□ö╣÷(╓Ü<fÄ╨l|Æ|Sæ±⌡mï⌠╬σπ<¼]Éñúh╥□!╣?ⁿ┘⌡▒ÑUM╝⌠╒ú[╤èΦ□╫²╨°c
┤*□Y╤ε╕\Ks░Ñ^┃4□┼mX─Γ½3╛□ï_□&y╚8¡┐bΣoçÆFXYΩâ$╦á2RM'lÖ□Kd□û(□ƒñ^□K╚□∩╫╦}Θ╧
╫j≥□/╖2yñYε╙KÜ8Jz<≥W┌1>□L╙ΩR╓╠`Æ□QB╬sI/╫ú─@╥╪1⌡□±XÄⁿQjA|─½]□┼σ┘╪

Ñ»6δ-Ñ1">╩$»╧Γπ─╒b╩≥°&)V╚╣:¿┤√Ωcn δXt"╤·I≥╥Q¬]εEW«| Z□êÅoml3▌φ`-
╕Ω╞h┘E9[É□D□φ┬¥═╕┌ä▄M╚╔Cë¥╩²<▓T7ìCm(╖¢╞8╣Ä.α

\≈RîkaA║µWφ(┐□u±g7│-Γ▒□╡φñÆ]T≈vΣn├τfÑ□□ó□9╗q═╙¡□□e~;≡ö╝å╪óîë▓ñ╗É-
L┤p╡╢T[êc\▓φ╕ΩB²m╧▌&╘╜àΩπ≥s■-ö▐æÉ│Eæ┤ët─kµu6Cómt┼swπ│□σv□ &j│,φ

jC
ε╦m┘ L□p╫┼J[σr\?{ö;▄F\╛ máÑV5æ

┌HÜ%²5π│□Æ≤·[qw□ì□□xr□╡t'io│ó3▌┌mt╡╘*┘R═╘□«U«âMHo╟ ╓äε║□eY║Æj[«╗n║┐TJn_ôé
═ñ╣

║Æ¡╤LW╖╙Vⁿnæb╥.j╟J«▌Σë¢⌐T□╡xg┬CZ╙{eΘπ▐)≤ΦHÆDµ╟m¢,K▄½âo╨GΣZΓ≈w+û╛R/\δMñ□.╫
╬□τ╫½□}≤╚┴≥╥▓Üα÷æüë▒╔▒í⌐`≤╪─°╪D ╘╚╪hq░)□
vì

ÜÜ

V
N

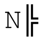

Fï-mâ'Å;╞Gw

nδ?66=□îì
Å

□□╞╞ÅM≡'é╝µ╥è`ÿ□T□□╗ ·cπçém²ú□c□çæ╗u∞╨h░m::╔·∞:42□î%╫346□▄8r062╨
*□Qf

Lâôc╙□□âA.ε╤■ë┴α⌠htp"8uh0°□╪√□°¿¬k∩╜╧Ö≈L┬$3$C-p┤□□ª1J─ê)¥äIL0$1ä°Åf╚L╚α$□
g&□╥êcî4 ┼H#"R□²╨ó╫ZK⌐Ñ╘ïâñæ┌öK-⌡úökS╦Ñ\½6Ñ╣□-
□ε┌δ∞Iµ□╨"m□┐∩┴9°_g¥╜÷δ┐╫┌Å╟$╬+»ò*|⌡▐µÉ≈V)Σ⌡J▐ªe^Å╟δæⁿ≥[╓π
╒□}□╓=¼├π
╖}■Pε□╖▀╖,Φcu╕Ñª□(□Ωq7çáöá»Ajp7·ⁿ½ÑU╛pú□j]□÷{Ñ`
╘δk^

ì□╙░╖ r6{ÇÇ`┃7□╨ò╨┣Râ╫□n
zCR╨
╜≡àíÄ·PÄ□jr□»⌡ε□<┃,M¡■░/□E6╖6yâ`□≥å▒ÇÉ□□╢└h░╓BΘ~□╦*⌐□╚ò|M□w}X≥5Ka╞5┨

▓@¢í«û□iÖo9□,W□÷╢à!│∩^o«─╖ymHjr7»ûΩ[aHσv3·ÜüΣá□·□⌠à□ú^wô╘□`╒@ë╦ßM╙╫

µß□Φ╨J╓%╖□□╨$╫┼£°╛╤□äåyâ╡5▌σ¡~wp╟»f┼¬₧┼ⁿßªà@□□éÖ╢7╬PP

║= ▌&w≡^╓□□╥1╧\

î□╪δ·□Φ~│╧□╨¡h¡╧vçª┴(J╖□[Z┬ìßp 4δ·δ=-⌡í▄ªX╬\╙p}xuáey╨□h\}╜{□°□3□K□k╜;╘╨╥

äâ╒xeí╓@└∩□╟ai╣╥ÆûV`l╡╘□.□f╬╩^3"Ωah├▌□╓π
□└üσ□
□}ÉZ□&^Én□Fo░╓□

Cq╥Vc»bε□Tü▀┤□c□
¼å£
√

~αi¡□τ0w\    ysX₧X□0>½□}⌡ìq-[□ò·Üδ²¡α√π¡oi□O╓÷Mô├"╬□J°¼╓╨Q□╛

π-□□}⌡▓C╞*@?îòu+2Éφâ Z
&╪T□dæπiY┍∞oq{ö∞╢e¬└│á;0|∞í5□ÇY└πe▌d6ì^□@╔(╟Kα╗▓9□□╞IúoÖ/╟µ°äZhrC
ï□╓dNuÄ┤╟□é╢╢4Å═□▒A╙µ╛αm╬]σ╗╫□≡z|ε▄ûα≥δÖv=X~à╧)╙`x╤-0□X1□ƒ□/6y╝┼-
*ÿ┼/□═+ZáOî□ê%?LlH╖rÜdT*&╩ääj68!

-Φ7Pαà\α╪└î'Gj□┬ñ╟B□□q9⌠Öq

\┴êBv⌐e□Lv═î□7N╘1?╗⌠^░□╣Cíûzƒ¢·□─□LY═a╖<ƒ·n└L6+Q╤[i>ƒ⌐□9
[Σ┴┘P-çï┌ß<╥^╟╡[

w7╓·X▓▀□~*╫═╩□╧+□╘ÇA━zÿ├µr_□ô^$$╨□□□5b└B╤╥ZY≡å╪Kε%╨├δíπ!/¢ó[□>yF²╘ª╩□□U╧A
├Ö╞F¼jli·î>▓0h
6Cc╝XÇ°□µPl╥□o}8µ`π~

╬∩±aα═Æ]□ª▒ò▐╕□╥╣%╠BF₧╠}<îeOßIíF╢-,≤*"╫□╫╤
½>□□g≥┴□ì¡<ƒE□ï╖▓□i~UiφóóÜ□⌐|╛T]S╡░▄UΓÆ«-
Ü□·╡9╥ó≥┌▓¬□╡□X╘□U╓.æ¬JÑó╩%╥φσò«□⌐dquM╓ⁿ·RUìT>»║ó╝□▐òW╬⌐Xα*»╝M*å|òU░«ùC$B
í╡U□½É□U^2ƒ□6»ñfN□¿E┼σ□σ╡Krñ╥≥┌JVf)□Z$U□╒╘û╧YPQT#U/¿⌐«Ü_□╔╗á╪╩≥╩╥□¿Ñd^Ie-
,╣ò≡N*Y□è4┐¼¿ó□½*Z□¡»┴÷═⌐¬^RS~[Y¡TVUß*üù┼%╨▓óΓè□╣*Φ╘£èó≥y9Æ½h^╤m%ÿ½□J⌐A3▐
║Ee%°□Ω+é□sj╦½*Y7µTU╓╓ÇÜ□╜¼⌐□╦║¿|~IÄTTS>ƒ□RZS□┼3:!G□□□·*KΣR□╒ÆbD└äΘ
µùî╖┼URT□e═gÖπìs□«|,pσcü╧┴φòÅ□■y
ÿ≡┐+
ⁿ■·╤Ç<zW>-╕≥±└òÅ□«|<á₧═»|Dáⁿê ╞╬òÅ«|Lpσcé π>&Ç╪ö□╫ÇÉ≤

▓û\∞ó`a□├ëD□■<ÖÇ┐Ñ└╛íOh6√╛┐ⁿ√Θƒqi4S-
□□6B▐Ñ┌'$0{▒αRφ'L`÷□τÑ┌[¡╞^[v⌐÷II╞^W}⌐÷6□╪k─Å √ì□
┌kα┐B─$á=Öñ□□LlSI>Nu╫├@═&w┬□6┬└┤┬□ué[?JûÆoÆ{╚
0Ñ □R·a:■□i'∩PüⁿæZ╔□ÜF)¥L□i6¥D½ΘT║ö╬áwS'⌡╤y╘O∩ó-≡╘JW┬² ╜ƒnáδΦ□Ç;h□².▌J≈╥-
╨□□Ñ∞wαÄ├□√}░Qq«á□□□╔Γ"!M\,\#□àδ─5┬
Γ□éS|B¿□▀□□ë-
□-±Cß>±O┬□qXxDⁿ│░Eⁿï≡¼8"∞□?□^□GàâΓ╟┬/`╟Å)y□▐·□<▄□<¼□-₧□-₧□-v□□»A╩
≡≡kαß$≡≡□αß□≡`□-Rüç½üç<αßK└├φ╨√┼└├rαí□εçÇçì└├ô└├

αß{ └├½└├□└├□αß╖└├{└├_Θ□é

x░□□W□□╙Çç|αa□≡α□-¬üç:αí□xh□-:üçGüçm└├≤└├+└├□αß0≡p

x8□²■P╔âv0ÄçTαß□αa□≡P□<T□□w□□≈□□½üç-αa;≡≡"≡≡□αßù└├∩Çç?□□ M┌⌐¥□╨o+═□-f□□σ└
├□α┴□<äÇçNαí□xx□xx□xx
xx
xx□xx□x°ÿ÷□Z║UHó?□ª╨¿p□▌/▄L□ä╣└├Bα┴
<äüç√üçu└C□≡░□x╪ <∞□-

□□┐□-~
<|□<nUn|h□ "ªè#Γ5ΓGΓ

qTⁿÆ°▒x□─⌠□J-îÖq<L□-▓üç¢üçRαa□~{v6⌠╥□±PB₧□-^□-~□<□□-~□⌐°I#─Z□╝á¥Nc┐²
<╕Ççjαí

x≡├S□≡≡□≡≡□≡≡<╝┘
<╝ <░▀Ω²□x°ÿ«â■o□∞└â□<Σ□□│ÇçRαí□x≡□□≈□□□αí□xx□xx□xx□x╪□<ⁿ□x8□<ⁿ

x□□-╬ï□□□┼a1□x╚□-n□-µ□□KÇ□/≡p≡p┐Æ□╦╓8-╥üçδÇç[üç█üç;üçò└├Zαa3≡≡=αa□x8

<|LεíFΓíôÇçi└â

x°□≡╨□<t□□δ╪o¼□□▀□-÷□□□üç_□□°ÇçOhï`ª¡B□])▄@∩□f□□╖□□w□□ì└C+≡╨□<<□<l□-╛□<Dü

ç├└├∩Çç≈ÇçÅÇçsΓ□Q#> :─'á┐∩ï7ëê%ΓçΓ□α┴

<┤□□□□□Å□□╧□□?□-÷□□?□-~¡Σ┴□èπ!□x╙□-n□-□ü□÷ìΦ═└├╖üç=└├»Çç╙d

□╔RÜ□<□□□5└C=≡□□-╛ <∞□-

□□o□□°Çç3┤Z0╨Ñ┬$z7°╣O°□⌡├□╖□-α□µIßδ└├S└├≤└├^αß
≡≡6≡p□x□í√E3□□»ªoêy0ó≤áç_□□ë>q▒┐J

□
k─.αßIαß%αa□≡≡Sαß□≡≡Gαß¼°g═$±/ÜΘΓêµV±#Mà8¬Y,~¼±└≥□`δ¬A□ ¼╓∞lWGg°AK
·í₧₧ßεεεaªΦ□▌□╕║□□□5□å╖╖αé□
ñ

G"├,
S□\æ╚╢.WüüRâ&┬/fªσ╧├□□5ÿ

□x□«'ƒ─<²²;v⌠÷n╪Ç⌡`ì`g╘CJ[□^m:l□&⌡twc¡u=□°dφ⌐3hëA7*╓□□╫┼3aç:;]«∞l½□=║╖½½Ö
ÖNKu·aC[ww¢NCu┌□kX□δ0░╖∞=3   tÅF"m□
1h≥£├NvüæN╫╓╙S□   ╙─Φ#æù□▓,rÅë▄Kôx▌ Jä7+è-
ÄDz·ó}}=□jt□¬3╜≥╙upaòrY╜v╕X½t╪s╞èNñ:=É£□□«
Dóy╓!╜åΦ5r√≥0'|▐╥¿╙□¥Vε▓╬Ht╞εHwd□╠kW├-°AJu╖a▄

║7▐6□□□BE°3óúT'F╪÷"□K>□#α⌠□═ÉI□[H╞╦ΘDò=░+□□E□≤┘┘>$-╗é¥□Ñ«□I□σ)□âUrÄ)□âü¢σ
σUW≈î≥æ□E╟ß)ú□½□□╦ïfÑ□4□1ê╬¿╙⌐□a╕å$τÉⁿα□÷?▌│┴_⌠╠e#□p┘┐█│ì╘`▐┘-
y□ε^╕□π□x╕~╠├!□î□«N╕\àΓ?╒├u▓ç□╡╘□--sqûP╫3╟□4─□.~1-
╟<æOur═╕ô□5╘□N╬╜▄H⌐qîª╧δµ,(Ä¬▄□π╨yq?╫}åƒδ╞²\□≤≤°╓²MG7
`□stpp╘c₧.╗║Qvu□┬qW□e▄╒1%µΩ▓┬]□öqW□e▄╒Ö2µΩr╤ΦΩZüÿ╨╒¥Zæÿ4Q0□ΓOp□ì─h4□;▄îü"
≥□

ùQGì□F≡(x╬(╟«FCa1v¿┐Éi╞╤.µë¥É╞F□Z□□ì╙≤░╤╙,7vvéÑbTÖÑ.ªî□M╘hë┬┤┤≤iτ&╝7└
nn4φ□·Θ╟╓¡{°ßç░□╪□⌠ #ñ□┬q·┴▒╗□╓X□□7δåÇ─□▒□□)3ΩëQ□╣╨/l_°|╣□░≤¼Σ"Φ8δ>ú┴@0
Å□½U»Ñz6□mα<∞Y^█║□&□5□4□Mx□T╜!¼╫Q=[ü╬F"□&
1i╟□─
 ûz}□□ï□□┤aF(bO?╞ÑL□□ÆêE<o□Å□ê□ôûÜXDu│@ΘΘ6Qj□°4ó7R╝σ√d□'□·╞å≡▓cìΩÆ½σ
∩√≈░£□¬τQâ╧,Φδ¼╓!□▄┌X╙≤░□╠□=dD░ÉÇÿ╨¢ê▐∞r║£╙#∞Né-É£

ë╒╓▌ª8S≡G□□L▒y□:□ô»áa▒á°T□¥a□□□(□αÖèTú□J□⌐I+┼□æäo╪â|AÆFCM║-╕°x≤HBìGÆ4╩╙

,0£πÜ∞□α5zGvvYY≈Yâ!µ╟□M▒┤│□û□"j¼åX@Ö5r@iêY;□%□≤°:δÉ╔HL□Pπ!⌡□°□z»₧ÜîΦñ,t╫Ü

á╬.Æ;X4¢⌐ª│¥□Γ□⌐l╘╟ó*é⌐hⁿΦâ□é▒╥ ÿCΘ╟┤│&35%Dδóu0╔⌠=&=&¡ç╗
n,à┼û□\Φç¼ArtÖM╘d₧═□□╗è╚ù ëÄ5□□¼½½□□╟óá╬╧╕2Θë╔0□aV¼σA~□□"-
□╞ÿü`╢▒(│rW╒t─<ìçÖYG═,╟.□gf╞:─┘XáΘ!m
s÷□¼▌□ÿ5r╔æf╔R3╥╚C═L⌐9Äσ□T¼▒┘╡ßΣ4ⁿ╧ê53□╠▒X╗┤`K□⌐9.╪XÉß½±hπßf╞pC╖ß[m╔U¡`2H
c□╟S5□\╝╬Ä⌐m0é|Ωsî┼□¬□╩╕╕╘▒ásÄUäò╡ΘEb╤≡z`□küpcV├▒g6yÖM─l▓└Yî▌W┴φî<□üÄ9#N│
₧Ü╡□cⁿÖ
áOv╦╜v║'3▌4║VÄ╘╬╡úΦ<g1á╨─lñf≤□R□q□áò<*τï╘Eª□╡ú░¼π±□9kNáµ QG╘╤ù▌ù▌S╔S╞&¬ç
□□:
Xj4╥□w□▟æ.╕;ß~0┬□îï,·½ L3H╜"Bï@╧ä□□3û#┤□[▀╔□-
╟30j═zbÄïQ+╔□╣α*éσ□9dî┴┐╚U╚"c╚□7ûé;H╣□▄^b╟é╦*v╟□═╡□□╕fò▓Ñ╢t□▀□│░àT-

Y┴x▄▓┴lî□ng□fù#W^½σ□o¼π ΓyS|∞JQïÄZ

q┴█eí╘□?(□âÖ□□□□□└⌐0v□ábc¼□T°□╖└<₧é1î¢l-├□╛┴b□ ╟□0□Ω£╬Q╣+□Xè\
t□÷▄å▒═╖Ö□,¼╓╣╬╣╬X(cQ╕╧äX6╟¢âw[¿`□█2)ó┘@□vö
¬p╓jç&ê╘┬┬9□╧≡$ß;|è┼3□┤E╧□□}Gεv¼τfc^u□wòs¿╖u┴`jXLÅδ□╘é□╛┼"╔b╖g╣\]τ!É1]Äj□
╙Ö~N╨─□{¼>ù

□╪    πüM□tcü-?│└╢ÿë┼£H□I:▐7Dnê╘EÇEÄ¡s□□╡ÿ╬

⌠ƒ8pα└└Yï^L!üHë╞▌u≡fü8▓X╬æp≥è╞]√#"τ: ▐9ªƒô_ú⌡öH└)⌡╖«ïóS"å'╛û▌
°ƒ│$Rïu(c(c╕≡p╬Q Q ┴è┴┴■
ol8`9`┴▓çó├╤├╤úp┬=≈δ╤╤²Q▐)∞ûòhíûä)Σ>▐Θ╪]╜ZBåH-÷·, ≡- ∞Y╓÷G░cà
╤ΦP[FóN7╪f1ï±╝cⁿ┬╓D/r╣#_&╚┐\╖ ≥H░1▒ÿ╬[îεê;2â░{2▄û▒w▄:▌ÜüüC+σôDû█Pÿ
`á &«[ù`╫≥[┘÷Ü90⌠/+L╨ü╓╒╒ì╓±╦┬╥Çí-Φê«ük░V┌±ôΣ+Aá
Üh&┼╪e╒£O╨µσÆ7~
%ΦiéæÑ

-□->:88┖3╞]F
5N8>⌠ç╝□┼ìgö▒·P+l┖τåBK\┌╔π¼

╢w<:□+æ¥T┌·┘(X6┤▒□çn╝w□X□/□╕`°□÷□éz┬εÖpg└mLä□╞}Ω□╦╖x┤Σ┐\8∞¿s╘┴₧╙h8╨╨PΦ(lh
8`╣x^□▄y$A□□Γ▄□ê╥èT╨B
ó□ÿ┤ìZ╞-a□▓□□4¬íZ▌░ò┼c-│è□A]]-╛─'~▒t-
î¢aÉ]Φ┼££□?□SA█╤ÿ╫¥╟□□□α□:¥┼æW7■BW¿â╖□□Φ╫ëαT)╨ï⌐ñüöéWƒÅΦ□╗╤z
c»â[│□τí0¥┼□στσì5í□█╤P□□åDmαel-╟1ë║!╓∞Φp∞□║2DΓ>;╦å □Å┐y9□╬
╔┴
┘sQ╨╜,G*□65τHsV□²9╥m▐û{□âÇA/<│o«τH□εp≤τ│╞□ÿDSj□╤╣!'ÖΩå£╘Q□9Θ¼σ`MÅ5é╡ÉΦ□k!
□XSZT#æ╣≤*ûH$<┐╥%æφ
j□□□9□-6▐z□┘ΘΣKΣvⁿÆAÇ¼'[╚7╓^≥□≥
≥∩Σ?╓çΣOöP-Z'├&=â\□[é□2ƒx╓}Σ□≥$┘N~

Ñ╝E▐!∩QJu▄╓□╖É□8+╟#╡°Ñ▒
d+·□yò╝A~I~K■H□¬G╥$béüⁿ"qÆJ▓□ >╙╫╔SñÅ□$G`□{-
V0□┌┘êÖL!╣░╫⌐"w□╙∞+)□╔6≥4·W≥S≥+≥;≥□8Ñ□□·╬∞-
É#ƒ□ôEΣ.╤J╓ÉG╔┐Éa2JM─JäΓ¬·□q╬»)f_□a╓□ü┘□└½└í\Σ-▓é¼é-
▌cΣY≥□▓¢╝F~F■7·=9M>í□n╧■>¢¥HΣ&2ç▄M|d%□δz╚

≥"·>┘G▌$oôw╓ƒ╓╟╘╞φ□ÿ□Sα░s3)!_!≈Æ6≥ ┘D₧#/æ└*2HÄÆ□Σ/Σ»4a¼□□ëH&ÉT≥□R□

^ Ç_

∞$▀
▀&▀%»É²ΣτΣ╫Σ?╙□9K□üCZ╛□╞╛ü95$ï\OÜ╙3\╖□B╝Öⁿ/¿_,»«.#sk¬µIdYm═φ□î6│I□++Σ₧Jn
╖É█╙bΓ&φΣ!≥5╥KvÆù╔□╔δΣ□9FNÆ " M'`-#╣û╞"ed
YF╛J║╙π╘è∩□Dïƒçeô□╔¡ñ£,┼»≡tÉçI7┘L₧'▀#{`□·7≥□≥□rå£úI╝□:ⁿDu□¼□àd.╣ôx╙
²d-YG₧ /É]ΣGñƒ□&╟╔)≥□9OôΘ□⌡7åΩ⌡°□ç□G□□□ó□╤èΦ¿w√├□ q1b□q'ΓaåF
b""┌□»F╟A£ëΦ¼wç╝╞╣ê╒ê
□∩B\å╪ê╪î□«»o□□█□7 nF∞C|□±U─7□Å"₧D□ah╨B¼E\å□@\π±√û¢╓#÷"nG▄ë┐
±U─╫□□"-B<Γini2□C□B<ë°>Γi─Q─s

═□DSâ»┘m╢"ª f ^ìÿ▌□t╫¢s□
□ï□+□□"╓!«@

#«±5√┬µn─ìê╜ê[□√|í□┐·9─□íßn≤.─□

-)êeêφêhcA□╦-─}ê²■µ╓&╦¢êç□▀F<Ä°.Γ)─□□G□?±╖╘√□□ó

╤éÿîΦ@ö□°"µ"µ#╬Bt6y=╛äR─□─Z─ÑêuPI0í□╤Å□DlC\â╪à╕-▒□qs
êämê╧ εD|   q7Γ^─(Γ□Γ`K╨╙£≡□ΓQ─w□∞²       ─≈□ç□╧ ₧eÿ(
□□□□φêiê□ΓT─□─□â⌡■pb□b□q
b□±T□□cB□b

b!b□Γ]ê~─`□bmB□b□q-ΓF─^─¡ê╧ä|═
□v"╛ä╕□q/b□q□q□±¡PS}`┬Q─w□▀G<├╨J□Mê÷P(∩□δd─,─Θêyê3□g#û"V".D╝□▒□≡Fk3b□▒□1é
╕□q□b/Γ6─□ê/"εF|□på╡-
±M─├êo#-G|□±=─╙êƒ0L□□Mê╔Ç·Iiê╙□g!╬E\è╕□1Ç╕□▒□▒□▒□q#αMI╜ê;□w#÷#-□£Öt□░
Θ}÷[[Iƒ0L&ê:D
b2ó□q2bV¿5□J₧Äÿç8□▒□▒□▒

▒□▒6┤*┖ÿ╝□q□b;Γz─¡a≡╓Σ□□_F|□±U─╫□□"-B<éx

q□±$Γ√ê°□G█≤fµ'ƒ□╝╔ª□£i3□▐l│□□╪R□o▒e□□┘«□,╢e□╬▒σ□║l·Ç%╢YÇÑ6g{▐═y╢R└□l□Ç7┌
j□g╪□□µ█ε□╝╔µ□£i[□x│-□X`[    xï¡□░╚╓
        Xlδ□£c█□Φ▓⌡□û╪╢□û┌·┌≤□≥l╧□▐`{□≡F█.└□╢=Ç·╢}Ç7┘·□g┌▐□╝┘v□░└÷6α-
╢πÇE╢w□ïm°□τ╪>□t┘F□Klƒ□û┌I{▐-yv□α
v
αì÷d└□v□`╛}2αM÷,└Ö÷ΘÇ7█≤□
∞3□o▒□□□┘ï□ïφeÇs∞òÇ.{-
`ë})`⌐╝«=»(╧▐□xâ▌╧v┌░ô│_åLâ=─]░7╗□÷[u°σµzⁿèq□~í┘□{╝{aO╒ä_P

└^6□╗ì' _r\╛XÄÿ}╟÷ƒaτ°╘÷^¼╡r[EΦe:∞╓ ╛'□{╢╧âz╪Åû┴╬n.£□*`ç╧╨Æ▀\ⁿΘB{-
∞7╡∞/%□├τ╥RαL±E8□\Å□╒Æ²e═|╪ü╧ä]u□∞[g┴~|□╬2|ß▄ßäô┬τ╖┐⌠ûhPôƒE╪╫nüô╨Ñ?Q8sìπö
0ß▓≡Ä╧@□╬□f8|ⁿπƒ(£≡>
½╟0□NI½αΣ|□╬□_à□┴²p.{□╬Y□┬╣µ!8A<

^²58%¼âSΦπ╚╙ +9(£□□N■□L╛,,F£âΦB,┴·Oα□w]Ph□qZ□□─□┤b_yÅiJ4]□&]□f^Çòƒë╓╥└ -
╓□□Y@□ÆEpÄ_□τ∩┐_°Σ║1d■t□jW]á┼▐□┼╗─╧à"Ö□≤╢π▓ƒR±ëÆ¡□Eü\M╛p□ÆÆmƒü□╓"╫\åñ<Ω.Ä
□√┐╟]å╘æG╚□≥u╥C-#¢■□:┼ƒr^□▓£□╓úÿ│≈á
Σ[ñÅ░╡≈6ⁿu»V≥╘ex□%ô>□■ú{AI╒gΓä╦┬zD□ó□▒□q9b#ó□q□Γ╝ê~─&─f─□─□Γ}êA─□b□▒□q%Γ*
─6─╒êφê_E∞@╝q
Γ□ê□─□□;□B∞B|□q-
Γ╫□╗□╫!«G|□q□Γ╫□7">è╪â°□Γª╕gÖ ╟□7#>ü╕□Q^¢σ┘A÷,9ª┐ë╕□±[ê}⌠ZíZ¿□□BDΦ□·äùà¿p
X□□åE"ZEI╠□¥b╡X'□─ê╪#÷ë/ïQ±░8$

kLÜ□MâfÑf¡f│µ9═+Ü□═█ÜôÜ3Z¥6E;U;S[¬]¿φ╥╜¿{U7¿;«{_wVo╤gΦs⌠à·□²]z┐╛├□4t□6□δî
φ╞⌡╞sªD╙dS«i╢┌╥tÅ┌┘┤╔□5□3╜g╓ÿφµ,s╛┘e«5{╟asùy╗·%≦>≤Q≤)≤¿┼`qXÜ-G-°□,   □
3□J□V$lL╪₧0ÿ0£hJ¼K|&qWΓδë╟□▀ƒ`¥P6a┘äαä═□₧¢≡è5┼:╥Zkm╢─▓╛c²0╓òT¢ΣI□'u
%⌡&╜ö┤/ΘPrZrMr$yO≥┴Σú╓°ÆGm□¢├÷ém»╜┬~ù}Ñ}¡}xóib┌─Θ□gM

N|ab ─#□OL□I╤ñ╪S▓R≥S\)╡)₧öpJWJo╩Äö▌)²)GRNñîñjRφ⌐Y⌐·⌐«╘┌TOj8╡+╡7uGΩε╘■╘#⌐'
RG□□ç▌æσ╚w╕□╡

Å#∞Φr⌠:v8v;·□G□'□#ô4ô∞ô▓&σOrM¬¥ΣÖ□₧╘5⌐w╥ÄI╗'⌡O:2Θ─ñæ4MÜ=-+-?═òV¢µI
°uÑ⌡ªφH█¥╓ƒv$φD┌H║&▌₧₧ò₧ƒεJ»M≈ñç╙╗╥{╙wñ∩N∩O?Æ~"}$Côa╧╚╝╚╧pe╘fx2┬□]□╝□;2vg
⌠g□╔8æ1Æ⌐╔┤gfeµg║2k3=Öß╟«╟▐╟□Ö╗3√3Åd₧╙□Ö¼Ölƒ£□g□µW□é;│⌠π*²ñJUΘgΓt
Φ°πt□!ÖP>ì╙┐0á╘│╣)≤_πWû?²░R┐«YY■□zDe ╟▀╨▀RΩ9┘*}στ╨í²7nWª╧╪¼╘○MTΘ╓8□÷+╖&½╥
+P□╪ ≈UεaßAY~i1ªh≡≤£╓░ΓΓ█┘£ç/╫╙╥╣MûE-
^╠z╬ì\6r╣¥Kò□╕Æò╜tàòzY₧J□*≤ù⌡)⌡╣=J²÷vò▐í╥?TΘ├*²┤J-
QΩ□¼²t╝}□┼*}.Ω*v**╣∞ß▓ƒ╥w╤:□╬Ωep₧\L╪ ╖4□+Q□Vï-ÿ╤√≡│¬=$J

Æ├Σ(□"°≡3?B
╘J□TΓσDe9╧/╤╨4.□╦▓¬[û╒&._òσ□\┐c)ù▄εÄô▓¼IΣ▓ü╤S▓£?ôK□ù¢dYδΓ▓ù╤╙▓\P═σ□Y.¼π≥}
Y.ΩôσΓà▓\┬=t     ○ ╥
╩Q╕S┼·¥│U·\ò▌¼╥╥─ΘP▄▌y╨⌠╖□T·JÑεW╒∩?í╘¢∩QΘ]q:┤┐y□Ω

2ô╠&Ñp≥[□gí□╪╜¼ä□╞Z╪□låòÜ}Æ·□┘G□╚!≥6yç£$╨¡mµú╒ⁿÄ,["▓

dq·ª,∩□╕\╟σ╥▓

≥Q□NτÆ╟lp'ù∩╓2─G!4ïK^Oh@ûaε□a>ja>èa₧┐ò╫╙╜██┌«d╜⌡u%K¡C¿█Iε%≥"ù┬{╖r.ù╧╙r╒╒\
╢s·ë,█°|╓÷û,Ws□V┐
╤v╬Z{'ù╟d·U▌ï»εÆe□ƒ±:╓╤≥~ █√ùq)╟v▄|╖&Å╤m╨~»9ì║Öd└□*çΣôBΓ"□ñ□╬╙-┬#z
Åñ□Ä╨2▓Oû□«öeg.ù<rΓ≤o□¢ç·X=╠Y[╖□Ω4Ä{Σ╫rUz╛Jƒ⌐╥□+√á╨a⌡Θ

(φ╗σ·DⁿÄAå▄ånεO▌/qy□mTsτ:>>δ8 δ°°«{_═∩z╬┴·╗ö~╢~+û□o·«,-
▒*τòG□T║èÑG÷⌐⌠~Ñ╛Q┼╥ú∩¬⌠wΓt`φ▒⌡D/(·≡╪!YnZ□g╔⌠□*}¡:τ&╬╦7□öu|πá2goÜJÅ(┘Ω}□K
6≡1□ÅE9ò╧9╜gd·8≈½═:Y>A╕tp╔}~╦█Xïëñæ½╔tr#ÖEè╔\RCû□-/[·Z≥□o╟S╧╔r¢Sû▀

╨r;ƒ¡╣║≤H°□Å╧>n╫╫¡-φ>>/⌡ì¿S₧µ>÷t°ÆôgG½┘}å≈{Gç╥≥9√à~²▄█▓ⁿvù,w□^h│ô╟Θ≤£┐ττbì
cσ>┐Γb▒≡<_/ □òσ┐≡9φ;║
k°╬YY╛T┴σ□╝╜┤¢╦╙ƒ5Γ▀σ#°]╛╞┐╠y■-▀□∞ΓQ╖ï╧â▀τk²□,D/▓°Σ╕1╟öW÷p╔ú≡ç|å■!∩═₧□Yεò
ö\∩¡VΘ~uÅ÷≥}═Å²J╦□r!?»≥╤·W
ù╖╤C╡°¡!÷□▒x┐≈±╒s∩▀k|/⌠□▀+╜╢S=□╜╞√≈┌¿2·ú≥.C╤è(ƒ▌ó|-Å

*τ╒²3ò╤╝□'Äòq¼ì□c{G╠┐□Hû»≤╡ⁿ@¢,√
╕Σ▄▀ä∩Σ□^öσ□□Y-äSïF╒╞âⁿ£≡╙░,▀ö╚╝┐·:ùg╘)?πQ²│□1σS█²│π▓□Σ≤╞ ○≈α^.·|≤s╛Ω■|-
ù╜µƒƒSÄ°íló^ë╜~èÅ-
¬σÆ╟╫┐±╣⌠0≈╪├├▓ⁿ┼□Y╛U,╥_╢)k9▓OÑƒPΩ┐□Tzârt□⌡Æ·îvtç,□╜é²B╠x╬c

σ¼}¼□²BÅú6╛ïÉ╙°╬Φ□_√□├= 8O?╬}∩▀y¥∩H□╣□D┬o·iπ╖|╞─▓p╔gΩ▀¥╗╪£⌡{. âÅ∞-
°╙¥≥`ï/¿σ□_□ ô»≈∩±òσ=╛ú·π╘8╬`°±╟□ö·_ª+⌡æMDÅ╧\?□□□à≥■╖]□Ñτ«Ff□/▓N¡ A╥N:
ƒ
╬±╜╒9╛S<╟O=τ·îr■..σ□*√─Lû=\╨1Hi#ù≥iç□s╣öcôèyè□R▒≈□[H┼â\╨QO5:.Wr)sM╡
à\╨s<╒ \.σR^c⌐╛ÿKy₧Ñå╓\>C⌠║qoñF¥jNñFniT«ƒ╘⌡o∞
¿q□ù≥⌐éÜ¼\F╕ög[jnαR₧I¿Ñ¥KyFñ ╜Ω6%r6□□J~□'□╜>«7ëà¬⌠Z╘╒₧K□·╕%vq□σ≥£,'╟╡x╛
□._αR▐□Pk□ù-┼¼□cc▌&╦ñ□□½□MR■tâ&¢Γ~F╞⌠□ò~De ûJ?⌠7⌠Aò■&ë □□¥8á8≈╥ö4ò₧
ó╥ò?]í)ë*=Kq╨á)9*}╗RO═RÄ\·nÑ₧aW┌gäòzf»b.íôw╟φ╥á=ô□UΘ∩*⌡)eq÷pO╛¼¼□╨□┼ⁿO°⌠*
·ÿ▓Mò□ïJW█≈(uiôJ-
R╓/╜½╥▀QΩWe─╒□²╡j¬*}║J╧W÷ ΩRò▐í╘|ª+╤╕fï▓╜k▀RΩ╛w⌐⌠ôJ}Z≥àz<_╙┌*⌡Θ.\Θ╞⌡□╨ⁿ╙?
TΘ╨□□²óñ╘s5*²ö╥ƒ«TΘ╟T·iò~F⌐τσ(╟3oû*}ªÆ╧<╕Rƒæ°╘o□QΩ□{ö·¡□╕□□╙g;òzæF⌐╧9¿□▀╥
*}╗R/½UΩσ:ò^°╥□ö·φ*□¬ΦSΘo+⌡y╒J╜RσƒUw⌐⌠M*}|J▀íΣ┐j°R»±+⌡□y╨ⁿïFö·b□_ïO)⌡%□Iⁿ
O╟ΘÆτö·▐µ╕]"ΣHƒ⌐≡□!▌»≡□!╜QÑ7(Γ□⌡8□□╥√□■!ñ∩SΩô;ò·UÑJ═╤ñ╘°-R┤_ÿ╢I┴ù≡╡QÑ╛╬á╘
7)τoß)I⌐∩P«□┬w□ö·+.Ñ■Z╡RÅ╓)⌡ƒ*╟Sx½M⌐ ªK⌐ƒΦQΩ-
lï█'┴¢3±■□²?≤£Æ∩3╗TΘ/⌐⌠╜*]yz□>ÆT║¬?-
φÄ█Θ└¢Åó╨·?z]Ñ∩#∞╗~4ñ₧>N□Φ6,u□φ╒Z╡IÉÆLtû≥─□KYb□b4□Of⌠□:LO╙ J,J£â·

VÄ≡?∞]□ö□E╓«¬[▌⌡^w┐Ωz□├0 *□Æ┃# """YÆê#

A@@D└a@$ë$□ÉEr□□I□Äê$□FΓ□$I□r╬9H

├□╗ ª
ª]╫u≈¼ ┘≤╣╠╣▀╝~□εw½·~╥½½{╥1∩·$S∩òB□m□-├½ ╝╫Æ▐□⌡┼π2┴l ªX□°╝≤ó▐'α∩5ë7┌x
□l╕3V╝ⁿ▐]□~o£□╚D2ì╠DL$│╓□─·$ë,G\JVæ⌡N
┬£□NM┤□¥Zh+╓π□ÿB∩OhLû'□3α≈S□ôe□a°m;┌d╣□φ*╣ƒ□·╡\-╨ÿ,□".┼∩ç4&╦├■∩Gⁿ▀Å·┐-
K√▌⌐¼};ó};Ω·αT╙K^╥K¬k»╓Φú«╒G]»Å║Q/┘ñùl±û 'o

□5Nδ≤ëÉO▒=«c,zαWù‖ñ□ìó□ôε4□═Ez╥□ZÅ⌠ó¡Φ[ñ/}¢╛M·╤╢4Ä|D√╤~Σc:éÄ$âΦ□zü

ªWΦ□≥
╝IoÆ!▌─;2öÖ╠$├ÿ├□2£àYÿî`□,éîdÖYf2è=╬-'úYnû¢îaàXu2û┼▒÷$ëu`□╙b╓ëu"KX□
╓ò,e=YO▓£⌡a}╙□6ä
!╔l8□NV▓ël□Y□!ÉΣ□□âb$□╩B9r□*BE╩`,îÑ└π°°ö□»□»╙"FSú)-j╝a╝Aï□-
î□⌠Iπ□π□□m┤7┌╙°î

F□Z▄╪l÷Ñ%¼ZV#z╬ΩcSÜΩ(τ□÷«≤Üô╘╛

5 ╡d?å║à·│□Æ╔□□Σc≥1pσπ≥qP2ç╠□a·ä|□╥╔▄27<$≤╜╝É^µù·!â,(
Bä,,
CFYL□âH□-ú!ô,.ïCö,)KBfYJûé,▓┤,
□╥2▓

dòeeYxDûôσαQYAVÇ╟d¼îàl▓ël□Å╦f▓□dù═es╚!▀ÆoAN·╢|□₧Éme[╚%█╦÷É[vÉ□ Å|W╛
ye7┘
≥╔≈σ√É_÷Æ╝áÇ∞+√BA┘O÷âBrÇ□□àσ╟≥c("□╦┴PT

æCáÿ□&ç┴ôrä□□╤rö□□O╔1r

□ù 2□J╚±r<öö□Σ□xZNö□íö£,'├3r¬£□Ñσ49
₧ò3Σ

(#□e"<'┐Æ_AY·╡ⁿ□₧ùsΣ□('τ╔y≡éⁿF~□σσ╖≥[xQ&╔$¿ ù╙%PQ.ô╥áÆ\!W@e╣R«ä*r╡\
Uσ≈≥{¿&╫╔u≡Æ■ 7@u·âⁿ□j╚═r3╘ö[σV¿%╖╔mP[εÉ;αe╣Kεé:rƒ■□»╙3≥

╘òτσyxU^ö□!F^Æùα5yE^àzn67□─║·▌ⁿ╨└─Φ□äåna╖04rï║OBc╖ö√

4u╦║eß
╖£[

Ü╖σ▌≥╨┬¡αVÇûn%╖□╝ΘVs½A+╖║[□▐rk║5í╡[█¡
○╖u▄:╨╞¡δ╓à╢║nî□□φ▄zn=x╟ìuc!╬mΦ6ä÷nc╖1─╖M▄&╨┴mµ6âÄns╖9╜δ╢t[B'╖ò█□▐s[╖¡í│█╞
m□]▄vn;ΦΩ╠╣q╨═ìwπí╖█╤φ□∩╖¥▄N╨├φ∞vå□▄«nWΦΘvw╖C/╖ç█□z╖═▌₧╨╟φφ÷å╛.~αC╖ƒ█□·╣C
▌í≡æ;▄□

²▌æεH□αÄq╟└@7┴MÇÅ▌±εx□ΣNp'└▀▄ëεD□∞Nv'├'εTw*

qg╕3`¿¢Φ&┬0wª;□å╗│▄Y0┬¥φ╬åæε\w.îrτ╗≤a┤╗└]□c▄àεB□δ&╣IÉα.vW└8wì╗-&)¬(LSá□ª+
□

ÿíä□≡à□¬
$*[┘≡Ñ□⌐□╠T«rß+□VaÿÑ-R□┴╫*â╩□│UFò□µ¿L*□╠UÖUfÿ°û½σ0_%½d°F¡R½`üZú╓└╗j¡Z

┍z╡-□⌐ìj#$⌐Mj□|°╢¿-
░XÑ¿□Xó╢½φ░TφT;aÖ┌¡v├r╡Wφà□j┐┌□╓Ωá:□+┍au□V⌐úΩ(¼V╟┍qXúN¬ô≡╜:¡N├ZuV¥àuΩ╜:□δ
├<╠aC╪

¢░1□□□αç░□╢`S╪ ;░9,├□╢ !□[├Θ┬Θ %£>£-╢à#┬□░=□□Ää□ß¿p□∞

g
gü]ß¼ß¼░;£#£□÷äƒ□?□{├╣├y`_Tµ¿2p□≤{#T╒╥⌠□╝├î4}dQ,□╥□□î╞F□úÖ╤▄hg─□±≥Q
ÖMfù9e.ÖGµô□d!YT>)ƒÆ%Σ╙≥□·¼°╧≥EY_╛.¢╧7d+┘Z╢æq2^vö]ew┘S÷æ╨■ráⁿ¢ⁿD

ò├σH9ZÄòπΣ°≥39INæƒ╥Θ≥
9S╬Æ│σ\9_.Éï<òFmJFòZ#╫╨⌡rú▄$╖╙□╣]εö{σiyN^É?╩╗■□noDπQ¡X▐ÿ½Aδ╙ª|₧y;P·∩░¼°ùd
□ÑY~µ╒?┤à╣δ       ?o□║ü▄jc╒┤èz%□
╛□⌠fTfí┘h-Zäûñei%Z□kÄ╞┤%mG;╤-Xg

ªúΦ□:ì╬ó
ΦR║ånó;ΘAz£₧Ñùp╦┼t□▌H╖╙²╕Σ<╝å╗
`|"┘ú,□V□┼Y□VüUguYC╓£╡a□Yw╓¢⌡gâI□□╦Ü▒╓,₧u┼%□┘0û└&│D6ù%▒d╢₧Ñ░╜∞(;╦«░T0AB□d
à∞É□□ßû╖Çâ□Θ!
.╓çòG)(□Uá6╘â&╨□Γá3⌠ä■0□╞└Dÿ□│a!,àU░₧düQ0□ª┴,XÇK╓└&╪
□ß$\ä□£qï°πQ<□╧├ï≡Æ╝,»─k≥□▐É7π¡é□ `¼Γé∩jl∞ñ1>°₧╞

┴.êq°WWìq┴n□█□╖kî□╛»▒C≡□─÷╕^Oìq┴^□█□{kî□÷╤╪!°!b<«╫Oc\≡#ìφâ²5╞□□h∞□ⁿ□▒□«7H
c\≡o□█□□kî□~ó▒C░3a°k□┤φâ}╤╞□□óφ□□¬²□'8╠τ7▄τ7┬τ7╥τ7╧τ7┌τ5╞τ5╓τ5╬τ5▐τ⌡⌐╧ké╧
δ3ƒ╫$ƒ╫dƒ╫□ƒ╫Tƒ╫τ>»Θ>»□>»/|^ë>»/}^CÉ┼;┴□═kóµ5M≤ ·╨τ5╦τ⌠╡╧k╢╧kÄ╧k₧╧k╛▀n▀°ⁿ□
°ⁿ╛⌡·-⌠·-≥·%·╝╛≤y-±y-⌡y-≤y-
≈y¡≡y¡⌠y¡≥y¡÷y¡±y}∩≤Ü⌐y═╒¡╣X≤J╓╝╓·╝╓√╝6°╝6·╝~≡ym÷ym±ym⌡yÑ°╝╢·╝v°╝v·╝v·φ╢█
τ╖╟τ╖╫τ╖╧τ╖▀τw└τu╚τu╪τu─τu└τu╟τ╡V≤┌ñym╫¡vP≤:ß≤:Θ≤:σ≤:φ≤:π≤:τ≤:∩≤║α≤║Φ≤·╤τ
u┘τu┼τu╒τu═τu▌τu╙τu╦τu█τòΩ╖█¥4~▐□{≡°Y4ìƒ┼╥°Yα≤;«y¥╒╝.i^7╝V├□[ùΣª¢╪8╟O/A3x
□Z┬¢≡

┤ Ç

≡.t□¼√á|ä┘j□^/□äCp□Ä└Q8□╟ß□fªSp□╬└Y8□τß□µ⌐ß□\

y≈≤s╙ì,□*C5╠yMí9┤Çvÿ·Γí#f┐«╨
║├√╨□>Ç╤ÿδ┘┼

╕□Æα;X

K0≤-âσ░□Æa%f┼╒ÿ□┐ ç╡░.⌠ñ╛╫/ΓΘ□-⌡2! ·J5Æ▄}ΓïÉëz

áò╖□╧╩ƒ≡╫≡□3ù┐ ╞$ⁿ
0□╖"ä°τ□πz╢x.b?░àwî\□□├{□┼┼╟√□7╕╔□□≡ fb¢;<─%w╢Γ▐x□≈8ßß╝m□□åù&

□Ä?G$■V£dé╓0□3²ù┐╞δ╫"ÿΘ[▄N└$ÿä√£□S╨¢Θ0¥0H□╟0□▒ex─âpè╚L
2-└□0□M"q²)╕^Γ»F⌠W┌

╖2╤│╔xî⌐0□Å1□fα1╛□╠^ΦΘ:<å╟G╧+╣││]╟ΣáÄ╔²□-≡▐⌡Nÿ╤Ü═■┼╙}i╧╔▄}╔c0·ä

!C╓02\?τ9èî&c╙XÆáƒb·□k╢╧░²&æ╓d□ÖJ>'╙╙t2â|A□╓ùd&·è╟"_ô┘d

ÖKµæ·Σ□▓Ç|K□ÆE$ë|G□ô%d)YFûô□$Ö¼$½╚j▓å|O╓Æud=┘@6Æ-
╚&▓Öl![I□┘F╢ô□d'┘Evô=d/┘G÷ô□Σ 9D

```
ô#Σ(9FÄô□Σ$9ENô3▌;~╙yrü\$?ÆKΣ2╣B«ÆkΣ:╣AnÆ[Σ6I%w░╙QVâ┍d╡Xm÷2½├^┴zΓU□├^c⌡X}
¼#□`u╤ê5f»│&¼)V□o`¡╤é╡do▓V∞-¼1▐╞╧ú-
□╧╢│□l'█┼v│=X]∞c√┘□vÉ□bç┘□¼5Ä▒π∞□;╔N▒╙`▒3∞,╪∞□;╧.░ï∞Gvë]╞ZΣ*╗╞«│□∞&╗┼n│Tv
□□□□□Ç□ëüuèÇ□□í□┖äZ║-⌐□
í□╝□m▒"Θ□╜í□|□#▒.Ö    _┴╫XÖ╟ço`=lÇì≡□V#¢a
là□╪□█a□╓&╗`7∞ü╜░□÷├□■4/┼7±═|
▀╩S°6╛¥∩α;·.╛¢∩ß{·>╛ƒα□·!~ÿ-
ßG·1~£ƒα'·)~Üƒßg·9~₧_α□·Åⁿ□┐╟»≡½ⁿ□┐╬o≡¢ⁿ□┐═S·□#dñ□╧ë▓ΓyQN╝
╩ï□E□QQT□òE□QUT□/ëΩóå¿)jë┌ΓeQG╝"ΩèWEîxM╘□⌡E¼h
□èFó▒x□?M±≤□~ZêûΓM╤J╝%Zï╖E□╤V┤□∩ê8╤^─ï
```

óúxWt┬Og╤Et□▌ Dw±┘Φ!>□=E/╤[⌠□}┼çóf°H⌠□□─@±▒□$■&□ïO─□1T

□├┼□1RÎ□ú┼□1V$êqb╝°TL□ƒëëb‖ÿ!╛□ëΓK1S|%fë»┼l1G╠□≤─|±ìX ╛□
┼"æ$╛□ï┼□▒T,□╦┼□æ,VèUb╡X#╛□k┼:▒^l□□┼□bô╪,╢ê¡"El□█┼

▒S∞□╖┼-▒W∞□√┼□qP□□ç┼□qT□□╟┼ qR£□°┼□qV£□τ┼□qQ\□╫┼
qS▄□╖E¬╕□
□*&ë╔bèÿ*>□╙─ÅΓÆ╕,«ê½VGδ]½ô⌡₧╒┘Ωbu╡║Y▌¡≈¡-╓□VO½ù╒█~╧εlw▒╖┌▌∞ε÷√v□√□╖°▌█εc
≈╡?┤√┘┘²φ□÷@√c{É=╩-mÅ▒╟┌
÷8{╝²⌐=┴■╟₧hO▓'█S∞⌐÷τ÷4{║²à¥h□i╧┤┐▓g┘_█│φ9÷w÷b{ë╜╘^f/╖W╪╔÷□√{{¥╜▐▌`
○┤□░7┘¢φ-÷V;┼▐n░□┘G∞c÷ √ö}╬╡ ` h_▓/█W∞½÷5√║}├╛i▀▓Sφ;

q¿├□p╕c8ªs╙9∞□qÄ:╟£π╬  τñs╧9φ£q╬:τ£≤╬□τó≤ús╓╢∞\q«:╫£δ╬
τªs╥╢φñ:wB$DC,□!-2BfHä□í`╚□┘!'□□╓É□Ríp(]ΦíP·PåPD(c(2ö)□□╨□╨□z8ö5⌠HΦ╤╨cílí
╟C┘C9B9CúCcBcC
íqí±íOC□Bƒà&å&à&çªäªb▐},m4VÅèvcπ□fP=╓·)Té╨d+Tàjd□─└kd□─B□▓K+▐-h□m╚^
T¬≈╓>□

â╓!□□#╙a┖α╤ΣêV¢úZmÄi╡9«┌µ□╟àyΣñ╬ⁿ°y ^Æ□=f╨

╥░h!C□è□╓úóE╠□µQz\□□┼ΦY=B·ú╒╟□═ÿ5╓·Äe┤V[╫X□=N┌XÅÉN╓Ω□$□P╙úI□RÄ╘±g₧P□╘╫fO│
≥¼"{╓·─Ügy≤ª╝YFσæ{□²□╛╜□%uH□+ÿX╥□╖jπÅ▀{╧Γ□ΘH║ó-□~∩ï□□¿Eâ╓□╕□ar╡=C█òh╖┘½╨ε
░Wú▌e»╒╤╖í]b∩╨v□┌∩φ=h╫┘√╨n░╧xδ╚

▐║2┬█Vf╘█▐╔╦âhWH
φJiδ%«^ó⌠Æ░^□⌐ùd╥Kó╝%¿ƒ╙╪ª□ô~■T= □kτ□F==}ⁿ╝Ü°∩╞├tóëh&Üï≈P]<U|□⌡░èV¬□¿:□╡□
╓E²≤ö/M≈Ü■N┼δⁿ□┤ εùJ7□5ε╛║=¿□ mJwO╓P≤Fá6?¿x╧aeß╒□iUàWST╟¬Γ║_S▄─èΓU¼&□t=1

½ë□╪kδ`Omα⌡╥╖║╚▌·⌐&:╩
;Θ£ç£⌠N□'┬╓ΦD:Ö£('│ô┼y╪╓Ω<Γ<Ω<µds-w▓;9££╬□N.'╖ôτWò┤╫»k⌐

JK ⊓ KQg$^{n}$RSÑ+ò

BYW┌½∞╒Z_╫■¬┬nCì▌a∩▓≈╪√εj¡îÉ□╡▐₧·M┼╜²K═òæ2ôî·C╧√□▌un □╩½û²□φ}ë2Ü□/üóh.Æ₧
V°╡╔π·-h.□K¢Æ╝⌠
·□)J[╨□ñ□}ô╛E₧ño╙Nñ8φLçÆrt□□KbΘ□║ü4fφX□Θ┬ΓY□╥¥uc∩ô╛∞□╓ç|─>d□╚
÷1□LåΩ╗Ö#┘0å·₧%░□Æ□

ñ#π =ñ'ô!□≥É)É□□ÆàP□╨æ┼Z╧╖h=▀¬»=R°□╛ü£4┬FÿF□Wî+4ôq╕┐Fúî□╞
Ü┘─Ç╤,µçµ□·░·▒·   =f

5G╨'╠QµXÜ╫□gNú□═□µl ·┤ 9╫Lª σ╠UµF ·▓Öbª╨Xsç╣ï60≈ÿ√hcT▄█┤⌐y□ò┐çê□O╙·Γ□±,M□Σ

ΣíK□·□□Θ▓@ß@a║2□□êª½□%□%ΦΩ@Θ@i║&P&På~(□(K╫□╩□╩╙uüèüèt}áJá□▌□¿□¿M7□^
╝B□□─□bΦª@â└δtsáEá□▌──ïq║├jl╜NwZM¡µt╖╒╥èú√¡x+₧₧┬║a4=ì╡├w⌠▓u▐║FSmf┐╞
ä]▀ε─□9π£â¼[¿r¿2[□□□□┼ûδyæ

»8□⌡╙z}┌╟_2≈ü%▐xéI▓ß⌡»7≤█ƒ□M≤╥□┤8}æVª#p²Iz£a□n□·∩D²mæ m□~█â-
åZ█╙ê22□Yîçì¼╞#╞ú╞cF6πq#╗æ├╙i<aΣ2r□yî╝F>#┐Q└(h□2□□Eîót3▌B╖╥□║ìn°;ΦN║ïεª{Φ
^║Åε°□ΦAzê-ªGΦQzî-°'ΦIzè₧µ└9\ü½p
«├
╕    ╖α6ñ┬¥□e□'£ro~o#¼╔┬□ùHÆE┐e#-
)HJblJδ□]ZÖ╜ó▀öΓUi═⌡¢*╜w□u├≤╙Ñ□╧ú¼4□₧G1□▌□┤ò■ÅÄ±┤
²É~D√╙Ax╞╟íïΘ□ÜLW╥╡zΣΦwÄ□²▐╤*o┤å?woñhÉ?RΣU°╧░┬,Üòcò░>y□½Å7¡╛╓çV?½┐5└□h}l
▓&[S¼╧¡i╓tkåòbm│vX;¡]╓nkƒ╡▀:`□┤

• Y n ~

≈r⌠▌

φe╘╖·╘╦ú≈»X╬Φ╡╢-
O┃z≈ⁿJ≥²Üé╢╢÷½□zV]{□â╛╕²╗╛%Z_Z3¡Y╓╫╓lkÄ5≈'╛-┃Ä['¼S╓iδîu╓:≈w=NSö□⌡ΣA

i·■ül»YTd-²{W%}.äxÖ╘─□┐-!▐ò□)δ]MÆ□▐î□R╥~╧yü¼GU{ÿÄ─⌡□ █┬x╡□═ó
E╓%pfÑX)□╚┐□ß╪:òêüæ¿Ä{¼╓j□ü□yà□0*1x□Qƒ╒'□╞°)▒f|ô8X-
╢%!δUδU"±□╢+q±*e0Q╓□k4╓é╫*sIvk╛⌡□ë╞+ûsñ┤u┴║FÜó-≈!oí≥

"¥PigÉ-¿ús╙P╘═□d,╢▐-2[p·□╒≡□YÇmyê|ïφyä,─6=F□a╗₧ I╪╢°╙w¿Ég╙bT╓sd
*σ□▓□╒≥6Yå┌hÆ⌡X/DÆ□¼□-#{▒.╙CÄb,lr□ò:L.á^Fa5}╖_ö⌠√┼ "·»G╘ïdYîOz

$\delta T = \frac{1}{2}$

■□┘□9▓wcZ÷^o² d ╝>¢δ^^²■╗┐í╟à╥" ·ÿÄe!o⌐5╞ÜêK□[½H╨║n{[ⁿⁿε╚?╗╡╖Uí□╬ù╧∩╡Ω?│ù╗
█□║τ┼?┐□åy0ΩÅ╙E▐lè?Rëαv

·□AF□oÇ[Är
)CvÆâΣñ~□>ú□Mç{²²s□V╤⌡╚`'z{ö₧ª□)V¼î3ï)□┴▓x≤9X□VîòdeXyV□{y]□╥Ü░û¼
^Etf=X_6ÉΣfΘ⌠▄Å<¼□«YV╫.1¼1«╒Äu┬u·▒┴l□¢└ª▒Yl>Kb╦┘□╢æÑ░▌∞ ;╬╫▓K∞□□0╝δ

êL¢╙Çτ█nÜGsti~ì1┤╕╞·┤ä╞XZRc□·┤╞å┤ö╞F⌠□ìiiiì»╙g56íe46Ñ╧ilF╥ilA╥klE+k|¢╛ö□[
#úç|«□⌐q₧æ╔Cy3`{h<□p<4'□B□□□ñ╞ñÇ½±v@iL
ä5▌ ñ≤□»@-╥°¼¢╓å-
inΓ⌡å╝hs╤|hch□]·□D□K▒ !║┬h□╥"h□╤óh□╙bh_°OómBú╤6ÑOímF╥z3tΦ≤h▀ñ/ámE_$▐ p«Ç╢
¡ê╢-¡ä╢□¡év□¡èv

¡åv┤æ₧0Σ¢□φ<├□i╝□`ä!S<3É'G╖(`áM□ÿho□□┌╘@□φ¥@É0Σå╫.ügΘ4dµ²°ΦΘ□δ╙□□cΘ□□□╨Dì
Θù□□╙Ö□_°_ilDgilB┐╓╪ö╬╓╪,≡░çΦOV□╤úG4.□<¬1)≡ÿ╞█ül□S□Åk╝□╚ε!zùCπ│4A╟z£Ä≥x□σ
Ouö'Φ(□ªπ;Q╟wÆÄ°d□σ):╧Suö?≈b□H»úôAG'BG'úÄNñÄN&□¥(□¥╠::Y╝Φ╕Dg□o&║≈▄`NΓ=E═1
□d@o□ß□εσ&oVYV□vY6□í{EF▌Üæ║╒2i█\╥⌡0∩oB⌡uc:²▄3ïz-¬⌐~û⌡ε╛▓D╡┼Ñ⌡ú┌í¡□⌡

┌ÜQφ╤╓Äè≤ûπ-"╓ç⌠e·□}ò╓Ñuhs½.*UL┌□□╓₧u┼3}(îé╧aû╝%o╦Ty□sΓX+┴□gì╖>╡&XƒY□1?.▒
ûZ╦¼σ╓□+┘Zi¡ÆW%ô ╣4ñ)à

X╫¡□╓Mδûu█J╡ε╪─ª÷▀∞┴÷'÷□{¿=╟-nÅ░G┌sφy÷|√□{ü²¡╝╨^d'┘;φ▌÷^{┐}╨>l-
╡Å█'φ╙÷Y√╝}╤□N└
:ûc;Ä□rñπ:y¥|N~°ÇS╨)Σ□vè8E¥b╬ôN┤≤öS▄)ßöt₧vJ9╧8Ñ¥g¥2╬sNYτy°£≤étdHJÖN
>$╙╦k≥║╝!3╦,≥aîhN=ΓB⌠(ïüW□òQφ[▓V¿≡q,

U╝
δBBz&╕╘c'«-□Q·«F□╛é»H:≤Ks&y╙£g╬#□╠½µU□ßì□Éî▐8□y╘┌k□!╣╝╤□R╪εï:_╥■□□■y{□*|□
{ù╝ïT╒:_Mδⁿ KZτ½k¥»íu╛ª╓·ZZτkk¥□Yδ|□¡≤»h¥»k°ó┬┐Ω(T⌡╞Z╒╗hU∩.3á¬□Ç<□Éÿ▀╙ó□¼□
-
φt╖à,□Móú□╘qL°πÿY╟1╗f₧_3Å╓╟khµ╡u=≤J┌xïa□!²TI%▓□mYÆ⌡┴■ ≤^ⁿ█²1¡∩xτ.÷öJ¼r┌]□
lß{Wτ╓aδ╙╧G1∩Å`bkDxww┤O╢!∩·t≈y□Ä╝«
÷╢!■:Äß>αw$ ▐?ç∩₧° ▐╜-
∞├5░□¿Ek{}□{ΓU\╗□╓≡{∩*¿e▐□7is/╛±+╟+i╧╒x-Rσ╜□ï½%ä⌐6┐║□B┼≡ù┤Ω¡▄╜╧╤{b8ú╨«r½□
ΩI□¡₧R┼U UR=ú₧Wσ╘
¬╛zQUP□U%UYUS╡╘k¬ü▐kG□O╤ÜC·{√r~u_╕¥▐¬╧O╢}□╖ìz`[¬╨ⁿ«╡≥ l-F:Θæ║⌐·)
o╜Θ╕L□A#HêjΦE@5VM12o¿û─T¡╘[─Ro½║Φφ;Ω□Γ¬÷Ω]ó╘{Ω=ÆAuQ▌Häz_⌡ ÖTO╒ôdV╜U_ÆE
P├╙#jöÜHr½╓□╧b▐\□d[□ÅQMU├Ñ5U-
ÆG╜¼^&·Tî¬O≥½□□¡┬¬┐ΩOèho≤iδ╡□É╬°±▐°5═ƒ═⌠W±┌{¢▄_┼kåδ╜|╧{N║Γg□±▐□Γ╜□!Ω/µ┐≈f
X∩
▓╘?□<╧╞╛τ₧╧T√lhƒâ┌g[√,╡╧«÷9╜÷9â÷9R√□Ñ}╬¼>R-
æ¼≈< L{>¥□U á %-Φ□U⌠□╫¥x∩■Iⁿ╓□≈ƒ≥σÅ÷α*:åòt■cñ·□₧ ╤²ÑσW╨Y▀√V₧xoìÅⁿ╙÷¢V□Γ╡□
╓°àHv□┐?_|µ╨<ÅQ╫▐DΘ≈╥T└┐cHCR√O±ττG╣∩[v⌠.δo°÷δ^y|8¬²IQ║∩╧□┘Q⌡OεcUΦ
┘Φ
┐□∩¬┐ê╨▀∩AU□ú□²□╧∩¬□·Öx?·L?ír £·sz∩╧[δ╧>s□╗u╜w(u·□╫K≈²¬½m□]Q⌠%═╙@Γ╜%²?[Q▄
≈ªµ/|J¢=÷▀σôWy═%□ò╨δA▀∩╓]^▌°╫¿╗~ε²|⌠>Θ/Σ²╧{Åüg|w¼{gô┼~V╧≥□d┴²^⌠╫o
∩]□□▌û≡╞A⌠√<ö≈l╬²æ□∩M├\
<T

=÷já┤{%Q║□ü┤±□╜4m
Ñ^Bå╡Tumkx÷∩nπ╜╖-=nπìdx[Ñaì4|╨□îbQ╥£┤&q¿q₧╓ΘY└$┴□¢╟\▓0φ²□d
┘I÷ôúΣ4╣H«æT╩⌐E□ìá▐ô°╣h□ZîûñehyZà╓ñui,mB[╥64₧vª=h_:É

Q┘╤δ∞*□┌□*'┌£Ω ┤O¿\hs⌐▄hs½<h≤¿╝h≤b<Ö╨°≥ú═»□á-á□ó-¿□í-ñ□ú-¼èá-óèó-
¬░═T1î3≤FT╨F½°╨>Ñèú-
«░V≥FX╨ûTOú}ZòB[J=â÷□U□mi⌡,┌gU□┤e╘shƒSe╤ûU╧ú}^aòΩ┼□¡□gµì≈á}QU@[AUD[□[Æyπ?
h+c▀a¬□÷□å=¿ª╛>¡Ñ[∩e┤/½□┤1Ω5┤»⌐zhδaƒb¬>÷)å=½?┌■□<╧£ªb▐Ö£v▀∩ƒδ¢≈√¥≈<¢ëgF,a
F/╧åPI⌐C>└□□F□ΦdÜHτ╥$ÜL╫╙□║ù-ÑgΘ□Ü╧L&Y□╦╩r▓□,ÜòfσY5Vτ□< ¢□□@4öå≥P
Ω@,4â╓□□]í7

äaÉ□ô!□µB□$├zHü╝p□╬┬□Hσ&ù<égσ9y□-═K≤≥╝□»├cy3▐Ü╟≤«╝7-
╚ç±□>Ö'≥╣<ë'≤⌡<à∩σG·Y~à°□ª!ì□#½æ╚(`D□Ñì≥F5úÄ□k43Z□±FWú╖1╨□f$`t2°Ö┼ï/╒±═úπ
¢W╟7┐Äo□îoCRDτ¥b:∩□╫y°ö╬;Ñu▐yNτ¥▓:∩╝¿≤N□¥w*δ╝SUτ¥j╪B├H
¥wΩΘ╝â5'ÅD₧┘□│±\╝□b>^ä□GîµÑxY─2╚-»╧y%^¥╫A¼φ=πî□╤¢≡ûê═1&qêφxG▐□▒3∩┴√"÷µ²·`
─A□⌐1êú°x>□q"ƒ╞g"&≥┘|□Γ|î▀r─Ñ|□_Å╕ûoΓ█□S°n~□q?F⌡4ΓI~₧_A╝─o□□1╒αåE└□`¼╙#ª3
"ì¼êYîlF.─£F>ú□b!lüRê%ì2Fy─rF%□3□╢Hm#□▒.╢K□─╞Fsú5b+ú¥╤□1▐Φlá.□▌▒╡≡▄0·□âîa
êCîQ╞x─□có1
q¬æh╟F£e╟7Æ□□□KìUê╓╞Zc□ΓF#┼╪ì╕╙╪o□E<l£4╬#₧5.□7□»□⌐&'`23`JD╟LgΓUí□af1│!>jµ
4≤!µ1
Ö╤ê┼╟Æf□─╥f9│□b□│ÜY□▒ªY╫îE¼g66¢#63[Öφ□█ÿ±fg─Nfw│7bO│ƒ9□qá9─□à8┬L0'"N0°Üëê
3╟Yµ|─╣µBs)Γb3┘\ï╕╞▄hª n1wÜ√□≈Üç═ôê╟═│µ%─ïµ53□±û`Γ ╪√

°¿èφ s7└n(Æ¥r╦Rä□:íä□□□□□□□æ^ÑJô▐□)≥xX~J□æ.  á
╝7▒□vi*óóby÷□H·ƒ·εfI□ƒ□⌠□■∩ÖÅ3├w∩¥{╩ös╬╠¥□á□□6□N┐α<╬o√≤r-≥□≡□ß<┴_┬_û≤╥■d□
□╬Sⁿ╒²╡8»Θ»π»╧y=□C□s╬¢·[√;p▐╠▀┘▀â≤ε■>■A£□≡□│ÅΓ|ä□î □╬-
≥Å≤Oß|Æ□Ü□6τ3²≤ⁿï9_Φ_µ_╔·□ Z f╬7·╖√_Γ|ù□Å M╬_≈∩≤┐╟·! □ °£-
│□ß û≤»═█W£ Ω?□≡QLÇ□┘╟Zv { .`s«□!│ó□╙H□öα╝Xát Ö≤ñ@Já:τU□5□∞%□j□Ω□□r▐
╨4╨Ü≤ûüvü╬£w□t□⌠ß╝W`@`(τC□#□c8-
□x(0Ä≤G□ô□╙8ƒ□ÿ□ÿ╟·▄└┬└2╬ù□V□╔r╛:░1░¥≤¡ü]ü=£┐□x=░Å≤╖□ç□G8?□8□°é≤cü»□?r■}α
╫└)╬O╞Rl6èë═□¢=6Ä≤δbUlês76□,≈ ╪°╪b▒▄ cK┼&┼ª≡ÿ─#╢Is%#-oµS-├□

é┤╝ö[□)

ñ0É"@è□)□ñ(Éó@è□)□ñ□Éb@è□)

ñ8É□@J□)□ñ$ÉÆ@J□)□ñ□ÉR@□ü$□I□R□Hi Ñüö□R□H□ eüö□R□H9 σÇö□Æ□$
H□É≥@╨□)□$□H2Éd □ÇT□R□HE □üT□R H%
òÇñ□I□Æ□ñ2É╧@*□⌐□ñ□É*@n□r□É□ÇT□R□HU ╒ÇT□R
Hu ╒üT□r#É□ü▄□ñ□É□@j□╣ ╙M@n□R□HM 5ü▄

Σf 7□⌐□ñ□ÉZ@n□r
É[Ç╘□R□Hm ╥□╣□╙¡@Ω□⌐□ñ

É█Ç▄□Σ6 uü╘□R□╙φ@n□r;Éz@Ω□⌐□ñ>É·@Ω□i□ñ!Éå@□□i□ñ□É&@Ü□i□ñ%Éû@Z□i□ñ□ÉV@Z□i
ñ5É6@┌□i□ñ-É╢@┌□i□ñ□Év@:□Θ□ñ□ÉÄ@:□Θ□ñ□ÉN@:□╣□╚=@ε□╥□H□ ]Çt□╥□HW ▌Çt□╥
Hw ▌üt□╥□H□ =Ç⌠□╥□HO ╜ü⌠□╥□H }Ç⌠□╥□H_ }ü⌠□╥H□ □ü

□2□╚ âÇ

□2□╚` âü

□2□╚□ ≈□╣□╚}@å□□□d□Éß@å□□

d□É□@F□□ d$Éæ@-□≥□É□Çî□2□╙h cÇî□2□╙X cüî□≥ É□ü<□Σ! □□y□╙ú@-□≥(É╟Ç<□Σ1
πÇî□2

╙x пüî□2□╙□ □ÇL□2□╙D ôÇL□2   ╙d ôüL□2□╙□ SÇ<

Σq Å□Ö□d*É⌐@ª□Ö

d:É□@f□Ö□d&ÉÖ@f□Ö□d□ÉY@f□Ö
d6É9@µ□Ö□d.É╣@µ□y□╚S@₧□2□╚< ≤Ç╠□2╚|
Ç,□▓□╚B
ü,□▓□╚" ïÇ,□▓□╚b ╧□y□╚3@û□Y□d
ÉÑ@û□Y□d□Ée@û□y□╚|@₧□≥□ÉτÇ<□d9Éσ@û□Y□d□É□@₧□≥<Éτü¼□▓□╚J ½Ç¼□▓□╚j
½ü¼□▓□╚□ kÇ¼□▓□╚Z δÇ¼□▓

╚z δü¼□▓□╚□ □Çl□▓□╚F ¢Çl□▓ ╚f ¢ül□▓□╚□ [Çl□▓□╚V █Çl□▓
╚v █ül□▓□╚

&5τƒ□á\TûÆ)à¬Γ
Ω:TÅ□PcjN¡⌐¥┘%┬₧}}÷≈î╟l"'&▓lNgIí≡█□µ┤~s>Ü┘oaNΣ□┐am╓□┬kW⌡«(:p□°wDb□≡ⁿ╣╞$▓⌐
□uª-╘□∩hÅª▒⌠□ìC\f&═Ñ□┤ÿû╤JZK□# µ▓π┌▒/┌1«S▄=∞ìvìδn╝Q÷E√░':(np▄}∞àÄê□iⁿ╧8│≥
╟3□j≈ß      ><┼ç╖┴├+âf┼π!▓≡e□-╣°┘µk
µ ⌐d╛}j╛yj╛uΩC£╚┬ µm ╢≥□5Γ╓abÅf╝`▀y□╥Öêz╠By6╨sP₧ï≥S(╧Cy>╨
æ.B║□Θ3Hù!}□W>ç≥≤HW"]àt5╥5H╫Γ╨u(»G║□ΘFñ[(&«□τ∞'╞5τ|□╨φµ-J▌íhb/>û□▀┴-
Dä1╙û·Nn┐|▀□ö╖Ür║hK□D·□□M¢Hα┘│ö║÷fN═□ôf□÷┘ ≡□1=≡╘'ε╣□-
≤á═Qæ5« $5>ûP□█¿□╪êφ╖δπZ▒¡U□æ½B□Ñt□╖9│Ω4:□□╖Üi]╚╝5
δ┘ⁿà«zjSê╝6î>⌐ö╞≡╕û□e╠Ä□2G⌠╪≡*i┌√¢≥x□├ZiÇ14\├Sp⌐╡7τq█∞@

QCî╧□u²fùEV╨ï┐╞□² Ω'äwµ┼GjN»□≤»ΘQ=ÿ□╝l╟8□F├╫Rà╡`┤ßb╓║ⁿz¬▓┤├r□aε╖ⁿÜΓÖ'-
K╖─y□H+)≤»□ùx²ⁿ╚⌡5"□W┴▄£Q: ΦΘ□Mm⌐┤╒îJ▒*¼å+Ñε»¬/ò╛Z▄ΩR[□u∞□╣R□ ║□Si,Ç14u╛L
┐?$m?k└□1²τ\k╝ⁿ}'iδ╝≡·7²M╧┐░^╙□èí╓°╚;`ÉM╓X\y3╥[æÄHgü⌠î¼≈,üì║╘X₧¿¡.□□¢S±:G
Fδσ⌠∩v$ç╟║█#<─éb≤
╓╪╚╬τs√₧├{₧;rÜ□□⌐▒G{ÿ□îτ»YqsΓµ≤□╡8n=[a1ÿmL∩23N²

P±∩ƒτg¢ùg═╕gΓû─-e□0╠τΦ□ƒφ■□> îCC┘¼□ƒ-
#|╔╣□>╧τ≡\²Ö╧gù┐Tƒ]«Z}╫ ¢⌡9 *ß│╟_¬╧-W¡>□ =·\|ò≡9Φoµs╨U┬τ╨┐Ö╧òW ƒ] ╥■┘⌡¬φƒ
f}.─ÄQ┴□µδ╣╡ê`_] ·{^╫²┴□u#□e3□ñ╕Mq¢π╢ÿh□Öw·ì-Y;b╧¡°[₧□Ä`□╕Ö▌Fµ□⌐╞6

ÿ□÷□╡Ä∞1r┘¢x¢ª≤▀½áµ·½`'╟σ╝mW□-úcñU
î□─mjvñM¡ⁿ Ä█─

pkS

óf╙«"^S▀_╝δÜÑ?±□°?, ;»Y·□»q·├≥op═╥ƒxì╙□
╥F╫,²╡«q·kgÇ~│▐□π╢s█╗µ√Σ1┴ε┴ßA│R╔Vì█╤5÷Ü/╪─╪□▒⌡∞□
«≤V□çΦ╛zá┌á╓Θ√î□d«□▐□|(°ê□¼▄í╓⌐
·□cÅ·═ öV≡ƒ┴╟ Ñ╝öâ¡╠□£╚Xù□PSΩL▌q^6█┤"(\"□□y( èê"░½╠
█>╝ƒΘ├□h╞FεîêÜ·╛åY_⌡GVp╟╨#4□k°ÅRV¼ªN°╣┤É»Üïu╙àém1ßê╢£åD3ö█ú▄□σN£µ□-
P╛□xKö█í▄□σÄ╕╞▄+pù─⌡□Wj\cï╓£□┴│éó□ε╜□╫┤□╥

W╢G
µW□u┌x║─╜

Ñ[□□═#╧ûHφ░╛bε2_K0·è⌐□╙#&ⁿ6vi:≈
□ú╫≡w3(╪/8î54åuá╝□^□▐d≡□-
□╣░É+Γ½φ*â≈Y┴□î«éƒ□╧□y╖xwÉτ¡≤╔Q<,ⁿ□¼½ÿ▐pσ╡Ñτg╒ƒ╘□□l□l□l□Ä¬ñπ(úw□:^□-c:¬z╡
ÿÄ╡▐┌□WU·/-WWZc□n µ/□V≥_Sgzi¡■Sφ▀□l
Yò┼>
òí√.■l╙F-ßï╘╓0Mmk■ñ╢¼Θ~π▒#≥¢π¡÷Vπä"_░â╙Üfä-Ç?S≈┌

╙$╒
ëÅⁿe∞>│*ò/╕>╕!╕1╕)╕9╕%╕5╕-╕=╕#╕3°BpW≡┼αK┴ùâ»□w□≈□≈zσ╝d»éW╤½ΣÑxò╝*▐
¿╒pDα6;8RæZ√□√□□□□□□□□□□ç≡x948î»·gpl≡Aµ ßα#┴
>□|î∩)Θò≥□╜╥^□»¼W╬3kò>ªi/╙ZPtƒí┬úÇ┌ª╢(≤÷YîzVφT/É╧╒E-
è▐óÅΦ+·ë■bÇ□(□ë┴bêX/6êìbô╪,╢ê¡b¢╪.vê¥Γ□▒K╝(^□/ïW─n▒G∞□»╩□ÖEfò┘ñ_□d¼╟.s╙£2
ù╝Nµûq2(àöR╔□YX□æEe1Y\ûÉ%e)Ö(K╦2▓¼,'ôΣ-
▓╢╝U╓æ╖╔║≥vYO▐!δ╥;e□yùl(□╔╞▓ël*ç╚√ΣP9L▐/ç╥□rñ□%Éúσπr¬|BNôO╨Θræ\,ƒæKΣ
rù|Q╛$_û»╚▌rÅⁿR~% %┐û▀╙oσw≥{·âⁿQ·Tî╩ó▓¬lΩfUK▌ój½[U□u¢¬½nW⌡╘□¬╛║S5Pw⌐å¬æj¼
Ü¿ª¬ÑΩú·¬~¬┐□á□¬A<gìQ Tc╒<5_-PO½àjæZ¼PtQKx.[╧≤▄F╡]φP╖° φΩÇ:¿

⌐w╒{Ω}uX}áÄ¿□╒GΩcuT}ó>Uƒ⌐c:ñ≤Φ╝:ƒ╬»»╫□tA□»
Θ□]X□╤Eu1]\ù╨Ñt□]VW╥) ▌ï ▌K ▐╤ ▌n∩5∩-o┐≈.k÷zqÅΦ┼3ß}é█ÅxM□íδÑûà¿£,/kQ
┘L╢°å≥-
≥!j#g╚╛ ╘Q.ò+⌐ï▄+≈Qo·ôⁿìεS~□ó□¬ÖjFôT+╒₧&≤╝<öª½□╒xÜ¡û¬τhæ┌ñ6╙RnC█(▄z₧S/¬²┤B
}«~ú□║ñ.IoδD]Ü▐╤σt□φ≈6{[Θá≈è≈□╜τφ±÷╨√ ▌δ ▌δt╪{█{¢>≡

x□Φ□╖└*4]╟□
─╙bíX$□ïg─□▒T,□╧èτ─r▒B</VèUb╡X#╔èuΓ□±ú°Iⁿ,~□┐è▀─qqB£□┐ïSΓ┤8#╬JÆû⌠╔₧▓ù∞-
√╚╝▓ƒ∞/□╙ürÉ□,║╤ ró£$'╤)r╛\ ƒû
σ&╣Ynæ[σ6╣]εÉ;σç≥#·▒<*?æƒ╩╩Σ1·╣ⁿB₧ûgΣYE╩ReTYUN%⌐≥*YUP□U%òó*½*Ω□UUUS╒╒ì¬å║
I╒T-
T7╒]▌½z¿₧¬ùΩ¡F¬QΩ□5Z=⌐ª½□jªÜÑf½9j«zJ¡R½╒□╡V╟Ω∞:ç╬⌐sΘδtn□°âZh⌐ò╓┌╓Äv╡°+Φè▐
N∩□où≈¬≈ª╖╧3▀╡╥╨8Aπ□Z.□-'A╤)╨rUh╣□┤\Zn□-
wüû╗B╤╜íσ■╨≥²╨µ(hp"48□║¢□▌=□▌Mâε₧4÷Z

▐~S½"z□'╞ï
bóÿ$&ï)Γq1U<!ªë'┼t1C╠□│─l1G╠□OëyΓC±æ°X□□ƒêO┼gΓÿ°\|!╛□_ë□ë»┼7Γ[±¥°^v
É□e'yÅ∞,╗╚«▓¢∞.∩ò=Σ├≥□·□≥Q·ÿ□'τ╚╣≥)9O«Æ½σ□╣V«ôδσ□╣Qεù□ΣAyH╛┼▀ô∩╦├≥□yD-ù'Σ
I·╗<Ñ≥¿╜*ƒ╧»«W□TA□»□⌐□UX□QEU1U\òP%U)ò¿J½µ¬âΩ¿:⌐{Tg╒EuU├╘²j╕□í&¿ëjÆÜ¼ª¿╟╒T
⌡äÜªû½□·~╡R□W'╘I⌡╗:ÑN½3Ω¼&miƒÄ╤YtV¥M√u@ù╫╓▐6○╗╖├█δ╜ß╜π□╜*⌠ÿµ╘?µ╣□╧üß3 è`6
4g╞'δå<o≈╓ì8φú┘╞□}¥SΣsfΘª└¢□o

ⁿ┤┴#δεÅb▀b□╨Rd□┘Ö╧£"7ee█VP,█╡║Ür░¥□ó\"ƒ(HAQH$É
{ ▌□ëó□yó╝¿@·E%æB□┼
ó*□□╒┼ìTX▄*ΩPQq╗¿G┼E}q'ò□
┘FN⌠Ä{°¿║w&öò░□/·E·□╙ΣFφ₧u□·=ⁿFs°t1│g╥Γ·q□ε≤qkΦJ─}╗7eâ
□ƒ╞å┌`«αk√2ç²┼□ⁿ₧┼╘¬{Θ▌°╫$□∩▌

∩V╪ÿi□±ë«α9-
uf□├□°B⌐gj\M⌠Ñ₧tiZd⌐t┐□m□█0?%É□'·°╠I1.S/·╣¢╚º{╕[Q▐å≥fö╖á╝□σ□ªL⌐g□≈ëh╙|╔07
╧½¼y₧K¢ÆΓy}□5σÖ}□5πÖp□5τ┘²□╡σy/ëF≤╠\Ö-╤U⌡¡⌠ÿ«»[╨ô║
╫·┤₧»ù╥Rg▓3¥V╗□▌╜┤┘²╬+L»c╘\□iÅ⌡"6╡yεfN═╛}╞ck□╙□▀╕□j╛-
□l=>sw÷Ü{□√□Å;8*°tpí█┌m□┐¿/j\~┴█╔["┐▓⌡□╒e□.'è□TÇ⌡╤ëÆ╝π,≈åæ;C╤╢┐□Ü□□╘/εëJ(
□æ╨à┐¢╒ô    hπ⌐Wd^ƒ┐╟îh╥û

δ/$≤ÉOµô□)ïLû□)V▌ ½SNYC╓ñ8Yïm$╓□h□Rly6 ç¡╬╞öçm╬ªöƒ-
£□Θz⌡▓zà□¿╝Ω□ègkε□ò4÷□òbkεS*═□╬n¬□╧╧µ┐IΦE ¥□H▀é╢Ñk!╝/hA□■n$ÿ□1▒Ii«
╖▀╟Æú□}-å]c¼□c╙Lck╞╪2s┘Ä1v½▒Zì=·,[½╞V]#╓Ñ▒□┬□┬9█/l·Ñ┌ g┘B0╓ü▒
Rm└8╢■îσg∞>╢□îφ°□Θ├·ê■P╒ƒΦO⌡1²à■R□Ñ┐╤▀δ⌠O·g²ï■Uƒ╨°⌡Ynélv□;½φ╖cφ∞v

√:;h
[┘ ┌╢m╟

┘·φ□vA;┴.l□▒ï┘%∞Æv)╖î¥dù╖+╪□φJvJ─>[¢∩░¡y╨;Σ□÷>≡Äxzy-
{G╜O╜O□ù0co~╜w0σ□·║Ω⌡□~K─╜+±□╛□ôy≤╖B╘╥──=+q-Ç□╧>□[µe"vÿÅφ╞I□╦1≤(="Z╣qn□╖ß-
Pißö-s«╦╪Lòτ╦1ⁿ╬P¬ì║=ôh⌠Q╛ê-
5ô=□┼T╬πYfü\H!╣X.í|*ù¬╔≤╤m¬%█X□╪╛ε╨÷SW□dƒu&╤□╫τf│r║□7ç□ts╣╫YÜ╡□┤□W║╢σ╣₧¢╧
╨τ^∩□░Γ]■│□▄ΓnóU╪-πû│J╕σ▌d+╤¡ΣªXe▄*ε
V9╖║{úU▐¡Θ▐lUpoqk[ò▄█▄□VσP╢Pv½n(gHÜ╙√úv╘CÖ,9i╕'2▄SV├=┼□ε)çß₧r□ε)h╕'█pO«ß₧
<├=σ7▄SA├=□2▄Sa├=□5▄Sq├=ò4▄Sóß₧¬□ε·⌐ª▌┤Äî□5"¡Gï8!à-
≥ê╛"^□□Ióó¿,¬êjóå╕M╘□wê□Γ.╤Qt≥╠≈ôr▓
▀Mt□=DO╤+ΩG│□mb╜▓<[□─û┼1Jpw▓U\□□D□<╖FÜ÷║π□ƒδc □/ÜdîTl²ög_:A

a_║æ|\N°N≡Ñ╖╦□Σ-Ω°µ⌐%4ö⌐°ì■íCl┼»5±5zC□╒├Φ-²Ç₧G∩8O8'Θ□7°¢╙jßµvs[-
]ß□½òδ╕«╒┌═∩µ╖εv
║□¡╢ni╖┤╒╪Mrô¼÷n□╖ó╒┴¡∞V╢:╡U▌¬╓=n
≈&½│[╦¡euuouδX▌▄ûn+δ^o╖╖█ΩΘφ≈÷[╜╜w╜≈¼▐í□í□V▀4φ»╞eIé»f¬s3╡L)╙YÉΘK2t1UU
5L
╙a¿□σÇ▄δD|ÉÜù·<KTå=X²#
⌡ß7¼vF-=π#c$@ Væ+¢Fm╞□8-Çêt?1
□7µ▒¼Æ¬ªnV╖½╗□YδàX∞@¥□╤╥°s▒Q▌T7╙═┘√bj⌠²æ║½├/)□¡=gÜhπ2₧τg≡<nónA■-
+b╤⌡╣e,ê╓□~δT`m▒9j

èqên=□kßyu\¥╤ö&╬├6Ç±αó¡|-
%░═ⁿ□╬/│d□┘□╡dIÑ┌<├≥┴∩÷QU+∞╖φ╨αs,j□╤ΦΘÜhñ⌠\£45B□Ä~Ü╪g8▐·I$╞YF%GcÜ╒"Q╟^jñæ
Q╘

ZÑV#Z ëÑëQ▓═c▀k≈░{┌╜∞▐v╗»▌╧εo□░ç█ú∞╤÷□{¼²á²É²░²ê=╬-oO┤'█S∞╟φ⌐÷□÷t{╞-
Hδ<□a%╟┤╨≡«╨□═∩∞┘□ö┼Ñ·╛Äƒk¿╔═┐□√.q≡]□ó╢x╘ûä■\ôG¢ù░°0ïëT#:ví▀·╥ ¼╫½╨z╜╨╗■ƒ
₧«F=àOlN¥┴_å□▌E▄+ ·èü&N╟c┌8╓□Åk,┼'─□□c╓□°ÜÖb

e□Oë·ö▌îb¼╗eb□σ□╧ë╒□g╓~╪b4cä╟2£H╒î

⌐:╥o:▌dΣG5aO▄┬úΣqΩ┬r<E]╒□uåεσ13ûz░□s╤ ─╡r╙}<ry4öÑXæg▄▀∞□z╨pG╗
w┤□╢╚kå?zâ9ⁿö▐è╠$q╪□▓□σ┌⌐c≤y½□Oëy╥ò-ÅKyydèùàdè¼"_ô»╦7σ█≥□╡□δ□∩E╓$N⌐ⁿ¬ÇèWE
▒Γ╨Y
g▐┬½□f┤□╖à3□_E╨²⌡²╨╓^∩ Ö□]î╓τìÖåR≤╬╛∞½Éfee½½□[□Ñt)□□¬IÇn┼û╨╗X┐╖h^Ü╢⌠╩U╚Cû
τ─┤ƒZ□Θ╟G-□≤m□∞Ré²i╨¼·□≈`ε╜▐ß
«▀s┴⌡Fb□⌡Aû╤╗·}╢N>╨│╢?╚┐R¼ΘçöçR┐8ÆZ╟^≤

¼2«UδòëΦ√°ë┐□q╖>B□#╫'c□,à;╟▌δ∞f□□-DKq╖±4╪ ╜╦m□
«£kv□ûì¼≤┴εα1φI-╖N│╝┬V┴□²/εS9m╫N┤½╪7╪Uφ;φ□v□√εê}0*b□╟┤ƒ╖╖╪○█G∞ƒ£9╬"gà│╬┘ε
╝Γ╝σ□è╨╡ÖΘè│□≡¼≤□√□═╩□[åf┼F

αqc□█æ┘─z±*Å·?H-[╪^Ló▓°□M7╨±l∩7a[g      ╡g[g'uckτ□Ω+?ôf╤
·╣ⁿå□╤∩Σw4┬∞□áæ≥┤▓Φ□╢üÜ╥XεK=Φ9║}□╤J╢□╞╥&D _P█╒. ┌┼Vf
┌
√⌡5=P□í7▄¡ε

z╟{╤{ÖL_L°π6□mg□╥╧╨┬

^ñ7oìΩ÷╡ Z⌐\δ¡c>¼⌠eτφoα□Äg3∞
`

;<ττ■7;□

²█╓±·□¡Tb"s^¥HΣ0ûk^╟ch□9ÿ┤|XNíÆ<b=Eò╕ >Åjÿ;Θ.╛w□5b½f-
5e╗µ□╛ΘσcoÉ╟vπúG┐□Ü□ò≡□XS
G□LTAΦ·<▓å£□╬6Ωσ╜╧πδΣ?¡π═

╒aa^í4≤y□8ïQy□{<zâ▐J┼⌠-²□ò╬╨¼□[ J├[|O ╝╞
MfgM

ó$Φ░□⌠VGwe╜╒sñSäεÇ▌┌ ┴╙╧QX',{òKòÄ\□»wΘ▌T╞>δ□*φ-v□│7v┌=M7{ÄτP-
»à╫én±zy╜≡φïî╪□å■√#╢┴█|O0□cdBI▒%□b}τô╫ô═□AA≥d□YæB▓¬¼Fy▒≥ò+_□ec÷□πß1□╥
Ö▀□²┤~ÜJΘEz□%:?:?1╜□Öδ▓ÿ┘S«Ç»░»i÷□∞JCwµ─□cú½█àXZ╞è)□
·V╚á□V«√:?0≈C▒r=□▄Å┐"ε├>£┘c≡╥□% ⌐╜ú&zGÇ√□ù╘m▄δ□zW╝{─=B□▌│εY¬Σ±□öΓ╡≥ZQe»Å╫
ç¬d░_╘Nc3┐sY╧a□╧█√önG¢~E∩╓√⌠~ç□╦ëq▓:▒Nv'ùs¥ô█ëséÄpöú□ç□É╧╓∩□tΓ¥BNéS╪)Γ4pε
r□9M£□NK°ìs╖╙╓iτ┤w:8¥£{£.N7°°╙╦Θδ⌠s·;□£ü╬ás²┼ÜÉåƒ╟i/%┘~1≤╚*₧□K╙²≥
¬"Å≤ⁿW-
δ□═╒05Ü:│5ⁿ□⌡R╦╒J□ÑÄ≤ⁿ2□^µB=\Åá%zÑ^O╦⌠v╜âV□□╥□╜╧>Cδî□¡ú,╓□δSûfVδ3#Qδ□╦TX_
□iZ▀▓<□δ{û⌐g²`Σj²╚Æ-b²ldj¥d⌐6▓N▒d¢X°ìt¡3,▀

>2Æ⌡ec┘v±□X╛▌│▒F╞╛∞,σA╛£lw□Σ□╬     τñ»1█V╖│M┘½y╒╫┌{├{╙w╖±≥}φî-
∞δ└s╤o╛Nù╒û╥⌠zk╥ ┤x═j╤£╡Sî╫φ╓▀ù.è╨▌í√î╖─X+ÿ+):COë^ov:v□]Xτ≈è{┘"φ+·▓O9P

ñ□÷▌]DsCö□│u6YT□%┐,.ïS@ûòe)□±▌∞ê∩å0[ùä╫]□╓QU▌X7ªz║╣nNwΦ□⌠|¬ÅY╜/f±~XeΩ□æ╕o
²46─╛╙¼╨
│é├╧╨#≤P.╢!□╨u<+4Γ▒╢ën┬□¢ß╟$ì╣┴an╖PΦ□Γ╧{/□□6°Y&Ñæl«╚□ê┘ôÅ□·lâu╙=£Y╬∞≤Σ▐Ä=
εâ·É~□□└╧┘√■Z□½┐C

≡GD□□╙╟⌡
}□╤┐□{µ╣∞▄v□Γ□□╤?╫÷∞É¥□æ┐Γ∞╡ù╣╥┌σ□√K6æ?÷╘g┘│φ9÷□{Ö²,{φ+φ╒÷□{¡╜╬^oo▓
7│□┐╒▐fo╖≈┌»┘»│G Ä╜▀>`┤□┘∩┌çφ□╪├ ╨■╚■╪■┌■╓■╬■╓■┘■╒■═>nƒ░O┌°╣τäGêd:╔#D▒

╥á}Ü╜╞S┼\ε│ô□S=íN≤▄╜Ç╡┐Éu┐X?s┼ⁿ¢□F5╗‖}ú]╙«g▀i▀e7╡¢!ªqÑ£_i½╔□oΓ\δ╡╕⌡v╟┐c╣G
Ö╡æófuäσ±4≈é⌠≈ç{gü╚▌√úæó5j¡█÷"╛Ñ╣‖!«=└O╨--G1Rû└□Y□cΣ▌·Ç¥B□xL┌F/Ü╤êÄÖ□ê╛F<
⌡[─S┐ç_d<NrÄ;╟ú>ë9í+Ä╞ñ≤┌z□»
■Z□°k┼ß»U┴}Uß╡Uâ╫V□^█Mτε─=□≈Σ╟=eX╧)T

≈4─=MpOS∩(╙dⁿ╝@╪W├║░Ç¥ò□□V)Hí4|╕▓f¡ûk1╓VRt□E}J▌┐y0B┴½□□JÇé≥áá□(¿═□╝L]@AOP
╨
□⌠╛"┌cα▀□ⁿ[□╥ ┐σe¿2_ß□□Ω

E47□Ü¢╞Æ=«□ⁿ

ÆXå5τP9h«?kn□Ma=╜CO░µ

╥4╓▄╟⌠Σ□╤ΦC▄üα≤δê╬δ├"&╨□□±ê∞≡D=xó7q½▄Hw"Ä╨╠╡╫₧A¡╣_¥í╟╝»╝oΘ9∩□∩7╝
w╣t]╤m/╜v▀²
·σσsα ô;ì┤□B[="úW□¼□à╟ƒ≈╞□□█□-
D+J╗ú╔╝□b▐τ1o}îù□"w□Ksτ√Θεî1w║µîΘ╓╤╖QR╫╟├∩£`]<uº□▐□1+ßß7F┬δßß7E┬kΓæ¥Tl□ÄD
ñ{9╖╞5□□∩«v^─╖W║ÿ≈□₧□╬▓═U╡┴╢╙Θ□ì}□∞|il8╖mäG│□É7┬πa╙D╗fÅé%╟W╦|jé}gt□O}+⌠□
≤╗·z□e□!æ,*ê╗E[□■·j(ìWx$,c7ⁿ6µ▌n□╛"□?¡"?╡&ò╞·┌╌3±çxªB¬æ&Γ«▓.NdöπM¬: □Iµ
qΘkI²B▄Ñ╒ôz2│O}ú
`»i□╝+l╓6╠┌JêJ▒═D¿╟F┌òR▀σ░d┬yuYjô⌐Q}íN░╓≥░v-□Ñµ;┤5Ö╬□□ë▓P□╙₧-
▒½s┤^nφªÄεö□e√ê╙□□▌Oô╤¥;▐ΘPÄ
₧£·□║ⁿΦ_1ó½▄╧┤ÜSSπYf1¼ù□µ9åÑXÅ□δûßw)╘║hiW┤┤!Σq⌐¢·5Z┌□-
m╨¡░Ω╕.Æ∩èΣµä¿≡√Dª┼·"OÅπ□%p^äƒ_1□è╓□è╓□è╓█&Ro¢H╝m"⌡ÜV£·■╞TH⌡πL▄□·╕Xóª¿Yf╞
╝╚╪mΓ3╪ùDë□┴╤`□/ç□<     #x·tⁿ╟□?G3ìƒÇÿ!µë□b¥Ü¿ª≤ⁿ░≤□·ZßδæLσ╤┼╫α□╘
P_□╘'Ç·"á╛□Φ.nzÆ∞$╗╙▐▓ƒ|LNNºƒyÖ¼ƒ∞Γ{ΘôkσVyH-æGσ□≥ö▓2□□Lσa'xº$╙x□ë┘bòÜ¬µäW
·╥∞q
»δ\░╕□╢╔_╚╕&ü≈O3qÅ÷,▒≥□y┘ò«O}ûyæ≤╦ΣüÑαm≈^╕╙ºÉy╝\┌ºαô¥e▀ï┤½╞ó▀┤½/┼∩□
□□

╝╒ú÷╪1XW%T⌡

▄¥y╗╨╙╙ⁿ9h.Ñj\C4□□Ü□╒MW1═Θwk□ëv╕□┤║M╞Vô═╝╡q∩WXM₧/₧µ₧░ê²┴□°â□■`>°âÑßCûà□Y->
d≥□ìïµ·5óv÷┐.▒O¢HTG│#¢}╟┴l╖₧_▀╫ùQ▀#r□╫7ANí¼iΣ□>Oµ□: =≥Kù±╖É±81üe<I╟c□¢XÄB
,'b9Ñ□┐)ï°My─oÆ/Θ9┼≡£∩L\ë╝µ□·9∞7│ ≤ô⌠E÷íe┤ª0╫▀╟║□ƒ╒Çô7.úÄ
█╒r#╤v│▄I■K¬ú□Ω°æ∩╔åUb/ 8)πτôf?▀"╧¼ÜP^│j□>mΦ□Θ√
Ü═kVåUYUôΣe╘±3╝╣≡)□83ΣÆΩ□┐ 9≡
<╥òùtg╕▀ èæj¡6°╣eIw∩0=î□εÆ□<∩7<∩yJ1÷ßt╡s½E╟3må┤öim>╖ï╖ü(ñB

5┬è├zûÇ▒ΩKÑí0å)∞─ú»╧mEm╝π<:n╙4╦$F,
Ö C╖ ║ G╕╣P║'2Q‖≤3$▌ ╫êtτGñ;%ìtOfÜtc╘bs·Så$|≥ æ0╬▐╖*F,Φ╟ò░Å≥èëb"?╒╨jëÅ─Gl
·ⁿ(~─zJ ╧"{╧₧öU>,µyc╜ ╧╢└ 9ç r╛£╧6╤*╣è▓╤Mr σÉ'Σ
╧)┴╚3öKσQyΦ:UFòí▄¬╣jAq¬âΩ@┬─╗H¬aj ╧╓#╒H╥j9Å£╢ë)ôç≤L≥Ω√⌡²l╔┤u█╥⌡É] ╚
╬¼]√╨ X≈V²½\≈╫óT╙»ç■╬iV▒ÉΘφ╔tÄ╟ [m┘ÿφ │F¢ 6_▄ ±≈Sªà ₧) '├Ñ9 Äm╓╘½¢G«6oM d
I-d ²ÿ·$û─|û└&µⁿ

Vÿ╙Φ□─r╡B=^Ñ5|1W├Öú≡l¥ƒgδ□╠IV╠┘qÿ¡â╤g╓Å<<≤î±K.τÖæsxb"3╝┘ßY□ºªÜ╙Rù╤Z┌N{h╝G-
▒g·5[Z╟Θîò┼╧n┼Y╢ò╫è╖èYÑ¡d½èU▌¬i╒╢ΩY
¡ªV╥+ùÇS□□xô

} Ç

╙Q÷╕îà□N┐e¢*
□Æ□ôì▓X>+`]g).EΦΓR~+┴*aòσQ⌐öò─iÆò┬iè┼₧1S[ï╙ZV]NδZ
8m`5σ┤⌐╒Ü╙╓V□N;X]9φj│Γ┤ù5Ç╙□╓PNçZú8□eìσt¼⌡(°ÅZô░
╩▓x~▒ª22═Ü═Θlk□ █ⁿ╧m╟Z∞╛╞¡≥zùφo]╞▌i÷□╖/s┌─}òφ│□εδ°⌡{ⁿ·&╨?áⁿ□╨?óⁿ□εz□w╝
ⁿ'α╖Ç∩□■

≡ƒü┐□|□≡}└□□■□≡╜└≈□ □°□ö□C· ╨╟Q>ä≥
ö▀E·$╨∩íⁿ;╨∩ú|□σ├(ƒF·□╨gQ■╞ö╜□(□ïrNö?B┘B·(╨1(□èrVö?C9□╨╟P÷úⁿ%╨□S÷▓#
-é'~Ä⌠+ñ_#²□W·p╫'(gIs²□H┐0)·ù·□δb£Ü╡□}∞lªì╗Y\╤╡0τ

'╥□ΘN&□π\ó□┐▄±εx■mèÖÅ╝b^q*□╗┬ⁿ¢≡∩"α╞□7fg╤b├ìE□RofD,.╬ìí>╝╖-d[▄σ)╖;╒}┬¥µNw
g╖s▄╡║ε<w⌐√¼╗▄}▐]φ«sq╛□NΣJ▌]═╝□od√▄╒₧±à·Ü≤w£[╞NⁿOφ87ö□µ^rn °╓4TdN[
`τ4aτtn∞£.□^Ü`τt□∞Äδî¥╜▌└┼░4\£█□╛²Üµ├╨nN□□∩`·3□¢gO≈ê{╓≤ómizä√,t╡∞╕NKcΩ▐δ£
Q*3KG·□wφÜ²║═▒█Ñ□÷δ÷├₧ùA╪≤2
√ugb┐εl∞╫}Z░╧╨"│c╓z□;f□v╠*∞ÿ
a╟l^∞ÿ═Å□│□░c6>¥T╬φf╛ε□r9»╡ÿêwæêT■{g□5æ I6╗ànì√öL▓ó"rê□** □"□□"Rá<?
(m)┤┤<□,□V¼@□yr±□╓&"□

□□y□□□=╬π<DN□æ□ADEö'ET└▀╟g°u╥âx£ê» ▀╫╛ ·₧┘123Ö╝ôL╢┘o▄Σ⌠{é%Γ3

□çî#╞╖╞w╞≈╞Å╞ πñ)Ö▓Θ6□╟□fóΘ-
█7&ùò├J▄▌ ε□₧M┌░=■(╓┼=ù·┐▓┼|▌█▓y╟─¡{%vnHgφÄ┼┴□□ïä├Ä5:;[\╠╬□W□»

VÄ▌Ö╓∩'f╡▓π>=N √╗B□m
┘9]j□╫┬_╛┐÷ísGa╫-
□·▐π[⌠·zc²v╜⌐₧ªw╘╗Φ╜⌠▐z_²^²>╜ƒ₧«≈╫│.°▌╓┐«□cw0∩g=□■╒═▄φ□ε`■□τ₧σ_╜╟K{D╧y□?+
√╡pΘ∩Bw"R4Å□=#D□□Bd¢╥╕6R≡╛αáα□┴]í╨íÜí┌í█a-
Å½±bΘ]═°□\±ö□6ÑQ5Fi»koⁿ□╞-Ç(:C□=°°⌠¿9□┌|Zâ▐B□╚╗┘Y┴íO╙╜⌠□:ç╬Ñ%t-¥O F□╨gΘs⌠
y║É╛@□╤□Θb·□]Bù╥et9]A_ª+Θ+⌠U║è«ª»ß~≥è╞Aπ□π8;[□╙e*ª╟¼êo□╟≡ ù╩╝π╢²K°n√╕m·δ╖
ßƒ█∩□[à⌡⌠á3╟Äb√O,÷S\Σ¯│F}:W?¥wT¿s«ƒ±+╓≡,╓1τ°≈°□╦¼ô
eGπ╟╪□π±└πl□τ□□ⁿ°p□;┌□
-'■╨eí□ó│1z&Ifú½1Σ□v╜₧JΩ░c÷tRù╖╓═∞╚²=╣%t"té≡;üo#
╪╤╖ i╠Ä└¡╓φ∞(£JÜ░#q;╥öìI3v<εJÜ│crO╥□┐ m╓╞^▀ô- ⌡1÷lkY□≈"

î█╖*ö╞Γaπ₧.°□□Å╖V■<─»C2ä□qτñ│ƒS╬┌ér╤'Ñäïx╖faα╡╨#°▀Y·6U╕hm□□╞□-
ⁿ=≡v░k░G░ 8:8>°dpC≡╜α■αßαw!OhBΦ□≤U≤∩°╡╣ΓE╝
▒8≡VΦ╤╨ⁿ╨╤╨wⁿ{ss3╗▓═╡}ë□±n¿
ü□£╒-∩┼≤.╪=8╩\e«cú¢⌡d╖▄Zn/wæ{╔}σL9G-*ÅÆ╟╧□Σ╔rD₧)╧ûτ╦ïΣ□≥j·My╜╝I▐"∩É
≈╚ƒ╦_╦▀╚?╕dW□ùΩ□╣┬«╩«Ω«□W]W#W3W+W[W□W/W_Wª+╫ò∩□σ□δÜαÜ∞z▄5╦σ╕J\
]K\+]k\δ\o╗▐smuφpφqφs□v□s□WdÑéΓSBJX⌐¼TSj(7*⌡öFJ□ÑÑ╥FΘátSz+ΘJÄÆ»îQ╞)ôòΘ╩□J
TÖú,P□)╤öWò5╩:σmeô▓E┘í∞Q÷+▀(╟▌è;╔φsδεJε*εΩε□wmw=w#w3w+w[w'w□w_wû;╟═╘═╩═╔=
╤═▌=╙φ╕τ╗ƒs/v»t»q┐σ▐Φ▐Γ▐ß▐π▐τ>Φ>Ω■┴C▄G=-OÆ'α1<Wx¬zÆ=7zΩ{□{Ü{Z{┌{║xzx·2fzr
<C=□₧B╧8╧$O□}æδ□╧,╧l╧|╧s₧┼₧□₧┌₧7=δ=□=[<{>≈□⌠□≤£LP□*$¿ íäpBσäj
5□j'4Lhû╨:íCBÅä√□▓□□'□░▐¥┼zε□k√ⁿä┬ä Στ τu≡_3ix.Gr≡ïαwñvH

àI╙P╙P'╥□╧σ╚
σå≥╔p<ùc

εm□ÅτrL┼7EOk╧i/Æ┼t8¥NVα□□∩┼ΓíαI□[═□¢□╙Gµ╗xZ╬√µf▓□▀#φ"▓┘╞êÇÖ ╗&6█
W□÷□ú─┼τ0ûΓ▌@╨□GÇ│┴°1╫□Q₧-
ûëÑ□åà⌐╧âàα│°d4µO□╠□y+Ræ+□çÇ╝□⌐ó□⌐hE*ZæèVñó□⌐hE¬hE*jO□¡H□¡HE+R╤èT┤"U┤óâæ┴
jεÇ\äMg∩┼⌠aá□Ä□gÇ6µ□å╣:bΘÄ╚±Ñ;èÑ;bΘÄX║#ûεêÑ;bΘÄbΘNl▌%│□r□α0÷)¢èO;π╙╬╚eÇⁿ╙
╬°T2╗░)]î<░□t└LÉ/▀╒╚□□6wWΣªÇyαp╨□ï┴±¼tF6W,□*RKñQæ

gσucKtCI▌P{7╘▌
╡wCφ▌Q{w╘▌□╣)`-8□┤┴bÉ╫▌]╘▌]╘▌]╘▌]╘▌]╘╬F8f_πA0□Σ╜▌□mΦa<□Nb≤⌡d╣{1▀╜ÿ∩^╠╫╙ÿ□
>□Nb∞┼·╫┼ë₧φà)Eb╨R╜□ ▐═@:╚xXñ╜∞{░⌠=bΘ{0ÑHLYèw□─╖□HcK▀#ûεm,□Gâc┴LÉ∩□╝æ¢□Ä□
┴(ΦÇS┴iα╦¼dV□jΩì□e│□jΦâ□·áå>¿í□jΦâ\l╛1`!8□£□N□°ü╝å>óå>óå~╪╓²░¡√!7□╞□çâ6X

≥m▌Olδ~b[≈□█║ƒ╪╓²░¡e3□e°ú∞tΣªÇy`l

□,□y┘ΘÓ∞tQv‖(;]ö¥Ä▓┘æ(□┬^╥S□█┬□?2ê⌐⌐bj*ª÷

╙╪:#σ┐ °¼égâµÉ□ñ□wⁿ╟"s╙sd     yò¼┼ ▓╖æ╜Σ 9&▒QóΣô

Θ□⌐Üö"╒ò□K-Ñ6R'⌐ùö.σΓ╬□■╠&é8╙^─Ö«─jmk:¼╢╡╞□û*,evë)Äÿ2
.W·YÄH√ït£H°ït¼╓╫¿¥╓ÅÿφX┘O│σ█í∞╪□GL□□ù+²,Gñ²E:Nñ╙E:ûÑ▓┘-5╡72D:Cñ▒⌡h_╢-φ╤z
┘L├╖41□Üÿ?M╠ƒV6□□µ s□⌠τ8Φ╖□□┼╬╒□8□fαhÿü\□╪Σ╟ï

1□╔└□$□Gà

£╒30□╓└□$Cî@2pÄ╧□#É

1□╔└□$□G╫

î@2─□$S0□Σ¡êσè└! oEªhE&ZæëVdó□ÖhE&Zæ)Z□½=╙(Mc¡╙D+2╤èL┤"S┤ó?F2²æπG┐■b$╙-
τ╠■¿¡?jδÅc~□ö█_îd▓░t□r|Θ,▒t□û╬┬╥YX:
KgaΘ,▒⌠□ît□ ù□≥æ╬□1

□êO□"ù□≥O□èqP6¢Æì│A6F"┘¿+□╝Öìσsp□╔┴□$□╣)`-8□┤┴bÉƒAr─□$G£Ar─□$G£Ar─Hd□[b□J
□ä┌□í÷A¿}□j╧Eφ╣¿=□╣)`-8□┤┴bÉ╫₧+j╧□╡τè┌sEφ╢óv^╞pî"å□▒<∩φ<┤!□π¢<îâ□│▄□╟7□≤ì
└|âqv-
î·□c□4□#Ö!b$3□Sè─öÑx7@╜¢ü46Æ□"F2C▒⌠P▒⌠PL)□SûΓ▌□±n□╥╪╥C┼╥├0J□åQ╩0îRåí□çaÅ□
å▄

p

X□FA□£□N□·(eÿ□Ñ

□úö|╘ÉÅ□≥QC>j╙G
·╙┼µ□□□és┴yαTp□╙k╙□5Σï□□░¡
░¡
É¢□µü├A□,□·╢.□█║@lδ□▒¡
─╢.□πáæ({$╨-ë▄□0□î═aâ┼
/{ñ({ñ({ñ({ñ({ñ□□eê□Oå□±d`─ô)ªfè⌐Öÿ:Lîâ≥□╥qPë□□òöìâJ─8¿ñl□TR6

*□πá□1

*□πá□1

*□πá□┬╟A≤─8h^┘8h₧□□═+□□═+□□═□πáyb□4OîâµëqЩ<1

Γ╢┤GM|□T"╞A%b□TR6

*□π ▌û4╝K□≤°ë·╙─ⁿie≤°a■≥≈ì┼ε┤J·C▐7V■

1■□î¬µ(s┤9╞|└,4-
4╟Ü□ÖEµ├µ8s╝9┴,6'ÜÅÿô╠G═╔µ□s¬9═£n>fF╠□µπµ_='╠'=ÖµSµ,sà·▓ií⌠╥ì»YΘ╒╠jΣjqGMr
┘□5Jⁿ=═╕o≈e≤Xÿó°$╓□½∞╛-ƒ°«∩Ærƒ≡∩·sQb∞S⌡¼≈Ī{╧÷tD╓3-╥▐¿`T!]îkìådê╤ê-
9-¡Öï^9╞█─úW1
■j>àm█%ε√G⌡7/ ╘⌡‖⌠□»□δ╤╙╓Ω╥▀╕⌠S╫╓w┴╖╒h≤┴╙╓╩≈¢ù□Ωz·/≡z%▓=▓êφàôΓ≈└╙╓╙□┴δ;u╝
□□x╝□─□i□;┌╠2¡╙╓8p□k:u]â□x]ô╪17v─-fG█Gqt┼░⌡┤╡

u■╤┴ í_<'h□σ£á]≡sé~□╬ ·□>'╨ïrNá□ⁿ£`ⁿ!╬ ╞∩~N0/≥9┴ⁿ□╧
ß?╟9!ⁿ;╒□□qN║î▐╧«VïYΩ1╢□'=~f║Y└«ä·▒Z!□╪5h□┬□□□"▒□îgkƒ\╓╛╬h▀`┤o□z□Oφ
□Q┌x╝□)─c□J┌ùóûJñ:╗▐¬"┌TΘ□µÄ²║╜┤⌡ùô╪□╡%Rü╟□µ╤□4^1V□»□»ïÆ¢É║ñ%"▐┼┌□«°¡≤≥

ôƒú╔ì$Ñ±≤xiUX+½ùF ô«°┼╢□X»Ñ│╛ εF□│>□HJ□├(┴┴ Ñ-
□▒O─╢╤□ùú□║èz½É□╓╞*ñ>k½êB+])j>≈≥A▓ê□æ╟╔ìd6√□L&Æ1d□ÖJf┼╜¢Å8o⌡~ú⌡ë┼≡√□²x□ÿ□
╫┬X$┐ç└┘h'╖Qµw┤±w·*╤Ω□ñ5Θ┼╓7ï²╒g[▓9i╞÷«√Γ▐σ▓╧c╝Q9«7~ï┌UÆC□╓█╙┘▀PR@₧
πXn□╓d·B6_╧o╙SA┤)X

╛ä6⌡Åk╢bh▌Θ¡4vïV╓e-JfF&│=ó ÷∞d╢ç╫b·ûóg«·╣g■╟┌é╟öe╞□■¢╡"µ╧B□4□

┼r▄¬▀╥ú~giu▀≤h{s╓·┌╟ÿ┌╟¬.$-
-e┴ñXÄ╗UjSò8¢~╗6─₧H□√%╛ñayφ▐SF╨Wï╧dvT¼¿σiâ╡!┌Pmÿû»
╫Fh≈□■{τßí□í√C□íçBEíçCπ┤□ó

>·«□ïo"±úW∞ ▒╝■□╚úÖz<╝ò▓□«┐D£9·æ┤«¿²/ⁿxΩ┌∩·û□ ` HÆΦƒαƒHBf▓y#íµ□s
óéJ■╣`I┘ot□òm≤≤)â?▌₧┐□□-=µ|Kq▕#s∞┼ƒ½█≡╝╥ß╤YK_═ê$bƒ₧OI□=[┌╟D¬÷+K(┴╓Ω
!J¿□╫╟τ[F²r}|╛Ñ4<ÑÅ╧╖£F°⌡±□_□o═el/-E╞Æ
°uⁿL±√°┼d□YM▐$δ±K⌡╥_⌐ï▀ÑKI□▀□.cπ├tⁿ^(÷¢æ]A=X5xS≡Äα+┴≈â╟≡□°±íσíuZ[¡¥
╓Ω╤╥ô⌠Mö è╫e. r
□╞□√â╡α`p□╕╥\HΣán>╟X╒|₧±&Σ∩□_□▀7ƒe<╞□Ω╤ù□í┤╨x╛lh9f'┤ÄSk╦°kφ└ÄⁿS-
ïτ⌡$p□'╜å═╔GR-v

Hf╟π·l+╡ΓV|αPα□ ═┐┘╦□┬╓ⁿÉ╣╨|üÑGbi░»H▌c)¢kí╣ê ╖èÑ/Æ╥□▐ⁿ█sYδ¼±{É+□>"}ëòQ+□2
□□%á□m□┐╫â#gΘá⌠¡⌠â⌠ô∞û╜▓O╓Σ░\E«εjΘεδ╛╧¥ε╬t□v□uτ½ò╒*jU╡ÜZ]MVk¿)j-
⌡&╡Äz▌zï┌@╝U╝M╝]m¬╢P{¬≈¬² ╘

uáÜúµ¬C╒|uä:F-Tï╘±j▒:I¥¼NSSg¿□UƒTƒR-
╒VƒVƒQτ¬≤╘┐⌐╧¬╧½ï╘┼Ω□uÖ‖B]⌐«R╫¿k╒7╒uΩ[Ωz⌡muâ‖Q▌ñ╛°nV╖¿[╒□Ω~⌡ÇzH=ó~Kxör╖öε

¬?zNV╕ì¡ò[Ω)⌡Sû{NThT╣ª▓╘▀≤□▐╜╨▀╗°¿╨≤╙Q■=êràr
|τ≈╝|9σ□rÅ╕ww╧Yl Θo╙wr-
╣Ä\Å}┌öMKò█╔¥Σ«r□·>9K╬M∞Æ8Ü}~╞WΓcσ_¼ö≥»z°┐□ùù□▒Z╧ⁿjz╩½9k[·WΩΘ»─âσ_l]╬≥≥N(
bδ\■╒⌡L/∩≤σ_¼ù╨┐zß⌡≤√√Nye░W╓Y^╣gzyOû□%·NyÖ°╝¬ö□ë⌡i╡□%░¡ƒ"▀
▀└.]Ω╧uë$▀"▀┬lh(7$.╣Ö▄î(r+╣□q╤mσ╢─#╖ù█ô□╣|▄ÖTÉ╗╔▌HE╣°▄ô$╩²Σ~─+□É□É$9O╬#ù$
vM∞J╘─1ëc╚Ñëπ□°□_b$±)R)qnΓ2R5qEΓkññΓ?□□ÉFëç□Åæ~▐æ▐±$█[∞¥J□╝3╜╧æ▒▐à▐Ñdå≈5∩
ç─≥n≈n'+╝;╜;╔╦▐]▐]dÑ≈□∩'Σ□∩°▐O╔½▐╨fæU▐/╝_É╒▐/╝_Æ╫╝_y┐"k╝□╝□╚δ▐C▐Cd¡≈ê≈□y
├√SÆçlH≥'□dsR8Θ*▓#ΘΩñd▓W¡¿z╔╫ΩÑ¬ƒ□R
5îxlⁿê▐▀┤Wù╙^╜N{▒πrΓ¼─9ä$«I|ïTLⁿ▐ïτ╚|╜ñ□c=0òæ∩}ê2ε□üⁿÑ╚≤¿Φ─╖°l╞□w╝≤σ·□èπ]
ó∩□÷╛╡o□°ÿ╞╟sÆ╗"úΓk┴ª╖
<Z~□;≥6fπ═╓h}/6╛╟bπN~«bGM_U▀╡╛□_□_]▀═╛z╛[|⌡}╖·╥|▌┘╥U/╣ü±Z0□¼□╓□o□δü╖Ç⌡┴[┴
4░;#Åp°bⁿ▀å]     t├□£□▓1·□6■─
□╤╞╘3╢ó⌐»╒o╨Æª`½S[%%æçñ╔RDÜ)9Rë⌠£┤XZ!¡û▐ö╓K□Ñ═╥6i╖⌠╣⌠╡⌠ì⌠âLdÅ£$□dC╛B«*'|¡
XùφMΣûr□╣□█
z╤Θ≥@y░<B-#□╔□σ⌐≥π≥,▄═╢P^"»ö╫╚δΣ╖╤εg█'-
öÅ╩╟╤▌╧V═U├uú½^┘□m¥\=p□WÄk¿½└UΦ□τÜΣÜεz┬□u=q-p-r-
s╜ΩZδz╤╕□≈┤}∞┌╤F*ⁿ₧╢ôèó$*>EW*)UöΩJèR[⌐»4VÜ+¡ò÷J□ÑùrƒÆÑΣ*·╨(e¼2AÖ¼D□■─
╛µ«╚¼!l+0ô|m|∞¬═╫▐ùF«≤u⌠u$5}▌|=Ièn─_orú»»»-
⌐φ╤≡eæz╛ü╛l╥╨7╙ùG□·å°åÉ█}├|≈ô&╛æ╛æñào┤∩□╥╥≈áo,╣╤WΣ+"w√╞·&É6╛I╛╟I;▀L▀□╥├WΓ
{¥d□┘]±ÆZ╪╬£□□½ü╫Ç╒┴k┴d≡:░□X□L□»□kü7Ç7é7ü╡┴:`]≡f░-x
Xl□6□o□□ü╖üì┴█┴&`S░□x□╪□l□╢□∩□[üwü¡┴6`{0
∞□v□╗â=└₧α=`o░/x/x╪□╞□3┴■`□8□╠□□ü╣`-8□□□µâ├┴□α²αHp48□|□|□□
□üπ└±α□p□°(8□£□N□°ü╨╨╟└□8□|□£     >□╬□-0□┌á□>
╬□ƒ□τÇ%α<p>°7p□°,°□°<╕□|□\□╛□.□_□ùÇK┴eαrp□°2╕□|□|□\□«□_□╫Ç»s▓cσ5■ú■∩ⁿ╟ⁿ▀√
□≡ Φ?ε?ß?Θ ╔ Ö s □■}■/²√²_·┐÷≡-
,¢v╚□╪□$6═╝├ln╢0[ÜwÜ¡╠╗╞╔µ▌ⁿ?hσ«hèⁿÅè+Ü+D}█²;ⁿ;²-
√w·w√?±∩±□Ω▀δ▀α□╠┐╤ «□ô ?■≈ⁿ∩√7√?(¢╢┼ í□klÜ┘╜╝╒ld▐f66o7¢ÿM═f┐X-
□z=5Γ,tε+ª╥i▒+°╪┤▓δ'▄ùTUJ■σ⌡
╚■φα

p'°1╕
■
~□ε□?□≈é□└w└ìα╗α&≡?α{α√αf≡□p
°!╕ò╙l□▌□6□o□□â╗âM└ª`|ï╨_∞Ω□ⁿ□ⁿ□<□-□□üç┴#α7α╗£■┴α□p(8

╠□çâ#└√┴□p$8□,□'rÜ╓αu`
░&ÿ□^□╓□o□y

½╪│Pb▀?╣ⁿ=²╜²}╪g─▀ô╤σ∩═¿░)σFWⁿ:╖ttÑuΣ╫╧ZWN1èJ┴7╜°ÅñNïδ¼σ≡√"5▄°╨8îù╟∩├c|□£
─ªπ▐I
wá2≥yp°.π# ÅædÉèñ·i⌡≥τi
□Σ□□□□□┴┐5╙□□□8Ü╖╓°□≥ú└□0□▀□≡{#┘ö⌐α4V□Åä6@πWêr □kΘ┴»åÑ╨│±π╔╨
|<╓ª╔■<\M₧└7╚╗°╡c∞;IÅⁿ=≥ⁿif│╚]ål╕

┼p□-úéQ╤H4╝<B□Å□f□ìÉí□║a□aπ2úÆq9Å□fT1«5Æìδî□FMπzúûq□Å□╞πâ±Φ`<6□Å

╞πé□w□¡î╗î╓╟▌F□#┌hk┤3┌□iF□úú╤╓Φlt1║å=ßäpàp┼pb8)|IX
_□÷à²ß@8x╤ε│½E┤_√l<┘G√╡O

20─╢$δ▌

╪Æ▀@OlÑ:°µv□~
ε?w$□6╖ ▄q_Xi*Q□»µ0σQú□<k≡┌`¡xc╕□■<ÿ└τµ1n─╙+K□â╬>ÖNÜùEj╪¬}ñm╙╣k;┤¥┌╟┌.m≈)
╧q\á=½=°=»-╘^╨□i/jï╡ù┤%┌RmÖ╢\[í╜¼¡╘^╤^╒Vi½╡╫┤5┌'┌-φSm»÷║÷Ö÷╣÷à╢□1
╛èï□qD√□æ ╓joÿ█╟φx▐║DÜùEƒx2O3≡T`V└□D□vαΘ└∞á□4┘ÜVbδZ%xu░^░~≡▀┴
┴ì┴M┴ □▀
~□▄□▄□▄□▄-▄□<-║"T9T%tM¿F¿f(=4:4ì⌡τ

╤úx■ ·UèδY■$□-┐bê6╧|=\c■╥ⁿ□▀┴╗±,°□R□▀╢‖Y□mc}≤ë╣ì╕┤═■<╢□║┤m"▌-
╥Ob⌐□¢╬#┌_N·Æ□R@╞▒>ÅÆ□d□YK6É¡d/9LNJëÆ.UæRñ·Rs⌐╜╘K╩Æ≥Ñ▒∞╩h&╗*è┼□ @√Ç□╢ò>b>
m╫v□à⌡εQRæ⌡█

và╟>τ5í6╖÷a\~k\■ú╕ⁿ÷╕ⁿÄ╕ⁿ╬╤ⁿ╟q·]q·Oπ≥{π≥ƒ┼σ?Å╦□□ù▀□ù 2.┐?. U\■δ╕ⁿü╕ⁿ┴╕ⁿí╕
ⁿß╕ⁿæ╕ⁿ7q·oπ≥G□╬¢q}b■▄'<zkUv₧ß≈hτ│δσq∞╜ 8█å%d!█è½╓:╢□7ô□lK~MÄ▓mΘæT╢5K∩╥n─
╢hΘ]┌9R>?o0âÄ□┬£▀A«èsΦ□æn□Θç"▌*╥ÅNqm╗Hwêt°H?□Θ«3;⌐φ□Θ°"▌+╥╧D·╣H┐□Θ>æ~)╥²"
²Jñ_ï⌠ÇH□è⌠ÉH□ï⌠êH┐□Θ╖"=Zn_qÖb╜b[Ç⌡~:;n»'¢╪╛▓¢∞c{╤□Æ╟÷ùÇ□f{L▓t#█gÜH¡╪^╙MΩ
╤÷¢╧R□█s&J╚╛3[Z -ûVJkÑ⌡╥&i½┤[┌'□û~Ée9æ
Φ°╖╧╔≥ìr}Ö ▀óûx^!"▓αYâ┐ⁿö┴≥╧□î□┬α*}□□╩`┘ô□╫╨╖Φ╗t
q□&╟╓,0EñSE·îHτêtn,
ë╧COè⌠{æ₧□ΘI╤âë"UEz⌐H7ƒ▓σ╛ïÑzÆH
æ╓□i}æ6□iCæªè┤½H{è┤ùH□êtáH│EZ(╥ë"□δ»ï⌡╫gë⌠Eæ«□Θ&æ□│ua

╡E:OñïD║\ñkD·ûH▀□ilÅ┬}ÆÑqç┤╡π┌
φñ÷ôNtIùuù«Φn▌âhD□⌡D▌ïÿDj\TóÉ«±╙DµN≤cs□3ªΩ┘╧Teg¢s£kp₧·
╓▓H>ò⌠╦⌡+⌠+⌡╩·Uz□²j╜¬■□╜Ü~
Γ√$δ╫Θ5□στ·╕8?u⌠║<╓Å╣█ⁿ─▄âûòFφZ□x!░(≡b`qαÑ└Æ└▓└≥╨╟É□èål¡óxΩ□Å(□êï)─ƒ ┼ƒ²╡
╓│□O ¬Aé┌â┌Xφ!¡H{X□°ì╫&h┼┌Dφ□mÆ÷¿6Y¢óM╒ªi╙═¡ⁿ╙□{R
ós□0Æ]┼ »üÿ9äσrHm²'─╨ö⌐ï*╘M=4üV@4M/M*□Q3êÿÜ:Ñ╘á&
╙╦h%D╫╝ÆVÄÅ░⌐╜⌐²][°²□□:7j∩j¢┤ ≡H¥ⁿ█▀╨eⁿ)δld▓ÜT╫╓k∩░s╕╟«╪Z╨ßt□╜ƒ□╨æt□□M╟╨□
h!}ÉÄÑ□╤"z
¡N»Ñ╔⌠:Zâ╓ñ)⌠zZï▐@oñ7╤┌┤

¡Koª⌡Φ-┤>+∩_┌█┌┐╡
┌;±⌡╥T─ⁿlO╙h□┌ævó¥i□D ∞N{─G□┼=Xå┘Å ╬ëT

]┬ƒ[éûVak⌠OR□±êRîm╞6╥─8a£ MM╓
ñÖ┘ ╒∞Jε0s╟□v%"│δ╨□┤□mHoÑìΦm┤1╜¥6íMi3z□mN[╨û⌠N┌è ▌E[╙╗i□ ·0□G╟╙
┤ÿNñÅ╨I⌠Q:ÖNíSΘ4:¥ ▌ïêñΘ4âf╥■4ï

á□□¢t□═ìÅOj∞4ë□6╙·/¿╬π)÷▒æΦc4BgP~G╚╩╩╞Ä}≥W ·□}Æ╬$±╧2æÑcΣ√▓'╗≥ΦG<ΩW□╜⌐▐L┐Co
«╗╨[ΩwΩ¡⌠╗⌠╓·▌z¢3╞╗□ªτδ├⌡□·²zü>RÑÅ╓╟Φ□░#x[╜¥▐^O╙;Φ□□K⌐P□P-
½?ñ□Θ□ï╪X]╪Q╛¢▐]∩üc²=zo╜O\ö▒

=□╤⌡-╤'ΘÅΓ°?Ufª OÅïú⌡ä■$bæ┼╟□s⌠°⌡┘·3·□}«^ó╧╙τδ□╙□Φ╧"□┘B²□}□bæ╜□□ì∞e}e╣êd»δ
k⌡7⌠7⌡┐δδ⌠□ΦoΘ ╘╫δ ╥▀F£▓w⌠ì·╗êV÷^\╝▓¡·Gσbûφ╓?╤≈ΦƒΩ{⌡╧⌠╧⌡/⌠}·ù·~²+D·:¿-
╥□#▐╫╖q□┐~╘Å#Ω╫□·:]K▀áo╥┐╙u⌠-
∞▄÷O║₧■ï╛M M7╨wΦFv«█D C▀ú∩╙═⌠□║à~H╥╥ÅΦ6║¥εá;Θ╟µ°µ^≤3sƒ·Ñ╣▀ⁿ▄ⁿ╨ⁿ┬ⁿ┌<`-4□Öç
=#µ7µ╖µQ≤;≥┐?Ky%Θ÷_╪p·╢?≈ûìÅÆv▐=*zΓ+╓
Ñk~■Oü■s¥ □Xτ╥∩╦

|╫└ƒ╒-√s□╒└¢O²╓M<æ;~

F<ú√τ∩□Ω□┘ x▓u∞Næf┐∩:⌡Y╓|╛0X[,├┘
╝□∞}j√î*£■□÷i□F>.pú╓Üg«»▄≤öσ╨■└eî'85□╝ÆS∩ N

≡{¢gæ╗~■NU¬┼«╗□▓½□■√╪

∞║á7╗÷□╙«

FHcñ"vm0Uz\Ü┼«

µK
Ñ%∞·`ì┤Nz¢]!læ vH{╪5┬AΘ¿t£]%TÉU9─«□*╥╒Σ□∞JíPts▄Hn&╗Æ█╨¥Σ-e╤3□ΣBy£<IPts.?!Gσ9≥□
yæ╝L~U^+┐%o╕X▀u□<ⁿ7πü□░□X□L□ô└K@□╝□│â□0□j □
╨□├ασα□αò`e≡*≡j░*X
¼ ªÇ╫â╡└□└¢└┌`□≡f░-╪□l□6□o□[Ç-
┴╖└╓`□0□l□ªü□└Ä`'░3╪□∞□v□╗â=└Pts`/≡-░7╪□∞ï Φ-
□┐□□□□□Åâ'┴ƒ8├□ö@□tü□Φfⁿ╙Θ39□ü╙□8□ü╙□8□ü╙□8□ü╙□8□ü╙□8□ü╙□8□ü╙□8□ü╙□8□ü╙□8□ü╙□8
□ü╙□8□ü╙□8□ü╙□8□ü╙□8□ü╙□8□ü╙□8□ü╙□8□ü╙□8□ü╙□8□ü╙□8□ü╙□8□ü╙□8□ü╙□8□ü╙□8□ü╙□8□ü╙
□8□ü╙□8□ü╙□8□ü╙□8□ü╙□8□ü╙□8□ü╙□8□ü╙□8□ü╙□8□ü╙□8□ü╙□8□ü╙□8□ü╙□8□ü╙□8□ü╙□8□·╙Θ3:
m┴i
N[p┌é╙□£╢α┤□°-8m┴i
N[p┌é╙□£╢α┤□°-8m┴i
N[p┌é╙□£╢α┤□°-8m┴i
N[p┌é╙□£╢α┤□°-8m┴i
N[p┌é╙□£╢α┤□°-8m┴i
N[p┌é╙□£╢α┤□°-8m┴i
N[p┌é╙□£╢α┤□°-8m┴i
N[p┌é╙□£╢α┤□°-8m┴i
N[p┌é╙□£╢α┤□°-8m┴i
N[□:}F°m8m├i□N█p┌å╙6£╢ß┤
°m8m├i□N█p┌å╙6£╢ß┤
°m8m├i□N█p┌å╙6£╢ß┤
°m8m├i□N█p┌å╙6£╢ß┤
°m8m├i□N█p┌å╙6£╢ß┤
°m8m├i□N█p┌å╙6£╢ß┤
°m8m├i□N█p┌å╙6£╢ß┤
°m8m├i□N█p┌å╙6£╢ß┤
°m8m├i□N█p┌å╙6£╢ß┤
°m8m├i√O°╧Φ┤□°□8φ└i□N;p┌ü╙

£vα┤□°□8φ└i□N;p┌ü╙

£vα┤□°□8φ└i□N;p┌ü╙

£vα┤□°□8φ└i□N;p┌ü╙

£vα┤□°□8φ└i□N;p┌ü╙

£vα┤□°□8φ└i□N;p┌ü╙

£vα┤□°□8φ└i□N;p┌ü╙

£vα┤□°□8φ└i□N;p┌ü╙

£vα┤□°□8φ└i□N;p┌ü╙

£vα┤□°□8φ$^{n}$╤¥╛□OqcuN!$^{n}$

□N□tâ-░□X□L□╜α%á□ ·@?□□Cá□Ωá  å┴╥└Jασαò`e░□x-ÿ

^□╓□ké╫â╡└□└¢└┌α=`=░>╪□l□▐□6□¢éwÇ═┴;┴Vα]`k≡n░
ÿ□╢□█üφ┴4░□╪□∞□v□╗Çê□F▀å□@⌠p□=□F□çô@⌠p□=□╛□D?ç╤╧ß□╚{√O°╧Σ┤□°-8m┴i
N[p┌é╙□£╢α┤□°-8m┴i
N[p┌é╙□£╢α┤□°-8m┴i
N[p┌é╙□£╢α┤□°-8m┴i
N[p┌é╙□£╢α┤□°-8m┴i
N[p┌é╙□£╢α┤□°-8m┴i
N[p┌é╙□£╢α┤□°-8m┴i
N[p┌é╙□£╢α┤□°-8m┴i
N[p┌é╙□£╢α┤□°-8m┴i
N[p┌é╙□£╢α┤□°-8²ç-
O_$°úp:□°úp:□°úp:□°úp:□°úp:□°úp:□°úp:□°úp:□°úp:□°úp:□°úp:□°úp:□°úp:□°úp:□
°úp:□°úp:□°úp:□°úp:□°úp:□°úp:□°úp:□°úp:□°úp:□°úp:□°úp:□°úp:□°úp:□°úp:□°úp
:□°úp:□°úp:□°úp:□°úp:□°úp:□°úp:□°úp:□°úp:□°úe▀çç±}°éÉ┼ƒ╬nd□╦Éf░T"╣ñ

ÖC□ÉEd□"?╛Uεù¬G╓qI)╖○╕ÜTC¬FûI╡╓f6╡í╘

╧sε┬÷ìL)ù?²ÿ²□▓26K□╪■±äΣ░╣xn╛┤HZ┴û^═ {□x&0ùìóτ□û≤;LBOô□■□q9■□æé =╘┬ -n┬ -
j│ûùα{í□-⌡ôq□X□

□úb╝ûΓ]>ΦÇ# └┘α╙ÿkÿ(╦□╦╪ó/f`Ω≤`!°,>□-µ╧□≤ED║□i-Åàè⌠A╠╡□□ìicy╝M╘1C╘<□O
τs━j□âΘàΓ≤⌐"¥&╥□<V)ZÇ≈▒¿₧H▒na□∩b╤<SHê4"═pÅp7╥ùdÆ□Dè(━]c▒╪₧Ñ█ÿ▀/qop
!┴£αpnH░/Å≡╩ê╪┐A>□µ∩□öÑ▒⌐9`□p□8□□╦ÿì□▓E ┘X2[öÉ-J╙F ┘(!□%dúälV□Å□ôHÆI-
Rù4dδ╤è┤%¥H□rZ;18Ö╡vj0B▄∞]1Å&╟8□Σ⌡□ïzïì'□°α≤)°|□>ƒ">ƒ┬>┐Xws ■√┘Σ└T╢ƒ²-
{g □9z'xW
├0äa□S═╪«*ù½╩σzs╝┐W╣▐\»
,a !äπZ□j□«â╕□!□Γ□ûσ□╫ïZ□Ü#□b□å%äσXûeß8ª┼í□B-ä□b√X╘BlB-äZ,╦r□a
9√□≥╢╩░èⁿ╦τ)╗-√)ⁿ⌐╟e≤}ⁿ|√ßq~Dùd□ÆH«εeq²î╔kF □╧τ□■[⌠o╣~÷wΦ▀ü~÷

|╫ƒü~÷

⌠|gáƒ=□²∞□Φg╧└·â_j□□□l□□□ⁿsPδ√w√·Γn¢»J0¢5W■□`'α_éw>╗½ ¥╗z□wσ"(┐Ωg╧ε ·┘3╨╧₧
ü~÷îδgƒ▀╡±·]╥■»9ⁿ

ㅑㅇㄱㅕ

░╛≤ε²╛╗≥┘]·9Φgⁿ'□╦w²∞┘]?{□·┘|□1kεo?├╚Å╩ⁿM╖┐δi□É□^äë□▌.ÿ□Ω∩ß┐τⁿⁿ°╣O√O·ⁿyZ

┐□Gß

µ°□α≈□□□8□°}└1@╬._ n⌐-≡
└┐□£□∞□$^{n}$ƒá.ÿ¥□>┐πw□?□$^{n}$-α□□G□┐□8□╚╗t~╫╥9hΘ□┤t

Z:□-
¥âû╬AKτ_═⌠²╗"¡·c·□₧?₧?æ π|ε|ÿ □Φ▒_≡¡qΣ÷£ #░µG`═Å°y─┴sñv╚╦¥σ~╗╧■╥□□₧X╙_µ╬□
╧≤_@pK?□√° ?δ8 □£□~□÷■□■╗╤±{Çcⁿ

□áεùáεùwu┐□u┐□u┐□u┐□≈≤Iê□g╫V0&≈      ╘□
□ ⁿs7╝ ▀|□τä½┘├╧ó╠╜╗□▌r█,rkf╕O¢      ≥÷B·W·WÉ$ : τ╨╨ⁿ_HD□╨τ▐àd|ε]■I£ⁿƒ±3┤τ

╝ ·3ε╙q╦\╔/}p

░□≡s└/@¡∩âZ╖`δ[≡è▀·÷nδ[░5°O□╟V└╧□┐□╡╛□╫F═╪W²▌6⌡α=~I━□á~πwªÖ+;9
ⁿÜ»z━»gô■⌡±╪σ╧CΦ-X≤

:τÄ╩»F-â3»îì&▒JX(ƒ[Æ≥τSÄ □p

░□Ék_"□»~□°□αf□■□~ⁿ▌W≤gâ▓·«ⁿⁿ«□╜+·╜Z@
û╗╫cÇ=Ç|
□≡Ω□Ç_□■) ▀éσ«□╦]
û╗□,w-X╛ÜΘ²wÄ2hù|&ΘÉtJ■D┬²rJ■□ =π8□╚ vⁿ1X≤╟`=sk~≤i□«ÅIµ└∩σoîtÉ,▐δα╕└ □▀√

╝√δ-■□£□□■╡▀□c□ a¼é0VA□½
îU□╛╙┬Xà╧í▀»▒□:Φ□≡ ë_▐╫╫■j□Ü_?╧>╬▀□σ'≤╥╢½b>≤±╟αyÄ<■Θ

pu₧α∩-8v□■□≡╧□g╕φ□╙□╕[¬□ⁿ.`#`□αO□ =αgÇ=`╗∩ Ö_┬>╜{µÜ┐Vc└leÑ▄]F
─▀}|(□ùⁿH≥c╓ñΣ/$SÆ .ÖF 7w┼5┴▀¡≥□╫j□|╓□░⌠S░⌠▌╗Ñ-
é□d═□σƒpWeⁿîÆ□ε·φ_■ ßù │·Ω«b

Üéf·'⌡∩a≈p□║G▄3@∩▀ú∩╤P-╚à⌡ ?&ò┐□µ°□`□`=αw□?□∞ÇD≈□αU'α(α□`α3@ε╛ì▀□□-
╥ò▀╢█ ·┐≥K╪°└Σoì±²σ|□≈,á}
╪ùà█□?≤▒-jΣ«εG┼n±╓{u∩▌f

╛○~□=≤~µσ□O│¿¼ò□+><═ε·□■╡┼£Æ£╖uΣ□Σ╫~£ⁿ°
»σô▄O&╣≤├n~ú$S2z╧}o½á«α÷╚Aä□Äµ=Σ□▄ò7□▓ôê└╝─Z¿□⌐Dε#□Éç╚#ñ□iG║É╟╚S«╞02åL"3╚
<▓ä¼ k╚□▓ì∞"√╚!rî£"□╚5□íÖh

╨¥□P□%Q□jE▌(â▓h□-A╤╤*┤□¡C□╨&┤□φ□3[≈íâΦ□:ÄNí┃Φ□║î«óδΦ&║âεí□Φ□zé₧íùΦ
&╞▓░\L□□í]Φc⌠):Ç

úcΦ$:â╫úKΦ□║åná█Φ.║Å-ó╟Φ)zü^c□ûëσ`▄┌□├1□3`V╠ì1□ïÑ▒□¼□½┬j░:¼□k┬Z▒

¼□δ┼·░Al□□╟ª░Yl□[╞V▒ul□█┴÷░□∞□;┴╬░K∞F*ûfIsÑ□)&%ñööû┌Ñ^i□*┬f░yl [┴╥░
l□█┼÷▒C∞□;┼.░k)$═öµHa)"┼Ññ╘ ╡J▌RF╨J╙╥□i╣┤JZ#¡ô6H¢ñ¡╥

i╥┤W┌'□öÄH╟ÑS╥YΘétY║*]ùnJwñ{╥□Θæ⌠Dz&╜ö▐╚─▓,Y«L"├däîÆ╤2╗╠+
╔Γ▓BY⌐¼BV-½ò╒CE╥]Θ╛⌠Pz,=ò^H»eÉ,Sû#âeê

ùæ2â╟*s╤□□+K╤Jdo▓*Yì¼N╓ kÆ╡╨:d▌▓^YƒlP6"□ùM╓fe
▓e┘¬l]╢)█æφ╓

dG▓□┘Ö∞Rv#□╦│Σ╣rë□ô□rJN╦φr»<$Å╦
σÑ≥□y╡╝V^/oö7╦█Σ¥≥-·□y?T$çΣÖ≥□9≈□!╟σñ▄ ╖╩▌rF╬╨╙≥□y╢╝J^#»ô7╙¢Σ¡≥

y╖╝W▐'öÅ╚╟σS≥Y·é|Y╛*_ùo╧wΣ{≥□·æⁿD~&┐ö▀αb<
╧┼%8å□8à╚┐□≈Γ!<Ä□ΓÑx□^ì╫Γ⌡x#▐î╖ß¥x□■□∩╟ç≡Q|□ƒ╞τ≡Eⁿ9Tä□p+ε╞□£┼╚x
^ÄWß5x□▐Ç7ß¡x□▐ì≈Γ}°
>éÅπS°,╛Ç/π½°:╛ë∩α{°□~äƒαg°%~ú□+▓□╡□ë□S□□JA+∞□»"ñê+□□Ñè□E╡óVQ»hT4+┌□¥è-┼□
E┐bH1¬ÿPL+µ□ïèτè□èùè-┼+┼k┼□┼[¿HQ«¿R╘(Ω□
è&E½óC╤¡ΦU⌠)□□#èq┼öbV▒áXV¼*╓□¢è□┼₧Γ@qñ8Q£).□7äÿ╚"r
ü□□A□4a'╝Dêê□àD)QAT□╡D=╤H4□mD'╤C<!·ë!böÿ ªë9bæxN╝
^□[─+Γ5±åxK╝#╬ë+ΓVÖí╟VµAED□╤M⌠□}─
1Bî□S─,▒@,□½─:▒I∞□{─□qD£□g─%qú□+│ö╡Jë□S□JJI+φJ»2ñî+
òÑ╨□e╡▓VY»lT6+█ö¥╨-σ□e┐rH9¬£PN+τöï╧τ╨□╨ù╨-
σ+σkσ□σ[σ;σ╣≥Jy½╨Pe½≥T□*ÖJÑ╥⌐╟*°╨□□)°ö│╨□σ▓rU╣«▄Tε(≈ö□╨#σë≥Ly⌐╝QëUY¬\òDà⌐
□□ÑóUvòW□R┼Uà¬RUà¬ZU½¬W5¬ÜUm¬NUÅΩë¬_5ñ□UM¿ªUs¬E╒s╒
╒K╒ûΩòΩ╡ΩìΩ¡Ω¥Ω\uÑ║Ug¿│╒yΩ□╡L¡Rδ╘f╡SφWG╘Iu▒║L]⌐╛»~□□⌐vT{¬□╒æΩDuª║T▌¿┼Ω,u«
Zó╞╘äÜR╙j╖┌½

⌐πΩBu⌐║B]¡«U╫½□╒═Ω6u°║G²D▌»-RÅ¬'╘╙Ω9⌡ó·╣·à·ÑzK²J²Z²F²V²N}«╛R▀Æ□d6ÖG□É2REΩ
H3Θ$²däLÆ┼d□YI▐'É□╔Gd
┘NvæÅ╓°Pæ·å□ôYd.)!1Æ )Æ&φñù

æq▓É,%+╙j▓û¼'□╔f▓ì∞$(╙'d?9DÄÆ□Σ49G.Æ╧╓□ΣKrï|E╛&▀Éo╓wΣ9yE▐j24┘Ü<MüFªQit□││╞
⌐±k"ÜñªXSª⌐╘▄╫<╨<╘<╥┤h┌5]Ü╟Üº Ü□═░fL3⌐Ö╤╠kûá"
Ñí5vìW□╥─5àÜRMàªZS½⌐╫4jÜ5mÜNMÅµëª 3ñ□╒Lhª5sÜE═s═
═K═ûµòµ╡µìµ¡µ¥µ\sÑ╣Ñ2¿l*Å*ádöè╥Qf╨I·⌐□òñè⌐2¬Æ║O=á-RÅ¿□¬¥Ωó-SO⌐□jÿ□ú&⌐□j₧Z
óV¿5jâ┌ªv⌐}Ω□*óJ⌐□¬Ü¬ÑΩ⌐F¬Öjú:⌐-Ω ╒O
Qú╘□5M═Qï╘sΩ□⌡Æ┌ó^Q»⌐7╘[Ω□uN]Q╖┌

m╢6O[áòiUZ¥╓¼uj²┌ê6⌐-╓ûi+╡≈╡□┤□╡Å┤-
┌vmù÷▒÷⌐v@;¼□╙Njg┤≤┌%φèvM╗í▌╓εj≈╡ç┌cφ⌐÷B{¡âtÖ‖□¿H█ªφ╘÷hƒh√╡C┌QφävZ;°]╘>╫╛
╨╛╘ni_i_k▀h▀j▀i╧╡W┌[]å.[ù°+╨╓t*¥Ng╓9u~]Dù╘□δ╩tò‖√‖□‖ç‖G‖□]╗«K≈X≈T7á□╓ìΘ&u
3‖y▌ÆnE╗ª█╨mδvu√‖C▌▒εTwí╗╓C·L}Ä-╓#z\OΩ
z½▌
□Θ&t╙‖9▌óε╣εàεÑnK≈J≈Z≈F≈V≈Nw«╗╥▌Ω3⌠┘·<}ü^ªWΘuz│▐⌐≈δ#·ñ╛X_ª»╘▀╫?╨?╘?╥╥Φ█⌡]
·╟·°·□²░~L?⌐ƒ╤╧δù⌠+·5²å~[┐½▀╫ΩÅ⌡°·
²╡□2d□r

░□1α□╥`0X
n□c`
iCëí▄Pe¿üè⌠[·W·╫·7·╖·w·s²ò■╓Éa╙6Σ□□

2â╨á3ÿ
Nâ▀□1$
┼å2CÑß╛ßüßíßæí┼╨nΦ2<6<5

□å
cåI├îa ▌░dX1¼□6

█å]├╛ß╨pl85\□«ìÉ1╙ÿcäìê□7ÆFâ╤jt□□#kL□Kîσ╞*cì▒╫╪`l2╢□;î▌├^¿╙pe╕5f□│ìy╞□ú╠¿
2Ωîfú╙Φ7FîIc▒▒╠Xi╝o|`|h|dl1╢□╖îÅìOì□╞aπÿq╥8c£7.□Wîk╞
π╢q╫╕o<4-□Oì□╞k□ó3Θ□□ª□□°I┌@[i7═╩,¥ªKΦr║è«íδΦ□║ënÑ;Φn║ùεú□Θ□z£₧ógΘ□¿êV╤:┌
L;i?□íôt1]FW╥≈Θ□⌠C·□▌B╖╙]⌠c·)=@□╙c⌠$=C╧╙K⌠□╝Fo┻█⌠.╝O╥╟⌠)}A_¢
Sª)╟┫⌠Öp□i2ÿ¼&╖ë1▒ª┤┌─Tn¬2╒ÿΩL
ª&S½⌐├╘mΩ5⌡Ö□M#ªq╙öi┌┤`Z6¡Ü╓M¢ª□╙₧Θ□*2□¢╩LòªVª□ªçªGª□S╖⌐╦⌠╪⌠╘4`□6ìÖ&M3ªy╙
Æi┼┤f┌0m¢vMVªC╙▒Θ╘ta║6CµLsÄÖ╖±4πf╥l0[═n3cf═isë╢▄\e«1╫Ö□╠MµVsç╣█▄kε3□ÜG╠πµ
)≤¼y┴╝l^5»¢7═;µ=≤ü·╙|b>3_Üo,bK□Tdn1╖¢╖╠Å═O═□µa≤ÿy╥<c₧7/ÖW╠kµ
≤╢y╫╝o>4¢O═□µk
d╔┤ΣX╕¢
n!-□ï╒Γ╖0□╓Æ╢öX╩-Uû□K¥Ñ┴╥di╡tX║-
╝û>╤áe─2nÖ▓╠Z□,╦ûU╦║e╙▓c┘│□XÄ,'û3╤Ñσ╞*╢fYs¡□+f%¼öò╢┌í"╦ÿe╥2cÖ╖,YV,kû
╦╢e╫▓o9┤□[N-
□ûk+d═┤µX╣□p+n%¡□½╒Ω║2V╓Ü╢ûX╤¡U╓□k¥╡┴┌dm╡vX╖¡╝╓>δáu─:n¥▓╬Z□¼╦╓Uδ║u╙║c▌│-X
Å¼'╓3δÑ⌡╞&╢e┘rm□□f#löì╢┘m^[╙□╖□┌Jm□╢j¿╙║a▌┤εZ≈¡ç╓cδ⌐⌡┬zmâlÖ╢□□lCl╕ì┤□lV¢█
╞╪X[┌Vb+╖U┘jlu╢□[ô¡╒╓aδ╢⌡┌·lâ╢□█╕m╩6k[░-
▌Vmδ╢M█Äm╧v`;▓¥╪╬lù╢□╖╪₧e═╡K∞ÿ¥░Sv┌n╖{φ!{▄^h/╡W╪½φ╡÷z{ú╝┘▐f∩┤≈@E╢
█┤□▓g┌s∞░□▒πv╥n░[φn;cgφi{ë╝▄^e»▒╫┘□∞M÷V{ç╝█▐k∩│□┌G∞π÷)V¼}┴╛l_╡»█7φ;÷=Vü²╙
~b?│_┌o□bGû#╫!q`

┬A9hç▌ßuä□qGíú╘Qß¿v╘:Ω□ìÄfG¢ú╙╤πxΓΦw

9F□□Äi╟□TΣ└□ñ├α░:■

╞┴:╥Ä□G╣ú╨Qπ¿s48Ü□¡Ä

G╖ú╫╤τ□tî8╞□SÄY╟éc┘▒ΩXwl:v□{Ä□║æπ─qµ╕ t▄8┼╬,g«SΓ─£äôr╥N╗╙δ

9π╬Bg⌐│┬Yφ¼u┌;□¥═╬6g°│╟·─┘∩□rÄ:'£╙╬9τó≤╣≤à≤Ñs╤·╨·□*rª¥%╬rgò│╞Yτlp69[¥□╬ng
»│╧9Φ□qÄ;°£│╬□τ▓s╒╡ε▄tε8≈£□╬#τë≤╠yΘ╜që]Y«\ù─à╣□□σó]vù╫□r┼]à«RWà½┌Uδ¬w5║Ü]
m«NWÅδë½▀5Σ□uM╕ª]s«E╫s╫
╫K╫ûδòδ╡δìδ¡δ¥δ▄uσ║ug@E«&W½½├╒φΩu╛╡□]#«q╫ök╓╡αZv¡║╓]¢«□╫₧δ└uΣ:q¥╢.]7n▒;╤¥
δû╕17ßª▄┤█ε÷║Cε╕╗╨]Ω«pW╗k▌⌡εFw│╗═▌Θεq?q≈╗ç▄úε
≈┤{╬╜Φ~ε~ß~Θ▐r┐r┐v┐q┐u┐sf╗»▄╖₧

O╢'╧SαæyT-¥╟

□╣G▄πε)≈¼{┴╨∞^u»╗7▌;ε=≈ü√╚}Γ>s_║o<bOû'╫#±`-┬CyhÅ▌π⌡ä<qOí°╘Sß⌐÷╘zΩ=ì₧fO¢°╚
╚πyΓΘ≈

yF=□₧i╧£g╤≤▄≤┬≤╥│σyσyφyπyδyτ9≈\yn╜□▐1o₧╗└+≤¬╜:»┘δ⌠·╜□o╥[∞-
≤VBE₧u╧ªg╟│τ9≡□yN<g₧K╧ìW∞═≥µz%^╟Kx)/φ╡{╜▐É7ε-
⌠ûz+╜╒▐Zo╜╖╤█∞m≤vz{╜O╜²▐!∩¿w┬;φ¥≤.zƒ{_x_z╖╜»╜»╜o╜o╜∩╜τ▐+∩¡/├ùφ╤≤□°d>òOτ3√
£>┐/ΓK·è}e╛J▀}▀□▀C▀#_ï»▌╫□□y╧╜ù▐□ƒ╪ùσ╤⌡I|ÿÅ≡Q>┌g≈y}!_▄WΦ+⌡U°¬}╡╛z_ú»┘╫µδ⌠
⌡°₧°·}C╛Q▀äo┌7τ[⌠=≈╜≡╜⌠m·^·^√▐°▐·▐·╬}W╛[□å?█ƒτ/≡╦ⁿ*┐╨o÷;²~□─ƒ⌠□√╦ⁿò■√■□■ç
■G■□□╗┐╦ ╪ ╘?α÷Å·'²3Pæ≤□~╩O√φ~»?ΣÅ√
²Ñ■□□╡┐╓_∩o⌠7√█ⁿ¥■─ □□┐□╚?Ωƒ≡O√τⁿï■τ■□■ù■─ + k □ [ ; ╣ ╩□□╚□d□≥□□□Y@□╩□╠□
g└ê□ÆüΓ@Yá2p?≡ ≡0≡(╩□h□t□-□₧□□□├ü▒└d`&0X□¼□╓□□üφ└.T□ê□□□Ñüè@uá6Ph

4□┌□¥ü₧└ô@□`(0□ÿ□L□µ□ïüτü□üùü¡└½└δ└¢└█└╗└yα*p╤d0┘L-S└╚□□úc╟îô±3□&╔□3eL%sƒ
y└<d-1-L;╚┼<f₧2□╟03╞L23╟<│─¼0k╟□│═∞2√╟!s╟£2□╟u□éèÿFªÖic:Ö-µ ╚╧

1ú╠□3═╠1ï╠sµ□≤Æ┘b^1»Ö7╠[µ□s╬\1╖┴î`v0/X□ö□UA]╨□t□²┴H0□,

û□+â≈â□é□âÅé-┴÷`W≡q≡ip 8□□
N□gé≤┴ÑαJp-╕□■

ε□≈âç┴παi≡"x□éBÖí£□□BBxê

□áóαPp48□£

╬□□â╧â/é/â[┴W┴╫┴7┴╖┴w┴≤αU≡6ö□╨

σà□B▓É*ñ
ÖC╬É?□ %C┼í▓PeΦ~ΦAΦaΦQ¿%╘-Ω□=

=

äåCcí╓╨Lh>┤□Z ¡à6B█í▌╨~Φ0t□:
]ä«├P83£□å├H□□ôaC╪□vçÖ0□NçK┬σPQΦEΦeh+⌠*⌠:⌠&⌠6⌠.t-║□▌å3┬┘ß╜pAX□Vàuas╪□÷ç#ß
d╕8\□«

■□?□?

?□╖ä█├]ß╟ß°ßü≡px,<□₧ ╧çù┬+ß╡≡Fx;╝□ ▌□-
åÅ├°ßï≡u□èdFr"p□ëα□2bêX#ε□□a#ΘHIñ<R□⌐ë╘E□"Mæ╓H□T□~□>□_ào#□æ∞H^ñ
"ï¿"║ê9Γî°#æH2R□)ïTFεG-D-F-EZ"φæ«╚π╚╙╚@d82□Öî╠Dµ#Kæò╚Zd#▓□┘ì∞G

#╟æ╙╚EΣ:□E3ú9Q8èD±(□5D¡Qwöë▓╤t┤$Z-¡è╔Dδó
╤ªhk┤#┌□φì÷E□ú#╤±Φ□T□-
ê╨ó¬¿.jÄ:ú■h$Üî□G╦óò╤√╤□╤ç╤G╤ûh{┤+·8·4:□□ÄÄE'ú3╤·ΦRt%║□▌ênGwú√╤├Φq⌠4z□╝f!
6ô═aa□aqûd
¼òu│

╥▓i┤║ä-
g½╪□╢Äm`¢╪V╢âφf{┘>vÉ□a╟┘)vû]`ù┘Uv¥▌dwá"6┬&┘b╢î¡d∩│□╪ç∞#┤àmg╗╪╟∞SvÇ□f╟╪Ivå
¥gù╪□vì▌`╖┘]vƒ=dÅ┘S÷é╜ÄA▒╟XN

Ä!1<F╞

1k╠□cbl,□+ëò╟¬b5▒║XC¼)╓□δêu╟zc}▒┴╪Hl<6□¢ì-—ûc½▒⌡╪fl'╢□;ê□┼Nbg▒╥╪
T□{□{□kë╡ ╟║bÅcOc□▒ß╪Xl26□¢Å-┼Vbk▒ì╪vl7╢-
;î□║Nc□▒δ8□╧îτ—ß8□╟πd▄□╖ ╟▌ q&╬╞╙±Æxy╝*^□»ï7—¢Γ¡±Äxw╝7 ▌□-
îÅ—╟πS±┘°B|9╛□_Åo╞wΓ{±â°Qⁿ$~□┐î▀$—ë¼DnBÆ└□DééèΓ□±ß°X|2>□ƒÅ/┼WΓk±ì°v|7╛?î-
╟Oπ□±δ□ö╚LΣ$α□Æ└□d┬É░&▄
&┴&╥ëÆDyó*Qô¿K4$Ü□¡ëÄDwó7╤ù□Lî$╞□Së┘—Bb9▒ÜXOl&v□{ëâ—QΓ$qû╕L▄$┼╔¼dnR
Æ—ÆDÆJ╥I{╥¢

%π╓┬d)TöXI¼%6□█ë▌─~Γ0q£8M\$«ôP23ÖôäôH□OÆIC╥Üt'Ö$¢L'KÆσ╓¬dM▓.┘ÉlJ╢&;Æ▌╓▐d_
r09Æ□ON%gô
╓σΣjr=╣Ö▄Iε%□ÆG╓ôΣY≥2yô□°▓R╣)I□K□)*E°∞)o*öè°□SÑ⌐èTu¬6UfjL5°┌áóΣq≥4yæ╝NA⌐╟
TN□N!)<Eª

)k╨¥bRl*¥*Iò°¬R5⌐║TC¬)╒ÜΩHu°zS}⌐┴╘Hj<5òÜM-
ñûS½⌐⌡╘fj'╡ù:H□ÑNRg⌐╦╘MZ£╬Jτª%i,Mñ⌐4¥╢°╜ΘP:₧.Lùª+╥╒Θ┌t}║1▌£nKwª{╥O╥²Θí⌠hz
□╬ΣcÅ≡□|∞□╬ΓcÅ≡ç|∞□╬µcÅp

{ä?ΓcÅp.{ä?µcÅ≡'|∞□µG≤┴░äÅ=┬≈°╪#$^{n}$){ä□>÷□ú|∞æƒ╧□╞`9-
{ä·YùqX┴╟-aéÅ=┬J>÷□½·╪#L≥▒G~-□ÿéM|∞□µg?6├□>÷□≤│-
[a~÷c□∞αcÅ$^{n}$□)$^{n}$▄)|∞æƒw□D^┼░□

≡▒GÿƒüÜü·╣°·(¼□

├)>÷□°·╪#\─╟─ßb>÷□ù≡▒G°δ|∞□.πcÅ≡7°╪#\╬╟─ßo≥▒G╕éÅ=┬▀ΓcÅp%{ä·yô┐
W±▒G°▀≡▒G╕ÜÅ=┬ ûÅ=≥s╣≡s║≡▒G~^□╕□µτf■wp──
{D»°╪#·3>÷ê^≤▒G⌠ƒ·╪#z├╟─╤[>÷ê■éÅ=b□{─D|∞□□≤▒G∞=>÷êe≡▒G∞}>÷(□□

□□□âéA┴á`P0(□□

□□□âéA┴á`P0(□□

□□□âéA┴á`P0(□□

□□□âéA┴á`P0(□□

□□□âéA┴á`P0(□□

□□□âéA┴á`P0(□□

[illegible]âéA┴á`P0([illegible]

□□□âéA┴á`P0(□□

□□□âé┴■□âB^□!»üÉ╫@╚k Σ5□≥□□y
ä╝□B^□!»üÉ╫@╚k Σ5□≥□□y
ä╝□B^□!»üÉ╫@╚k Σ5□≥□□y
ä╝□ :≤□
bHîêæ≈8╛çdp╠@▐τ°>Æ╔1□╔Γÿà|╚±C$¢c6≥5Ä C>Γ°□Æ╦1□╔πÿç|┬±□$ƒc>"ß(Aεq╝ç□p,@PÄ(
éq─□)G)"π(CΣ□σêéú□!8□êèú□╤s╘#□Ä□─╚┬ê╨□i──┬äX8Z□+G+bπhC□□□êôú□±p⌠
^Ä^─┴┬Å□8□É□╟□□µ□FXÄ,□π□C□□□HÆc□·□Ä□Ç□r,Dè8□!┼□ïæ?Σ°çH ╟□Σδ□┐Äör,E■êπ-
!e□╦Éopⁿ□R╬▒□·&╟o"□□+Éoqⁿ□R╔▒□·6╟oc▄▒┼2▒□8~ÇqG□╦┬╕#î}êqG□╦╞r8µ`▄□╞>┬╕#îσb
sⁿ□πÄ3ûçq╟□√□â9┬□w┤▒|┴á`P0(□□

□□□âéA┴á`P0(□□

□□□âéA┴á`P0(□□

□□□âéA┴á`P0(□□

□□□âéA┴á`P0(□□

□□□âéA┴á`P0(□□

□□□âéA┴á`P0(□□

□□□âéA┴á`P0(□□

□□□âéA┴á`P0(□□

■~□ä–□ }îⁿ□ÖD■□Ö·U9â╠"s╚<ƒìÇ╧J└╒□D■□2ä

#ú╚□╚□2Äⁿ%≥W╚_#□â,  □YΓjdBRÄ"Σ└kε□≈Y≈æƒ jΘ=Σ□·□╩Öä((□²□²□═C?Aa4-
╜ç~è";□òí□Të¬P̅□╒p⌡≤É□@■□·'Σ " □·□≥╧╚╧æ[Täè╤≈╤□╨,4□σ╛I□□}êµó□┤□EQ

òú8Jájn█□æ3Σ□╣ß┌â╨≈╨

4□²É[;□□ë`□"┬Eñ╚ ▓è▄"F─è╥ó□Q╡¿JT#¬□5êÜD¡ó

Q╖¿W╘'□□ìê╞ESóY╤éhY┤*Z□mèvD{ó□╤æΦDt&‖□▌ê┼Γ,q«X"╞─äÿ□╙b╖╪+

ëπΓBq⌐╕B\-
«□╫ï□┼═Γ6q°╕GⁿD▄/-□Åè'─╙Γ9±ó°╣°à°Ñxï√K≥ßL8□éα□°cΦ}É≤πCÉφπk≡°0□}□╦a%⌠
╙▌Q□S0□!░ ╢A(∞Ç]□

rr(A6

5╚├í□□8┤ ≈å-d▌ 0┬ep-dB»╨□(ä ▐b∩C1># ≥S># `□αçÇ┘Ç9Ç□µ□~

◦

(□╝□°) □ê□bÇr@□P□H□*□╒Ç$
□h□4□Z□¡Ç6@□á□╨□Φ□⌠□□□□└ ╧▐╜┐┤╓α√□_[ôÿ╟±8N╞q»¼¼▄Vε≈{▓r[╢g¼Z╟:Äπ8Äπ▒Γ□□□+"
b¡╡╓c¡╡VV¼╡-│j¡êêêêX□□□□□□□□┘êXæìêêl─π□O═╖Ñ?
²
≥╥√ □ƒg├■╛v|ûƒBA□╨_C
í┼╨□h)⌠7╨rΦoí□╨▀A+í┐çVA □¡å■□Z□²□┤□·gh□⌠»í⌡⌐≥_í□□}â■□⌠□·□²╟T1□·□Ü□═ä▓á∞╧ª
M %╙4ÿª┴4
ªi0Mâi□L╙`Ü□╙4ÿª┴4
ªi0Mâi□L╙`Ü□╙4ÿª┴4
ªi0Mâi□L╙`Ü□╙4ÿª┴4
ªi0Mâi□L╙`Ü□╙4ÿª┴4
ªi0Mâi□L╙`Ü□╙4ÿª┴4
ªi0Mâi□L╙`Ü□╙4ÿª┴4
ªi0Mâi□L╙`Ü□╙4ÿª┴4
ªi0Mâi□L╙`Ü□╙4ÿª┴4
ªi0Mâi□L╙`Ü□╙4ÿª┴4¥6²□M'└t□L'└t□L'└t□L'└t□L'└t□L'└t□L'└t□L'└t□L'└t□L'└t□L
'└t□L'└t□L'└t□L'└t□L'└t□L'└t□L'└t□L'└t□L'└t□L'└t□L'└t□L'└t□L'└t□L'└t□L'└t
□L'└t□L'└t□L'└t□L'└t□L'└t□L'└t□L'└t□L'└t□L'└t□L'└t□L'└t□L'└t□L'╥ª óΘ$ÿNéΘ
$ÿNéΘ$ÿNéΘ$ÿNéΘ$ÿNéΘ$ÿNéΘ$ÿNéΘ$ÿNéΘ$ÿNéΘ$ÿNéΘ$ÿNéΘ$ÿNéΘ$ÿNéΘ$ÿNéΘ$ÿNéΘ$ÿN
éΘ$ÿNéΘ$ÿNéΘ$ÿNéΘ$ÿNéΘ$ÿNéΘ$ÿNéΘ$ÿNéΘ$ÿNéΘ$ÿNéΘ$ÿNéΘ$ÿNéΘ$ÿNéΘ$ÿNéΘ$ÿNéΘ$
ÿNéΘ$ÿNéΘ$ÿNéΘ$ÿNéΘ$ÿNéΘ$ÿNéΘ$ÿNéΘd┌⌠_4═Çi□L3`Ü□╙

ÿf└4□ª□0═Çi□L3`Ü□╙

ÿf└4□ª□0═Çi□L3`Ü□╙

ÿf└4□ª□0═Çi□L3`Ü□╙

ÿf└4□ª□0═Çi□L3`Ü□╙

ÿf└4□ª□0═Çi□L3`Ü□╙

ÿf└4□ª□0═Çi□L3`Ü□╙

ÿf└4□ª□0═Çi□L3`Ü□╙

ÿf└4□ª□0═Çi□L3`Ü□╙

ÿf└4□ª□0═Çi□L3ƒª+□5╥ê||Ö╚0f∞g╓f╛░F┘J÷6°Æ≤ÿ5╚□q╫□*²Θ.╗≈g□?[╬ëτ\²Ugnnεⁿ╧⌐ƒƒ
τ╡■é≤ï□₧ôwⁿ╤╞_~ΣOⁿ╨°½²é┌éù»úHΩg$⌐╧÷Cα┃²öH±╫¥»√_Å╛₧~╜°z⌡⌡÷δ²╫°»/_▀╤╘╧Jr╤|□
C%¿□╒úVöD)4è□íeh%Zâ╓úMh+┌üvú}Φ
:éÄ□>Å╧ásΦΓτô·:║àεó□Φ1zå^ó╫Φ□ ·Ç>ú»ƒ╧╟¼╧gσ╝╧'dⁿ≤╓Z═7≥φ|□?╚Å≤K°σⁿ*~▬
┐ü▀╟oπw≥{Éb⌠■≤╣·□} |╨µ┬s5÷·<¡Σδ·V>╓°°Q~□┐î_╓»ß╫≤¢°¡ⁿ

~7┐Å?╚ßÅ≤'·3$^{n}$9■"□à┐╬▀Γ∩≥□°╟$^{n}$3■% Ü□╟□α?□■=·└XX6ûç□`8F`j╟ê┘1□□─ΓX
VÄUa╡X□╓î╡a¥X□╓Å
aú□ìMa

6Å□c·□åI0%ª╟¼□ëQX□+┬╧░J¼□½╟Ü░V¼□δ╞·░Al□□╟&▒□l

[−V░ul
█┼

$C^\infty$

╗─«▒;∞□{╞^▒□□KÉ-╚□□□p□!P
î□╗└#□□Γé□A╢áJP+h□4
┌□¥é-A┐`H0*á□S□F0/X□¼□6□█é=┴íα□)□Pé¿áHP&¿□╘□Ω□MéVAçá[╨'□□î□╞□ôé□┴£`Q░"X□l
v□□éc┴ÖαRp-
╕□<□₧□»é□£àgπyx□Äπ□«╞ì╕□≈αA<Äùασx□^ï7α═x□▐ë≈α²°□>è╙°□╬α≤°□╛èoα█°-~êƒατ°7ⁿ
□ Ä?Γ?≡7ñ□o┬[±

╝□∩├□±□|□ƒ─g≡9|□_┴╫±-|□?└Å±3ⁿ□┐╞∩≡□ⁿ□□┼?ä,a╢0OX ─àäP-4□φBÅ0(î
Käσ┬*a¡░A╪,l□v□{ä²┬!ß¿É□N    □ß╝pI╕*▄□n
≈äç┬□ß╣≡¢≡F°]°(ⁿ!|□!"Ä(G⌠·V%□ëΣH▒p\8)£□╬      □à+┬ußûpWx <□₧
/à╫┬;ßâ≡Y°*ⁿ□▒D┘ó<Qü□□□"╡╙(▓ï<óá(.*□òï¬D╡ó□Q│¿M╘)Ω□⌡ïåDú"Z4%bD≤ó%╤¬
hC┤-┌□□èNDτóoó□╤w╤úΦçΦMîê9Γ□±τ█ÑX$ûï╡b│╪)÷ë├ΓBq)R,┌□φè

D╟ó3╤ÑΦZt'z□=ï^Ebû8[£'.□πbB¼□□┼v▒G□□╟┼%Γrqò╕V▄
n□╖ë;┼=Γ~±ÉxTLï°─îx^╝$^□oê╖┼{ΓC±ë°\ⁿM|#■.~□ □┐I□
GÆ#·|╤ûê$rëVbû8%>IXR()òTH¬%uÆFIïñ]╥à□ï∩─□Γg±½°C┬ÆdK≥$□□\BH╘□ú─.±HéÆ
╕ñDR.⌐Æ╘J□$═Æ6I°ñG╥/□ÆîJh╔öäæ╞Kû$½Æ
╔║dOr(9æ£K╛In$▀%ÅÆÆ7)"σHsñƒ▀6ñ"⌐\¬òÜÑN⌐O□û□JKÑ□╥ji¥┤Q┌"mùvI{Ñ□╥aΘÿtB:-
¥EèÑy╥□).%ñj⌐QjùzñAi\Z"-ùVIkÑ
╥fi¢┤S┌#φù

IGÑ┤tJ╩HτÑK╥UΘåt[║'=ö₧H╧Ñ▀ñ7╥∩╥GΘ□Θ□ü□□"çα□(!"Σäû0□N┬GäëBóö¿
¬ë:óæh!┌ë.óù□ åë1béÿ&fë□bÖX#6ë□bƒ8Bè □□$ΓD QNT□╡D□╤L┤□¥D□╤O

□ú□ML□

1O,□½─□▒M∞□ç─      qN|#nê∩─#±âxô!2Ä,G÷·MT&Æ╔eZÖYµö·daYí¼TV!½û╒╔□e-▓vYù¼W6
□ûì╔&d╚▓Y┘élY╢&█öφ╚÷eG▓S┘à∞Jv+╗ù=╔^ÉbYâ¼Y╔&δö⌡╚·eC▓Q□-¢Æl▓y┘ÆlU╢!█ûφ╔

e'▓s┘7┘ì∞╖∞Q÷C÷&GΣ□yÄⁿ≤□╣\$ù╦┤r|▄)≈╔├≥By⌐╝B^-»ô7╨[Σφ≥.y»|@>,-
ôO╚°σ|≥□·▓|M╛)▀æ∩╤ÅΣ°≥
·òⁿV~/□Æ┐╙▀□□□«"Wæ»└□□ñX>*°σSrF>/_Æ»╨7Σ█≥=·íⁿD~. &┐æ□ù?╩□╚▀□êéú╚Q≡□¿BñÉ+┤
□|┬⌐≡)┬èBE⌐óBQ¡¿S4*Z□φè.E»b@1¼□SL(ª□|è□┼▓bM▒⌐╪Q∞+Ä□°è
┼òΓVq»xR╝(▐ò□J«2WÖ»─ö□ÑR⌐WZòñÆRFòEH▒bC▒¡╪S□*N□τèoè□┼w┼úΓçΓMë(9╩£╧ ]Cò"Ñ\⌐
UÜòNÑO□V□*Kò□╩je¥▓Q┘ólWv){ò□╩aσÿrB9¡£U.(ùòk╩MσÄr_yñ<U^(»ö╖╩{σô≥E·«╩PqU╣¬|
□ªÆ¿ö*╝╩¬"Uö*¬*Rò⌐*U5¬zUô¬U╒ü□+oö▀òÅ╩-
╩7□óΓ¿rT<□¬□⌐Σ*¡╩¼r¬|¬░¬PU¬¬PU½ΩTì¬□U╖¬K╒½□P
½╞T□¬i╒¼jA╡¼ZSm¬vT√¬#╒⌐ΩBuÑ║U▌½₧T/¬wuåÜ½╪Uτ½1╡D¡Tδ╒V5⌐ª╘Quæ║L]⌐«Q╫½¢╘¡Ω

u╖║O=¿-QÅ½'╒3H▒:G═SújæZ«╓¬═j°┌°

½
╒ÑΩ□u╡║N█ ¿nQ╖½╗ ╘╝Ω□⌡░zL=í₧V╧¬□ ╘╤Ω5⌡ªzG╜»>Rƒ¬/╘WΩ[⌡╜ ·I²ó~╫dh╕Ü\M╛□╙H4Jì^c╒
É□J□ ╒□i╩4òÜ□M╝ªI╙¬Θ╨tk·4âÜ□╕ fR3úÖ╙,jV4δÜ-═«µ□) ╓85>MXS¿) ╒Th¬5uÜFMïª]╙ÑΘ╒

hå5cÜ
═╡fV│áY╓¼i65;Ü}═æµTsí╣╥▄jε5OÜ□═╗6C╦╒µj≤╡ÿVóUj⌡Z½û╘R┌¿╢H[ª¡└╓hδ╡M┌Vm
ç╢[█°□╘Äh╟╡ô┌□φ£vQ╗ó]╪niw╡□┌cφÖ÷R{¡╜╙>hƒæbm¥╢Q█óm╪vi{╡□┌aφÿvB;¡¥╒.hù╡k┌Mφ
Äv_{ñ=╒^h»┤╖┌{φô÷E√«╤╨qu╣║|□ªôΦö:╜╬¬#uö.¬+╥òΘ*u5║z]ô«U╫íδ╓⌡Θ□u#║q▌ñnF7°[╘
¡Φ╓u[║]▌üεXwª╖╘]δεt□║g▌½εC╧╥gδ≤⌠□z□)╓
δ╞t□║i▌¼nA╖¼[╙mΩvt√║#▌⌐εBwÑ╗╒▌δ₧t/║w}å₧½╧╒τδ1╜D»╘δ⌡V=⌐°⌠Q}æ╛L_⌐»╤╫δ¢⌠¡·

}╥╛O?¿╤Åδ'⌡3·9²ó~E┐«▀╥∩Ω□⌠╟·3²Ñ■Z□°□╨?δ_⌡□û!█Ég(0α□┬á6□
vâ╟□4━æb²Ü~S┐ú▀╫ΘO⌡□·+²¡■^ ñ□╤┐□2

\C«!▀Ç□$□ÑAo░□H□eê□è

eåJCìí ▌╨dh5t□║
}åA├êa▄0iÿ1╠□□
+åu├ûa╫p`86£□.
╫å;├âß┘≡j°0▓î┘╞<cü□7□F╡╤h┤□=╞á1n,1û□½î╡╞□c│▒
)6\□n
≈å'├ïß▌ÿaΣ□sì·F╠(1*ìzú╒H□)c╘Xd,3V□kî⌡╞&c½▒├╪m∞3

□Gîπ╞Iπîq╬╤ h\1«□╖ î╖ ╞□π▒±╟xi╝ 6 ▌□-
î╧╞Wπçëe╩6σÖ□L┐ ë0⌐MFô▌ Σ1□MqSë⌐▄Te¬55ÿÜMmªNSÅ⌐▀4d□5╤ª)ñ╪─5σÜ≥MÿIbRÜ⌠&½ë4Qª
¿⌐╙Tf¬4 ╒ÿΩMMªVSç⌐█╘g□4ìÿ╞Môª□╙£i╤┤bZ7mÖvM□ªc╙ÖΘ╥tm║3=ÿ₧M»ª□3╦£m╬3□ÿq3aV¢ì
f╖┘c

Üπµ□s╣╣╧\kn07¢█╟¥µ-s┐y╚<jª=Sf╞<o^2»Ü7╟█µ=ñ╪l5ôf╨□5□Ö╤╟òµ□s╝╣╔▄jε0w¢√╟âµ□≤
╕y╥<c₧3/ÜW╟δµ-≤«·└|l>3_Ü»=wµ□≤│·╒ⁿaaY▓-yû□
n!,jï╤b╖x,AK▄Rb)╖TYj-
ûfK¢Ñ╚╥cΘ╖

YF─┤e╨┬Xµ-KûU╦åe█▓g9┤£X╬-▀,7û∩ûGñ╪Rc⌐╖4YZ-□ûnKƒe╜2b□╖LZf,sûE╦èe▌▓e┘╛□XÄ-
gûK╦┤σ╬≥`y╢╜Z>¼,k║5╧Z`┼¡äUm5ZφVÅ5hì[K¼σ╓*k¡╡┴┌lm│vZ{¼²╓!δ¿ò╢NY□δ╝u╓║j▌░n[
≈¼ç╓□δ╣│⌡¢⌡╞·▌ ·h²a}│!6Ä-
╟÷·Xî□[□¡#╓qδñu╞:g]┤«X╫¡[╓]δü⌡╪zf╜┤^[∩¼□╓gδ½⌡├├▓e█≥l□6▄F╪╘6ú═n≤╪é╢╕¡─Vn½▓
╒┌□l═╢6[°¡╟╓o□▓ì┌h█öì▒═█ûl½║
█╢m╧vh;▒¥█╛┘nl▀mÅ╢-
╢7;bτ╪s∞ƒÅµvæ]n╫┌═v°▌g□#┼║□█║m╦║k;░□█╬lù╢k█¥φ┴÷l{╡}╪Y÷l{₧╜└Ä█
╖┌n┤█φ-{╨-╖ù╪╤φU÷Z{â╜┘▐f∩┤≈╪√φC÷Q;mƒ▓3÷y√Æ}╒┘a▀║∩┘□φ'÷s√7√ì²╗²╤■├■µ
@□□GÄâτ@□"ç▄íuÿ□Nç╧□v□:J□□ÄjG¥ú╤╤é□█/φ╫÷;√â²┘■j p░□┘Ä<Gü□w□

╡├Φ░;<Äá#ε(qö;¬□╡Ä□G│ú═╤ΘΦq⌠;å□ú

┌1σ`□≤Ä%╟¬c├▒φ╪s□:N□τÄoÄ□╟w╟úπçπ═ë89╬□'╧ë:EN╣Sδ4;¥Nƒ3∞,tû:+£╔╬:gú│┼┘ε∞r÷:
□£├╬1τ□R∞d9│¥y╬□'ε$£j°╤iwz£Ag▄YΓ,wV9k¥
╬fg¢│╙┘π∞w

9G¥┤s╨╔8τ¥K╬Uτås█╣τ<t₧8╧¥▀£7╬∩╬Gτ□τ¢
qq\9.₧
uë\rù╔ev9]>W╪UΦ*uU╕¬]u«FWï½▌╒σΩu
╕å]c«
╫┤k╔╡αZv¡╢6];H▒╦Φ▓╖<«á+ε*qò╗¬\╡«□W│½═╒ΘΩq⌡╗å\ú.┌5σb\≤«%╫¬k├╡φ┌s□║N\
τ«o«□╫w╫úδçδìDH

ÖC≥Hö□ærRKÜI'Θ#┣d!YJVÉ┏d□┛H┫║Éφd□┛K

É┣Σ□9ANô┃Σ□╣L«æ¢Σ

╡║O-æ°Σ□yE▐Æ≈H1YE╓Æ
d3┘FvÆ=d?9DÄÆ49E2Σ<╣D«Æ□Σ6╣G-Æ'Σ9·ì╝!┐ôÅΣ□≥═ì╕9ε□7╧ì║En╣[δ6╖¥nf;∞.tù║+▄╒ε
:wú╖┼▌εεr≈║□▄├ε1≈ä{┌=δ^p/╖╫▄¢ε□≈╛√╚}Ω╛p_╢o▌≈ε'≈ï√▌ôßßzr=·H▒╖▀=Σ-u╙ε)7π₧w/
╡║W▌□εm≈₧√╨}Γ>w□s▀┐┐╖▌?▄o-─├±Σxx-╘#≥╚=ZÅ┘π⌠°<aOí°╘Sß⌐÷╘y□=-
₧vOù°╫3α□÷îy&<╙₧Y═ég┘│µ┘⌠∞x÷=G₧S╧àτ╨sδ╣≈<y^<∩▐

/╫¢δ═≈b^ëWΘ╒{¡^╥K!┼₧%╧¬g├│φ┘≤□zN<τ₧o₧□╧w╧úτçτ═ïx9▌□/╧ïzE^╣Wδ5{¥^ƒ7∞-
⌠ûz+╝╒▐:oú╖┼█εφ≥÷z□╝├▐1∩äw┌;δ]≡.{╫╜¢▐□∩╛≈╚{Ω╜≡^yo╜≈▐'∩ï≈▌ùßπ·r}·>╠'±)}zf╒
G·(_╘WΣ+≤U·j|⌡╛&ñ╪{ε²µ╜±~≈>z□x▀|êÅπ╦±±|¿OΣô√┤>│╧Θ≤·┬╛B_⌐»┬Wφ½≤5·Z|φ╛._»o╚
7∞□≤M°ª}│╛□▀▓o═╖Θ█±φ√Ä|°╛
▀ò∩╓w∩{≥╜°▐²□~«?╫ƒ∩╟ⁿ□┐╥»≈[²ñƒ≥G²E■2□Ñ┐╞_∩o≥╖·;ⁿ▌■> á□─?Ä□√□?╟ƒπτ·Q┐╚/≈k²
f┐╙∩≤ç²à■R□à┐┌_τo⌠╖°█²]■^ Ç□╪?µƒ≡O√g²
■e Ü□╚┐π▀≈·O²□■+ ¡ ▌ Σ□±┐S□□ù╩Ñ≥)îÆPJJOY)Æó¿(UDòQòT
UO5Q¡T□╒M⌡Qâ╘□5NMR3╘□╡H¡Pδ╘□RLi)3σñ|Tÿ*ñJ⌐□¬Ü¬ú□⌐□¬¥Ωóz⌐□jÿ□ú&¿ijûZáû⌐5jô
┌í÷⌐#Ωö║á«¿[Ω₧zó^¿≈@FÇ□╚
Σ□░Ç$á

Φ□╓□□á□╤@Qá,P□¿ ╘□Ü□¡üÄ@wá/0□□ î□&□3ü╣╙b`%░-╪□∞□

□╟ü│└eα:pç□□*□╒ü║@cá%╨-Φ□⌠□□□├ü▒└D`:0□X□,□╓□¢ü¥└~α(p□╕□\□n□≈ü°└Kα=ÿ□Σ□sâ·
A,(   *â·á5H□⌐`4X□,
V□ké⌡┴ª`k░#╪□∞

□Géπ┴╓αLp.╕□\ «□╖é╖┴âαq≡,x□╝

▌□é╧┴╫αGê□╨

σ!┼┴▐α@p88□£□N□gâ
┴σαZp3╕□▄□-□Oâ□┴½αm≡>°□|     ╛ç2B▄Pn(?äà$!eH▓å╙□□èåèBeí╨PM¿>╘□j
uä║C}í┴╨Hh<4□Ü    ═à□C+í⌡╨Vh7t□:

¥à.C╫í╗╨CΦ9⌠□·□│┬┘ß╝pA□□□au╪□╢ç=Hqh!┤□Z
måvB√íú╨iΦ"t□║
▌ç₧B/í≈pFÿ□╬
τç▒░$¼

δ├╓0□ª┬╤pQ╕,\□«   ╫ç¢┬¡ßÄpw╕/<□-    Åç'├3ß╣≡bx%╝-▐□∩å□┬╟ß│≡e°:|□~□?ç_├-
□V$;Æ□)êα□"óÄ□#÷ê'□î─#%æ≥HUñ6╥Ç□çO├□ß½≡m°>ⁿ□~ ┐G2"▄Hn$?éE$□eD-
▒F╚□□ëFè"eæ╩HMñ>╥□iìtD║#}æ┴╚Hd<2□Öë╞E□#+æ⌡╚Vd7r□9Ä£E.#╫æ╗╚CΣ9≥□·ê▓ó┘╤╝hA□
Å□Qu╘□╡G=╤`4--ëûG½ó╡╤åhs┤-┌□φë÷GçóúHqΣ=Ü□σFsú·Q,*ë*ú·¿5JF⌐h4Z□-
ïVFkó⌡╤ªhk┤#┌□φï

FGóπ╤╔ΦLt.║□]ë«G╖ó╗╤âΦq⌠,z□╝Ä ▌E-
ó╧╤╫ΦGî□╦Äσ┼□bxîê⌐c├ÿ=µë□c±XI¼<V□½ì5─Ücm▒╬XO¼?6□□ì╤▒⌐□□¢Å-
┼Vc□HqL□╚╟¼l2F┼ó▒óXY¼2V□½Å5┼Zc□▒εX_l06□□ÅM╞fbs▒┼╪Jl=╢□█ì□─Äcg▒╦╪u∞.÷□{Ä╝╞
>Γ¼xv</^□╟πD\□7╞φqO<□Å╟KΓσ±¬xm╝! ▐□oïw╞{Γ²±í°h£ÄO┼Ö°||)╛□▀êo╟≈Γç±ô°yⁿ[ⁿå╟I
-=╝¼╘╥├πªû- ▐O⌐Ñçù¥Zzx9⌐Ñç≈W⌐Ñçù¢Zzx?O-
=╝_ñû--/╡⌠≡≥SK□∩W⌐Ñç≈5╡⌠≡╨╘╥├π°û--ûZzx┬╘╥├□Ñû-₧8╡⌠≡$⌐Ñç'M-
=<YjΘß╔SK□OÖZzxå╘╥├3ªû-₧)╡⌠≡╟⌐ÑçgI-
=<[jΘß╛SK□╧æZzx«╘╥├#SK□╧¢Zzx╛╘╥├úRK□/ÉZzx┐N-=╝┬╘╥├+N-=╝Æ╘╥├+M-
=╝▀ñû-^yjΘß²6╡⌠≡*RK□∩w⌐ÑçWÖZzx┐O-=╝¬╘╥├√CjΘßU°û-▐-
SK□»&╡⌠≡■öZzx╡⌐Ñç≈τ╘╥├½K-=╝┐N-=╝·╘╥├□M-=ⁿ┐O-=ⁿ╖╘╥├ ç╘╥├□O-
=ⁿÅ╘╥├ ╟╘╥â!⌐Ñ□√ÆZz░î╘╥âeªû-îòZz0vjΘI_0}┴⌠□╙□L_0}┴⌠□╙□L_0}┴⌠□╙□L_0}┴⌠□╙□L
_0}┴⌠□╙□L_0}┴⌠□╙□L_0}┴⌠□╙□L_0}┴⌠□╙□L_0}┴⌠□╙□L_0}┴⌠□╙□L_0}┴⌠□╙□L_0}┴⌠□╙□L_
0}┴⌠□╙□L_0}┴⌠□╙□L_0}┴⌠□╙□L_0}┴⌠□╙□L_0}┴⌠□╙□ⁿ?□D╛ E□üT ╒H□╥ê┤ φH□╥ï

├╚□2üL#┃╚□▓î¼!¢╚

▓Å□!°╚□rà▄"≈╚□≥é╝□╓°┬²Æ√%  ╤τÑÉ/ⁿ-▄▀!ƒ┐δτ?ôƒ²ù|µ│╜ⁿ┘╧■+h⌠_├»≈≤ █g
┐□v□^ [x=□²[Φ□|²┐â╫├≡5 -^Å└δ □□à■GΦ□⌠?A╟SE □╜ªß┐ôÇ□w□·ƒ·3ƒ¥ä╫ □:□²»≡δ╚≡⌡
□≈┘/□₧╧?╜ ╧τ=d£e\f\g▄e<d<g╜f|d▓2│3≤2
2±L"S¥i╠╡gz2âÖ±╟Æ╟≥╟¬╟┌╟å╟µ╟╡╟╬╟₧╟■╟í╟╤L:s*ô╓£╧\╨\═▄╚▄╬▄╥<╟<╓<╧ⁿûyô·=≤1≤G
µ□
aqX9,-
eëXrûûef9Y>VÿU╚*eU░¬Yu¼FV
½¥╒┼Ωe
░åYc¼
╓4kû╡└Zf¡▒6Y;¼}╓□δöu┴‖b▌▓εYO¼□╓;;â═eτ▓≤┘□[┬V▓⌡l+¢dS∞(╗ê]╞«d╫░δ┘M∞Vv
□╗¢▌╟-dÅ░╟┘ô∞□÷□{æ╜┬^go▒w┘□∞c÷□√Æ}═╛c?░ƒ┘»∞□

ïô═╔п□pp

┴Qsî□;╟├
rΓ£□N9°èS╥iα4s┌8¥£-N?gê3╩í9S□å3╧YΓ¼r68█£═╬!τäs╬·╞╣ß|τ<r~p█▓É,NVN□/
=□e╔│┤Yµ,gû/+£UÿUÜUæU¥Uù╒ÿ╒Æ╒₧╒ò╒¢5É5£5û5æ5¥5¢╡É╡£╡û╡Ö╡ô╡ƒuöuÜuæuòu¢uƒ⌡ö⌡
Æ⌡╫═αr╣╣▄|.╞òpò\=╫╩%╣□7╩-
Γûq+╣5▄zn□╥ò█┴φµ÷q□╣#▄qε$wå;╟]Σ«p╫╣[▄]ε□≈ÿ{╞╝Σ^s∩╕□▄gε+≈π'╓O┘?σ²T≡·≈ßg ∩s
@ö□√≤□)êH>▀C□□; ·□σ σ(□%É*Tâ·É□üRΦæ┐E □²ƒ╚ ÇOjxσ┐b_æ╖╘√kJ$/
╩à■□═åµ@ □Ü
²94□·
(□·Kh>⌠W╩□ΦW(□σC1¿□èCàP□T

ò@ÑP□*â╩í□¿□jÇ□í&¿□jüZí6¿□ΩÇ:í.(   uC=P/╘□⌡C)h□□ä■□Z□²'╨"h1┤□·OíÑ╨▀@╦á

Z

² ─┤□·;h%⌠≈╨*Φá╒╨?Bká□é╓B

¡â■5┤ − ·╧í
≡╛3â~I}ªiΩ5Ü eA┘PÉÇé□□

á`□²□□$á □□ (H@A□□□PÉÇé□□$á □□ (H@A□□□PÉÇé□□$á □□ (H@A□□□PÉÇjáZ¿

¬çé□□$á □□ (H@A□□□PÉÇé□□$á □□ (H@A□□□PÉÇé□□

á`□
A├╨□4□ìAπPpéé□□£áα□□'(8A┴ □NPpéé□□£áα□□'(8A┴
□NPpéé□□£áα□²π ╜°■@ 7{w□c┘y□x°VUù¥x9ε⌠b■«Éα8╬Jbî│□□╚-3□□

╥$3$╪ÄΘ}▀╖▌ √╛∩√r╫{÷╙φn≈▐M4îB─D□!□í□!ä□1häf╨□a2

□Γy∩S%╞Üß├¦□ì?┤¼·⌡┐Ω▐║Uu▐s▀z«σ÷ÖÑ│uÄ╬╒y:_□ΦB]ñïuë.╒e║\WΦJ]Ñ½uì«╒□u¥«╫
║Q7Θf▌ó[u¢n╫□║SwΘn▌ú{ufε╫□zP□Θa=óG⌡ÿ-╫□zROΘi=úg⌡£Ä╧T3
ÜkíÑVZkú¡vzA/ΩKzI/δ╥zE»Ω5╝«7⌠ª▐╥█zG□u╨□∩╘wq▄yÄ;╧qτ9ε<╟
·Å·}}e╨▒₧

⌐]b╟.1fù□│Kî┘%╞ⁿ┘□│Wî┘+╞∞□c÷è1{┼ÿ╝b╟^1f»□│Wî┘+╞∞□cS╟╧╥▒i:]-
╓Gε┌Φ«ì▀┐█(eúöìR6J┘(eúöìR6J┘(eúöìR6J┘(eúöìR6J┘(eúöìR6J┘(eúöìR6J┘(eúöìR6J┘
(eúöìR6J┘(eúöìR6J┘(eúöìR6J┘(eúöìR6J┘(eúöìR6J┘(eúöìR6J┘(eúöìR6J┘(eúöìR6J┘(
eúöìR6J┘(eúöìR6J┘(eúöìR6J┘(eúöìR6J┘(eúöìR6J┘(eú┴₧₧N╪(eúöìR6J┘(eúöìR6J┘(eú
öìR6J┘(eúöìR6J┘(eúöìR6J┘(eúöìR6J┘(eúöìR6J┘(eúöìR6J┘(eúöìR6J┘(eúöìR6J┘(eúö
ìR6J┘(eúöìR6J┘(eúöìR6J┘(eúöìR6J┘(eúöìR6J┘(eúöìR6J┘(eúöìR6J┘(eúöìR6J┘(eúöì
R6J┘(eúöìR6J┘(eú±□ƒ⌐│t╣╬╤╢:Oτδ□]¿ït▒.╤Ñ║Lùδ□]⌐½t╡«╤╡ ·ó«╙⌡║A7Ω&▌¼[t½n╙φ║Cw
Ω.▌¡{t»ε╙²z@□Ω!=¼G⌠¿-╙πzBOΩ)=¡g⌠¼₧╙≤:~T3
ÜkíÑVZkú¡vzA/ΩKzI/δ╦zE»Ω5╝«7⌠ª▐╥█zG□6J┘(¥░Q╧F)□Ñlö▓Q╧F)□Ñlö▓Q╧F)□Ñlö▓Q╧F)
□Ñlö▓Q╧F)□Ñlö▓Q╧F)□Ñlö▓Q╧F)□ÑlΣx▓Q╧F)□Ñwmt╫F»□□elö▒Q╞F□□elö▒Q╞F□□elö▒Q╞F□
□elö▒Q╞F□□elö▒Q╞F□□elö▒Q╞F□□elö▒Q╞F□□elö▒Q╞F□□elö▒Q╞F□□elö▒Q╞F□□elö▒Q╞F□□
elö▒Q╞F□□elö▒Q╞F□□elö▒Q╞F□□elö▒Q╞F□□elö▒Q╞F□□elö▒Q╞F□□elö▒Q╞F□□elö▒Q╞F□□e
lö▒Q╞F□□el4╪═│
□elö▒Q╞F□□elö▒Q╞F□□elö▒Q╞F□□elö▒Q╞F□□elö▒Q╞F□□elö▒Q╞F□□elö▒Q╞F□□elö
▒Q╞F□□elö▒Q╞F□□elö▒Q╞F□□elö▒Q╞F□□elö▒Q╞F□□elö▒Q╞F□□elö▒Q╞F□□elö▒Q╞F□□elö▒
Q╞F□□elö▒Q╞F□□elö▒Q╞F□□elö▒Q╞F□□elö▒Q╞F□□el4┘Γ3uû╬╓9:WτΘ|]á
uæ.╓%║TùΘr]í+uò«╓5║V_╘u║^7ΦF▌ñ¢uïn╒m║]wΦN▌Ñ╗uÅε╒}║_□ΦA=ñç⌡ê-╒cz\OΦI=Ñ°⌡î₧
╒sz^S□?╢As-
┤└Jkm┤╒N/ΦE}I/Θe}Y»ΦU╝ª╫⌡å▐╘[z[∩Φ└F□□e□6╩╪(cúîì26╩╪(cúîì26╩╪(cúîì26╩╪(cúî
ì26╩╪(cúîì26╩╪(cúîì26╩╪(cúî□I6╩╪(cú∞«ìε┌Φ5`ú└Füì□□□6□l□╪(░Q`ú└Füì□□□6□l□
╪(░Q`ú└Füì□□□6□l□╪(░Q`ú└Füì□□□6□l□╪(░Q`ú└Füì□□□6□l□╪(░Q`ú└Füì□□□6□l□╪(░Q`
ú└Füì□□□6□l□╪(░Q`ú└Füì□□□6□l□╪(░Q`ú└Füì□□□6□l□╪(░Q`ú└Füì□□□6□l□╪(░Q`ú└Fâ}
<L╪(░Q`ú└Füì□□□6□l□╪(░Q`ú└Füì□□□6□l□╪(░Q`ú└Füì□□□6□l□╪(░Q`ú└Füì□□□6□l□╪(░
Q`ú└Füì□□□6□l□╪(░Q`ú└Füì□□□6□l□╪(░Q`ú└Füì□□□6□l□╪(░Q`ú└Füì□□□6□l□╪(░Q`ú└F
üì□□□6□l□╪(░Q`ú└Füì□□□6□l□╪(░╤°è╧╘Y:[τΦ\¥°≤uü.╘E║XùΦR]ª╤uà«╘U║Z╫ΦZ}Q╫Θz▌á
□uôn╓-
║U╗Θv▌í;uùε╓=║W≈Θ~=á□⌡É-╓#zTÅΘq=í'⌡ö₧╓3zV╧ΘyM5╙±#£kíÑVZkú¡vzA/ΩKzI/δ╦zE»Ω
5╝«7⌠ª▐╥█zG□6□l□&l□╪(░Q`ú└Füì□□□6□l□╪(░Q`ú└Füì□□□6□l□╪(░Q`ú└Füì□□□6□l□╪(░
Q`#╟Éì□□□6□wmt╫F»□□σlö│Q╬F9□σlö│Q╬F9□σlö│Q╬F9□σlö│Q╬F9□σlö│Q╬F9□σlö│Q╬F9□
σlö│Q╬F9□σlö│Q╬F9□σlö│Q╬F9□σlö│Q╬F9□σlö│Q╬F9□σlö│Q╬F9□σlö│Q╬F9□σlö│Q╬F9□σ
lö│Q╬F9□σlö│Q╬F9□σlö│Q╬F9□σlö│Q╬F9□σlö│Q╬F9□σlö│Q╬F9□σl4╪┴≤
□σlö│Q╬F9□σlö│Q╬F9□σlö│Q╬F9□σlö│Q╬F9□σlö│Q╬F9□σlö│Q╬F9□σlö│Q╬F9□σlö
│Q╬F9□σlö│Q╬F9□σlö│Q╬F9□σlö│Q╬F9□σlö│Q╬F9□σlö│Q╬F9□σlö│Q╬F9□σlö│Q╬F9□σlö│
Q╬F9□σlö│Q╬F9□σlö│Q╬F9□σlö│Q╬F9□σlö│Q╬F9□σl4┘Γ3uû╬╓9:WτΘ|]á
uæ.╓%║TùΘr]í+uò«╓5║V_╘u║^7ΦF▌ñ¢uïn╒m║]wΦN▌Ñ╗uÅε╒}║_□ΦA=ñç⌡ê-╒cz\OΦI=Ñ°⌡î₧
╒sz^S=4Φ°q.┤└Jkm┤╒N/ΦE}I/Θe}Y»ΦU╝ª╫⌡å▐╘[z[∩Φ└F9□σ□6╩┘(gú£ìr6╩┘(gú£ìr6╩┘(g
ú£ìr6╩┘(gú£ìr6╩┘(gú£ìr6╩┘(gú£ìr6╩┘(gú£ì□=6╩┘(gúⁿ«ìε┌Φ5`úéì□6*╪¿`úéì□6*╪¿`
úéì□6*╪¿`úéì□6*╪¿`úéì□6*╪¿`úéì□6*╪¿`úéì□6*╪¿`úéì□6*╪¿`úéì□6*╪¿`úéì□6*╪¿`ú
éì□6*╪¿`úéì□6*╪¿`úéì□6*╪¿`úéì□6*╪¿`úéì□6*╪¿`úéì□6*╪¿`úéì□6*╪¿`úéì□6*╪¿`úé
ì□6*╪¿`úéì□6*╪¿`úéì□{w1aúéì□6*╪¿`úéì□6*╪¿`úéì□6*╪¿`úéì□6*╪¿`úéì□6*╪¿`úéì□
6*╪¿`úéì□6*╪¿`úéì□6*╪¿`úéì□6*╪¿`úéì□6*╪¿`úéì□6*╪¿`úéì□6*╪¿`úéì□6*╪¿`úéì□6
*╪¿`úéì□6*╪¿`úéì□6*╪¿`úéì□6*╪¿`úéì□6*╪¿`úéì□6*╪¿`úéì□6*╪¿`úéì╞W|ª╬╥┘:GτΩ<
¥»
tí.╥┼║DùΩ2]«+tÑ«╥╒║F╫Ωï║N╫δ□▌¿¢t│n╤¡║M╗δ

▌⌐╗t╗ε╤╝║O≈δ□=¿ç⌠░─╤úzLÅδ
=⌐°⌠┤₧╤│zN╧k¬Ö□═uⁿhùZi¡ì╢┌Θ□╜¿/Θ%╜¼/δ□╜¬╫⌠║▐╨¢zKoδ□□╪¿`úb┬F□□□lT░Q┴
F□□□lT░Q┴F□□□lT░Q┴F□□□lT░Q┴F□□□lT░Q┴F□□□lT░Q┴F□□□lT░æπ╞F□□□lT

l⌠ Φ∩±
Vg▌î┴Ω¼ƒ1Xù
║Q7·°µ□âU█2c░^[═█║╠█uçεt ]µ▌ε│╟╜╫╜O≈δ□=¿ç⌠≡á² h>ΩqÄ·▄πzb╞α,:i>Ñº⌡îÅƒu s▒wφ
x╫Ä ┐φX▓c╔Ä%;û∞X▓c╔Ä%;û∞X▓c╔Ä%;û∞X▓c╔Ä%;û∞X▓c╔Ä%;û∞X▓c╔Ä%;û∞X▓c╔Ä%;û∞X▓c╔
Ä%;û∞X▓c╔Ä%;û∞X▓c╔Ä%;û∞X▓c╔Ä%;û∞X▓c╔Ä%;û∞X▓c╔Ä%;û∞X▓c╔Ä%;û∞X▓c╔Ä%;û∞X▓c╔Ä
%;û∞X▓c╔Ä%;û∞X▓c╔Ä%;û∞X▓c╔Ä%;û∞X▓c╔Ä%;û∞X▓π`▀)'∞X▓c╔Ä%;û∞X▓c╔Ä%;û∞X▓c╔Ä%;
û∞X▓c╔Ä%;û∞X▓c╔Ä%;û∞X▓c╔Ä%;û∞X▓c╔Ä%;û∞X▓c╔Ä%;û∞X▓c╔Ä%;û∞X▓c╔Ä%;û∞X▓c╔Ä%;û
∞X▓c╔Ä%;û∞X▓c╔Ä%;û∞X▓c╔Ä%;û∞X▓c╔Ä%;û∞X▓c╔Ä%;û∞X▓c╔Ä%;û∞X▓c╔Ä%;û∞X▓c╔Ä%;û∞
X▓c╔Ä%;û∞X▓π°è╧╘Y:[τΦ\¥°≤uü.╘E║XùΦR]ª╤uà«╘U║Z╫ΦZ}Q╫Θz▌á□uôn╓─
║U╖Θv▌í;uùε╓=║W≈Θ~=á□⌡É─╓#zTÅΘq=í'⌡ö₧╓3zV╧ΘyM5╙á╣║□:~╟+¡╡╤V;╜á□⌡%╜ñù⌡e╜óW⌡
Ü^╫□zSoΘm╜ú□;û∞XN╪q≡²ⁿ¥■╝■â~O Q┐»»

□φX▓c╓Ä%;û∞X▓c╓Ä%;û∞X▓c╓Ä%;û∞X▓c╓Ä%;û∞X▓c╓Ä%;:b∞X▓c╓Äσ▌□»v╫F»□□UlT▒Q┼F□□U
lT▒Q┼F□□UlT▒Q┼F□□UlT▒Q┼F□□UlT▒Q┼F□□UlT▒Q┼F□□UlT▒Q┼F□□UlT▒Q┼F□□UlT▒Q┼F□□Ul
T▒Q┼F□□UlT▒Q┼F□□UlT▒Q┼F□□UlT▒Q┼F□□UlT▒Q┼F□□UlT▒Q┼F□□UlT▒Q┼F□□UlT▒Q┼F□□UlT
▒Q┼F□□UlT▒Q┼F□□UlT▒Q┼F□□Ul4╪»½
□UlT▒Q┼F□□UlT▒Q┼F□□UlT▒Q┼F□□UlT▒Q┼F□□UlT▒Q┼F□□UlT▒Q┼F□□UlT▒Q┼F□□UlT
▒Q┼F□□UlT▒Q┼F□□UlT▒Q┼F□□UlT▒Q┼F□□UlT▒Q┼F□□UlT▒Q┼F□□UlT▒Q┼F□□UlT▒Q┼F□□UlT▒
Q┼F□□UlT▒Q┼F□□UlT▒Q┼F□□UlT▒Q┼F□□UlT▒Q┼F□□Ul4╛Γ3uû╬╓9:WτΘ|]á
uæ.╓%║TùΘr]í+uò«╓5║V_╘u║^7ΦF▌ñ¢uïn╒m║]wΦN▌Ñ╗uÅε╒}║_□ΦA=ñç⌡ê-╒cz\OΦI=Ñ°⌡î₧
╒sz^S=4h«àû:~Σkm┤╒N/ΦE}I/Θe}Y»ΦU╝ª╫⌡å▐╘[z[∩Φ└F□□U□6¬╪¿búèì*6¬╪¿búèì*6¬╪¿b
úèì*6¬╪¿búèì*6¬╪¿búèì*6¬╪¿búèì□+6¬╪¿búΩ«ìε┌Φ5`úÜìj6¬┘¿fúÜìj6¬┘¿f
úÜìj6¬┘¿fúÜìj6¬┘¿fúÜìj6¬┘¿fúÜìj6¬┘¿fúÜìj6¬┘¿fúÜìj6¬┘¿fúÜìj6¬┘¿fúÜìj6¬┘¿fú
Üìj6¬┘¿fúÜìj6¬┘¿fúÜìj6¬┘¿fúÜìj6¬┘¿fúÜìj6¬┘¿fúÜìj6¬┘¿fúÜìj6¬┘¿fúÜìj6¬┘¿fúÜ
ìj6¬┘¿fúÜìj6¬┘¿fúÜì□;u=aúÜìj6¬┘¿fúÜìj6¬┘¿fúÜìj6¬┘¿fúÜìj6¬┘¿fúÜìj6¬┘¿fúÜìj
6¬┘¿fúÜìj6¬┘¿fúÜìj6¬┘¿fúÜìj6¬┘¿fúÜìj6¬┘¿fúÜìj6¬┘¿fúÜìj6¬┘¿fúÜìj6¬┘¿fúÜìj6
¬┘¿fúÜìj6¬┘¿fúÜìj6¬┘¿fúÜìj6¬┘¿fúÜìj6¬┘¿fúÜìj6¬┘¿fúÜìj6¬┘¿fúÜì╞W|ª╬╥┘:GτΩ<
¥»
tí.╥┼║DùΩ2]«+tÑ«╥╒║F╫Ωï║N╫δ□▌¿¢t|n╤¡║M╖δ

▌⌐╗t╖ε╤╜║O≈δ□=¿ç⌠░-╤úzLÅδ
=⌐°⌠┤₧╤│zN╧k¬Ö□═╡╨R+□?■ì╢┌Θ□╜¿/Θ%╜¼/δ□╜¬╫⌠║▐╨¢zKoδ□□╪¿fúz┬F5□╒lT│Q=
F5□╒lT│Q=F5□╒lT│Q=F5□╒lT│Q=F5□╒lT│Q=F5□╒lT│Q=F5□╒lT│æú─F5□╒lT▀╡╤]□╜□l╘░Q├
F
□51╘░Q├F
□51╘░Q├F
□51╘░Q├F
□51╘░Q├F
□51╘░Q├F
□51╘░Q├F
□51╘░Q├F
□51╘░Q├F
□51╘░Q├F
□51╘░Q├F
□51╘░Q├F
□51╘░Q├F
□51╘░Q├F
□51╘░Q├F
□51╘░Q├F
□51╘░Q├F
□51╘░Q├F
□51╘░Q├F
□51╘░Q├F
□51╘╤`Ån&l╘░Q├F
□51╘░Q├F
□51╘░Q├F
□51╘░Q├F
□51╘░Q├F
□51╘░Q├F
□51╘░Q├F
□51╘░Q├F
□51╘░Q├F
□51╘░Q├F
□51╘░Q├F
□51╘░Q├F
□51╘░Q├F
□51╘░Q├F
□51╘░Q├F
□51╘░Q├F
□51╘░Q├F
□51╘░Q├F
□51╘░Q├F
□51╘░Q├F
□51╘╤°è╧╘Y:[τΦ\¥°≤uü.╘E║XùΦR]ª╦uà«╘U║Z╫ΦZ}Q╫Θz▌á□uôn╓-
║U╖Θv▌í;uùε╓=║W≈Θ~=á□⌡É-╓#zTÅΘq=í'⌡ö₧╓3zV╧ΘyM5╙á╢□ZjÑ╡Ä»B½¥^╨ï·Æ^╥╥·▓^╤½z
M»δ
╜⌐╖⌠╢▐╤üì□6j&l╘░Q├F
□51╘░Q├F
□51╘░Q├F
□51╘░Q├F
□51╘░Q├F
□51╘░Q├F
□51╘░Q├F

□9>l╘░Q├F═]□▌╡╤k└F-□╡l╘▓Q╤F-□╡l╘▓Q╤F-□╡l╘▓Q╤F-□╡l╘▓Q╤F-□╡l╘▓Q╤F-□╡l╘▓Q╤F-
□╡l╘▓Q╤F-□╡l╘▓Q╤F-□╡l╘▓Q╤F-□╡l╘▓Q╤F-□╡l╘▓Q╤F-□╡l╘▓Q╤F-□╡l╘▓Q╤F-□╡l╘▓Q╤F-
□╡l╘▓Q╤F-□╡l╘▓Q╤F-□╡l╘▓Q╤F-□╡l╘▓Q╤F-□╡l╘▓Q╤F-□╡l╘▓Q╤F-□
vτv┬F-□╡l╘▓Q╤F-□╡l╘▓Q╤F-□╡l╘▓Q╤F-□╡l╘▓Q╤F-□╡l╘▓Q╤F-□╡l╘▓Q╤F-□╡l╘▓Q╤F-
□╡l╘▓Q╤F-□╡l╘▓Q╤F-□╡l╘▓Q╤F-□╡l╘▓Q╤F-□╡l╘▓Q╤F-□╡l╘▓Q╤F-□╡l╘▓Q╤F-□╡l╘▓Q╤F-
□╡l╘▓Q╤F-□╡l╘▓Q╤F-□╡l╘▓Q╤F-□╡l╘▓Q╤F-□╡l╘▓Q╤F-
□ì»°L¥Ñ│uÄ╬╒y:_□ΦB]ñïuë.╒e║\WΦJ]Ñ½uì«╒□u¥«╫
║Q7Θf▌ó[u¢n╫□║SwΘn▌ú{ufε╫□zP□Θa=óG⌡ÿ-╫□zROΘi=úg⌡£₧╫T3
ÜkíÑVZkúπk╤Θ□╝¿/Θ%╝¼/δ□╜¬╫⌠║▐╨¢zKoδ□□╪¿eúv┬F-□╡l╘▓Q╤F-□╡l╘▓Q╤F-□╡l╘▓Q╤F-
□╡l╘▓Q╤F-□╡l╘▓Q╤F-□╡l╘▓Q╤F-□╡l╘▓æ#├F-
□╡l╘▐╡╤]□╝□l╘░Q╟F□□ul╘░Q╟F□□ul╘░Q╟F□□ul╘░Q╟F□□ul╘░Q╟F□□ul╘░Q╟F□□ul╘░Q╟F□□
ul╘░Q╟F□□ul╘░Q╟F□□ul╘░Q╟F□□ul╘░Q╟F□□ul╘░Q╟F□□ul╘░Q╟F□□ul╘░Q╟F□□ul╘░Q╟F□□u
l╘░Q╟F□□ul╘░Q╟F□□ul╘░Q╟F□□ul╘░Q╟F□□ul╘░Q╟F□□ul╘░╤`_ε&l╘░Q╟F□□ul╘░Q╟F□□ul╘
░Q╟F□□ul╘░Q╟F□□ul╘░Q╟F□□ul╘░Q╟F□□ul╘░Q╟F□□ul╘░Q╟F□□ul╘░Q╟F□□ul╘░Q╟F□□ul╘░
Q╟F□□ul╘░Q╟F□□ul╘░Q╟F□□ul╘░Q╟F□□ul╘░Q╟F□□ul╘░Q╟F□□ul╘░Q╟F□□ul╘░Q╟F□□ul╘░Q
╟F□□ul╘░Q╟F□□ul╘░╤°è╧╘Y:[τΦ\¥°≤uü.╘E║XùΦR]ª╤uà«╘U║Z╫ΦZ}Q╫Θz▌á□uôn╓-
║U╖Θv▌í;uùε╓=║W≈Θ~=á□⌡É-╓#zTÅΘq=í'⌡ö₧╓3zV╧ΘyM5╙á╣□ZjÑ╡6┌Ω°è\╨ï·Æ^╤╤·▓^╤½z
M»δ
╝┌╖⌠╢▐╤_□öì║
□ul╘░QτΩ±▌□▀□;╓Ä╕≥C╝╫┼╖d≡æ⌐∩ï¥4²┘°÷_ª,Ö÷o□≈□□-▄2σàW▀o┌Z≈√ro╤ö∩L█0⌡±
W▀6σÅ▌÷╤╝{°-
ΩìL·πΘ_£÷▄┤█»╛╟┌┐□□╟▐╙{b·s╝⌐SτO█╤□Ö■Ñx╧Y╙₧¢·⌐)□2m├┤ⁿ□╝≈≡{°m·┐║▀□±÷W $╖|ì╟
°□Ñù⌠ tⁿ3°^ƒ6;>▐φ▐╘┤oN}n┌∞°╚ √¡O╝·╓□µ±₧Φ□╡7⌡ƒ{╝)/°) Θs Å√=±╧▀∩╒_#■£≈L□n·≤
╚┐:²àΘ┐2π□±+▐╫□₧╛x·≥┴ZM[Θ▐≈▄38ûCo¿^5╫╜^ⁿ│╫{á╫ïΓì╧Φ▐¼°üΘ╝ííççf⌠åç╞å▐□τw
²L£??t,╬╟çΓ│sΦΣ╨╣8ƒèτ■╨═í┐î≤_
²uodΦ;C -╬▀□·∩q▄█í┐ï≤▀□²C£┐7⌠²8┐2<╘□□--₧□ì=:|O£∩□╛/╬≈□?□τ□çè≤Σß7━y╧≡┤8O-
~$╬±ùc£╟å▀□τ□~K£□~k£~G£▀9ⁿ«8┐{°▌q■íßè≤{å▀□τ≈

┐ 7╬O

ƒÄ≤Öß3q>;|6╬τå╧┼·ⁿ╚gzC#ƒ□y║72≥SúS{C╤─±τì.■T£?=·Ö▐╚ΦgGƒì≤sú│π<gtq£ùî«î≤¬╤¡
q▐6║─╬█G┐-τ_-
²⌠8□πu±êFa╛¡7ⁿ║╖┐~noΦ⌡≤^?»7≥··□v╝í□/<xí7≥α┼□□=╬▀x≡?─·¢╓¢zC╓¢ôx¼Æ┐M^Θ
=╘{(«╓C├ô▀┌□Üⁿ°Σ/⌠F& ▄Σƒï≤╧O■·8 ┬Σ_ê≤/N■╫q■ΓΣ/╞·Kô□)╬_₧ⁿσ8□e≥WΓⁿ╦ô≈─y∩Σ╝q
▐7e4>°εë·□Ö≥┬⌠ƒì╦·L|▐
O J|å
M□6₧3├╙ƒ□ΣzCÅ|∩æWΓ3║╫ÅK¥·π╜æ■G√ì≤@¿#²□█ ò8□£:╥ ┌î╖╟¥α□±

□b╓æ□∩₧▒vΓoΣ

«□□□╥□□f\ì┐6#₧a3ε╟□∞□_¢±╖q-Φvd¼?╓∩
ì═□□ï;═□▀°«┴3~Γ□□ε²j<ï■╨·≤]g╬▀□╬ÖíW£-ú╬ô√¥!ô¥□╙¥□c╬ç╟£
±\□y:«Θ`╒₧ï²ΩΦWc□e4~ ±u═,+╕$v┘Φ▓╪□ú+&╓Γu╓ú╓úq--KΓπ$Å'qWJ▐₧╝=÷¥I|╟Σ▌
I<╗Æ≈$±╜J₧H₧ê}2y2÷}I▄⌡Æ□$ê²P≥í╪Å$ë²▒Σ╟b?Ü─cÿⁿD≥□▒-
K>□√ëΣ□▒ƒIΓ╣ùⁿR≥K▒╧'╧╟╛É─=2ÖÖ╟î¥ù╟ï]É,ê]ö,è]û─∩6Yæ─∩6Yò¼è]ƒ¼Å▌ÿlî▌Ü─│1┘æ∞
ê▌ò─=9┘¢∞ì▌ƒ∞Å=ö□è=Æ□ë=û─τor"9□{*9□{&9□{.ë╧σ$M╥╪Éä╪")b½$ε□Iô4▒]□W6╣ÿ\î╜ö\
è╜Æ\ë╜û\ï╜æ▄ê╜ò▄è╜ô▄ë²Z≥╡╪_K~-
÷δI|Ä$▀H╛□√□╓o─~3·f∞o&┐□√[╓o┼■v≥█▒┐ôⁿN∞╖Æo┼■n≥╗▒▀N╛□√{╓∩┼■~≥√▒□ÉⁿA∞-
%□□√□╓_─■e□≈ƒΣ»ô┐Ä²¢Σob┐¢|╪Ö?╕╬∩□«Ω;y╨Σ°┌lⁿ¬╛S°Lì≤°U}₧≥p£q%▀7N·ü8┐╓u{-
¢≥X£╟»█√╢)oï≤█]╜≈╜«╥√╘ö°ΓG▐τ·╝-
£≥┴8Å_ƒ≈#S~4╬π╫τ²ΦöÅ╞yⁿ·╜?□_□▐?q}▐°°ⁿT£╟»╧√╢)ƒï≤°⌡y??σgπⁿî½Φ>-
ƒ│≈╟gb▄°√±5@oR?╛

ε▌□_=▐τ□·□⌠εφ?╪OΓⁿP í8Oε┐!╬S·SΓ<╡ p£-
Θ≈π<ú ª8┐╢ µ8 ` ╤8┐Ñ û8?□_àNΩ┐╡ ╬°ÿ∩Ω┐7╬O⌠ƒê-
ß■Å─·┌■Sq~_ ²q■@ âq■P ├q■H #q■╤■Å┼·╟π.qo▄-
~"╬?┘ X£?▌ D£?┘ d£?╒ t£?╙ l£ƒε□1╬_è;╔╝q□Y□□ó⌡Q¥»∩oê·╗°□<*o┤□:·n┤?╕*▐h□≡ °
□φ□■k≡╤■└e#²ïQ[ú²□╤Yú²█q□□╡≤îFI}Åÿ■▒w î∩╗^≡=c≈⌡&ì▌?v□∩▐▒□╞ª╞yZ▄sεÖ╪_ε□zε▐
gz≈>╗Σ┘τz∩yv╔ⁿ□╝¥╧»^2»w|µ

ù⌠╛5s╔
s{□8δàτû⌠■δ╝gù-□zá≈□╝Iƒ■╪3Å÷▐≤╣□⌡┼G{■┬O□≥╤▌'□■Ö╪╧≈z»╝╥□ì┐üg⌠▐┘√@∩â▐-
Θ╝╛7╓{╫─|_∩ì╝wO╠≈╟╟·íë·ü▐¢z∩▒δ
▐Ä□<╪¢▄□■°╧|ß╤▐¢┐≡╠╟π╫s╦ñx█C╝∞=1±▐αw²¢{∩¥xo$~╬ú╝-
■°╟□Ω╝ßƒµI±ƒ)╝╗⌠₧î▀├╨╙┐□□éi□_kj∩▒ëiZ∩¡╝ç{#O□■≤ƒφ=■╟╧|ε╤▐ô?≈╠┐|┤≈/&-a┤7╝≈x
∩G&╛τç{oδ=5q╦=╝Gzo∩╝oΓ╝{π»ìw⌠▛{··EK□
▌╤□»┐í┐Ñ▀╥▀╙?╘?¥√┬Æ□C□>Φ≡□·╙║@≈δ∩Φ╗⌡□⌠O⌠?Θ_Φw⌠□Φ≈□□Öñ≈ΘΣ·╧.Ö;≥░~Z┐¬¢5╙»δ╗
⌡╧⌡{âN≥╣ô₧╤ú┌Ω┐╙o=ƒ;□εñ?╙∩Φ≈□□╝G'δÿ>ª∩╓°⌠G⌡π·S·╠╥ÑO~h⌠K▒-
-]░µ╔□╛□tk∞□Fw╟~p⌠`∞çFÅ╟~x⌠l∞GF≤╪Åì╢▒-
╝□√ë╤□▒ƒ□²Z∞°F┐-√Θ╤o«y≥COÄ■v∞Åî■n∞Sú┐√╛╤?è} Φƒ┼~`⌠? O╔=□«è¬δ √2gf╬a

"-.-É¢Dèªå Σ□M ╤

∩□èdjHªff╛ªFªhxëx╘╠╠╠╟╠∞F=djfVffff^æ

╨≥éÜß%S$3τ²═╟V9□²{₧ ≤╢║>▀Y|f_╬₧╜╫^kÅÜ╪□ ╔5□■┘□₧╫□▐æ□?H4²┐=Θ@:Æ$╥ët&#╚²$çî$
╣Σ□2è<ê3σ□2û<D╞æç╔x≥□ÖÇ^ⁿ}z▒╖·∩╦#|╨ ≤±Q≥¿yÑu«□k▌k╡«uk□╫`∞╪;▒½║Ædr□I!▌H*ΘN
zÉ╗IOÆFzæ▐ñ□ΘK·!ó

Θf,░j╫Wδ┐+⌡ □ö÷C╡è¿└═¥/cW½êe ▌│Hî╟□,□æ)□æ╟âê□î╪□èÿΘEl

G□î@¡(xT

óQ,bL□bI3D╨x#Z■⌠≡□²N

r□□Jε#├╙pX\X□wg|ì·²□Z%□$ô

"âI□□B▓╔╝xµ$□δyJß┘╞5╟╝Ä4»╣µ⌡□≤:╩╝>h^G¢╫1µu¼y}╚╝Ä3»□¢╫±µ⌡□≤Z{WL$Wh5ô±▌ò─╥X
6□╦≥X![╞è┘&╢ô□g□╣─═╝□╛░&Kπ□█□eÄ□r«<O╛¬─;°;τ;W8K£¢¥╕¥ç¥g]ü«□W□╫t╫□╫vWÖƒΣτ±
ï⌡K⌠K⌡╦≡╦⌡¢ΣWα╥╪∩óµ»Eh□┤₧Zû6Z[⌐¡╤╢h╟╡ïn╔φq╟║╗╕√║°╣╫╗╖·°°□┼··┤ ò□«□╝□╟o░ó┴
╞Çv□=□╞□li¿5£▄pN├òü┘üï□K□╧□^k□╙(íQJú⌠F9ì&6Ü╒hQú
ì╨□]≤─xzx╞zµ{╓z╨<Wé\A▐á°áñá┤á∞á▒AyAàA╦éèâ6□φ

*□:□t%╪□∞
Ä□N□N
╬

-□£□\□╝,╕8xS≡╬α≥α╙┴WB\!▐É°ÉñÉ┤É∞É▒!y!à!╥BèC6à∞

)□9□r%┖à╧íx£⌐╥pB□□Ü□Zê┃PqΦª╨¥íσí°C»4v5÷Γ
+⌐qZπ∞╞c□τ5.l╝

▀W¢□∩l\▐°tπ+^ù╫δì≈&y╙╝┘▐▒▐<oíwÖ╖╪╖╓╖╙[ε=φ╜□µ□≤å┼ç%àÑàeçì
╦
+

[□V□╢)lgXy╪Θ░+ß«pox|xRxZxv°╪≡╝≡┬≡eß┼ß¢┬wåùçƒ

┐╥─ ╒─█$╛ IRô┤ &┘M╞6╓3≈Ç⌡τ

êmaE░U≤Dπ┴^ñ╞Y&¼▄╥»╞YzππZmpåqª°┌Θ
╛÷└*_{pÖ»=eó»¥≈ô»=m□q¬╡∞·újò╦ä╛╛▀╫~╧╪INs°□Ü#ªδJ-╜⌐□₧╙¢=}╛╥╫▐╓Ñû
╛-g┌7g├x╢\Φ]û▐┼│√╪╒╞╡%D╢`D▓8½Σ¢□K∩÷□:EΦ⌐fφPd│.êα}▒≈çaGÅ'ô╔t2ç╟'K╙□≥:)!□╙f
▓¥∞&eΣ09A╬è╓yB┐)⌠vK∩aBG =TΦëBo╢⌠^MhQ╛w⌐╨eû▐τ□:CΦ┼Bï≥²üB≈□z╛╨╗-
}@╜φ□Q~á@ΦMµ[╟ [Ñ#╧σ VM$yd□)$ï╘2▓è□ô╡d□┘JvÆ²ñ£□%°╔□D'F]4Ézi

ì°    4ëª╨4Ü.z□3P┌├╥□┼

□▄bΘ21╥▓YB_┤⌠╖¡ä₧#⌠Oû>□#┤¿□Φ╕Ñ┐□δ·¥¿ ▌)Kù»╢tEÄÑ┐□φ┐_dΘ-
$í°□╜╫╥çπà□²□□²²╪[Φ╡û■Iî °IB▓⌠æ

B/□·Ü»7-╒|=·L¼ik ª g╢¬╟δ¼Zb4g«Z ·∞BKƒ□o□«ï╨b4τ ▌□z¢╨W,}>AΦ4í≤δε°≤óφ»mä-m╓1╛«b
q▌K─┘.□'Ü

Σ▌\Σ1½Ä≡ú_┼∩\XiΘ▀z□]eΘïb÷/%
╜┴╥╒bò.□□]T▀₧╜,z»□k÷╖x■{ÖY╖□ÖF□H□YLûô╒Σ□▓₧|L╢æ]ñö|OÄô*ræ\Ñ□╒¿çå╙X┌Æ
&╥╬4ò÷ª□t(═ÑπΦ$:ì□╨"‖ÿ.°½Θ;t=²ÿnú╖h)²₧-°U⌠"╜╨$ª1□
g▒¼%Kd¥Y*□~qE∞▄?─□\ì░⌠ƒ╔û╛&▐P╖VÆaGÖÜZ~╟ÿ╡▓Ljfi╟ K╤╓╞2e╜ÑUk╞Ö╙┌'╟%
]ni?╤?ÖVhiw«Ñ²S-▌└·█□□
m·7khEO□hy□k□.┤□9ÿ'▀╥A╓~f┴Ü╨Ñû

▒ó03■□╚╩ì[□}┴╥▐Mû

│÷/
╖µì5□┐╙─≥□□±▒Ñ#τ
}┌╥Qó}┤x»□+B▓ªb-Üèq╞Z╛┴ni'┤□WX£ÿ┐[┼√5□σ═«Y║╣□⌐Y╝╡│Xïlƒ}╦ZF°∞[╓fó═.░╛╦}r
k│╢V9∞█C}φä┤:;æ╡□>╨V°@[[m□╒môX"┤□-
┘□yf□□╬≈í8├7#m─≤òB[Öâ╡□δ┘.EΦ⌐⌡╖k╖Bh▒εφ]B'
=┘w-┌»≡□s√□⌡∞l╓lJhß=□┼[t¼±]àñ╬µI┬▓qéH□Q½▄░G╫*G√ñ▒╡l□√{_╗S╝»¥Æτk°n'Ng¡╖Θì≤
□ú¿q}N:αï⌡'╛+│D╢□WYƒ╤ûε+╝╕ƒò▀┘□▒□╙¡∞└□è]ù)·┤╓τñä'â┼■╓Zπ;▓!I÷Ü≈ZÖò
φα;□Cù█∞╝╛∩4┤Ü8σzV·>Å╨IB
/╝oì╨W╟ôΘì□
ï≡]½√G┘╝□╧-ΦmΘYQû^╢╣V□□a/E□°½V :_╦F·{U╛▐⌡╛ò'j═┬□1Ω
ç}G≤┴µ·ⁿεôΘB[yÆm□╤m≤èz╝-
┐ iîÑ╖è±lgû■▓╞╥{'°╛Oy╟┐æ▌┘□1úG¡S□;&bτq□s+E«8!b▐╓□íEL<%÷≥╧"û²Æ
⌠Yƒ┘┴ε½ZeΘ3=ê╙»╓<₧Öj┌□3╬3"╞₧□{²¼ê⌐τä□ƒ□╢δⁿRK *b╧»"& z┬╥□j,²[_í▀!NÑ╓h.□┌∞╓
µΦd╝à□g╘xÆ@äO^□┘≤ÆÿëΩ╫-
}YD±Üiû■=Γ»z°=GΦ²û■C╝δU1ù□Ä□║╘╫≤«Ñ╪=∩Ü╚╠╬µÿ1Γoµ≥Ü8□\█!┤╚├║┐╨bÄ⌡ëB╧□Z─_}½⌐
9z╡┤□Q8¥ε3NN▀⌐ô#8▌!┤⌡¢£⌐">□█╞lò□□¥*⌠,í╫
m¥╨9w ¥,⌠$Γö(Q╟▀╜yr┤╩□à╣╓ÅKDΦ6B[½┬□Γ-□══□║j¡^╩ìo□½╞□íτ
²▒╨τ--
G□▌[h1□yï╨╓9ü+±>;ÿ+âlδ╟¥ó□τx│$░╬xΩ√v▓ZΣ□╝HΦ═Bï1║╝B≈□zé╨╦à▐.┤¿∩□*tè╨πä^*⌠.
í¡²╞╡(í╙Ω═φ\□s⌐ë9╘6□m¥%┐;└lWw□q╖uÜΓ■b─
è,□`y;o□ tæ-"±åG-
▌┐ì╧9üç¡2wæÅ▀å[gD▐D¼eô□╡╬X░#ⁿmvK¢▌█fÅ│┘≤m÷□¢m²╣å╧ÿ"─ÿ"àwDv¿['2]Φ<íW╒SG°Bñ
□#yö□▌:Q±Bg□=╝₧:┬Wóä»D¥»['┌_Φ

Bτ╘S'_Φwä>TO□ßW1▒B≈»['FxDî□W╟ÄzΩê8╘╘#t▓╧╓é7═⌡╡πV√Σv>$╨╫-÷║»=ó▄╫╛ ó»=r½»¥█
╞╫~╨σkÅ□j│≈·┌π┬mví»²po_√æ<_√╤⌡╛÷ £⌡▌□ôsm÷q_{J«o√)╟}φ╝BƒS4□bu¡□!µwå╦▄{7φQ╛
÷¼Tƒ│(ƒ□πk╧
≈╡τi6√¬»²t¢Z┐Å±<=╟<æ■╡=╓f╫■µ0∞Y6╖╨f/▓┘╥j¥å`□zmvè═-m│τ█∞⌡6╖▄╫~å╪∞8¢▌█fO┤┘K
k¥Å

{ï=>σ[┐(└f╖│┘├lvü=.╢┘{}τ»¿┌╫■çT╦_

[╡┘«┐) Om·∩□Åⁿò-╒╡τ'·┌
╞·╢_╕╠f»▓┘┼6{╝=▐l₧în⌠┐p½»²lá=εk|□█∞π╛²/jf|█╪∞$¢¥j|√·₧ü□e┘∞═╛⌡ƒï╡┘-
mvó=εl|m┐ \φ°êx÷\å»╜4┬w}ùu⌐k╫·;ö║v_¢=╘fÅ|┘y6{₧=^j|ïm÷&¢╜╙fù█∞S6╗┌╫~I▓┘-¢□
k|□mv¬═╫░┘╣6{Æ=.░┘ïm÷j¢╜▐fo|┘√m÷O6√¼═╛Ωk/w┘∞P¢□g|m≤▒<┼f°█lπ\∞éτ7#²æ»ⁿ▒τf□
┬┌░■d□[═╛%+·?·?I⌐4Izà□t□ÉτQε□Φ║ƒ~ΦÜδGΘ

−@δ╬‖i≈j+╪πε\≈X÷ë{å╗êm≤g■*█∩□┘ 2√ÄP≈□π▀□╔e₧· k□ΘαI⌐%=à┤¬G╥=·7d

d)d□dç) ╒uñU□ □

╛(Σ┌M QM□V»î

)╣!°B.\ù╨

!y⌡H□dæ╖µªä1K╟□¢ä□ä┼▌ÉûßЈ
⌐ªΣ╘'íEß□┬τ49UK.□□□U»┤è╙║!□#ª▐ÉUB▐¼WJLY/┤»□□W╘ïî5%ß║X¡#;G÷ì£□╣5≥jölê╜≈(¡>
▒zÅ□ÄJ□Æ~Sî_ë╧2«□ç
Ü□7 zCè¢»╢!⌈à∞Ç□n╛#~!dEïó□½Ü∩╘╡¿e╘mq¡□MIo╡□▓╖u□ñ░Mr¢A
╣═╩█πn┐jH¢Σ□oBOHN┬╕äëmC!
ë╦!%ë□∩╕bI╗≡÷ç;□$⌡H:▀y□d·¥ô∩▄xgY╫□!▐«▒]□ÆτAû$OIΩûk╩╛╘(S▓S□ww
±╘╩åεkZ}╗ÅÇ╕║□÷¿║█{wQ»
╜6⌠ε▄g{╖│VmΦ╛V¡~╦ìz²è√τ≈ƒ▀□·□y@ü)%□vÿR6α°Ç+╕ûÑ7âtI?ö~x`τü╗!□36úVIf╦╠ûΘ═pM
2ε ╔ÖiÖYÖç
□$
è7Ñ≤á4╨yP╞á£A□(O□┤qp╥αΣ┴√!τ│ªú₧ä2│$δ╨áî!)Cz▀[r_∞░
#'î╠□Y≡p╬├□╟╖□ƒu]Å/□_°H└ñ±ô≥'ùM■iJ─öΣ)iS2ªΣLY8e╒ömSvO94σ─öïS]S=Scª╢Ö║{Ω·╟
σ╟√?>∞±    Å┐■°û<o^b▐ó╜-
yW₧X ─ëiß╙╥°σN+£╢·I╫ô⌐O«Ü₧5=o·ΩΘ¢°∩¥~eå6ú▌îⁿ□¢□□┌σgσÅ═ƒö┐$□e■╓Ö╥╠v3τ=\5≤π
Ö╒│╘YI│2f¡ÿuΦ)∩S╡O¡xjoü□AlAv┴╘éφ│Ñ┘i│ù╬.¢ú═)·ïXσ⌐□Å|ó═£╥¢b─æ9╪nè□A■b∩╜i▀q
╛√─≥⌠zú╬⌡╙SK|c╟▄╪¢bDç╣ 7┼è
F

Q#c¢\@□ε╨╜/▌─è┴åF╝
)A|¡

"┴□=¡n─L╘
=h╛╒î┐-O⌡═╪)f)▀h□▒jσ□æ□│ç°F,6δv0╩═τb□╤»□┐║□Q¢x:ÿ╜╡≥,┼/{î║f₧ΦYO~H7□∩f-┐æ
î~ΩF□□√Ufê?ZD√S╫π╜╤Å·╓½p_}=□b=dk╜î╪d┼-
+╛Yδh─DD@c╒╢^ÅÄ7V□1.Γ═╣╓Fï¢k▄|═▄Σ╡╓êqF¡töòD%7_S╫'□□□jE╘zΓlφ┐Z7ªè╚□kzô□Eï»
╟O#«π ~unZöî'k"J:/╩l□₧cσ1S#gEd!SσäO┐æ}DV═
╧╢Öü,»4r¢Y;╟¿ü╢Qß□î□≤
j□╧C≤<¡«{j─TöÑåO0┌¢≈µ╙¢y┤v&5╞bf═δy≤fµ£Ç╤┘≥ñOª1%≥πD37ÜúGyû°uⁿ■ÇÆ≡.□½
0-ƒ┘7f═ÿπ╡╔╡w∞⌡9╢vó1¢û°4▀□òÄ⌡└j□3□QΓ¡1╫[6╓ª╓«^□Ñß]Eå5W□≈s╙"

╧M│─°□C7 c¼èqgyÜíτª╡(è_hae╕°àfV¬%Få│▓¢Ö ?┼╟╒╡ñn
#╙╓□æqoH▐□fª²Å─╠┼ ╢\╧╪□%÷Ö2ΣF-
13√┐-µiπ▀□√∞ÿgöZRw■╠│K-1ⁿ▐ZΘ LΩ÷ⁿ≈ú√≈─Ügπ∞□D║σ÷╧O═÷t0N=åt;k<Θûk£tLδl |π

$╩ 8A-
1NM╓S33ì░─h╙kâq▓2╧P}╖≈5╧G╞□¬ojT╖│╞Θ□°Öδ°□C$£oÆ2□□'□├□'¥╬Γ^┬)(┘xb₧u╨.SêyΓ┘
o₧ìP╫,òîköå┌ÆqÜB┤(╚L2╧]eBJ╟'□╞┌╥┤J2ôî╕$╩ 8╣═░├8íÖφÄ¢g5êyN█m₧τP╫8¥▌<»
(Θ>┬£ælc.□∩╖fó[«·>□▒5╥⌠ff▀╞/□7·▓·╡φ┼:+Z█□Z%Z□a┤□╛┌n╞╫b□ΘJΓ╓]É6ñ□□Hn7 ╒p□□
BFæ╬d4)"id>)&ÅÉMd3¼-Ég╓NRF□æCÉù╓□ñÆ¼$'╤π[4îåæ²4é▐F

╨▐┤□9F√╤

RI┃ΘP≥
□Nçô┃⌠~ÜK╣╤qt<·ì>FùÆj║

ΓÑ╥!at□$£╛Eïi□║àεíæ°^Má¡Y"kO□X□KóφX□╓ò╢g▌X*φ╙z░─┤□δ╓z╤╬¼□δCô┘□6É▐┼□▒,Ü╧εe
≈╥╖┘p6£÷d╣∞□z□{É=H{▒1l<φ═&▓╓4¥MesΘ`÷4√□}É-`KΦ8╢ö╝H'ß
·=:Ö¡c_╨□÷%+ú/░C∞□}â²╟~íδ╪9v₧n`□╪e║æ²╬■á¢Ö╬
²î3╬Θτ\ßn·□o└□Θ.εß-║ù□s/▌╟úy

_
π▒ⁿ□zê▀╨¢╙r▐Æ▀F□α¡yk·#┐¥'╨ƒx"oGÅ≥$▐ëV≥;y□zÆ'≤d·3Oß)⌠4Oσ⌐⌠□▐ç≈úU<âg╤s<¢Åñ□
·8■0╝╞'≥)î≡i|□ô·t>¥)| □ü⌐╝äù0□_╧╫3?■□ ÇiⁿC■9s≤▌ⁿ[□╧Å≥_X

»µ:k)9$□╓N≥H═X▓t°t'╥ö&Is┘ ⌐Pzfìò6J¢┘□Θ□i□√°┤_¬d+ÑSÆ╬╓;\

□▌σ╨□□√╞□α□d╗□□□▀▒}Ä∩□?▒CÄcÄc∞□╟ ╟
v╪q╧±3√╤±ïπ<;Γ╕α╕└N:.9.│SÄ▀□┐│_□□8■`UÄ?e□;#+▓?½û□Σ□vM

öâÿ.ç╨□£╥╥r[εÆ∩É∩αMΣ÷≥▌<Bε'g≥╓≥}≥,▐N.Éτ≡í≥╙≥3|╕╝@^└sΣgσE|ñⁿ╝ⁿ<□@~A^╬G╔+σò
|£ⁿ¬ⁿ*□X~M~ìÅù▀û╫±GΣ
≥╟|¬ⁿ┌╜ò╧É╖╥_≥°Σ»Σâ|╢ⁿ¡|ê/Æ╥σr╛X>, ╙ƒùO╨°·
≥»≥U■ÆB□╞▀P□%è□+qJ"▀ªtTεΣ□öd%ÖR║)w≤∩ö4Ñ/?¼

P□≡cJåÆ┴Å+âöA╝R╔Vå≤□╨H%ùW)cö1ⁿ¼≥É2òƒS≥öΘⁿO%_Ö)1eÄ2WÆöBσ□IV□(K%UYª,ô□òσ╨r⌐
æ▓ByY≥(½ò╒R░≥╢≥æ□ó|«|%5S÷)eRkÑB╣
▌í\T«H}ö½è.e¿qj£öÑ6SπÑ!j+╡╡4TMT□ÑajG5I□«vV∩öεWô╒diñ┌SMôr╒▐joΘA╡»┌O□¡

T3Ñ▒Ω□uê⌠░:R}P□»>ó>*²ÅÜ°µIô╒'╒'Ñ)j╛:KÜ¬╬UƒûPtsPƒQïñΘΩ□uüö».R□I3╒%ΩK╥,⌡
⌡MiPts·╢·╢T¿û¿%╥3Ω□⌡7⌐H╜ñ^Æµ½5jì┤└I£TZΦö£Æ┤╚⌐8]╥sN═□"╜αl∞l,╜Ω

sFH½¥Q╬(ΘM╫@W╢│⌠ûkäkä⌠₧+╫ò+¡uìvìæ╓╣-r=$╜∩z╪5^┌αÜαÜ
mtMvMû>tσ╣≥ñM«i«□╥G«╣«wñO][\;ñJ╫A╫≈╥Y╫aWÑTφ·▌╧+]≤kΩ╖╨□σ╖╙∩□╟|┐
~¢□+ⁿ÷°]p╝í)Z¿πk¡à╓▌±â±□□8j┤ç┤     ▓S¢¿MÆ□hô╡⌐ráû°σ╔A┌4m╢□¼═╙µπh╕P[(▀¬-
╥□╥═┤%┌J╣à╢J[%╖╙Vk∩╙φ╡w╡⌡r▓÷ü÷æ▄C√D√DεÑ}¬}*≈╓>╙v╚}┤]┌~9S+╒JσíZÖvH╛O+╫~öGh
G┤≤≥(φ7¡F₧¼]╤«╧╚┤kn"╧p37ôg╣%╖,?σV▌nyÄ;└□,□╣C▌í≥sn»;\^∞Äp╟╨/╕π▄q≥□≈

≈

·e≈L≈lyÑ{₧√-
≥kεg▌╧╓o╗ƒw/æK▄/║_ö╫╕_r┐$┐τ■°√□y¡√U≈□≥□■╟▀_■╪?╨?D■╩?╠┐ë╝╟ ▓ □y?æΘ□╜£≈╤?µÖ
ñ□╧"□ƒ¿ ╨7ô÷╚;[⌠╜░*╤╗*₧⌐'□╫╥äß·□╧╥≈□?≥╢~òl╙»╥□ñ□╜ƒñ╙□╥ÿÄ$╤╙□
ΘCñ!j&ófW>N öP⌠sîH¿½ínC╘╒P╫e÷WëZgßH├H8╧¢ó<□σMP▐□}▌é╛ó╤·%îτ0±├▌Zî╖!ƒåq<⌐□Ç
±&±c·
ⁿ8i├+I□?IZ≡ƒ⌡}ⁿ┤±»╞╨√^⌠~öH╕c<δ┌□═b⌠⌠9ÖJ□
g□Ç$╥£t□╣·>≥□□□■G?I&Θ□╔c`2ÿ□ªé╟ëF≥⌠²Σ 0
< ªâ°╨╛□╞□s╘\0□<
□┴3á□|HRÉíSH
ε»□¥4°□P╨ƒtó□@:□□2└□╥Ån'æxπ1|□Θ╞∩%*

╞æy|□ëα3I□□èDH/δ√Ñòα□░ƒ4ù

ÇRp□öüo┴!≡□(□□α{≡□iε□╨≈9~╥≈;~!Üú□≈g└y}┐∞ irsΦ╢ñ╡■

z£╛O~□î□ÅÇ╟⌠ô≥dÇ╣æ172µF╬□ÿ□·]╥I^□>□ùI'%₧D*-
└p╥\□□r└ú`"x□ΣâÖ□sñ,□╧éù┴+$Ey□·

8
╬â_┴□p□`

╒æ □<□-#æNB:9=$╥⌠▌□≡kùy≈3V²2 é╫«â╫«â╖┼┴█εé╖═é╖
ä╖σ└█εü╖%ú÷j°K+>Hƒ╧□δy≡á;α7╧úç□|│■:?□?½$£ƒÇ□■Lε5²∞8j}O□ ▐╪□├HδZ²≈D ô╤□w⌠▀-
╡│╤≈b⌠²□Z╡E▀K╨≈KΦ∩c⌠7ê°úùsΦσ□z      @/╖óù±ΦÑ5ziì^Záù[1╩├Φ⌐□z╩E/
ΦßM≤M┐┬▌╗$□}|è>>E═Φp}□·iì~åúƒD⌠3□²tÑc⌠=Φ½5]¬oD╦Å╨ƒä■&cdú╨g#î∞)⌠÷

?¬_─Φv≥S╪¡?ô█°i▒c□ó╫x⌠:□╜┤║G»▌╤k,zlå▐

áσ□∞╝>x╦LΓ'"╠ƒê$Fdyæ<ÑWæ□0□╠□s┴<≡4(□╧Ç"░S»!_â]α□░□∞□{┴>░□□Ñα
8□~╨ur□ⁿ□~□G└QpL Ü□□òαé^A~├>┐□.üjp□╘ ║²Ä≥+α□p□ⁿ «a,║^E
áfT<╞│ßaC⌡s|□⌠□²£┤_»Æ

ÇRp□öüo┴!≡□(□□α{≡□8Ñ╫H?â╙α□P□╬Ç┃α□8□~□□└oα"└Xñk@╫┐v□Ω_+╔zì╥□ñü^á»~R╔Ç╬□┘(
┐□

□├⌡*e□╚□□í∞QΦë`□ε°Ç⌐αq╪╙á≤ígé9╕ƒ
░

╨□Φà╨┴éτp┐□<□ûÇ□╨ ╤x╛□≈½q 6ε▀┼²G□kñ`ì□¼æé5R*t]·-`ì□¼æé5R~B¢#α(└□)?δ□╧i≡
▐Ñ□£╤≈*g┴9ö¥G▀┐é
α"l¼¥R
}□6╓H□
r┴□X/å»"ÅÖ╣8Ö□▀═ä□□┘╤□δ_░╥`▌□/▀╞≈É□äΓi5IàgV└3+αÖ□≡╠□xf□<│□₧Y□╧¼ÇgV└
3+P√$<¡□₧V□O½ü°╒└╚jαi5≡ó*xL5<ª□-S
Å⌐╞∩m┴∩U≡√êâ▀□rαA#⌡c≡Ü□xM□╝ª□^S□»⌐Ç╫T└k*α5□≡Ü□xM□╝ª□^Süò¼╞JVc%½▒è□X┼□¼\5V
¡□½Vü╒¬╞JUcÑ*░*□Xì□╠z
f╝□│^âY»┴¼╫`V½0½Uÿ╤j╠h5f┤□│XüY¼╞,V`□+0ï□µÄ-
'□µ≥.∞d□╣≈□Σ▐≈·^Σ┌}╚B╚6µⁿ₧╞□ε├□-1τw□¼PXßÿ▀YΦß[Æà<□ì<□ì<□ì<□ì<□ì<□ì<□ì<□ì<
□ì<□ì_jç\□ï\□ï=[è=[è=[è={□{÷□÷∞%∞┘K╪│ù░g/!ƒ□b╧Vb╧Vb╧Vb╧Vb╧b╜I/Σ═D∞╙#╪°?bƒ
-┴>²æτÉ8>□î#□╙úæ╙úæ╙úa╙¥╤╙¥╤╙¥╤╙¥╤╙¥╤╙¥╤╙¥╤╙¥╤╙¥╤╙¥╤╙¥╤╙¥╤╪ïò╪ïò╪ïò╪ïÑ╪{ù
░τJ▒τJ▒τ*æπóæπóæ▀óæ▀óæ╫ó▒W*æ█óæ█b▒W*æ▀óß Ñ≡ R°□)ⁿ┐□■□□■□□■□ ■□
·/□·/□■
ƒ/à╧_é╧W"□F# E# E# E□■«_└_└·l╛>□+╨□±ⁿ□Γ·cXë₧Xë╫PZ□o∩╬≈π$U¬_π□IÄ╣z□
¿]ÄZçÉ1τδ╙aσáφ~┤=Ç°╔h;m┐D█4┤-E╖!D□√h0j-D═R╘L3╧Wå╩╛a÷⌠□╧╗ó|7╧╤P▐
==ì╥5Φ)□=φDOm╠·▀Öτ─├µ╡Ü╕h□□I│┴8≡0x□L□Åéë`□(DªoH╖□7~e□zfè~╛2╧F+I□ ê▄
┴?├·%Mæ┤□Γö□ê╠φ┼)▒)?à╚≡3Fp□╧~!w
ƒO╘?Cï`£)cî£Ä÷π╚═╚`┘≡·{╔=|ÿy·║ç°cda□Y□F□åæàada□Y□F□åæàada□Y□Zz╨r<Zz╨r╝┘╥ì
ûn┤túÑ□-▌hΘFK7Z║╤╥ìûn┤îC╥█╤2

-o7[jh⌐íÑåû□Zjh⌐íÑåû□Zjh⌐ëûëóe"▐Σ^□Å╗xsÄ╫Ögä╥ÿ¡□╠p□0□ñâü â╕pvsß∞µ┬┘═à│¢
τ)□Nε[H#┤Θ/N□█╠5:BJi3²(m

ΓA
╨□▄□Zü╓á
╕□$Ç╢ □▄□ ┌ü÷á□Φ□Æ@'╨□▄ ║Ç« □▄□R@7É□║â-αn╨□▄□╥@/╨□⌠□}A?░

╝□ûâ□αe░□╝□VüW┴j≡□x□╝□▐□oüb≡6x□ⁿ
öÇw┴□≡-X
╓ü⌡α}£╓╢@□ªù╙¡αs░
|□╢π·ù·A║□|□vé»┴.²□²□∞□{pé╚╞╫╨0}»⌠□N□█┴ù`□°□∞□_â]α□²á┤□∞╤□:□ΩG□-□□éA□□□ì⌡
ú≥□≡"└□╚+⌠□≥δ·9·
≡&x
□â⌡x■94N¢≥□╕▀½ö□á■!▄WδGò& □Dé(□¡ƒSb@S□
n□q·AσV╨L/WÜ□°é□_P░εJ□∞╢(δñƒP:C°δτTª-
U9ÉÇ□╚@□*p□□≡□□p□□╨□□□╝»□□□□╝╖è≈V±▌*▐[┼{½xo╒
┬@8└°Uî_┼°Uî_ì□1á)ê□╖Ç8î)A?í╢□□⌡âj□Φäg╓á□╕□

G╜□ΦQ({□⌡Fâ1`,x

eOéΘ`□╚□
≡ⁿU╘□□⌡▀╘╦╒╖`□â
xvI?Ωñ□∩ΩlñtΓ=£A·g□|Φ  è┘íÿ□è┘íÿ□è┘íÿ□è┘íhA1;□│C134@?I□é@╨□x@□□□!
□4□^£Y#@$ê□╤ □4□▒α□□□n□═≡ò▌□─â□á%╕
┤□¡A□p;H□mA"╕□┤□φA□╨□$üNá3╕□t□]A2╕
ñÇn □t□=└▌á'╕□ñü^á7Φ□·é~á┐~£

□Θ` ╚□Ö□≈ 0□dü!αI²

¥

fÇ|0□╠□Oü□0□╠□s┴<Ç∩
║P┐Lƒ□i└s`1x-,□/Çeêæ/üσ`□x□¼□»ÇUαU░□╝□^□╚Ç⌠M≡□(□oâw┕┐@
@¼Ñê╡⌠=░□¼□δ┴□─≥╧└V≡9╪□╛□_é□α+░□|
∞Q$S┐Q:
yá□"□gΣü□ê■¥□╡≈Iêx□"₧äê'!ΓIêx□"₧äê'!ΓIêx□"₧äê'!ΓI%°Fy□¼□∩ü╡`□X□▐□□⌡3╥ç`□°
□|

>□¢⊥∘ˋ
∘

l□ƒâmα□óI╗┴-ó9□□ù├Cⁿ□A □äÇP╨ÿ°╔E·□·-
zò╝□≈Kp┐T?)┐ê£ä50ú┘Jöß]Σ╫På1╦□│î1╦ê╥≥╗·qy
Xï▓u└êr□P □<√□σ¢└G░?□□°îqÜ╤∩K╪;Q÷5⌠.<√□∞□{└^ó╔□≡█°╢ô±m'ùß┘╖·e3Rûcl°₧ôOó-
╛YΣ*▄πt-πt-ƒ□°fæ±="π¢E■
\□ù@5▐φ▓~\±╫╧(
@□h□B⌡╦Jcα□a □4!.%□Dé(□G4σV╨

4□╖πY□t[Ç,½ ╖ZQùh*#~*□□p□□(@□Nα□~@□nα□□Ç□╨□□éF└C\j□□□!
□4□^□□┬□╞⌐b£*╞⌐b£j4ê□MA,╕□▄¬ƒQ[α□¡%╕
┤éìôéz;ε»GΓD▄╖□φA□╨□∩æ□zπ╛□└w«┌□φ·δ█╘□ □

╤/½├1╬Q¿gÅ╥° ▐U±╜½N□O■/o≈--
w]τ{ⁿ╫Ö6i3□ε□□□╣╙èèêÇεè7öeqY½ε«.┬¬┘#ê)□╣òBkz1□ïÇσN╢T@*□┤φ┌lQäå□E┌@J╥µ2MCô
6□ÆL'╙$═Lª)°=╧╔V

z╬y£≤╧9□/□Ö╔o~┐∩τ²■\╛┐1á5╠╟□,t■-
ε¡■'║÷}ÄK\≈□<êç≡K╫½├ƒ║°»╝╟├⌐yƒ▌□╞ªEaτ┤I÷JSCv□=°U8-∞²CúΣDg7íª□ß╜#q□⌠πi╟ö╛ù
,U·■}Uì□m¢╪ú╜°╬√Wx åë∩QJ√¡\4%v^°╫°ù├Kv°□Ñ∩╢ⁿn0·H∞c!□;□ƒ─τp^hë})╝□;-
_╢+ z╪fw╤ew╤UqAx╡ΓB▄□2□ Ä[≡□▄è█p;<╦U,╞□╕□wßn▄â{qε╟□<Ç□±□-╞Rⁿ

ÅαQ<åƒπq,
ÖΣçC&è[i!vügΓ½=Cƒe²yδ╧╟>□╥╓ƒÅ}╤±û░=÷□╧.□E°Φ_°8≤╓è□□Θè□╞7≡»°n╪^1□ùπ□\ëkqs╚
ï-/╢╝╪≥b╥ï-/╢╝╪≥b╥ï-/╢╝╪≥b╥ï-/╢╝╪≥b╥ï-/╢╝╪≥b╥ï-/╢╝╪≥b╥ï-/╢╝╪≥b╥ï-
ƒ°√░=q>■□_╞

|□ ┼╫┬v▒τy°╓╜┴í╫b□>å⌡□▀□-+÷:q╫┼.□+b□c□n ki░╢⌠ⁿ-
÷:▒╫ë╜N∞ub_+÷╡b_+÷╡b_+÷╡□╫ç□□7`-□ß╟aàu¡╡«╡╓╡╓║╓Z╫ZδZk]k¡kmt6□¬9Pmm╜□¿╢╛1□
4*âF¡│█JRVÆè□²Åú±
■ÿ7]*9s¬ΘR╓¥S≈?π»ô]ú▓k╘ΩRVù▓║ö╒Ñ¼.eu)╬TsªÜ3╒£⌐µL5g¬9S═Öj╬TsªÜ3╒£⌐µL5g¬9S═
Öj╬TsªÜ3╒£⌐µL5g¬9S═Öj╬TsªÜ3╒£⌐µL5g¬)Éó@è□)□ñ(Éó@è□)□ñ8S□}æ□UT¿Γ┼□*T±cC∞╝Φ
╜óƒ!·□√┐o╜u ≤⌠ç¿0¥□°Sa:□N▀ -
±7y╡üW□x╡üW□¿1â□3¿1â□3¿1â□3¿QEì*jTQúè□U╘¿óF□5¬¿QEì*jTQúè□U╘¿óF□5¬¿QEì*jTQ
úè□U╘¿óF□5¬¿QEì*jTQúè□U╘¿ó╞

j╟á╞

j╟á╞

j╠á╞

j╟áFUT.□FE£□±¥"╛N─çêp╛□τDG╤h□}╓╤ª¥6φt8ä□ç°φ▌Γ_'■uΓ_'■uΓo□□╖°█┼▀.■v±╖[G╗u┤
[G╗u┤[G╗u┤[G╗u┤½òΩ≡╦┐Φwú╤)▒»Θq□áZƒ¢⌐╟]å╦ß┌V▄≤N»½╤3□äW□≤B&±C╘`>□`!□ßG¿┼ì°1
né▐ÿ╨□□zcBoLΦì   ╜1í7&⌠╞ä▐ÿ╨□□·bB_LΦï
}1í/&⌠┼ä╛ÿ╨□□ÿå□$⌠╝Rg╧L¼=»╞╚j<¡╞╙t+=°ƒΣ╖¢╘nZφª╒nZφª╒n┌┌≤╓₧╖÷╝╡τ¡=oφ
yk╧[{▐┌≤╓₧╖÷╝╡τ¡=oφyk╧[{▐┌≤╓₧╖÷╝╡τ¡=oφyk╧[{▐┌≤╓₧╖÷╝╡τ¡=oφyk╧[{▐┌K=δé░à┌»Q
°àwzV)óεΦπ"¬≈√□~?╞ì╖╕±□7▐rn╖s°:7íR*D·QòR!┌Åε

Φ□□zïCoë▓^ö⌡ó¼□e╝(δEY/╨zQ╓ï▓^ö⌡ó¼□e╝(δEY/╨zQ╓ï▓^ö⌡ó¼□e╝(δEY/╨zQ╓ï▓^ö⌡ó¼□e
╝(δEY/╨zQ╓ï▓>:S$╡╝Y╧¢⌡▒ΩΦ□■¼□┴wU└^□P□╓ì"9b 73Gö╛Ö□╓²Ño│x╖₧wδy╖₧wδy╖^T╡ó¬□
U¡¿jEU+¬ZQ╒è¬VT╡ó¬□U¡¿jEU+¬ZQ╒è¬VT╡ó¬□U¡¿jEU+¬ZQ╒è¬VT╡ó¬□U¡¿jEU+¬ZQ╒è¬VT╡
Ω°éë:■kQ╜╛ □s:╫¬∩╢ΩUQB╜MΓm□kô╕

□╙ß~s»xÜ─╙$₧&±4ë°)*ï═µδuaolNx3vú╝╕=Σb≈û╛i≈εx∞╞Pê&·∩╜╤╔╬(─«ù□7α╞╨□╗)Ü□╗┘°○
²▒√ó╩╪Æ░/÷@╪ù░┐M╪▀&▐ï≈ßX╝╟ßx\∞£K≡=\è∩ú□3q□.╟,\üαJ\à½q
«┼l\ç9╕-7`n╪7□╧╕ò÷╞jBƒXv╞ε ╗c₧⌠ó
cW╦÷k0█╗╫ï≥□,□═▒àXäß╞Φ≡╪Mael▒≤ε□=▒;q□ε╞Æ≡î°₧I─┬kë8&c□╧PÄ⌐ÿå□$ÉD%

└ü8□□π□□è├p8ªπ□□ëú≡-□□r4╠╤0G├□
s4╠╤0G├\ΓSí9q□>ì╧α│°□>Å│±□|□ταoq.■

τßK╕X□ùα{╕□▀G5fΓ2\ÄY╕□?└ò╕□Wπ□\ï┘╕

sp=n└▄≡L4YµlúΓf*nÅ▌□åσ╥ìaD₧îE_σBæ
E

îsáöa█M£éëSpFü╨E*□Mÿé
S0a□&L┴ä)ÿ0□Ω□⌐_ñ~æ·EΩ□⌐_ñ~æ·EΩ□⌐_ñ~æ·EΩ□⌐_ñ~æ·EΩ□⌐_ñ~æ·EΩ□⌐_ñ~æ·EΩ
□⌐?N²qΩÅS□£·π╘−
°■8⌡╟M╣é)W0σ□ª\┴ö+ÿr□S«`╨□¿[ñnæ║EΩ□⌐[ñnæ║EΩ□⌐[ñnæ║EΩ□⌐[ñnæ║EΩ□⌐[ñnæ║EΩ□⌐[
ñnæ║E5w¥∞.╒b
Mτ╥ε□ú□¿▌Kφ□╘▐□]Iπ□□7╚⌠~g«°u/¡{cs╜« □>5"≤│2?+≤│2?╥ç╖·╨└ç□>

╟~□^Q□□*áC□t¿Ç

╡⌠Ü▐≡□-╡±¿ìG
<jαQ□Å□x╘└ú□-5≡¿üG
<jαQ□Å□x╘└ú□-5≡¿üG
<jαQ□Å□x╘└ú□-5≡¿üG
<jαQ□Å□x╘└ú□-5≡¿ùG╜<ΩσQ/Åzy╘╦ú^-⌡¬É¼□╔¬É¼□╔¬É¼□╔¬É¼□╔¬É¼□╔¬É¼□╔¬É¼□╔¬É¼□╔
≥╕ü╟
<nαq□Å□x▄└π□-7≡╕ì╟m<nπq□Å█x▄╞π6-╖±╕ì╟m<nπq□Å█x▄╞π6-╖±╕ì╟m<nπq□Å█x▄╞π╢¿Üâi

ª9╕ç▀/rq7τ:9╖ïs9╫σ8ùπ\Ä I■»Γ^û{┘╪¡▌╖¥╙ï├r

÷s░ƒâ²□∞τα
□çσ╔□.vs▒¢ïY.f╣ÿσbûïY.f╣ÿµbÜïi.ª╣ÿµbÜïi.ª╣ÿµbÜïi.ª╣ÿµbÜïi.ª╣ÿµbÜ
ïi.ª╣ÿµRÄK9.σ╕öπRÄK9.σ╕öπRÄK9.σ╕öπRûKY.e╣öσRûKY.e╣öσR7ù║╡╘═Ñn.us
⌐¢K▌\ΩµR7ù║╡╘═Ñn.us⌐¢K▌\ΩµR7ù║╡╘═Ñn.us⌐¢K▌╤╟╕TαRaó□ ╦àQ.

saÿ□□

ö₧¢å⌐;L▌ aΩ

Swÿ‖├╘─P╥@▌□u
╘─P╥@▌□u
╘─P╥@▌□u
╘─P╥@▌□u
╘─P╥@▌□u
╘─P╥@▌□u
╘─Pgÿ:├╘□ª╬0uå⌐3L¥aΩ

G╥□▐╥□▐R²Y≤╝"v½(n□┼─Ω²|-
ûÿ≈□ÿ█G█╒□â≈Γ}8□∩╟q8-□;τ□|□ùΓ√░âñ⌡□¡╟h=Fδ1ZÅ╤zî╓c┤-ú⌡□¡╟h=Fδ1ZÅ╤zî╓c┤-ú⌡X
⌠}Z≈╙║▀è▕V£U□□UÉQ□□UÉÖ╨ O□@≈ )≤φαcÑo6■≈┘▐╧Å~~⌠≤úƒ²ⁿΦτG??··╤╧Å~~⌠≤úƒ-
²ⁿΦτG??··╤╧Å~~⌠≤úƒ-
²ⁿΦτG??··╤O┴,□▕□╠R0K┴,□▕□╠R0½□2¬!ú□2¬!ú□2¬!ú□2¬!ú□2¬!ú□2¬!ú□2¬!ú□2¬!≤□Q
□

e8ößPåC□

e8ößPåC□

e8ößPåC

e8öߍPåC

e8ößPåC□

e8öÖÿ±C□ +Σ'xòσUV╖╔Ω6i┌gi_╥8Kπ,ì│4╬╥8Kπ,ì│4╬╥8Kπ,ì│4╬╖8Kπ,ì│4╬╥8Kπ,ì│4╬╥8
Kπ,ì│4╬╥╕□cVîY1f┼ÿ□cVîY1f┼ÿ□cVîY1f┼ÿ□cVîY1f┼ÿMöra6«├□╙71f┼ÿì

╥ï≤□^32φ╓ëJ/Φ⌐à Sì╪╗_gÅΩ╓T╡%U[Öj█«╥

Wi□╤îw:╧l╙┐□≤=ù▀Φ^╖ä!Ö=Σ∞ó┌□2¥G}Ωú□.Px⌠]╗ª!┘=$╗çd≈É∞−Æ▌C ƒ║═É∞□Æ}C▓○H÷
╔╛!┘7$√å■ƒεèJO+EJ╝≥╬s╦h□▀ ^æK√ó»╙╢æ╢ìⁿ□Σ▀ mKO6¥£ÿB▀>·÷M⌠┐┼^▀π□ß^;Ñ%▐{
⌠╤╡Å«}tφúk-
]√Φ┌G╫F║6╥╡æ«ìtmñk#]□Θ┌H╫F║6╥╡æ«ìtmñk#]□Θ┌H╫F║6╥╡æ«ìtmñk#]□Θ┌H╫F95(°□σ╘á£
□öSârjPN
╧⌐A║≈╤╝Åε}t∩ú{▌√Φ▐G≈>║≈╤╝Åε}t∩ú{▌√Φ▐G≈>║≈╤╝Åε}t∩ú{▌√Φ▐G≈╛D)╬┘┐

sp=n└▄╨7í±▌²òPî

ì¡Äª╟^░π|Q^╛□□╞^ u▒=÷□·░8╢74╟u╬°)₧^O
+πgä⌡;□¡ⁿìΦá°┐D╔²□S╪ƒ▄□6rlÖδ«└ï*αÑ╨□['╙_╞+ε╣▐±╒░5╢╤ôn½╗╡9╢ú?Ü□□P⌐y{▄é¥╨□╞
├p<□=±rL┼Q₧■O
╜±╙┬₧°╟q:╬

à°YaG▓*dôùäªΣe╨#Æ?p╝2lM^□=!9╧▒╞q>∞íô╡01ô╖CU&□√²▌▐╙√Æ≈{╜□□╗╞▓░7·ñδ»─□ä=╓▀`
ò≈Ω╜~╞QL╓f∩╡`□:╝Na½ƒ╗╨π╝┴╨ô▄â▒╨SyX╙U-ÄΘ≡tXΘΘ░≥D∩╧

Mò÷⌠ò╓Uys□¡╝=∞⌐╝□□α±Éï■~┐¬¥|*R╡â¬âT□ñΩ[T▌I╒□U;¿║ç¬□TφáfüÜ#╘□íΣ□%G(9B┼╜T╟S
1O┼<□□)╪I┴

□vP░ôé□□LQ0E┴N□ª■B┴N□

RpÉéâ□LQ░ôé¥□□ñα □;¿7H╜AΩσ⌐ù°▄
┼≥□╥S,O⌐<Ñ≥ö□ñ╘□ÑF(5B⌐□JìPjäR#ö□í╘□Ñ:÷+╒I⌐AJσ)ò°T₧R#╤±▒°┬╝╪Ω≡-
öjÉâ√(⌠□Uv╫║àKσ┘∞╪@xDv□#6j°╜7|V₧²!-□δΓeß°±d╕B╢╖┼□
╟┼Åì╛□ @╕Vµ h°□╒-ù²τ╩╣çΓƒ
≤πgçï÷ uVwⁿ_┬ú±
┬╠xuXS·√%Q²^Oz┴öx»ä7▄±M~ls╟┤;

╕ΩÉ+εp┼▌jΘ,╡⌠□OäOq∞à╨ΓSÑzymóF·ú≈·⌠&ƒ▄αô;¡-mm
Wh¥¿ç3B½O╛□6°╘¢>⌡┤O□Ω□█»{ó~=UO╘≡▒Ω⌠□»O
█|¬╟*╫E∩òY{&>╢Nf╝î⌡2µUƒ▌(½Zφ"█□█├N┘▒Sv∞ö□;e╞vÖ▒]Vlù□{d┼-Y▒GF□eDQF□e─vÖPö
EÖ░ôs;9╖çkÑ╬▀-
□`=eV╛╠²₧r▀▀ë⌡□¼□πtφóg:y}(╕■êδÅ╕■H≥□»□□□«3□M÷⌐Q+┐┌'vö≥▐N°)╜d╡X^□═█□k╤GJ□
n
□║╡╕nçδvD□╕δbg/TS╜□┘≥╖Pπε5>9LëqJî╖B/%□%F≈╫╒(%Fc⌐░┬□δeRs,+{*pX╕$>¥□GαH£□«ë
ƒê□ä]±□≥·d£┬═║╟?τ≈gOⁿφ≥iVsÜ┌δÑε(uG╒^/àG)□(□╘^/□j(□(▒ÿ□ï)▒X²⌡R{£┌π╘-°vP□╜Ω
»ùΩπT°V
σG)Vô\«□¡└|ßÜΣ:╟╫╙äì╪éN╝ßw▌Ä█]cG╕ª2□□¿£□VTûí□╟y}□fΩPï┬b5╪╥═±╩√┬Ä╩√▒□□biX□
%dΣêl▄┴Θ╙uƒ╖uƒ╖uƒ╖╣■Iò■╢J□[Ñ┐¡¬▀ÄÄßG╔╦□φçh?ΣSez╘░-5¼G
ï}T∞úb□≈É╕ç─=$╓!▒

δ-├≥{Xo□╣╓Qδ□╥+å⌡èa╜bxRà;.Æ□≈q□-≈∩Γ■]▒5□m└
ßò╪:S±e╝□-ù□√b¢╝▀*╖RavlKx.╓ë¡Φ┬□╪□nÄu;ε@»kεtLú□²╤"┘R-
╦°y□▓2o╨1ç▌ßÜ╪□å²<é=íZoj╓╣S:wJ□□CÅ┌□█τwoßφ░&÷G╟`□OB

Ñ■5Y╢M±sÖ>U□□╞□~NåY□²∞@╟âp0

┴aß,┘zPtsl=O╢Ptsg╣▐□□Oÿ□?┌∩Ä┴▒╤7π╟9-Å□⌠╝□±ü≡»±ô╝■+|╨δô±!?□□°ä/Ωæ ª│,τ┌"«-
Γ┌"┘■e²≥÷°'£≤Iⁿu°Qⁿo□?à│┬é°°□?â╧åo⌐è≤Γƒ≈≤┘ßjò±ì²□1╖\à╠ë_□□□ 6¬├δ·δ»ô╒í99□
Wå}¬dƒ□╣Kà∞ô%ïd╔"Y▓(╣╚∩□ä□╟-°
nïª'o╟O▒╪·≈z∩>▄∩⌡□<α:□y²3╟G┬¼Σcx□╦┬M╔_ä9ª┘éΣS^ □┐╞≥p«¬:╫ä[
□□╔└E÷□7Ör
Æ □~ö\ì°¥≈î≈Ptsu▐s~^â□∩»≤·□∩»w▌F∩╜è╫╝╫äìhv¡□l┬fτw87à-~╫
▌[v/R╡τ&╖àτTε╣ªΦ□╒{PtsΩ=7┘δ=9ÿöâ╔7!□ô²□□kô≥0)□ôY╚┴Σn

aX□□A┴╧┼░&╣□π~~□r.)τtààò≥«R▐U╞├Ü╨╔ÄS┬l]b╢.1╗r¬╫╙tÅ□╚┴╨dX[Yë□ⁿ|

≥■┴8□çz ░É2ΘS&}¬≥□╫;╥9Gß=8□╟α╜╬=╓∩▀Åπ▄ x∩Θ░║╤┬╧□íYà/¬╝9Ü^╔δJ^W≥║≥V▄å█²εε0
Gσ/╥┌╬╒┌╬╒┌╬╒□□ΘVτV>Σ:K¡√□╫│▄⌡ùy²
<ü_åkóπtë½uë▀LLµ□'µ·╤:Afè_¼▓┐Ñ▓W½┌ò¬vâÖ¢W▒╧½╪^U┘ó□□Uß□U╕Y╒²¡╨·╢JZ⌐bnW1/½ÿ
>Ur»*┘¼□□d /d Wd Z┘_·'□>!π_Å■¢~⌡ñòⁿ┌─┌□[iJ¡╓□~τ╜g≡ó9≈Æ▀¡
φ║g╖╓┤V╧□4╣V¢üâV;`z¡6╜Vδ_╤¼ⁿe}j└╨7ΩEδ¼:Ñ▀∞╨ovXyƒ~▌jσ╖⌡∞V=╖U?Yg⌡╤⌡éσz┴r½▄g
ò X┌≤ÿ^¢Æ ª╙^□V¢`½M░M&╪j╡9¿6□M░MΩ≤I⌡9¿>ƒTƒO¬╧'M░M╔□}ε╟╕□╖àv]╝]WoW¢âª┘&╙lô

▀«├╖½═'M│╒j≤I╡┤\▐/ùτ╥σ⌠Çy╥j₧┤╩█□3ÑU«

╚╙u≥rÖ╝\&/ù╔┼□╣╢C«φÉk;Σ╥Ç▄□ÉW;Σ╒

y╕╫,jòSδL╕ ╒rΩI□nô╓╤.?û╓Å□·▒├

rì<h└
vh»ä▀Qz°Θ╨"□╛ á¢wΘµ]≥ßU¬÷P╡Ö¬═rΓ╖:≈6╧«╫┌╖(╖₧▓δσ╞.╣±ªn╝Y7▐¼□oû#-
æ#c║l°.█)W╢╙ô┤╬┌ñ│6Θ¼MrªM7▌óïªt╤═:bïÄ╪B⌡¥T▀Iφ¥:`ï

╪ó□╢║ΦÇ−:`
ewΩz−║^ïN╫óúÑt▒N]¼S□KΘbM║Xô

û╥┴╢Φ`[t½–
║U°ε╘⌐;uΩN¥║Sôε╘ñ;5ΘN[tÑN]⌐s□Wj╥ì:uúön┤Ö;δuû.¥ÑïKδ9┤^w┘ª╗l╙A╢Θ□]║Eù╬╨Ñ3tΘ

]£jµT3°Üuàm:@□°Ü9╒¼≥╖8╡^σ╖¿°□□▀óΓ[T|ïèoQ±M¬╜I╡w¬÷N╒▌⌐┌¢T{°j∩Γb│*∩Rσ]¬╜Kòw
y&ε╖;.φ½╧□oEg¬▓╖s╓e*jëèZóó^Σ≤BU│ù»O≡╡₧»⌡¬%├╫^╛«αΘ□₧«P□EUPΣ┼B^,T□E~,ö±EY╛D
û/æσKx▒Pû□eyQû/æσKd≤^z¡á╙□┘╜ùV+h╒K½^Y╜ù^╜2y/}ΩΘSOfz·⌠╨µ╜▓y/ìΩiTOƒ□▓╖({ù╙▄
╜b«□πKßv□;&é5^φ▒÷|xJnnï▐#▓=^ÑE6 ▓□æ
ë¬I╚ê¼IdMV╖╟ΩÜ¼«╓Ω÷X]ôUφ▒ó=V4`E□V4`5{¼fÅ╒

X═Ç╒4YEΘYv
:╓¥≥ε┤┼¥╥εöv°~□ûPtsQ¢▌m╘▌Ü▌¡┘▌≥ε╓∞n═εûw╖fZî╨b─]≤┤□qτ╝;°▌9φ╬iZî╕{▐▌≤εPtsv≈┤╗7╗
{Θ·0φ□a¢~╣'╝.Ω╫▌y╘□╗⌠▓gt▄

□╖⌠|≡█ëÄ[µ¼╤²╧PÖ²

╙⌐±
óÅO(╫π7]~╙3±¬⌠l╖oB╟)√?5ΓU╓⌡█]□╪n8eO¢Ñ≡╕8+(□aè=i□╨q£╫'ai□rìm□╬┤8{½)RZπhtÆk
╝∞7┐ú▀êk²▐□o■Θ·~b▐D·K9ªó"ⁿ^T_□═wΘ8B╟mt▄F╟╥≤⌡6·ìX├∩¡ßekx┘□^ªσƒ?w-
ìc▐⌡ⁿ}£≤OTï'9.u■#▐+=sO□s.:┬·å¡i╪ÜvY╚«²▀α∞╢·□δ┌m]╗¡c╖u∞╢å▌ε=∞▐├ε=∞╛╗▄wù√εr
┐]ε╖╥╜v╖╧░{∞èNt⌡gE □æ»□WùmÑ≤rw*Lt╒èë┐□·±~/╖ê╛║⌠□=□Ω>"^∩«╧║δ│ε·∞ ▓≤ö:=q╬+u
Öô□K□c⌐s ▓cL¢ÿó{∞□÷z╢.πδ╫├ò√ ║πuw■µ─_î~▄║╖9≤╖\k≥\╨n²╧Siσ╖:Hi2ñ(╡ö╫Ñ╢√&╡ûR
k⌐x₧w╒[]m□□¢∞▌┌)╕öéK9┘D┼Ñ*"Ñ"R□m□▀≤¬"%╞mb▄&╞m\m▓□k┐□k╖▀j ï╫æΓr□ù¢▐Θ□╠╣╞ëa
⌐╪ƒ□≈6.7MtÅú⌐╛òΩ['╛ì╚δ"{├KV=H·¡V<h┼Ñ∩p□⌐╜ò┌[¡r╨□□⌐╝ò╨[⌐╜ò╨[⌐╜ò╨[)╜╒¥□)╜ò║
[⌐╗ò║[⌐╗UUσu▌q╙O÷╙░|x>èÖéπvJ{ú╕▌╚+^
{╒□□τU╬3L╤■$g□Æ3)╟L╨1ôrl wä□{û!√°óëù1Θ2&▌ÿI7f┐^4φ2÷ΦE√è£=y╤t□3▐╞L╖1√εó}w╤
d□3┘╞∞;r&[╞▐#g╥îÖ4cª╥X4═,▀k%□¢▌93╗┤»{╙]s□|£âÅOtòiª²hⁿ0¥ΣöÉ□┴Ç│▓±3ú□u□╧<╤i
εôè&╗╬N╫)}τZ,E ΓΣ─7□Ö╥·ö8L=¥□è▐/}+δ

ƒ█□□εU)·Q╤Åè~t"≥
φ□╛□┌▐□·¿╚G'ónvl┴&lE□D'▓Qæìèl4z┐╖mñoPts╛□⌠φx≈ô╣{g▌%M█╝;ñ▌!²╬╙°¬ëoⁿ╥┤═╚╢â╢·?
{B∩≡:5±-α─ô:m;▄=M█Äw?¡GôDPtsÅNîW·Θ░≡ê▌R╬n)g╖ö│ªº¡Θijσφÿ□∞ÿJ▀«
╥iù¥QÄ□oqαW□°òτ╙C<Gû■:▓┤δ□░δ□░«°φn□∞n□∞n□∞n□∞f□∞f□¼τi;Ö□╖ÿ£5=mG1`G1`G1`71
□ò[═o▄yÅ;□▌qÅ╖φu╖W▌φ╒Φ□┐▌N╖>k▄bì[£Y╪ □÷ pΦL;╗│Σ⌡┘tX□·h8N├±w\Zσ╝z»ƒq|╓Nδ□╟
w╗╓ßu□□r∩
τ⌠8□G╪≥g.N°Z□╒z¿╓C⌐-J⌡Xw≈■∩ñz(╥Cæ-j⌠Púç□=╘ΦíF□5z(╤Cë-*⌠Píç□=TΦë▐#╬7─°å□▀□
πn1╢èq│□7ïq│¥j)δ6ïg│]e╞«2#û7∞,K□╕Y,¢┼▓┘N2#Ä═Γ╪,Ä7─≡å□6ïa│□6Oⁿ Sö'─┐□¥□-ë.

□Dùα{╕&<□═
wF≤≡C╘`>z├Æh'╥□q╬▐pG4Ä}x
oç;&}04O:□┬ç±□£éÅΓT|

°ßπ8□gαL|□ƒ─_πo≡)£àOπ3°,>ç╧πl|□_─9°[£ï┐├y°□■-τπ≡e╠└WP□□1imx~╥
ß╖ô^─KXçù±JX3i=6á□»å5ô wN~□Åí╔δìx□b¥ⁿGäp╟öâ┬□S

Kª╪eO▒╥₧bù=σ□□ëú╨□ε£Æu╫ å┬¥e'π□╕<<P6
Wα□ÿ□--╖

t/[□Ü╦Ü├Ü2O<σ'à5σ□à□å■ûƒîÅπt»?ì
├Æ≥ï≡φpG·²Xå-»╖c□xV>□--╧`╖■ìz]□wLìàµ⌐qL╞□ö┴Nq¬¥Γ╘i¿@□ITΓ□□êâp0

┴í°¢░fΩ°≡□?□╧qíπ/□δ┬o°µC≤4╫Üv¿²±╖óC┬╞ΦPΦ~╤ßÿÄ#≡W° N╞ç≡a£Å□└ù1□_┴W±5ⁿ#■
■└7qqxXµ>,sû╣≤úk├╥h6«├□\Å╣íN6╫╔µ:┘\'¢δ& $l£|+n├φ°)□π

▄ë╗p7ε┴╜┐□Å°▄úx,╘q²ß)□aπö.╝ün⌠x M╟>d²~□C▌{;l,+C9ªí□GΓ(|□'ü

etÉ□ueg8~┬±,╟┐├╖≡m|□U╕<<,s-û9□╦£çe╬|Ö3┐L╝eΓòAuS□P╥&‖34Gwßn▄â{q-
₧└/Qç'±□□±*^C□6Γu4ú□¢░□¡hC□╜aò₧░JOXÑ'lê÷`□y□0å╜aÑ>▒RƒX⌐O¼╞'VNε□═ô□É┴.dßΘd
r

╗1äaî└□╦ΣQö>≈Gä░R╝¡*╫
╧╒~╢Z/WδσΩ╝|F╪P■╧Ä_╟à╬║□▀

+╥/≤·Z╠╞□\ÅΓ&■

⌡VNúr□ò╙¿£FΩie·╧□ù9«t|□t(°C9□╨Θá╓V⌐╡UjmòZ[Ñ╓6¿╡
σ╗É┼nƒ□⌡>=╘▌╨Iì&G□GSPårL┼4T ü$*q□>□MÅ╬┬┼a₧□ƒ'╟τ╓±┘r|ª□ƒ)╟g╨±Ör|ftâ+╠
│Σ·,y>K₧╧Æτ│ó┌Φ└ΦFⁿ□7ßfⁿ;n┴Op+n├3╤√ó▀ú7╠σΦ\Ä╬σΦ=□¡πh□Gδ8Z╟╤║¿⌠ 2╣7╘p╡å½5\
¡ßj=ñ□C█ñç≡0~åG≡(-├╧±8ûß□x□┐D□₧─S°□~ìσXüò°□ⁿ□½PÅ

m▒ÅE□╞Nïª║╟╬p n □╬
≤b_□╫─╬║╫╝«

ïb3├σ▒╥pу╕▄₧φⁿ°EßZ√╢≤π▀q╝64╞gçûxs4%▐□□□▀l╫█µ⌐╜=¬ê≈å║°N{æt⌠┴°¢Ä}Ñ□7Éπ«ΦÉ╔╫
F□O₧ìδ0□╫π□╠┼<ⁿ□5ÿÅ□x$╠╥/fΘ□│&oè

£╝□¡hC;:É┬□tb+║≡□Φ)█kd{ì^3o╩┴íM╓╧╒cfM┘□UΦ/≤⌠ùy·╥¼)√óâ╥ΓÉ[eçαP£Çô├¼▓□9₧å╙ú
Θz╩¼▓O··≥0O ÿ°□╠╙?µΘ│⌡Å┘·╟L²cfÖ*¢
╣T÷@h+{pΓƒáo+□/▐çc±~£å□íNÑ═UisUZM·U╤üσWc!□ßN▄∩²G□ï▐°Üj╩□στ-τo╟

╚9òsÅ╝╣GσÑ┌─£║≥┴hZy

╗¥?Ω≈≥O□╒öÅE□N=,┤M=□╙q□Ä─Qx□Ä╞1░╓⌐╓:╒Z°Zδ╘πp<N└ë°□╛δZ□π□╘x=
B█┤Ií¡ΓépM┼à¿     ùW,Ç║⌐P7□ΩªB▌T¿¢□uSq;~è┼╕□Γ¡╕
wπ-▄ï√p?ûα□<êç≡0ûΓgáO┼úx

?╟πX□□ÿÿçó□≤▒□┤M╨6±#¿∩ä·N¿∩ä·NXg┬:□╓Ö░╫äu&¼3a¥ δLXg┬:□╓ÿ░╞ä5&¼1aì
kLXc┬□ôÄ

<`□*É╨-
b±╫UJ»nT·⌐⌠∩-9"6G7KΩfI▌,⌐¢%u│╥ wUR/JΩf╔ë TΓ□□□:φ□:φ□:φ□:φ□:φ□:φ□:φ□:φ□:φ□
:φ□:u╛Cu╛Cφ□2v□□;üî¥@╞N c'É▒□╙╪
d∞□2v□□;üî.y⌐.y⌐.yi⌠²Éï¬1□ùßr╠╤□°□«─U╕□╫äj□⌡J□⌡J□⌡J□⌡J□⌡J▌⌠□▌⌠□▌⌠□▌
⌠□▌⌠□▌┤B7¡╨M+t╙□▌┤B7¡╨M+t╙□▌┤B7¡0w╖╠▌.s╖╤▄φ2w╖╠▌.s╖+*}▀Qç'±□₧ëÄ╥yÅ2□sµo╬ⁿ
═Ö┐9≤7g■µ╠▀£·¢3□sµo╬ⁿ═Ö┐9≤7°[_Ñ[_Ñ[_□⌡yûφ╟□2╪à,□æ├n

a□#ß~¥²     ¥²    ¥²    ¥²    ¥²
       ]²□]²□]²□]²□]²□{·ö=}╩₧>eOƒ▓°O┘╙°∞ΘS÷⌠){·ö=}╩₧>eOƒ▓°O┘╙°∞ΘS÷⌠){·ö=}╩
₧>eOƒ▓°O┘╙°∞ΘS÷⌠){·ö=}╩₧>eOƒ▓°O┘╙°∞ΘS÷⌠){·ö=}╩₧>eOƒ▓°O┘╙°∞ΘS÷⌠⌐I_ìªO·□■□
ä□╞âí╒$j5ëZMóVô¿╒$j5ëZMóVô¿╒$j5ëZMóVô¿╒$j5ëZMóVô¿╒$j5ëZMóVô¿╒$j5ëZMóVô¿╒$
j⌡,Q∩YΓ9╧□╧yûx╬│─s₧%₧≤,Q∩Yó▐│D╜gëz╧□⌡ô^ï*&5a#^Å*L▒ñ)û4┼Æ▒Oò■□U╟/:₧□□ÿf3L│
□□╙∞óÉì]îj╙φ]S-6+dM╢╧ÿl3M╢╧ÿl3=ï/Ä_□û╟ƒ
/╞□ó□Γ/ÿ~»{₧o±£┘9:┬ö╥ÿr±xçτ√ ÜtSL║□'■□ô□∩∩2y«ìÆª\╥öKÜrIS.i╩%M╢ñ)ù4σÆª\╥öK
ÜrI;Θî¥t╞N·┐Sw&≡Qö≈  ╬╠ε╟f3□B□□"≈ΘQ-
Xδü╓┤□δ▀[½¿□ATöb±@Aσ≥¿⌡B□<AA□A¼bæè□çx□"Z9□äEÉ;!ä
       ≈ò░╧∩=╧nbB□!üj │»╧╟3╧<╫<≤}>╧τ;3╖¢ÅÆ╬GIτúñ≤Q╥·(Θ|öt>J:-
%¥ÅÆ╬GIτ□å+/0□╝□^□#┴(≡:□
╞¿

╟£□ÿ9;αwM┴∩Üé▀5àY4╟,□f□
3ïåÖE├╟óaf╤0│hÿY4╟,□f□
3ïå╤Ö-:╙CgzΦL□¥Θí3=tªç╬⌠╨Ö-:╙CgzΦL□¥Θ□v½é└-░□∞□√┴□P□è□céÖ╡-
3s?fµ-╠╠┘╠╠╝± r≡ r≡ r≡ r≡ r≡ r≡□óx
Q╝ä|╝ä(3xçα□σß)D±□ó╟Σ=ÿ╔{□iSÉ61úw`Fw±□ó┴□√Jy╢□□ÿ└□ùÖ▐┼úêΓQD±(óx□Qf~
ùÖ▀┼│êΓYDφåñm□Z□╫è²╓□«┼╤êó

:á

\╗-╟▒A╘A□╝Ä(□í□□┴┼≤êΓyD±<óx-Q<Å(₧G□σ╨□σ╨□σ╨□σ╨├åGmx╘åGφ√@-
╨W⌡DM⌠DM▄âÜ╕□□╤□□6□%æìÆ╚╣Gδ_d╨░'â□⌡»2e╪s╪.TSP□┘6╫□┐7╟▐+□(Äl□G6è#□┼æì/<□_x
□╛≡

|ß□(Élⁿß□°├S£s%îO<□┐└├/≡≡
<ⁿ□□┐α□T╨□ⁿ□□┐└C¡⌠F¡⌠vnT□N□╨U⌡├?≡£^ä□S╬]αnp□ΦMÖ≈□╬
▀ß□|□□▀┴├w≡P8a□N□┬├ç≡£┴ñ□F ¬áçΩ πOx°□-■äç?ßíé·íé┬¿á□°□-J¿-
J(îoßß[x°□-╛àçoßß[x(ñ▐(ñ▐(ñ▐(ñ╟□╨▐□6□╕▐üδQM├QM├QM□PM□PK²PK╝QK□PK²PKa|²□|
²□|²□|²□|²□|²□|²□|²□|²□|²□|²□|²□|²□|²□|²□|²□|²□|²□TW6¬+□╒òìΩ╩Fueú║▓Q]┘¿«l
TW6¬+□╒òìΩ╩Fueú║▓Q]┘¿«lTW6¬+;t:m·
8GM ╡□7Q÷¡∞w□╖ü█ëδ┴÷»á'╕□▄¡≥Qh┘(┤l□ZvΦ□≥

%■m╥■S=□╜C°]░[σ$ëdáα▓ô8╖ñ:jJR]     ç»Qδ├╫éδ└
Ω□ö▌□ß□
?¿□┬²└□Pó⌠-%ⁿ8xR\□ƒïΓsQ|.è╧E±╣(>□┼τó°\□ƒïΓsQ|.è╧E±╣(>□┼τó°\□ƒïΓsQ|.
è╧E±╣(>□┼τó°\□ƒïΓsQ|.è╧E±╣┐áΓs╥)╛║2D¥gtò╥ìnrìq│<h▄"□□╖╨yFw╣▐ⁿ□râ┘S«│:¬
¼□╘¼ijé5S]n¡S≤╨åΘ□

gmR╧[yj«╡YN░≥±╖╢¿=╥Då─f╔D╡Xf½┼ö~~Γ╫`╧ñ⌠S(²□J ╜╤Sφan▌H-
xsxe□U{j·□╡⌠╡f¿Θ╔g`f¼└·B}─□╖▄·J═▒f⌐!╘■□j▐gmT╣╘▐₧┌çR╖Eφú⌐}ûä¼·j╝╡É6ß╔[ï╒¡╓
□5╒╩&╫2╡ÆYq□:uó·Ü╡║}M╩┐0w╬'⌡pR□░□╟bñ-KΩïÖG?"╟□ΣxM ╢π»iφ
f≤F╞▐□¢ù3ô≈T=╤╗─2▀E'╧R╖ÿs╒□s╡ⁿ╓▄═î£.╡¼_½╖¼□Γ2K Ü3°75═┼-
╡¼┼°ÜK╒ç╠╥AJÅqF┘╞╘□□3╡ò≡I-
╬,╫┌╟Yσ□┐Em5«ùÇÜ*A`□□ä@□□âdαé□H□╡╘tI□φ╒J9□<ª&╦?└πα ≡$x□<
□âg└│`□}8U-Æijæa¬òå□□ □lαÇ□H□aÉ

" [illegible]╘[illegible]iá

H□uA□¿□Ωâ□á1h□Üéfá9h□ZéVá5h□«R½î½┴ƒ┴5αZ0□<□-□ÅÇG┴▀┴cααq≡□x□<□å⌐□╞≤α□≡"x
╝

^□├┌□│¡Ül₧□▓└┌ΩS≤i□5□½(V▐æ½RÇ¥□ccô╣□□╪╪ò╪X▒╡'ûgφeD∞SÄ╡?┤╫:□[i□)█*ÄσZ□Uû□#
^⌐□ü`,/`½
□Är□í╪▐@Rle ¼∞@r,7α¬¼@ä°□╥⌡QS□}┴²α□≡ Φ□·â□`
□□-□□â7╒╨└80-╝□&Ç╖┴?┴;α]0□╝□■□&ü≈┴d≡o≡□ÿ□>□üO╒¬└T0
L□3└g`&°□|□╛□_üY`6X¼&□ûÇl░□,□╤A

X□ó`%°□¼RôâEj¬m□∞╫

¬Θv□█:á□8□┤□┐Q+φ│╪>½V┘»Ç□∞SPts÷[ä9¢≤▒9¢≤▒▀'n2°□L□ƒÇ┌─O□╙┴

@█m┌n□K°;≡┬▀â·`□X□û½□vöc╣`
╪

vÇ¥`□╪
÷¬UN□¿□RAmP_¡p□ÇLp□h□╬P+¥│@o5┘╣□<□-□╧â1`¼ZΣLd╗WM

╡Q½B°¿òí╙╪║e{□╕Æ≡_╘è╨¡□∩

n□O□?é°W┴k`$ÿ□è╘è$Q½Æj│e|%1«Æ2AC╡2|½èå∩□╝└]α-╨□0▐├î≈0π=╠x□3▐├î≈≡s`□□□å□┌□
~□╝□^□/âW└p0□╝□^□#┴(≡:□
8╟≡□`,x□î□π╒ΣΣKT4·Rp□╕□\□«□Wü½┴□⌡i≥@0□<□-□ÅÇG┴▀┴cα-
αq≡□x□<□₧□â┴3αY≡□□□åéaα□≡"x ╝

^□├┴□≡¬·╘=EMNIRƒªäA▓·T□╠□ôa■|k⌐£□/□╦╦╥_ìö□`
□□-□√U□ 9è □┼□ÄΓ?G±ƒ=ⁿg□ ┘├□÷≡ƒ=ⁿg□ ┘├□÷≡ƒ=ⁿg□ ┘├□÷≡ƒ=ⁿg□ ┘├□÷≡ƒ=ⁿg□ ┘├□÷
≡ƒ=ⁿg□ ┘├□÷≡ƒ=ⁿg□ ┘├□÷≡ƒ=ⁿg□ ┘├□÷≡ƒ=ⁿg□ ┘├□÷≡ƒ=ⁿg□ ┘├□÷ⁿ_ß2╛ª¥sU□>k□>k□>k
□>k□~Φ□ⁿ╨□°¥K≡;ùαw.1╟½<²~dⁿ¡ú╡µ^╡û┘,çYlñ╡@Ü0_«a□{□-
n$>▄H|╕æ°p□°p□°p╛ □┼□èΓ?E±Ö<|&□ƒ╔├g≥≡Ö<|&□-
i$~╨Hⁿöæ°$#±!FΓCx°□□°□-~@□~@üs▓è:°Φ▀π,@√√Z>è╬Äó¡úhß(□8è■⌡╨┐-·╫C zΦ_□²δí□=
⌠»ç■⌡╨┐-·╫C zΦ_□²δí□=⌠»ç■⌡╨┐-z╡□╜ZÇ^⌡╨¿□í╛ö²□ß╖²_MS-z╙Co□$Ñ3₧nP#╨ÿ#╨öK╨öK
▄A*╧}□<¼≥"Θjmñ.╚□M@S≡(±π╘Z1ÖU▐c^G╟Y╙Σ□k║t▒>ù3¼/ñ>²√ë⌡□Jjû╡▒µ╤□⌠⌡□°⌡A□├·°÷
iV╢£N┐ êrhî╬YG∞z9□╜p□zí╡ò'□ó▄»□≈▓Oíª/╒D╥┐¿δ£╠▒;P□╙%à╕y∞-
≡□ù▓ΓoΘ□=%½≥▀╙Ñ=φ□□τQδe╠ç□╙åxL;f╤╜─^└l9¥┘2_ F±□1¿y╜4d∩|}O▒-i[╤□ ┐□6╔⌐ñ8ì╜
□Æ┼□ªs¼1τΩ Ω▌
Ω{½Å┤°²_□~ç^3ë·å╜∩H═▄ä&,do□{╜$┬▐□÷╛æ6□É, □□8
□Æ@□$□□D@□5vö║V'4^W╨ïsÜÄ

n□¥·ÑZ□Φ#Yü╛α~≡□x□⌠□2┴□0□

□□üç%
_>
ƒ=
ƒ=
=
=
ƒ<
 ;
■;
□;K  E□u╗ïÜVq□¢¼╧╢Æ■┐Ö|⌐>F▌nß▄√╨'╙h╫gñΓl9≈êñ□
Ñà▒H┌╥3]Θç?Z¥H╒Y:[]⌡o╞u╣z⌐/²_%▓εWδ¼WΣLk╕£E=-W║□JfRα□9=╨^┌╥[¥Ñ19□S╧□\=>╥öÜ
╢·⌡δÜ"ë 5Ök▌Hε.ñ∩╞÷f╢}░àj□□╢□}╝_█╧2
      æ╦□█ '□RgÉ2âöIñ⌠HQ(□▓-□EC╔Ft╚╝╘Σ_╙√╒□tw□W╝□î╗Hùù═□\J.╧⌠□q0M□π├□π├□π
#□π#□π#□π#□π√□SgGòτ□πë□Ofñ8║┤Ñjù╘+Wτìp V7p'τ╓□%╛@m°uà£çç┼╒Ñε▌ΣÜC╝╓╤╗»╧zô⌐w
¥ ▀,öûF╝AJ▄Më□ö╕ï□ô(m{Γ,è□g□ë⌡□/≡Fö|7p/G·H□r&╤b¢£{╚YL╬□më·╜F╬"F┼z╣H6Çì`?û
}□□übp□vΦêτrâjk▌□[tæ¢¼nlof{'╛╧╜┤τ~5╬□ê]╝"gc□τ╤π
⌐▒╜╛6ï╒δ║╢l╡î1ùÄùs a#°□(;□□J┌□╙Σ"°□Φ

║J□g8□□╓░┐□¼□┤╙)$n□█=┤═ ²╟BZ╢ƒs▐O╦Nµ╜≈╙▓ô9∩L╬█gî□τ□µ\s¡σÆ¬¡n□9╛"╟□rdÆc□92
╓q6⌐Si≤&myïU□φ▐G╬
:W╢■_éN╘╫□Kε╨÷&╢}a┼u╥□╞+äc┬0c□ÿ▒6|7C úÄ□²óñ▓ê)Σ:t$tâ-□■»ßeX≈aU□0▀mó▌y╘╕Yy
┌▐╓Éo□·┬ö-ódô#Qi ▌╒v╣
■

επΩwΣzvó]]A_,╙O╜-+┘DOτ╥ª=°ù·ö▓ày≥wR/ÿ¬╢□
└V╡▌ε□ε□wü╗A_p?σª$■□(çÆúö□╡επ¼·┬·δ╕Äδ▒ó
î
}╢≡p-}┤Y²G√Γ⌡h_□φ+ó}Eë│≈∩)»ªö╒öbR╨╓┤1òR÷RJîRⁿ_Ü□Q┬Z  êh_□φ+ó}E┤»ê÷□╤╛"┌W$
°Jw╡Ln□╗â■╥A□Çü`□xH:Pc-
jⁿ□£□ñç»å│é⌠≥╒p╓█⌠⌠□⌠⌠g╪Θ\∞⌠b∞⌠2δ]⌡<τ⌠□3DδxkÿπⁿΣí&╬æ÷╪h√└∩TN`ît□╝□╞Jç`¬\
□\├╢ÇφV░M:╪'ü3A/╣╠╛□▄□ε□~√B┤jO┬n╠ä▌ÿ·Z·=╕YσΩ╗□ôh≈äD¬îD¬

┌φæ≥t}□b│Zée⌠è═┬□▄è∩╖□_o+╛▌Ü└ë▒ì╪Z»ÿGl!1àü□╒·ö┌+╢┌┌C?□æ╗□n8¿µ□éj/~ß╛@▓┌E╩
·ñⁿô╬√%G□□│êÿ░╬δY□¿»ê^9¿ûΓc╞□Ibô7F¬Ñ°Æ1Rf┴K╝b¢¿%åù║ïû□X√┘□Qk1û□╧YL¡1╝╙]┤╕
□b□ª□╓─╟K*µ

vcu╜≡k≈èA)àö□ú□E y‖n[

r□Æ;FnE╬╜D□N≥√)6î6¼#w
r»$≈-δ□#╓o}1v|□ïïí□ö:H[╓QZ
J[Ii{□I*[ƒU2╫┘òT<σ|J>H¢■σ╧ó╩ñ─}┤cò□□ô\√¿{U
B°D╒╟O□[@è\Ω≤{*Jè\╩⌠{)J□█Φ▌C«□W?q¥╚]┼⌡╤i⌡u!m□╫âs<╞δ□ƒV▕ aÖπ▄∩£πa·[-
┌┤ƒ%%É.Iü║┤»╛ä□Ööv□y□ó□□□n╟▒&□k╬▒û∞╖ΓXkÄ╡a>□□2¿ß□Ä6e█èkΓ□╥┘├ç□╘ú■Lj8üÜⁿ▓□
□▀ä°f─┐$╛□±ö├U≡S√5ƒÉHß╫ΣùòF╕LÄn

d□S□╘ù╞┤/ìö□)│1φ3iƒI«ìüª□o□Ü□▀Æ4¡êkM╕
τ₧B)½h½□åfá□m═ö`ó□?≈*┌∩ƒí□h┴▒û□ïτ69▀tP□█╥á=⌡)7ôs9ü½▀É║□·τ┼±&□o╧±µ□oI\+Ä╖µ
x□╬Å│α┌╘Ñ▄

bδü·j□mê╤;δ□
╣ûì8τ╞ñiBÜª□o□Üôª□iZÆª5i┌0│·╫╔╒²Z_╥iç▀c√hG:φHª□«ε█µ∞╖╘═╕Å6ñ╙åd ¬êÑ╧=3╤┴±╓
√╝gΘ≤Äτ(L┤┌öZ5╡
F¡G □b□î÷_Kñ║╢A«╢Γ□╬>8┌JΩ□/□í┤_q╓5┤□rƒ(╡Å╒V(σ□ îÄÅ╝p%╛╒╫▒F6úτåHuφF│
·ë╓₧╪fÿ┤□î╙□V╖▄:□+ä╒.┤èc·░OwX¡)¼╓>□îmåQ╖┴F
a╡╥□I▒BXφ┬@r,fΩ

½5à╒┌□╤c{ΦæSΘæôΦæô□⌡┘oá~EÅñ╨¬v⌠JkzÑUá1±MH╫ö4═@s÷[É«%ΘZæ«5Θ┌`5Ixn.>Wûσ »╧,
⌐â┌MGΘ╢DU£ìVÿâ┌½Ñ [hÜ╤U╬5║╔ƒî¢σ□π□╢╖Γ╣wTú¼δ≡E«W╙P-ú⌠?╒¥täTst* ?Éûδ╪Æ╝╔Ñ{&
₧ⁿLπ
5Yçⁿ□╖['f□å□q/¿}ò£(Mσ8-jÜZ»6I[òGxeÑ)bjñzƒOí□╟▌U¬3yµ□╩K□╧S·¼╫&÷÷T╚∩-
═W╖°ⁿt╞⌐ñ₧¥αÑ*█;□|V.f55d°╡□van$▌□t/□H_▀ê╧,j}·6û£M%u■G¡Q₧·₧□╓q╢╣U╡±(û□Ñ╛Æ(
}â*Ps╘╞─▐Ä□╡o□½╘Å□□J]"I⌠▌╔╥¼╞±XUò┤▌\╖]ö≡S╤Θ□l*~⌠-⌡û╕á⌠□-Æ{+╪¿óö▒Ü▌
#í╡£O¿ë>:[═W╦░l□eUy²∩¬7╒h╢Oü,uÜΩ½·□*╚Å%gO¿áBεÿ·ZσbA_½∩h□╫┴∩╜≥╣J╙■°è«□öäHè

I─xö² }ëmû╡èD╟.╬|□}┐Rφäæk□u□Wí┤v╡E_í-
%⌐+Σ/P¢□c^IÅ√╛½▐■P6MUφNñïû█δ]n∩¢ú+âÑ¥Nƒ░4╡£δ□R╦½¿yoÖ▒▌N╬«"⌡{Ωƒ■êV_-
u¢╧τ▀Σ[ço│□Ä,=è▄£ÖzBç>>t<½[Ä"?6ó>╚╝╡┌┐n╒]╘;ÜM▀í_+.íú*íPM╙¼yövQI
;Ä▐¬*╔¥`X╡┐F╣'δ⌡rƒ9Ä√≥¢ú¿□S|.SE╪╤╬j╫α-±h□≡g]K╔î╖6■I□oRI₧ô°4ßsR╡VNHl
□─?G╚▀«╥ⁿë▐┼Jv├N╗□╫`°s½┌

â¡╤c╨╖Ω}:■E}╕▒·B═T┘■î~ÿⁿ┼e┬╧H□° z╣╥!ë╕U╠
╙+rqi₧ó2ßa╟<╡ΣbΘFxR"n=╜╖Φ≡│jI²┌ó_%□□∞soé╔²°□╘√b⌐O

¢ P+

óPtsz□ \Γ°7j.² mb»"□(□-Lε□r╣°J(+□≈ÖÜJ :l²□*ÅÅq┼|~TW½+Twue"⌡ÿ□·

ï╜Ñ■Ñ□¬∞2╤ªtæ╟ΣYBCd¿ V│╝çσNÆOPç╙eª£._≡9Sf╓29KV╙F╣Tr
Cn0║íM∩3»6 ,}╠╪fg╣▀∞n ▌&□ÿwÿwJ?3╟╟æAµJs╜<dµÖy≥ñÖonæ°╞Bs¢

6≈ÿ{σY⌡╙,Æ!ª▓DåZªe╓≤ûc%╤ïV□½ëî░n┤║╙½V7δf□□°8≡▒╝-X□P2:ÿ□Lô ╪┘-
╓≈÷g÷LÖo»┤□Éà╢▓ò,v■Φ\$K£K£+$╟╢╧╢ZV9╫:╫╧Å╫u╫⌡▓╞ΘΣ▄$δ£[¥εÆτ⌠tzJ╛≤7τA┘Γ⌠w-Æ²
╬#╫ú□s₧p₧1─□Ω<o╪╫ï╬□#╓y═y═¿σîrF□⌐╬hgîQ█□τî7Ω8□¥ΘF]gû3╧hφ,rv□°:╗□e\□▓BAús(
□□□]Cn(┼╕9T;T╟Φ-¬□¬g⌠□eå□□wä□çÜ□wåZäZ□≈ä╬□e□≈àn
▌f⌠
=□z╨x0⌠LΦ□ú□hRhÆ1 ▄?▄▀□□-□~╪□ö<
yÿ±h≥éΣ□╞p7╒¡oLw▀s▀3f╗δ▌m╞□w°╗╧XΓ-pïì□n,"╞□□3b□?F□□█X□ E"╞·Hj$├╪□⌐-
⌐olïdF□□█#ì#-ì]æ╓æ╓FQdddñQ□y=2╓8□□□y█4SÆRÆM'ÑnJ=3£▓/σÇΘ▀⌡Y«-╞╨□cb1»αQ

ù╫░Θæ2₧ÿ╖ °82A ▌eûÜê=┘ ┌₧l∞i□úε3¼*¼¡ *îU=#■[╔ûdY╩║─╨ûí¬W╚□¿½U▓Ä1╢-¢k&╣▓¥□┐ âOs
┘){Ñà∞π╙R÷╦Ai%1,▓╢╡╙F┌"-mæ«╢H□ï∞%⌐µ¥╪Ñ½φ2
╗\%□µjs╡ ╘1□4╫J=s¥╕NΩ¢δ▒╫å┌^O╨÷Z_█k]m»Ö┌^δÿ╩T°v╚□╝;╙2Y│H]l╫!╠┼ù□V□v£«φ°□∞°
Fimu┴Ü█`═▌□▀îM╖╤6▌□¢^%F`u`#~εª@«╪ü╝Çç°\□╪%ì□╗□{≡╢≈□èÑIα
╓▀J[□3m²ì┤⌡7╥╓▀H[□#¼ ÅÆεtp:H▓sísí□£?1-éîçKê╣ ╘╣öÿ╦£╤─q.w.ùÉs□πñ□πΣ*≥^═hI╥ú
%Ö╤r¥D£δ□3)îÖ╬╥╠╣╤Θ"╡£«NWiσ▄─(¬¡GQm=è

F╤▀╙╓╥╥╢ç4╜¥{ë╣╧╣OL°Å╙ùZεwε°Σ□□i╔î┤□Σ□Φ

$~É3ê⌠□1÷"z∞□î╜'I≤ö≤4⌡

v₧ßΦPg(1├£aΣz ▌y₧4/:»□3▄□NKF8#êa|J╪-
ƒö3┌□M«1╬□Γ╟9π(g╝3₧ö□¥ë─╜τL"∩√╬√⌠├dτCzµ#g*φ£µLúOª;╙i╒,g

¡²┌ÖGÖï□,╙YΩ`ô╬r'Ji+¥Ñ⌐│╞YOƒlp≥¿k│ô/═¥-
N□═╣╒±ñÑSΦ□Rπ6g□m▌σ∞"σng7G≈8{ê▀δ∞Ñ%√£²ö□└9@╔EN□%□;┼R╟9Φ□ñ÷ÿ□#»rö □xíá4≥┘ä
5l┬□6a
¢░åMX├&¼a□╓░ k╪D

╪ΣI╓OàPts□╙τ□ °£"å╧)Γ┬)□Y□□?,⌐>│ê□│,□7yyrÄDÆW$∩ÉTƒe─≥YF□└2δÑÄ╕┴▌
ΘεFwúD▄Mε&╓ρs▌\Äµ╣yR▀▌∞nûån╛╕ò░τzñ/t
I│╡▌FÜ¥εN┬╗▄▟ÆΘεq≈Éf»╕Å4□▄□□-
rï%┘ì╣JΩG|╫║Ä╧_¼□æ□δ`─û4X,$⌡"Iæ░╘ì$GÆIΘF"╥□^½CLz$C2}vô

╪─ô⌡ æåñi□i"Θæªæªö╙,╥£pïH
╥╥î┤$

≈□□≈□≤zd4╡îë╜A«▒æ▒ö<.2₧2▀è╜-u}6□╥gCI⌡┘PRa¼□'╪p□K│a□6□Ax$<hi-┤a┴≈□OÆOYO□¼
6ⁿé≡Wpá%sαA
-\□c.â_-
╔ß□╥<hi-¼½y0C≤`X≤`=═â⌡5□6╨<ÿ⌐y╨5j┼ »9■Å=± kÄ Ksⁿ□Ü²□jå%»□S│d□,┘¥╡╧Æ╔Ü%ô4K
ªhNL7
╞□⌐¡y0M≤`□≤áyPji□L╡□V@╥α╛□ß░□û┌V'½ô4┤:δw
|εkñ╣»ë╒╒ΩJⁿM·²□ƒ□□i-lb▌b▌Ω çyé□s┼é□wI□ε+û░f╜L═z□╬∩¥▀3> αⁿü╤{üsüXÜπB╬Ep\□
Ä╗ö░╧nûf7[│[}τJτJb|v│£k£kX_δt$Ñ╧q□═n□Ü▌┬Ü▌2a╖nΓ:╖8╖░╛╒╢ò⌠╖9╖▒εß⌠`φ3]H3]8┴
t}£>─⌠àΘl═q!°ƒ╙Å╜²¥■ñ/a║ç ╟9ε∩╬cä}ª
iª│4╙à¥g¥g╔⌡£3ä□ƒ⌡BÜ⌡▄□δ╜α╜@╜╧}!═}ÖÜ⌡,═z□τuX╧J░▐□╬□ä╟:ca┤7¥7I∩≤áÑy0│

□ZÜ□C≡α4┬qε¢ß|Ixû│É╡╧}!╕/J╪g╜║Ü⌡24δà5δ╒╙¼W_│^□═zÖÜ⌡\g°│ô\>≈ehε½»╣/3┴}┼p£Ñ
9╬
□!C¼8[à

≈ôñ≡Ç≡□╔â┬â$9ⁿ0▄ö□~4ⁿ(1Åçù$═Sf≥
╔»è⌐□'▌▌□╫ñ║██□Æª·%U3K:╠▓ù≡>w┐╘éSbîsƒSjG¼ê%╡`□GR4Åñi-IçA╥□√

R'R/RÅ4>wñG□E□□▀$┴□═(┴τÄ4═□⌐Ü;jkεHâ;^°╟1æ1Σ□□□G·±░FÜf
S╟╚╖·w^╧┌⌠╟3σ□╣ßp:  ÅEσ⌐═>□{k*≤╗ⁿ√<·^_u╦▐α▀ß╥₧≈□z□eI¥z╜0ß}□°■°÷Eúj¥╧-
□G°ΩzKε╨⌐{¬▀┬π╗¿K±<²φa}∩□9≥≡┤τ╓ⁿ╛Li9□çε⌐φz¥ê╟W▄E╧«S-(╜│W╞□M/ô;J¬□±∩{╘#ö╕├
XΓ] LK╕┤5eδuσ/:nKew□T~┼{sjçZ½Vpñ┬SêÜ.%w╔╥∩·π'a╒eε□╨v½4\p╕½¼~¼xW≤x-
ò?┴⌐2╫x5Voï⌡▌≡o|°≈ç╘;äµ%╥öXû?éw½□%±╒¬gâ╢╤u?φ√w┴╘¬2)₧╙≈âⁿ{σ?Ω╨□ZSûí□²{┤╫W▀
╡^Wu║Ω/XZÖr╒-U

°≈║╘┴rΘÄ⌠\Ω□l·Ö╟ⁿQ,j╘1d╛¬Æ≥╓╔ë╪`πc(⌡╚╤ëó╣╒τS═⌐ò.p├Q?C<÷╣ΓÉ≥╩╡¬∞╪;╨ⁿ-
¿ÖjrΓ·@║□úfΩ╪⌡■∞^v÷«æ~╙ü□╫h²É½╡ëf3□NRk╪NLñ≥⌠≤╢o┴□>╡σ∩\k&k
%≈fg3□╠Sï└(b/QK╘w:>;«"⌠□φ┐T┐Ñ□Z╛╣▄₧₧C╒┐╤─▄í╞⌐;╒╙■]~uoi∞╢─}ΩÅ╗èO□┼□µZ±Yh╛·
ésë-┐æZb□■<□âòΦ┬yÆx>[╢
≡rΘ│□ □K□%□□╝┌X╙à^èΦφ≤■≤µ□G√¿┘σ╥╞╗½ÿ▌╓√□Râ·û·V»⌡ûε'?─ⁿ╢&╤k¼UO5__∩╜bU2çEñm
à2=╞┴╓─╙%
µ(yΩ┤7~⌠╪τ╗ƒ₧Cù□^YóR|φÑτφ
|╝□┌≤G¡=+□φîµπ╠]ò-
ç≡┘Æ□╟ï□ìI─≈«<^¬≤□╝┌ï║╜Ü□Γ∩X<Ñ-╫█B═□S|□·°·8─╥╟J⌠Ö~▐╔òÜZâ╓}á>à1?J∞═V∩è ~╨'
~□└£░╪1XóD□□┬╛▀%x"■ⁿ,ÑBÖs╒GΩ≤DÖΘ■^"╛□;(U²╓Ω|îR╡ót»─wYδçJⁿ╩╕□╫î6╧╗Å°;"ë±│C
3r□uò▐√\ⁿ°y≈Ç□□
S#ÿδ-HöRµ▌□z`║Ω_â╓▐¼□⌐7╒¥ä╛bT┐⌐zh~xÄ┘ΦM··s5J²ò╣╡╨□□¿╧1ÜÜñ▐ê╫£ÿ52╒WçöÖ½ûßU
╞GεoKC
▌⌐÷╟q⌠è╣\┘╗⌠x/}+¿ⁿ,ÑτΘR╧W+▀5·╜ç▓o\£V■ìòƒk) □W┐┴┤╡ΩûΦ3¬≡■╒╧▒ö≈d²^┼åw
V┼ƒ·Ω□7O╖:KY²┴h≡╜¼σl□≤ñ╗4e■▒╖W╜«□¬□¿ß:╝□{-
δ┐)ôÿçΓzq╖·□╟<╢ztImπo▓□S□δ╒&fB=?rM7açÑÜ;~╒╒64╟╢╩□`╡δ¬üµ.ô√╗°UÑ-
>□~ƒ╪√11~□¡■e╞seï║]▌ªf¿Å┼╘{â╘²░u╗╕"Pƒ¿}∞=½z½sT
x⌠

⌡ÇΩy

u┼⌡c╙cjoéôΓ>mΘ√åc╤=₧ï□□□╧≡¡wY£╒╤╖□«╛>╛N-
■i□■e□Z│Æ1°∩yb├╛°XΩ⌐─ò.Gτé├╝½·s/┤wH┘æï╛Ü÷K╢τ≡
ú¡Å»¥Γo║¬√PG┘î╛°▒╧⌡zÑÜ¬:½°
U?─πjX╫▄coo5k▄U÷=»▐ÑTπε8÷╖++{╫²x.quê■▐╚¼w□εXT⌡Ä≥□≤-ÑE⌐≈⌡╜²-5»⌐╠╥α╕örT
ZΦÿò½z■x┤ñè:□Lç║=µ√≥╟Θ*UU╦zöφ□yñ□┐□╒│δ╕⌡L┌1┤πxî≈ƒ±yDM¼□▌│.₧3±═ÄÆ√"≤⌡så·G╠
|W"φΣΩ╫√s/5·

Dà2

√4Σ□y⌠▌z NQ▄□Ä▀╤)}□□>Æ□¼∩φ6É;┼«~╜:□
╛σÑr⌡▄±╙w╓Jε╓□¡où,□U┐╓_t╓¿i╞Ω?y□ ¡□ ╣t⌐g»ªδ⌡V°╣╩°□ k
║□≈ß┐3Q&▌╛ ~[Än9:å¼Θ¼^ΘwÑ¬¼K┐A≡╙w□⌡□ïR╤□WÜ⌐$¡□»¬ítf╠²□Kyφ-g
╝°*xV?ë·□ε≈⌐φ╟▒¼╡Æ┐ú\Θ7ÄN╥▀r≥ƒá/¿ΣhUe√▀úZ[Æ│$ñ∩≡»M─ö╘y««δÉvò┘{≥°2K┌Γ□_½B½
ⁿoe╡≤ƒ╥╘─kWú╘□5¡⌠{`ëÉ»□□≈4□ö╢ú]à÷N¿~}σ≥╫αM!╡X?ò°╢t_┐□ä▐┤Å·I▀Q|{∩0uW·▌Σ*≥l
╥w¡ⁿÖ\sü▐¢═╪ï3C°H·R╧(╡Σⁿú√╛f%·k≥■├□ √û□{Γ√z¥╕k~dvH£K├≥∩□a_█╒"ìQR□M║9±4iM|
Lk[╗ú·-¡Γ<ΓO╪╩xδ¬¢z@╜¡Fδ▀
(}°G]¬>¿f╓│□-┼∞╥±≡⌡¿XeOòπO□□ë█^⌡S£Ü.·□Ö□3½□Φë□Φú□□²ëëT□q■3π│╒uz□□□░LuQsⁿ}
⌡╣zI}φ▀1╫╟^,W÷¬Æ°j╡ΦJu°zT]Æ╪╙!,░ç

OPπ╘╜╪┴(╘┌4f^?┼╟Ω#⌡ab╓÷∩╬gH[²╠·A╒K╟┼▀G□ì«~▌┐─■»$ö╛□Tε^É┌_≥m■j╡≈U⌡

╛ ┌kë╜ · ║εQÜττδ>≡ƒ╛NV╖ ╘ù:A$^{n}$ [√ë7

□V$^n$█Ω╫·K- òocW¼emc┼ƒ; RKM₧SqÑ╖JÖ╖

Ñ┐Ép4sO□±▀▀╣Fç□╩□°₧Mu▐ì¿Äìz69A~úû2B²╧*╡Z¥├xΘ!«è╧δ
?ò╤□≈⌐Ω%÷?H<⌐0Ñ⌠□╙:■╜#£ç~╖B⌡g₧K▄üT□P7üK╒φRG┼ταÆ▀╨□□.Tτ¬Ä*±═□⌡ì·A┐-
ßÅ╪¦µñ╡ ⌡□9Q╧£°ΦTG╛╖Qy╗╞¬q¼▀)▌ƒµ√rσ▐¼┐6□Φ,□û│σt²;1¡⌠æ▓τ-Ä-
V╓▒╜zª£í■ªª°sÿzH=µç(up╢jπ∩Ç²¡□φφÑεµⁿ∩╓;!B╜4o>ªgΩE\╦▄Xⁿ¢⌠ƒΦ_□)Yt╧¬√□e□àÅWi
▌¢½NS!Oü~#└╫ ┌Ü┤5╧f?á□╖G╘;~«Zr-¡7e
9Ne┐╜d╓╓≥□>mσRl╖¥\'╫□█In'σ_σ∩r▒a□uÑ╗╤╪8U-4.3.ù°î+ìke░╤╓Φ"├î₧FOy╓Φe▄
%/□}ì┴2┬x╓□.ôî╫î╫dÜ1╨□%╙ì╤╞hÖa╝c╝+ƒ□_□
Σs│¡┘Nµ¢gÿg╨B│╜┘^ûÿYfûd¢□4;╙R≤b≤RYn▐k▐'Q≤A│ƒⁿ`

3 ö╒µxs╝¼3▀6'╔z≤c≤□┘bN5°╨Vså9S<s╢9G╢¢≤╞y▓╙n ▐£/╗╞àµ"┘c.1ù╙>sÖ╣L÷[«□æ□V¬ò&┼
V╥ò.╨╨╨░2
▒ÜY═ìá╒╥ji8V□δ$#d╡│N7Æ¡3¡3ìê╒▐:╫H▒~g¥oñZ┐╖~oñY□¼

F□½ô╒┘H╖║Z7□□■wσî ·ü>ü'ì╠└α└G╞iüO□3ìNü∩□
î[éß`╪╕-Φ□]π÷`j░╢╤#ÿ-l`⌠

. «0ε

■□\k■\\o

n□n2□□≤é¢ìçé[é█îGé;é;î'é╗âE╞ô┴âv╨□b;vè1▄N╡Sì1vÜ]╫x├«o76╞█═∞▀□□φ▀┌┐5f┌g┘□□
ƒ█W┌□iàv□√ c╢²╡²îi╪Cφíf└~▐~┴

┌/█/¢Ä=▄-eå∞7∞qfè²û²ûÖfO┤°ÿu∞Åφ□fC√s√+│à²╡=╫lc╧│s╠ô∞¿□5╧▒W█?Üφφ\{ïy₧╝▌■?╩
╬□:¬Ωl≈√£Ö│τ╠d╧$ä□!≤'3ô╔ⁿƒ╔ⁿƒIH0`êêê□□)EJ)-
≥Q>J□)M□□□#"bèêêHæRDDè□□)"Ñ╚E.σCDJ□æRñöÅRèHæΓ}÷;□Y]δ«u»Y√Öφ{÷9gf2y≈∩üòç½j
o□3⌐Ω]&ô╔¡

2·M□u╝⌐▐╘ñ■D■~£:CWuU}Dτ║I¥⌐ùΦ%Ω,╜T/S-
╙+⌠□⌡q╜R∩í╬╤¥║Sm╫=║W}R≈δ~⌡)=¿ç╒yz\»Sƒ╤ôzR}V╧Φ□╡C»╫□╘ƒΘìzô·£▐¼≈Vƒ╫√Θ²╒□⌠□·
□u⌐->P┐[}I┐G┐W}Y ╢■muà>V□@}E í■#⌡□·□}è║Zo╙█╘W⌡ç⌡ç╒5·L}û·Ü■ñ■ñ║Nƒ°╧S▀╨ƒ╤□¿δ
⌡

╝C▌ á/╥_T▀╘□⌐»V▀╓_╙_S▀╤▀╘╗⌐[⌡φ·çΩn²É■▒zT â■G⌡ÿ■ë■╡·Ö■□²ΩY²
²
⌡K²▓~Y╜¼□mV╘□Üìfúz╒l2[╘□Öà╡╗A1≈0╗

V║█∞6t5{═ACà9bÄ□∞µö9e¿6g═Yâ█£7≈4x╟Mµ>□┐╢┼▄bêÜ[═²

1s□≤¥åä·.≤▌åö·>≤0C╓<╤<┘É╖╕->Cúσ-╥pCo╥(╥(├□û',k
²-█-╖
ô,YÄ□~b9f·|ßQ╥?K¬

Oû

)·╛ß5·[{åwJ6òl3ⁿVÿD⌐a»êê╛åÅ─w─8├y±□1╔pML□SîF1UL3ÜD¢h3Z─t±╕▒D┤ï·╞[─□▒└Φ□□b
í╤)□ëσF╖X!V□#bÑx═□□oêì╞îxK╝cl□┐□┐1▌*▐□∩□¢┼{b╖▒╖╪+

□∩□□┼Aπ qH□6~K□□ƒ□çê╧─ πpδ#╓Gî#¼ÅZ7▐om╖>m□m}╓·3π8δs╓E╞-
X_░╛`ⁿ/¢j╙ì□m┬f3>d+╖U□°┘∞6°±°╢/m_□gö▓R┼°□Sò□Φz68╛RV╞□╓□_□VÄ}╪╚*▒wk╪╒kQ≈π╦
─□╪□u□Eù4ú-
÷d□²P&q≈óîr┘1m╘1K╤1çΓ¼√≡╒□}≤~\q$√>kfc╨C{úçN□9<ê»>l□¢╞na?┴W7╓╞fα╬Åá├Vó├□╓C
▒*6VE┐!lW╩╨scΦ╣□TéJÉ%öÉ□F=óD0Åó□≈á^£D/╛□:□□╣/:≥□╘∩G_NQ_NQ_Nú/OG²aσ
ûQ┌òv\≤Itj;:⌡3,°,P₧cye□║vÆ║vÆ║vÆ║v□]√U╠╫áw'╨╖□ç²`ù▓ï⌡T▐W>`ì╨^t≤&Ωµ*
║y□ÜEOτ╘╚╦¿°½╘╚╦¿°WPO┐ìzz□⌡⌠□⌡t□z·½¼Z]ú«aN⌡5⌡uµQ╫í╦{⌐╦{⌐╦╗╤σ╖B□â^∩ó^∩ú^∩D
» ▀╨}Φ°ntⁿ²╨ F▀wQ▀wQ▀»A▀□¼╓`E≈≈S≈□R≈□á√W▓░íçí□ï□¬

U¼Eε□ÿc'`!∞□□h╨□┬Y╪□XTε□8½┴╨□φiΦëúMå&h/C/¼┴▐□┼▐Çèⁿ]δ█Θw¡√╤∩W▀N┐_▐Å~°║□√─#
¼ùqª±
ª`╖X└Jì╧□□▒zπ≤╞┼¼½±□π2╓`|┘°s╓▌╕┬°:δa\gⁿ5½┬Ä≥□K□7□╖▒î▄WXú▄WÿÉ√□┤L+c╝
╡.Z□ûö╖
Kaw9╚

┌G┌G╟╒¡□╥

▒Rφcφcf╘

k□`□v¥ú¿|ó}é╧1φ□3ifjf2];«□g╖hfif▒□╣'1½▄ô░≥┤vÜu╤■ó²àòcg·+S┤│┌ αÄτ┤┐▒«┌yφ<δ
.≈*▄±
φ
V⌐]╥.▒&φKφK<╖╦┌e<f□j ─ⁿèv□≤»┤»X/φ_┌┐pσk\e]╣ü□Y/«qì)╪ßL

¢□╫Öò¢╣àò≥□^┬

\p┴*╡ò[Y□╖q□╓`□ö ε.∩ès+°-8╖Æ≈└·*ngσ▄┴¥╕▓ï╖p«ç{á^ε┼□jx
╓√╕□δky□δC<─║≤0□ú-ß□fΣQ-e6-πq\┐Ä╫ß▄□OαjI₧─Ü□Oß▄4O3!w\▄+╧≤¿□x□V÷Σ=qàF▐╟4▐¢
≈┼╨V▐╚Lⁿv~;₧≤▌ⁿ[x]â·╜╕■²|□ε■=>□w·>-
ïδ<└□└Ü·□■C╓¢OΓSp╟ç°T╓ç ÿú{≡ƒ≡6╓ì ö □╧v:ƒü╫≥□ƒëδ<╩-
┼□f±Y╕┬cⁿ1V┬g≤┘╕╥πⁿq¼Ö├τα. □fù□└□ ÇgYåw≡

ûû□└zÇ□₧╟╤┼|1½Γ/p⌠□■"□æ5≥Ñ|)▐φσ|9⌠τ|□K±W°+X□V└□^π»A╫r|J·:╛

τ╛┴╫│╛ $^n$W$^n$W╕≥□■&ÄnΓ¢pε [$^n$─╘7≤─X·

▀èò∩≥φ8 ·-▀┴r î]¿┐╧▀gqp╞ ┬·=|□*≡□░r/ =Vετ√±|■¢└Ü□·çxå□·Gx╬ç°!□π≤ÅY₧-
µçq.□□g□π╟pσ0·°8δs■9«vÜƒ┴·┐≥┐b²▀·□Xsë_┬╗±% □╧φ2┐╨zHÄaipî□s¢⌐
╥ÿ╨M]Ö▌TaΩ╫rªJôâσMNô¢%A9□╓h□ÜB∞

S╪□a=MQS□òÿ⌐Ä5Ö□ª□«É4%▒2eJaM┌ö╞╤î
▌□lT╧▓ª□S□ε╒╙╘□δ□Mì8┌dj┬╜dªÇ"ÖëÑ$3A┴LP0□□╟□□3A┴LP0□□╟─¬$31╗d&(ÿë┼$3
a

fbìÆÖX□0S3ïδ╝⌡▐8
Σä□╚  k@NPÉ□╥IrbyÉ□£Ç■Ç■□k□?²ÉòΩô⌠a
(□τéóP□EaσL}&«≤¿■(µ│⌠Y¿â¿≡|@TX î■

╥Φ
│□8
\┼╥α¬E¿<»πS°/╓_─ⁿù·/q»╒·jvç$-T@Z╠"I
□╥éé┤á -Φ_⌠┐│[⌡
·□▄σ·?p□P□KHΩ┬ⁿk²k·»úÿ□δkV╠□δ!
î┘A`&¿n╓Y╓î X┬l1[0□f□┤╘î²╫\f.c9s□s9*]=]Yú╣┬\┴╥µ[╠╖░&s7sw╘{ÿ{░î╣╨\┼b
f╗┘Ä╣├∞└]£f'Ä║╠.T└vÿâφ≡L└vP░□□l□□█A┴vP░□□l□□█A┴vP░□□l╟,Æφ╪¡`╗{XÖeêe□πû{-
≈b>╘2□≤√,≈a>╠2£UH≥Cσ ╥JªZ~aYï9°□s≡-
╓Ç ░µƒ%□SK╘Æ*v¢ñ@V(f7H□d¬ñ@((□·□±□µ□#─□µ□≈ï√Y□1Rîd╒Γ╗Γ╗¼Fî□úÿW|O|Å□─h±-
ÿÅ□c▒■□±□╓î□π░µ□Γ□ÿO□ ┼|bóÿê5?□ô░f▓ÿîú□è)╠□▓ⁿ1Ω╙─4╘┴ù╨Θb:⌠a1â9─#b&≤êG┼,¼|
L<åò│┼╥╕c╗x□òyb>«

□┼]:D□⌠gb!╓,□╧π9/□ïq¥□─□╠_□/b²R▒□≤ù─K╕µ2▒

G_□/│ÇX.û│É$W□□╣«d□±
±
╓"VëW1 #╓`=kΓ5□}C╝□]/~┼óbâ╪Çúoèì8·û╪╟┬Γm▒□òw─;¿ÇwíαΦ{b□½□┐□;▒µwb□≤ï≈┼√X╢
[∞╞]÷è▀ú▓_□└5A├┐■!q□·▒8î5G─q⌠¿8èδ|"Äa■⌐°öe@╓ƒßj'─
□É¼╟\`σY╟a}╞:¢y¡Å[±.ü¢█Y╘·ñ□∩òu₧u-½║>m}□òg¡□,b²Ö⌡g¼E≥4*αi□ò<=*$O3U≥
4□<
□O│□╓╙,□▓k&₧n%₧Vëñï▄|¥ÿ%-
█êÅm∞█°▓□□≈#2εOd\Nd<Ç╘╕□æqw"πJ"π-7σ≈hö▀úS~ÅF·=□σ≈X(┐Gúⁿ-ì≥{¼ö▀úQ~ÅF·=□σ≈ö
R~ÅF·=Ñö▀úQ~╧□ö▀s'σ≈tÑⁿ₧╗(┐g
σ≈▄M·=â(┐°□ñ^□n╢*Vb⌠-,½T)U`hIΩ□É·▌¼üXⁿ-σ^σ█¿K□∩⌐îU╞é░-
R-éNU┌└═╙AΣy□y;k□ï?ë·S╩SX/ë<□"□₧5â┼ù▓▐á≡ì╨_+┐f}öM╩╕8*)ⁿ>ó≡█ê┬[ê┬√é┬□╟@□n╕
ë┐
α∩█ê┐∩□□▀I□.□åîö0╘à□å║P┬╨-ö0╘à□²[─Φ⌡ΩôΩ\╓K]¼╛└åtÆ║Σ≥ê·å·□
⌐¢┴σ5DΣ╡DΣ□⌡□⌡□≡╖dqÅz@=Ç·Gαo□Ñ□9╒?¿ƒÇ╚?U?à╩□ú(Ñ║à╒ôΩƒQ·\²□*▐▌\ölΣS G=ç╣╟7
≥½□W/`.SÄéΩWΩU╟e╓Q╡zM²Ü╣(±╚kP

*µ2≈╚○╨

□µ2²╙KΘG>Cëí□òR╨□£╕?E▄ƒ!εl░□□¿K·Å□j@ u□?Φ?N⌠ƒ0ä
a╟úå(4iH│4£@-≤éí└båz°ü8·üñí□~
n╕╒p+«/²@££╘╝Σ□åÆ□╕ù£└P≥□¡á E╟□ε_╞╚ë°+ë°φDⁿ□π&□□O□ N╓dⁿ¥q/δC▄▀rS&ôFÖLÑö╓╘
ò2Ö□æ□ΦONá7σ3▌I~á□~αC╞╓□ÿ┤?└□p≥□&≥□6ó□□╤□ÑvR;
╨?Ñ}ÄèΣ~N─▀¥ê┐?□□9□□%□□□φóv□*Ö╛òÿ▌DL_NL▀JL»r

ª7□═¢êµ{□╡╖□»¢ê╘╤ë╘{□¥╖□ù¢ê╤+ë╤[┴Γ≡╜<

"τ─Γσ─Γ¡¥□₧ß□¼╧±□╓K□o%□/2╖ë8█Dl▌Å╪║?▒u9▒⌡□bδn─╓▌ë¡+ë¡{□=≈α≤°<0σ╙ⁿi╨ññτ□"µ
F╛ê/B]□sûê╣7_╞ùü#%+τ°□░r#▒▓¥X╣ë»Γk└±»üÆφD╓≈□7±ì|#╬Æö£#J╛□ö╝□τ╛
V╢□+□êò¢°o·N\ßwⁿwX/Y9Göl'J.□%7□%╖≡□áΣFóΣ▐D╓9óΣ&óΣfóΣ╛D╓Y■ □G%-
□╓8╤╧≥≤¿H>.□7□▀├»±k TI╞ìD╞M πεÿK&n&&εm≥ÿjY"π□"π√êîo#

εM□|qp
q░▌ö7σíÆÇ√□□╖ÿn5▌èk╨D▒R╨□╙(K¼öR─J)ELú□1
Ñê
ñ□1ìR─4╙`╙`▄]fëiö%VJ)bwRèXWJ□□D)bUö"VE)b□Ñêiö"ªQèX)Ñêu╝)E¼öR─,ö"VJ)bUö"ªQ
èX)Ñêi7Ñêiö"VJ)b□ÑêuÑ□▒*J□╙(E¼öR─¬nJ□╙(E¼öR─□QèÿF·a┌M·a□σçY)?¼ö≥├4╨□□tS~ÿ
F·aÑöªQ~X)σçiöªQ~X)σçiöv□σç▌I·a])?∞.╨□□H·awS~╪ ╨□½óⁿ0ì≥├εñⁿ░üö-
6Φªⁿ0ì≥├¬(?LâçΘ╨□αXjYo≥'}⌠Ç-Ç7□ΩA░~DÅ░é-╒c≡□q=ÄzBOt·û£₧╥╙¼/╣ù£₧╙
PΘaZ⌠₧zO\Gzÿ>z½~;┤ƒ~'«6@┐
k□Ω□YV┐□NªIñ□åC╕O┐□GÑƒi╓┐½□□╧g┤>□g□ô□Ñ├iü├□Å{Içc╙□ñO╞u-╘─Y□Θ□▒█⌠-
δ?Fσa²□╝□Θs□╚█╪)╣1G

°Qƒ»╧çJƒ╙ù|Nú■£Ä.A>'G

°I□Y□□òW⌠WpwΘvZ╙φ▄°┐¬»┴Y╥≤4Θ»δ»c═□·zΦ¢p>%·1²O╨?├≤öÉτ╣¥<O-
²ó~□WûP₧°A J □»NzP₧□≥<≈ÉτΘMP₧°æ▄NÄ▄N□╣¥£┘□ç╙□ç╙à5ô├i!çs□9£╛p8▌αé║¢+▒▓□□Nü╝ì¥
ⁿL°Ö□ε□åƒ)üƒ╓@sµ□h□<L
yÿ□xÿ╗í╥╜öÉ{)!≈r;▄╤ÉN╟"╜╨0°ÉßΣXFXFá≥}╥≈Y/╥x╥xΦ□╥□ΦD╥DΦ$╥$Φ□╥□¿╠óδBY
t](ïε□╧ó╗à▓Φ║P□]□r>□≥6▀*▒ùxY}I Æo▒^%cJ┌╪□J¬3Æ█1┬ßDα"ñçëÉç
ë Çç±ê □πAΩ╥╥x╙▒DαX~ê·$±#8ç⌐b**╥½╘êƒèƒó≥░x□.E·ôZ≥'□≥'!°ô╣¿<□ù□"ù□□╧
êg░^·ôêxN,┬╤τßO□≡'/αj╥ƒ╘Æ?):ô□r&q±s±sΦ+Γ□¿t&□r&â┼½p&I8ô╡¿┐.╓▒□9ô$9ô49ô

£╔¢¿l□┐ f1▒Il┬╨╖┼█¿K□R'╢┬ƒ─┼6▒
Gw┬Ö$╚ôd╚ô

□{─□8║W∞C]:ô┤°P|êò╥ôd──
─□╘ □OÆå'·□W;□gΓ"gÆ□║┼q▄W·ô□·ô:±'□╞út└(σæå┼□q□□Ö□Φ□τ─y╟e^áƒ≥□╝ö□□Ñ╝@/σ□VS
-⌐KⁿKⁿ
*│□úΓk□□ñ□A└□□H9é╒öMΩó4A'eô║(S╨OÖéQ╨&
[m╓R╘e╛á▀┌╒┌□□Ö2□ñö┴jkÑ╡□Ge╓`ö▓□²ö5□ñ¼Aƒ╒k⌡Γ¿L□⌠SΓáù□□}╓±╓±╟CN¼□N∞Qrb°<Xƒ
░>□ç╓

≈UKε+M╛k0|╫sÿ/▓.fr_iδ□δ□╠eríƒÆ
¥ö\□ÑΣ┬ %□·)╣╨□o$╪□0■l╨û╧║+τò┐3ª|í\b¬rE·è□ò»UàqUS93├∞
Vóû⌐]ÿU╜Eφ╞J╒* ╒

Γ≈¬5¼½□TC≡□/⌐/▒εå~å;└v¡┌φ¼J¢¼=╚□┌{┌{╠e├□½╢╣mw1╖mÉm□□h√«φq÷□█│╢w┘,█n█Y÷+█9
█%8-ò┴cP.è
n╦╟║░!¼□Np4□╒□÷□□┴₧f≤┘l8╖□┘□╕│╧α╤■ñX╪╟èÇ╖·Z)UnQ□E■φç.□ò╥]□«<á8ö Tµ(aÑ]Y¿⌠
S□+/┴g²Z·╜≥□├δå╫ò⌐`Ωçö□g□g)?1╡□ƒRª□ƒ5>½╞□_┐á<□╛~EÖ
▓^»<  ▓~[Ög|╫°«▓□d²╛≥,²MφBÉ⌡ç╧≤ δπ╧
╞S├┐(Kì□3■MY

╛■R·╣ΣkeÑ╓Uδ¬ⁿ□4}MY
╢│)□yÇ□öï`╒≥□╪│A·J■┘»≥5┐ì╖¿F▐╚∩□■▐═G¿6■=>Fu≡▒|▓Ω□{╬Pcⁿ I>_═≤□|⌐┌─_µ½╘■≥╧T
╒┴|□ @╜ç∩π√╘üΘ

½ô·Q~T²)?╬Å½╙┴kg╘ç%⌐⌐Å≥□≡ïΩ□É┌5╡□êmUƒ□ù▌ó╛lΩn¬Q_□ïe╒⌡ª▐ª Ωv╙âª

⌡¼Θ9╙s□□╨Xj░é□╓□║╧─eCw╙[ª═□çiïΘ=âKÆé┴□F8l╙ÿÄÿN□□αé/
}▒ço0

╤ a÷□>┃}e√╩X╞>e╠~□π,╞□î╦□╫□s□o<*

─╥0║a80╝□Aî8F□ú□ú□ú□c□╞`îa□#1╞`î╟ÿ─T╟T□╠1¥åΩÿà╤Ä·|îà□K0ûc¼┬Xï▒□c3╞6î¥¥╧a╧
 σq□τ╡Σⁿ Pτ9G1N╨1µ8ìq«≤·εΘ|─kt\─┐┬ÿô□δ¥Å¬ô╙P£□ú□≤╨□╡Γpa°ès▄Wuå;δë╫æδ□ì□}0·
a

─□╥╣v8¡g╫Q□cï∩ôs┬ì≈╜┐v2¡c╫i□30fc╟->wτé╫√-
*╛VτRî□□½;Å»δ<╛▒slAm;╞.╜₧╜□□n╜□z═╬├□╟0Nb£┴8Åq π*âY╟╨;-
m▀<^_∩¬└¿*>╨⌡≥<· 7Ä╖1ⁿ□Qî□F□ú╫7Å≥{µj┴Φ  ⁿ¿║□▌⌠╜┬ks
┼□Qⁿ~ □=n√╖G|╛]úï≈í╧Rg¥ε{≤□ç1±¢G·╜mδⁿ╜¥└≤¢éz□╞╠╩╧ƒ╜╬£o-]≤0:î]∞Uv╖▌oÅ┌Sñ□╥
^╨□{□Φ √PΦ□√hΦ8√DΦ□{□T₧5╙>╟>╧▐a_l_f_i_c_o▀d▀j▀A║√╞|ƒ² T-=b?n?e?k┐`┐

─╬ïz═atX□eÄnñ

¿ùµ^Ü□□qh╞╤□mv┤B□8□;,t╓0ΦHT╞8╞;&9ª:ªCg9┌í≤□
í▓┘─▒▄▒╨▒╓▒┴▒□║═▒╙▒╟▒▀q╙q╘q┬qÜ⌠□ΘEΦ□'~j¥▄)áσ╬J¿╥Θâå¥
□┼Y╬£│╤┘╟┘╧9╨9─9▄9╩9□:┴9┘9ìtås╢s«süsæs)tàs5TV╓u╓ï║╤╡┼╢▌╣╤╗╫y└y╪y∞åΓ
╙□ò⌡3¥z▐y╓y╒Ñ║tR□┤éµU.7╘∩èBS«□┤ù½σå÷w
r
uìpìvìsMtMq╡╣f║µ╕┌Σ╒\≤:+□«┼7TVû╣V║╓╕╓╗6╣╢Æε╕1ù⌡▌«}«â«#«π«SP9?
╜Ç·e╫╡jc╡Ñ║î┤█ì╣ú┌

V╟íÖΩ□hsu+t@⌡`Ü□½6╥╣#½╟TÅ»₧T=╡zz⌡¼Ω÷Ω·7t!tI⌡≥ΩUÿ»¡▐P╝╣z[⌡N·□¬≈Éε┐>»>T}□zó
·t⌡╣Ωï╒W«½¢╣9T╕╥▌ònù█G□&M@sεFh-
w?Φ@≈□Φp≈(ΦX≈□¿<k▓{Ü{å{╣{«{ü{æ{⌐{à{╡{¥{#tï{;⌐£∩r∩à╧ú□▄ç▌╟£╙▄'▌gáτ▌╠▄ù:⌡¬G
⌡Φ-¢°é┤□Ωª╣¢µ~O□Ü≥□á╜<-╨■₧A-¥╬□□□ü╨h╧8╧D╧□O□tªg

t₧°□*δï=╥<+=k<δ=¢á[=;<╖=√<□=G<╟=°H╧Æ^Ç^÷\≤□±□╜□hÖ╖□╘ß⌡Bâ▐8ÄΓ,○╞█αm÷╢z□x□{
çyGz╟@╟{'y°ÆN≈╬≥╢{τ{□zù@ù{WAeemg╜¿□╝¢╜█╜;╜{£²<g╜√»½≈É≈(□u∩ëN=φ=τ╜Φ╜R├H·⌡y
ì¿)çV╓╕á╛Ü04Qôâ6╓⌠ü÷½□X3ñfx═({G╪Ü 5ôkª╒╠¿Ö−
»V3╖│▓áf□t)⌐¼¼¿Y]│«fc═ûÜφñ╖n╠e}○═üÜ├5╟jN╓£ü╨··ÜK5W}¬O≈┘|□╛*O¢Tƒ√╞▄∩ïBS╛□┤
ù»□┌▀7□:╘7□:┌7

*╧¥Φ¢Γk≤═⌠═±═≤u°□√û²¢«⌠¡±ux□√╓√6·╢·v°v√÷·

Æ−╣1?ε;□=δ╗α╗∞╗Vk╝«╡û┌2h╖ZG¡╖6X□'═@□h▐\█□□P;□:¼v$tLφxΦñ┌⌐Py╓⌠┌Y╡φ╡≤k□╓.⌐]
^╗¬vmφå┌═╡█á;I≈Éε»=□òGÅ╓₧¿=]{

zæT╬»°Öƒ√à┐▄_Θw·}ñß□¢'ⁿ9hú┐□┤ƒ□
tê□8T₧5╧?╓?┴?┘?═?├?█?╫┐□║╚╕□║┬┐□║╬┐□║┼┐□w┘E║╫□└□╪□╟□╥□╞□▌□ë⌠¬╘Ç□=□╨²g□╢@□
┤*αå·□QY≈ƒ

ñ□à@/TZ□²□â□C□#□ú□π□□íS□m╨Öü9╛MR□≤□□ü┼üeüòü5X┐>░ ║2░òt
«S¼□uG`w`_α`αêƒ□Ä□NA╧Æ^□\åó-╕□4□-v□τ╝,╪-Φ□zâA╥°
=□□á=┴VΦÇα`Φ░αHΦÿαxΦñα╘α⌠α¼`{p~α`papë□Kpyp µ½éKékâ□ⁿgé¢â█á;IQ
ñé{é√âçéGâ'■Me²t≡\≡"«|%─B<$Bσí╩É+Σâ╧y□Ü└<□jö»+╘ç┤_h`q-□□□

□□□
¥□Ü

¥□Ü□¥□Ü
]□Z□┼╢ükíÑí□í╒íuíìí–íφí] ª{C□áçC╟B'CgBτCùBWk3R├Ωu
δa□┤"\□vç²ß(4EZ□≈é╢ä√ç□I& □
Å□KV□□ä╟à'å°8,ß╢≡LΦ£≡╝Γ

−εÉ√`xqxYxÑ≤jxMx=vL∞DßMß¡ß□rW□∩╞

à╜&╜/|0|─u$|<î]O■╜ä╧å/ä/╫zστ6|-bîXⁿòæ▓H7¿#Γ-~╞"A·²ì─#ÖêⁿnûEÜâ

·>DZ#□"âσ{□□□ÑW□□□□□□Ö□‖$w£╚╘╚⌠╚,∞>Φⁿæ÷╚ⁿ╚BÅ-Y□Y

]□Y[∞╧æ
▓╥E6G╢Evó[₧Ä∞±^ö}&▓?r(rT÷£╚ (:IΣtΣ\ΣóowΣJö·v╥O~ □σQ□-
ÅVF]Q_4□MDs╤╞hƒh┐Φ└Φ□√ªΦ≡Φ(√┘Φ╪ΦäΦd¼Öå53ó│úsú
óïóKú+ó½úδó□ú[ó█ú╗ó{□ô¬╟D□TOè-Ä-ï₧î₧ë₧Å^¬^□╜Z╜9ª╞⌠ÿ═╢+V□½r₧ÅüUb■X┤v~,□+─z
┼Zb²câè╜□□□□□□□□□□□[JóêMë╡┼f╞µ─µ┼:Σw!╢°·╬-[□[I║□║>:-
╢)┤5╢#╢;╢/v0v$v<v*v6v!v9v-nî[Γe±nqG▄[dZ╟ ⁿx□-rTæRΓ±x╞▐QΣ╞xC╝□┌□-
□è├g#>╪òè□ï□ÄÅîÅëÅÅOèOìOÅ╧è╖╟τ╟□╨òÄσ±%Ä╡±σ±U±╡Er│Åïo░√π¢π█p/b╘°╬°-ç┼Θè∩w□
ìè┼▌{┼O8»╞O╟╧A/╞»┐┌ΩX□w╡U7╫ëjc]y]%╘Uτï«sl¿
;╓╓%Ωruìu}Ω·┼6╓wán`▌É║ß┼╧╢wz▌¿║▒╬½u□Ω&╗Ä╘M½¢Q7╗nn▌é║EE┬¼[Z╗ónu▌║║ìu[Σ╧E▌÷
║]u{AΘ`⌡║□E¡;\w¼Hαu'o╥3ñτσ]Ω.æ^M¿ ▐aI╪□□ÄIë¬ä□□
óN°□╤D¬s^ φ%□╛□-
¥∩$x8╤ƒtÉ|Vëíë□ë╤ëí┼9Θ┐───□τ÷D[b&x□T£ÿôÿùΦ(2pb±M║╠╒□▐kK¼L¼ü«ù*⌐5▒⌐¿ë¡ë□ER
M∞N∞K□t¡O□I□çóÄ╩⌐─┘"╡å≈}úë
≥°>qÖ⌠ZQô╞ñ□,□"Mû%╗%□ Opi╥¢

&π╒¢ôÖdC⌡µ─┌d3>ô□ô¡αL|_Æ□èÜ□£□û□Y{%9□;□:│ƒ'╟''a▀╝Æ£è·⌠Σ¼d╖□Kr╛▄□Æ
ôKÆ╥ô½□╥Ækô□Æ¢ô█Æ;ô{Æ√ôçÆGô'Æ°ôτè╜╜╪╜C"y1y%┼R□▌°LJñ╧ï¥0UÖrÑ|┌p*æ╧Ñ□□²S}R²
R□SCè

α?ƒ□Ä]ÇvÖ╘¿P┐δ{tjljBjrjZjFjvèv█╘é╘"▀&┘╡RKâ□R+RKS½²╛╘║└ö╘╞└Φ╘û╘÷T«╕/□Ä°v·y
joΩÇdë╘ßZoΩXΩñ▄╙Sgpσ≤)∞┼⌐½i∞┬┴1i]ε_i[╨æ«HW·╧ñ▌i┐ LhA:z}°H°╥àt»tK║┐ƒKûê»M□
J□ìmÆ».="=:=«╪i²ú╥□╙Sp¥╢⌠╟╨%╡τªτñτ□Äñ;Σ>ò^£^û^Ö^ô^ƒ▐ö▐Ü▐æ▐¥▐'▀╗⌠A║╬æ⌠±⌠⌐⌠
┘⌠à⌠e╫2┘├╚╫:i□□iφ╘δTs:cöZ¼d,ñe≥9d║æ:2▐L╨?9□╧dⁿ│3
D# ôHkª9╙Z£□«I┼Y╪
2□d╫═

+rEfd°ΓUDéÖ1Ö±æ`q.□+'eª□÷eªgfü(└□Ö÷╟ⁿ╟┬"EDZ┐╤LYαxfIpIfy□╗□fmfCq╟╟ε□═l╬l╥∞
,ε≥Ö=Ö²ÖC┴CÖú□∞√▓Ä╩Θ╠╣Γ.ƒ╡xô^æ√TûI═ö╤£gE╢□{7v≡le╓ò⌡a°╞>₧
g□┘\Φd╢1█□┌/;□╗│?;□{1▐≤∞p╥Q┼w&;6;!TÖ¥£¥□┌Æ¥æ¥□║ö¥¢]Pδ═.╨.═«╚«╬«╤n╟n╓n╧ε╨ε
═-Ç-╫-≤╫╧₧╟₧q»╫₧╧^rN╤^═⌐9═╗%g╤ε╨UΣ¬rε£?□u»╬Ñrà\»\K«□nPnhnägów|n┤wjn\nbnèg
_«═s$737'7/╫æ[£[û[Ö[ô[∩=¥█Σ╜ÿ█Ü█æ█¥█ù;ÿ;Æ;₧;ò;█Θ

Σ.╘□╦]╬]╦□≤ûÜ½·▓|╥╝#∩═□≤q≈%▀╓|µ:çτ□≥═·╓ⁿÇⁿ`╟çσGµ╟Σ╟τ'σ°µ°τgσ█≤≤≤
≤K≥╦≤½≥k≤□≥¢≤█≥;≤{≥√ï

╘s<□□₧ï£N╤SΣÅµOΣO□]^■£╧
╜ÿ┐□╧à╜╛└j|□^`╛k□Q(/T□\□_!\HΣ█=SΣJ╧▓B╬│ª╨XΦSΦ⌠YnWaαu?[⌠ÿà!Σ+Å├╗└±□å_┐{Mó0
□J^⌐0╢□▀╘Θq·{µ°*□ô≤═u[<δ
╙p²□à┘à╣à□àE5│σ;PXZXQX]d□╧╨┬║┬F∩┼┬û┬v▀ε┬«┬▌┬ü┬ß┬▒┬╔ó─
,£)£/*\¡Wδu╔9⌡╢·è·*xj8δz7⌐┐>ZƒékåâN

ÆZ_
7=â̄µ╜Σ]Ω[èZ▀┐-ε╕~¿»┼3▒~D²hx^°▀·q⌡□δ°t╬█HgJ^¬ƒ╙·N┬╜╓╧δT<½·Ä·┼⌡╦Ω;ès╥ò⌡kΩ╫{
╖╒○¬▀□≈□□[┐π ░w÷q6_╫■ >₧Ö□2□g╬ôßÄëg"

□╬9s₧"₧Γ¬¿ñ**ó2WDTED╘□□QQQúÆ¬x¬¬L□□æ)bDT□Q□APQ□W\d"¿ê╩╕{╜≈□¥D√J■ ²^c»·X▀╡
╫^{φ╜╫^{ƒπ½╙÷N╗⌠ë╡╙▐□⌠╨═3;□╜yT°□¥╬(zN¿£1[╢╔┤╙ÑNU·\□v┬╡┬Y7»
√┬·7»□╛Γ□å¢Θ3f╕u
┌^▓╕p□Ü╨4▄%▄S¥□╒·1▄'▄/<P¥□╒)2<$<,<▓QyxLx╜óô┬S□m□╧□╧╛╜ïîKx.tQ├□ßÑ¥ååùçW▀|$
╝6╜▒╤¼≡û≡

%╣;╝┐ □>□>□>)g□╜I∞j<╖ë'\□╛□╛□1"₧Hφ╞╟"9¡ùG□æ□▓wD□EZD┌□U╕C$□IE║Ez)┌≈□φ□□)ë

Åîèîìiì LP╡&δ3]drdZdfΣà╚╝╙ΓHYdedMd}dSdk$ ±Sh╙┐╩╬╙₧╢k%ZF

@Å4φ□Qτ╖╙⌐╙┘╙┼╙ò¿□═l‖$Z╖iQ╘□
E
óMó¡óE╨Ä□'ú1}╢□□φ□φ□φ□╜Wƒ│ó□óâúCú#óú;lÅÄïNîNëNÅ╬è╬ë.ê.QtYtU┤\bft□tst{tWt»
óçÜîè-ì₧╕}H⌠L⌠▄φC⌠₧□╜□¡*vèk□g□√è≤ï
ï¢□╖.n_□.N□w╣u╜D╤╢ùï{□≈)(+εW<░xH±░Γæ┼cè╟□O*₧┌|e±îµδïg□╧m^V╝¿xΘ═k⌠

%┤xy
»┌
□.^▌εÉ╬▄n]Y╜╢xc±ûΓ□┼╗□/-▐▁|╕°X±╔Γ╨Γ
┼ù├'#²cFñ$µë┌Ä╠îσ─□▒□▒F▒□▒6▒

▒h,□δ□δU8#╓7╥
╓┐ª╢╪áXIlxlTlllBlrlZlf∞à╪╜╪ΓXYlelMl}lSlklglO∞@∞H∞x∞T∞l∞b∞J▄èg▐╢5^7εU4□/ê⌡
O╙&±Vì
πE±Ä±X╜s╜Gth╜wⁿ▐°Ç°α°╨°ê°Φ°╕°─°ö°⌠°¼°£°é°Æ°▓°¬x╣îo|C|s≤S±φ±]±╜±C▒n±úà3Γ'Γ
gΓτ⌠╪┼/┼½□NóV"½╒╩ä/æƒ(L4K┤V┤}"£H$║$z&·$·%□□5jûò□╥,?1,1R╤lë±ëIë⌐ë□ë┘ë╣ëEè.
ì₧H,O¼N¼m;0▒1▒Ñ■╪─Ä─ε─■─ß─▒─╓DeΓB├╟─σñæ⌠$k'sÆüdâdúdïd¢f╗o]ƒ∞ÉîF╓$S╓n╓^ë╨d
▀åÖ╓■╓A╓□▌JrxrTrlrBr≥-σ╓i╓Ö╓□□ï
j\Ö£ù\£,k║9╣▓Θ«ΣÜΣ·ΣªΣ╓Σ╬Σ₧ΣüΣæΣ±Σ⌐xτΣ±f;Æg¢φO^L^IY-
╜⌐╟□□⌐║)o*ö*H5I╡J□Ñ:ªb⌐╬⌐-⌐▐æß⌐{█«□Ü□áO²⌐┴⌐í⌐□⌐╤⌐q⌐ëÆ╜ñªHûÆÜ.╖(⌐Yz┼IÄQ□e·
ªΓk½C4ⁿ≤f 5'╡ ╡$2V÷≈╘29â°V╓lLòδ█!ë□M½R□"
ö~2▒╘µ╘÷&â"⌐╘«&â╥╖7▄½ñ÷6^₧:T╜%u4uBƒ·SgRτRùd¼[63╟¬√£;□φ∩>¬Φìαzα╢α╢éì╓÷□□╖s
║)┌▌□═╨╨<²5x║óEε½αn`¡í─╕7uoU┤5ⁿ÷╬□⌠H▌"Z□α┤□Ω▐'ÆW_V-□ε8Eφ½∩└Yí¿╫I u-
St5╡□è%_ü┐¬└¬I≡□╖□╖□╖°▌vi:

·Sdö╬»■µ┤T⌠¿~*X=╜□;±□¡≈ro□Ωëê═W_é▀□:U╤6╚┤□╥□]î═π└Ü□áÑ≡u▌ò°°□>|□/
σ)|√□£Ld

¼z□╬#┤╛□
∩é□□8ÄPts║y·□z>┬■¢Φ╗z·╒{Γ≤»▐s□└q┌█ü‖%b?╕
╕╚ ├□εÇf°╨v<-ΓΘφÄxπv≈a∞

#)╕¥}

□=□╙╤╢□█Φ□3ñ¥╗□yí╖:eHûß□zτtä÷Ar□mò╙û□#δn▒━~Q¿u≈╒aB=°□ττB¥ûαâB□┐□N9x□x□x*x
*╕□\)8# uàZwW%□n∩

2L≈öP╖=m▌□▐!╘‖□╬3εHÑíôPδ□═┴Æε╨┌X≥îτ□æ±ⁿP░h╢╞ê□τA⌠îí╓`Σ□ë|£V^σi£°q·5-‖

Ö:i‖

╬2≡T≡Tp%X ·σù╓φWi=N┐~àµ□ε(╒bÖPδ□¡l┴å▒h^àg^G├□∞□□

░□é°▀Y╚(Láε□Ω÷ªε"ß╗□y║H₧Üû`ôQ3-═□Is□≥ÅC∩éc1^╧
UX·5R¿┬╥»QB□□½║□UX┘f.àZ╪╓□█Ü╨zw┌z-▄P¿q□

₧┤▐Ç2╖¥ @ªê╛□í╟`ª▌□▀Çoα□∞TO□ú≡TíûüWK─½₧ç□╗□Σƒw□¼4┐%╘z^s≡╥□iJOik⌐╠mg□3|)ÆK
æ9)╘mLïδíKÑûΓöâùüùü°é°é+┴òα╬□7□j-
¡┌«l█Σ*□╧¢╪╢ë╣▓┴GÖ□┘iÄèH÷6╫ç=□àâ=7bC╢╪l~(╓┌≈H40?─½ΓƒE╨3Ü□@≥<|=K¿y-╔≤╚
o₧□□╟KδÑî┼k°╝□KJi≈      h9¡ù▓«╦±@)δ║□□ö▓«╦±@)|≈
<P*+┌y□Z□τ╧°ⁿN┌·=m▌)m┘┐□£Q╧□kÄ┤ÿQ▀#c=□¢τñ·╦α≈Su╜Φ±í╟½{╟╠╣□▌åσÄε)╓:╠
g╝m/╟'G⌐u□²∩#yö5╗\V£y┘yWßAB═╦H^û□πj═□jùBπB═╦εyß□Uxx-
╕□\A▌Bj□é{P╖ç`|▓█y[═ûªB¡▌°▀□lOÇ├╩╡·8{□τuíV═┴åMX╒□ⁿ48∞~(Æε3□╖@□□┌Äá--
╔+Hvü╙à▐}åLWíµgÜâd]Φ]⌠⌠}Φ6·x□}▄F-
∩óÅ█Φπ]⌠±/╪╨Ul0?c¥╢ú¡□G±¥□íû^¡%Φ»─₧□⌐½÷àBë?ó┴iI/JΦET╞┬)□jE⌐□Ñεç╘φ┼╙╣╬X┴≥╘
╕╚╪M□j\╘□∞°/Vw( n┘╕(cgM□¬░Ωù⌡}í□WÇ+¿+Vu□¬°╩6≤7Bìï╪Vç┘▓Ä╓δα╜-┤~é╓□j

┼å/æ□□Ö/y·%k╢é ▌□ä_□┐éZ□⌐UA⌠≤ 5+≡¬═╠Y┬╪┘°ñ;~h□╜E≈T·σ▄B-
╗cφ=îBêQ╕çZ≈êñ╙□·□╡ εí╓S╘║G╝í≡>p□╕éZà╙□éδâδ#ô»≡»äZ≈T]ö}□;▀gv}î╡╧jL-
ÅßìaN□┴┌-ë~µ╟î∩<·~□·1^*├≥OàÜeh(C▓□û3╖═ÖH«á¡a┤╡□╔□H╞ÉYü╠rGφ┼÷╧äÜ╥i╜+▐k╬Xt╒
□<╙□²]±LK<╙□╧┤─3]±LK<╙ò╡Pëg║▓▐+à*,^·□^□CδWi+╘ê_%╩╡□∞ΘîOZh

║╛ ╞╗G░┤Ñ≡╙□WΦ9F┐6#┐□╬f═í°ÅBW╤▀═¼▄U¼▄╫±L7<≤:≥»#9□╔╫Y┐▌X┐°¥ƒH╝□j₧F≥┤╗S8╨±B
═
╨╙ε|8≤┴#┴#┴≈â∩□┐
~□|□=g□3ƒ²╥è°□-
·±|O≈O□/ö│â╒ô╓/I^mw□j^╥□,·□6□à₧J╙°D╥╜U°╞|Å√ªΓ|,╘≥H]╖▒╗MßÉ╗Y±?s▀□î╬*lÿJ»½4
çV~□▌Å■*·╕ƒ>V╤╟²⌠▒è>ε°ÅU╪░□¬□±┴ε

┌╩╚□íf = ╒)├▐*╘<V╒B(¡lÑòch█è╢ch█è╢cεzYGBò²ΓÑΕΓ%╙f┐nΦnQ£?Ég6$·¥û║µ

í╞Θ4G⌠ ╖P½□┤□ ·╕îá┴□Z□B
F╨Ω$Tß≈□n%÷□îÜq┌y@4CÅbßπ┤uT·bⁿ□╝ÿv «9i·║<ò□╗`╔
z1₧^£└W∩ïñ²ÜHÜ∩°mV2µ3Bì╙□1U≈ Φ□□G╗H╞╤¡d∞tV□□o□¬╝$^²ê□├i:╬|y╧

┘&πΓ▄à|>u≈jî╢║|4□□Ω╘CC╛╘Ux>x$x$°~≡²α╖┴∞┐2^NSF-
▀Y.9░PÑ_¥=φq╟ ùhw□k□ƒ╢\ⁿf∩□╧╪□Öô□┬?«1û□╟ç3íÑ<▌┼S2:│□╥○ΣΘxzÜ∩╛╥£æy°(sróPC»
)┐sUféP≤□Ü#∩□▒y3ê·$□~èLê1:□¥G+□▒°æ÷?->¿=╠z9ê□B¼ùâ°'─

?ê□B─äyB□□_5□j╛ç■╡╟£≈╨|X░√ûPδ░µH╖ε[r█á≡H░¼═
╘¥@▌
⌠e7£upv│Γvc·:ⁿ│□;╫Q≈ygÇ╥vQ¿·|·\#Æ^$□ªΘS"#½╥┌!>┤□╣Æm□U⌡2ΣT;Wh□}╧h`╙yP8uα¼â
SG≥jurΣⁿ%|≤□╘Br⌐ª┬qN"┐╘}C⌡«éQ+σi)O╤σ⌐√□cz^°*√-
'√□═τæqDF┼[yz╓S╟è#b└⌐é│_8*v═□□
äo╛'╘á«ò»)≥GD▐┘σLd«□?□  o╖1ä»╝:»╨ε░¢°δhσ2φ^÷,
+ε⌐≡68□pεé≤□9÷≈tå&|≤ÉPò}═%╟Ç□Gσ0J▐
9□ú║┬∩□┐%ⁿ;£┐╙>ê■═Φ\E▐╛Ö╢Vëî╡Üsô>¡-
g^5ü□â╙□N□°Å□]»`8m9╫□─█║Θ□ëf¬┼áτnàƒD├b4|┬²FCìYπq-╣└┘Σ
ì╤0K╓╖:╔╧▐7ï╡≈?£■6□5 □Ö:╘*ÑV¿*e╚╝ü┤;M┌u^µ∞_╥²/E?□≈EO.τª□ßΣ□╟Θ╚╣i▓P╧
\ìß┐í╧╤óYφò╥║ƒò╡ëU╝Æ~mÑ/+▒°□u╫ó┐Uò∞Y√░p□□ε╙wG╘zäZal.├'-ßöΘ□¿▄╤9σ╨A╪ë▀∞æ╨
Hfh*≥-NpÖ□B=£_<┐É╡Öi□⌡<╬^╛éâ√k╞n□╜□$+╫╤{⌠W□□<X₧┴²âτ□⌠┤Æ°₧wà║;D╞Ü╨Ö±$g S£|
╤8o╛«o6└╖Θ{□N┘HÜ;⌐╡Bƒ4»╛H[½òà/qâ▒V█∞¼□¢¥g□πç□⌠h□■□╞}┼-
╣»≡ê▀□}'s╖g/3\╬åP□I□?ÿ╛½□ⁿ(2'╙╖□□
?¢3√ì≡□├┐□Z«O±i╝ù╣°² ÜΓ<G∩fa╧Z∞Ö┼8fñ╧αδöⁿ□∞□wsª~æ[,εï£m£»╧soαΦ√@≈$3φñ`╣╣╡
₧ü≤;8╧□G¥εà3Z¿y-ÄÄ┤∞\µL=»╛:°Φ-₧□è┐├3E0□`

φ>ê
c4°╨é□(Ωç╞íu¿[J▌-X°:□N@├B4L@├□n□₧π

dé╛¥□
*bï₧.╚□A
M╣'£è6Cd∞□Fv(╥□%ó■≤░Φ╥G" <-╛Eï╧k

Ü│±τQ°ì□[╜êf┬┐□▀~□τjF7┘▀=□%╬g,□£╤╧ïµ,=□Φ,¡Z□□|'√┼∩╣τ╢S⌠╪┐□·îz╪÷K,±ó┴ç
^ⁿ╨Ä□╤+╚╟få_@≥6╔╚£ZB¡█Xq9Æi½□^b≥¡nê=Bq¼□yù┼ÄFV∞₧!·σÉ├\É┘e╛ƒ╬□%□,@gê\æ□¼vä
zr□r╖
╓q¢<Ω#rí&╟╧àé¥Åß,Dfí╬p╨ □╬□╦ä:¥╚+å└_#╗┐▌ò□`
╢φæ¿Æ╤ì|c□zvc╧□ë□n3b┼>b╥^"Fäî(ô╟┴/9Å°û[GqêTûùⁿ ÆP╦K6▓Sƒ/2:Æ!td╖M▓╧▐╚╤╡ô]L
z╘Y█ q#ú□v.ÆⁿG¥¡╓s╢╥TƒD╓s~╤ö∞¥,┤çPâsÖ╒ÆL∞□2G¿⌡
2ƒæâ=ê╟±t╢&£═┤8□╧l«J╚ê`≤G╪£┴iδ
íµR<╔□DìáΣK·α╝£

vô
«úwcΦ╫cî╤c╘Ü/╘ε.z▄úΣ└▄0█ ╔û╔·φε°φÆú·n»□¬╬å□%J╤└■e╓'▀[

¡Å|è□ƒâà°%zÿWÿ╖-ΓCê□æ╔║«é«⌡|í8□□¬╫_≈s□ô

╢║ú₧ì┬W+ε
rñ□▓╕□#□C├\≥(╡║+Φ$╘.bo¬#πÑ░╟ÖJ╓!m□7B°gúp╘¬┘I+Oq□É╨▀□|x□ⁿ
╓{)£]pJuåπiJ╬/τ²R╧séσ╙4ò╓□╧ÉS▐W□?ÇΣ□⌠4D□≡`·{dkk╓╓▐├₧├X°□Yε-jm└₧
╡╕ü£y*9≤n°δÉⁿÉ╣·2≥─1│»°ÑΓ┐ =,y╛╥9ùZg¿E^Jl-
Ol∩αæ\nóτIσ╜⌡¼ùÄrkßd╓ìì╒æ╡ □╖□□╕├·"═□
/:ì%ôt─█□¥IJ╛□■□ïm□⌠╘E╧SÄ∞â├4fv=ï╢Mh╬╫8}□+╖g1g▓Γ╜φ∞ùêφ<-
X▀D!□ï╚□U╠Σ□Zdñ£·rChp+h~*╪╘╖
fâÅsç│Ccl□□╟▌%□;Hδ▌⌠═-7ì□Σ□Oë%╓;╥G╖5}|□╓ ╜╤úG╘5/b[w·rQr3;

▌□□ë╞H.τ^h9÷≈C╧T⌠⌠Cs□lhëUƒaφZ44ú□Öî·
£'α∞t$n?ΓH¼▐I₧S(╖L□█╕╫*ä│Lpf/8╥╪)å╤╨%h□£]ÆW╕□□Wƒ&

πI╧#xë╧□=ú\╡sex╚╛□`φ□▄k
É{9╖)╖szw□⌐oby║C▀yÆ¡uµN√A}□vu₧╥├

╓╪&}±┕┍╥J.ö┘K≥4╖è{┬║╟èN╟è½U┐P□åª∩╟σ

░#7Ñ¥╣□ ÿ╗┴yαÖ°╓½∩i╤□e4╗ï□≤ÿ│UI╛"╘▄□τ2≈râ╕□┐ç□≥□wt¢▒yò■¼┴ò;╞å─└╦dS}▄&ΩΘ
= ëè+\┐Σó╚¼Ç3Å╧□▓D╞□¿?∩ ¢▓hq□-╓&

Φ3i□°Gα{╚┘Ä─ì≈5σΘ-<¥î╧úX[□'è|ö╖S/w°Q}├/u╒ÖÄLLƒ□í/╙;╜½
w▓┼cεbnDm╤∞.□═╢ìµε┤╒Ä╢║k

╡^╥w╚╪÷,~√é¢ⁿ2ù√d8e┤╕òl¡ô°âp<'└│±¢╘□åΣ□$W`m╕j╕!τMΘ√Uεçâ£+»ÆA╡ÉZ₧╬XX@¡□Φ⌐@
╧ new□Ñ7├_

□-3│16∩ñ╓δB□/+n□╡~□m!w╦J %b~□féα╦₧;╚f□5O▒KûôM¥b|τsÅz°SO0£?├·-£?_═òOj╪-
CdD½╪═ éå»É èlj

;u-8»╥╩□Éyò□v'Oδ#┘@c$
╪à▀ü□p╫wL╛ka⌡vΣÄ"[¿╒□╔]r√gsçcεb?)▀äQæg░αt¼□Lⁿ-
,□Ö□≥èφΘ█╬2à□h√□z-e┐╬'ô|□■\8╖└Ö
τ7p□Éà□\╣uY@-╥V4x2╣AΓño╖à»?┐

æ□5τΘ□ΣÉ═æ)ÉΦτ■A¿¡○

▌ß«rÿ#ƒÆ╝ H₧<âô■ï╪□₧║#░÷A∞·+÷î └┬┐ ≥t║~*≥╞~¥ß@╗Γ╜2í□ƒ

X╖
VqX▓╡┴dk;±╪B|╪â¼{□╖□[□ï□p~

τ□εxW▒□∩à≥}□≤□{┤□╟Iτ╧╡T¡τá}─6╡G<└>½Ztεπ&-é=-≡╧YP₧╢HÅ▓p⌠Φ∞"┤║O╓w}≥¥□ò
îfo□Öƒ└ ▀½φ□F°²□╓fóg&Vu└¬□x■G·ô□÷▓i£X╥8▒NC■ö#2δ□┐▄╨2□╜9¢|"g□δfZΣ~█║
9=O$╤J2╖□·Té'╗Γ╓≈ßTPδV{□kτ□fà∞╘-
╙□≥⌠╟□□Θ#+╥3□╪6d╙û=πα|q╡½╥3FN(╬"gà`zΣπ¼□ú╫ï9□∞□□≈

d÷▒r#≥╘□δÄ□îL□µ╪}¼P>≤╡*ß⌠e*u╓─▌□≈$□□"F
ó□╥ó+┌L╝a?*½╥┌ u]□
▄¼┌φ╔╔│XΘ70:δxzCz▄Eµ≈B¡ûp□æ9äLí╛Arεcu▀'□½╢!╙ûy╗
╬¥ç±·├é⌡τ□U╖╔□□█

²y²v∞|à┘▓□█nbD8╡║£µ,NsV╪□.ƒ╨9A┴·₧
;□╙¼░·╨7╓£ë÷gέæ∩k□¬8O┌▓│╟vεT°ö| ╧ÜM+1Öo₧▀□Kch[π

Q°□íjÄ±ié⌡ó`íµ^z7ì▐φMτN□≡░p>Is┤╧Ñ/
ßδ╙δm¼▓:╟╧"╟í□èZ
u╫iîUEÆ□╕?□¬"òΩïIⁿ┤ □═□Ω±iLï/aC□┌z╬(<v

╬(╜╓ê≤'□╣□□$╘\I¡<d╕□┤7□ô¢┴┐ÇåfX■k, _, 5£^╙¼Fµ1N·ƒ□±-╙╖□δ║Çu=Ü□¼□₧L▒│─4F╧0
8≈├□╞)∩□µⁿp¼}□mçßtÇ≤□ƒb∞Γ-ασt╓-÷ôYa∩□j-ô¿nNÆ∩┼Y□5'Ñ?╣╖A╓ⁿ\╥│
□≡µ⌠
╖U·≤╨úî┌□l{ä^?@»╣]┤□├Θ□g0£₧·ôJbl□Z□φ╤z□□□╝□g□⌠·⌠'¢/(|ê~▌%qLφ╝·∞╙□²^Ef□±ⁿ*
╡▐└█·X■□□╛╢í÷8c□∩ë═0╧Ü□7/¢W¡¼║¥Ω╨¥□□këⁿ□□╓*δU½B▐Qj╜g╜o}`φ╡

X□╕■&o─╡>ô╖è┌*
│]█c╫ûwâ╩█@φf≥╬□;j╟∞ä¥⌠╝R√╖ⁿ_E├╙eT║î╜å¢□âî□⌡löΓM0&□╙îÖ╞
╞<c▒Qf¼4╓□δìM╞Vc°▒╟8`□1Ä□°î│╞EπèiÖÖf]╙kå╠□│ë┘╨,2.Ü□»}/5²φZ·vñ╒¢□wA▀╡δ∩1Θo
?q'╨≈┬Tv £|╧k╨O╡┴▄±█ôy┌æ║|KHƒ□═Md▓|nou÷▄hñ┐gív6æ┘□τ-
≡9τ□│L₧^Ñε)¥Ui╩═zs¥;╚╖8⌡τÉ·□0╡□g□°mµ8╖Y*b²ùæ■┤SìdÄΓ(O(l▐áµ»1▐pîl┼
□·╩╗MìV╞¡F╪ê□1ú│q°qù±□πçF□cá1─□j<jî6₧0╞□O□O+┐ù□┐µ□┴KìWö∩╫□o□□╞ƒî-╞

c┐≥■Aπ#πcπ□π£q▐╕d|iT⌐1░MGm
-3├¼e╓1o2│╘x40 CìIC≤f│Ö┘[■┐#sáy┐9╙□lûÿ□Ö├═ƒÿÅÖc╠ 6'ÿOÜ□=°╠Iµ□s¬·¼·
s║9╙£k╛nû¢□4╫Üδ=Mµ[µVs¢·Ä·«╣╙≤Jfô╠ª≥₧÷╠╓Öm3ï2█gå3cÖIy□aµá╠□2□,▀kV□╞+Gx╠┌f
Ä□P╢42[ÿm╠

fT╤ö┘═∞e÷Ñ⌠W□ò¿g├═QµXe╦dsÜj²□s₧╣╪,3WÜkh□½╣╙▄c−0ÅPÄ¢°╘Z╣h^▒,+╙¬ky┐÷□▓□¼&V+
½Φ┌OG+fu╢z⌡°Θm▌k
░□+ε╨»²îP%fì╢╞Y□╒∩Ωƒ)╓tkûó≥3╟Zá4.Qr╤□Zeò[□¼═╓vÑs¿╡K¡╟C╓Q╒·■û:²Y²$+│[8□╔

v~°Φ&┴VÑ≤╣!▀@S╘>τ,5ΣFNI□çàÜC⌐╒M¿╗L¿πü╢v╞│╙=D₧·Ä£»╤pE₧z ·R½□┤=┘α%hª#9╒!√g□'
m·~B┴╓D'K╤ßBφæ÷□┤)Ic╗P│P╫é│G¿sF¿Ωà╨┼÷s╥
⌠tf□▐¬⌡≡┤ƒPw□□.AÅ@°AW█≥IV3ív
í╓□{□╗î─·Kpr▄■X+≈τ╡àc∞□l□□¬Σ□o□y7è₧□tnñn□[n╤¢╪s$╩╪ï▒s╢|

E▌╒╩0⌠x┌□┴²¿╖└-';□ⁿXÜ.ùL;¡a▒x
½╩□¢G▒ñ╣ß3,╥░Z□ûa╓¥Rwªaⁿ ≈╟²?≤■8╡├dì6î¼q¬LTeè*╙╙╝Y¬╠Qeü*Käτdgì╧Üö5⌡
[è╚╠╙Ü¥57kQ╓╥¼σY½┐Qä╖6kc╓□Uvdφª∞┴:£u∞[è╚£╠¬╠║Éu9█°gß∩₧∞┌┘9¬□▓□d7╩næ▌µ[è╚t
╙Äf°▓╖e≈RÑov□╩á∞Æ∞ß¬î╩-
₧É=9{Z÷╟∞□▓τe/╬.╥^Yú╚▀╫d»╧▐ö╝5{τ╖ö=┘□▓Åd╧>ò}ûr1√JÄòôÖS7]□û╛σx Y°{(°
°ë*ítiòSö╙±;□æïσt╬Θæ╙;τ▐oö□9â»Θ¡Yåµî╚□]úî╦Ö°¥╩ö£Θ9│rµΣ,╙Y≥/╦▓£Uö≥£
ö═9█┐S┘ò│7τP╬╤δ╩ë£3ös9ùr¬r¥∩Rrkσfσ·r≤)à╣=(¡s█S┬╣
~w╓φÖ█'╖_ε└▄!╣├rG^W╞ΣÄ╧¥ö;⌡[╥î▄┘Φÿ¢╖ê▓4wyεΩ▄╡_+□s╖\WvΣε■Z┘ƒ{°;ùc╣'s
+s/\W.{
»╟[√║Æπ
╘,⌠√;□o□o#o
o□oç∞▒ ▓╚│¿7σφµφ□εδφ ¥╩
oëw╕w└uEtîUeéw▓wÜwµw*/xτy□{╥«òò▐5╫è<_»╩&∩V≡N∩-∩□∩□∩q∩)┌·f9δ╝╙∩+y╓╖ò╝╠╝║y▐
╝P=·y□yM╛VZσ□}│P╖c^,»s^Å╝▐y≈≥{@▐α□i╧┐)yC≤FΣì╬□w]Öÿ7%oz▐¼δ╩£╝□_+K≥û]ïφ5b±╡
XÖÄqy½≥╩½cP▐å╝=5π╚╡9Rs\½╟ñ┌G█≤v]│yo▐íÜ6I,╔;¬ΓëZ√y't

╚;ô^┐ȷ]σ¥╦¥*√å╟≈╝K¬T·£Ω·∞½Ñ~½vΣ╣/╦τ≤σ√□}=|¡}φs.╔■Γ
√□┬ù╛·║°z··°·I|⌡
⌠
æ8Θ□µ□Θ□π□/{ÇoÆo*▒]⌡Yµ╖oåovu|÷=⌡-≥-⌡-ù~√V√╓è/|□}[$vèN╩

▀n▀~▀a▀1▀I_Ñ∩é∩▓▀≡{ⁿ╡ ²9■Ç°□ƒ*_è□²
╘>Ö▐╩ⁿì╘■ô÷│┐à»ƒ┐ì┐âΦαY╘ƒ≥w≤≈Æ}τ┌>[sî╥:╤[╝°ñ≈□▒I÷F□_□□▒═?╚_R=╬╚½▒c∞╒╛,{₧⌠
═?▄?Jx■░j□ƒ¢.▓_»²F┘»≈e┘»╪Åe□«▐ï=Θóµ□}√µ-;H□ ä∞yR╪c½≈╒t±O╫>+ÑzÅñT∩ì5÷╧»φæ╒
√d║°°⌐}P÷B┘√╘~ΦƒÖ3X□udƒ□¬╦╡ÿÑè □ <~/÷ù·W·╫└W±├┐▐┐╓┐╒┐╚┐╟□└□─□£y¼╓░∞-
¼[╡Äd=·O·╧·/·»H,□XüL╓E⌡:¿Äïjnë-ësü║*6Ñ╫êîù──Ω°cαukδ¢δ*□_¬φGçè¢□o
$c-(□4╣V_Σ╒z
┤□□□:□ê;ü╬ü-ü▐ü{%å□ùT□□□â□C□#¿≈m1(mW`t:ÄW≤╟╓ÉI█L_┐□Å½√#q╕║ⁿ╖X≈oΓi`\·≈D5

¥ Y«ïô5cÑ─╟Ω□Y#-è,zDF=□□ªΣ┼□╙□│□s□
ñHn#π-9M`I`□<□│□½□σü
ü═╒·K`{`W`oα□qLσ□üúü□Σ□*ª□╬□╬□.□¬¬séá□¼%±î²_≥□□δéYAƒ∞╤┴ⁿ`a░Y░u░}0□L□╗□{□√
□√□□□ç□ç□G□╟□╟□'æÅÑπÑ╘%7KτMΣ<Θ□□]i□≥,858CΓÑ╪u-
»½╬├¬■□â)╒9L:≈□]ÆÅ□g□τJ╛□\□\Z]_ΣÑ?ⁿ]·ï<K│─┐<┐□₧Σì╒Ñ:O¼Y╛Ö
Vτ~5K┌»╫σu╒Er▒Ω≥═£«:G√□╣Yp¡.▀Ü¢IεU3 R9╫╡╝½FÄ%╢RW╔T√Σ║╡Ñ╓_pcp╦7╫UpGpwuÄ□▄-
<□<□<)▒¿Z.X□╝ ≤:x9d╚|║□╟DF╓£Ü□≥;Σ ╒

σä□α□íFí□í6Rj«╥PçPTbD(□Ω&≤3╘+╘≈║<FòP ╨
)╠GU╙eT▄□òäå≤{Thl⌡□ö5□Ü□Ü□Ü□Üym²⌐u□z!4O╓[hq¿,┤2┤&┤>┤I÷₧Ω"²ò3û°I·□┌□┌□┌□:
║%~äÄäê┴╒≥íSí│íïí+⌡¼zÖ⌡Ω╓≤J,¬±ÄµÄ⌡b⌡:╤■'à8⌐réz=xO≤□ë╟⌡□╫□*≤T÷┬z#x[3∩i□□U┐
½Y╬ ≥Ff±ô╝°YΣδm«╖¥w5¬wTr@ë ╒▒·┌[¢Ñ()▓╧╚▄û╖├ë■╙∩n╬ùyû_ÿ■Lⁿ(πÿ■:┐═╧┬·
╤æ■%┐°─≥ⁿ>·²≥□╓xg│╜▒Y▐╫£~S│°W▐╓L□S²ùw6≤{G■nÖ□╫█\Ö□A▐╪\=□$□ùⁿC▐▀,oo
ûw7├O╟\yï│╝┐Y⌠╥:⌐·-τks╡·□P╜G)\┐ñ■p▐⌡<¬■Xß╔σ¬|G╞0£│B=^≡■└ux½─ìα█ß □╬_

╙·¡gë╨*·£≤╖□├
╙SO8₧zp·≤┤?╕3╕│╞"Θ£üs□╬□8Wß\□Ä}R░}Rcyj»Ç┃B8nm┴nmì⌐{à║W╨vE8n□Öäp,G░σhîⁿ,Σg
┴yÄ°╧i╠╙f<mFδ?ó▌ iî%â5à3Æ°#iw ╡□□÷|ê-
>─┬□X╥Bcl╤Çô□°'╕°╞<²-£∩íτ3⌠|å■B⌠□"9□□lp·┴∩°▒÷□⌠□╢¥└╬□hFâ½5□╥╙i╘¥&£î□é3□h╠
╙ª<mèå7╤÷ª╞Φ┐□τ□┌□áy□6αm□o█∩"≤.ⁿ□ß?□~□ⁿ82□æÖ□ε
εì╠$d&ü╟é╟éτâτ
6ÖEª₧E╠=ç╣g┼□╙□:▀Aτ;≡Å┬?□╞?□■▒╬é╧"┐□·}°▀â =Φ/E□)╕□\□^□^□^□^Å₧╖╤≤6ⁿWß┐□ S°
ƒé□k¢▒╢□□■!≥╠Iç9i□□ +∞í_-▌»¥≡wéƒ□?
~□ⁿ□≥╖"□+:█ú│=2╠g║ª<□□-╤╙□áß-
xµ°xµ°≡╖└▀ó╡1Cεá╓□î┼~╞b?Æ¢É▄□ □ⁿS≡□□ wZ^4X·Φ╧çC╖ªnù^[║╫CΦ┼□┌]Lïï┴□┴□┴Qp□y
<π┴3╓

⌠∞á⌡å┤ ▌É╣>°¡╧æYë╟J0}┤Φú╡□╝ü║╟:¢Yτ«e╢»à╧L╢⌡L^□^□n

n

&:┘D'w6ugâ╖é╖b "∞_□▐□▐C▌
¼╡ëBM□'ùAëTn-□≥⌠□Ç ƒ╘²O⌠□□□báúG\Gî·╘¥@▌ X╥□▄□=x╠╞cn]$δΓ
╓ª┼┌┤ÜÇ¢□+n n▄Çµπ┤r□=»áτ□°q°qtó╟╒k<□?□«
ª¡╠Wñ▌LΩf╥╟L·Φ▒□Y□=]╤╙□□■ê
□□{┴^ΣδΘxÄ□wá≤y┤=Å$q>â8∩∞Ñ⌡╜hnäµF⌠é□l□üm╞╬f∞£■∞wó?□Ku╟ñ¡╫É|
╔6hnâ=¼□□δ┼JΓ├$Ü╫ ┐□yv□G∩□¼wW»≈í╚

┼rvÑ

v%□q█╙@»/1°3□■

□m6┌¼gi±YZ9ì╟i4ⁿ□
┐□│3z⌠╬╚ⁿ▒ÿ?÷(⌠î╥1ü>6d²«df~N╗─□G╟èJΣ+iδ□°ÇùÇù
├εc│√╪τ┴τ╤ D╗E╪□÷Ç]ls▒═iç₧v╚╛Å║≈üÖ 63┴₧  ₧
ªÅ6}┤⌠τÇτα╔]xr□3φ-µ╞═┤E÷ΓÆ╜╕°╙┼ƒ₧≈░τ=Ω2"□îêç║-Ω:┤σΦ╣ê▒61╓ε□εñ#?>|□╬ï
┤┬■σΩ▄Θ64▄□ÄÇ#α╗┴wúa7uwπ▒O≡╪'α/┴ "?□·)X°c,ⁿ1ⁿ∩├ >φ2W]µ¬█□▄□=3╨3□╠:╡X°□úf1
j╬□⌠î□π□□□╪╪ik;□}¢╤╖ë¿╢Ä¿oÇ▀áε├╘┐□╠║vX╫v□Ö ÷ □√□□f╡zX¡□╚d
π0ï□fæ;Ä╛îC₧î╤C╞Φ□φ□ó╜├^Θ░W┌∞ 6√ù{□uoB&□Ö□0{┤├─m□╙lbÜìm6╢YdMû╬Ü╙□lr□ù╣\┌r
Fúg4°e≡╦α2p□≥D'Wgí≡m°63╨f□║█É┘□ □ⁿ#⌠ùXΩ!û┌Σ□69Çu

▌╠╤□q╪!

╖╚╕ :O`\□=.□α□⌠╘BO-⌠╧C <dê]ÄÄ]□┴□æ∩â|≡ ▒≈=≡]□W╛s ▄▀

╓/)ª,═▓<JæÑ1╨H)ïH)M□yHæ╞É┼ö╥4b─□iñ□□iñ□Y□ê╚╥ê4PDD─□□□1Ñ4e)σQ>.Å"ÑöeyHi╨ú╚
≥(ⁿε;τ;≤√±K□□+·^?╗Ö╧9≈Σ▄Ö33g╬£Ö¢╠╣7□t.hΦ╓én-∞□-
∞□▌╡É9□φä«LÑ+╟2□│╟~□⌠█á▒R█j7□-
h├□□╪╥□╪╦╢ÅBµú¿δa╘⌡0Σ_äⁿïαcτ`aτ`╧Ç£□á□é>êⁿ≡6ª≥6░y
6»NΘêsΓ"ƒ╥ë¡#| √£╥□%n□ ╜═Y¥{─□Q┘Θ□;┐□{─▐6τvNèw─∩█£▐▒S'v≥Rguε╛Ωî╬cW¥═y=u2g
Å=╧~┬~┌^fo╢_╖□f╖╪'φw∞wφ≤ⁿ∞'NP/Oê╙Tτ□¬╧6┬T[Ä╤═ΦA╕╖╤╧□áÆ8
□d

5FΦ4╨□k□□□ì╓╞□úèZ2├ÿm╟5□□K¿
+ì5╞zú╤╪ll╫⌐□░╙╪m∞╙ΘÇq╪8f£4Zìs╞ESÿ«□53ëΩjv7{Ü}ê╩7
═┴µ0│(òä9┌□gûÿef╣Nòf╡9¥≥═D;⌐]╙GwP+╒C╝|█ⁿ╡α(□>≤▓é▀ⁿgσqñ╕╡

τkLµÿ∩Ç┐Ç·ΩL
»┼·bµù2v÷3╣√üs╓▐ï□k,°□ⁿ₧8_sÆi╖□y╩üï ┐□╥╬ß£K▒>≤╥S≡□û#▄*p-╫τb·
}ÜF╝ï□7#»£ù□□qé&Ä╙4δü╤·öì▒ÜÑÖ√╘□□┼G╬ÑáMHh□«□^k-└°
┤a□ƒ·┴Θòq|╛╞l┴╓ù□á□X»▒□Σ≈qµ%Kƒ╛!,~╟y£□H╕└╪□ï▓├q╛f□sî,╗₧⌡ÇR98/╙□yVΦ≤5δS│»V
╠Γ┘g▐H╕Çf_&═╝O╚¼+└¼π9ù£iS─╜4╗□╤╠ZC│Ωe╟*₧S╓Ö⌠Ä8E│G╥╝□â□Så□Sà□ô£'»□¢íâJÆ□σt
π╒τ□¢┌£q╜·öú┘µñc-N;2εk□□-h

1ås□Fb▒╧°╗╞°{·Y□t1ÅÇ╣ÅiπKáKpw8Φ7@D╬□áâá□ßεOQΩ"°

~!Φ>╕δé■□Φ∩)        L□┐F╬S╕;]╤╕¢⌐┌□╬▒□ƒgΩï<ÖÉÉ□<
w□(î╗╢Lδ╙ÄùL□#·≈ u▐╔²╧r▐ëµR░Å▌%╪τ¬ö,

KÑ"■û5╥╕½RI░î┐\ìT□¼ª4¥8φ╙╟`]p-Ñàöû"╒□7≡w°u┌□lΩ mA·ª`│N-:φ
ε°tÉαH□Θx≡T≡L*¥G^ò.⌐□2»J┴É□èçrS)/╘K°╛WÑé╨└d]í!íßíß┴≤íüWÑæí1í±í±¿»□i
R¿é≡T.í╚┤

ñ≈
┏ó|▀ñfC┃tÜ□ÜOi1┸ÿ

╥▓α■╙èdóR½Si¥JWk*┤1┤ë┌┤-òväv!φM╙D2╝□:D╢ÉL▄«úí□φ╥ié│í
HëP"l+~8□ûäsR╢R-l

W
≈Φ ⌡

≈
□□□□□E□□-Ñ╙╪p1²>æ╥Σ≡─49⌐□₧□Ü┼_Φ╓⌐8\ôLZ√Ñíæl▀ß□░▄▓≡∞≡\╢▒≡□╓Dx
█Gx9Q+╤█□ß5ß⌡h╤zHWÆ╚R┬ì□úüß═ßφLàw▓÷├╗íΘ╙ß}4w□ç÷╥╝□□>□╝□>LZ«□#
'├¡d╥
├τ╙┌¢┬□C½#é,·H─ìDC ¬╖ò,Ñ%T□╔îtìt□¡ê⌠î⌠ëΣSï┘■ÅD□╤╦à4b□â-
æ┴æε┴≥╙░H□╔Γ9ï-!°Ü+<║-
┴Ω╙ΦpÅ╨┤`ƒ╚8Γ∩°|├h╓φÅö□58╝2R□□□)ÅTF¬#╙#3#uÿ╥╒*EµEx╢.î,ì╘Sjê¼Ñ┘┌áfldCñ
╡QMæ-╘□╠╦H
I«Å∞ë∞Å□î□ë╨□î£╥≤ÅgαÑ╙Ö╚-▓┤□╪█.║{>▄/öê\□φèÜ╤`╘
Åè╞iti┤┬¡╤▄h^┤□iεD╕□╡ΘThS┤o┤ öê

ñ4$t!Ü

d
╞Xq>Jd1¼ÑΦpéæ╘≤╝αæΦ□Γ╫D╟GKúCóôóTwtjtZ┤6:+:ç╞e}t>█{tqtYtEtut□l╝\ìyt#┘┌d¬uS
dOt□Ñ□╤]ß□Ntoot~⌠-▓╒~ßítτ□╡■(█)ßë╤□╤╙╤│╤
æ║h"4+fç½ba■╨<≈-&c9╤·└╧¬p
≈/╓-□Å⌡ê⌡&¡⌠è⌡

#+=Ñ□
-ë
ê
"?s)6öⁿD▀╙·╪ê╪¿╪╪Pn¼8║.616Ötu╕ò┤□¢□½è╒Dâ▒□▒┘díΣ9╙"=╪└%≥U□*□I£□[@▓╪█┴éæ□^□
□▄□□-[□<□[-2c+Θ╬j╩ùKφÖ-
[CT<║)╢>╘+<#:?╓□█□█-█╓^0Θ╓b╖Cï┘╙E□ç□├ì▒}▒□Στª*_□;□;╞╡qM▒ôñæV÷fäτ╟Zcτb□=ß╣
^4╕?║Iy.°«uß▌^f∞pxö╫ò[Γu°q"█í▀{z}╪~T□╖R╖√z·∞ô╝┬Φ□opxP░▄□Fvuéj9□□A▐ó▐+□τxú
╜qt°─+#╥ÿΩò{n¼8V∞Uå√□δ├CC≤âM^uhÆ7▌¢□k│Ωh□┘│≈Ñ5!□¬⌡µàµ{
╜Ñ╤ß^==₧²▒▐^C╕ÿ∞▓öF∞¿WG3╕é|╓─α~o¡╖┴k□φ≥╢x=^ï╖'╪□Zτφ≈

zñ)∩╕w╨;□¬≡╬ô╘□∩Rå□□OÆKcs╜▓PE¼8#ÿße─3r3≥2zQ□□Æ∞uΣδ+2·f□d

ε╧□□Ω¢1£µRyΦD╞H*sé╞'æ1&┤+6"c<ì╤$▓æ□ßVop¿4ZÉQÜ1)c<ΘauFE╞╘îi╤╛□╡□│2µd╠╧X£▒,
ú6▄Å«+óc2Vg¼ú▄□⌐╡}26el
╫d∞╚╪ò▒7π-
»%┤8LO]ë▒>=□┌╜x▀h≈J<C╧□╖`□┘(8Ω∩□ΦYë¡ⁿö└□+□O*╖&₧π°□╨CÇ□⌠□NOcÉ#□□7q-≤

╙⌠,╧yûA┌oqw5∩f¥²╙oαn□╫bÖL╗□□╬zΣ□" □|╫8□za□1□╙36±2∩äÖv╘S╘□α>└uè├–▒gBZ
·╡□⌠ ⌠k□8k┴┘å~²–Ñ₧DÅjQc□⌠e□·▓
9+┴»@Kµí÷░zåcÖN□εε□^Äû»□=□·□í=S└┘¼ΩE┘□≡wéâ□1

@'E‖╞□  9ÄaR}G{N gW┤≡□ zè□█□[y╚≤Iƒ Ä2¢δ□∩ó¥S!φÉε;s¬ τ0≥£`l
□}□ⁿòxε╪ÇZfóûπj□Ç
æ┐û·╓%p╞íÅ·ëKê⌐╗─z□₧╟╪8□|□£n╙s□t&°¢┴·=8úü□ü┐â▒⌡Eh`□╞½□m¢Ä┌╥Q∩QXÄì-⌡V÷π▀Öz
FkD½2Ö

μ0

□+[e]╣]QΩ□╙╬□ΣëéSó¼ù╥┌▌ö□╢7c

d□'8«ª□φ\
:£°□█Xé#U▓ÇGúT
t°%╨ô8°q

Ñ≥AƒG╬□HX□z>°□╨≈=α≈□τO╕╗□£CÉ╢□£[É≤ÅîΘ□÷□
□íσú╨ï▀í
Gy▄]Xú╣ä√kƒfL·┘è╛l┼|□

-Σⁿ    HΦ°╟t+$4α?ä<□π□æ»αε απ░£²É╣OkLΘü█<□╜8□-σÇ-
□.F╬J]π%Xπ%X⌡YXê╩╔□δ╬4yÅ│≡□£g"≡Bp╛å£╢¿+□9≈óT
≥,╫6╞w/└èf16□┌╫▌□>µ,F□░Üπ╧╤▒-⌐'t²ö▌JO╤<s
    □║┴óαC¼ô(;□□~╧▐□²ƒßz¡╡ë▀`.£┼\`[□°}╤Y╪╢╥3╖|-
·ó¼n-4s□y^□┐□╜(□}□°½╤╗□á╫â?"±□F╣□t□4□û FÄ□Gk╧S└□i├¿5a⌠□
    >zd╛ë▓█0╓S╒ês-Ü5ê│B∩╘▀4V(□`□èÑ£ç╜╨V╚Σ£9╨'k┐}□z║□=eÖM╨U£i╖□┤ZâZΩ╤╢zH
□½τ>╦)à□÷□.C□Nk╬╗□≥≈(▀ÿXåæ-└Z░□-
)@~üUâe╓Q^n╒d╠ö╓<é■□DÅ╟XƒB₧*σ█╤╬·J{µ/0ù╖┬J╣_═j¡D╬G┴ç>.ü□
N□¼

J╧küâ╕¢ç■

COÅ□/□╛□╔├1 ·Cü{`ñÆ±lÖ□╧□█□√Ö°»x╢ <□▀í5N□
CNñ+┘½£bwæ□σΣN┴□é*éÜk└

│5╞φ$, X╥□,╫░Æ`M'a=Aúå═□╣k╪⌐»╗ ÷□□ 8▄□□#8┘ h%8GpQA□╥h□╖-
áoφáK┤□d~□ΦJ╩╝□Φ┘ü\å>φ ┐ôPH0ÿ`╪5áH├h
π:
%□e□@╣åJéΩN┬téÖ□Ω4╠╙░P_ù□╘□4□¼φ□6□4u□╢h□═□Z□÷┤â²□└┴vpΣ□└qéS□└□é≤□└Ñ
vP▀9╙4 éz~t□|/╙#êk:╖ôÉG╨½□□jΦKP╨I□H0$
å°A2╧H}□C0₧á4¡«tÿñ»□¥Ç⌐□╙┌ò»m□│:□.;ç`>┴b}]vì÷\
V□¼ε□╓□l∞□6╡âmi┘;▌▀&}Ñ÷cÖ;d╩┐dεÆm²G╥F╥╟5⌐∩ñÄ÷ª╛·¡╢mJ·ôt-
É£┐zn±Üæ┤·╠CmmÜδß√ÖG N□£V>é╫ù╠│è╧}╩╝@ÉP■5╤û≡ôYa□⌐╓Ç¼□φ█╣┐d∩Y▌d╩?g⌡
Φ¡·¢╒OΘ!kÇä┐dÖ

Yâ□å□î □E0ûáÿ
⌐_¡O.ïu2╣åmN╙3╦Ö¼d≡╜,╧ùUÑ█╒~£┌ìQjMIÄSB¡ìY5║m3╥╧ƒT}┴∩5j=C▀fk^U□╟Φ□┌»╥╥;Ç5i
δk·□¢ä□i╨~}M«ù-
f¥∞*█«à=σò50m╜K·,é¼╣·╧c╛D≤╔□d▒-iÅæE}╧Z»·│╘·üy╖M═°,┌SdmV╛(k╖₧□╔yÉ⌠ïªÆ□?7<m
Ä$ö▀Byφ□»Ü[φτU╥┐$τVB╖□°-≤▌iσ+╘|╦ó}M╓□╒ε,╥u╓1φ├gΘ>Émd╡ΩrΩ∩τ□┌√±Ä≥$▙æ?NBi□\
½«≈≤°≤█A{?Öε+╫╔+>2▌□u┘╒┌7▒

╙Gg╤-.δó□ █≡x≤₧&.4Å|V£l4╬╢Ñ≈/q▓┴xWφ╟h▀□g□\½ⁿY£∞0▐τ╨₧ ₧»²┘A╜o
?□/Tkt£÷Xq¬3^D└ur]TG£δá²P╛R√╧ñ┐d?Y(»∞¢Ω«°Q╚╥2╨╞jφ/╫vαç█·α╘-F√aû┼√▒°të²N|f
Z·&▌ƒ|Ñ/∞│¿o±:═□£□E□@√╜`y□á⌡z╒╛.
i░╢□$≈hfov\╢▐□¥æW÷]Θ{¼r]÷TÜN┌═-
₧□±yW╧½°┬+{¼8φSπ⌡╨□%≤┼□ö]║╫*{J·▒σj^▒²ßJ{╪xôªif□oVÉ>▀Γ-
╩G─≈h√▄/»▐╟□─□j(RÇ╣G~+~D_Å_Öâÿ□╘∩°Ö┤·G≤*~^╤╖8Θ'¢ⁿavP¡=I@ƒÅ)=qf│ioÖ□╫▓╔□dτ
Ω~Ωⁿ┘Σ□▓╔ d╙~1╗@·ól≥┴┘╘₧l╬?R¡□

Φ/φ

▓i□ÿ═{@«┐T┘)»à┘┤≈╤&=dOU·╩ª╜]6⌡¢ƒ□▓g)=e╧Q·│⌐Å┘┤w╤^&▒□d ƒ⌠═┘╘▀∞╒□ÿ□W╢
¥╜NΘ=¢⌠É╜I┘Y÷6ÑG-╟∞□·▌.-c»≥σ┘┤o╩ª╜O6╖¥÷9┘4o│i□ôM√Ü∞ä╥oÄ¡²□⌡?'¼»R┘C

φorhOôC{Ö£▐i÷ô°÷□9┤»╓í=M╬
═╫>7ç÷49#ö|₧'9┤╖╓í╝MN±□[M=□Φ5èΘ£ë*O╬d┼C$┌N┬a`a╧□=_{@TÜ□8·⌐béT$ÜèASqgê5╙Qf
ê,S1e*ÄL┼Ä⌐¿1□)ªb─T\ÿÄ□C<àè Ræ_*µKG{⌐□/□█à□-
□╓Ñb╕T⌠ûè█RQZ*2KEc⌐8,□{Ñú«□iÑb¼Ttòè½╥□U*□EEQ⌐3╝*rJ┼L!nE┼I⌐□)□□Ñóót<öèä┬╣Y
□²ñΓ₧t─ôèuRQNêoRæM*ªIG3⌐8&u:□║RQK*^IG*⌐□%£∞UqI:"IE!!■HE-⌐ÿ#□mñ"îTlæè*R±D*
ÆHE□⌐╕!□1ñbàtöÉè□┬·^□□ñóüT□Éè□╥▒?*ΩG¥Nç╞TîÅÄεQq=*óGYêèΓAⁿÄÄ▄A╟Äè╟╤18*·FEΩ
⌐X□D┘¿°□□'┤uL
óiT□ièáQ▒3*jF┼╦ΦH□ö□Γ«èïQ□1*□FG┴
■E┼╣¿□□□█óóZt<ïèd┴╓□□ƒó"STLèèFQq(*□E┼₧¿¿□□oóbLTtëè+Q□%:ûD═□h@EÄ¿╪□□□óπA□
óc@pn▀╘▒-¿WEhΓ▄╡Ä∩Pæ□*ªCEs ÄCEp¿╪
□╡ü╢⌐H
□úíó3T\åÄ╙P▒□ê┐Pæ□:µBE[¿°□DXΦ╪□DU¿x□□Iíb(t⌠□Γ&t─äèòPæò*>□╡δÿ□□
í,M¥┤W▒□□A□∩á#□0j*║A┼5¿ê□□╦áú□T¼fçèY╨╤□*ûPE(@ÜèJP±□*□A╟
¿Φ□─□¿ê□□k□φ⌐°□□Yáb□T4üè#P□□*v@G

^@E□¿□□□□Ç╕□□□áb□T□□╝Ö:·»╬ⁿδ╙■Ω£┐≥□░7u¬_¥τW'·╒□~uz_ƒ█╟ë}uV_¥╥□Q▒å □a%ä)n0
╬□ [□π]π╝0ìï╞ƒàm°&-
□ªc║"hF╠¿□¢╥∞"óf╢Ö#<3╫ⁿñÉf□≤∩Dª┘█ⁿîê¢╧ÿ╧ê□½╚·è°äSφ<(n≡≥╝¢─'╝■▐p±≈▐7╝□qïWΘ
= ╛ΣM≤╛#n≤fzÅè;╝:∩q±Uoæ╖U▄Θ╝ßm□|╝]▐∩┼l»╒;Oφ√ ╖e/╤║┌¥á'Aé|éBé┴i╫a□E□ú
╞□ö□ö□ö□T□T□L'ÿIPG0Å`!┴Rézé□é╡□6□4□l!h&h!╪C░ƒα
┴□éπ║╬S╫╕₧╤╫≤: %!□S±¥ ü°█vJ_⌐□N£ ù O±S╫^□}U[·öD▓╧╪@é!□├
F*9╬□Uf3₧áö`ÆµW□L%ÿªΣ:╡□|□µ□╠'XL░î`□┴jéu·║1φÜ╠┐ë`¢╛«╨σ╢Ñ▌▀A░ï`/┴[□ç
□Ä^╣▓^£□□°?└5⌐ï|□□ö.?╚□:I┐Æ¥8 %πñ·¿/
°lä□╛r┼X-╤ryjHé□= ┣w╗]╣║=□zïù¼*½╞Üa═╢µZ
□K¼σ╓Jkì╡▐j┤6[█¡¥╓nkƒu└:l□|NZ¡╓9δóu╤□╢kGφL╗½▌▌εi≈▒≤φB{░=

PdÅ╞∩π(ò╪e□σvÑ]mO╖gZ¢φ:kƒ=╧^h/□╘█
÷Z{â▌do▒¢φ□{Å╜▀>H┐-
▒Å█°∞3÷y√Æc:A╟sΓN«ôτ⌠r·:□╬@gê3▄□Θîq╞;Ñ╬$°┬ÖΩL├²!N¡3╦Öπ╠w□;╦£□╬jg□`ú│╔┘╓!∞
pv9{¡%╬[:□ó╘□}ö╥ τ┤sûΦ
:%\¢┴
SÆör▄nn□╖╖█□0└□$î─□╝ì╣ä▀Ñ£êÇ

â╓oi□▌ ƒ╝âæ□└∩■'■$≡ƒN╛ ├9Qα╝

║□┤*{□Φ1(√Y┬²└└∩└&9\╢□≥Kφ■î¥ xE-╤q~╥{"ε|¢p#≥<╥⌡^□}·
┤a6°≈éV∩öε□zÇj¡╞╡└□ □╔╝ⁿ;√F┬GuÅn─▌ hòz⌡τ╤»{╨≥□ª¡âáâ╕+PΩypεC┘█└╔□}
╧>□i□h╓-└

≥□"O9ß|╨·á
∞AαO□]□
α□≈╟▌□▄╜┘■□cτ^┤d□r2▌ƒ#Ç(Å╥├|H█□iEáƒEN╞ƒ%▀╠y□V∩Γ-ï<ô!□□Σ│f&≡wó□%ⁿφ╕□
╛R¢□â╛ ⌠Má√≤╫¿□□°█t─»□□□Φ╗╣v■f
╤?□▌□·4╙ü«(╗àφ□ⁿ□ⁿ⌡□Æ≤□z±.,ü█_╧o □é┐¢ùê≤□J□q■bjó╤¡d{□<╞÷└⌠σ7ÿN╠v□└÷□8├≥▌π
î□□□²6╦□£□²g╨*╧□└²æ≤δ└ô┘B╕mùÅ¬6╗O#□□hUΩ□┌|

ⁿ₧αK╞üíΦ]?α?ó┐u╕█□□□ fΣ□å║Nâ┐□2
└Qûá8□qw□≥╧Cì╖íÑï└Åó÷[æ□6□α£·á≤A□╕╗└┐□·F╚Qⁿ^h╔x╨ƒ□}□ΣⁿO╞┴□h╪|0ê╗□└·>ñ╝╬□□
7C┬Máo□▌ƒ┐cF·□□:□8□Ñ╛ä6□á═ô0╩╧á°∩Γ.┌µ«□τnαƒ□ƒ╟O░▌□^□╝□2╖âVv■□≡ôα7ü
▐□·O░Σ_á╡s▒{ñ┘$Ü
≥<ΓFgä≤eτ'^w»└√Æ7┬√è7╥╗═□σ▌εì±ε≡╞z_⌡╞ywzπ╜»y┼▐]^ë7┴+⌡ε÷╨╜»{ô╜{╜)▐╜^╡≈áWδ=
∞═≡-±f{▀⌡╢yoz°?B╓7≥□C
QéL
]5tOú□hùg⌠╤4τ╦╫tíå┴□h╒6h╟g╨J╬τ┴î□¥╖Lτg^y┌∩ò·Z¡azZ¥3⌡∩u╘┌├öÄëôóU£CD-
πsåh≤╓w$q8⌡÷≈Lc0±±□x╩½▌«▐□∩□òt¡µq⌡╔≤□╤ □╨/▓□□°╧ü■

0√¥|}≈yα□Σ»□¡pWαEα½▓□@oÇ┤⌡└┐□τ7á□"□≤√π½X╓e4f
□□*q╙σw8τe╟j■■□q╪/≈┐ⁿ□╖Gq□Å□τ□ú□$▄ty'ró∞e╟ƒ╥╧Ç>

Ö?□}□t+ε┬7]■58
r╚°êyé□#□5<c3ÑfJ╗î□µbδêu┌v¥í╓iδ┤SD√æ"g2=Y╘RÜφ

╖N╕c)ò╕%╫Pg(ùΓ½S¡≥╥▌□*□ä╤¬D\û┴i2=╟&╤ÅMIíæ□C¡;¡bδ.kéu╖Um=-
╟╓○╙╓▓BV╧√ΣTy┐¼Æ5≥!9]~G╓╧GΣL·ÿ£-┐+δΣπ≥{r«ⁿ╛£'ƒÉ $□╙àr▒|R>%ù╨gΣ□·¼\)ƒùδΣ
r╝|QnÉ/╔ì≥□┘$_ô»╤-r½▄&▀É▌σ¢▓Yε□▌Σd□»┐WN±≈·√Σ╜└U~ú▀(-
≥▀÷▀û╙²WⁿWΣ#■r□╣|□ⁿY└|²ì■F·]α□╚|□r₧─▌gA»□~

27Ç □≡FαW □3Φ-É┃□x□≡□ ┗█üw░|a¿V≡U⌠ªd╧□HX├X|╧║╙/≤╤Σ7ⁿ=■-·M╘\

| □≡ } └Sü□«□«□■

p-≡c└u└Å□/□^□ⁿ□≡RαgP╫□αü╙□ⁿ<≡:α□Ç□ü¢Ç_□~□╕Ö▒╕YN≥±-
í╢╫√⌡r2≈Äz≡□ '╘□╞□"OΣQ?ÿ₧Γ╖·¡4NîΩ□g*≡²║gLW□?□╬4▌Kµ<ñ√╧t-≡├α╠╨²fN□≡π└
╤ÆEá□k□0²□≡Rα-
óσ⌡└╧á²+ÇƒE┘ò└?B╪□α5Φ╦sh ≤α¼□~□x=jy□°e¡9µ┐ó⌡╟⌠½á7i]2τMαf╞┬┤εñU,j=φ$od[╖s
╙6╛α□┴¬÷╫°kΣ8╤Å╥□║╙]t°;?≈□n□□▀┼ÿε≈□╜h□(□ì┬y <ì□π□pε□}≡Tα√²s■9╥<╙╒└□é3
╕□£Θ",┬ñyªkü╞▌□└ÅüS□ⁿ8≡□Σ_ê□□ü│□9ùÇ~□x)≡│╚│□°Gα4□»□τy╨δÇ_□^□9/□┐

▄□~□≡½αl□~□£7ü¢üwp½äA:úY╓□τ⌠_≤_□□²>┼ƒB┐=δ?K┌□DëF┴ ⌐ S·¡□%*!■û□ù╝▌┐]Σ╤uï┐à
┤NûBZg\φƒ÷OS■╕êô╓╣■rα{Ç∩□₧¬5═╕Zkù±wÇk╡.□╫□?

╝HkÄ±S└Kü□□▄□ⁿ<≡:α□┤V□7iM0~□╕Ö1⌡»Z█JOJ▄»ú■Q≥L¬┐ª;èW□wE0,▓Eû°(□,Jφ~ⁿ?√□■└r
"ö╕∞┐√ .rDNGYp»3?Q£sV?

╓Z┴≤¥G8%ï|╢ o■┐ü■ò ½k╡█□┬□"┘- EJ4â²╫²╫╢▀<°S≈■╒ W\□δ 6UÿWëÄ·qî□┘s□▐Wⁿ»t¬oⁿ
C~□~[╦o#½@□┤╧{U[┌╖ïl-r╪cÑ╖i╖┐;E
φµt]■q x▓╫jb║«»║≈K ù╫║╫µçg{√▓ⁿ≤□ □┌▀┌-■! P¢< ?æó ├ □\□hwy3%╓□ì7{

M ▌à╜ ç╕ ¢□ ¡

<½Éƒφ%⌐╫\J□⌡π╜·O{Ä≈δ-v□|σ▒l□Åv%m~g{%»≈^r²τi-
M}□²cJz£▐O6~ÆvÆ╠├║p'Ñ÷?□┘t╖▓b╕□▐F╓ƒⁿ?ë□ÑÄ┌≥¬ jGⁿ╘O·\N╩j╟K▌c╪Ö¢╧╙
e_┌÷▀δ°²\┼OGσ«╤«

eΩ·□┘·ö▐#Oç≈~ß óS⌡Φ╣┴▓╪□ΓÜ£□╝è╛▀Å)b⌠ä0ü□²Dδna╦2·-ß╩{Σ=┬ú°äjæ!┐-
gê□z6xBt°güÑó»\&□Dí\-
□,nÑτÇìΓï┤√o□╖╔□┤é▀E□□≤3□φæJ¼□Æ>üñ{$}"╤wS□-╒±uZ╦&╓I┬óτæoê□=ôLª║┐)╦EP~ïZÉà
□dQ
*i╜ªg□║{┐╝_äΦëÑèv.□P╦▓¿e▀&\#kê É|ê┌:¥vB&?═□G>LφÄP╗gèL·¿£EÆ-
ôÅæ□z┬□ÆƒqD□9G╬! ⌠¼C╡╨╙

⌡$^{n}$     Ωg£ ··Oä□╨>╓ö?É?á╓.ñ▌Æ-
□╓E$g▒\L5>)ƒñû,æKH┬S≥)║╗ö┤╙à┤├╧□?ö⌡╘;zZó▐╤≤□▌$^{2}$□i-□¡eæ╓^

==æ£
ñ┴0i≡□j□ôlóû┐*7Q╦_ô»Q╦7╦═TΩu·:σ▀"╖S╦Θ╣èja]¢öå@╫aΦ:□]Gíδ□t□╝J╫nÜ«□⌠$XAu░«□
rè╝ùZ└□∩□ì□á± 4-é╞□≥A9ì8¼±□4-K╚x□□□Bπ□□□Aπ□h▄â╞□⌠T9ùΣ│«□Φ║
t▌σ□║

@╫AΦ:D║^J£÷·u╙⌠□ÉkhgÜ)ƒô╧Q_╓╩╡$Öu□Ç«â╨uH╛,_&║Q6□ƒ⌡-â▐□╨{□zù╨{□z□@∩-⌠-╨zO╛
)i□φE
j=X┬□Nê4·□Yσtzû^@}YH}ατµ%ⁿΣîw□⌡□□□}i
╧□□┼□éßbñ□#╞ïR1ITê⌐╓╖AÖ▀ΣH5s

h─óÖƒcÄ°;╨ú·/□□Γ°UD╡ëÿi□≈ªbú
□!²Y╞╘┌-Σ□τêfj╖%┐(o□BÄûwP□∩ûwï□qÑ□r╙âô╝ö┴^╛Iú±-
9à┤[)∩#√╜_>@╓⌠á|□-ß;ñíçσ├"¢z·(yç╟Σlq□ìp¥°$=⌐╢⌠╘╧₧ΓSr╢\-
>Cc≥□╥öG╬f¢0╚Ç□7┌□≤¡4`▐□s«╣└\b.7WÜk╠⌡fú╣┘▄nε4w¢√╟□µa≤ÿy╥l5╧Ö□i╙╞_╪╟┤║Z▌¡₧
V+▀*┤□[├¼"k┤5ÄµHÖUnUZ╒⌐√╙¡ÖV¥5╧Zh-
╡Ω¡□k¡╡┴j▓║X═Vï╡║┌o□┤ÄX╟¡S╓□δ╝u╓6φáφ┘q;╫╬|{┘}φ□{á=─┌`□╖G┌c∞±v⌐=╓«░°┌╙∞Z{û
=╟₧o/╢Ä█╤∞□÷jj%{¥╜╤\oo▓╖┘;(τ.{»²û}╙>JwN╪°φ|╫║ªΩ╣`'□█ ;╥╓q║9=£▐N?gÇò∩

rå:#£Q,╫□δ□;□¥╔µ□gèSσ╘83╠}╬lg«│└YΓ,wV:k£⌡)²╡╫c;}&»Nú│┘ÜεlOΩ╫┘Θ∞6g;√Æzt

8ç¥c╤I°òZw╬╫ÿ║ƒ╘»╛║┬u▌¿¢Ö╥w;╝╗]▌εnO½{≥Ü¼╖╜▐Y/n7▀-t□╕├▄óö▐»íow┤;╬-
q╦Rz╫╫$?u-w+Irur\¼#.┐|KjV¬Y÷4░─╞z□4bσ
╠S±?Ç□╫k¼□╞√└─3└═└·[╨?□µ}åß 3Σ#n█X

■≈⊥G

▒q─╕ □ n wÇ□╟/~

ⁿ)Σ─√□|╒B─┴
╝≤└ 5≡D╙WoSÿì╖□□₧□╫oÇ┐□╬>α;ü □ⁿMH@─|Γδ╙·U≡□+,□1Å▌èÄ;□¬²┘α½╖Üßì□╞\`╝∩A
å▐P±╓╒αα╜
□}⌠±^ä□▐□q·Y╞ù□]-□]-≡▐□ mα2╙╖ τ□▄à«─├αá¼q□8ƒ□²=╨ùQ÷

j·?α┐□|3εn□■□0Γñ²a╚ ╬e╝±┬x□w□τφcD⌠√█ágπ
°xπézç£▒□·±ª□⌡-□□æ─xm□ ┼ÿiD□ï╟ü±> □╤τ╞1α α-
àWW∩ÑâLπU`╝1┬T╤╔ê╔6-□Vm√□╩¬7:¿□╝□t#≥½≈□t□æ▀□┌c┴è

╪ë~Cå▓^╪¡æ□îQ3`!Γ□0Ω2╘█ j└A─╣□□
²√Φúq□8?□å4÷lⁿ

£╖Ç∩□å¥α;╙åÅ7g$-D╬█┴ 9≡H`╝A─Åα.$□xúëÇEë□@/╛

■OÇ±~□±&°░□πIp□□≤■□╢%·□d┬□<╙δ⌐∞)?#√╔╧╔By│□(?/□╔□É_ò□┌¼»ú⌡·□╓2≥Θ.?W░öi∩)à·
AR╛▄ª▄└□PÄ≈$#?TΘó╘-°F╠□g╘╟▓KαV«Ñ□Q·7¡[⌠[·╧∩}l!Kki√àk╛⌠ço;╖Φt╞╙E)ò┐ÖεÉ<▓┘-
4SÜì╛ó┼(P∩ä□{îüöô╙~cñ8hî1╞Φ7K▓∞±)┘₧Q□êô¼â╔□A╝□¼f≈≤└[ü±å□±□`XÜ└Zα□7=□ƒ□₧□î
╖ h▀½∞_=¥√ÇW□+o□O%>
i╧□▀
ⁿSX»≥█░□±□0|Ü»h╡ª╝Ö▓j-▀«Φ╤╖⌡□3.kc9à·╛I{∞₧Æ<!┘╧Wi╘&╙□□yr╛£O{╥½╤□╨ò v6≡/*=Ç
v½]æ□²EσvP■|=╙j⌠£□Ñτd□s2╓A⌐8σ╠%OñJÑ▀g╝:¡g□⌐gCR╥;╫▓t□∩]╙¬δP╙¬½F8╝₧σ·n□Fi4₧
,▐»å╬jqñ-╗1z6_⌐ⁿ□∩\ñ▒┌"»Å\e°□?╘h*k□D0⌠CKa9FJí╓╙`éa╫A

K·0r□□□ ┃ΦF□εFO╗□╣║ñ□3R½«╦H¡║.#╡Ω:ìT{9□ΘH¡║n#╡¬âuú┃□ûw╕n|╩╥φ╫ì□Z~%σƒ┌╫ìçêε
ó≈;╝╙1░╙q░╙ a°□┴N╟├N'□;¥8v:┘╪Θt┼N'□;¥OΓo]▌S√¥U╪∩╝ ·╦□i╫≤y¬/C÷Æƒû╜eyúⁿ¼╝I

É ÉCΣ-rÿ,ò ╙▀+G[-
╜█░Φ╣¡₧┤⌠q╥≤╜╡·A·╨L#5¢╥□╜:²u÷b4¼u£╢╓ç:Q║│δR%,y□=≤îOΘτ├┤║││⌡N╒▐ñÉ₧□è?╓Ü
ß5□wZôW╧▐q□ì□Qr]┌}╡ⁿ±┌╗

□ÑQ
□╤â!┌√~0─┤]─╟k√ß┐+_▀╣╢¡°8e/ⁿEÇÅ▓ª□»¢UônV}l║Yò╢"╘╙Ü≡╫┐"¼
°k^□v╨ÿ≤╫+V□Σ■□≈â╪ƒ□4□ o╔ƒBéÅ{²╒╥√▀ü□5╫i
Θl▌╝r□╨∞²hW.─╧Åjσ║Γδr╔█}tu(·ô»ôm┤≈°▀╖5ò└□╪□╩D·uò╢uòè□N╪

"α □_∩□j[█x=»?₧}Ar┤>╨ÜJ╥µm□ΓK>║║>jφ¡·╪┤╖Ωc╘─╫Ñ■□ë |Θ▌[2╧¥é ╙0äf┴Z├%·□≥┐Ñ╓
∩K5ΣS□□▓?Ω□îÜnÑ║εL·╪ΣΩ:QNΣ:⌐V□+¡àò6Çò6éò6åòVbÑφéò6□+φ'░╥µbÑφåò÷o░╞µß□□}≡┐
ë╧±□□·{c□Ç½»╢╛Z□f┌²⌠r╬5d▄a╫╪3∞┘□sëZ`/▒ù█+ ╓□╡▐n┤7██ v□╡█▐g─
░□□□#Ωñ▌jƒ│/┌τ□ß╕N╘╓t║:▐
z□╒╟╓w□¥┴□├ê*rF□-GPBPµö;òN5┴t╨9ô8u╬<éà╚╣╘⌐w□□╓□╡í]╣L°╓┘B╨î□Z£═╬~éâD
□qÄ;°£3╬y√ñs╓5¥|7Φz□qûΘµ║yn/é╛D□┤òΘ

tª╗C□å╗#▌1εx╗ ╘¥DPA╘TwÜ[δ╬rk¥jóµ╕≤▌┼ε2é□εjτö╗╬▌Φn"╪µεpw╣{▌╗▄C□Gë:ß₧v╧║□□□ε
ΘÇM╡⌠

ä $·ù□╚q╣□á□ünü-ü▐□²ê□□□Σ╘□å□î□

□îr≥□c□┼ü▒÷röδ⌠om[□ÿ□ÿ□ÿBPET
╒>#0¢`«□ú└é└□□%Ñ%░2░å`╝╥gá1░┘9╪«⌠□╪□╪M░Ås□

□Ä□£d)εΩ@kα□┴En╡▌□□Aù z╡╜□3â]â▌â]φc┴₧┴>m¡G□#¬P}□¬¡-
Θ»Bò8=âσ▄j|□¬28¥█ó┐□U╟▀ür

□δ¥▓`□┴Z□■■╙╥α□|πiÅ3□_w:BÇo9æu□□╧□\b;k[«¥¥╡√-
d■_÷╛□▐ª*²□φùsεï≤║╧┘√∞}+IFÆ|JÆ$□IFF║I2╥═öJHFì$╔ÿFªd$·ùæAªQ#╜Lc

ÆdñRcî1FFªæ·ÖÆ╧¿q╬·}ƒ∩▐≈:W□╖4~]□w□₧g=√Y∩k=δY╧>k▌╡è₧ö█¥╛(â┴}Oi╣Θ
╥ZM"²;₧x╖ô▄ΓTφ-\V¼Jz□·ê|-P"∩σìO²J□√2h>Y2

0Bd0╕═Θε≡óÆI%S┐(â%╙□│kû┴Æ╣E
├╧S□σMεpZ$o%╦D>KV□5/Y]▓û~□ΣÄªJ┐Æ╣%█├╧╙»
oi┌
□ü□╚=MEφ□¥JK!╔∩ö&J¥╨«╥cÑ%T╡□L¥+°&≈Ñ□■■□□□ô;.é{*ⁿ}/■.Ä╧é∩Nß√τ▓Å&═}□┌
j·r=TπJ║q□}yúé÷;U╡□□▄Å╟╗5┤∩É╧|σ□□U░è¬±╢□âe
÷N°kª\]╒Oí/≈╔ΦSΦ╦▌□Ω:╥Σ°╗

é╡Qεc╤xGä╬},■¡z┌╓Σtg;öÉ÷╫[╣zδ▀╙arOï■4├ æÿ°αδ▄#ß∩
≥╫y²¥'□∩□│≈3h
□τX6□ƒ₧┴╜::w╣Σy│è·□9▄Gdûæ~₧|┐²█ô∩ƒ═OÄ╞█H╘─
Σ°··{è╕▓¼qwè┐╛¼│m═╙Is╒╪▀□Γ»;δ▄□cp_ì╞□Hⁿ¥!┌╓τÄ□▌▀I°&▒┐╓ⁿ□}╣≤Ggk√+╫■n"□²Z≤
≈ì░□╡▀□sτÅ┐«φ▀┌a0≈`o□≈≡□ôs?9□+α:█═Σ╬□├▀1ƒ╛∞)ô╗ttεB
v┼°7╪°√C╕n«»bH╢╣┴□YÜ□πü□¢□≈ô°w┌Φ∞≈□√▌α].:≈ ÿöpâ╗Yⁿ█EL╢¡■Ob▐─
α▀"bp▀Ä≥w^╤┌2╣j»││t▙Γ▀╟b·╗}╕½╟≡k╩Qô°<`□¡□▀ù·_
φ▀QÖ≈≈▒┤ó»┐3ç{~0é└±≈S·╗┐┤□

π∩1□(┤NI□noÿI╛┐7µ□±5╡ÅKτ▐6î2Iì#Z╦æ├]a■Ä)î2╣ëóG□Rεδx:á?□÷≈_▌─╕├─
╪δ╫▌□■~6╙□╘
■>"ìz#∩Å▓GYr□»ö┐≤m«p≥╧¬□αP□°7ßΦ╛∞q╫+|?áö□¢'╥╟▀ßú□₧╥ós□ößKê┐╫┬□ñZr▄□
╙│┤6Gô┴=H■▌A&cà⌐≈tÄ

sR Ñ╥□□
h)□≈\à╧fHε╨3⌐%é╗ò╕ π╬∞╬≡▄ù□²#¡═Qα∩Ä3¿▀x#ç□[w

W°|φ¿α-₧=9□l≥╢ëo½┬_ênUò;ón;LÑ╨Ñ□Aεôæτ≡`Ñε≡Σ□αoó┬▓Mü;□Å≤ì,¥⌠YYP▓J-
Θ╫₧5∞□╗¡VΘ□╞«\┐m≤□π╗□R□~⌠┤}J▀è;g╥╒B╘ ε▓å
_╗╬Wî┐oΘN?hΘN?─╥╒6╛∞¿ò□┐ZÉ√ê├║z╜╖-
╗▓,2Z□ö@~╙≥KS□ª6┐ê-jⁿ╨]ûòZ≥÷├ñƒ┤─□ⁿ□┘□□□Üzⁿ░µ»½"╙Ie╦££(ç┤4M\û╕L5K⌠å╠£╧╢:¥
Æ╙ó¬┤&∞├æ\╒

,╫oxyÑoçπmN□(½/   #Ωÿ$î¼CÆ0Ω höW╬X~□╧½□=\Yµ╨~₧_'·╢▓╘òπ~╘ßεmZ
╒u╧ß/├Θ□:et0╗î□J1≥0╦}W╨╨|jè┬Yπ≡öπ½╨Éö:ñ₧üu6□Å╪┐O╓Örç╒s░□G≤9□▄yuªΣ║□ª¼╛Cyò
2▀qÿ╨ⁿ═█╣ih┼Zî#f½╥8╦▀ ▌□b═ùCéDB□I¢h≥┐∞[Å╨V·zvσ□jkU[□⌐≥≤⌡┤ö⌐╢⌐mZc╤≥Ü□|□-
m½²I╒▐û╥╤nG█Ω@r⌡╛z_k"s0µ5□oúÅ╢╓□µ╗╞Z│úÆ⌡ÑfCSFcU½□╒\╡i│É?*╡µ┴┐╘ÄÄ╧ZIZa¢□╒e
╡ò╡φj╗£ö,┐Kk┼H}╗□s┤▌

nσπ
╓Ñ╒¬½àj·al▒»gÅ╢í₧Wï°½┴(ä_«f╘í□°}░wmd
δ!{¬G⌐⌡¿╒ßö▌÷^z⌐Se²⌠#«v&□□Z╤▀Çª!╞□╡Σê¬ƒQP;íÅ┤·· ±║ÄkÜ╙±>FmTKÅ¿□□Φ┐
□=╕σêΘ┴ΩZ╘ññn¿□ëGj=Cü─n

dVW∩□1□╖oÅ□╓S╞µæZS  □╫½M¬üÆ]j□ ▐╢¬eGd]⌡α|□▒;g½╣▀(½¡ª 1¬Kσ¥E}?C═⌐▓ïσ─«obë
eó▓Ñδf╓gQF□□X≡╤╚╩:3rkÆx⌐├ó*ï╜«╫╞╖╨ùT┘□╦┌¬:]-
úá6B╫⌡·°□°╥éY^╬□¼╦5:r┤┴¼#J□╟:┬┤┴¼#L□╟:Γ┤ü▀CK╨+{{Φ5■7I▌¬OM╓ÜI╓[^5ÆÄ╓δ¢^»1<
}w∩╚Z¡╓-□5╙y╧ëⁿ°ç□╖Wp╓╓□_CÄàV╫╤Q\ùΩ□□µæ□U┐Æ□)╥~$Åπi┴.jM=<î5·▓τ-□h≈┘┴oB▓g·
¢X╥╜φ√0[v!╦;□╘╚:⌠□Ω╛φ>▓¬-Z¥¡à╓▌ εò_TZ□·òw▀▐(ⁿ.□Ugδ▒∩□í|C╒═Ü□ΦÅe□=R≈╛□½k-
ô□╢¼jñ╘⌡·ä□I½┤╢┤:°e╖o□U╖-G╫·□}╤n□√¬.╫INrkåt'rⁿ─x╙╟⌐_╕kª}pS╬a╣sN╬□□hü{ (

╙]δ¼u╙zh╜╡□¡┐6P□¬
╫Fic╡±┌Dmè6M¢⌐=«═╙₧╙□≥}⌐╢B{M[ú¡╫6i∩h∩k;┤]┌─]╫ï⌡ÿ₧╓╤⌠□zc╜Ö▐Bo¡╖╙;Ω]⌠εzO╜Å█
O┐^¼m╥çΘ#⌠╤·█·╜╒áü>Iƒ¬O╫gδs⌡··≤·"}□┬«╘Wδk⌡
·f}½╛]▀⌐∩f·*╤XYNÇ₧3Lm¼Qj$

G╨h□k44Ü□═±▐Æσ□□mî÷,wPvúô╤U□q╥⌡bú□╥~╖╤╫╕╞□└▓│▄╞□)╖q½1R╨.
4aî1NΩbL0&½α|) d)├?□ç7í□■ L;I≤╓&═?□ΩDΓ□b?<OΓ1x
û□┌ôµƒ ╒ò8╧0▌□å □¼□ï⌠ÅΦ╦□n

▌1σƒèñ≤ⁿ□═O▀┐)ï'-□<ëJoO·°é≤L═┐çM;ì╛<àHgj■ =╓╖ñÖú÷□Θ□0

o⌠2$^{n}${Ñx∩Öε▀ε┼□n$^{n}$│a⌠ï□åτ-Θ$^{n}$Ov#%╪Σ 1■)Yå□■ì□N

Ke°w|═g╚▒Σ░□fÆ|₧èd≥|□ t-
c3▒□v□╧%2y*Æ▒å4∩┼╥wÉµëM·□⌠σY8┴YD<Y╨?╗╦?=K≤∩ú{Çax╨æ╫s┃î√╚τ9I┌·Σ\IÜg□·g)□lO
¥'-□■▌h<E╔α·I□OÉ≥╧|2y-ÿ╫□q

₧▄πƒ₧Ñ╥╔C■Y□2O√'qîR{∩¡╥⌐Yⁿ□
¬B▄
□JtN|'╤%qQΓ¬}┬╒»□7□╪LtL\ÉΦöΦV□╧┐=Sε□ΩÑj╛▌½2ñ▄║$g□╡)□yo┐□┤í½□°.(╓╠┐{
î7└σⁿ╙╙"╚╤D89g'ZÑIk╥ú5▌Φ□U≥?Θ□F≡ Θ><GW'╜É_6□ w]`)ΣuóÜé┘u&▄╟ß╬█Å√\α.<ê╖4pW
∞╟¡║Y╤┐∩.╧^╬√τf·≈"r┤µx□SÄτ<)JNÄτ╥□□_è2¼k─öO3□┤áïÜvµ·╜ªH□dá
h°╞□╩╬□z■┬*IΘå□±*▐%=Éá□α(9)uá□J¿P├!ãc╒x╢▌X╢₧▄^&|╣┴¼6░□±₧██□9jÅ,O┐╥Q[f⌐±▓═
╔ß
xY₧8ÉπyZYÄ¥□⌡Iûgñey N₧□K^╢?i₧σûσ⌐`╣g9ÄΣ¼-
╜□ ╠ÿ╖╘üoç½<▌σ╥╞Ü√òb=┴-l□▄æcα@í≈∩'#J┌«<□íÑE√□∩rF▐ÇÇ?D╓ì1□▐╚█□Xnσφ«·□w)╜¢
╫ù╧α^M

╖1ñ9
Σ²;╓¿!s╝A1╟│┘rMëy╩Zûz;G□₧□óTp╢@k╢Æb>c

Rc□┌á=█Θ|~ììQS½¥□0µ0²FΣùc□zQâ]y(u╖░å╪?¬u∞╬ç□√;ç□╖╤!┼╛ΦÉb_UC∞▒╡Ä▌±Éb_pH▒;□
R∞nî},O!H□─«ìî¬└□≡-ü
╡╧≈O⌡□¿≈~□E8

·¿9_"Nφ┬YU:i»;2áEπ╚I¼□é≈!□=9á□╠□êu·$R□²5

┌a┤║[▌½&A[L□5ò╗

τ≤┐[ûUΘ7 ,□ öô╤━■¥║<ú4╧╗mâ·Ü°ä□w┤≥LJ□µ╨╤~╥╥₧╠╤"═╥÷╬±──,ce█ÆC¢?w*qwbƒ_□»å╠
R╙f Ñⁿ9Kµ□╣τ¼3╦{╖ ·≥τS°7£▐╠╘□╬/:▄._Kzb╤ë□ïm╫⌡kI▒îu5â;▐║□rÜ"▒iª█êcπ≥ä▄ÑáaªÉ
π1c□ì#├ùå°╤╗┼ü═□6.1.7z□âî¢ì□Σkíôα□▐S▐|∩Θ`$φ='∩'Æ6╟e2y□róR╓┤╓ìJ╖□Y├UI╨NuPv
¬c¬ç:?╒3╒S⌡H┐£^½.K»K S]kw╖╦╒-v□√□5▄┘╬┘Nì▓o░oTw┌w┘┐Rc▄àε½Ω∩Y∩Y╡╪[Θ╜óûx□╝
╝ß┘ë□√»9Oπ3╘>□·╓xªσ°□σ≥Aε

°úΘµJ│╧╢╧V║ô╧áⁿεFwú╥╝+╝+Ç□{b□Φå▄D⌐î{═┤*ß¥╥ ╒┐ⁿÇⁿÇ▄·αiç°□₧┤x6□╧gx─¡Σ═─âp┘
┘
·╟±î╧Å' y<□fHnH~X~Xa┌·□·□UiK>√I╗▓LL√{x.╞_XòτZ ╔W╨b▀[■µ·µ5⌡@ß{a╡sæ\ñÜ-
╩MwiniU╣°□< <ì≡T╛╧ -íâ₧╬ç≤aªì°Z9└»⌠°D□mÆƒ□<ëαÖQ≡▄ÅG▄qx──
╦ùI¢J··Ä÷□ùσò┤ñ

àφ╜
Å┤╣öΙΓ╒ó/┘g5⌡Ñ┤wMi#╧Z°
\c┌█r█¬Ñ╜<╥<»≥*╫□Og<x╘═

₧┼x─¥êG▄J□|<√x■&<²≥²r#±H┌S±H}DNtUÜ:□□@Q□$¿□Ná□hH
╨□#}óΩ`O▓g¬rw□╞√≈9╥□qñ▀$Z□±1√ª╬L¥⌐┬⌐V⌐s@╖M¥
║]Ω<╨φSφA□;⌡m╨□ÉS□rΩ□|□≥╙Öƒ╬ⁿB╠/─ⁿ

Σ7 °□Σ□B«ÿ□▌ù▄□¬╪²□J`ó□+U╪{□σ╨Y

╤m≈B+^é∩QhFαA╞ Ñ%√$√□▀É□□<()£¢ÆCÇ∩HÄ□-¥□
,□QâF╝Và¼■╓□Φ┼ü╓@eB;

□}¢uƒ*▓ε╥-□₧bM□₧jM□₧a═□~▌┌ñó╓▀¡□¬-⌠h□╡:&u

≡q⌐□└╥«ë╘σ⌐üá□öÜ□ⁿHj&≡□⌐gT,⌡lj!ΦWS¢T$⌡qΩ▀╙─╖S{T$]öÄ¿"Ω┴x·≥t/╨▀K_¡J╥²╥╫â■a
z□≡O╥≈â31=□⌠ñ⌠dαç╥(U·ë⌠ô¬4=/=-
°Ö⌠3└┐O┐□,φ¢L┐¢F.Θ╧╥ƒ!d6¥□╬°≤¬╚>┼>EòPτªφ╢v[p┌┘φÇ██φü;╪Φ3╖úì>│;┘¥Ç╖╪]Ç┐k□□
°□√□Ñ█σ÷eá{█}ò┼Y"l▀êYB╖□┘âÉ≥`{0|o╢ç□╧┬╝Ql╡ƒP
√I{üè╤┐│ù¿╕²é²□°╦φW@»▓ ¼l{¥╝K□;τ8τ⌐"°ââ28□□ö┴ΘΩt□╛╪╢□°□τRα₧╬σ└W8W□_
Θ\ |òs□≡4g□RÉY$₧9)╙F┘Öï3²Ç□ÿ□
ⁿHfÜ22Åf₧R┼Ö╖3o½zÖÅ2C┬□║ïò□i{ °e≈eαòεJαUε*α7▌7U=wì╖□⌠Zw-
≡:w□≡zw=░╟U)≈-≈-σ╕∩╕∩¿□w½╖□ⁿ≈▄≈Ç σ■KY^┬│T╥δµuSE^w»;p╡W

▄├δí"^O»°*±zyΦw╫yq∩:∩:╨7x7└≈F∩F°

≥□ü#sa▄[Σ-□²ó≈"░îèä╖╨{□!W{½□≥
∩
╨k╝5á Σ²    a╓yδ@ =√□°□╜M¬╪{╫█□■ç ▌G¬╕ ╟++â=ák«╟»ZWmæ□≤□╡/7_.─3□⌠2▀τ□╧z<ò·

∩5àà▌ ╓ ╢R
K·□<ΓJ╪₧xá≤E/Và□~Éf╡░½±t═wσ£ ┤╟□ÆG}U┐¬

δiG√aí
9□U╞█ô█Sÿ╢ö□:ªÿûôóσTä□▀BÑ8nNç¥t
tÑ╚~KZHgR╥[#ÄE▌b@╖⌠ç>╣╬║╴┌µ□ΩÖ□æû□ΦÖ¢¼¢□fê5□⌠═╓P≡□`
□}ïu+Φ-
"?¥·Ö╠/─ⁿ4µ°3?ì·╓)/e_M□▓ö┼╘h:5öA□□b~E□ß&≤+ßê□qDòrDòrDòrΣÿ□□a╨n╚╗╓╗VòRRC▐□
o□,Z]▀

┘╥⌡]F7╚V·╤╩╓╫√'╝
ⁿV■¡*╝<┐╝□├å±g^▀▐ü□äY9≡ì√□∞█V□÷c▌û┐¡@&Uu·É≤
FÄƒBÉZa\î□░ÜαQò÷T¡≤e9}╦p┐·-╕╛≥¢▌ù»om≥⌡S╪₧▀^┼□( ╨>·~í╛5⌠°ùlg┐%U[╒╢*□┐<~;3▀
îOπk□c□_π□
/B╗╧╖Q□▄□╕σ□½ù┌ùbL⌡└h╥∞₧÷σ□S╜□┌@Φà░q~∩■^Θε"w□Φ┼□M║╗─]□▀□▄□0▓û║KA┐êtMª+ú²Θ
C□φ□¡¢Rké5A╒âì3□·µg╓╧@O▓&ü~□VÅiM╢&â≤ l▌z╚z□┤X@║⌡ ¼çA?b=□zÜ5
⌠╧¡ƒâ₧nMG¼G¡G┴□+╔┤~a²□⌠Lk&h╤/aZLó?OT┼┤ÅJiò╥>*Ñ÷)í}TJ□□í□TJ█°□V╧$eª-
H?□╗q2l3²`·AU/=%=□⌠CΘç└ƒ□k╚L?¥~□|▒}bΘ▌Θ▌á?K□

■□╥{□&¢╬⌐z╢▓□░fkΦ▌ 6@¢v□t╪╞╟g□┘┼á╧░[□z7A√¿ö÷Q)φúR┌GÑ┤ÅJi-
ò╥>*Ñ}Tj≈àM□° ▐L╤□*╡G┌#æ▓o□² █F⌐∞╧∞╧ □ƒ█{@gφ,Φ£-e╥█y|□+G)╙╤□] ╒s

[illegible]esT

╩µ□9Eáï¥b╨%N ΦR°-┬G£(81'□N▄IÇN:□Φöô□¥v2á╧v┌Ç>╟i½

τ\τ\╨φ£vá╧â╡□r┌;φ┴·6l.├9▀9┤X^åsü╙ ⌠à╬àá;;¥A□╟·

Φ.N□─║╚╗□□▒╬B╫w¥∩éεµ\□║▄)GÿKa⌐à£-N□≡/s.□▌╙Θ
■σ░▌BN/°□°W8╜A□╧Θ□■òN_p*£□α½¥½┴∩τ|⌠5

∞t°┐╙⌠u╬□╨/8/
ⁿK╬K└/;/□»pVÇ è≤*≡δ╬δ└o8o□┐Θⁿ□╛k□|k8□q╓□op▐□û·,╧·,╔·,╔·,I□0N□0N□0N□0N□░ê6
`£6`£6`£6`£≤_ä6`Æ6`£6`£6`£6`£╓_)¡┐RZ□Ñ£/#┤⌡ⁿ·2B╦«╘{┴[è╤.÷]=Zpª≈□∩/°Ω·╖╖□⌠
█ █á Θm┼ⁿ ·«≈¯.┬lâ5□≥▐≤▐Så╖╙√□⌠'▐'\¥PⁿU╖ò≥WüΣùKME━-ü□□ïC,
▒*`M

□{□□h□±δ≥δî&7□$öK□¿aö7╢╬é{□⌠H□ªq□t╔□δvΦ□Φ□î≥╒╓□□σ¢¡╖1╨·àêq▐+╒/⌡}î⌠▒⌐{Rπ1╓
°Ñ-M═└h□□c²9|     ²□▀A╖±²s

╛x·a┤▀Ä∩£ë°ªyJ╛b0ÄWñ╖Ñ╖° àQî▒ï±X

m□Mè╤u#╞╓:√□∞□C·█ê\π√α□æ!|□<φ,v╓áÅOB▀>□{ ùÖÑÖòÖ╖ík_B▀╛Ä▐\â^\ç █Ç╛{╦▌î^█Ω■
}╘□}╙□□y/»Å╫╫½@∩▄□
|Éw│≈□∩□·□ÄeZù■
╛S'│ò╨╪╞╝□□p╧╤û□Z¬8╒(⌡¡}ZKWÄ╓□╕╛j\¡╜╥╓□G█φ□φf¿
╧╒d╟τd5¡¬σtò▓╤V)╛M"V\à¼ñe⌐□┤ÑìÖ7cò⌐¿u£uéJZ'Zìöâ╢m¼\δTδtσYgXg¬·╓YVku
éuÄ╒V¥h╡│╬S▀▓.┤:½ô¼ï¼«Ωd½¢u▒:┼*╖·⌐S╜▌▐─╓╫╤òà╘·ó─
¼⌠═▐:áö╖ZSαF¬Ö╥é¢Tε⌐j7=≥Ä╢▐□Zo_ì:■hk□¡□½¬█□⌐N(╧]÷□w□R]ÄtW╘¿]╟╒«_▓
k╥┤πÅj┌:╨s5i▌±G╡εWhI□e>íå╥≥≈□□φ□╛┐>X)□é□0╘┬w¡u½5\E·╗d□╡□¿,öt:w □□tàiªΩ╘╡%
╣g╜ë}¬▌▄>▌næy 39≤`fè√í√æ√▒√⌐√o≈3≈?n╓=yÜ°{!»╚+÷J╜z^╘ï{
/ΘY^╧│╜îτz╟x╟z╟{'x
╜F▐╖éoï┐

ï$ⁿ⌠+╒π┐Q2¥╖MMmÇ╢Sàσ°ê┌ eì∞ε▄ê▄Él╖l╟▄╢ⁿ÷ⁿ░ⁿ÷l╖▄ê|≤ⁿ□·□╡9·&·à·╓·Q√ïƒ█⌠┼É┘Ä
┘╟s
rNN╧.BJú≥e·QH¥½ÿáσ7≥«□

╣ûk¡ò!□0Σ└∞¢╣>╣≥\□ö≈y«□O╦

╠Mò_╪≤□╕ &0

íe/└⌐ö┼L╨MòÑÄK□ƒ:A,éTπ╘I⌐ôS═R═S°Uìû₧÷σ≥╗Ö═╦~╠■eµú╠╬╠╟ÖO2ƒfve÷d▓Ö\&∩.─╪XΣ.
vù╕/╕K▌□╢N)+t□RçL"□X□╚σ8pÄO□□|BΩ□p□Ñ□â>)u□ΦôS'□7KAç ≈µ└°ÑNπjWσox□□├
·┐ßí4│α√ÿ²□≡/φ *□%█Ñ4ö)╧ °^Σ/v╟ê□)MΣ□~□dª¿□ΣΦC|i□Σ~ñè
O▓□⌡⌐√⌐*à\²[ò@╢>S:Σδ?□ôu⌠*Σµ▄£□ï¼)
╥□┬w?$NÖÉ╣R|≈╫≤Ω⌐R╚^TÑ □q%╥ùRQHá
N╞╥¿□$╤UIæFpÄ≈ÄWiæI□çT6R1Ä√□┤╝z□cFvLE⌐)EO

└ê□d
╣/□╜┼ò4]à╛8╩1▓'⌐å□┘▓?+]9nj1b+≤∩□Σ□▀A≤7¿S□uJé+╟
╛d₧■/$eU┐ÉL¿┌ƒR□╟□□¼□â£»□ï□²Vm▄WKú□╙╕□∩-□╟□▀OK╝"wç√╣╗╟═{ågzß¬□U═E╟0╓DI]≥æ
òH≥bH±ïU┐╓ΦεçΦ[#╪▀╙0híƒ╓/□h!ëu□Wî4e□%si═┤□ε□¡╘âôΣ┐Å\═□φ^τ▐ü╘∩t-
PnY║,úNe>□▌ƒ1ò╜%(L╤╗╟5■·φ°w≡8_,┼□□-
┼□ε¥_ó□□□4Ä«φÉ□σzXƒúΓÿ█j±ùƒ─τòÇz]0·╥□ ·U+ÜUn═Θ|□Å½┴┐óÇF·≥╧╓Ü$₧I╨╘p┴·
²□ε╗╥□▐M√/?╫6ä□VA╕□║╝2'╕○╓É□k╞¬α│╝j¥µ╤
╟·╜!≈┤ç°I£╧°■ÜJαwëOú¥û√½9{[æ■≈□µ▓d≈T╡ε╛δX Γs$ö┐N[α7ƒδDò⌡[₧▀"║!╣
╣T⌐Σ▓Σ*UΓ¥ß╡æUnU□ε□Ñ%□ùⁿ¥╥ô
ô
□ε≈╓▀+3╣(╣HàÆïôïU8╣$╣D□%_H╛áèôKæJ
RYåX/%_B¼σ╓σê⌡r≥e─Zæ\íJô□H■A╒K«L«TæΣ+╓W□kUÉδ□J≈Zz-íí╧⌠╬ä╢nσ╡éV>╥;
·╗╡╫Z□yg{g½ÿ╫□σô¥Ç╟}┼≥╜ÿ|□σ8P)δ▒ö□û2ZX╩Σ½╓W1w╘\╓□╥□)(k▄/½wÄw

w+Fá├

╒╥□,≤□╛Æ┌├┬║╨║F¥πΩn[uà█╬²₧Ü∞φ.K⌐gé²Ä½ò2:□□]otú½□=é≈╝á□╝□J□≥jΓW□_QEK~_□╪ç╞
╘é╕C≈ë?╜╞▓╘ª£□
╖┐▓+□□ò1╢α}|ü;qÉ┤ª□ª□φ┌φ□┌╞àq≈mπÖu¿ìkjç

√╔π±²≡□□o^□¡□;╔e╬√£x□1╧δ√l)Θ°ê»!°Oò□╫┬□⌐]¼d?9ΦⁿG□□x□-□ß╝EⁿnÉª□┐╞FΓ%J╙»
Ñ□τ"é≤ì8≥;a╟_oαk□╓Rσ∩#ìí□Nà□h@-╨□Z
¼╩▌b╖í║▄=□□a(5┬▌╘□│Eg$□'□CO▀£╝Y╓¿e□░lΓ╓ Γ-≥■¬»W╨ß▒ì□l□l
▄Ωá┼>8(]╙½ë_=ⁿ'□⌠τ{├╚fⁿ┬p±pìy╓ª<□
╥┐²¬8╛ πV┴╗╗╫ì╫»

ÆV□╝□o□┤]e√²_┤ca□÷m╟╙ΩP;╓T▀ì√╓úU═ⁿâ╞k[Iδ╖"φ#¥"]#σæ^æ╛æk"□"C"╖□□DF□╞≡}\d□▄
╓Çç□3"s"O□₧ë,□╠ë,□,τ√*ä¢□y□░

░1▓%▓-≥AΣ□└F└τæ□óJΦh8▓-□ëZñ▌h}▀□4B╪-p¢┬┐i⌠┤h+╨mú□Φ▀6┌9▓%┌-
┌#┌;Z□φ□Φ□□□□Jz8°úócIÅÅNñ;□0
a{└¥ Ö╤╠ú≤@?□]H Ñ╤□≡{-║&║-∩¢□»□▐□╝Oz□°╗ó{äÄΘ▒b║1@□a╫└-
ï«Åò┼□─□ân□kA ╓▒v╤5▒Ä▒.▒ε▒₧▒>ÇÄÇ~ÇδI□□□Xlä╨╤ª▒╤Σ▌
╕□a╗└¥□ I▒─▒Θág╟µ╥□vl>ⁿ₧Å-",□¼□¼

▌□╓□╨╤b□b¢
▌□┤║□«≡╖╟v□v╟r▒▌q□P□z{<□pΓ╟Æn□h□╤┼□╞¢≤╜%á
á}╛□▀╗é┐3^−∩□/Gⁿ╛Çk□□□σ±!Ç[π#IÅ□┤ë≈à;Ä∩□□ô□□╟g≡}N|□╥x2■

a□`    `y≡.░¬Ç^□□3╛«α]`cα□□K|[ⁿ□└'Ç-±╧π[□*■IB%┬≥₧ê$¼°╣ä
¿□h$ß□M┴?
╨*╤6╤!╤□╨
╨6╤□╨□t∩D□▀√'□┬□□□

□□□¢□□■D└ö─┤─╟─πÇyÇτ□╙□
□K□+Φ╛□▌□└·* ÖëMÇw□∩≤}Gb□▄=I=Y

ê□╥I□∩eÇ□Ç╞Γ&¢ü▀□╨║╧_O╢□t□tß{≈dO╨}Æ²j
╫c~□□□æ□
·n╨□úô≈é7   0□0□0□079?·<`□ⁿτ"▄≤ä╤°.ÿ□╗□~r5`-
`□`srk¡íOr{ì░3╣;Ö□∞┤L½Th+□┌□╛ul└o(t└▀O:V□½╣╒□╨&p█[¥¼«Ç≥└mo⌡é█
∩╜°╛o°╛╓5╓□└É╚□0□0□ ~π□o□`2▌ç¡□pτ□₧□î│₧▒□XK¼σÇUt▀┤╓┴▌□╪□X`m│>░>▒>╖>I⌐└
□"□
∩≡K╣⌐·╡éF⌐ª⌐╙□¡@╖J╡%▌!╒Ön7@□≡ZI▐⌐▐□⌐□kc¬"╒ƒ⌠└╘P║├□úR²┴¢□_+ÿÿÜÆÜ□ÿ
x□015/⌡\PûàÇÑÇτP₧□A╛»□╓□V Å⌡⌐M⌐w□∩□v□╓°vÑ÷ñ⌡t1 û╓A°àå[ûnÉn

`ÿ╕Θ□Ç╤Θ╗A▀k│Jw□t□▄¢₧öεCwjαNó□│0╙·>8ÇaΓªg□µª∩□=?²|z□`□`~z%Θ·Θ╒Θ╡Θ
Ç═ü[□[kαm□∞$╜;¥│M╗╘N╪Ññ□√╪}áa□▌─nn╥□┤□┤□4▒;æ'╨□P

Φ□Φ╥≈k╚□□`□┘□n¡óG┌c∞q□q'□&█□█3□ôφ9÷ôpf▒□╪K□╧╪╥φUp▀┤╫┘□□Γn□l│?□|b□n□α(í¥░□
ík9.x⌡φ□0ÅW□z╨□Hz□τ}╠∩Núhà╙4Z□k@X#«╠Θpwq-╟|φ£å0¡□m¥

Ç╬N7╕=H≈v*Φ÷w□┬□□□εîr╞□╞;□¥)p°YK£Ö╓Æh7τqg-Φτ£àäÑ╬□τ5╕k£⌡╬&τ□║∩╟█ß∞r÷□□:ú□
è¥⌡Ö□ φL╟ö!]ä=4╙4╬4□┤□┤

hüvÇÄÇ.□▌ =≡∩
Φ□Φ□ä╗>383,3"3□p7α▐Ç□ÿ□ÿ□ÿ-╨│□s□≤□╧□□□ßûeVfVg╓f6d6g╢□░□░Ü░3│;áàƒsM@
⌐¢╚lv□ß╕╟║
□fkfg┤¢█□╡U□∞¼y░╗µ┴□¢G{¡[t₧▄wΘ'v□·═m
~□yw█#▐<┌i»╡¥`KU─Z└╛Z□╤$v□m¼╞nW▀Ñƒ╪iΦW╖□ⁿ^≥ε÷ì╡â·┘╝╞□α

qouG□╞□╞□«!Lp'□⌠H║□□□â₧┴≈9ê7┴}╥¥ ⌡uƒ□oü╗─]

Xσ╛Θ«s7║[@
lC┘?□|éⁿ>Å5÷ö╗æ□÷"₧σ╢^²╚□»>∞[╪┬^úh□└x»⌐wÜ╫*║╞k□╨╡á;─║x□óM╜╬▒₧^╖─L»GR°N□]
▌;╨┼ócíπ⌐┐í3e|êⁿï|ïⁿz╝3;╜èΦ<»?╧0╨¥P≤w|ß□ⁿ╫≤φ«>╒σF╛O╢ΓvûÄα|kÑi]┬≈□≈Uτé3╝¿
□tè⌠╞╨ú└·▄mBçe=cnx¢°µ■N╬bΓ□ê□□ ûq[0¥FLßuå-
K▀H□E:9∩ô3ä□_ⁿ·f≤<Ñ□?╬]K□_ïF╖ⁿ#Bçn#╛Å╕éa,·╞╓□LNî⌠%ñ╓╓ε&'D╬-
└7τ:Æc□╦¬┴FSNÅ▌ÿδ─r╬□£┐ÅσëÆ├□å▓

□ªoÆ╛:9┼⌠²'Θ□▒⌠╘╞∞.≥□CNOΓ~Σ\E·N╥≤□□
╙aδÖ¢╓□êx□±OΦδ1▀π╚╡ù£cH▀D·xb?d ╙·ê4√+≈cr▐#=ë▒┌H▌ìÅ┘&ù▓¥▀fK■ÿm┬û□u
╛îm╕K|sYrz□ƒK■-ª╨ç⌠Vª│ô£∩│¥[ô≤GåG+Θªy□≡9┘rí┘¢ª┤¢nå[□╫â<∞□|»'åîΘìM)φΣ▄çéÑ_
⌠╞í╬─πêW╤7Oⁿ=rfæ╛Æ°jr╬"}□±H┴┘═Σ ûƒë╟Ñ□╚/%┐'±â─δ╔/c╚[╔yÜ⌠p╥□ÉεOⁿ□S■□·/ô≤□k
²,k²□╓ΦnΓ¥¼∩y─.j²Iα{
±°α┤4_□₧Öƒ+tΦc┴a╚Å÷╫▄vΓï╔▀"ar□╨≈AΓƒ□▀□<DZO¢Ö²îa.æXYñ¼╟CτÇû5M?5|èp┬ÅÆc
û╛╙O
φ□~ε□

≤□±c─Å□7a¼g□å┤╚3≡ë└WÜ□k┌é▄²lçKëù□□□~÷C·▐*┌&δ≈áHÜEI░Bïë Lⁿé°å»fx÷lXñΦ<·_□p
Na»ì'■î°1ª □}SL┐+·"i┼2╥uK⌡
rîÉ▐Ez*├ƒIÜ=bⁿ□xh«Ñ⌠áë□°┐J⌐¢□ΩAÄ╘σ╒▄□Γπ╓oF▄æ°MΓ□

9G░±'ªp;·gÆO·

Eë┐ELë□i⌠² ╦╨¢eH□#ûí¢□□□╔≈'-ZqHn▒╨╥bá□M\N▀¥Σ ^iªgB├□τµG□□□Aⁿ╣ö╟\$⌡
Ω%299

═ª}(□¡ë?·▓╥╬M┬

±□b¥x-▒Hf□≤□┴╥Sÿ;Äæ□r≈Æ3Å£$qöⁿ╙ü∩Æ╓╨□0ù╕(çwà-`√▀N║)±=─?&-⌐M1¢▒<□ä

¥J╝Æ╕1±bΓ□□µWµtΩ1□))æ□φ.µò□5□:≈□·SHw!=å⌠oêe□╝╦ⁿ9≤¥,t╪#}6i_¬g□ƒ─╜Nb^2·^□=î
₧zJhs□9úë □ⁿuYµ□·□sî□ éu□L|'╙Å3εtª#≈╬╕nⁿæ□ª□□²≤aVΣg¬·□┴y╤EWçDå»

□□°ì\Æx□uH□ûφ*ΩßñαlPtstΟΓ8s¼╧Æ╝$╕Φ□╓╥%τñ∩

3Σ<å⌐ τG─

9»╤╖î²δ□▀ALYb╙□%$fü7□τ√H╗ë╓╥□□nÄÆdd÷□~ÿen╟2_┴□□│╠╗H□ù□iΩMMX,µ□æg-
eó/î_·²k╬□╛╩,□M¢█E};Ästæ°□│yoΓK□□□G█π<╬å-
ô =Γ-⌠e╦╚h╥«ÆY□i^"xÅ┤╟,Sµ╟;┼n╤fàñwfQ╬+$ñ╢Üσ⌐□]$tε╗ñ E▀□⌠╜æ£å⌠╜à⌠Rñû4V3═≈ä

¥Bⁿ √]tπ9·□k┤ç≥p□·O┃T¥I»Ñ∩

í├╖ô≤2δ2ö╡√£┤A·□ª╨¥q┘V┘╙(i▀' □råÆ▌B║
S{ê£╖E╥╠Mh≤p~îHÜ·äî;πWΓ¢_$ⁿ╨│ög┘│²í)'Φ>û╗\h╤ταⁿÉú∞□ó□

j
ÖïßK=#ûÅ6EZ╧x┬l!XB□[ñ□╘G!□│C≥▓'µú░∞□·╙ö□ªäS─}Y╬≤X╬eLs-Ñt,≤φ@╬=─½ë»□
╟æ°í╞;└╫□▀F╝₧°»∞²M"Ö╣δ╙ßⁿò
1M¥£∩p╓°)Θcë_#~îavÉεI∞ç□ê°a╩¬╬ö{HÜ&σ07êv┌╟□5rª≥cµ»□ç╨_j0e⌐ï9æßoe°vlß-
2╟q─⌡╓·_Ω╛□¼¬δ╩w ;τP╣\.≈^╟àXπXBî%╘Z╞Pk╒╡

e¿Ñö□┬Xk¿Cë╧PJ)c□Bê╡öqêπ□π≡¿c¼%─a□-╡╓□π≤Y╟°·î╡╞╟│>k)M¿╡╘□b-ÑÄEÿ▀Zτ▄
□□}_Ü╬╫o□k∩▀Ykφ┐τ∞}÷Yδ▄s≈"~╥≡U=Ωε▌ƒΣ°□╟?µ▌Θ*û.`N6╟╝kÑ╜-
ñ▄#E╫φèδ√╕4Z1^╗N√╒g⌡AÄ7q
ΘKîòñ#≤╒∩ÿ≤~Äτs▌τX≤□.φ5╛Z>╧δ█Cä
¥àçT+Å□qε╒▀τyßJ┐┬φ·□╟τ9«α□%╚┐╒├4C5φ□6ì²o«²<┼c╝τ┤x.[┐π±ñ;N╡╗□╨Ü

╫≈Xτú╝.±>╨÷≤y─

P.╡^α·≥.╓∩Γ≤êÖΓ·6δt²[≥m╞%?£╝ZêΣ/╥∩⌠ô╗ε╕&>□y(≥É┌ÉRÉ≥╫Ω─
R>ùR½₧I⌐KyT=ùr.σwΩy÷vûzT

j□╡ ·╢é╫

Ωî╔+|▌á=á²||╚w□Θ _Ån,¿□t ▌├D□AùA├--±⌡$(P□╚□□╗z ⌐ 3@Θ □á╣
┘Hsp╝□öƒPÿP∞╤rÅ▄πò ½□*A+□j@uá╡ M8▐□┌ö╨□┌µÑ□
;□vü÷&□H8£p,ßd┬Θäs±□ /√µ%\H╕öp□t╒KG²╞?□□≡OMh□m≤╥

┤ä]á╝■Ö    W$^n$3²|▄□·Ö■,╨$^n$ä╙■àá\ □□□╥□╨□PÖ□5≤½└»≥»±╫□»#≥»≈/─1□δ{▓ì■═■zΦ╓#-
□3□Å╩f╜-8 ▐╠╟;PF=σ≤wüv3oΦ ≥Az□t□t□e□┐□$^n$^Φ≈□□Ç□æ□ÄëX▀ô
í╛~Φ÷#□_□⌡□Å╩&╜Düπφt£húî~╩ç6⌠ú
²ë╛─ (Æ8=1
Θl╨□╨╜─□╟_□■ó─╜─□αÑáeëÑατ1╣ ·«¼<▒□Θt╨□>«□□0Å╩&╜Zα□>nD□□£»□╘□jM▄□jO∞L∞N▄□j□
φ□□b|□$^n$□ë=î╧&÷qz-t□║φH/C~9q8a□≡□╩â<á□qÉ
□□{Θ~╞'╕

Φ□ⁿë▌ü0δªB╖;▒□·;A{╕□≡í3├MYFφ@ ▐@:°□t□ÿ□êCJey╝@v
'░8▒█┐□egâ≥Aàä□┼ü┼üσüòîW□*9¡□╒·w#Ov`-Σk□Mü
└¢□m,▀□ΦÇ¼ ╕╔K≤□/τ2Hw'Äw▒ε▐@□╥6≡□□6á╠
\□6╥ò^┌╞e□s▐□└ç·°□Ω╪└ey╝─V BPnbï┐□i
h□¿,q□≤½□;A├■zαu□âôü8≡çÖ\}W╢□·δí[Å|t∞R
°╣ñ□└< d≡±

öQO·□Θ1▐>╨AΦAz□t□t□e□┐□σ÷B┐-
x□4êⁿ√pLΣΩ╗▓!Σ∩çn?≥╤±5P?≤¿lΦí╛~╘çπD□e⌠S╛(□π□□Ep>╙É╬□═□═╟╣%■"≡□ß£□□/□-
├°□qLΣΩ╗▓r£╦□Φ□ □Wâ□ÿGeô▐□α├|▄ê2□8ƒ╟K╕□8□x□t□t)p%p5p

╝Q/╜B≥ë:
ù□Å╥qÆ±hJα£▀$□@Sôªßx&h□(ôpRV╥|N□zi□╦o╘╓σπ%-í╜ñóñ□╨èñiIe■⌐╛╜ñ╒á*_₧□j
╥ÜñzN╫yΘ□ÆO╥Y╧╜i.%mñ4i3hK╥v□∩HΩJ┌
┌□┌æt0Θ□°╟╜⌠ ╥o╘9┼╟g<Ωσ┤4É4ÿ4Σ°╢_
£
□î├╠└òá═Θ¿ù^!·ì:ëGÖùΘ□Å_&╞/╙□┐á□□□E□'e□°s║╨K│X~úN.□O*»□┤é╦K
╬□=±╥ë4∩&╝□áEî≤é□áÑ^:æû▌äW□*g\□¼

╓é□<j

6□[@¡á¡á÷`'¿;╕'╕t(ßt≡(Φä□I░□ΘY Ü`□¿□°╝G}-¥
yó□÷·╖╖<□□hw░/╤□╝□║£8=8ît$1□i^H1╧Gi□CV
▄α±ÜA-á╓ ╓▌αë─╫`□R¼▌HçQnk≡╝G}-¥ y =╪□╚pyQ□╚

^□]

,

#□ 4!m□)µeS□┼É□□>∞±<□è
·=□âR=Ü□J□eäµé$^{n}$-àA⌐-═□æ<;!5öôÉ□Z

╩□□åèC╤C+A½BòíÜP]□√í╨┌PShChS¿-┤-╘□┌□┌□┌□:□:□:╞)╤╔╨Θ╨╣╨╤í
íKí+í½í╤░ O
□┬S├╙8¥ëπÖ└│┬Öß¼≡ⁿ≡Bñ╣ß%1>Θ□□àK┬+┬eß╒ß¬≡Üp}x]x}xcxsxKx{xG╕+╝;╝Å╙â8>
□|$|<|*|&▄□ε□□ä□c|╥!□≥U╨▌.░╤╕□T□╛å5|ù□-
Φ8≡╡dæ□□═N╢▒÷φJl□¥M÷%□²k□╖ô#╕gVΓ▌V□Xò<=Ö╓╬εΣ4▄ç╝▓Æ#πe□_├}3□Ü¥£ûX-
■X-╟^√δ"µMd¢πW╪;□⌡□N□R╫ΦÆ▌g∞1┬µWl□:M╪·kû-Ñx∞
,²□┼«t4ì-□Æ-
□τ(╓ç╪JÆL▐ê╤R~å'╥ïkw~═┌╞╓½□╢d}ò∞Å□{□<┐┬°X┐Öπ α╪⌡+╝┬╥Yîk□g│ölF ç¡??&╖<╩≈1-
f□≤.□/$∞Yæ-α□*µⁿ%Kƒ`<ìc÷a░□i-
[æ÷▒□iƒσΩs_╪èΣzë-□²9╟╡"Ω%z'≤▀9║æ°∞□╧b□╨#∞ía+╣·!Y╓Uëk+'√╕h⌡∞πΣ╤,╖N■╬Åæ L□□
╜[Dφµ«□¢m╟Γ╛δ #Ω▌öíδτàg)□╧Æ□□£▀F-░B\'□Åk!═ Kûτs□T▓□ñ≤¿□▄Z¼╫╪óD╢¡'»□ôky-
┼6yL≤«╖2□□k■DD=╟╧Z\»½oô╫≤~╓|û4┼²∞ú╜ ·3"Ω▀¡□{□╖Ω^Ägrⁿ Çêz|┼⌡°D╘w+« ═╖ô²─∞»╝
·6□≤π┌y1¢Ü□yrÆ┼¬Æ═╩ò∞3«□s□╟q∞π╕î{:╠╕ÿπò□?╬±□9╬ïYε*]¢□{ï+┘[\Θzà┘g\9µû|'╟w
q╠■`÷%WN≡□W▓W╕Æ╝┬òπ■``╣□▓ƒ╕r⌠æI6┴J▓ N┤┴A≤k┬│≡╞|τ_□π╛s╫?}òπ
b▓∩<
╞}τ«Ñ≥⌡╛≤¥"µ5≈ⁿσεJ┬πo/□π÷═AÄ□8f?╖;Vlï∞□σ□¡≈pⁿ∩□□ïuÄq{\K▀Åÿs£±□╞'9/τb□h/=_
p■îπ√ÿ3Åqªê·Θ▌╖\╤πu╫j·mÄw3τ□|-
¥bM₧5<V╧┌?á□v┬¼Iv╥□ySDÑ}î±c¼╧Wà≤^«æGÿ<+α<├sßU.τEµ$▓m═┬£□□?╞1Å┐╜û·kÖ≤□□Oe╤
⌐{╓╕╫╘□╫□□╬1qâ7□╥òΓ□?□8=Té⌡
□⌡ å¿%2──y═≤*╜ n⌠□àhµz^Ñ
╛"pzó╛Iî□¥δ√φƒsLW╫│lƒ}╓▐╬╥ƒs∞zÅ¼[y╝áπ┐òçΘ&□j╢D▀`°~êφ╘?╨┐σ╪╜VΘ²ÿ╓δ ╚
1[τ·}ù

Γ &N□╟Å]

ƒ╖°X≤3╟ □╟╠w≤║xî■╖αÜ╟ Ü≡▐ä╨OÅb▄⌠√i╡□ª╕╤ì»│╟¥▐c╨EΣ□@L^▒-
Q]≥G╫╔≈╙NÑ┼▐□X4ε√'⌐Jd⌐τßf

√¬σW8»τ□e<D▐h·
Ü√·#Σ╜0azGG□äVx`┤÷·□≥ú_□Æ-
□x□c\Wz/▌}L*▌wÇ∩□^B>T▌h̄╨_=@>T▌¿≈2NàTÆ┐╙H÷w-`┐µ╙|û□╙°¿□êÅ╝

ò ª □╟╢hW░W
□ª╗ÿ.2⌐□½□ûb¼╠□╘fδ¥╘fs□╡□□m╢□╡═╩Ñ╢□úmV-
╡═║Å┌fσR█¼√¿m└⌐□S█á┐åzAm3/R█á □┼╠∩°╢Yaj¢UKm│┬╘6½û┌fv□.ç°f□╡╨□_∩Σ²□»¿₧-
é²□Σì╕╤ƒa□é9┐`Θê /┼tíD║X

∞□□é■ ╢z┴öα√"╪
┼▀9wäα▀I╗D┐8ƒxL └1M╟□εo╨▌╜□·8WP»σ□┌╤╔□M−±□ █╣~æ□╜µ-
·EΦ█┬+äH■l≥gE□□+┴-
┘ƒÆ.é)⌡)Oï╟S÷Ñ</╛╔_ª~å<"S«z4*D╝q╙I$π°▄□OΣ▌î□â~|`□₧:«□?mÆ▐╠¢╓y;φ╣ò▐□╡Q─π▄┼
gN8╬□Oπτ▀H\□εd±╣ε╪E╟∩┐b□oΦ├Σq\≥°3Ä7δo⌠·|]□E7τ▀2_I

?+ù╚bY*W╦□┘ ╫╦Mr½∞û□ΣI┘+□òPSTXMW│╘\╡@σ¬B╡Lò⌐JU½╔⌐
¬UmW;╒-uP□S=¬W]Pù╒5m┤_G⌠L¥íτΘà:_/╒+⌠*]¡δuôn╤[tçε╔√⌠a}Bƒ╤/δïzHÅÜ8□4╙L║Öcµ¢
┼ª└öÿòª┬¼1kM│┘l╢ÖN│█□0G═)s╬£7âµ¬Ñ,ƒ5╒Üa═╢▓¼□+╧*▓û[σVòUg5Z□¡6½▌Ω▓÷Zç¼π╔i½╧
□░«X#╢m□∞T;═╬┤│φE÷□╗╪.╡W█5vâ╝▐▐do╡w╪╗∞²÷□√ñ}╔ε╖/┘├Äpª8ag║3╦Öδ,pr¥BgÖSµT:╡
╬:gâ╙Ωlwv:{£â╬1°╟9/ö□pª:╙£Ö@╢πC╛□P¬3├Iw2bR¼ï÷Çôµ╞vµ□⌡A₧ßñ□]A-ÖÄaΘtÆ
C·Ps╢ôπ,v≥¥Bí▌▓└[∞Σ@N╡□¥□┌µû°¥9α═f▐t╚⌐─,g╛│□m_Γ□ü├⌐SΓ¼@?V;U¿¡╬^k79e@U□ü.g
□P╣]aW;┼@╦12m╬□á"╗─^ü=⌐▓g╗φAÅrφ┼v>vƒ╨YD⌡;≤HèQE~·□å¥C╡C:□úìⁿ@sæ{□⌡7┌2á□╙¥τ
□□íÅT6□╡╥═K2-+w

¿U8+4ûE@Φ°Sα,⌡╞ÖF┼□τ·N╓δJ.vû□y}□*w*p>KüVß£□:5@U╬□°▐Y□╘α4"?I╫:M8█¢Ç6:¢1ª□
°□g¬╓i□┌Σ┤ß□I□┌∞líû□╡:[¥v'
h¢╙a□█#@█¥□N□K;¥□_□@□╙╜ôÑ;£⌡╬v√□ù▄ε┤┌╟9o¢│┴>□┤□τ¬╚>═%ù:y,ÑzsX║□φ╧▓≈z¡Ümw
╣R\╫ìn½pì╫┼Z╒╓⌡εñ│─⌡╓c-,wδÑ│─⌡╓8u╬Z«w╗3àÑ╙iƒ▓│╝ÆÅ┌│╜■óW@(òJτÆ╖aNlΓÆ╗0K╓s□
╗╜▒┌Θ∞┬H╙8w9╗╜│@y▌│ÇVyτw□╧═σqvÑïb╥∞ÿ4╙ôv≤8S╓s·,tA>╦□τn\□6K;£░7╬]ÿ┘WX┌-;
àÿ±}|Är1√O╗²u:y£i¼÷≤8S▐]<╬(Öµè[/VÄv╗G4WÇ║▒&T╪□╜6»Σ│░├«Dn:
¥v=r╙╠½┤½qÄp□∞&{â{□∞═÷□≈,╪¡(wæ¥ç□δ∞

g╜MWvú╜├iµ│╨dou╓≥YpÑ3X┌àz°╥□k╖ú^-
JΘ░wb=├BÄ÷n╖□xå╞┌nw:□x╫UNÖ╜═Öi╤╨Uj/q"╓9áσ÷"╟o¥óÖ□Θ□δ(╨2╘[ègYe╖█¥XYw╖%còφΣ
Æ╖░Γn□Ω┤╗▒·n□┌ëæZe5#/µ.VnZ ╜╡

╚[æÇ≥h÷╥Üây_╠k£Γ⌡r Hh-□┬▄uV:½¿═┤n9╪#:╒╕¬▄╣O╫+Σ@4{æ□W£7≈q-█ùφa
╠loUí┘▌╚δs□«HwE┬╒jÅ╥□

Y□¡¼@ò╚{╤╛╟y╫╪/█□╕Σ<√î▌╦⌡µ╪'∞-w▌░□π·ûZUa∩│□r^┤╦np[e-
░□╗¡▓w█√▄V╤║□┤▐┘h□┌╪╗óEÑv.₧A⌐Σ&{ü▌┬¡┬èe╠╣m╢g┘Öεèd0╖╙╜6çφU^╔S∞□Xπδó+û⌠╦░L□
□δ¢╕Φ╘╔░□æJ╞ë?ÆÑVù█≤□□l
▓╔Æ7J∩èïoa,≡ñERc│⌠□ï▐t□σ╖▀╚I╟dÄ⌡s.Φ¿òc⌠«▌L√5~┬#~□Θ½fΓ;çm42Ä₧WñuìR₧u0□d□~╔·
"⌠7í-
R«BΩ²s50₧xL╝ò□M╦┬╙X\ú~N╙w▄K%█K═WQµ°╞≡ldε%╦«·╔XÇ┌FX╝{∞╗⌠îE┐e░ç─+ê∩1▀□τ
ΘΦWm<ßÖΓ▒LΦ□┘ÉUª╔═{=üπtzjΣ■εß/^─QOu#Y°t9█¿z°≈□□╘/≡y

)6±&□±G·−δ□² █K₧ª'?z½▄zü·≤⌡

µ□□ƒ▐Ä□ Qε╤╜ê□╙√▐Φ□ò≤9┌□╖W╬▀s£├∩├╗%
╢f±╙d□█`~á ë▀≤$K└fzg╒┤╙¢ürò╛ô·>p□□O≤Vⁿ□Y>:Φ}?⌡□·╜î⌐a■□êoeÆ╒┴╛ƒ╬□r)µ□□çFUv
Pφα▀MúJoK┌▀□√-π:╞År╓°╕d<⌡:qd9p■¦¼ì±]î╤G√τ⌠F║▌Be□ofLgⁿÉ·
òµ╒Ñ╕mh°╡n∞^~▓ =¥;-
┐·)≡½dÑ╨°Φ□□=Ω▀·îⁿä₧¡G à,□î╙Φ¬6id▌4gΘ
msëñ└─╔c{└╜ú⌠dƒ¡_Γ3≥□°;╠%α_q½╛g²3≡ôc ¥[╒╙#Ç6╪▀íw\φj▓ïÿ□z?╓~é▐b5#tàX_°╖^m?
╜⌡j>Go/;k°L╜B╫╣^C┐ú□;$╜C█JX╜F╧╛ε∩k╘o¿%b I⌡#tûM<±═S║¥«v╞╗¿%V□a\
∩Cⁿ□Y╢L□y?╟╝¼╙└o/ù╨{√║æKnª½N▀╔8ƒ╞S=c□□_τ÷öRì¬ä²33╙*,çh

┴Ie+╘#tMÆσO5█≈░-ƒ╤ñæT?$╥.b┬╧≡»ì ╟╧e«µ2□╩■£GX √⌠╛▒.ó╥⌠╟·7
Eⁿ½ó╝⌠╢┐▐╦°²Σ╧╤/ì╨/8-6┤┌⌠s▐┐á╤□├<2□N#&·╣ⁿNΩú╪─°(⌡Q40nΓ>■ö·(v2τ□⌡NUp┐□▌^P¢
⌡¥╘~─e<■dÑ▐╝╡╛çZ.N□╢ƒ
?å▌┴∩9□Ö╬ï\Aτtñ₧l<#C4√FO╙ÿÅ¥ñ┘7□æC╠┐¢84╥─1Üeα +±┘□òA□±□┐C√»4∩D□═8╔│C-µwÆK
hû!╫Å╣4≤δÿ (≤ □ù6H╜ú□°~I3

°.╞┤b□╨îôCT&≡f╞dU□4πΣùëÅ□Æ]·□Ükúφ4nc[Θ: ²-¬W|îµ┌╪]4╫╞╛Jⁿ╤╙∞ô□4¢F_%<╣Üµ┌╪j
÷$ñ╨\□#)08c▀σQmÑ╣÷√
4□óò«sî□·Rh╞╔┐αVM°□'«╤î□ëÉ¥

╝ª_ô□·¡≥│□óî▀Hó□'╩h╞╔

~╧n0 µδCtª╘_▓ªα■

$Q╔qìΣ∩zG□ì│φ≈εD└·gÄ√¡Σæ3□╧çBE≡Φ"DΣ]□▓ÄIQ¥TÖ⌠E▓⌐M°z≡□éUⁿO□_≥╛#\▄□|>╕?°?▌○
çt╚ä,■╞)}╫╘√µ.□S╕6⌠╖ⁿMß□□╡≥7à %╘╔▀□~!t$⌠²╨ë╨□B┐

]

²*⌠¢╨PΦ╖a□╓a□v┬ ?□^□■t╕*ⁿσ≡W├□□n□ [°9■╬≡íΦw╘╝»
┐û|g≥]╓3Æg&┐ƒ ππßΣUε |$W$□>╣*·ï╓□ô\ùⁿ(}²ù╛╝₧■u╟?■±tDÉ╜P

╝□
èjyF÷╩~9
□σÉ╝ª°□R·@AÅ"-MµM□Ñüf{x≥±□Å(╠≤h□hæGy-□ÇûNHù▌éJ'Q┤}╤vò{φêªQ¬□U{iφ□j≡¿╤úfÅ¿
╝□P+h+¿□╘ Ω÷°T╫-╨■ ΘíItt□□╒δ╟5┌÷□-⌡xt╓#*°╧□ƒ□»/τ╜╘+
τÉ|g▐□├°+a≈Åò□∩ƒç┼²úd[µ□□╓□≥╫

±n`3±□ »┤╟g=^▐ë÷r▐^µ/σ▀□/σ\Kiτ$≤□□╕1δⁿ
╟ù8na~ïï⌐|□r╥ß°+n╠:_aΘ□\╫1µä╕¥╜╠□/²╢Y╛wöⁿ∩ç▌╡ì±∩□ÿ□□ùs□τ╧g²|≥S@zä╜r─_δI⌐ä
Ñú#╤╡@Σêσ»[
ö░\
6}{X$D÷GÄè─╙ë╙áÿ╞Vδ□╤,R
▀Çr┼çEúh□-╪æo□φóStï=╪ï-
□°─Y╤/□─á□□╫░Σ╪╥'â2"°╥49[╬æ≤Σ□╣Hµ╔□╣T.ôÑ▓\V╙jY+□dúlû-
▓Unòφ▓Sv╥=r┐<$Å╧□▓G₧ò}≥╝╝(/╥a9óöèS~E_D▌)w!εαxù▄□πlÆxBû□ΣN─mr¢╟1r□tIC╥.╣[ε
□g□$╖áíσ□≡6â□9┤¿¼□▓

që\ü╕HΓiO╬ùx^ö╣□╧ö2S╬g╬B─3e&s≡╠,°╥╨ï\xvökd=Γ*╞%▓è╦Yìx5π¬
:⌡îδÖ┐åu╓pφU\{□╫╛Z□qL╡ùqK╧╕÷à▄□╖╡e¼_F·b□δùÉ>v□╣\;Θ/a²%▄┌\ny«╕╨±□Ä/┼
·╕D╜╠±9¬E£µ║▓□-
π°0ìÇ8ÇQ¢/≥┼*pî£#□è□r▐ì│ô╛ÿ□\╥┐▀»σ╖Uú╟)e╬,z┬"ÄuìÑ/╣╧P4□lï9ç(6±□▀Kq□ù`╖≤lö
τ╧+∞¡Γ≥Ñƒµü┤▀╫ò□{╪▄yo╚□i□ε

c\├⌡fri<╧£8╞?τ║╕^]─·╟X Uµ£b)▀à═YÄ·½╧V6π□□□╧½σ4φ┤Ö3┬□^Ñ░╟ó^∞u{è╪²*≡□╚¢=¡═4
çâI┴p≡Ä`$ÿ□LÅ$D□æP$□IÄ╨;
∩╛┘}<v»Ä▐ï□├w_≈▐K≈]║τN║π&/K■t≥≥Σ╧$»α┘/àì╒Γ]æ┤╙▌æ⌠╚=^╦ΦL□÷Zå'5æ□

□ôâ⌐┴9⌠_□┴□â%┴┐□.
~:╕<°ÖαèαC┴╥αgâ+#■HRdj$#Æ□·Pñ ≥ëHaΣôæó╙°"K#□Dè#□FJ"⌠▀-∩╣ΘÄä÷#╪ï╝-
=r┐ΘL{Ωù'⌠Θ═F√O╡┐t/ÿ%læâ;□}╣·ß╙□▄
■1▓IñG6G₧□│·╦╒à⌠╒a·eù□⌠╦.1ò~┘%f╙≈╘u┘█F·╢⌠Vâ¬@k@X1⌡:&9I╧Φ⌡oB□o! C╨fa!╡□oë±
'≈'□;¢Ñ□ûaOW-├□╪▒╣╕□╘αßFP│ç[&ßV□o□╡{╕□╘φß=á²->□:Ωß□á-∞╖□≈ü╬{ⁿï╠┐□πS□÷⌠úÿt
F▄c¡t£÷{8¼S⌡

FΘZ!╬`<Wg#╬□»W/-»Kτδ┬h]┴1╝▄+sÑ^Ñ+c╕F╫yx-¿╔├□@¢<▄□┌µß

╨N□∩□φ╒□□-
□□≤°'Ö□:╧τ⌠£½╧°eOτéw|I_╤W=<jîÖB╚□╠T─╙□╧4┃□gÄ╫□├(╟dÖ·╤║Ç□F1»@]⌠Σ)-GlcçE°1─
□╣σ
Θb╓iπ°∩╪r:7jK╒]lGα√èε□{æ-Ç╟ÿ∩0&½δ5¥C╧╓"æƒ3g≡33╜□5DoJ¿tΓ█≈É]├^H%█□E□□√
╛½φ$δâi`√┌N²□a╢□I╥7r∞,±¡^▓,É□P┐D□=@╧├zÇ0°⌐□k·Γ├K·□~úµ.Ä□M□⌡

a▓ "εúτj╒î;±ƒQ·Γ[╪□úm⌠

□Θâd▒ó╖□φ'(╢jΘ
+√-=B-°╪lí╤0'h□¼√h□└q╙■B÷Γ8Ee╞▌I}□Fπ▓°«□ö·h]á>:╧Q¡
TùòK·V.⌡┬⌐á>╞òS-
═∩╡╫≈▒òφ>┬αúÅqm╘╟╕╟)╢-ó₧ó╠╗8■5q¿°(y□╟▄SΩ▌;>╩╡╘Æ&╬V+╡É·kà╓V□WN=uµSOπεñ>╞)─
t_╥σ□aÿτ▌├Z╜ ┤(□é ╟"-r□;√y)╟R^□H9ùrN|Éƒ□6G÷`²□

+?╜□¿Rûªⁿ7╛'«├=▒É,□□╨{
_F~
‾
╔□%é▐╒╥√ ∞┘├æì(»□w╢YoR╛□5╪]E□╬æhⁿú⌡⌡v┌µ>╡Y"]╓▓N⌠?&₧τ°6╔ ╙"ΦÄ¼┬á╘╖ìΣmΘ═□Ñâ
2@sàR┘⌠L□u┤GεqÄ0j±¢P■-Σ□□*□□{xyî'é8+9□┘╒b-
«à.î÷□äQqE□╨□:Æ⌠»╪╝ó□°ñù₧m□π·VÉ┐δ(ç│á>Fτ╣ä]□╫aùIΘ□z║□[□·A]á□>┌□n#┬YP7≥□>/

ü{ZTrX
:└h□╕G0;(¼□╗9▌G╧□╕_┤▐÷}Σ╪□▄MD∞₧╥╬k⌡^┬rê<I≥░δ1b~#[NZ┘╞zë□╤╨┬╥"÷3□╥⌠%¼
σ┬=êl5ⁿ□□h.a+9Θ<┼╛(┌ù:b«\∩φK7ßj▀□·Ä╚ê|□╫ⁿ□▌ù~Æ»|·G-!-└U╘÷ªñn!□3╥╥Ñ╖
╘□┌    ┌uK}⌐÷╛!Ö7æ²!╙□┘▓π□1■Σ6N┴Z¢à╒░Jl─╒s\

αΩN├U],ùâV"^%+eì¼ôküÜ╙n□)□╒╓|─·□╞E┌Fv□╣┐¼□e ╬@╪─·38OS,»▒-
! □ó-?ΩYëga┼╡¡□ùeÑ╕î8C╬ò┘HsΣbÖ?▒Uó╟mùΦë□┼e,□{ΣJ▒□1Σb□i
□▒t1ñ¡r1µJ+╫╓ç┤ÿÄ ÑV□èZ□╫"F╝£fS

)╒ƒ#û!∩2─h3°⌐□▀r▐Eτ□═¼□ôë²├√h≈e╢□÷,□_┼J#Uå■4█□7▓òÆ╛½ujî╛&VB│[╛6F╗ö^²□èΘ K
≡□P╧·⌠⌡ƒ

▓ñ¿┴▒□ÆÅäⁿ╙╨_─·w│f5÷H½4·/╖Pφ└Uî□M;+n╒'╡U√□□ô█ô╧φΘª÷X»p╝»x⌡µ3□6zΣú□!ukY╙ⁿ$
öⁿy«δ□\╫τ╢«oP]⌠╘┬:Y╜□╟─²xû\GOƒX□╛â□Çµ■║├yε╙□ZFN□▄[e╒█F≥╢⌠≡∞)δAδ@δoC□#«Ö═oB
[n!□δñcx{î≈║╛≤?╝σë□▄√ûëRQ.*D⌡m\╧ô∩
|m¢ ╥-²/q<à▐o░>,Θ≈□ A■E≈:ùúΣ□òCl│ú▌Ä#2d│╖Cz^□>∩ƒΓπ-
D□àHIOI╟5≥á□╓í╖ì╘mΘ□□¥□⌡Ç╬□m⌡1EσfÆ╛E┐¬ó»q╛!]╛àⁿ□A├┬Fj3-ë±'≈φ]╕□╨▒δh╟«úOî╙
TÖì╡░åφπ'σ%5E═R╣¬L¡S█╒A╒½«ΘêPtsº ùΩj▌ó╖⌡
}╤─Öt│X(│▄¼4½L%ç□Sg╓Ü&╞□L%ñ.°ÉC□x¢L□πmª□╥b╬=ùCôùf≡Zâ▌%-
çcíÉK"┤╥ä!E¥z8□▄z□5Θa7»>□
╙ôj□3J╫g▌6δ²╤└¡»aT°≈Cè■Φ¡▒pV≈Ö8F□⌡VH
M▒nê□ôü╓f3╩╤
ÉµáPts╥h0·¼M╕[ù▓4]/èà╤£□□y□Aè▓tZ,öΩrH Uk▄¢═

Σ╡┃cß2zÜA□m┃!E□ ┏@4É«╔ ñτ⌐□Oz<□t-z:âQ┃:

i£εS]¬╦°)Fn╥&tMaWlö>ñ6¬ì&ÄbΣ□╓#îz□╓;\
ùUò¬╥├□½.=é▓Φ╕ZUAzT≈¿"UñOP¼6Ω>öE╟KU□K╥U□dY□t□·┘╠x¥╩étÅ╬SS⌡~5òé.╘╡W┴
╕LMà┤]╖╦½║S^Ñáτ╨□□R╣≥*²█ænöτ⌠VyÄé

bî[□⌐Y≥□ñì║Y-êà½r□R 5E-Ç┤V7`Gπ□╥àö≡%≥,ΘZ∞`bA¡F½□□ƒök!-╟

#□╕G╡î□░;!ΘΓ±á2⌐╧î7açí⌠RÖ>-T□}Nc\#╙!¥-π&ä+╘gF┼x□Qz║╕8-╘□f½
ä+π"╓╒□□â▄+□ÇO(□▒┬≤╟ä@╖9╡Ä±□Hëûëüvq¬îqƒ ╦r⌡
í□#▄┼Φ╨m▄Q°╓dl╧□>Ö╨╬i□┐□÷8²N╞\!∞φ£^U□╕úä╓fñ:°-│A~π#æÑ>▓vYÑcƒñwú°╖LÑbî⌡-
åµa·
¬- □æB·V)÷Q╥já_e█&█□thg╙LσâOûªYd'A╧░⌠Aû-α□Θì┐/╤[N÷□j│²¼╞

╔▐C|Φ□░╛b²□¬æ÷F╬;Φ-□τ□4&└O3&{▄A□O1▀5├ñÅ╜╛Φo├∞â·w\□φ▐~Fo!íUÅq╔Θα┤╤sù⌡oⁿ╛L
╜┼ú%□î▐S\┌□y¼Φ7╠ç⌐_f─ñA □δ□R ·ùⁿ«┘╖╔F□M·Zj
iΩ»╤ô¢)°╢í4£Ms▄╙┴₧┴▄M_p4°°Dα□□╗cNû53┬OÅ3╕à?bΘ□I═╖╡╡σ∩ß□ ⌐òIπ`
≤;Da⌡□ε{□≈¥ÑµEn'²□-ô┐ä╟┐Râ

}ir╨;¢_Γ1ñ7┐□8╫V■]▄V╢Z*╓q²"sio4ß·≤yææ2+σ^Q─{æÑⁿⁿ·Z╩▌8□%B8½▐6R╖ÑW
¬□╒ü╓□φ41EσfÆ╛t6╝
m║àⁿ□Am┬Bj1▐6ü▀┴╟ô√w?₧B¢Eç8(╬è+╥ç□oí\åUoú∞öçe»□╞Z8[-
V+T¡┌ä╗╥Q⌡2╓╖░╬╘yzÑ«╟Z┐K╫τ⌡¿ëα~║□╫ΘZ│┼∞1'═ÇÑ¼i╓<½╨Zm5Z█¼}VÅu
;┐│Vƒu▐║êp┘□µö□$²╓Ç5h¥B□▓«q╧□Æ
╓%k»□«─╨^H¿¼¡^╕□C╪)Xg¼^k¥□·c□╧═╓Iδ┤╡╨
╟bh□φJ¡úVü□║c¿□Æ□Vùòσà=1ä;%╜'oÑzí#åR!iA┐à□ZcH@▓╬Zo.╕┴Ü□Eµ□$5µ╝9ß□+.è

ε□V⌐Unv╖┴¬ê"│□Æ□k□÷u□¼ó(2mÉΣ¢V╙`□¼BP1!7@▓╙╘¢2Sfσü□□r□$S=%∞£≥¡iáÖä▄□ë▀J5s╟
□+

è#Σ□Hl3┼L╡|f*B□╟□░□╣è╜╥ê□┼╬d─ΣS∞□H.ÜN▌o.δ~─s(v□$╜fú>f·⌡1─S)v□$'▒s∞÷┬┼□┬¥□
╧╨ç⌠f7ÿΘQñ▒╬ÿ.]□
f≈8åd█ä╜b█8åñYτFâi□╠╕╫gÿ5:├
□┴□å$8-╨╚□□J]□□f╬8F=Y∞□∞S}f╛║H⌐□ IW╜╪C-QG╞\¼╗Gó□Ñ¥├

▄Θà
1┤□Æ≤(ú┼
ùc□;□∞E□╒□/£ëí5^i╥╝p!åûú?{Q▌"/4─╨"┌²⌐àx>qCY

=édú:¼n^8□C~HΩ╘~9Σ□╒□E╕O╥╛∞□∩

╖@╖▌=#□HèΣYyH-R□á6Bn@½½U₧▄!w¿ZP□!7@▓Ze╨
râ¬□ò□r□J¢"╥e╡

╚jäC□s@₧□╣Dûhz{°□ÑöD□$â╩╚□5$s□6p╠□ÆùσEÖª.╚4äjÄ9@╥#□╩)^╚ìí)É□T╚╚┬╞V╢‖(BP*N
£ë□îV

%»ë□╤Ç╤èaH.αΘ╤
□¡□╟▌s╝∞□δ▌ ╧Fæ ¢oσx└hì╝%□(ê¼╓/≡╗π▀□²□Γτ╞wïΩ
·□α]≥╥╪□╝¢╝ó:Ät⌠O□WH▓Mσ≥Ä⌐éⁿÇ·k$□ƒ#}∞□░S3w2τEz□╪□goµ╧ê#ƒT
╝╟í²µ□▓w5ÇQ□╫ ═╟à/·8÷÷ε÷&ê|F@╛

êÇ|   ┴⋆"""èéRDEDDKòRïh□⌐ÑêêêêêÇêHæ"□EPDñ□æbTñ╓éEEk1┌Φ▀óBε µ╖{ù╤σÆ
x□m╖¢}Ö¢¥Å73┐≈µ═∞εL╨°dì└ô─°î█╫□□╙0─²

q√└é√N╟4╓ô╧¼□└□▄n└ⁿ·M╞^ô╗□~▓╔]>cl4·9┬⌠2√.¿«╞≥b▐╟è╥∩e¡1e1╣p:fµ·□¼₧┐┴╟┌╔┼╞┌
Æ»┴F▐à╕╒ß □∞ÖQ▌<!òKî═Ñj`5â▒¿ô╞µK□«½Öª-L^>i,/▀
╞□π≥6µX'ú₧?7╡¬.4uΦ½ÅÜ\`jR□¢r╔╧QóòxW╛

J1╙≡└ÑXcε□>Uc≡s□ⁿ┤âÑ┘□|■╘°½.≡┐□|□â╧▒å╧╕□wgÜ7[□╛═╟││¼┌╟∞Rò□g±σ≡╒É/û╨¼V╕DR8
_V√□«NiεgΓΩFYⁿ?
ε▐q Σ≥4í│h□Mó┘<B▄J√ΦÉ□ë÷óƒ□%&≤°q╡╪.÷KÆÜG≥□Σh-═╧ù·r°ⁿT·TC╒Y
Γv£¬□▓>\Ñ≥═;□j□≈¡;α╬Wo⌐w13`µ[╢½□3√□≈çj¥Ö}Q;╒□|εR∩qO²
▄àΩ+ñ÷╛□═τz╡_□α╘î¢╙RlSq□□pεßk
\¬ìj□Rδês□_7├⌡-≤gf-
╢*ìs□_o┴⌡.τá1πü□╓╟□⌐½ß2<▓MÑ╢q ┼°┌╓╫B╕û¬□Æm*╡^n7°┌─W.\K╘r╓6òZ)W¢S¡ß╥ük¥Ü)┘
ªRKσ\s¬yLïßZ¿n6╧╗╘,ε
·TsÖ~□╫□εm┘ªR≈po╚'°0JnâkÄΩ═#v⌐εÉ²╟⌐ª±σâkå≥╥~Φ□█¢SMa:□«⌐¬«d¢╩╟^`εe2╙¥p▌)▀├
∞╥xu│8$

⌐[┼!9□«1≥]┴6òΩíFï}bƒ□)÷╔·pìô╤□█T¬¢Ω-
╢è¡Ωl▒U▌□╫`9Cÿ7KWèò¬□_gëòr4~⌡ù□□█T¬│ÿ¡z≡╒Z╟û□─lvσ╨K═╪M╡□ôT{╛°┐∞(&l▌){qƒ&U
□1B5ß╤/FH-F0═ù9ém*Uù╡<ƒ╨f║□.-₧3o╧¬jxf╒Z╒╞ô+π▓d-┴6ò,□Vⁿ£h¿≥ë/ä▒⌐╛áâ▒S~
zHI≤f-ÅK≈─Næg(≈▀δ·?≈┘┤9vèVå╨|▒êîM╡Äû╟Nß7Tn□╙╚╪T½Kµ_Σ

╨╜r-f_nN8▀3T.áO╚¼t2,ß▄d(k╫□4∞√⌡▓"`F╓^ƒ:█w□╛%┬~)TDΓ╨L3-
Γ~QthÑ□√□□å>8□_□═7s8t«Ö╖·fåΘ]èk¢□¢Φ│x3Θr3û□M╞îì□ÿ╣□

╚╫|╡cε║_□E?F╚3□µ^Zc▌¢┴:z3═î═ßß°:τi3W├}π@│ªÅΘ¢e□33#{□■9═[L╛ª°q┐IÆ□╠\ì╝╞╠╒░
?╛┌9╝┼╠Hÿ^ƒ╦s&▄Å└mVh╩1}-°Kµ{#╠╧Σ╕s2ªƒπ|╣&□┐lf-êL '□f6π≡2c[□┐`┬D□gfc

[ªt4╨⌠ïó╡ S▄├─è^Ç/É╚⌠╖╤□ª╞ \╖α[Ñσª«Lε∞□?r∞`╨hεFτ¢9□«aô≥╗HgîΘ;ú≤±

föyÖrM□jhⁿ)□■╣xN┐╒╠└p┘
╧╙MIE_≡│□>sL▀□°K├?=1│.\ç_!ⁿb╘ß$╘  ú"┌┘ägä⌠□▌□«□□NÿÜ╝=Lδ¢7ùµ□A□Üu|Γ∩▐╘π

▓╛eQM╦║été□╢4╒▒N┤□S}½⌐╒îCµX9ΣXm¼S)bu░Núå╓ΘVgjl¥auúªV□½'57oΘ╥╓╓╣V□ji
░╬°S¼A╓Hj├□┬»¿çs□[□□E

F

╤┤æb¥Eπ`'▄î°ìôAwÇO≤å╓@¼2┤;╬i·o ▌ε▄Nâ£╗£╗ΦBX-
â1ï4)rÉs╕⌡çZFad╝+╗6æ#□╞KÖU^)QÜA?ÿ╥H□'>Lδ÷@δ÷Dδ÷Bδ^
₧Fâ°δ╦╘mkv;∞Z(□ê╨□

δÆ□╟ç□Σ▌ □y≈±j╚} φy"╘aN╝╞$-ü.7u╡α□V_□s«h)ƒ·hoA
□T╜
Zé:ùlaⁿ0k¥id2▐Æ\□Y□£∞°≈·╖╤C

╜√ƒ°¢KùV\.╟╙ón∞▌LZ

·ƒû╬îûN¬σÄ┬╝ìÑ┘╜H44∩¡ⁿá$ß□ª»K╒¥╤~δ□¦┌»T□¦┤î╧□Θ9`╘╡Q□lÑp╫╘T╘Ü·▒╗║3╞Öα▄┬2±k
û□│┌k□τqâsús╖3ì╟□╧=Γ□ß╩π½-
τ╤â▓xñ╫Ö╧-áyá¥·▐@ªCJ╞ÄL≤AQ[Γ│╪êÉ ■□·é□╖a}ƒsïì{îΦeFof5□µ╒bj╓∩4e3∩╕+τ□τ□g
R⌐╥σ¿╥ú□□-
âFó╖â╬□u{«□^╧òGíx╕áσ╖□V╚╩╢□XM¼µV«╒╓Ωbu╡║[╝¼s¼~╓y╓@δ□δJkT─¼□^□▀ßì▒╞Z╫[π∞æ÷
ò÷(√*√□{┤ÖQt▀δ╖_╡≈π╜■╫Φ,╜╪?□}Po║□π□≈¡ΘçÄ0gN¥s-├╕□kì#□□□□J╒≡å□]/τ<Σⁿ6Y╚╣)τ
╝ò┌@┌Li▌Æ∩╝╣╘µ╗╨x⌐τ□q}_a▌k═┤ε│fY≈[s¼╣╓B¦±□

d□N

£□h□x;░+≡ù└_□□□▀□╛
|gg█
∞□φô∞╞vS╖Ö▌▄╬▒[╪-φV÷)v█─÷▓□i▀jO╢g┌│∞√φ□φ╣÷
÷z{â²ó²Æ²ì}╪Äå╬□u
u□⌡□⌡

J

σà ·ç╬□]□□□□□║8444<tE(□ª░□½pV╕ z╕ F╕ f╕ V╕ v╕ N╕ n╕ ^╪è╝□·sΣ═╚╟æO╥╓╧σ╘;í~─>≥û) 7uûÆ
`□Σ1ªT─≤Å8Åj╓5╓5▄ ·7Z7Æ@~□·┘╙»9≥3këg┘╖╪□φ_╪ô£╫=─ÅH╚≈æ#n√:╓√╓▀¡}╓□╓~δS½0≡L`
Mα┘ └┌└süτ╙π«ö┤─╚(ΓUá/Σ|□╤╣□·□Kx╙örv<rMû░G
%¼╝Ü╡║ñ□ε□-qkñJ╖D¬J°■XåR»Xå□eXåR╫£0_□╨YΣ╬║□-
6O─¼╫¡=∞fmÉ(w┴█âï¿)ñ»/ñ∩||├ffåéNm°₧c9a╟q□:ì¥╡║N«sÜ╙╓ΘΓtsz9g:y╬O£|¥K¥╤⌠
╚╙÷≥t▀□_RΘ£ÿ╖└Sü╒¿í#╤╙╘n ä┌}ⁿê█PX7X□Q+┘╓

k□Kεlδ□÷}╨zÉö⌡É5Å%z╛⌡(k╨╟¼┼T╦z╥Z╬-
▓┬ZA⌡╟-JΣ╖≥¡╡□╢∩│∩úê=█₧C┌₧g╧ú□φG∞G⌐í}╨>HìφoφC╘─.╢ï⌐yÉé5)'xB░

╡□╓
╓ú

⊥ ·⊥□u

v□vóε┴□ç:╙Öæù"□Θ╫æMæWΦ«╚;æ▌twΣ╝╙ç⌠¢╕Ä□Éáú■≤╦,ΘôRÆ║⌠êKⁿc('╖ï};}□□Z·Sn▀╝äV■
╧/|≡┌X└╞$zΓê5e+.±<δakü╡╙Zl=m¡▓₧▒╓rIµp
-e₧┐|□┘┼╟»□·éY┴Ω┴□╟uφ`□µ║~╨□□âíá□î0▀□â°□;1╧¢└+Å└□*└╪
╝□╪╞#ÿDN□Ç╙eG╟⌐╧z└Z╬╡√Hà⌐ÅDΩO-qΩu▒▐╙Φ╪□□kÄ▄dM░n╢na╣□T╙·▄·╞·╬)ÄêHVñzñFñ□ñ¼
ù0wyî&û║║°êr¥W╩□Yh-
▒-╖ûZOX╤¼°¼g╝>l┐Uh□Y_Z_Y□□□□□╕20:pm`Lα║└⌡üqüf□&□~-╕9≡h`aα▒└Γ└Æ└πΦφ^□¼≈,ÿ╝
ü≈□xV╟ß@q ╨ûL╟éii╖|O╖;█]∞«vwX1╫0╢╞╪π╪è╣
6î▒`-▓╢□┌Å┘O█½î
□¼□¼[╨èqmÿÿ□36t}h\Φå╨°╨╧B┐□M▐□║;4-tOh□[61ïªN8□╬

7□ƒ□n□n□>9▄.▄>|j87|Z°îpƒp^╕o°¼≡9ß≤├□ç/ □
_□--╛2|]xl°·≡╕≡ì\δ╒╣Å∞═φƒ□□L■└□ε═□s□·□╦╫.√#╨E»┘□╜µ(î■OL─êÿ□î, _k□ù╓2▓≤à≤¡
s╙ëF(ó"╒"5K╖ç≈mΦæ╖G-╧f?æφu│□É┘S╨╞NV≤d⌡░æ╒°

êφσx49╒     ╢      ╢
₧□╞Mí□xⁿ┬cû
ëé□□/&□□╧πù,î_¬ACäáô╠╠K▐q╧]`╡,Srw≈ù°ÿ╓0≤rfe&╟∩╘7½/▒ƒ┤Nu╟╪┘□[°□q═¼R]o«╩╟▄^
╞6ªÖWù÷Z{#ç W≤┴nvg║Jà▓║8°a╬░□µ»L▌τ2┐
¿+┬¢¥µD}âÅj╠╤PÄτgn▄ÿ9╓Θ╚τ┌áY
J2□□α╚AÄn∩╛╥─╖╟∞^5┃□ªSα╝Θ╝σ╝φ¡┼╒

|òäTn-^□≤╡B□çÑ2⌐>□□æeRσz=)╪□▀Y╛A>gù≤WF≤√╬╟T/E>O°╙G!&!f□b₧P&╧U±Æ□√ô2w Pr7ÿ
φσ┌□¡π▌_ìz□□□σ|█▒╝₧□╛□6æ┘√╟╞≈═J□]e3σex⌠qi□«├÷@⌐╦σ3qlö╘g

□5áUì6²MαPts└╜üÖσñ▒ªé4å▒╒πΩ≥┼Pts□■
²²/ú╗í¥ìnN╘╠∩@3 =I3╗c-|¡°}bì√î²,F·┼Fδ▓ì┤+°O╢µjç£P¢PWO
ƒ□╜{yΦ:O≈Nü╢╜74?⌠ç╨Pts╨ù□δ¼c╧`}ze°╤≡□ßUß╡ß┐ç □■&ⁿ¡sE□t<□»╙·╓╒╓½╨□╗ÿ╡_╣-
|┴┘∞╝ΩlAlâ╪□□□σµúVBÄà ┘N□¡sÿ√îßí□ 2Ä□□i-
ñ╡>╗⌡p°Ä╙├□î▄o*□ε╣Tßéú╦ä{>ezπ┴g+Å╟f□╣«£É∞╦¡⌠6≈£□═,Mèÿ/ö├│Ö╒6□α÷Gδ}□¼à□ß÷@
*m╙ªóî┼□╝□zôk▌▐b┐âÿ¡╙╞ö&&4\,Nexvc╝j∩J□c}╣Ñ¼æX?qMZ:÷årk╗:╠▌□╪╟6╓aF<┼√à□q□╜
□╧)ïC╞┬Öτ□nJFc5≤j╙┼∞K□╓e]Fq3F0π╫[╣»K□éK╗LE⌐╣½ól!≥╕hò└┼╞rδ8ïÑ¿yáúW╙&^δäx/°
nO≡ε«□ì╕)╩╜)%°<╬□Ü\{░N2·¿┤.2║εjh╗□c·Ä⌡Æ╤JF'╜
¥⌠!δñbh!úüî÷1║gupMÆ■ΘεΘ¥qq}│!T`t

⌠Jr9Sq+¡±α╡"╓I.⌡+□û┌□╙║)Zhs╢ÿ÷1₧?□|gS□╛S┼Æ^nè¡┘åßKSΣ⌡j╡yUçⁿ∞6≥├╥s■□┼⌡▒D░<
@µÆ·L□KÜz⌐É╧-GÑ═ÆS·c╣≥₧¼-
Rò╖ⁿ╪╒□uàWΩ╬É╙X▄¡)Γû=3╓[HΩö6vr₧n▄╪┌╬yx"R:Äñj)lµ╫¥≈yΣ░╧┘G┌·╨·ä▓╓lh╥
σ╒ê¡┘Σ⌡@&╥Σ□k>»c=│à╢S□φª=⌠!}J_╨╫têy0+`I╛╖1N╫±)▌Ux≡=·±óε□Hµ╓~σ⌐iì9±╜e░çî╖'
v
±÷□┴N#▐ε"ε^"╪E─█3□╗àx√ä`ç□wo□w?□o'Éä▌?▄}?Jφ°ßε⌡ßε∩ü¥═▄══▄█<░wG▓┼±°r╡wr╙mσ
⌠□e%·╡è4Äu/╤π7òÄσΩW□°└N╒gÑèπó╜□╤M-╢□□▓█ xiÑss{-
□□U6/⌠Å¬▄╛q{□5!¼Ö)z∩T1Jz□aÑΩ□R┼quád-
≥]Ñ·èò╛yÑ≤□□□ƒlσÑ╬a┤ùCç□6W·9─¼╤ΣR╝^!bJJ▐1M£j▒⌡öâú├Å
ε┬ö²o¬╕"M ö:╬Φ8╧Æk!u°½┴U0e╝UÉâ≈î▓4G;*QèΣ╓ⁿsExA.╓8N□ú─ª□ü╛GÿKy#â¥σû╛fl⌠□_±
┌¡ì"Qù∩·1Å#y£~|▐╙2½ü÷pj8'Pµ│.²─╞6╤9N└ ╤╣N─9æ╬wNr□╤`°ë╙äå8-
¥6t▒╙╬9òå9□£Ätàs║╙Ö~Ωtu╬á+═Zßtò╙█ΘC╫8}¥│ΦZτ□g(]ç╣à[#┼║□▌«kδ□═0≤a±□A□┼╓ m╬
²Σαφ03┐σD□G.è

ë\□□□╣,2,2<22reΣ¬╚╒æk#c"╫E╞F╞E╞Gnî▄□╣%2□k{¢9ö»Φ+ß'w╠²┐:¼ü║3ufΩ╨¡#≥□f□·╞□A
□TL%w5Q□□µIZ%τt+√┤>rXƒPa[ö}╧╤□|3û!·úib\≥Å□□□±Éêïéc╞â▒┘┐"!v·║1?9□╞▐ÖV╞₧A`+
░5□╪□□l□□₧ □₧□□÷□□√□ü□Ç└A@α┼@αP ≡R ≡□ p$□8□□╝□□□
□Ä□□╟□üπÇ┘█Ç└;ü└√■
d∞!6Çg{╫<╛□z 3u-⌡■»H≡[¥α~.├·┼«□·┌£≡{[┬ ¥IW¼▄
□▄╟ó

□»─:╪Uàσ«∞⌡▐Q≡▓/ε₧#ε□╙°£!fïy|.□K┼□÷[╚╫j±£xQl□█╪╡P∞d ]╕≈-√╗√ú╜□█!M╝(kKK:bà
$╛√£l□;9ⁿ□▓Öl┼┤╜∞$╗╔▐bå<□╗[┤╟▐□#8G□√j9╓|»═>7q□╞5QNûw≥╜{╪=╙╕8⌡Kσ□v╧ùï°ε2╣R
╝'╫╙ë∞┐Nnd▀år

ç¥/τ3ƒ+ÿ>╟┌m□■r

▀rÜ3ÿ╙å╠∩vY└ε▌|φ┴·3ⁿï▌□ó╨∞k!V╥»9╓!│o□≤╥ⁿ╔N¬«ÿª19□z╝∞Ä╡ú@°SÑ÷¬Ñ2√╙b╜hC╫âb□
T▒╥P│6.╗▒2-╓é#ë]s±u-
I<G□K8²▌µ.S7¥□p»å√fP╜s"±ⁿ□▀╪ΓK[vK°∩4□║⌐Ö/¥9nO▄╜□>cß╞□ⁿf□□ ╓°/å□▀}¢╡<ÿ╬0╘W
╘Å0□(╛nv·1½É0□

:╙Σ%>EÄnYzü«□▌□·)ΓN1<{Ñ-i╕5⌡├▒P□□▀v)╝█»:éΓδ'≤mr⌠Yeµ□sMYóc╟Q┬║□$▀6w═

½ö+ùßnu°▀ê0╖áW□├t□▐piSA╧¥g⌡╡╬·^²≈▒
┌p9jâï->ß□â│□ΓjEπh□_ôh□█ÿô°£N│°ÜK
╪□     6k-╟óUöÅ∩ë6▒□εá╖░é■√┤ƒc-αpE∞o╓□:╚!ï±«4₧O'φ□
Y6½╥3φ

j╩█□▀┐╫s}░□□Ö}╝°«yï╛□$^{n}$[x■°Üσ0□ê□í):□t□B

A:¢ß3□□o` }èΓ9R┤?(╛`).□¡
·□╣s╕èzß
æjl┼£jua+úìsÜ▒+±-C╚█cΩrδJ╝╧`y√I]Σ\Jª,╖r)═╖ïw$╕°▒M:┴½▀ΘL=9ïδ╫╕□p
╖>╥Q├«{=jy□Ω9□▌╘≈□∞Wα·□q]╟┬□'Φ╙°cÜ▓°□≈M Wƒ□½ÿ}ª╟⌠_q>╓x°╖·ª«°□|□
MS£/□├ Vª]îE_<╠┤'╟Ñÿ.a≈└ÿF,■7}□╙ù∞┐&ªτè□╔□
¥╤&□÷/√&ü┘QWX6┼₧(ƒ□□Ä£ⁿ□Y1▀íR∩□╝Yào□TåπΣw5

□K┐½!(\÷    z╔\Jl□╒ñgæF├;^Üo¢öΩ▀n▐·¿ q²(Åë·F╫úHdmd-5ë┐φ¡╝ïhWÜ≡Æ├┘
          φ≤N┌⌠M-
╨□∩╪4@î▌ishα┼□^î┐ñëíÉ╢□╟σΩ▌┤\╡áÆ≈□■ZN8NC;ej⌠=ñ=□qv╘▀╧q║E≥8N~$▀K;1⌠▀□zD%C∩
AΦƒV□║tkφM╦KΘ≡∩°σªt°┐°ß'□
√╥≡ô□■â4ⁿ$ç 0m²$úφú┤5ö□c□┌:JÄ±q┌ZJF⌠'iδ)9╞?╥╓TrîO╙pUVj■Öå½▓1

ñß¬lî╧╥║á□ì¿°jΦ╜┼Wÿ╢
╦╞·<m+ûì≤E┌vlΦ┼Æ±8 J█Æeπ□Ñm╥▓q╛L█Ü
π░bo?~ò╢═╦╞·┐┤-Z6╬╫ix≤yu∞╞rq≡∩4╝Ñès0
o⌐Γ|S!oR□p,Æ-T□èPMJyDWE

;3÷{G⌐╗≡/ε~x□█£╖$·¢;╤╗KçKⁿ□▌æ□
≈Gò╩xTÖ╝J²*╟°│{ò÷)═OÖXg$°é/╩K□AsK·σûß□Gqw╢ùΘ≡┴≥╣Lê⌡r) █ÆR»Φ(¢╖Yσ¼Ñ╬╓
⌠I║▒nó¢Θµ:G╖╓mu«εáO╫¥uW▌Mw╫=u-
¥°╧╥gδ~·\▌_ƒ°□Φ≤⌡@}ü-ñ/╘C⌡e·□=BÅ╘WΘk⌡Xm▐Lm┴⌐f│=╨@│µτ╘O"┼94f┌D7!-
τ╘î▓8╖µTìs╠íΩ£kkªmu[÷╧╔╢□ªâε@5ÿï╙⌐&s╥Ö∩v╒]⌐□s╘ìN`«║s╙₧║'╒fε·P□µ0ÅΩ2ùgQ=µ⌠
l╬ï╣%?≤█ƒ▌τΘ≤¿>≤=Ç,µ²|÷□¿□RÇ╦p□°3H□bƒ
⌡àdsyår╚╦⌠e□Σr]┴■#⌠□□q·F2'WΘ½°ε╡·Z□sY╟≥▌q\bç╦\C]ñ.Γ2□n$╕Q║»ε╦%1<eΘaz□ùv╕-
╬%Ö¿'R
Æm╛2≥╘v|√E4öz°i┐πzD7G□─▌k··ê»╜▐∩╫óOÜ▀∞┌□▌□□5-
â┘ÅóQ╤Åó█x▄I╤u|-α≤ìΦ"ù½Φ6╛÷p▐opê=╤=^▀█°≈□╤5x{8C<p¬»z<□x╣□╕%Ä╛µ²┌╦|∞ìEë╛hj
ä∩u╩

╤□«.ê~¥Ö⌠╛□'□╤²^ÖÖFGÿ╢Gy'─C╝□wmµZ┘k■¢x□παúΦ╜╤Ñⁿ ·ΦJ≡Pïq╕□h<┼═ò»█úo└╜ù⌡≈ΓΦ
□■┐è□╗X═□□e⌡reÄ∞╟Σ^rx▓g$ÆK□╜▐≤▌□]Σ□╚ö□T─├êΦd⌠⌡½P∩anàu@I╛□Γ·w±▓7·[ûƒ
n
e, ·Θ├□ÅâÑ}_ò2`t╘□É╫▌╔╗Γx8╞╟Q╧E&9p¡╡U└áΘ/₧4v□≈□½° åΦ╒±p¢!1□╕╒□±ò∩iì72╚╔öΦ

ªKìªb╥.╨m|

<┤èç╙≈t╘G╟═║L╩□□ïnT╝6ÿ□□≤Y6╫□%É□â□S/q~□âù╡╘'3,p╜w«≡~□╡]□├G╔├ÿΦÿR┐?r¡□╕≈╞9
□XN∞

Γ!!╒□Θ├□¢â{┼ÿ\□⌠τq√»â ╨xêÆ╤┌ƒ9╘«╟δW╞║┴Σå╟ªz□·Wª<)╞f□Σ
ôUw□k║Ω≡□│i+□·éc╚╓⌡ΦV╡┌░°K╓é∩@□╢Åç(æï¡¼G═╝╞π□╤}LY=□ìÅτ] σπåLσW

□;b÷dU□▀G.2╒□Θ·¼╧□□□:.■;A╓*#wÖ░σó
Aç$·ε.δJyñ¢╟Jƒ┐╤nsαlùα╗□9wé▐δ├}┐]a□Ö¿çìáτ□e⌠∩]□╚}]·0╟£çìÖH%□G⌠□k╙4╣∩□□ù&╪
▒σßÑ∩┼Cf≡É<b«P□Å═ß╔fò╢□5□□□Ü+<║├¥==j□ò□-6éV⌐l║G⌠⌠úîÿ□╣¿≡8N2Γ╡┼1□
Ñσa<Φ%±▀
#òL═□ñσaJ□≈╥Å£2ú'wûsúVÑóg□ôì2Æ╩≈:<{≥ßö≈╥?□9÷▓Y╣÷°■çyJ_╒²┼F╨■G□=3r1>
ΘwU<□kä£Å╢┐╚αQ┼6L{≡≡fⁿ≈□í33O

<L-m[|╧.:!‖

≤∞¢y¼U7║#z ║8║è╟Ö¢ú√

2╤[┤úv∞ƒ▒gíσ□ì└╧/Äq.òßaRU▓α⌡□G;╫|∞√ïJ□Ö{ª□φ□w╢½
╪1:╝╢□ƒ≥▐qΩ╕╤╡úÄπßΘ¿□U╩├*╨≈¬Æ□≈pQQ╡╟Q█0Ö╓}a┌≤P┘├{Z╕'·ü█╧W┼ô#≈99▐*¬▓7ó<-v┼
Fí╟┌-≡□═╝Φ█▐»?Vσ¢□╤5Σ╜□X%xp1╣π°═□ñα□σÄ.ï╖¬ó-▄£
¬εy.≥?&oL□A■▒'ÿ¡*

v¼╢└╝v⌠┌╥3▄╤1Uⁿ«┌≡-
└╖□□Å⌠╣ ▒9R=s?╬ç$[▀¬o#╥╥δ_ôO▀Ñ∩íΩz‖■□ ╒╤≈ΩYTO╧╓□Q@?¼□PD/╘Å╤ëzë▐A'Θ7⌠;╘O┐½▀
Ñ
⌠Gz?
╥ƒΘ╧h░■B□╤E┘*█Gùd╫╓«Cùfç││├tYvπ∞╞4,╗ivS□₧▌"╗□]╬y□τ╝o%í□Ñ□ERO╓ôIΘ█ÿ□-
ss;eΘ;⌠□TM Ü9½╬£▌E5⌠T=òjΩ╗⌡▌TKO╙╙Φ□²□²□¬¡∩a╛δ0▀╙⌐«■□s_Å╣┐ùⁿzª₧I⌡⌡}·>▓⌠,.O
Ç╤3¢C>áαÆ╧╤s(¿╘□RH╧╒s)¼Γ╥:\┌ç╣┤≤⌡|╥·□²□eδ□\■□\■à\■╟╕□□r-
,ßZx\?Nì⌠R╝ö□δ'⌠□╘D/╙╦¿⌐~R?I═⌠r╝£Üδ°⌠SöúWΦ□t▓■╝■═╡╨+⌡Jj⌐ƒ╓OS+╝J»óS⌠⌠-
¿╡^¡WS□²î~å┌Ω5z
╡╙╧Ωg⌐╝╬╫·t¬^½╫R«~N?╟)?»ƒ°

z¥^G□│
·□:M»╫δ⌐ô ▌á7╨Θ·E²"u╓/Θù¿ï ▐¿7RW²▓~Ö╬╨¢⌠&Ωª_╤»Pw╜Yoª-·U²*⌡╘[⌠□ΩÑ ¿╖╥Ö·O·O╘[
o╙█¿Å~M┐Fyz╖ ▐╬9╛«_°╛z□╖²Y▄÷o╨Otü.á│⌡¢·M:G┐Ñ▀b4╝¡▀ªs⌡.╜ï·δw□τ□□□╤·z?úd
Pr□úΣ
F╠┐⌠┐ΦB]─ê□

—£ —\□—

□b.□b.□bΩ0b.p┐jPù¿K□1□=□Φ±□=Y@O5=EOa─□

╒□åj□C╡⌠¥·NFîARm ⌐

╨S□Φ⌐°□½□╤ê1□¬□⌠X@O□Φ▒⌡² ·~╞èAO□╕ □7

□□□b4□□├╨ú·Q╞èALC µ$╜X/f¼□▄4□nÜ□1MüÆf@Isá$□(9□(i□ö4□JZ□%9@I+áñ)≡╤□°8□°h
|┤□2Z□□müî□ ú%#π%v□L┤□&┌□□°□□╣@C[áß□F├□a[□□□üëª└D

0╤□ÿ8
ÿ╚□&:□
-üå□áßt┤}g²7²7Fß>╜ÅQ°▒■ÿQ°ë■äQh□╨□□Φí┐╥_1□□δ├╘+╗Vv-
:ô╠ù·M░&ûY╗T·√°╧%≥□≡□áÜ■+ⁿWÉ┘°└ƒU3½&√Ä≡Å ßΘ┐èñ □ □¬µ-
δ┐₧j·o≡▀H╡²?≤ îΩ·□ε □·²┐⌠ Æé■_·oúÉ v □□±▀Θ┐ô▓²S²╙¿ü w■9tÆ□₧□
╡⌠/⌡┐L□Θ□VÉ~Æ ù·-Æ.Θ²ê□\╝⌡[▄╒[dC│RKl¥□oò□│F╦╒r¼╝ δ▒tÆw╩{ΣL╧□

VbY&W╨5XüeïY[Eε÷╓U·B~φ«úóΩ*[i╒Hσ¿╓*WuV=T₧Ω°□¬!jÿ□⌐F½qjéÜñª¿⌐j*°╨ëcLW│╘\╡@
-Q╥╒*ò»╓°ánj¢╘V╡C╝Ñ▐e┐≈╒~u@□⌐â¬╪τS²|╡|~_╚╫└╫D|ó≥}-
|m}□}]}╝|gyσM,ub┘A}²}â|ù╕⌡α╗▄7╨7╞-
5≤5─7▐w│∩V▀□₧┐[□á╛i╛□╛┘^¥$╘îo₧£╓a]èö□kF«π□□·û·V°V{5SªN|╧·^⌠m÷j□╘⌡±Φ6▀N▀.»
╞╡ⁿ╘τ«/C¼}░ö╝╥P□à√m╨<°╝□≈bP|Ñ.ª├ì∩á┼o@▒ÆÖ|□εOAG┴τs╨╥ß│□t□h□CΘn╕≈é^□ƒ¡αG
╓x╨]á}□µ%╨□□≥*C±□S╙■≡□□⌠4°d├}□h/ñY□■7├τ
╕ □:□>∩"╡τ

-6│HΓ≡ú≡ ;Φ@Σ╕
ß□ü^□ ÅαPts
è⌠┼φp╫D°├H╙═+┤9┬ e□Ç╖╜æú╥≤□▄²
Φàα□5áåüvG□≡&sαs□▄ô□■+╕□
≈╖á□□µ°≡▀ ┌□>ƒ┴╜□n┤╕tπÄ□▌□z□Rx
a~□≈√p?□Z□aª□Ωδ□ƒ╟@²≡G¥╥□AQ□±:▄ □□Tê=pWâ√τ¿
αD╘â?┌BPts□ƒW@□ì0╖á╞PK╥m□
╖ï╧6□·□8Aï╥°A[┬□╕òh□±□Φ`ñP□_⌠O─Ω'Æ╡≥OÄï-6|»└50σ*╪█K╓┴□R╘□uà-
┤h$rDkæ+:ï-"O⌠≤N[

□C─01Ævï╤∞□╟-
┐□)Ü47èI┤ELíφb¬ÿ╬ ?ñ□┼,N¢S□s·£ä╘□ê%£‖I{╣X┼⌐≈`‖JΣs.δc:┬ç╢Q;@τâ□@]£}é·}□□r«
ÇB⌡τ□ëUÉaàû≤!M⌡ië╘╢¡«Ç□u_ë□⌐°KñE]ih╓H╕!╖╛Ná>°■
≈Ñá╡@'├ »p╧□¥□ƒ┐ü□"□Φ;u□Φ¢%h≤d□zD¡□àû±u□à∞⌐Q-å╟¬□7╙¡µδn□ πé ╙*╦Ç₧-
÷Θí'z┌□áªJΣlï'mµ┐æ╕-,s[J╦£╣gΣ

í=·à╨êσå▀╚w8=╤ê╔ë□■┌ÜÑ╟ôLû╡╬X»₧╙□²X*□r ß«lo╥-□O╖.K¡╣lNkw¼ªΦ)PΦ4z□□²□ì□à÷╞
WΩéûúf]▌□□"h?¼Γ$╕¼åε□E/FΦ√Φz╨àáÅân□=□⌐AW╙□á«ε╜□nα¥₧□=□!]7z□╝⌡□k▌╡¿s╙║∞π┐Æ
[s4╖σ╧²7√±/≡/µVYµ╟Jo╘êc4ó□j=W

σ▒█∞□ƒ╟Wn╝6 sä╛òzéB╛▌^□o∩
║╥╥█∞α>ëª▒[·¢√╗·╧<.°b╥╟╪□|□;aá8[

□â┼Ñbä╕Zî□7ëëb▓╕S▄#f≥□s╧□≤π┐□ëebÑX□ ub#ⁿ╖êφó@∞□{─çΓS±à°Z□ÆR╓ÉuÑìp□Oj┘Hµ╙╓
°¥xq□Ö+;╟≤JL▀─3qbⁿ┼xî±□ï├ù∞!≤

/▓ƒ□h°┼∩Xÿ□▀^┌rê□f.≡╥▌π□╝'≤¥|1Oαï╙JÉâ"╨BHC□h!dó□┤□ÆQ□Z□·(□-
äö□ü□≈5p┐∞╤B╙M□h!ñ°□┤□2T□Z□I*□-ä<□ü□B¬è@
![EáàÉ░"╨B╙Y□h!ñ¡□┤□2W□Z□╔+□-äⁿ□ü□≈

πfÖ+ä┤□ü□B┌è@
╥╪,î⌠Ü═┬□0à╒┬Ü░çw╓à²Γ┘-l╡└faKe□[(□a⌐╠b█ä¡□╓¬sa│,°O┼*╓┴·|N@Ωδ┼&▒5n□┼╬□Γ-±n

C□c□┘ □z

6àÇ╝ `²╩SJ4¬└₧h«UΘZ╫□V╖k½
╫Ü□□┌
>¡ßåì*├≡·7(╞□b□Φ√╕δÄÿáuσv╕]√╓□M
□w▄$▌Q@W╨□áε¿j?h₧Wè□öó□Ñ`*O⌡╧R□╫╒@┐Çn□WyÑ0!#□ëÜ□▀x▄□ ï╜▓¢□

z‖╘ ¼=÷!t□Væ.7I6├òVƒ
⌠J-oΦ¡─&╨Ä╚²□╕?Ka+t¿2[ß┐□▀ⁿ╚≡]É□▀╬□α;╤RΘÉ KE└-□□E╗;□
w6ñ□≥┼,å8ασnv╞Φ□KÑ₧?╟□▓┐à┐ò □□; ┌■Ä■3ⁿ=ⁿ=²╝²├ⁿ├²╚+Σ╠î╚.9Ω+}ë~□:□t!¿;├╥□%┌
≈■□}□δæ.⌠_÷Cδæ*?èf_╚+KkΓ(z□KΩTû{#╣S!▒s╝Q⌠□>°⌡~±Qtg-G□=░□πΘ<æ-
ùXîx%∞zë·

ßÄB▌Ö$╫Γ╛ ⌠
°╕≤p░⌠y,n▄ìαv├cd+_E`FM<
┤ì─êAb₧├¥ò!w

»□¿;JçÄP⌡ßây8
█_AV}≡WCßÅ□,àæ╜□□Ñ0s#▌╤√ƒ□$▀ò├ò≡□oεLí─J¿╨NÉ·₧ qΦ±░├xID╧TF╧tFÅ╤·╙ü₧□
-zû≡9□⌐□ñ─╨ô□⌠ÿ╛`=τ▓)å-o>░□Ω

┃ï╥¥}┴£ärg║╞áßñδs□h{*Θ□áÖΣm≡║╘▄á:╖□=≥

°╕ ²@□(fn▌Ö=□=¢ß□ïH¥□èYuU    Ä□⌠ä□>□¿R▌@1╢UΦI|┐Ç□sq□êTτ#à□áSαπ÷l@¬äσα╬
+
L╣║s9c□dα□H¬,Æ╨□╧òi╟¢I╖%▄╣µ¥%(æ□ßâyaw-╞EÆ┬|½┬ⁿ╡;√°□□É□½╬¢g√E□Æ-,AÆï<-
,□5□n¼ô¿

ö┤½□Θ√á?|W├:R}
╠∩·Ç□▀□≡□

ö√╠αc╕½ù
╥EÆ║%□I▒□|ìß■□┤┐ç$│&∩t¼╛_█▀╠▀╥▀╓□Ü┐ô t□g□□□W□≈πå.3█▀╓□]?-□║ÿ8□φ±OP╠φ║²á┬<
ôka╕Vá½ß□P"á½Σl╨'K⌠ç½
ú@â7≈□╦R╛ÅXÿßò┼hc╖ƒZ
□£IqφΦ╬ⁿ*<╗É□»▄⌠±╘KaåL╡□uq≤Q ·╒?@a={Ol0s-'┬°
xÇ╓öx□Γ÷π□OB╘4P╠o╓g□⌠Yl□╓╞▀+
□KÅ├îuù╥v]

,╗╓lq

î█!«M∩÷□x·'▄'0ÿ
ö3αvƒ╤╗#äc╠Åj□Ω+⌐%W╣và½Θ=äá□q╟0╟Ä-
_{P╪?>⌠╗╛□%Ü┼τ>eBφ)╪?j╗╟Å□Q╠┼╧╙VÆeƒδ∩□δ╛[)√■xh□3B;Éæ+â#c╓□₧~ßæ±□-□O2Vv╔╪ÿ
5╠,hû%<2^╬í═□⌐qH=-
cπΣæ±V▒├]╫ƒΓ}ïrφNW▀c═g≈·⌐k∩z║á.|0n÷₧ló╧qƒå+<5≥¼□`╬□∩û▓┴╨╦╔wJ$╠}║*]ΘrƒΣB╦x
}Θ*P╖τtæö]óAñ½A╞ùh□µ┐□ⁿ□Ç□ª*∞òó
f│qYè╪ì±▒<╫π▀ä─3\╧┌▐δ±Y□╙M□π∞ñÖûπ≤□⌠G÷ⁿ¬╪<╟öîÅ,z╣xÆ{Fç≡╣.═YÖ0µ▄QEτÿ ¥8+╙

╔mV╒<╟╬L╖ ⁿ□gRX╔□⌡δ▀[□&æuòu□Iδzk"÷WφM┴@^`0⌡

í┴÷½÷[tæ╜╥■Φ █{

¿(Æª{6□ûΦ&f□$JÉ4K□L□╚*A@DQæ£$H□0□"┴ê□□F└ä-*
fPPP1#"rxè□□□D┼Ç□÷∩□□9□πw~≈■≈>·MmwUwMçΩ¬«¥Y□xq∞8÷ éπ╚Ö

ó8>□░äπ╠ □K9▒£╜

=ü^□╡║X!V□Nb□╪□p□½├Ω╙¿▀≥p╫□0□x□▒□A ┐□d|-Qª□╙ù□@F∞ì`╬ç≈Ö□9√C▐□
@╨□□⌐□□╬#ó┌□u□B╘éÉΦ□╘z(□3ùxƒäxùç□▓@>╠+é□µ∩1∞√□░□┌>MD□Q${²□|@|ßτhΦc|ΦIêµ╟\
_zLB"å□à⌐┬4$╞O╞àAÿå░D⌠╫□╬┐□á1╜Ö~Ç╬îf.ä#^├L□é╠+╟kpfΩÖ≈ü□□│□□□□[□ù5ìσ

0û;╥□H│û▒Γü□+Ö╡□á¼═¼-
`0+ôò□dX╣¼}Ç┬*d□□T╓%╓%╕▓7Y7□â┌╞jâ·vV;`░□┘é@£=ÿ=□H░]╪.`□█¥φ

ÿ∞Hv$`▒□▓□□6;æ¥□8∞□6∞□;ù¥
d┘y∞<□ï╪E@ä]┬.ü≡,√□□┬nd7□9v3╗□┬≈∞≈░~□╗□□rFpF└U▓τL□≥ñ╠╚9□p┼Φ£y£yÇ═⌐ß╘└:M£&
□▀q▐□A«(W□┬æ▄╤□┌rm!£╬¥

ß,εl□╖p╖@X└─Ç≡$≈$äU▄*□·\>ÉÉRòR□ÆR╢R╢□nû┌

ΦRÖRÖ@P*G*□┬R⌐R□+ñ* ╝/□g□-BïÇ<Zåû□i⌠,z□áΦ□(G□h%Z ⌐Uh□─Wú╒□_â╓□□┤□¡□T┤

¡âu■D □□Φ□⌠

ÉD∩í≈ ª□m□l⌠1·□╙ó-h
P└l0□ Ç┘av□├∞1{ à9bÄ@□s┬£ t╞£ü86□¢ □a|▒┘Çâ═┴µ□9╠□≤ü¡ⁿ0?X'□
Ç0□
ä5τa≤`=0,

╓î└"Ç▄»æv□□úí$≈H╖ #LÜ0í≡Ωæ÷Sä£□!□╡4╓□²?i ƒ┤ ≤╥^JJ√k─□BεÅIu□╡δ\╖q╖A╫₧«=▌G°·
ⁿ╓ⁿ□]╘n╤εN>à°∩å╨Bª≥òß'⌠ô:3╖▄╗Ω║«w¢┬║÷░ª□▀┌≤rWCwF≈╢εj≥?╪kwù≥%·½>±φ

ü⌠┐±%ε▄╟ù□äá⌠{Cïδ╦ä÷ù9ù□□≈@(3□0ÿ≤í⌡□#-Ü$ö¢╣Ç  Wt□□5AÆ▄1□╕c╝□□╢$lílOàφτ┬|
3□µâÖ┴░N□ΣHà□     □□□·R!_h)ÖæÉ;╡╫^·p|a▌□▐□!∩A∞JYÄ□G□╥x□]ê²Mj□□E[╤V¿□^ó/ü
┌å╢□!⌠
·□0╨wΦ; î╛G▀□□┌üv└:]h□áú▌h7\G□ú□□úct é  bBÇå10□áb"ÿ□``bÿ□`a□ÿ□`bÆ□□«,
c□1îâq ^□ô□Æ□èíp▌□c2□#ç╔□6ªÇ)□
L□SåR1□□□8░╟vñ$□A_û□ä■╛┌Σë°□╬:q-lε· òⁿ9Σ╧à□½╓C'k□t□ê□+□g□╬,£W8°≡□Γ├
Ç3┤□▐ì±=k□[⌠╠»□╠□╗-![□≤+F╬)BÄƒJÄƒBÄ_É□?ò□?ò□?ì□?¥□?ò□┐□9~□9~□9~┴∩□┐
6)u┌0à□π∩▐NJ,1~Rj{Φd═≡ ùπ∩9Q¬□φ╧Oöê/□²□&j∩E▄ƒJî
÷╙÷⌠°ç╚╫x└y
·□□□┤□-□a√░}H

öm2*,□m┼ê▀□I┬2üÖ□□ü°í▌─n└╨>┌-
É□╙¡□i₧╨▓Ä■¼□q□□□▌P□□DòPet(¬é¬÷α╔Q■□Å≡úAO╝≥C□áG╤π@í□ÅdB<≈┐□O¢êπnî.ES┴T2Æí
□╔0êîΘND½à□□L"#mr╛7**╓╒3εnπƒiE╞ⁿⁿ±Vr?╒j⌡O┤B~¬□àîJ·πs╕²┐╫è∩≥├¡át≡Wô□[□╕□□π
&┌}○

]22/╤n□□╙⌡╟┌□s□≥Sú;ⁿ□ú√Y╔ó@}⌠3½²π#√╣}╓3√·D{2┌φtµ

2╨¡□:Ñù'□gù□·╣└ò▒∩¥A9R□⌠x□=²%ó¥÷─8%8zÆ=Θ+#│╗┌■R █u║»,D■&Ñ□₧
û⌡πN`\>╟└≤₧i?

ö−[└□îJüPtsÿ┘□╪□ê╖F□áPts┤`╫B┼Pcöêëm

²¥u╟□╠▌╨╫╣╟╝╧|╓|┼l#ⁿ□û7+É□─ÜG°7¼╡¼□Φ╧□▒èY5¼[¼:╓=╓}V□δ=½□z*KíWRH°□□qÄ□τ
τ□⌠+Ü8∩á/ür╡á/1å≡-╕□▄ ε□εZΦC□@▀íè{ù█å║□Γ9U╞Çù1ê □□±--
□K8èvΓ□≤≈≤≤çh╥┘ ¢┐ @■*╚·#4B·□sGα╖

Ç'4ä□□□| ╩£/R□ÆBφ├⌡¼¢□□7@}≥

O«–

*

xQü8`≈F┌▌@╝1F ¥x+ô╪Qö□‖y□E0z□■□<!□╝ç

x□$^{2}$ $^{2}$ ╚╫ö╬ΦπÑ

î□α□=à ▐₧╧□xQzƒX⌡∩g□}╒7g
ä□╧±²╣□áè÷¢ü ä?æz·╙≥Im%D■□q└□F□·╡_áDÆQß□ó\&OZ□P(╬|╔□(_-□⌠┼╤3(±,ô□ôΘÇ□¥\
□«1⌠»αÑ▄□%û□qä□┬äëX/╤╛YuªG²
wòí½⌡Z┌üñù└G}□□Φ
°─ü±Dt·□w~■Çs □«>âJ<┘□·_≥«(¿□⌠«äP□`□√!

,ê≤□░BY(□Léε│,░EσPyαê*óè└ UG╡└4T□σüÖ¿-¬□\QC╘□╕í#╤Q└□Äc,≡B'á&└□5C═ü-
jàN□□ñ▀╣□·m

□+−*═□kα□i(é┬⌡Ç■]□\πòΦo@ë⌠Θ4ß□□Jq∩u□^□╝ƒ ╘u╡≈|‖−
««_■▌?x»╟≡jεW~╒∩│²│δπX+·σ□┼╕√_²╟▌²/Ä√;/Γ¥áφ
ò╤ù╟ß)ª□ª3á□\□╒á

┬{α1ëk□»@;ä▌ á□í!

D□ß"20)"j0G\┌ê>2□□çÿ#ôa╥å╔₧ä┌ê32□±Dⁿæ□R├|Φ;□□≥i□ ╧≤□□6I╖□Å╪B(AΣ·/A-
<╗╖U□ {■#├)▓~□Q□i$ □Ω

■□$□┬½=y□^Ñhô°ïd~äw(⌐□∞¡IΣ»"¥$╖

Æ:Ö─\$1ìd¥╔$7┬û□é□ä⌐$$1╘□d═xΓ▄éx□τ□□AΓ╫r□≥WßT6Yô╟S₧ô░Æä
ñf#f □!l ░∩⌡Φ╛╫□╞║á□▌≤=╒q□05L□Z□Γ]Ö╬□µ"~Dé□²≤ⁿ□q≥R□■0àÉo▄°├ò≈□IP?ªüM
∞□√α╡□□□╟á□öâï□Vé□(□5α□hä÷Γ5ⁿ$╥□≡□─╗□□Qñ╟╨□2uçö»'╝2EHÑ□▀□B¼;à╚ôPëä∞▐ⁿ□▓╬□
▓╬e▓╬e□Oµ·idM"╧µπ$åÇ□ ┘▌»╓·□□Σ]□Y≡ ╧□⌠Γ╓sZ╖/
5ë▌╨E╛SC≥□|w□Γα∩>1Yε\╘»L□ƒ╞╡ëî£■d□«║□f
0∞(v□₧¼H
█}σïT╪·├┼ƒn²M▐□]_ß-□□;T║Ω╛Râ□[CW╩□ó┬ô⌠w1▀á-
ⁿ□¥Φ□┼/í□cù·z╧┌Å}â■∞δ⌠N╟o╨□ □╜╗Γ□⌠□ !╜é□≥╒■═·□=⌡+⌠_╖▓ -
┌┐V□ë?äL─'É%▐ïεì□#¢Ä╟╓■& <,┴2ß¥□"H╓èôìç¿□□é≡Dpa□║å8ò"M□╕¢□CC□-6Γ

)□─╥□ƒék÷├╚l□#C>╩□
lü;□□┴`.≡□ß≡2&□«╨Å□ì╜â║8≈ªπ!°

╡║Æ
ú≥r<ª8)/╬èC°Γq┤2<ÄÜ¢EÑ □□ï°=τóbå#□╥~ídç╧ßb}╝EΦ░_æd7⌐Si□,╨T□-
D□äXîina╛~A>ß┴A<I\£@□▓□φ╝<□ââ<yCp□□├`q¼²<Bâ├é╝├σMéCCéC▌┬²`
□\ÄáSYΦ'║ú_áùûC╕[`ê╝¥╔x|êö□o8>□7Σ□□□□Φ═ÇEú~E<╢≡ù⌠L

□!Φ",Ü╡¡¥=O□┌S□□dΓ□Γδ□*oΩ`&oµ`3╥▄@╫HK╧╨╨P╦h╝ßp▐P\⌐gD2□Ä╚┴+t╛ƒç□-ç(÷ƒaä

¿qê□Çx□%

A└~□Ñ┴{.&⌐▒ç?,≤¥%□»□1>æ╢g╥^}╨£∞²µG□b√v▌□37k╟█&╙□6¢-
▄y$Ck²█┴JIo°□>┘<═⌐╤·╥vâπÅ▄.·░)Rªφ╓□ï,-
╞ZÉw)▒tóτ□úS≈Wi<+K╨;óQ*¥ ^uô□-bT_╠*Å╡6qN╞╝ç≈╤éÅªî┤x
)Æ□Ü4së▓ëx═∩9□·Iu√"S-▌ùX⌠¢Té╥j∞╞·yτv╜═╖╙╠£qyF>r>-
«□ΘαP╝ÜâNI□¡D·║□│W□«□╪<σ}/(≡µ╜¼ë╖∩ªm[░°□«w)2L╟V⌡├îGφ/e¢─io□╠å░□ùzn╕]y£o~╒
┐$LÄBà√hG□"

gäÄ╤┬)ò□°qi∞ΩÆ╖║·I<ë╟X┌Kπ□▐□□èä0)C▓J4□τ╞░ò⌠█ ░7□a┤îδÿ▀Q¿æ_fP(ü;□□Σh╓°$▄*╦
"╤,┴─7<<dñÄÄGΦ\φ└Åδñφ□□¿□□αG`uBBâ=#<┬├t·ûæXEr□íTj├*╕│Ç□▄ÿt║
é╨&π□q╥Åe£Æ0║≈□æææ□▌└+⌠+£├q□╤▀í4Q£±æ%UΦ│
I%ñ$├□ⁿ┘║├reâ▌□ƒ4σ╥α╡°╞╒Å╪¡i¥¼╢g║▒.├ rτL)Z□n[┼□▌╛ⁿε╨3┤æB∩l□É┬╗A&^6≈╞h¢à¿G
T┘·┘rú□».4n┼÷Y□□ê╨╡Wªºº╘Z╓5Üvñ╕q°╧┘Rá1u}ª²╠╙Ñ╕¬α≤Ü╔¬╤àe∩&□êa╓;xg ╝!¡╕ZUX□
£ß╒mû2+"Vÿl¡Uw<┤╟p.{[E╘▄ú╪∩ëQ;

=O!⌐═w╞-
u□$ΘÿFƒQ╖┤Pm□s¢~▄J□╡9åÆ/}ñ½π┬n╫δv╘δφx8╬@í╪╨E╫7°R¡F#Γµ▒.=Θ±|□·ö╝≈∩fv╓╟ûΘ/9
4σ╬`╣f√µ□x£□□╒╪╙~j¼ⁿir√éX╕°|Rìò≈ƒ5□¿╞ûⁿ□eíå½⌠lz╣■tO/y□?ƒ
╙òPd≥‖8ÅGj3C▄ê╟╙┼a╥∩╤ffèx°/Θ_/¥·□·7╡Q╥èc╨eék7┼Ds:Uµtå&i~x╜#=iâ·╤□ù\ôuFΩi□
Y□⌡a╤^╣8Σ≡éK╥┼╘ïµMg7╛δá╔╛Z╬α+□e┐≥□sV□}ñ&≈åû6▐ú·ß ╬¬□╓&â╗F!Ä┴úÜ≈¢
πV°O¡┼7è^Ü□ß]╪znΣ⌡òEiτàû╦╖

┘c≡r ▌Ö{ß`╥è¬?╫5╒Du»■░□N╥ÿô╟σ

╕°ù£ì/H9Pô°q├▒├áε╩╜╘╟C°═≤□.-□Ü-
~Orèe⌡KPa9yçá┴úΘb]ï╢T<₧±p·¢ÜM□rkv7─K¥«╣ÿ)ï£∩▓╟aÑΩÑ+XΩ╢ƒQ▐

₧r╕╕,H▌%╢╒((ª¡¿Ö%╥⌠Q□┼└□Y╘únå□Ωª╧2O□B·v*╡ƒ║║Tπ-□m╫êg|ƒ33½*èrÅû▒2p{é<ê□u╤N
▄∞sKúÅδ□E:KCW□╟y║□-F╕╛╗üù¢û■□w}-}]=#-#╝ß║Z₧F□<o7]]□}oÅ┐¿@╥ ╧Gv⌠□q┐K□□*-

▄s1é▓■╦*p@
□□□FjA(.PÄí□C□&Σ╫ò□Z╕ í□nD¬@╖~*p*

O+²Tá┘7o≡Q
~σ□ß╕(╤q□é≡i□□|╢¥⌐q□□□pσnO;cWíd╗}J╘¡û÷«+'oû╛|?╪⌐┼í┬╧é~│ⁿR≤â╬ì.δ]□□⌐ò╥═X≈6
E'□{τ▐.jóLU::F)j|αü÷ù`F┌╞□2ùà╫WnÆ1┼≈εΓ₧?aß≥FC□eµZg├2□Ö<┼ïÆWjπ$≈□╝8áX▒Vyw∞
╧zUÖ□o┘dcm■4¬⌡ΘáeY║Mç□u∞£f □pVU╚z□
□}X│@Eb╪□│□▌e╞□î°YE*%w□H₧_±Hê3σ¼╞

₧╦□
{v&□lP
~Y~α┘I3⌐╦ε6▒ç□Ñ-╓d∞□,
R=╫«*W╤"┐WñασUæMi□ⁿ╖·-
╦-~+P╛{·M~┘▒⌠ß┬▌c╪°3╪{K□.╖╞¥╬¥¬lé-╢\-òP·╛jδX∞□v≥ô╒Ö╛╨I╛ú÷₧Å▒Qy"ñ0┘úk╦o□k╜
├NsloM<n┤å»}°└u°I└à¿k□E□kù═M

²²┘«Ä╟;╥5#:=/□□
=Z┤¼`□±Ä□
»mp┌╣└·□╙┬╜Jí╡st9OΣ¥Ä▒τ.├α9vcÅÜª╪fë¼<╡─·φyƒD╖██2╨+V]□╢╕_¬¥╓R≡6-
□l÷╖┌≤t├ⁿèôBσ▌ú▐□□3□8Φt
½.zôv1QµUî?b{dplXß
□┼▒#¥╤·ñτ>σV9:□

]9fve⌠╛Θ:┘Γuó≤πî[╤k╡▓iö5û∩[∩P«Q╖C#
□i@kÅ□`╕q}{□ε╙|~äu%╒)C8U%·╖WÜ₧□╞ÑBiΣa╕╘_É┬}┬□┼PúGo*□╥¢÷┴┴PyB╤⌡≤÷≤p
≈Æ□ε□□Ω□-M(w▄□╫╟⌡x║□z°□¿▄uydQ□'è ▐□·[·=3{nA²m╤╘aï□┤▒√'-
<<╗qèÆ▌■½wP□eëτ╫s«O▐Ä╤□j□╝Θ╕₧cò6xBΩüîÖ╕J□□h\x▓9YPΓ¥8-
πE≥e╣Kz╧ë[_╜÷æ╤∞\°$I÷┘□¢□┘°ò□.«■`vM╕rv^e■□┌÷≈╗τ■µsKφOsçⁿä╨Gjµ┌¬√□l°┌ï6P5;
ⁿSR≡á─╢Θ°╓□Kj╥
□□╥ù┤W▒┌äÄ:□┌2K╔┤□□-
╝□⌐¬{∩Io╕!□;q√√°£A□lß╕╠°û⌐Q▌╙&Y;íσ@□7o9zW╔╝¿\╥13oH╘x^Σσ═⌡úû²ûφF9,+V╨·n≤AΣ
¬Γ$G■{z┘□yæÅ·=□╬H

.╤°qΦ8□~⌠╙τ□₧.    ⌡-+AúA·K└%□ä{m□□!0□Å═Φ╤═▒)x∞Ω□╢°╛╕9π£T╙
eu

╗╧pX?╝ag╢╟N╖_.₧qÆ╤√╣┘□┃vφƒ□µⁿZÉÑφà█⌡□□+□┌í,ô¼±      c┐ \▄G

àw$T9i□□√□□K▄□7φg□î~ΣLLî├ñçδw₧çß\Kª»(¢I5□~τΘí²æ╖»FO▒F□┤├τ╡□è▓r»₧Z╕÷ÿv5s√¬
@≈c╙(ùlΣYv□∩,□≈`ZQ₧≤&Ö√▓H┬╛ó¿W++¢G!╧£Z╦áW¼║|≡┬üs╟67╡ß╓j ¢1°-
°╜□╨YN}║nÿ▓bH╟█╬å¿ì┌b∩□ä□ú6[╫□0B╫╤-▒┼Gδ∞□±gε3╟r3V╨Å}
(¡√■2oΓ|▐□ìPæèg!c°╦□¼·3

╖5/n□ôj▓Y╣⌠¼ü╞∞□%M┼ïE&,¼v□UxÄ_,è≥ÜΘéH1╪ΓUu∞î7úÅ{;□jΘ<y┐<ß≥□°╞¡!is≈ìÿ\²6║Σ
wtü╗zδ÷═Ω·□æ╥ε□╞

öï{!r^│ΦÜIßú≈═ï□?▄╢'▄αÿ═┘yJLò·"úφW═¢an┬..,╠╖÷⌐╚£└ÅëVê┘╞┴╜□'0gKWlST¿
4y¬±┤Φ╡σe═ΩZ▌ÿ╔*├,ò]g<sj▌}wπ╓ï#âO╞¬å

z>_íds▄iU╟#□■cÆ│τ╗□□╩fφ.·▌Γ□3╕kàε▄â▌⌡S*V)]≡>╡U6æΘI□úò7}φ▒□àGç≤/z□èrñWÅ╫║█
ùû┐+*╖0kCä⌠-
⌐ë¼□E□▌=BAY.½åûd╡╞_T¿i□b{a╙s½{∩□»αdæ┼□~□Åâ₧σñ_σ⌐≤┼╧║╠¼╡-£]√Ag█Xφ⌐▄Ç
¼□]x£α□<Äε■╤□ê°Tæª Ç·╣□□¢⌠KT▒.Ä≈lH⌡∩┘Éƒ<□-4□F║╕┴ê-ú1£,≥pó°»{,qö┐█

□a;(╨v└=ù√ΓC¿ñî÷■┌á▀π$¡⌡O╝:Γ¼É9a≡░Ç°3∞~?&`$M│:▒┤Lt╙□├Çs╟Zæ□Fg6□ΣWî╕ë░y□n$
ïE{&.I¢ú<7o¢╒û°╛│½Ω7;□dhûσ²▒Wπ└□ß╜[□ª £#M□Ω=┐Q╫^à⌐≤$W╙εZíΘ╤Y╡σ┌╘ê\▀╢Küm#g
fs_¢f╕gΣ╣/╚╙ jwûçä╓ìq┐╡?╝+(vsf⌠.+⌡'b°▓Xæ°╥╞┤v<╘ÿ!)gφñ╢}AΦ=µ╙úV│k[ZL╓─
√cß┴à â 0.X5½1┘6^·U╢╬⌠åöQZ□⌠£╧─5ε╓╜QH□Sp0/╒hI╒╓□═76Nδ□

åûì□≥\Ωpbï─~L)■╥δ□╘ä╒∩_T┌ù¼JK,.U□-
Ωè¬□╣¼¬f44c──ß╫□□ñ-ÉQ╩┘δ▌∞&τ□_=j½k╥âí│n(L2╢/?<m¼2⌡┼⌡□.:7ò-å╠Æÿb-Y╪

ε□∩ú─╢▐.σ□₧□\=u╥ô□┘□Oò¼è╤cªï╟□Nùà.╕□·D╣╛─|π┘╓32╙n/[▌lmàτΣ«⌐ov╔╟δ╝ô∩²αtz∞┬
ûÜûIO¼╘sXj╗s□√─<^ß-σzP'■╥┤-3K"╒╘^╢□û⌐¡╒\;╬╨÷⌠²σª╔σ┬ô╧V∩2╤
_ .¿=J▐Yô5k╬·M╞╢z±u·IRw╥┘╝▐É_l₧57úΩ^M╥¬>█┘□mτ╙□╟▀'π9á_é⌡5`ShóC□└□D□
w`□╞ ╒«■═(≈≈xB╡FRx)&╟┘t¢√╧r╬≤«+%δπ3zî□±□¬mûu╓ñ□½─
·╥□ε[╕kßfφsJ\q=W]]╥╠εgµ∞q;▄ªƒÖ¢≡}fε+ⁿ├±╪Ló≤≥┤╪t<6
Å]╫7I┌T<v□>÷πφ(□W∩[nûg░G□□Ö_á[h┤GHÿ╢ox >«Å□□╫ó+/
&□/α□▄└\α□B╙oò²Ç□êåÑ0□

±━7╠ü≡□é9my┘ü□1ƒW╗2ε9FKk▀¿
≈Q▄,▓a╨}Å╘ì□6,«è□M9φσ¬¡i▄^□z=pY≈⌐▒ìîïúJ,÷εh≤╗φQóh░+}ûW|╩ΓòµvSkES□UIOÆi□=a
Ñ}e~W└CcAm⌡═Å╟

▌U}X62m─â°₧□L╟D-Pjc-
▌¥□╛l⌡δK*□≤agVH□φ▄K□▌▄Γ√┴W{}╓░▒├□£¡<Σä²éfdlhX÷║tm¢╣╞╪Qò'
Zâå-xö°┌Ry°M<oúZzå╡°□æWB╔5re║Φâ□g╡«║l;d5éqÄqµ▄▀□Å

■q¢ô4┼╟┘HwPts¬⌠╥é╫¬φw5G╨√e□£Pts∞□□£s4╝l□]`72L=8n,╦┌[ñ┤╨·=²╡Keé9ï=rµ?□7╠kG┘,{≈
ä2YÅßΘ ⌡umφ»╕┘¢T∩_┘ò^·|û╟°ç.é[□ì□"□«
□D╚▒O╣╣□~±τ╣┴┤S⌡π╧ï½=┐δÑ╙£■6{µåZPôm~rz[·.ßIûÆ□cΣ*ü·┘é╤╗╞ÜE

18W╡}{µé□è,╫╤σvX(┼╜┘╓^□ptR·âªê(Θµgå□ú╤IⁿÜB%▀ê╟y-
:W6ë─<≤□ò╫ë╖╨&»⌐»Å□⌠X7µ·V'□█Æÿiè┘Qât□□┤Äg□îφ╪syτ¼╚╝I¢°=s▓░4+¥pa≤|□Fîe@Wtµ
Θôüü■□∞├Xb
∞«≡Γh·x□m□A≡╪⌡ ╢ß□°δ└O□G▓b╤
σ╙+─┬TPh '/░□fJ"<q╝?òâ+}jHπA╒╓òfÜ│µ╔╤ÜXf╝·╓└ö°#M╥wq╧~MDyN╕c╓░□5`Mn¼
P╕┼êç7▐p;╓□G╕╤êMτ□±n0τ
ó│Ubö┐╕│□úCé}B▌B|úσ?│═┤8□8X¼┘╜∞╙╢α□¬□╖y│φuè□°□Äσë╦.8□iß8│─P_┬P≥åâ╖≥Tü:√u£
╞îM\┐P□═□à
┌ΩÆC┼═□□~ë⌐□s╧ÑzN║}f□¡▐╖òùpδεíï√╫╡¼▐=eip╘^äV▄U|⌠x┼╙û«│ëáεI╤V╧□Uú╬╧=∩┌±┤π□
°2▌hnïå└½Vï─AQò▓ⁿiú«<p-Γ╘x>Iêyf≈▄ì[-uö¬{╡Å-M▌gyHqⁿ□à£Γ╟∞╥)&□.â[lτúπ□∩┌k)▒
b╘╘c■gèwδ▐±É<5▄y
]{¼L╨¼φ½ƒ4J'7ªe\ë~k▄$□□'εÅ\,vR±▌)&W»ΓX;I╙EaEv□E
-Oö?¡æ□/Ä┬ü¿Añh«·╫□±üƒ┤⌡ô╓Y8┌_$E>=1Dα═√(tP□·┼±pPü.Å°¢±7ë4y□?j¢¥┌·&òU£áΩR_
┘=Gó?sÖ□Yß┘░ûRÆºQeªOL□ob,¢¿«'¡~~V[▌├W╧□σªmVj╘⌡a6ë>¿╗╣┌f¿┐╩Ä·M1│7jU
ƒφ┼▐√╟├╜%▄└gπÑ*├∩≡â[à│'l{5q▐╓a÷3╢╔=°□jYÑÖ*T?□/"Φ╓45zëP⌠Æ⌠□ûkûùï□]╬√ⁿ┴□∩¡╒
╧╣εÄƒoq┤δn▌ú«╕εG-╙»¥xx0]╠»╝j▐·ùoµ¢-┐W-}╜√Ω╬c"Ö<║├ú╓╟èÄ╤M¥ò▌□ 4⌡εΩΓ|æ╪&╓Vπ
ß■□[.╧□□²Θ╬¢╖w□6╓▌□]╟r«¥áY□TtK}T|╙□▒╥eéSεÅl╤¥>·αè·Hk▐□⌡W□╗V≡Fⁿ╣┌□ⁿ6$Lü
endstream
endobj
32 0 obj
[ 0[ 507] 3[ 226 579] 17[ 544 533] 24[ 615] 38[ 459 631] 47[ 252] 62[ 420] 68[ 855 646] 87[ 517] 89[ 673 543] 94[ 459] 100[ 487] 104[ 642] 115[ 567] 122[ 487] 258[ 479] 272[ 423] 282[ 525] 286[ 498] 296[ 305] 336[ 471] 346[ 525] 349[ 230] 361[ 239] 364[ 455] 367[ 230] 373[ 799 525] 381[ 527] 393[ 525] 396[ 349] 400[ 391] 410[ 335] 437[ 525] 448[ 452 715] 454[ 433 453] 645[ 550] 853[ 250] 855[ 268 252] 882[ 306] 894[ 303 303] 1004[ 507 507 507 507 507 507] 1011[ 507 507] ]
endobj
33 0 obj
[ 226 0 0 0 0 0 0 0 303 303 0 0 250 306 252 0 507 507 507 507 507 507 0 507 507 0 268 0 0 0 0 0 0 579 544 533 615 0 459 631 0 252 0 0 420 855 646 0 517 673 543 459 487 642 567 0 0 487 0 0 0 0 0 0 0 479 0 423 525 498 305 471 525 230 0 455 230 799 525 527 525 0 349 391 335 525 452 715 433 453]
endobj
34 0 obj
[ 226 0 0 0 0 0 0 0 0 0 0 0 0 306 267 0 0 507 0 507 507 0 0 0 0 0 0 0 0 0 0 0 0 606 0 0 630 0 459 0 0 267 0 0 423 874 0 0 532 0 563 0 0 653 0 0 0 520 0 0 0 0 0 0 0 494 537 418 537 503 316 0 537 246 0 0 246 813 537 538 537 0 355 399 347 0 473 745 459 474]
endobj
35 0 obj
<</Filter/FlateDecode/Length 196933/Length1 570396>>
stream
x£∞¥ \ö╒·°ƒ≤╛∩╟└,0├

ㅏㄴㄴ0□□

¢ α┬êÇαÆ▓Lüªéè╤╧

Bp+╙,═░┼÷]m»½µ0ZaZÖ┘ r+½[╢o╢▄█╨═J╖Ñ1≤{╫√

ïñσφ.²■ƒ <≡╠≈£τ<τ£τ,∩Ö3è□

"≡Eéå╥Ü╧±╒∩wMâÇÜL;╨JJk{╛═∞ΩQ║╝▓ÆIπ>ÿ▒·m`δ▐P4î/-
+▀≈ⁿ¢A·╤M╧Å╟O¥RSyΣ═oAzδ√r|ì│Σφíu└6ⁿb╬å)5Ö9ª░╧▒╫å9
[gÖ0 0╢ù3gQ╖╣°╤║»«6c{kτ╡₧╡αÄ/K╢£ùzVπ┬Vê
÷ "╓╫ƒu╬╥y{B[Ç∩X²jsSπ▄─
╧\║█?╤≤¢╤á█√µ∩╞|R≤é÷%╒ú╙käÇ│╖╠oj;wk╙÷`5T╬iÖ╙Φ▐╢■J`ª
░·Θ
ù╡ª▄─
òÄ⌡ƒ├·µMφì╖¼┬┤╟╙Ä·K╬m\╨⌠a╔kY└ªc╝YδZ[╢{ì░π█┬²[█ÜZr<┐`╬_─G└τZY⌠╤wcù▄
?+x╘ê

□.╖╛║ÇÅ□▐¼\\}∞¥▐<uL└T⌠
□□H░₧□<└÷⌐7□{τΦ□uî▄╖ ë▐┬-╞ap□Φa êXS□Ö╨□á²□√□░Tö&□╗A□□è¢□╣╪d□Q|□-□ □ä`ò
JÆ(Hƒé▌╗□Æ╬╟f□y█ôk╟f└±□ô(□╒□┴f□µσeΓ-E□)äIA□╤░ùq;╜
æ≡○□à□«°=⌡──
┐]OHⁿ}m╦φ╧>q]Ñτ≈╖9TñÆü╢ñï~_╗èB╕ⁿ▀□K□ε¼▀≥□□Ω□║▌□X#╛□╙NXv5>SâD╝Ω°ⁿ╓D¼;▒ƒt█Ç
]╨ z[X-u╥▓ƒO\WY□kñgO╥≈;αⁿ╡■åè╪;╨Äd□2□-0εäu╬□πq}nüKN╡?)□î╩NH√à]□¢╕-
4┐□∩≈0■T√Ωo{"\(╓C┼ë╩T╦╓«

"ó∩─πΩ¬`┬⌐⌠!¼àhσ^ê

ähΘÑAΘ□ê>ò ·╨çO═∩D2┤□▐û"b└ªÜyΩm≤╢ΩK│□└n■≥Gƒ
□»År□\8╕┐_─2·─kvR Am   o□▀«ÿ
ò'¬ú8p╝]xµ°≈□à-"Ñm┐ 2^├}□

êTuóª n╢ ⌐°

□±╧□í°□∩&Cφ[┴,>□□ L[┬□É/□üiB)□
ïägíê╜□▒r┘1ÿ╞û┴ö>_╢□ªIτá∩O¿GáÉ╫ô²□≤E0è□⌡╔╗□≈║□╠┬□¿≈■{▀¢ »□εk` ╤Q°┼/~±
ëp+ⁿφñeg├╥'-│└≥ LDC·Y□#╣■7·:U□W┴╥?:å┴"|@≈5±□îô□ εd~°Ö°:-
¥¿1'mo+╜Gï½a▓°□$¥╟O▄H≤Ç¼A=8Ñ°JO9╛ô÷+(└$╙à≈æ₧_▐q~M■┼║VÖφ`□:N■╡≤T²ⁿΓ□┐ⁿq"=
≤■╒6─╨ü?Å□τ├□Nß□"□µ□H
g┴□ΘûA·├ö□u□6q £;╕}a
□■╒□²Γ□┐°┼/~±ï_ⁿΓ□┐°┼/ o╙α╧ÿr~■└τ┼>·╡╧Ö²vⁿ╝┘≈YsΦτL┐°┼/~±ï_ⁿΓ□┐°┼/~±ï_ⁿΓ□┐
ⁿwà▌ⁿGGα□┐°┼/~±ï_ⁿΓ□┐°┼/~±ï_ⁿΓ□┐ⁿRäV¿⌡▒□5·?╥╟<¿≈q□jµ⌐╫⌠▐ ƒê╟/~±ï_ⁿΓ□┐°┼/~
±ï_ⁿΓ□┐°┼/~±ï_ⁿΓ□┐°┼/~±ï_ⁿΓ□□∩ú□t□~±╥,óOcΘ7I▒-ÿ├öx'Hl=□ªé□□≥ ñ¼âD░C6

ç▒P□σP
ô▒┤□£P□≤αlhàvX□¢`+lç¥,█ön╨2σÖ□M┼ª▒µ@│▐▄n^a╛╙|ë⌡┼cÆW■¥Q╪ó□2░┼▄▀□'⌡╖╪
□═p

┤┴ó_┤X`□s\ï□cïÇ-2∩□y@╗╫▌ ▐°╝□╝□╜ⁿ WWA□äB┬└Ç╜sä°┼rq

Σ}uΘ└WO≥'|?Y≤Qπ'½?Y²ßh□▀∩╒Γ±

û≥■ö□&`i-╘¥╥,□±└─Γìb¢X□cáö ,ÿΘY

ïc)l□¢┴ZX□[─û│╤╪σl=╖àφaO▓g╪│,ì┘┘xV└&éÆ²
╖⌡├╨■·ày┴≈□┬□°uíÜr$'*ûúπ∞A²F<t╥v$·ò ▀H69E?    äπÆy□*²■ªfƒ ▓!a

□=$\mu p^{n}$▓╡□

0-
╧□╥7□┐1«?^─Ssπ½yΩì▓y■°□■o=àα(ƒ5s╞Ö╙°╒╫9kk¬½ªN9m≥ñë□*+╞ùùòÄ+□δ(-3z╘╚ó┬é□·y
Ö÷î⌠□¢5╓Æ□□f╨□δ4Ω└□òR!ë□â⌠2Kyâ┘ekpI6KEE□╧[□╤╨8╚╨α2ú⌐ⁿxù╡║Av3-
∩Θ@╧yC<□ΣΘΦ≈dz≤(□òæn.│ÿ]√K-µn6¡¬

╙WöZΩ═«-9=YNK69ú├LB□╓0ùE5ùÜ]¼┴\µ*_╘▄Y╓Pèφui╘π,πÜ╘□Θ╨Ñ╓`Râ)WèÑ╡ïÑîarBH)+Ω□
@╟╕uë╓▓╞╡«⌐UueÑ╞ääz┘□πΣ╢\╧q.ò▄û·l-3¼3wÑ∩Θ╜╝[□│□╥┤s-
s□╧¼sëìX⌐S,δ∞╝╘eHsÑZJ]⌐╦>ì┬!7╣╥-Ñe«4
66▒║┐□µRX⌡□sτ□└α-=_-
oi⌠YöV²□αI>─■i┬≥╛4`l□!Ä/!ü╟▓«█□│1πZYUGy3╟6║┴æÖV∩□□x╓₧╛Æp'/Y┘W╥_╜┴Æ└ù¬¼┴≈╜
¿9╧╡r╢9#□g_■╢Γ7û¢]ó¡a÷£f╬╞ªNKi)=[m¥╦Qè  Gúo¼e]YÖΦ▀╪Çâ8¢OCU¥+╙╥Ω□│öÉ□□╟|
╬«⌐ô½°¬╢┬╞╡áaÄ»û+│¼ö╟e.δl(Ñ□y[û¬║¥Éδ=╪5▄l▄₧ïGP=Å├□1

□┼V╓Y7w₧+╛┴8□≈τ<s¥1┴σ¿╟Θ½╖╘5╒≤U▓Φ]⌐□▒╖□╡G╣□Ämêwƒ3╣╩□`«□îb=_-4ÿ╤±┼R2□
⌠╕\rû»h╔(s□3Bƒ□÷Γ≤α⌐π┌┴îh□W┴ïD^u\à1í>üΣWB2·bRX]□â┌╥úí?&Ωτñíæ7□(╒\╓T:(└π□U
°□⌠╡vΓ8□>□╛Ä▒F□_╬è╛"╤èO.┌□lF6±Uî2╖`¬╣╬╥d⌐╖α-rL¡πcπs-»∩─□╦─¬iu≥j√vIφq9*/á£
□░╕/#î├=X₧f∞[V9?^╬≈g+å□W÷□¢;□,□k:yπ□_â`╞'□□¡┤U6«+□□ÄÅf9₧nû≥F
╛╫öw6v{W╬ε∞r8:[╦□Üïx□û╩╣¥ûÜ║QF9╓Ω║σ╞e╝½□ÿ╚&╓ûdñπ┘S╥eak½║□lm═┤║¥°>j^[[τ□ÿ0
«íñ╛+ ╦ΩvÜ±lù¡□╖r#╧ÿyå╖TìÖ□┘▀╕╙□░R.òdâ£ƒ╙═@┤□⌠┘□╟Θ□╚ª∩│ hô╚µÉm\pæóÜqè±╕-
3╧σ╦sA}sgC=□╕ □ù□┐ÖïY╞ÇK░îΘbéRδR[ÜJ\□K ╖□s{1┘ò▄«┬ì┴"□N

?ô:□,xNßå¬□#ú¡(≥&═▌^om]┬~cO}□n╡3Q°╓║□╙≡∞WX'á▀x«
h-∩Z9°æ╟□╬:^We¡£SÅ█╢»At⌐t□b
ü╛□╨ú\«├╖#VÜâkâ
(╫_ë□╫╧zW}□∩┤ε∞zy;δ]Pa)┬e°6□6▐Qf}gê%G~6±QP[/σ□─╪áªÄ,F╞bg⌡4I*-
F>╟éEs□╞8█□╞⌐┴¡Ng⌐┌Hû&<□%[ô¼jú»□°░D½F°v□┌▒Aⁿµiì¥?Æ□½¬╛₧éùsù·□░o╜Kâ□┘□MÑ»□
╬

□U≥X≡√R

ò╗>╔¢⌐Ωåj╦□<Yx╨rK*,vΘ¼òìx°S}
Z,□}ò□°□í±╡▒Å¼*>r-╬╗h¡φ÷▐gYÜ0H2╥-ⁿ═üoL0εΣ≈ñ·╬í□╫⌠┤î⌠ÇíV¥lε∞

╨¥╕□═WÇ«ƒh□wá°╒Xô8

╖ⁿHq,╛«□│αVT□$1□µó╢ú-@ò─

q□□@╝ÿεcÜ8╠]□ƒ⌠□f∩F▌ü*z≈á╤Æ\╛SN─Ü╦╟╬□GAü8□£b□▓□YÇ□ü╠Gµ!ç#sæ□d"2□i╞[lÜ╚-
┼·ⁿU□Meÿ□ë╢$1□jQ□95▄ù;î*Aÿÿ

Ñ¿ƒóè□u2·ÉÑ□⌡□┖δP□á-F
└╨□▒┼ß╪#├║f⌠6ú╖□[4c
3╓0âR°╓□gè∩□~t╟Ñ!■ßÄKGⁿ@8B8Le▀Sε;┬╖äCäo□□'╧-┬╫dⁿè≡%ß
┬τä╧□□#ⁿò≡⌐;.□±  σ>&|Σ6à

║M╤ê ▌ ªL─ ä≈ ∩ ▐%ùw(≈6ß-┬¢ä7 »

□^#╝J°
ß□┬╦äù(ê²ä□
/□₧°n L₧┴□₧%<CxÜ░Å≡□a/ßI┬-┬□╘µπä╟╚╕¢░ï≡(a'í¢≡□ßa┬Cä□äφ□7í╦□¢âp□╢╣cs
□□□╢□╢□6□■ΣÄ═F<@╕ƒΩ▌G╕ùp□ßn┬]ä;⌐·□äMäìä
ä█ ╖Q╙╖□níΩ7□n"▄H╕üp=╔╗Äp-ß□┬╒ä⌡ä½□WR╙WP⌡╦
δ□¥ä╦□k⌐┬Ñä5ä╒äK□□□V╣ì├□□□V□V□.$,'\@8ƒ░î░ö░ä░ÿ░ê╨Ah',$┤□╬#┤□Z▄1yês

□τ□µ□■çp6íÖp□a-íë0ù0ç0¢╨Hh ╠"╠$╠ £IÿNÿF¿wGÅ@╘□╬ £Np□j
5äjB□a*a□ß4┬d┬$┬D┬□B%íé0₧PN(#ö□╞□J□c

B1a

a4a□a$íêPΦÄ*D□□F□≥ yäßä\B

!¢É%Cdε(;µ2╔h'd□╥iäaäTB□!Ö`#X▌æ#□I□ï;ÆoΦDwd□"üîfB<!Ä`"─□îä□B4!è□Iê
äS□a╘C(□C□□é₧□L□"Φ□Zéåá&□R¢□□□□ò□□A"ê□ü└□ ây -B/ßg┬1┬Q┬Oä-
É╗e?╙#bG╙xÿ≡=ß;┬╖äCäo□□'⌠□╛&|E°Æ≡□ßs┬g╘▀▀▄□□─_ ƒ║#pâ▒O□╗#□□□

‖#╞!>tGö"> ╝Ox╧□Qåx╫□QÄxç≡6ß-
j·M┬□╘╪δ╘╪□┬käW⌐▒┐P╝W□/□^"∞'╝HxüΩ=OM Ö≡□□ ,ß□Ω∩iwD b-
Uxè:┌KQ?Iìφ!<Ax£≡□a7a□ßQjz'5▌MM?BM?Lxê░â:┌Np□‖¿[□a□ßAjz+a
a3ßOä□▄ßxε▓√▌ßc□≈□εuçOF▄π

?
q╖;|□Γ.wx5ΓNw╕□q□╣l"ùìΣ▓ü\n°▓█╙≤V╩▌B₧7□nó□7□npçOE\O╒»#\K╕åB║Ü<╫ôτUä+▌ßUê+
╙≤r┬:B°;¼

qÖ;¼─▒╓□v&ΓRw╪

─□w╪□─jw╪t─%Tv1y«"ùï□█Éçé╦Γ┐ ¬ê?¿=-
~/Ωô¿{Pƒ╨£-∩FφBuínC}□u+Ω□╘═¿□B}□⌡~╘√P∩E╝□⌡n╘╗P∩D╝□u□ΩF╘
Ωµ°[PoF╝ ⌡F╘□P»G╝

⌡Z╘kP»F]╪□□□Ωò¿Wá^Ä:6P°Y8□°C╝p

±là;ö?Ä□║C°╓j',t□°╓j#£Gh%┤□╬%, £CÿO°┬(┬H╗₧úêPH( î Σ□≥□├
╡ä□w0▀°┘ä,B□┴@╨□éA□¥□□Ñ¢i    □éÜ□H□
¿▄:╛╘J╟tΣ▀Q{P┐F²□⌡K╘/p9?D²□⌡}╘≈P▀E}□⌡m\û╗P▀D}□⌡1╘▌¿╗P-
E╜□ùΓ6╘n╢ÆfzÖ█└╗ⁿRÜ£%ä┼äEä

┬8B =├XéâPL□C□MC

'ä□B9vèó(╕□±w?.□°ßNÇ}¿ó□□╦·ä□Z⌡jè¼è0ò0àp□a2a□a"a□íÆPA□O('ö□J
ëä□□▐Lê'─□LäXéæ□Cê&D╤0#
□Ä[æ╜¿?ú-C=è·□.≡Å¿ @²□⌡□Ωa╘∩qU┐C²□⌡3╘┐í■□⌡S╘OP?F²□Ww?Ωï¿/á>Å·g╘τPƒE
}□⌡i╘}¿Oívú>é+■0ΩC¿;P╖ú▐╩W_ΦÑ9^N╕Çp╢█ÇW!╓L8ïªe-íë0ù0ç0¢╨Hh ╟"╟$╟
£IÿNÿF¿'╘□╬ £Np□jÖ□;Mu□!¥ÉF□FH%ñ□Æ
6éò╓&ë`!(□□A$□□FO$8εDzQ=¿ƒπ─╛ü·:Ω□╘╫P_E²
Ω+¿/ú╛ä□╝□u╡hì┐D┤╟_╟∞±½*V:/┌╝╥╢ób╣≤┬═╦¥Üσ#ùO\.jû□□τ/▀╝ⁿ▌σ╧
*û9╧▀╝╞)-
[&¿ùV,v.┘╝╪⌐Y╠┤ï*:£╡□ƒv□ε□├:j;µv┤w\╫q□
¬╗;vt∞δ□╗╜{□!□□#╦Wv¼∩□┬░\Ç

□╠═ □Üá≥÷è6τ┬═mN⌐mx¢0≥p□;╪╞ä¼66╡¡íM@»φmI)σ▄;»-
"ª\▀û╒µh□╧½hq╢nnqNiiiY╤▓▒σë□┼èû½Zämÿ□□-ü║≥s+□8?\└`╖α□=Ω-┴δ□╒-
╖□□0°F≡8╝l>N└ αD£m?╦┘╝·,τ<√\g╙µ╢╫9÷┘╫F{âsû}åsµµ□╫3φ╙£╙7Os╓█δ£gá Θ÷Z°ss¡│╞
^σ¼▌\σ£b?═y□┌'█':'m₧Φ£`»pVn«pN¡`πφσ╫21?-▀A

┐[πV╞□èô4
ªVô╨j:h:d□[c□┼□+î,8fE╟U1b0╛□⌠□□}U⌠╞Φm╤è`9!j[CVå□¡åò□!╥α0╝b8hÉ└░╔
□_□╝1x[░8%xV≡7┴▐`i[0█□⌠D╨╥AΓöáYA-Abp□╧ïzGÉ=╗<X□»sî╧╘ëú2u┼║):±*□sΦ∞9σ

]Rry▒vèvûV■¿e

¡─╡ⁿ□╡W-8╘X≡Má7P≡□2□ÖÖ1`zä□Çk│âà╟ùïÅ1■Wí□`l=╘ªM∞Vy½'║□ªNw▒╡.k
□uTMs)╫║└9mz]□cW╓w1a\¡+î ┘║£_}┼□`*ÖΦ2╒╚╢┼M¢L%⌡□]+y┌ßÉ╙^₧□t⌐O¢╣░cß┬÷┤àiºé:
s!Z┌;≡[□├WdG;/i_□ΦÆv□ß-
9:d°à□│:░
,@≤B┘╠s3eùô╡±_òôÄΣ┐!∞Å∞ⁿ o□▄╚|W/□╝□·f└}║0j╓L·╟□T□□<╫

·yéï≡δ6╪

□┴ú≡$<□»┴≈L

░□₧ÇOαK°

Äßs½bß,ûÑ₧╥O(£Æx.V,□¥╕□ö□ α=Ω²┬≤Ç≈
<-é□Y«┼\ñd□░xC╝=Cm₧k=▌₧ùö□╨╥u⌡┬
h=─z╝Gàb₧≈µ≤╝p)O╥5

⌐6x╣║y6-□N+┤A□,üÑ░

╬çσp!¼Çïa
\□kß2£ï□ÿ^□ù├□p%\□δßj╕□«àδαz╕□nä¢αf╕□n┼y╝□6└F_□╧o└»□ΣR^r'▄
□└□Σ]p7▄□≈┴²ÿ □╬■□x□mdíⁿV┤lé;╨z/Z╣□╖m├/□tü□╢├

\3╩≈σ‖a□<

Å
wΓjεé▌ ≡□<Äδ╕ □Wv»lπû╛ ⁿ╓═Θ⌡)╪□O├3≡,<□□╞¥±□╝□√ß%x·wò<▌ oß╣Wα/≡*ε╡□≡:╜□o┬█≡.|□
┬A°□w▌╫┐(□
=▐Aƒ≈}^í╫_ß
⌠∞AO≥#ƒ≈Σ╥╧σ□

`▌ â≡)
Ç#LÇcα┼□_╜□Σ□║Y^G╛ z|uεûτÖ»╟6╠≤□║»□m╢Γ□o┼⌡Σ9₧╛┼╖□□óo□╬`■ⁿ¥x╓^≥¡

═≈n⌠ßs┴K÷√µΓY▀J≡v-∩»√é\µûδφφou`Fiä»□Ü¥≈□═ß_ßo≥╠╨∞QΘ└∞qÅO╤ç╧2oπ°╣²□δ╥∞≤║▄>
╕

/{□≤_αΘ≡5╬4τW≥J|□ƒ⌡°?≤ò≈└■ß□8"┐-éo±<·-

c■□┤□┬■/¡C- └»ß'8è+°3⌠

╩⌡

)Θ□□«1^0ÿ└D≡

ñ□¼▓JL┴öxª□░@ªfZªcA≥╧P⌐åöh·K

┐(╤₧á,P╢ä░P□åτe$ïb1╟êτªë┼▒xû└□□òE≈ùÿ▒─┬Æÿ╒W□!╫îε»□Å-æâ|SY□[î»ⁿτ╣21¥═å│<6é
□ó%□≤9ÿ/┬▓,Ö%0□f├9pT±╣≡"╢-
å°J╫∩=╡□□ép╪Σ²╤[Γ╣│w╖°0½e/Γî□ü□WΩ\µÇMèÖ0_╤Ω²ü%z┐Uî≈~-□⌡~═▓╝çA-n□τßs≡æ4
.└[ x□è∩Γë-é□□a2£□╡╗A╟n╟c╜ê╜░ú┤4 C⌠8f□0│□
□ù∩vG¿$Φî╞bK₧≥r▒╧PY¼║\¿àΓ▌□▐□□_÷ç□fεgÖ∩≈╝╤ú∩}╞Pÿ┘sá'+¢□□

▓å□    *òRiI┤
y╔╢ⁿ▄▄£1B ▌p¢%1HÉm├≤Gî□ssΓ□1¼╧2Fαy&╛√≤□▒¼7IXÜ0▓&[┴╥¼æ±í□□b|£╬Ük

₧8┘Æƒ□úÉ□öó"@ò£_bq.₧É°Æ:*9╓ö□ÑFÜbæ╜{□AG┐S□□;C*=╢[°╝░nLÆr⌐N#(□□nOë
O╧Ä□=Q□¼S□□#cbU□å
⌡░è╞▐¢c¼æjuñ5&╓╙█▓÷Ä─□ë⌠-ò₧RäA"╪α}~Gv╓φä$∩τ;4┴lÆÑ█√╣├─SV¡╬□Ñâ□□□a╙¿-
ëjÉ,╟`▒Y±S°#╬í□-
□╡┌dSÆ┼□°╓EÇ%1J□b¬

q*£□U\\□□YX`╙5α─╫Ü9#7ª'çEg╬£□╡?'w·Ñ√÷▒¿}3gP2+□o╨╞πcxê'■à╛▓╙╖Ω¡□□┤f╔bé*H┤
$┌l·#□-Tñ╩"&H]ZeDAvna£V:├□S-ΘLyi÷ßaJ-
╗J⌐╖î╔□YP₧lPεeÅ░û┘I├┬□bá^╟ñ▐áPìñî□fæ.0äkDQ□□·L∩;8│W□H{pWå@<┤╨╠>□í┬¡╕≈cäk
□ó|#î┬O⌡Ä└á*c□╧□·'~çó□G□╙ôV▄ô╞h≈ßö£bà¼∞z╛E-    ë╢<├≡ⁿ▄□□áb╕]░X

|°J{f<°╙□╧
□□ l╥╓○∩9▌s(m╓⌡KW_v╫us▓à[▄╝¢&&°K═Θ╓U□┐╝δ╠
φc□^_p▐²°üδ□∩Qq

Ä(□-í±t⌐B╗àδ□□:□─ÖT)┴l▓*J½côTz
&eg@¿≈╨├ÿ

ìVv{□nG□$Ä"êMRv│Θ;□ëU╤≥óaΦi╕`i□□qµ>C!_6ú├≡∩k╢□^

| F┬) ┘ ≈ä÷M□

P□□Φ⌐gW□□i□rzí6>'┘û□°╦H□□╡U‖3.5Jδ╣[□ò□□ù□ú±─i⌠□Ñ□_ñδ╙ô5╤├h«╪⌡°\àCj▀S□┬u□9
╘·j┌Ñ,3å»Ω÷╛<Fg□□å╬ïpv╝.Ä·╓┼τ╪ÆsΓtIj╜Z⌐─□ΘÖ╛□∩
@¼┼▐bá▓»╖p\□
□□Wç╤╖$£ 9öC┘╖KXµ~▐╗πdσ}╤╨-≥E#╧□÷ú

V≈nK╚╩┼σ╧!▒□╤á87.╒¿┼}sc_X╟╛╤D°╥L(╧├]3□▐ñ╪□□]VVdfª┌-□□╙-
╠╔▌æö¡╒¬1±□$σWEk5Q╗X□8└ε=┤Co□&ew{□9╠<□⌐τ»:zì╠╠╧╢+πS¬Γ¥²O>□⌠·G□|µsrpÉ□zr

╣z■b(□¥Ö¢k╚┼A?⌠οφΣ╕u│0~░α□├,╟φ,·îa╣ⁿ┤ægRy₧╞öeM╩è╒□₧╤ñÉ°¼──¼°□╤sâáë╥D╖Iôf▒
┼^Æe╔▓(ë%ΩΓS
¼]╞ΣΦA╦o:÷⌐╬á□□|╫┼-√ñ▀~Qn~░Ñp╪╧╝"□Vö□□ä╡°┐â└UÉD\àXHà
}OoÆrùp-
□└$<Θ□□âU▐□x╨ªmW*╛ûε■□ÿÑφpäWi√-(|₧°⌐táG/?ó□ S⌡·-B╦╨'O□|DëÑ½-[yÄo∩k│SX╢╜ª}
qm║°'½|rjδóbg~¼╕z┴²
Gyµ⌠Åⁿ≥╠LUΣÿY+fù╓
╙x*□G;}π₧îπ╬çR╕ò╞╝Co7ñ¬w ╧αI<B╕╒¥ZlÉ□~├«∩
]▀═¼█□Ä╚╤}å╤▌,⌡aGBUd▀-Φ□N!₧╬9□≡Ç╞w¢B£î«▀╒╚á=ö,┌┼_LNDd£╚╧+U£□□□□┴å█Æm╢╛╣Ü□
□Wö3,╟ñò┌├S▓□├¬√ª
Å≈)╣%╞╙ûƒaOp╟□e╩═H ]□¼÷l-* ╤═X┤ªá╣
6Q□¼û$ìA╦□▓'σ╞xB√g≤╞⌠dI╘Σƒ▒x≥╪·╡cBâR□+φ^¢E£δ¿
Q(=W□│K·┐G╕▄√à┤□O₧4p╥∞ε□│░-g6B╕╓íU█¬⌡╒²oSgΓ█╘Θ╛□ª╕∩m═í9╣O▀f9εL4ä┼
≥ì½▀"m-_√▄¬e{╫îùOj<Ælπτî-3╖╘¬┼#╩û□°e/▐╜¬t⌠□;/□√G╪+M>oé╒V9┐T╘⌠-
Ñ8"=εÿ(□╤0ÿ╘wûF□╫╖uZ3 yÖaFαoyjç╓ZmTåT+σÑ

)ΣτyoßÛ=·7°á–□R╚□2░ª

o-·≥æ□□□□Ö¢ƒ?"ö_C≡~ó□nóS5^δ┘□¬ë□3┬>┬□¼Z₧□.äªä^⌐□Ä□₧VX□⌐
a_y□√óf╧□OZS├□Æ&$╚│╫>» □₧¥ì╥çj%E°░$■⌠½qLcñtHé□ÿ°PTd▓╓ªδ□¬-ë┤íEc├├≈╬ç└f5
KεfzGáV□bj□iV4□?φpïΓε╞√Z□n≤┬┬ÉB²√─¼∞ü;U≥╨;òDw¬4M@─╚□┘□▒□i¼g▐h□┐Sedç¬4∞4Ñ!
iLnΩ╚╘□□╛Ö W3δ,K□▐¬T┴║╟╗â≡hSF

K□o╓ç¬%&⌐┤□φ=₧Iⁿ■□¡┴ùCÆ
Γp╧□α«│σε□╬□
─
╖?□ú □╫α□╝Γ□K╫─¼Hf╔╛Æ}0[h╔f┘┘*+ ¢E²▄▄n□╨Ñ:
è{è{pp3╬δÖQ╚Å3╝τç□╥íå╗N:ε■Σ{·ÿ|Fà≤φ□:p\ëç,┼°7╡ò{▄q⌐⌐q¼¬ΘΩ╡·ß)àIÖSG&z-
▒ì╚Ω╝6sxó!'<¡tΣm█3ïR#╪╕Q3+r□éÆlΓ5╢ñ╕Æy□╔eà├┤□╔┼°│≤Mv│■τpKªg╢97)╘≤]Hb6ε╤i▐
»─u╥H╚â╤ε(H▐%╝□Z╝Éτφ0¢ÿ)Q■;▐yB7
y83╗8[╚N∩f≤╗TgπgÜ□3zΣ□■─ß⌡┌╧╟6≡íD□≈φK■±%▄≈l╔-
^╔□Ür+ªτu<╢ªb≥e{┌╙j╞□─j□□║□mRQußÿå▒ë)òMcåO.H╔¬╘J±ε╘,SlTpΘ┌□/]√ΩòòAæq▒┘9&[
ö┌h6fO╗p╥┤ïkÆúM╤□□⌐|GΓ*JE╕èⁿv£╠∩┼w╔≈Γ□ⁿ^╟▓▄As±,╚εR╨*·╬è_╜╒□U] ·z╧□≥□□]╣ ╨
₧□*┌□µ╧»k¢l□□»□e╒HÜl╟E{/+_Rƒ╙█ÿ~╞J£W╛ƒ20Æt□▐□ô▄-¼x$╨□j

à└ÿn□⌠░▐åO½2Ü ═╣n.>□å.e ╬9□□█_XÿÖ)∩□δ╨ΦΦå□>Φ▓)▀ƒ2°□÷εσü□EÿÖ$|±\─╩□é□%)0(└
│ï]î&Eú□?»Q╟ü□╢XcRñ·SL□c¼□ü-O`ñòƒVk≡N«╞Φ-É█Ñ└≈▒█-
ë╒hî□kTαV╪n0DJ▌,o╖yn$□ûy╨=t╖╬⌠φsσ±□

y■□ Ϝzâτ

Vèí)□<┤▌ ┌╕□□₧░Z

>╓á□ƒ╠╡{≡#ÑæçΩYÑ&7╡╕█ûößïNè└s'□åwY┤ⁿ□□#ô4□Σ

ê□╓ñφf
□üIIC╬□║╢
L⌐□╬α╚·oú□o□□x╨[yX₧┼□á£ΓüZæ∞29à∩ç±jÜ`5╗╔╙▄ù□?├□à□│₧5lY_┌□;δ─╪├!ö▀▌o▀í╓7╔Ω
Γn□□Z ]╜□▀}Σ«u&▐uⁿ@çΓ□ü:>3║@»□?┼□e╫*ε□l≡ nyÑ`ï□┌à┬a∞-è ty(ƒ!ò«)¼¢□vI≥~ú
╗ò»╫/«Σ┬ß╨É^O*
9^╦╧░O⌐iä□7╧π}= ╝□?«°╞Ñx□wL□T∞H□╧HÄΩ□Ω□üë║LuFFΓp|+hv□
1onFäF4┘µÜÜ⌡╛┼ß≈^Z£□╝Lπ}'è▀¼·╡g≡□╪≈~pó
phn¿|□Ä□W<Ñ1fYmY▒j┴≤ÜTPl╬ê
□=o□h╡┘2ìj╗m[å├─»}[·≡□ë√□Φ8¬3▌Z{⌐Ω«▐ùΩ};▐≈M¡╡$┘Ωû╘─╔ûììmY▐d□â▒ì7b£□□l□
ä□╙Br╞>ô≡Bÿ╓□K▓Qb□$8de&/ß$o≥

╟╟
∩pÿ□╧d□2╝!H~≈V⌡&K▓
╟╜τ>G╒]nu▀√} ~ {ÑtF┌□▀□D¢`ª▌⌐FE╚□2< ▄▀6εNçójO18□ +å█╜¬h¿.□╜□╒

$~╬¡àmà6"    .╝□▒σ°Φ░    *□╛G□╜Γ"╔ôxü7
α□□≡Ωà¿c╔lá∩Äπ-aHoä√I(⌐}══;P

Cσµ░╟□cσqε□Z°┼( ⌐┐▀àí□è╜┘░╟□aσδCQ.î╛<≡╣^╟@╡ΣX└Z'#╫┘⌡
[F-{╞╓Ä·╨□σ*(Ü*9░¥δ»ⁿ□qèΦDt □□║SÅ}□□Æ□{□í□□-N½v{í▄É╖,▒¥-
Ñ(┴t╢X|ΓTΘ┴ƒ▌{╧□O»-□╫ô/=P^x█┌=5X┘▌c╡v∩□□┌├█0≈Θ_=ZΘ╝≈┐□■₧_|«┌}∩╧ƒ□╖{s▓0yr⌡
·√6'□ôwC╖

∞╤│@║∞└;°╬q ├┐□d□`p╚ê□ÿOσ

I*n≡j╪Ñh1I»6n ε╥╘□F┐dk╙ï?█qΦ╔├√□M╧╪□q

ì□+╛╛╜σ└┬┐ ªΓ║0╗∩hª3¿├^ƒⁿ∞drß·VT%R:;║∩ª┬êè$□╬[Γ=Hm╠oé1g@>╪≈□ä┬₧₧M⌐#Ü,l4τ:4
╨l┘"□□<╠ttÿè`≡τí╪êJ!î┐û□□□·uïi□□û σ□íä□╚7cê7□«÷X$δb≡*c≈'ⁿC⌡Θ□□╢nΩ│{█–
╣æ,□÷{╘δ)┘┬≈5\g■╪φÖ₧░A'ÑH£á╘è▀□ï£vßôìΘ~Å≤y╨□å≤¢J95σêu□■╬f╟~fKz⌡
¼≈gí~⌠_·'<

$i□Y≤□ñ□;y₧╥rY╞

[Σ□&y□à⌡T□ä□║"xÿ╗τP·╜╜ƒî∞6CG(□□ÿ4î□í□T
Djßïª─IΩ□'VI□»%W┼□□<▄}αëë▄Ä▒óN&┴pÖéR$J█║²φaS░o▌╞u╜┴Ä=ƒ□ï▀4ÉVKI□ù╥r:▄5ûtg|
┌P ·¢╫≈à╨

Éδ$╘¼]½28ì╬áÖ▓y¼jg╘µI▄┴Lig▀╨æ▒0c`╒î╓├Z▄zÖ╓bR█FOÆ≤╥Ñφ+Éàm@\ê≤BW5cT
 ò+╠XwQé└ª╤─Ñ·WD▀┘┬nC╢'┐Mú╛▌ @ eW^æ)!#JÄ╓r ~[∞²ù,uë╒]XÖ
Ç∩┐Φv X¼ΓâÖ╘I!ô║√<┼M⌐º¼M╡ε╣Z¡»ò-╖ÅV√Ä²▀:▐Ñ░º²αá┼╤x╝Z░╤Ä$J╪i⌠∞╤»▄╓₧┘
²─═╪╛║┐ÿ ╞°║é╒^╞ªΩ≈D|ƒIM#&α;è│-e2╠a'yè6┘°îd═M┬£H╚äàA╚Ω┼æf■ôif>?#
U₧εL╫°&~HôåB*Ö╖╙─`∩J{6KδdtD¡°p£6h±/yCzXüU╜ {\⌐╓╤ä╘≥é±E«╝'ÖπδDnΓσ
Jüt&ôè⌠6╬S¥ôYΘ≈z-@+»1+Sß⌐ñù╞[αΓ├f0╥1½┼τ┌óÉΓ╫'ü{±òª│xbΣ8⌐≥vg
:ⁿ

·□■$⌐÷±┘\┐_C.ⁿ╗□╖┤gc□+à □√1í░ª#╔éâ&~é═Γ┤-
□ì┬□L£▓é╒c√τ?½3*□M_úz□-√ôFOâ□^°₧╟▒≈5□□R□bC

╙≥ □XdÉ⌡╧!>∞

bAé□ê4¢I[└□aαÄ

3¼╜²□»F□□°êMq□¥sJ╫ê╟□□3
3áP`□Φ6°□u╜5√├□R╝æ■ßM<L═╠□╟┐╧ó╢₧H¬═┼Hµhjûd∞9_¼█ÄÖP┘┐□╣n"∩TJ┐┴╨gI╞ûè
fèzZ≤÷T╥°æ□rì□¡╪l
3□5EH4▐□z ²║/ñ# ╡Z▒≡□ç□⌡1Z≡?·Éga□╠£□3□I╚w□0{┐□d∩□╕ ├g□╔ß▌
┴uNÖ%┌)I]N□BΩ■Ü░╚│\Ü▐"ª5╖cS.\╥)⌡¥ Aw*╔ß/Ç╠6□╔╗□@j~zß╦ìPs□╓δ
□¬d*┼┬1□"¬d8⌐□Æè!Ñ+oß╟≡▀@»â□─J├┤▄□£╬µY$□@┌τ░╔╝Zâ¢╨□3íª9E□}?ïfawÜ□╓┬│┘xox

5≤╓□≡á°╟=□y0▌3µ┘µ┴U-°□S□-□aƒ╗≥□<úPí□╗YìVφ∩┼ç`1èùâ□]o≡è*üÿ□╡Eâê╕Z2119□│²D□
ª┼çÇò╣$ñ□░╢┼½■

F¿ÆAüπ╕\«╢ⁿ□m\&Wïejw□┴ΩIE┐b╟ñ≤□ⁿÿ>□Äà4àçn*□▀É∞·╪∞±
Ü@o▓gg%úª5┤ä▓
l=╨q╦π█ó∩nδ║)╧ûzr7╟¥îZ*U3ÑÄ> αmσæ├├╛|╕'¼╖ylîà39}v»C□Z□z╦o╡╛î╖ì╧ga,±ë+o□□y
□     #]╚c5V)w■"╢
$□□∞>^Ä□¿|╬MÉ╔z]09ç

≤Jn╚:á«□à:X□╢≡≥d╡^□□É□├TïÆ □□>ΣG┤□╘□åÑ«C4^⌡╨P¬1□à╨□╔ε°∞µ╪Hi╡Åf├

gêÑ□÷ñ▀ƒ┤+<½Vòâ;▄□\°ô&▄ƒaôÖ╝#╖=ùZ□╙ú┐?■ⁿΘ▓åk□m□jîöè&╝ö╕NB-
Φ<I'3zzµhq▀┌□π╔□□■╟¬Rzl7╜▌2êI▄°»æ\╜^;mC□╧cG□□1úN─┘X□⌠┴□e▌□±]┤îñÇ(╥4ZMEà┘G
aƒ3/□f?y5r╣□ⁿäí≡%X▒~εú~ÉX»ò┤─╙ Üæê┴îdQ
┼ìôRs√╨å°₧3╖□·∩ⁿ·Ä`╡?göô╕^¡ß▓σ⌠Ä╜ûL5ô□nπöràöx╞Γ5½LnïÜ °∞æ╙/▌▌=ÿ□Fò┘╦║'Ç╪}
±╤≥φC~'τñ¼aDDJJÉw"Gæ├3{&╫∞âª#YXâ╪µ░╔Ö@`R <6ë╚@\w□ÖD"¿¥°□ö▓ °╜τ▀╥╗╨δ/é╔VÉ□
:└S¢¬ê
≈Tÿ□;çV╧ßCBÇ□M ⁿ½ù{2≡çÿE└P7²·½B▒□XJ╤8╢fF┬ⁿÑ═□□W
εêσß1,□╤h□è-┬ë□ê┐∞;=┤·πUÅ┬û≥·S6àûkπR▌≤⌡ùr□¥□î□m~Jéδ5jOzu¬åσP╬□░ö□8)3╢
▐$ ╧∩╗o╡┌├X═·├╙'□¢·┬□|#▀╙╣≈╙;τ_ú──èBτ{çr÷ü■·o╫∩□ƒ┬P6\t╞;□*ú╫┌₧░8-"□

┐â╢║D▄□»Iet¢□╢║N}∩╬"I▓|┤≈≡M)ÆRh5"CΣ»╔C╚'Éô⌡╚±[F±9l╦l╣0╩Ç└iÆ°3]ÖQ≡8«τ6═aGxΩx
σ▌╡□▐─:Y▐□┘┘àLó□3wT3 ┌v╬0]e□lΓÅU√τP█9┘ÇÉ▌⌡d.°□4 □>┴E
ï^Ωùü╜╕ñüT5Ç┼

□□x!v5╢□A╘├Xn▒Q╜a÷╨≈YSv∩□{v=:□ □äP»{)▐íwÖ╡R %#hì+^tTn/{vΘ⌠□▌¥:□╤∩m
□L>9ëΘ╒jOrUΩ*}h╒-~▀)└!~üφì÷-□O&6▌w╙□e□┌
ëàC□âR╣║Tj≡┘óI
úÉrúwεF╧'□÷áë╩╞VGì╞@╤□Θ÷⌐LPô□,èÜΣn╒9└b¢□┌±aα¥□NεG8É□}╛fçhk±"□{-
□╪□<Ñs□╨┼Çò`┬u+

\α /7□e□╙æ□»fyªJVjδ_ó  □#w╤í╩?ΣG┤«9╢zBáB
cÄ7öO ¼Ç £2ç□« K»■Γû▌□▌□╞∞xtr°D'-
ÿr¢Γ╜ⁿ╬|¬□1hC½▓ûT&∩≥╘═÷╬í╡└R∩äµ╝½□²]▌å╧gWòSk°rm√╞╙*O!□Q□□¿]□1M□╔ó╕ê┌îNτÄ┬
²«æ,H°□nn<¬ïb╓ΦK□îLJ┤è□j□½î□█□∞,±

ü□ä-□□ü╥·≡╩╗└{□opCµ□G□5âipFnVáU╣□╝A■╝¡f¥#»éÿßr-
|ÿ8┤u"ryδ□╠╞a▄Ü□≡■□·╒é│òx▌+¬

x□╚
,I±
!▀ⁿ?Z;&z√v
&U└~ß□!S╢o:╥w|µ╬Äεc▀▄w≡╠εΣ;°µ╓d)┴bΦ{±hqó╫ú3ΘñZ7kt□Uî┘ñΘ<±▌Å-
□ß╘@▀╤│[]√>µδ□O□+─^y□√□≡□¥╙í□'F5bàN"□÷Ss¿}&_▓pu□ΣÇ╡╣└'╦«è║_ªLC□p)3□)sIΦD
ín∞wZ┬
A□[mO3on□□q=□√□!úÇ□a=&k└ó°Ü\0%_S╪╥>_╩N╘ΘHpδÇ»z|M`
□π∞╖φ^¥T*ôjⁿ□æ╡óIÿÅ╫ì8÷K╤

o■╘µ╕RÑd□@N┘+oIÄ□DªÉ;æ;f├aâ/□ß(R[

éδ∞□âí╕{

□k4▓¬╗H□:╕à o□!╓∞÷í¢□□□░íR╝□╟Öù»*g+╛▓║r□─[τ@╕□]',□└δe!h╗ö□<'0╘Eí^Ühfφ-
1ÿD≥┴P2ë▒┌J1¢Σxf'ê┘¬σU>E-f#□R╧è□c¥^µ1╤>?«░°□ü□▄pj½╡╗-
bfpT╬ªB>αi}²½╦┴□bî□ZòfSΘé3□b╝╒╤σb<Γ┤D"ôΓZ□ghE*¬¢>╡9FHσrà\

¼⌡╡□Bæ-┘≤Σ  Dï£E╬╠>÷╪ü│ÉÑ≤'''çn▌
ƒ□╨□Φë╨s╪6^>Σ□:□-'A₧wΩBε⌠▌'╧û┐□□¢²╠└┴¥'!cÜ╙σcò[*7ùWWzh*B$□□)╖WI╙or╙
?0

▌~Ä- L^□8∞□)Mïe°L¡─%°╪ó╪^"ΣH"╡W□ü«@ÿß□ïÇ{Ei□█□□.┌ ¥╙äj┴xvG. ê·SWH`╢□5+
TfM□□╤√ÄOo□╢╞≈NGMV╕á +¬╟)å&
┌/╘#»à≤αÖ┐Ujⁿ╒úc╤□V□tQ⌐Æj═n}{□²φUDb∞Φµ□□┌eJz
φ;δé┤âV┴╟□□╥â□á╥╕åδ□¡_*HKà¬o╙□¢παwüé█SA
ââπ%╤÷□□□dg;▓y╢T≥î√α±□qà□╞_╘:Å╓úEèÖ╕

è╙ª╥xy□>Θ□≡□□@V₧-ûC□[EQFÜ□
□┬KP□2┬ZAMí¢Φ□$Ω▄═█─▀□05¼╦╨₧□6□R≡⌡□□«R▄e)□µ¬G╫DJ,¡Æ□─;I□èñ<■í┴□Φ□ƒ╧ÿâ"«ÆA
`A≈#╗f3Ö┬ö-┌═M

G?
□▐+Ç╤àj⌐□J╟¬AV1╖whr □-τx⌐╨□⌡É¬û╖*▒2[□┴MO½═,□íÆë⌡tê▒α╖²+'□-
┌r╓≥□╓á□k╥Ak²0┼(╘Q□
f─┤╙`^%·╤□╠╛√□╖£¼z<╫H,>¼æ□s□D─_÷□Éδ╟æGfx`τc╖á□<8>▐]▐□=┘╬╟wf□{╪¡Φ▐
-□#É□τë#□+?□eⁿεü]□

B-T'╨√*□òj┘d)V$^{n}$░ÆP¥╡

i□J╨

Æu3□½d+□┴Ñ&pyá»æ(■°Ö>7÷╟σ,]∩²âB.iOè╣ñ┐╚%wΣΩU

@┤▄□]┘┌Ño@╒□╒r╓φFr╟ □3'°H╪▀w□╤!δæ▒s½æï╪w□□q□]\┐╞□e#ƒî«)Wí□vû\Q└ⁿl>⌐BUsΦ]□
╚|Ö½░é□╛zyB}╣╬lê£AV·7K□□±□`fQI─αN□
□«t□Bí6╖RΘn
à□.Ñε□>Ñrd╘□
:h)ë╤jë╩`3ΣÆΦ{□Gⁿ╘ƒq*ò╬î▀ùvlî+²°∞╡≡□¥Äîó□ú╫Æ

□□□ÿ@╖ODL2, v□·│┘▒╪¥□°┘ΦD⌠6□<<wüèéGQ8uΦΣû□⌠□≥■«Γ¥σ□j3$┘□□j╥╬╥û≥F°dp
µ*B╖╤_╬TΩÇ6▄□,₧L╘a□òG}·o□▌╤╢╞ä╫─°□p□╦┐æÑ□HåòεB8Tp3î╖□□□▄ÇâÜ□,╟^GµWs5æg▌ ·"
⌠.Ç"C"╨ñ╙«╧'╤?qü□ªh□±fÿ%~g□□□□ê □°á1ñ□╓Θ⌠

P╨⌡î▒^(ε┼Lbx@□┼╜°Σm8s╢L╓Yi╙°½5│σ ╙-□ C□A£û╤╛h─p]╫╤┍-nu□₧ÅΦ!j₧A╥□Σ·8r;/-
O&£NZ─d["╤╢å╓╙╟'ô≡╞□▀yç□╥∞ε╥dy3|R□Hz;□ <P╬WZ□k□r□6╤?□≡4┌□□α□òΓ□└Æ╢å╓ªΣT
α□6┘■┼□;Tw▌¡□±G3╤╩Ñr╖╚!□∞ⁿ-─ú0≤£<╟□A½óÿ≥zæ∞╘öb`c□ü╛█¿Vî╞í%₧Ü¼≥σL╢╜▌□â╞╪JC
ê□V□ìeI□⌐±□Ç∩□bRüÆKÉ□m╜╖δÜ(╖fσεF9Γ:┼A⌠Θ%Yxσ╪Ü└░ùæß0iùhX╖
╢⌐úk┼ÿ╨«¿W□c¥╡Jáöñu₧D▌□+ü╪;+ΣΘó□hMδ□D⌐ßv

5⌡úZ═ob░Y≤╙¬h 7zτZ⌠'"┬R

╙ #╙°g╗╗-#░╨q>░eï≥6□┌w╦¿σc≈B▄
╨²J
x□>ëñ"üÅòo+>£┌
æ^_□)ù┴ô≤àü{¡L□╓6RewE_╣-
╨rÑZK≥àε Üe∩□cóûΓδ╒AQúδ≥╞═·r□}0µñ□─║`□□X/|{e²Ç╡öP┘□k)¡Ñ□t╪_9╢û□□@zÑ4á╫∞6┌
α□¼ÑφΓg╡╥╦jäB∩ëV>«¡5<ΣZ╘Ü□«K╒═ƒ┌□S2 ╤░█╝:`□%j□gx²╨"□X □≡□┐$□"iΣ╓z╫6
╝A:

□ôm3Ä0█┌ù_σσ|l╚7 $┬û

í□_∞ΓIª¼╙7⌠÷┼;v«

i□ïj╡Φ□ e□□¥oQ⌠Y┐╓b╘kñ
¡δg⌡L□Cn╝≥-·□9é□□7╥_▀gf─^@lê□█□â@⌠«≤<½-□□ k╦σµÄ▓% ╡∞.$□┤╓@<≤░╧□ΓΩqδ║╫¡∩ΦZ
┐«│1rⁿ□Ñéπ□sHV┌█□+□Eæ!⌠□■r╜ƒr█,%|∩╨²öK╛⌐·□'δ╧Ωƒ
ÿ7 ▌╡²E*Ñ□Niöª P%é╥□0¬█α╞½□qûΓ╞+ayy┬:S┐╗ⁿ÷½òç╨Σ□□╟
æπ┐□æ╚7k{kÇ[□ª▄ßHïAIw□«□°□uïTM□□·µÜKÉU₧ß{ç║□bmâ▒JS╘α:\c#`QΦR┐\□╙√(ƒu□┘]Iÿ
╡åê║?û+l░ì╙Nk╝9□lK^H░□L□O▌□▀╥□q╩□r║┬&jΦ≤càì½╙Ü`ux8p≤ëaW□NL□╗J╪ù▐i▓╛gl╟□Θ⌠
G║□║╬=□Væ║■□□╥╙'k

äu□rç`□□□╨τ?╠╨hU╨kE]»iá╫a╓7╪ÇH+□T█ Tç·□ⁿΓì┘□├⌡lB□░/Å_╟&,□□Ç▒□Xä≥ò╖□□`□√C□
;O⌡╪QíK⌠(∞□¡I□;çZx╣j╚k«m╪▓┤

┤ε<╜┴_h▌»│Rƒ)╤ybε«π╧□iδ:±∞]w>s╕ma▐É-∩i[ù╖□Sδ║ïδ≥□⌠¡;₧{`¿∩□s╟ε°▐²C╜ƒÿ╖°∩└┌
xh⌠@ \cíæ□░╧bßq□□slφ│pτ⌐zƒ┼⌐k⌡Y

¬G?jƒ┼u>ó╡╧b□ ·WΩ│╙∞xtká╖½╙╒É□6$╘c□├#π   í□ ₧&╘ƒfS░╧b[6╡:j@/-
□ßtYσî;□╢╘M□±z](nvà⌠╒╙╙╟ï╖¼M⌐`5÷╖²âΘ5╗Eì┴.□²G╖╫4åS┴Ææ□▒¿('òáp%N┴╡0
ⁿ╘□:╬S|dêS□\âåè╕¡I□·I╕╞v⌐ª+╘u■~╒Æ╨r╩!á#┴.□2%%╚│

¡!□□*ròjx╗█┌lJç╦Lô□å□√Γ□□╞□╛╬Φⁿ½Kòπ@‖ùSßR9Ñ0╘▌╣âW▐┬▐□sD▐¼G
╜Xⁿ╝/φK+@╝╕è≈ □"Ä╞▀(□'B╜⌐)≡╨□□\□

/h□□ú¬□φä□F¡P□:▀Φ╡Æí!□╢+╤□bD╒ä±φ║D□t"BïldBhÆ¥£ê¿aëeó╡S╓╩╗■k┐¼
:qú▌╕╪█┼╜□Å°7ùôF□!S╙Θ□┐>∩╓□⌠■«ΩÜjù?╝⌡■ußQ>¬ô□8.U╙Σ\q8ΘI╗╘\≈ΦÜ╤n

uTÄî□T&│!□╡{
R╓aa,Aï#Γ▓yóⁿª-■╓JXí5¿T□°╔Ω╤K
f□c±Ω¥aù═□σo□9"┐J-B-E╛≡□╥å┼A╩┤□[à⌠"□▒■Y_Hw╫)!èW▒¬²╜S╜:òJ╫;ET∩A¬w┴□δl╝φΦ@█
û}□ü7π├o«ìâ╟å╞□▄╛í
o□TO⌐`╟□[■□îτσB</□□2═Nf╕ƒS╚uAPo□≈`█ú╪U±:┤

j1dæ¼■4ü]ì¿ßÜ

┤4┐¼□╟ô_┼$2ò'

S°Æ≈□¡üñU≥╜║@╤∩m□□Y¢□ù╤ÉåJ+
╫&1╖■╣é▐úb═╣[╛▓{τú█□╦à≤6ÅA╔4□·zS┼¬ÿ╤└σ]▒6K6┤

□]╫f┐ û▓ƒ$⌠²□□√┌xk'G3ñ□□`‖≥{∞aΓ□╥Ä|N╘╥g5□eG□±┬j▄┤I□½¢σ□Lï╝e╗▓~C
·5òS░aåùè┌□,≤+éS╦╠°/Ñ┼Æ□0Y▒□±□óù_┌1s-
·░çi¡╖ûï▌*v└∞╗:ïéφ2□vZB₧ê&tWü~═ⁿ□`E║╚;æ]╚╓≤k{{╚╗2évî╪┤4Æ÷Ççrπ╚«≥╓¡Æ

7□'┤╟▒\□□╗P«F+┤║▓□╓·%ÑZ^
3[ÿ╙□ñ.╒≥┘Wj+]░N╡║8□¡uÖ¼Pµ╡║vâQ□.╝α»é<┃□ÇδT2╣║DΩ
□mAV┘▓∞%T╬□÷╖□┤2Ü°ï═╡~!»T╩WΦ

h-"\□n°w▄□╛─*r□π╨°@□%┼ⁿ╪│HMFë$É╤^ñg:╤½å□åê├□Q┴σ□<□Θ-½#P░:re=└{╞_ò
╡¢₧W.├≤      ─╓□Ç-□6m4|□≡■w╫▌╙∞□┤□Di}+□Σ≤╝ ╘≥0─

+├ JÖî°G`è'└la=e:2▓□JÜQ┘°┤ü□Æï¼CF╩╝σr╣c#□τ>¥+kí

·½[Üσ□A╚^I
àôK░f╒hZ□░XT-1.Θ₧Y

çδöBH□¡≤╞
VXvZ╕°□&£ö¬=±σüB?┘╘Pí╞╪Z╞:□ìδ□E
½□□mnúJ╔¼□Γ╗⌡M█∩«`±ñO□-~□╣□z╟æ~ê∩╓{□P¿·Ä÷⌐-
□h□≈g▄Ü╛□g4r╡╛5$▓wδφw▌~╫^╪╞├S□öε)-
)≈□,[!≡{╦%□ⁿ╞*áít╛│*tτê□.V»─σ╓D□j║ΦΣZèW5⌐╝^ ╬≥éz├Γδ^·öÉ>□½M░

e┤èfá╓□Tô≥²-,P:▀r¼RZ u9E?□═Ä┐□P╓±δ⌡□╡¬H╘╪Pæ□╘£&Θ═ΩªhS$╧
6eV┤)d¬nS$O□-█ç∞ÖvvÅ□EΓ}Θ}╟╛ëë}

n□üb╤ùé╡Γi┐u□ûèÖ] ╒rÑ╗£*╖╡EF□+$▀_&á║□j╤ì¿lB⌐X\Ç□°□êN□╡Γ□N╫
╪+⌠δ¡□╗Qï\Ö□î□{Ü╓∞¬ó▒╚3÷╬□╡▀òQ_┘▄╡ûÄ□≥╞+∩ß3°╥H□□⌐σöj╖s

╖○╓αû╕╜s╪□O#╝;8Φª-âtÑ~≥□¢░ÿ_kns▓>{╒□jß╖┤q8L3=nlæq`°

èô─┬;ñ&╨ƒ╧⌡s□rß□ë□Ñü≤
┴╙=~&æⁿ□W┌□£?aí≡3$ú12∩ ¥╞á
HàAì□⌠.F□W.H╣F1□êe▒╧*4rÆáT`^▐+∩æ┐□≤Zì|í□I┘∞┌x4¬

╧a²<mW╖1j□ooWw╬a□^╔πΩ▐┴╟á:I½╩φsW~1□«Qpσ□°ñ]ì¢ⁿâªè╝R?m+□ë╘vMYÜ[ÑΩ√ª─ô"¼╝□~
Σ2┐\;µC"¡∩°¬o&éO¢Aq
R─O╔_Id L¬▌]⌐T╖WM|□├-$T╛εT║
╝·╜£□rσ□ªm4~

├■□«┤ $ n■╕ ò╞°q∞i

╓«ⁿ    +àƒÑ]Ä&ÆÿC.ƒ _M\φnÜé=I□äUíÇ░BÉU╘ⁿmtφ□!WAΘq□Θy□á£@N╒╧B±`□"f─çàyy▄

-êëV╟a█yß(5▄H'□»ùªµ░≤<â≡th╨Kk∞âÜfQ⌐E║
¼f⌡k░╤T└W╟Sä□Tû√=□ú□k%□╝ågA╫▓7½eg╓)□jkO╞□N□±Σô□c╧åúY3*□≈
9j)ªó9□C₧·s\aë□ó9□J┐₧□óGΓr%àv-
╝D)σ8╓□5Φ│ΦW╡,#┴%Jjß╫hXª□╤□├Ω□n□4kßq¦□`πCvè╪\@σr□▒Çl╖o╓gí,µ9∞0»Γ□ïsÉÑtâ╘0
1è

╫╤ûMß□t

-╧□'«X÷╜`µn\\é,Φ8.Çr┘û}\0╫2ΩÑ╪╜╖╓╟¬┴ñ□ô-W□╚àWöµb"Æ╢1╥_Γ/Jt╤Bñhò-
\bìR╡YâF$,âg╜~â

W░ª·┐─╢[42Ö╤╧"□Æ╟₧├ªH□□□yJ≈┤╘╨>ç■⌡,□╫⌠µ╨o±:ò╥e5|&æá¼_

−*<N□┴□#=╡□$`1□M\~Ñ╛┼□□
φ+lnjµç¡{¢░).□≤║οεèÅ┤╗âú╟Fsö9∞□v┼£ö╓¿ε┐ò/∩Θu>ƒ≤ñ£╨á╟òb▒□`ö□□τ   Ü@

ù□ê□¼□°ü╥□4╓░ëu□┘▄┌╢╧╙4¼╓ε░┘└∞6ü┘]ö(□

) ┘i╨Ö╝ê>□
ïΦwx
ósRL⌠»=ç╪² ╟ß╟3ΣæZ□!┤p╒kh─╜«ÆeWFñ╡2⌐A¼éa□▌ⁿû

G6□5·Æ≡tD╩╚┘∞~ú,╝>╙╗⌐╙■Ln≥█l□ƒ#τ░·═4■╟≥ß╡Q┌Φ5τÇ ô╤R\-í$8

~,╜Θu'╫▐:α╙GXWΣï>□╬□Il├╛çYH□ÆDr╙f─?ç^α□╨≡τ□
OQ·║Ω□■$░t/>½5öú┴oJÅ└≤S"ï╬O□╬åiÉ╤R8æêî□ô┬,8)≤l,>⌠hl∞`┐.□≡□iqS│LΘJ┘█║;;╝yN
!ù□(₧╙▓□┌`√╥├c╟¬□pQ*Zc╥26│Jb╤V╟╞åMnÑ╓□Wz┌□□_æ╨@çrHzZ╬µ
□□□Cgy╡╞╢ƒòπ─┐2-J□K╤"c-√
jMè7╢c□□╓W<│S/ìm∩\5Q┤╕·m=±è-@bτî≥8≥Nk╨L╥M□¢╡═ï²ND?Kª╓■╓
8ë╕▌¿A*R!]□≥qûp▐Ω(ä¡₧H}.□□¡±#qñ≈\□─*╧╠Z5□+7ç■□oB¼

#'-zå{æ├8╬□ ·╝δÉⁿ╦µ#═âW□┼┌'O╡sL□}÷F├"èÜ]÷╪âVδ┬S*o[(┕¢q□├EY╣ⁿ@∞∞Ö≡╪ßíí[W╣₧├
3Yk╨┬`°╗Nç=ΩP╔□ö╔δ┃3Ç╖╧}╣|t,□,m/Ü┌║┤╬░□╚Æ
√!·▓─éΣæ─¼Vï0 æ>╟k┬£G÷□╔█=O□ƒ

░□f

□A≈eqÖ~>}⌐┘ε╙H₧ì╤⌡ÿΣ¢½┴Φ╥□)%%n¡╞ñÆlfTîrBn⌠█φ~ô╝Å□/{┘∞Ü□¢╢P□î|Q╧*IJª╡¢□Äp╚
╜░GJ├╜Üûó_vç┬Äx~╝═.òQ□+┤╥¢░bgü~'æ╡á□□ö
í┴$TêM□41τ"ç<√Mç╔├b╫LS│□m=τ«╜⌠ë¥□Rc╖ü▒□#û£□╪M╕ì3RM┼Nt¼═□▒▀5╞Öh╥√<
▀¼┐nUiÅ╟╫╜╛□%h□`□┌□╫=]╧□□·/`Φ□·╠│ö≤_X⌡Aa╚»/9■ó1╕Bδóª Cn□┴□╞□\
re╝ùÅ%zzc═Qaz□□|ÑöÆ}7□□f▓í`
?╘□≡□W5ü□SΩ²┬Wúëe┐□╡]=²µ74τY√dB□4d□ß╧σ╜≡»D┼;5"A□Φ╖y=┼£δ=Σ:W<╘Ö□Ñ□å

7X□+ùpµë╥Γbß5¿║·5äC\ƒ2╓W·□ì▄└┘ }ü
¢v╓í≥ç,Θ□ù▐D╥ô▄₧+▀dfSë4█╡&eXÖ╧½_c□□ⁿkK╞╙a│╟D√║╓□kÆy□╠?è─╬·4M╓d□□≤LαÉ╧Σ:Xƒ
2p·ù□Kv╫ÿlsrP*╧└Sòl£(ò

áN6q&
org╛s<mZ4â□□±SKF,î□Cè└Ü=□╞¬□÷╠≤□óG/└╡6αYΣ□{Fu╚√5╤7«t□ït╤"□□(óδ>62~|
─□Z{╫°Φ▒æ└Åi[▄δL8T┤5εφΦ┼ 8pxm<X988p╟Üh░ráΓφê▓ªH'╟uäM□¿□¢╨?b□┴êá▀.L;ô□□╨
°m=báÆ
'AB╫m=¿>┌Ω║M╤╤╗εEb▄└p⌐δεÖ∞b├□êôjr!╚9MIobW□┐╣="╕nk▐π╚□ñíδ-<╝&*╫X4□Çàf
╪zé╝)□j□Éì'□kn+A╧φ }┴τ□=7╘╫àHï¢±X□□╨ ^aí╛8ΣQ□□□
çæÜ≡úë·K┌ΓΓΓ┼R9□Å⌐C_╟HJ*Ñö□Ñ╥lqhZ%┌□≡{┤î]/┼QΓ□ï□\IBªu□□╛╖X□:└/╚ ⌐L
┌m'É□□î▓□Y²□ñê>z▐□uE□∞□·⌠,ó□?£□■Jìæ-°≥Å░E╥□êzDc|ä□□
ß4□□o,sÄJ╤║A-─ä─Γ2l}s&üæ┴╔█è₧εöS□F+ô╚□ßé╫□
t«ε□·°¢≤╬║¿□@,ææ□k0cτ▄ß«rW□┐+QJ▓4úR╪□:3C¬4î┘f▓□Lí▐|┤/fÆ╤J┌µ╘ÖöäB¡░Θ═□â1╪+
°┐τ╨ù╓3H□ë╬ ^g□2ó╓⌐hτü└ô,²ñε@Σi⌐(·»□}âùµ/╝╓□Bσ«rs-
«PîÑα=⌠eÖ╥∞ß■/{_□-Gq¡[╒==3=▌│∩3Ü}òF□ì4Z╞Æeìm┘≥Äwca┘╚╣l
d┘h├K╟û╝Å8╧$

üÉ°æφronr
ï□î0¢¥Ç□≥Γ@-|ää=!┴l≈▓$É□╨╠;U▌3□╔6□H≥▐≈▐ò╧τ_╒5▌°N¥sΩ╘┌Θ₧j≤╓K│:¡N╫J□*
ë?WΦα░▀□p·8N□a╙π jy□╫┤Θc2╥
é#)□□âd

|)□□9╜ô<□╞£f

p□□C5GL░╢║╟GÄÖ□jX█π;│□U áuφ╘

á¥RT!½ÄÆà=□iJ╫┌╤Φ━╡)S

ÖæFíⁿ>┘9║2Q∩╒▓▀Wqé=µ⌡╞∞□╢Wí╪╩jl□╬┬¿xâ├û½$╗Yp□âÇƒ▓9

α²¼RΣs7√²°r^T▓dE╔£f?□igúU≈·□5╣ΩjK□I1äÇ┼╘lQ½ZZ,¡dòaTY□vV╖XXw|°{á╨□i├«Γf&τY
òOεQ∞┬¥+)▓▀÷P□┐Ö□\╒╤X╧–
2+1│ê∞▐□ïW╗E÷áJí▒╟<╛ÿâg:□▄┼≡□ÿ3B□₧┘╞0□3é]╓ü═Qó□ƒV¢{y\#v√╕FDQ╥□M%┐^8ó·yö∙2
Φ°□¡Ç╡6>K╓┌$ΩÄ»╡±k╟`ò
Klⁿ:╠□!í|gHα<;ì┼Öâjϕ╤q3ù¼¼qa²□S□╓╧▒¿¼□╗╝┼╖J┼\°dlU╤P┬íaV]□δ├rO,icö▀╛Aë¡ò╤P
àC├._N

∩ï$¼ÿ√□≤å□╔-
┘¼²«╗ö<⌠J¡□░εCì^Ñ`ö╝Ω╗▀S≥□TΩä≈╔wü°=µ□Φa□M'·┼╗G╒jì□\αX└╞█,#°▐¼¿▒òφ┤≥·¥|?;î
Z╧▌xç□□y=▄X≥□└°▐,°□evz░▄╞p
₧╘░µD$□sê╧+ÿM

oï□âσ□¼dîF¡□

≤□å▒{

Jå7□sÅ`<¢|5┬Θ▌62τ
÷$}□M@"▓Ég╝->ªΣY≥≤⌐□╬╙ΘPI╬ào(<8û□PⁿR~N,w;ßúêΓ╜▄Å╞·ⁿÜ≥┘|□>{¢ù.mÜ╛tΘ┤▄□π¬┌3
sα□ε□≡·S■□□q█╙-▄╙□□"3éⁿ╙╨|⌡╕└E▄ï
sAY╧ ¬0%□ⁿ~ⁿí╖ës4~□k£ ƒ┐┬┌┴.╤WÅ╫·┤£6╨@□╔@□┘╨□╨Γ□₧┴`□h-
ZÑJk╒~tQy&¿╫□3σ□╙Bz}êf»Åτ_├╧(·¿l${e■ë╧÷O╟□C□H╫â@4├#ô≤àót╞╔╥=ñ▒ù√□□v▐┼ù╒'□
i□/z╚▒¿⌠□~4û÷èx
»%_üjyµ)¥□D□═║Åδ"╥□·æh□·Kf┘}·≈≡□q□┤╧-
ß┘E¿⌡î┤□^Qß?£╕revµ¬□┘â¥┘╓ï╫g[I╢┌î┐╞dÿNñG╞#H%▄ç□Hü╚╬n≥NY□Θr║]a□╞²ÑN¢═ë┐/□E

□╨ö¼₧ûIÆ²  ≤y╘╟°Ç╙[îèφç$Ω Φj7╗Ö·□we┴ÄVF ┬□□σ±r╬□m7┤â□╧╘é¬₧>»¬èY

□òÆ□µg╝5ΦrC8rêεJ┐┐╥¡╓⌡≥ûÉ╤□┤¬▒□ô╨Ö5∞Wï█δ>\°J97sb¥╒* ║║Iæé□Ω;l□Fÿ;Ä"ú(îαkÄ
û]┬¡□┘╞╬╨}□KƒqT)ò╥7¢ì□9╓fÜΣm*uO╪<F□□■╪□SV╘□K:U□≥½Hû╫ï¼j@P□□QW╙nP□V(Y╟¬□▓/
╫∞\₧╝U╤é:╨╢√PÜy>½]║*╛tV|Θ╥°,û∞Φ÷ù#H╟▀ÅwAÄ┌Ä»<▐b□rd╙½-□«JÄ`╥=┴ ╖p¥c□□-
µ║ñì▌`φ`ÿ┤╖¢t╫□□╤°#¢ô7xcBπ_^□j╞7τQ□~ⁿ¬▓zY÷╓□{∩▀7w`MúÄ'┘èá□R
╖g6¼h≥D┌╥╡]«7ô┘└¿φmΩÿε╖%f'δ/i»□öéÜâΦimY╖k▐·â□k==kª╡^╛ñ≥α▓»≈═┤z<:â°*α⌠¢öe■
2wφ£Dó╝╓¡╢E}▐░UUV;°"8╜╥ΘÅ°U╓¿╧-░□-
æÉ│r┼«┼═[.Üªcö⌐ï.'π╙¢╫*f)*Q□JóiGÆ□├□│πXLí@U#LΣ^[┬□╕$Θå|g°-C°µRE'Æ□!Aƒ">C□
□Q╪F▓ÿ=╫□d┼□^;a5Æ±□┬□┼,¢Θv╤¢èÆ█▄co□£□□'Zt°{╩▓T[⌡┤·σ·█
Ä\è╓}
□lK╫?Vp├╟TÄ╩░┐6YΘf~I6éWè&φ╟O╫0?-√2°─M□æ∩Vñí□⌐√P9sGVo⌠□□
Σ0¢Bùö□G≡╠├J□²¡┌╓τ┴I
w√▀*nf[░πy□╓H∩Y▄¡ä╢l¼□╝UI▐ò≡╬□vÅQ╓¿u"q^G╟□¡v¿ƒ$╖±7ùI[▄æ□Ö□L≈Ç└□+ó

ƒM»:ªαX≥═ÿ·π{□Gî─ÆUα╥║┴ù/F□∩C5╟ïYφÆ□╤%┘ΦÆ%╤,½sÅ0∩â/╧□┴│Å67█□ê
╧_QI\╪∩τµ»úNnD6┘æÑ¼ZvσΩO≥Γå╧Θ─╟□|id√¼┴╡═FpbâAS│h╥╠├ò═₧╨£₧÷¥Zô□í├(^╤╘╤□.▄û
¼[7?-¬Θ■4╝í╡s▀é⌡_▀ö÷6¡Ö╓╓╖0■ì«onm░╕╜Fô°╝,□q√╩▄5m±¬·Θó□╖k█+□M─ü}JK╘τ

╪⌠╞h╪=ε└Ü·ï║┴□]y-√û∞┐ÖI■□□ ╒□.ë╣a▄□-
S□□□≈M□~≈e▀▓Ü□DOMä▄╢□╥δ┴¬É╘≡°6à½rv▓n^┬<áwΣzÿ▄╥≡e□▌╫Öî√½c!□sû╫≥□R≈±πÆ√Æ╒□D
╓2Eu1▓~τhÇF╓½ÅûI[

Ä╤ù⌠\ ▓6□"k□D╓□┘ □°^¢╥└1*¡≡é ▌Θ)≈Eô

╡ƒ╫≤£é╫kX├Fì┬\-vå£Fσ⌡<·▓□╓xDè┼·╫┘╣èΘαìC─□ σ□o|åxπ²╪äÜ□Å3Y╤°╡µ∩53═╞f#qNS╓□
▌·å┴Åw°1q╨─╢□£*n₧I╢║/D┘╬≤åYⁿ7;Φ▄╖▌ ╝íiπé□#$û□¥&╓▓zZu{¡╥5m═î-
Zú┤qCo╒╝Zù>╪□M¡╚V≥dA

δbcπè ▌∞╥k.«r ╒╬½j^╫Ω╟ì+≈¡(╫Y¥Fô+^û≡Y]VK¿╬□¿□¢òû░╫□2)¡æz-
$Jf°╫í4□=V»═h≡z,┴┘▌m5╥gVï¼2æ]
-□╬□□╤)ü*Q□M?Æ□ΦGÿ₧c1ÄC╒#°≈Gm1wj□□│□EU□╞Tá[#φ╒□½□Γ¢Ñ^è s/à□ë╕Θ▌□²╡é
'□"√▌Σ┌⌡□¥□L/Γ9é'9½║nNà±ZòûWµ63╣□p□╧¿I=ñæ╢ε╘<ñtTE▌É
█p╬Σ╥½8≡¢▒o≤÷□ú═eêù∞åX¢U$a┤UæX╗∩^»□┬¼`6ìα½Äå║I¼5H[ç╫R╖}äè Iü╢qBá═BΓª□╗Ö╚G
J?╗╫Ω□_åò├½jï'ßìV:T?é!╟mpç╔╗ÿa╗□ûó╠└&
╢%#6┐M»<└┌8XOi╘▀í▒EIª╡□<{□x╢Æ²5▌≤¿* !╗WÄ□╙ÑÿqÖ□3≡s─"□/j┴"ë┐âEñ╕▒╫b╕T[V□

°╩ä▄□¥M½$╧ÆΓj┴]9╖.=°┬t⌐╬û√"ô{□δqó&u{a[┼█U÷╨ÿ»*∞32é╒¡'?«□╟

&q.ù*dd£□ΓF┘}É'▐ÿσφFA□╨╙¢vZΘLw&²╓▀ÆÄà□╜å▄Y□┘╫√I:□qç∞z⌡1ûS`Éƒùv╓}ì╜òï@╘╪Fó
╞Φ9QπU)j¼íQc²qc!^¼Æ&3□¥╟V@¼X}ÿ[²⌐≥▒┐=P▄:}∩□W╡⌡»╬ÉÖLo□R
╣□ô▒╥┤f▓░1èù□ô▒Ä÷□¥è□□╡mFτP█åol*&cxx┘┴φ¡û2»▐TV□¿□╣²nW□r▒╣╡e*[╟τëX╘ε┌╣□┴µJ
°/ΓS[ó-G└ná╣╪╝▌
¢╖,═ΦX.╡T╬┼>TDΦ□9Æ¿E≥╥ß┬\fÆ²r□╟c□Mb□w
,╫¼Æwû■╝Ä⌐êX!≤╘åúΣç╤|1√"╔╟ÆM$-
3YI>÷□╝□_¥I╜□╥`Ä<□≡╛öÉUU╣Ö[╓ô□J╤,Äq╒╟«▒{Q!#π∞╨Åú≥oò┤|9µπX□├╪äStGM=┃8¢┬,*-
an<Ωu□╞æⁿ
≈@Ñæ□ô}Y>┤╝\o└□g
/■.╝▓i├·N)░$╬<Æ&█□oΦLáNL~πöuö╟q94S╥□iαS░□ìmΦö╪tv«²╟&à∞ébR¿SôñÉÄÑÅ₧$I!Σädg
12₧p½ⁿ□ï║□kµbí□tv□┤â:p═□≤╪ñqvéy
íⁿ┘cΣ□□┘╨KCKX╧Æ┴╖z╥αô~╜╨Px□╕d4fì╥pt└τ6·├□22╟▀Uô(

╧DódÇÆ□≥ƒJL□Ñ`û⌡─(ZY~É[╪■├e!å\▀IZ^√╖çëB┬█d·L  /úΣ-
3╓∩¥WÜ≡Γ╜]▀▄Ro.≤□,εr□╔x▌«Ω┘σU≤╥%aó=□hN@ÿ≡½,Q/d╝□s4

K╢▌ ïñ0í«_┌M≤ë□┘WΣ(±Ä<║,¬$V%░▓

½

XÑ├J-□Φ □ê□ñ@≤4Ülòó╓ åG╢ⁿ╗Y-|hs'cT╖░╞[]î-╟▓4╕╨═°Hx□K<□±%1-`ê┼Θ/
ëë□bz□KΓX□G╤p╠Çc:□╒Γ≤êD%·╘
J╢ö[æ ¡²╘Sε+$UùWÜ9úÄñΩ*¡□▀╚9□│¬╙≤□û□â¥ñΩ╖π5x0] zß╣╣╫U╬Ωÿ┐:□43ÅÆ\¥╝x∞π┐╘0_
□╗âΣ□-µQ|
≈"úR@*┴▌

5□µ□s□≈G¿Q╦5!µQµ□▄oíå'50¬=·□╛àY□3╡□fj| VCgΩ╖╦vq{σⁿ₧N╒▄'Σ≈°□▐□°▀░∩2ZEÄQj°ƒ
@░÷9=!ôΓ□╣ƒ□Ö╝èÖ╤ñf┤^°┘i□ò█□□âY╙çHL╬Σ#╟□╞R┤□¡╛□Ußw▓┌╣≤├s3ß╢s├□Vτ□aÜ▓eH╫≥├
·¼┼9»>⌡ôÖ
*~α⌡r3w≤w□mwp_(]b□∩▌|Γ□│a≥xk(╛=4:>╪öd¼1W╘_▓wnra&└½□î ¬<U╙#¡│M±ûDï
Æƒeï|v▙T]`ZÑO¡╒0,'V╬XV=k≤L ╜à±╢jù'█┘Γ□

z^k÷;}e:ú.Yeï8EÑ╤c┃║⌠╩t2\a░□╝1úC»□□V╜°nAe√F□├z½[╙∞□╩°ÿ_0m%▀äαcà¼≡@Voï9Æw╙
)ß.=▌
Ñä□ZJÜ'°'¢f~í□╖4÷(╜□ù;
N╞Azí·Ç5□╙ßx&á∩□

╣│ç≡Φ╧□æΦ╡ □π^╦Öé-{¿╠Ñ┼□╚╣□Σ╛O.□├□r?'□ÄΣ?─╖1▀ ╧
□/;æ□φ╞k□LR┬½╣kσö░≤é9a╤╤nS█π>o98Ü╓&*░RP▀¡□L~°7lµ,*êàΣ■□■UFÑ□▄Nú├$p¢
□å┴

°T╨g0 âÖ╟¦
-C├─╧▌╫jτ-

╧k□╧¢□nbE≡│x╣□ë⌡⌡σ(eH1û╘-│Q9╢ò▀Φ╙δ-╚g≡1╙▐÷ß╖}╪τπf_²░σq
c·□w□då¥DπW⌠»∩ñO├¼∩$ Θ≤1τ≈C┼'□■å »▒- √αë≤Æ+□τû╖gΓ&╡áß▌σM□╛J╖V□l,oà□
²□X[vFE¡°.ßUs<·jR)T╟XÜlΘl⌡□âΘ`Ek╣σdφÆ·2^g4à}~ï╓á□═.╜╓c╤p:°┘∞╨*╧Cεê▐
¿τt

│▌¬Sk¼&╤û╙╞╗jπ^╡┬□Kâ?□ ╗▀╬▄@²▒uÆ?■≈¼□╥┴çôÅ'ÖΣ-
▓S^Ñ╣v▄);?ÅW¬╥╟v¥ÿσ□▓W╛í1≡Σ□H╒□¼)P□⌐h□j│□Cε%µ□╖ì□â]àç8║8╝╧e⌡:¼Z<_□·P¿4╩▄ù
╝╕,□▒0┬\ïoπ\□
¡0╙«°╤qÿÖ╟·á╞.╫□ÿkÖφΣNÖ┬AjÉ□mA□èK□KÉ□Θæ¥╝9
UúF╘è┌╤Eh
┌Ç╢ó□ΦJt5z:█╖t[∩╨▐╟«/L B|τ`σá ╥═ß═Ωyï─E(█ªh3ñΩ,u╜_□▄╝¿¡««m╤µ┴/⌠¬╧.^τ([╨?╝
dx╓₧½µ^U{Y_Cf½c╜w╜i·j█jªiåråª"⌐K

_╒╥~⌡îdr╞Ω⌡}W
½ó[6□ú¿·L⌡□ún□□·'Cƒ

ÿ\a·,WÉ⌠=□¼»K╫╞Σ┐f·»]■[°\5Θx≥▀╔ƒ½l□Å#ô°□┌cƒJ╒╒Ñn"≡A║&]□&Ñ\c-
ⁿ╗#]Sôfû□□sæ□µï┼s╟εL╒╒╓åqM]]
-%µ╓□ⁿÇ£}□)▒▀¼%εXô╬²&¥«y□

≡-

PXM╕φ□└□╓V╫Å═â╥═⌐T□πùO╧⌐ápû\÷█║T]□□Σ²R╟/ÖÅ╕│îR}□æÖy□≤□kπ^ücΘ□K╗ÿ╟Öe▄+╚ìÆç
9▌□╛+½╡≡<▓hoτ8úΓN╟

&çîL~ⁿ▌<Åö<d□*yVì▐□Æ_╤├,3δ■·₧`4□∩┼ª5û√□°□╜ë╗9□╕)□Å╞sÅδ⌠Z□≤¿╤□ö$·%╪P

(H╚┤╤πçσπ_*éTΓôàcµNzⁿh±°Yz|Ü-≈1?e5⌠·ƒ╙-□Ö3╟!ε,□ú∞}□π╗εß8½Uσ║-

▀□UFⁿoY^Ñ┤èÉé°D╥Yûv6-E□yú≤ûΩ┤úZz□□ÿ▐8~╫╘.┐▒xΩ∩⌠▐

]┐□YSE░,□□∞"╤-b┴⌐]╟≥µÇ+Te├□√ù▄╗.□╓ßú6ù□"â└?X╓□

╠.{É□!nj]6êj▓╒@└□╨fφ□¡Ngïèé└å]▒H⌠□m╠'èJ▀² «□)ë└ΘΓ<ï╔¢m□gjM╙ªQ
7«m≥╙╢┼≈2ÆùÆ□εΓ│Θ□≤

╟┼Ωâq╗ÜMσrU,oë·jóJε?□é=-è&]□≈╘□ù│f╤«┤≥Σ¢╨»~à╝└F░pv-sX╘⌐□X╧isï╟Hå
{□HµGQ≥⌠╙ï`□7~ε(∩z@;┬ΣÄ╕PÄ□Ö'=□U≡-
≥¼Ö═ª*y□Æ¡▒≈{[∩w□âÄ╟.╗)]╫p┌╓░vv¬v·Üî[▒e√íìU./≤/-□8ⁿüM
kª√saW≤:Ω█╘│É□┘□Ox

)æªhß╥[╟≥3@8□╧Lï┼3Ö8≤ª▐h╘3╧δîF¥4J¿O"□╔L⌡°9rSôtE îBεè⌠F╢┬╜MÖ!Ö0ÿ6ô)wú#X╤PW
y╓dbâ╒!□≈□ß<÷c┐$%±oö□K≤AΣ╫┌□□¢≥~ⁿ,¿═Çƒ╜╫□PΘY!µ"φÖYü╢ùNâçÆç│□εÖû-αÉ╗CƒwÅ╞╪
ÆQ9■ö?□L□

gΩú°T▒tm□wxs$µ┐┬¢≡┌°C▀σ¡ε░s0-öu Ä╜k□⌡zF?÷-=>□

╢░#╖□▀ß

┘àPP▓9□ô0
□QΩ░Ou?([ï□°Ñ¼□i}□(ò□∩□F2║4Ñíd┬k╛¼Ñ▓Ä{Çò<□wg┼▓]KΩcMÖX╪3╥╕╡óíu╘□MÜô5│¢□o┤n
¢□òèv┬τ3┌<[ù{c6■àlA.ê
¿□╒íàG├åÇf□┐|□íè□ⁿ┬æ@
$
╧eì╢p═ëô·'⌠/ΘY╝▐£z└E╞□ç╠╥╕ó/□-╝╙+]-┐φáp□Yz
┘d╤I║n#┴ü□∩├│JkE╪□╢≡∞\{mU□ΘL╨±ö7aΦΩ_╨>úbû└²╗-Ær╓47╬f^W
*□╣   vGSMε5┌╖S-□├Φz;□w╗"]■vZ□EY╩¼:B■ⁿ G<ΣA□≥Σδs╘q₧;óvë╘g₧ae-
M□ç[ß┘{╔KΘ├Uà#Vs╫mï≈¼J&Ωδ*□╡═Ö□~[í□~≡úΦ╥▌+±#─ms¡k□.║□ é

îVΓVªL▒□yæπ░h□É¥à□Σfg#ûmª▀o°'▄≥'▀□╤≡╙╪0>D╩4║▄-¡V/\w╜┘ñ╤▌"□├□╓~Aïw·¥N□╚┴¿ù
ï░□≡h╢¿òéfl╪Ez▌åCLÑb-Üàóç│ε□v÷╤Pê+□□Z₧□2Xß/╟╓□₧□/}□4□ìû.╛óí┬sg□W^
RP°K/¢ì⌐¼Z╥╙□₧æ≥├ÆB├½▄σu╛÷EK;30╦iÜµ,«¥εK╟<jìå╫ïßÜ╓≥m;ù-
Φ▄═╕Z╓═≡ïF│Rφ□8}zô~fª⌐╒ß≈╪gg*°¢a@□╝^ü7□à⌡]
╖Y□ÜnΓ╡1\╟╠W,å□J□□□├Égg≈□]æê╩u□║ª"è-
╞{Ü□□FÅ£│□^!L╡╦Æu╘|√¼@4ε╔²A)≥£VxB⌐wU°ë╫ë□d^■□c£~╝¿╙ë╫+⌠-G╒4□╛^k╥(,µ\╣□ƒ╩u
□m»EÅ0"╗ |╠uD+┌-d[æ
l□)╪¼dµ3π/ñöφ<┘╠ó µn╨è:a∩5aìxP□□╞-█□±}Å╒Vµ▒Γ□s,-
Nç·bê□ⁿ╪36╥Ω□t'πf{P□%

╖äF╪Σ1╖¢Kû?

φ6é□V°k□3╖└@□;°?»í%□eC╗□
║¢BMU>ÑZ⌐Q½\╤□o█╝Ekδ□╜₧╧╟ZÉj≥╘D▌*^├δ∞δ□m∞]2wφz|M·óz╖FoαTû2KÖV»m¼«m░╕¥µi⌐H
┌`6□<

□»□, [╓║╓¿α╓¢╙½╤âL#╖A╢▒7

};╬□]é@l▄□6╫~f□7┌Z}æhYε□╨'□úJ¥3µkÄ╖j─g±╙┐┴╖j□5éá□T└▓>Ü▓αàóPtsgìå▄

□╛:≈mómöƒ┼╣P□"╖ε~─░3æû
#▓o┐╡> Θ□Æ□┼ç&□/ä╫⌡⌠O□Φe½▌□PY□!+┬·q?Q□öi$"⌡aæC⌠QrU┴#╪w⌠□▀GOδ
□╜2φë╢%£.≥V╬ⁿ ╒□╟\u
bß:ó□ê□l└□ÿ╦□□□T╫| æ□▀ò□/ⁿöt∞┬─,8ç«²Dz|£╪□?
)tö-║0q▒OA_?ç>┐□)□ö┐□'òOª½╬Cg╞I¥á┤f□m£@╖î□»σwò╨Θ≤ô╞OΘ;□
╤□┌"╙±≤æ¿□□□H╦k/ÖL║╣□ñW⌡_□'├b├bc□╨ú□Ö

₧Kf°·m½╬z┬╢╟~¥├}-·°ô╚y╝D«╒n□í2µ╝t5Ñ'╧íⁿd≥╟□'/π²≥□·≡ⁿΣ√äⁿNÖ▐□°└¢□□»ô(T□Üq

▌□εÄ¼ïl¢L╤╚□Φδ▒╢"²»xδ□Φ╬r{·`·∩*Z*╛
⌠Qb□Ñ╥□oT╬¼ⁿJσƒ+ \╒^⌡`╥□┤=9V▌W²╚ □:¢□°hè■

╘1ü■u□²⌠s╨½┌╖ >T╙·wóδj«½¡¿=0ü╬╓₧M□%²f)╒U□=[?k□▌□⌠╧
Ω ┤□Φφ╞U□Φ▌╞w°▒╙~:ü■'╨S╙₧??5·Çεj6\ÇV□}0╝c□m□Ω¥>4E P╦⌡ )}ì╥-
@╧Pzë╥┘û╖)²╡σ»3□%╡L□°hèªhèªhè■K╥IëZ╣"UP║╕⌡Dü▓╨∞náæ)Üó)Üó)Üó)Üó)Üó)Üó)Üó)Ü
ó)Ü"Ö■£²≤╠«Ö╖□□·╙╠?=Z1δ;ôΦ□J╧L╤□M╤□M╤□M╤□M╤□M╤□M╤□M╤gá╫ªhè■δ□²]□FH5□a\°DH
ì3Hü╢σƒ□▄ô_ï□°P■□α╓ⁿ»□G≤?C□÷bñ□∞╧┐è□Hæ□□pO■1Σ`/╬?ëóPs□E1ô 9á□╬ëbW■M@o■≈Ç√
h=Aép■□4□┌·□α₧ⁿ"4□╟≤»□&(&)f(╢S\H±P■╖Ç'≤G□OσΓ□□═▀ü╓@╖CÇ{≥∩í5╨·3Çåⁿ∩□]╨╓□h²
E╘8H╛□'(&)f(╢S\Hq=≤`■u╘C┤fö Hδ□∞ç>v@+┐C□┤G□p■sÇ'≤

x□«Ω□y□AK□┤
ñz

mâk ä÷@y□ ┌□▓
□□≥╥□]·□Ç ▌n:└x■a└□┼$┼

┼vè
)vµ_□▄Æ┐□pêΓ0┼}·K□≈╙≥□è□≤╫□-□▌ε□┘ε□<ò?
x: oÇú·{╨-É╩□╪ƒ┐□3╨·_□□□ô□3□█).ñx( □α╔ⁿ□Oσ▀□<MkF≤╧b=σá°□⌠öâ₧r╨S

z╨A□R╝□x( W└ô┤|èΓi╨a□╫1Ç~■

╕ □Ω╜ └≤□ └□┼$┼

┼vè
)─╩┐
xÆ₧□èΓΘⁿKÇú [□╕╜□h╚?□ΦéVΓáφτ□≈σ_□<ÿ□□≡$▓□₧B*└Qñ┴q╨╞ìÇ`k£á□□öCérHP

╨!A9$(ç□σÉá□□öCérΦáÜΘáÜΘáÜΘáÜΘáÜΘáÜΘá║φá║φá║φá║φá║□ó□å(ç!╨aêr□ó□å(ç
!╨aêr□ó□å(ç!╨aÿr□ª□å)ça╨aÿr□ª□å)ça╨aÿr□ª□å)ç}P !α⌐ⁿ╟Ç°≤┐□□□Kφ°£≈S╬√)τ²ö≤~
╨y?σ╜ƒ┌}?╡√~j⌐² ╘R√┤▌≈S╗□=? hÇ╓□É1
Φ═┐□╕Åû□R<ëLÇ°É□8èx|□⌠╝□░?

>□Æⁿ□`ébÆbåb;┼à□□□╧â ├+ÇDåâ ├δÇú`═C ├¢Çåⁿ□]pµ!ÉßU└}┤■P■,>□m²□ƒä3□□□±
□Γ□á□tu□"î

0A1I1C▒¥ΓBè√ΦÖ□Aƒ'!┬h□O"□□╨/éú□í_;□√A╧°á¡╫□
p╒)hδ□@/Xτ□┤Ñ□LPLR╠Pl°╕ÉΓ>Φ⌡)hδ=╙CH H┌:Euxè╢u□┌□{@[┐╟°í¡ù□
·_□‖≥┐□⌠µ□▄Gk

Bδ°ß╟ç±(£·2á! '@ó½Qj»QÉJ□ÿáÿñÿí╪Nq!┼}╨úQ*╒(╒└(òjöJ5JG╨(H⌡E└■ⁿ≤□I□H□╕-

α₧|;{1╪·□└□┼$┼

┼vè
)B⌠□<Ö?□x* 0α()□╘╗╪~αs;█O"

`ébÆbåb;┼à□OR<
Æ⌠□ç7┘□┘□æè²P┴#òé'e□O╩t.¡béÇ,)2□Z├╥□VGÅHÖAj┼╙rÖEI╫▌rYü□è╙rÖâ≥
rY    σ≈σ▓□
s:╣¼F□▄ìrÖG~╒7σ▓å·~▒-□¡V□ù╦"¬PïrY½S¬□Σ▓

-Çs0ÆR□╡m‖\╞Heoô╥

R8■U.│╙ε╕I.+ÉΦ°₧\µá|╗\VB·^╣¼B═Äƒ╔e5▓┌f╥e-□□9╣¼┴Kïm
(ß╘╔e□Y¥╙Σ▓V┼:ù╙e□è└9,┬d╫D╞─}I.Kzû╩Æ₧Ñ▓ñg⌐,ΘY*Kzû╩Æ₧Ñ▓ñg⌐,ΘY*Kzû╩Æ₧
Ñ▓ñg⌐,ΘY*ku

2╣,Θ·╟╚ÅjQ□╒á

ö□ú-┤ ⌡ú□h□■oAâP7□J²h'┼.¿ΘüRJ┬'3Q/É-ç║¡É[

┬UΣ¿□■v├┘├Ç¢ßL-Ü□ÑìP╙ì«ä3.□n▌└c%┌MK~┤□8∩□╛C┤┼^(mÑÆ°ß

8g7\[h├ ö9à╥d7úΓQ#¬ñφw□ç¥p«┌φév□ÅMΦr·▄□p┤
j╓°C ▀@▒?+í┘ç÷í≈é≥líz≡úYp╝□>!╡]T
□√(±┘!≈╘O[□éO7╤■□┤{%\█Okåα¼═Tk~¿▀Fδ□ú·
□╤N□╝«ÅΩ╡Ö^▀M╧ΦF█íMóσ═□²▓Dàs²┤~Ç┌┤□d)Xo╝Σ≤AÉó□«□□-╟ª╜Θí=Θ)÷ú
■oç+$ Ñ■t╤6ⁿ▓¡{Ç#ß┌□τ□^╗ßΦJ(
R;

@ 6B╣ù╩╘OuA·█□╕U┌ö━uÉ÷Ij┃Å÷h□ò┤Å╢2@φ4ƒZe
╘□□□ó□□á|╗e[⎢╨>I║□á^1□\╗d□%□█)╫□Z┘

|z⌐~v╩R÷A═v┌¬─sÇjj\□╥ΓN┌□il□t+╓▐K╜åx┬6┘sëT█ß▄.h□É-⌡Q[□nZ╥Ö╘èd╟>╡_;¿n7╥3╟%
.φ□╤┌.z¥╘δ╦ß8I╟n⌐5cö█v╩a7╒├É<JK⌡]≡╛>┘ôI %╗⌠So(°h7╡5±▄¥┼▐H2nò╧□Çú=2≈AΦàdíß
óò║¿ÅÉ□░}B┐□æg□H╥E█▀$╖ƒ<Oäj:°ƒdtεÇπ═h╡∞5□»o□

╡□5&₧ U< ┬▐?Hσ╪L╜ô╙ty╤.πú⌡▄╪╢U÷⌡¥┼│ë7K^╨□τwS□·?□â5SQ° Ö(╝□$┘äΓtΣò╦ƒ√Q;⌡è□
T▓A □├ÜP5╨f¬[rσ÷s╝')√\5öwS┌J╜ê╪f7╘vü∞ÆÄ
\%₧╜T□"┴□*¡□√$^τ≤╤□Ωτ;i▀%-
□«#V]K█Éó╧n¬iI3âEk□╬.─èMr<'#┐ÆΩÇ£╖S÷è╥╪╜ôΩ╡OÄ□□ùn·┐KÄ╙▌4╩⌠╨-J╥mñr□¼<┘bâ≥□
Æ ⌠ƒS│Ñ╪ç╩O□ ñÖb3╒Θá<#IπSj╖▓╫╬Σ-Hæ⌡J¬°Mt<¥OgW╧=φí#¡ùÄ)iΣƒ½{rì4█━ßⁿ≥
-|~εÆ

ƒW╖ÑπCÜ±²≥£=H-
╖i┬▄9╣□π3σd╣ÜK|Ç⌠DΩïöA□be□1□┘Lτπ>□G║.╪S╔≈║&xò□□v╚(⌡J*□╤±"┼°=tnδæcï─ç£┘Kú
à}TèΓ}▓e╠╣□FHOIª▒ì╠╗-Y╧$¬ki╝∞û√P╚:□Z₧Φ┌ò╘2]┤╝□□r«╔qn≥HêOè
▌4N_I│î-j}b╒.¿#□┌□g□>½ûynÿ□;╥σ╤;--
╞3äé4ƒev·ö│ü┐l□ÅE□-~O╤¢/â:╔N□»æ2û^y□□≈εOÜß□^yßYÄXniqΣ

öΣ(Æ╜%/Φû█Æ"vfl≈J┌τ~y÷)Σ□R«┤U╢s┴Å%┐┌)τAR
;h.▐E√Y≡ö.4>╤OÄg □[□5╘E√N⌠╔#╟·=≥X▌$τ▀}T╓╥9│çfΦ□╘7e□/l[(»ÿ8╧â╡╥Kt┤╣d╒P:->5
?4╛╥)£}■ΦV9)║□t?·Ω^║RΦÖ╘∩é\π9╪°¿□fë□6¼Dà□□YÖ□Ä╗K<d']ô⌡R□█V2├JRoñ▓t╦3╒P╤ûÑ
░D▓a╡l±□:Jzï2□╞⌡D_·⌠Z-¥ßÑ^û╬4□}z\□WR=n £v,╠□Ct┼)iª╗Dé═□I¢πz╣

╬T2w

~B<û" f┌â┬î╫4!èK┘╪0-ƒ/δεúsDaû)]│□µë≤┼öëW
╨X!┘jú▄∩≤╧╢]□░h□▒≈□╘K√(wi□¥╖□■╝-Pÿ▀µí9⌠╙ï╨\8Z□│σrZ3Ωⁿ□Eù├'½ß¿
j█á&□g¼É?ÅQK¡í≤╨<8o□¥π$-╥ 7{▀□Uuφ╝÷Ö≈╠╔Σ1!

I└#"»╞□□1bJ
yÉ`Hb□-r⌐7C2!□ô╟tf□□#Dï@i┤H⌐FDJ¡╡╓K¡ùÅkòó□□□bDKQúª6Uïh⌐Fδ╡⌐ª╔╗÷:;a╬
□¡r█▀∩{0□ u┌┘» ^kφ╫£IéXè·
4╟□éB:╫«├ⁿÑX□/[□ï⌐ì□¼¡ÆrVP▌≤±n ╩□æÅù╚├;
Pτ╫si□T█+┼RΩ□óX¼ë*╙*╝»

≈P╦¬ÿZ□b6╡□¼┐Hñµb▌┼Tτ╧█/ñδ╥a₧àéi.┘ê╫╟δ╟CF%ñ±╗
Pûc╛Jj?ù·¼▓-Ñ>□b║┌ù□b└[╬□}U≤q√,□)▄G£_ ╛╧÷*ùlPDl╬┌/□e92τ⌡╪┼╘*Z!╩░d>⌡┤Æ¼W
l╞{[B┌┘^⌐₧╨ú▐p½r□Σπ⌡|ⁿ?w╪v□ä*ùèÿ┌┤╢[DΘgs⌐²╥□ÿGû+#M⌡F-iUΣ+₧Ü!|YA²╨╖║ê"▒Çrσ
RÅ+ç#ñÉóWe?□¥j□e1L╘÷╕oc╡

E╡≥□cD¡e(}ü≡⌠╣vßV╧%¢p^ò├-□^=86□ªL═║2[ÖΩ½
□┬ü║êÆ□□□□!O─□h╟Tr²~Ñ┬╥╝>□V*╜aohò╖63«╚╕,Σ]¡ö□╝ìUké^Ñ─│&╨□Qⁿüσ╛□Ñ&□\
□Γ%□^s╓4e<□Wg(□-□░^)≥4╓□jVΓ▌yü·FÑ¿⌐6╠█⌐¬≈à□□l=uüÉ2╠╖╟∩½±°□╤"µ
`úJ8╨□¬±*£εjO╙½45╓zCJñ▐½╟/«RJ|5▐╞░≈Z%∞⌡*▐åe▐┌Zo¡ΓWΩ*╡▐pM╙□Σ▌ú6j╜□Å╧
╬╠≤°}╦B>▐åGi□`à╪Ä°1î╡ä|uJ¥°┴τ_ú¼÷EΩòp╙▓ê▀½ä□╪«»q9Æ┬¼□o□ûl¼E□ä□╜ípªR□QΩ╝₧H
S╙□VB^∞à/ém╘ä3öpâ□φZπ
Γ5/╥╨ΣÅ°éXecSâ7ä9├▐□U□Véí□zâ│┼┌²■└jÑ-ì½°□é₧ÜêΓkT"▄╓╙

ï`□▒¡@¥▓╟╖£*V□èx¢#X╪╖╥¢⌐ênN□+
₧╞5JM□║Tσ═╫êF

y░/!_ÿ[╘δiPŸé╝□¼q9▐ √╓b÷H□;┤èw╓úá□□╘╣x≡╘╘{BH╟□╨□

¿ÖCm*s□■┌àh□n ·□ÖS°ë√W≡√□≤GB₧Zoâ'┤Æ≈à▄:□¥╤╤ΩA~╗&Ç&h⌠y├Ö%M5□=ßIΦIen(□ê╘G"┴≡
╠)Sj□5ß╠åíÆÖX`JdM0░<Σ ╓»ÖΓYå▒╞│bN□Sì'\□hDúc«│ìà¢éA┐□âç°e*7□Ü╨jkö&

ú□□X~¢□ú□▌□±f(╡╛p□âXuj0Σ├╘□╠ΓΕΘAWzC
╛H□½[╢║åz5□Æh.î¥@hΦóÄ╖Éqn▀1□j¢j"□<$Wa┘

^f¿□⌠╤Ωz_M}

│╒╪¿»▒╟▀ä±□û}á□úeóoÆ:4b▓c
_─V□I□∩Φ√p$Σ½Qâr¿□è┼í║«%
╘⌠a+8.°t□Γú°6░║╤≡┖j¡τQMà╤à▌A≈±ïªH□géZ/∩&╧S∩⌡□╡□┼╢
πW═╬□Γú▒R∩[µï≡9*«□)╫□°êßöà⌐3öe₧0r
4

╧□CNÿ(b┴█ÿ╣ ╥□⌠╓ ·<Öü╨≥)\¢é9 ]╟+ô╨╜□□4

x5τƒ□╧7ü¥□9JxÄ▀r3»□`ƒ╕ip<·qr#sk°JnJ═d□□W╬¥□ªüä²F□x▒□□6Zª6C⌐
ß─╟ç□

╞σ╪gnc┤□z□ï+üe8ß5rúxh▓-è│/▀
N╚□

□j|--8╬p┌jîx╘9╒τG╦LΣ5jz½Tè┘ ·╖ôêQ-═ê¬╬¢ÅµZ~;&▄2D╕q÷C╔~╞⌐┌6»+ñ«V╪□
"▌├

>ƒ√Ω╕⌠ÆAéM╪íp=
X¼zY□╝a~SD  ÷p□v<∞σ╙t ΦSg╒╧Ñ¬

xlR□4┬╥Dbu}áß
·╚çAS¿□╓x⌐é┌□╬ú─eà╖&2□`gπ□â┐╓G□oª□Γ8ì¡≥╞,║ìü□-
2Ωäε□├Xì□æ□«τk┬2»fΣzb:□Γ═ç#□L>t╤≡Ω≤E□απ¡¿@⌐,+¼Zö[Qá□W*σ□e
ï≤
≥ò    ╣ò¿O╚P□□W□ò-¿R0GEni╒
JYíÆ[zâr]qi~åR░╕╝óá▓R)½Pèτùù□□α╝Γ╥╝Æ□·┼Ñsò9X«┤

╫÷b□ëXiUÖ┬□□U□□T≥╩µ□TΣ□íÜ;°╕ ñ╕ Ωå

Ñ░╕¬ö╫Yêòµ*σ╣□U┼y
Jr+ö≥□□σeò□╪|>V[Z\ZXü¡□╠/(¡┬e╖□∩)□
QQ*ïrKJ¿⌐▄□╚╝é°σòò▀PQ<╖¿J)*+╔/└¢s□ÉYε£Æ□╡)∞T^In±ⁿ

%?w~ε▄□*Uå╡TP6┴nQQ□▌┬÷r±_^UqY)∩F^YiU□¬□╪╤è¬ßóïè+
2ö▄èΓJnÉ┬è2¼₧¢□KöQ%X«┤@¡à¢Z╤x□│p}Ae┴Y.·□╣%XW%/□¢93εΓúüïÅ□╛ém/>□°╫=□░╙ ïÅ□
■∩|<áz∩Γ#éïÅ□.>"╕°ê@?¢_|Lá}L0d¥ïÅ□.>*╕°¿α ╕G□86╒ƒA□□t├F8▀ïa

□fî□╥α ó$╛╣□l"╨}Ω╧□~┴╥h□+╥î╤ƒö²Æ·πΓx~iδù═-
╧≤□≥┐l■ä□╩▀≈e≤'&≥ⁿ╞═_6┐╥à·iå┐□ I□#σ7Γ   än4√xH┼┼w□╟éΘ□≡ïp□.íì`□╓┬═Lé-
,-~└□α~û□?gc`?Z√□╢□N░o┬∩Y□■╟ÜαS╢èÖ╪═,ü}çÑ▒6■sjl□█┴f▒(¢╟

▓□¼âU│#1Ña-k6,f╖□B¼═p7█f°3█a°Ç² ╘╨╤÷□■╩

□■╞₧├n╜ñσ╟^╜@╬?F╬Å τ□╚·□r~ 9┐à£?D╬â╚9□9°!τ±╚y□r₧à£ïæ≤BΣ∞A╬!■ô?╚y
rnG╬?D╬?A╫?G╬√æ≤□Σ|□9┐ü□Ok9□

╞p-ë£/G╬╙És.rµ□τ╜

9≤Ñ∞VΣ╝□9∩B╬□#τ'É≤3╚·$rεA╬ τ□□`q¼ëÑ#τ)╚·Z■╚j╚y!r^å£□æs
r▐î£∩E╬?E╬Å!τgÉs'r>ü£_G╬∩"τ┐□■*Ö

□ôΓ1«Gi9¢^ïß<□9Oñ┐L?
□k>

≈E┓

/ü¢æs□r╛□9?î£C╬□╙·□r~□9┐Å£□╪7Ö□9ÅF╬W τ┘╚╣□9/F╬╤╤æs□9»G╬╖#τ{É≤-Σⁿ8r>é£_A╬o
τ?"τ≈æ≤▀
╜Æî£╙É≤□Σ<M╤┘□□├9
9╧@╬à╙y!r«C╬╖ τ;æ≤ÅÉ≤□Σ▄ë£_E╬∩!τ┐▓1╠è╤:é-
┴□°&╗□9╧E╬ïæs□r^ï£7!τ╗æ≤□╙·?·O╛!ττæ≤¢╙·□Σ<hX,%□B╥H╟▌╥□├ƒÑå□ñ½æ≤\Σ╝□9╫
τÇû│╜%å≤hΣ╠□╞╫!τÑ╚╣□9oA╬≈"τ╟æ≤K╙·□rµ?cjf⌐,□9ÅG╬╙æs!r^î£}╙·&Σ╝
9∩F╬Å τ_#ττæ≤δ╙°O∞á$▒

i□;éⁿµI·╙y!r■&r«E╬ì╙y=r▐è£□ä£□Äƒα≤½╒2h╡╕▌9πΩ╓╒╒Y-
`╡÷uvα½│╧j□½╣/□┼□╤¿U□½!*^V#XMΓ║╫j□½²`δ[°■kδK¡┐k}□▀T╢╖½½½ùg7c+}¥¥}]¥¥¥╘Dç·
jß)µ╬╬µµ,|ÖM`6≈║¢╗║Ü⌐8╓▌┼k0□┴l□≥╢ét▀═│`&╩-
∞Bz=Vπá╒ÿU▌[=_▒à╗l□╪É7□Γ▒╝0[┴l Σu■R¢ª□óV|±╓─]∞à┘□fcÅZP%╘□╟Ω▒□□-
F╡]ò?f.□□y╒6¼╖««N▄
bò╓ß

Y╚RbV#Uç▄$  $Σ┼

8ú≈

╠j ┌╜{7□'+°(╖M?u*╪ε

║▌φAJ╔╬^╝╕╜▀φ6±─╬Ä╬₧=█äï°1¬╟VódΣ∞l┐▀?╨╤a53εTzíS┘τ9ò°êδ>½ìY□τ8□+Γ~∞∞#°▓□°≥
&╘□:¢╔▌τq¬═

6

»£ê━xò□dYnß -f3ÿ-═¥²╤hï═4h3fW
┐j╩wj□+┤!╧┌┴∞°¼╡3:⌠ª:╒rózAT▄φ□Nφ╒√ù"╝ ·<

╢a∞<ïvy⌐u5«K5≡yn╢I╟ª║·\?¢└╞≤æ╜å²╟òµ6╡Yû█TK

√┘□vCu□□1□┴n∞╔¬╬Ω▒┘└fKçt£□g□ì⌡p░⌡`½═┬l6u□π8╢ÖÖ═╥□>Å!─5□│
9=┌gs0[\□╛>∞·M⌡δ°>V}□▀╝□δÉ¥ó├□y▐fe6√╙Ω½.╟f□¢⌡Θ║║£□7╛,f░≡╛á_[∞ff╖

;»╧bb□s≡(▐8□ññ!≈w╢X╠╠bE□⌡G□7█M`7
□@╡╢ÆN╖─∞╞ß□ê□uΦe▒3ï□□□¥Q¬Z□-jå□é²∞}▐CïæYD(╨╡Öb□¡ì

├`Φ#.┘□
÷«ú□╟Xv╢┼□□╟a4UctE⌠ªΦò¡W╢¬╔ÿΦ≈w╪c▓║│▓8╤íΘôééIF-□╞hΩ1Üÿ▌B□□Åv□□Y²ñí)╒╕
═□□□

πP`8L□□v;╪φ2î┴≈4|{Z╫π¢îdev{ └□ε»#□²■óû~

Ä╛╛≤ÿÉRçö■~╗╟∞╬₧`/╛^}ö┐_╚z!½□▀v
|█·q¬Θ□αq6¼÷úM;:¿I□"∩¼╦▒[┴n}:&H╕φ√╤√²-

s╪x[▌TW7Å□K3g┌╫Lif|¡ó┤U¬ ·1L░£ √¥▌î«Q}ú¡□□h□┼!1GL/)RΓ^o□îì□
│╪Di║níV[∞├≈□║yI-╞]}1æ╥□¡╬ΩÑH1
:═┘□+Xåzlæ┴"s¢□¬□╒╫D»ëÄ╥FJLVî□╬t(RbBE20ô⌐ç╟èâbà╞EVo»□+²□nvk╦└Fs╦:≤F╬□╒⌠£□
┐┐s`@û-□É╒p⌐«╢□A╞Ç┴ê╓Ωq╪└aÜKf╨\ó╬&□[□6µp

L <▌zp°$^{2}$tδ

Ç├╠□V-@Qé¿å1╫1}8ä0Å#Ä9Γ{▓{▓{¢{i□□╛²·÷□┌Å╕Å╕╔kgú(Ω░2ç¥⌠■■ÄÄ°ƒ&□□[╡∩╖Z╫π«╩a
□çφlL╣i=jεo├)Ñ▀/[ÿ□□
□▌┤t5≈ÄF□≈7SΩp\a`Y-╟╚▌=□=4╨"¢╨@1æU□S┘)è

□LC▒□ò%&╟□W╘*3½│╕g░≈□□+Ω¢□□¬b¿Eè0çÜ╥1□b┤,w⌡⌡¿uÖ8≤╛ho│╕»ùOÄCAåQf╡ΓÜ╧m└ì`òq
█¼6□¿÷U»Θ╔èf⌐╗□-h<╥□▒┘1╘dIÆçWéíX3Q¼Ö╒Xô)╓□f╓a□è5

6≥□□□«▄□m▄k6τÿ□3ΩΩ:□□=f▄!┼□┼<f1A£║ëi□╨

Ée'8qG╧▀|╨\]¡ÄΓ┐j┘╫d□□

□□k╩â¡â¡□@.□□□

92yl╠Ey

δY□ú ▌╓Σ─₧⌠₧⌠ ▌£ ▌£.□ùƒO⌡G┌Ä┤=-?-
╥V&½SΓ□5≡d□ô ╒p°≈□⌠C□]±Ç#j╤s^□ê«o²□PU1qΦªòÿ[g□□¡8+ï│½┴≤

ƒ

□║i1═ny□∩╜▌ÆMΘ□│]╗₧╥╫_K╦k╬║w₧-î■a²¼8□ï3╟Fc⌡pìo

╛Ñ╬░┬□├±□ìôX£╞:Q¢╟l±·ê∞╚ó╢åδ□nÖ&mÖk├¼úΩ╞┴2□ûΩ₧□GTOsz[□┃â╧K├$¢┃iù┬mWC{□>╗≡
y╞µ─□▄□╥█ù╖╫║úgz▌┌εj2□.û–┘-■Ä

·|ee|╖·□□Æ□w6fcâ╓╙p╫┘kB{YiWOÄq7≈╡½n□□Σ!bτÄ□è[╨ß□ô2□╖□╕α4
□.s

□«·╓╥╓╨τ2ⁿs)⌐╓▀╕\\gå╒δàⁿ:7ΣYûíΣå□□3ö╝5!□å2╫□XI□B

y±Ü□+*C)±D□┐Znb`7╪G╢Ç┘â%a╝□K2w
ûd3ùcn÷Z=µû£~╠─MG│□
s+□ÿ7┐Σ□□"òÑ·□∞ZPü╪□08□╔`┴q8□╛ÄτT■□v□╧¿φp∞çgα7≡;x□▐ç□□0□σN┬ôn:|
τ▐□¿□/|
╛
≈└.°□╓v□▐└│7cfæ╙å'α

°□╠ç*z ┘□;αçp□Ä└oß≈≡'&1
σL□;εW«Ç┘P□
Φw□▄ÄgΣ▌p□NB□£┴
Æò≥╣└□ù@&ΣB□\ÅûµÅ;εÇ¥x■□□₧àùα□≡gfd6:}≤ⁿ2ûÿ

s≡╘╝□Ü`□|□■□zíÅ┘!□ñ9eò□╠«¼ÿ├G≡▄#╨▓qêùb¿σ├ì░□VC+▄
?üƒ├>°5<□»└[≡□|╧dæƒ NÉdPα*╚âoé□Vß┬╢□-Ç=≡┐α)8□/├¢≡!ⁿì9D~ g╝□□
WC□ⁿ;¼äf╕□╢┴â≡□ⁿ□Dí□║αm°
|╞Γå{0□

□□#ß2╚åB¿ª□╧╖┬≈ß°≡
x

┬≤≡*ⁿ□>å~µD□▓ΓEΦg7ò4┬8ÿ□
p┐╨.ç,hä-
c√åΓ≥≥"ÿWQ6_üeU□╫)Φm₧g$µJ└╥πßJ╕□µ┬b≡└Z°6léφ≡□<□┐äCp□^âS≡▀≡w□Oel0□fB□▄□╤α&
╪@ƒ╟±√n0íWGß╙ÿ□╫B1}å□Ç□╕
6├]≡3°Ox□°╒□αux□>ü□û(□ÿ1jRa□ε\s`-ⁿ□╘┬═░□╛□w├├░□₧Ç├≡"t├i°+

▓$v}=╘pìσ4a/aG+□Z□□▌5-□─¬□.&l%|ê≡EÄ6#íôÉ≥█╞□f□╬ £]π{m≤□╦
□□.%\FXO╪H□⌐⌐i□┌╓□╢□▐E╕¢≡a┬□ä╟□╗□O□~╠╤>Ä░èp□aÉp]¡▀╖▄┘àp;ß.┬ç□≈□- <D
xö≡8ß╓┌╞@â²5┬-┬Säg□?"∞#□αΦ0□┌δ|ì-G□a□a:ßX┬ëu!Oì#ô0¢p

a ßB┬j┬□ä□┬u╛ F_━▒Ö≡

┬φä;□w√┬□┐ πA┬=H▄π╪K° 6G9à░êp-!σæ)Åⁿ8ßSäç²ìM
≥1┬□ _&∞&|ô≡4ß√ä□~Ω□╘°πÇ╨L(□&□║ □┬±äÖä╙ g□╬n≡╓·Γ□
K□½□ù□Vc#í╕ :B?aê░Öp□ß□┬-ä[ ∩□áê█Ix?ßCäÅ□ε#▄O□%∞ ∞

äj□πN□v□╛ □Σ≈▀&|Å░ù≡□┬~ÄNë╨JΦ$L&L%T□╟□f□N
╒°#╬l┬
a;aö≡t□¥□ƒNÿAÿCXB╕ö╨O□□πXïo&l%▄Hx□ßv┬□ä≈ç}ìu±□□>B╕Åp?aö░â░ô≡D╕í&□▀E°&ß□┬O
8&□í¥09□╪║2a

ß8┬╔äYä3□g□□□û□.$╝æ░

qjB#aäp-a+ßF┬6┬φä;
▄C╕Å≡□Γ┤ä├ä╟□_$|Ö▒¢≡M┬≈□?"ⁿöcóDh'LB£₧ÿJ8Öp&ß<┬%ä+□âä½□[□o%▄Lx□ΓUë█
▄Gxÿ≡$Γî─╖□│□╧≡o□'~╨1  □═ä2a□í¢p

ß╕pS0£4Ö0ïp□a

ß□┬"┬R┬¬≡Ωp}╥□┬□äk ╥□εê`L&=L°(ßcä□□□□-%<NxÆ≡5┬-┬Säg□?"∞[¢5cz╥□ΓU.#Γ

ù□±jW□b╢+□±□W:b«k,Γ□╫D─<W&b╛k:bük&bík÷┌¼½│\àêW║J□°║*□°╣□#Nw▌êxò½□qåk□Γ╒«
b╢k□Γ5«□─\╫¡ês\¢□≤\w µ╗╣#□╕v
□║v»═╩╬r=êxÑk□ΓT╫^─i«╟□°╗₧B╝╨u□qåδ□Γ╒«□□│]/#^πΩF╠u╜ë8╟u□1╧⌡>b╛δc─□╫°êà╓░6
δÜ¼d3Γò╔2Γ╘Σ$─i╔n─Θ╔c□»J-ç8#y2Γ╒╔Yê┘╔3□»I╬A╠M₧â8'╣□1/╣□1?╣□▒ y
bar⌡┌¼▄¼Σ:─+ô²|°ì;╣Σ
É⌐╕çXè{│□q┐UM_£⌐í»»╘╤ùe|╕╟[ë{¬□·≥K□≈▓!▄m▄ìσÆb╨
ò□╧?ö≈_æ╧²╣|╧╟Vσj└^ªß₧ⁿ□v┼p/≈U╨é√╤"▄┘=├SC
ε≡y]Ω¥≤_¥¢▀ä√M□ε°,xó°*Z□₧)«└S└□·¡Iⁿ7:M╟□°

▄Ugπ╛u&ε7s≡,3
╤□│±ñ≡╒≤□y&F╥╘k□εk█±$⌠σ»□₧╣╬Γ%τ`▄□ß⌡_Ç□<#8@■□\1<ß}□û□πH<%¡╞ô╧□<[▄ä'é¢±\╢-
╧Y╖α╣µ█xé╕
úz□₧□╛â°╨É¥■_)┴≡ñ╙q╟τ`╥□ß□┬<┬|┬□Ü $±╗>%ì╞□'┼ □VP.■u¬!Mï÷
┬─
┬╤τ`Θ□b┬□α □δé□□└BXäτ°□≡ⁿ²?╫□|m□y<]J┌ÑτhC≈@s╧·ò╨□#p▌v_≡╒H║b░π╝(┴X╕∞□$â¥_Ç
□îâ╥/@21Ω╬Å□îç □ ═≡]hâ█a+▄█▀ :úO9┐

≥Æw└≈¿Σ÷□é.┴□a7≡╡w.}ò╕ ε╝Çêb0Ω+ß?╗□

╩╝□π/□k□k ╜äuä╦ δ }ä+□W□· □□□ □äA┬o□å□├ä□┬&┬Uä½¢
╫□«%╝ë░à≡f┬uäδ [
o!╝ò≡█ä□□o#▄H╕ëp3ßw□╖□~ù░ì≡v┬;□┐G╕ò≡N┬m1╫¬²□@x□ß▌äφäΩ┌¼╬

jd⌐c·>┬]ä?$▄═&IUR¡□æ6H█Ñ□ñ}╥aΘñ⌠╢⌠▒┴hH6î3L7Σ□¬

╡åêaâa╗ß□├>├a├I├█åÅìNcÄqàq¡qïqç±aπ~π1πk╞≈îƒÜ∞ªT╙d╙L╙<╙□╙f≤úµ¿·EsÅ╣╫□û□ïb╔
▓╠╣ö[¬–AK½uòuú⌡.[¡m¥φ

╗dO▓Å╡O╡╧▒W╪ù┘C÷╗∞çφo╪▀wX□n╟DG╢ú╚▒╪Q∩hvlv▄∩╪δ8ΣΦv£q⌠╦▓£.çΣn·ô╕ä8%nf▄╝╕╞╕m
q≈╟╝□≈▒╙Θ¼u>Φ|╠┘ß∞q÷╞'╟ù─╫┼»è▀□ pⁿ■ä╘äY ï□B
'□▐L°(▒(qqb}bsΓµ─÷─╜ëç□O$ìIZÿ┤!Θ┼ñ₧ñ^□╕□\è+╦u╚u"╣69Æ|G≥«□╓□cGL□1gD┼
ê-#

ìΦ-qfD□è£Æ₧ÆæÆôRÆ▓4┼ƒ╥Æ╥û▓3eO╧üö╫öεö3)²#σæΘ#3Fµî,□╣tñ□d╦╚╣æ;Gε□y`dτ╙εægF÷
╗ew║;├¥π.q/u√▌-
ε6≈N≈-≈□w°╗█}╠▌?J-ò>*cT╬¿ÆQKG·G╡îj□╡s╘₧Q□FuÄΩ-ufT□¬£Ü₧ÜæÜôZÆ║4╒ƒ┌Æ┌û║3uOΩ
ü╩╘ε╘3⌐²irZzZFZNZI┌╥4□ZKZ[┌╬╡=i□╥:╙║╚╬ñ⌡°╦ΘΘΘ□Θ9Θ%ΘK╙²Θ-ΘmΘ;╙≈ñ-
H∩L∩N?ô▌?Z-¥>:ct╬ΦÆ╤KG√G╖în□╝s⌠₧╤□FwÄε-}ft □yL·ÿî19cJ╞,Ñ▀╔╬≡

A╗│⌠□¥₧¬╙3t·Σ□▌ê·°□▌

0z1XXî~iûVk╓û√▓╢■ë3uzù╢■s⌠≈┤·'╔ @╧╤Θ█tz╧W╨æ□ûU¢~%h⌡∞áN□┼Φ╕g╓ÄΦ╥-
!]┬□mÆ┌├Öôà▄O)Fzª3□W]║{φ□Uµ∞Sσ╫□T·ì≥≤σ₧╝]╙c¬╟□!giYΣF┤╜╟}Y½□l╙Θ'╡σ
%¡>≈#¡^╘¡╙■╨Ω┼σ:╛B°WΘ⌠à:¥≤gg·□?á╙≈É«│N±úB~ñ╧yΓ■╝|╧=□╧δExª\î;b■²≡f_6π□╨Ä╗
□■╝Ωqê┬Qx□║á□N╙s?`Vû└▄L□⌡î□≥╕*»kQe╓T!▀Sσ|┐*K╟□9ñ□RΣ++□2(ΣQUû╧□▓]╚

!?Vσ⌡□□·ë*+¬ä▄½╝╚╩□B-ReU⌐*□HB>«╨à□U.□ⁿ□╜»⌡┬b¥╒□∩╓Θ{t·ï:²╙□□δ_▓M¢╛Σ¿N∩╥Ω>]√
+□╡ ·╨¿N?□ú# ò∩ôεå□⌠╜⌡R<¥▄ê√¥F▄ô¼├}├□╕╢∩ó°┌Å┴S╨□╟ßex□N┴√*█ò┬[■┘B╛┌╨åì¬l╞□▓
V╚²¬

êq□^□┤        )╞l╨.dëÉ┬
┴¥Bèv╛ 5QH□□▀□^
      /åD·Éh'$°å║╡V□Å╙Z)<ç⌠d╙ⁿÆvQk□╝
∩Qe─(Σ□!╗U┘┤TH1ƒ¡Ü.ñ░┬jYHa╡ ╒o½▓9GH╤ï5╒Bè□o═□U«□╝]{Xò7⌐c;f╛╜i¢É□┌~╖TæεÇt<G
e└t╙ü|(ü*<'╫é¿╡Eîñ¢g¬r¥□·δ║T╣~½*[┼╚╜E╟┐╖□▀▄*|⌡mß╖o≤╒╞v6"7l╒ΘwΘ⌠v¥╛_█□ìÄ½╧
m'┤·oS█3╨≈

$\pi^U$

╖ëx┌ÿ d□σ╤═¥□à□6□√o□■▌T¬╗∩&aâMOiπlS?╒□ôss╛É!φ╝▓yçN╫YΘ;èN»╒┐½│╥φ·:}vîÄV╗π

X$M╛ù%ΣüÿÿÆ£\∩╘Θ°⌡%╖□╗l▌ímπ╬╔┌Æw╢Φ⌠7╡╓┌6Äj┤║□£□ïj¬ÿs╢-
□R─╒≈δU╣}ÖÉkà□1□W6╡bçT□
ôa*╞ä90□*`
ê□wï±▄.÷□≈□>≈▄»╨□'Uy»ÿ¡vèæpƒ□ƒ≈ë|≈╜°≈÷.1/φZxN╨f!▀╓┌Σç}zδε□²■╤□┌£?^u
n\?É-Σ)U■d╫╣y-□π⌠Aa┐□≈Pï├⌡>╪y╛▒≡SßàçûΩ≥gbN{╕▀▄□■πF!-
□≥□╗∞I□▓Ωï<╛GxpÅXπ□.∞ⁿê╪□ⁿBî║_êy≡Q▒╓∩m□ïü_%┼°£R÷Ñ□)Fß>1C ùΦ═c
U·╥[╡╢■σ^¥~\▀ú╟┼╛µ±π┌£O,=╫>O□∩∞»Sσ»R(BM⌠═!■
□─Hⁿò X=□%·w@∞à

ê╜╥ôv²<⌠ñΦ▀ôK┤ú Iuùía±ñÿ▌₧□≤°SÖ┌y⌡⌐vφh■╪¥|e□µÿ=╝wñ≥┐₧ú╨¿X╥ú»⌐≥α

U-□╢}Z∞Σ

;à<í╩g╢Ç┼¿π╪!╬    □/½≥╚¡z╖-
□'ΣΓsR─¿~╓I)ƒ╤√┘YBèyπY┴√ÿαpLΣ;&V▌ c°U·£h·╣j¡╟ƒ█ó╙─
%╖~Nî?≈ÿ*;┼*√╜Xæ₧□cτ╕ÿô^□{ä□D

ⁿ╞¿mσD╣N_Ñ╙╖δ⌠>¡w□;[□F{Y∞ÿ_╞5╟□3?╝≥░v╓~ÑïΓ┬B^;╗ïP╙─°Ω□6|Uî√Wà?_□u╝÷Ç*_▀K#
≈£æ╨-Fv╖ÿ   ~╖Kòoê·ε≈[╧7g⌡ê□╚¢□½≥Ån!?·|╞°─LpJx·□1JNïh8-
ⁿ≥«ÿ√▀=□cG▄}╝Wñ╒?<¬╒ Æ□□║□·°Åj╧■²nφ╚φW≈≤╬≤¼]+
□kßV╪"J□█ⁿ]⌠■∩┬6□□Hò□b_6╨(ñ╪-

è▌Φá┌S□▓É□!╒╜?cσB₧╘0dR÷ùd╙$┐É╕äT╧ª╟á□┘,ñ:?2cÄÉΩ■ÜÖîBn□R¥□Ö╣NHul0K
X╟g#öY-╒═ô╟"rZ╟kF□│µ â╜□│û□╣]HuÄf╢╔Bè|÷□!╒ô□sî□R╪B₧Ñτ$
k╙ç┤÷ò1é,1╜æOΘ╥□H╫Å□&½±└Γ2à\!ΣCB₧>□9g¬ÉUBn□R=ß│x°fª┼□N|í*□□jV□û╨«┘╙▓ä}1ƒ¢
ß;i¬Nƒ¡=ƒ4K°╧ⁿ□z╢Nƒ□▒ƒK▒□S5ga6búN┐U°»╙÷g─Z¥╛Us≥`#┌╡zè]°kO:,M╤Θ¡┌ⁿi=Z}4hµ□
6Fë┘╣!ƒ1┘║⌠R¥╛/&?╛/æ┤φ_□╗ª`}ùÇ╓─ùXuσì:]ù□L┐«ⁿÇVWJ┤φ+Ñ:}₧N▀□╙─÷G┘ªK┐K°∩╓÷
╥╜:²┤V┐∞.m²ù□╡⌡MÿÑ╙Åj⌡ë□:}▌╣z¼╜&~ñ╒'=J½▀░>╣\[~≥□¥^½╒3┌┤·□A¡₧YÑì°)┘:╜P°▀¿╙
ùΘ⌠v¡?°<¿K┐_k═)]Z}ΩN¡~U╡V╧─°╒g₧ª□Γ░■⌡=Z}vP½τM╫·╗áO½□┌╡·▄¿V/□Θ⌠N¡^<U½╧╙┼Së
¼╙τΦ⌠□Z}╛.>KÅΩ⌠□¡^&Θ⌠□¡²╤Æ╡·⌡]Z╜j°╢ⁿój¥«│╫Γ*¥■)─~b╬nH╥ΩO╜□│s─□⌐≈kΓEJφ╥─┐ö
zR°ƒ╨î□╥cΓYJô5±!ÑM╓Ωú▀╫Ω╧^¡~┘*¡>~ªVƒ8á╒┐vH╙?)sú╞₧R█□¡~{┐V┐█¬╒wk²#²L╤Ω{3┤·
»┤πU:£»╒ƒ╤ÄoΘx╡V□┼»╒{Ü╡·╗□┤·G[c÷Qxτ│¥Ü⌡O·lç╓─
ƒ=¿K┐_°?ó╙╡ƒUK²╓:]╫ƒ■çbvBx°□ƒ╢²■╟t·^α?-
dä□÷□¿c;⌐╓el╗)┴öê)I`ûï¥□ræ≤░3Ωñτ□∞╧¼ù}─▄█Öδ╟#}□»GJÆ°╧5Ö⌐VfêCL┴6°■É ¬¼íOè▒
]╓"9ñ□iä\(╧Ñ:
ÿ^□ⁿlz÷≤Σ□▄□□çƒ┤□ç?[6└□≡0≡┐jⁿ□∞â²(ç°α0╨Cp□Ä╤╫â$□╓□ê┼r%Γ<τi0H
╙■]Æ□╬≈PÄ@²O$;£/âäZ□bç≤5─ú╬-0 »├╬?É∞p╛ë≥□Ωoæ∞p╛─╥ (╥Oë⌠w╘t·:ΓPB□µs

r)▌)ú;σ─Ω□╡┌I¡-°V_ñ;'Φ╬I~ç □e╛{4îÑ±□≡#⌠□╖╨¡¿╞│xX╧Rq╖╙╨&▓ë░ü-aKß6µg
░Ö□X□╢║░o▒□|ùma[α{¼¥▌□[┘ç∞C╪╞>aƒ└≈┘g∞3╪╫┐░□?É╟Æ□εÆdIå╖ÑD)□┌Ñ□)□εæ╥ñ4╪!]&]□
≈JôñI░S╨Æ╨ß>)"5┴S╥ji5DÑ╡╥Z8(▌,¡âC╥□i□□û6I¢α□i╗┤□:ñ╗Ñ╗ßê⌠Ç⌠□□5─□£╨oÿnÿ

□å9å|□4□□èÖd╕╧p3□#╞1ú⌐╞T├ªÜ╝&/¢fZnZ╬ª¢|&╖╨□6à┘

Sô⌐ë]mZmZ═▓M┐5of╫╪+φ-÷ü}ôâ▒□9A.É╔╙ &∩Æ~□W□╖B·K▄·╕6ΘS°Σ┤□¼╬K¥ù□Γ¥ù9/3$8/w^
nHtNpN0$9'9'□\╬»9┐fHv^ß╝┬0┬9┼9┼≡┐┘;□≡,è¡ÅO█¥}w≈□□ä□"""bh□□╞╨□□ñ□ñâíJ□□ñç"
]□DÜ┤ï□B□=⌠╨□ê╥D@ñ≈▐╗⌠□╛│'K▒▌╧δ⌡▐τzƒ√╝□τƒⁿ╪▌w╬Ö┘93│%□*ƒ╩╟╙½□¬□□Ta*îgPß*£□
⌐B¬□□Y□QExF□í"°+¬╕*╬3⌐H□╔_UÑT)₧YòUe·k¬╛¬╧│¿&¬
□]5S═xV╒\5τo¿╔¬5╧ª┌¿6ⁿM╒N╡π┴¬ôΩ─│½╬¬3╧í║¬«<°ΩÑz±\¬ÅΩ├s½O╒°<D
T□y-5H
Γy╒□5äτS├╘0-¬F¿□<┐□ÑF±□jî□├▀Rc╒X-ªbU,□[ìWπy╕èSq╝áÜñ&±BjèÜ┬
½ij□/óΓU</¬f¬Ö<B═V│y15W═σ┼UéJα%╘B╡ÉG¬┼j1/⌐û¬Ñ╝öZªû±w╘□╡éùV½╘*^F%⌐$^V¡Wδy9
╡Amασ╒7Ω□^AmR¢xö┌ó╢≡èΩ[⌡-»ñ╛S▀±w╒

╡âWV▀½∩y□⌡â·üWU{╘─^MφS√xuu@□α5╘!uê╫T╟╘1^K]QWxmu]]τ∩⌐¢Ω&ÅV╖╘─
^G▌Qw╣╖□±>⌠E╖Θ□·äi)╝□
bYX0/°5╥Üh═┤µZ{¡ú□ú2½,*½╩ªéU

òKà¿╝*┐zK╝¡□¬┬┬¿*µ÷e¬î¬°□½ªΩ□╒J}ñ┌─Ä*FuQ=Uo╒O
Pƒ⌐┴j¿·BìTú╒▀╘ùj£Üá&¬╔j¬Ü«f¿YjÄÜ°□¿EjëJT╦╒J╡╞φ█αî▐□τ÷V╡MmW;╒.╡[φU√╒AuT]V╫
╘
⌡ú║φ▌1δ╬□3πyε«αj┤-m*ûΘÅìê :/â╨╦⌐n╓á-
⌠C %■╢≈√jα[u╚U¢┌█▒R_□ƒ{ ZFÜàµáí┤□ìñσiUΦ⌐□╤û┤=φF√B∩<é╞╥)t6]DW╥»ΦV║ï-ñ'Θyzò
▐é=╫╤═t'▌OÅ□╣N∩┴a
êO ╦╟éí/

g┼YYVÖ╒f
Ys╓ûua╝Y□6ÿì □Y}╓î}─bXO
C┘□□╟ΓY□KdIl#█╬÷▓ú∞,╗╨ε░d«s┼□x&₧òταya╧G\p¢°σ□üΣé■║□/┼úxu^ù7ß¡xG▐¥≈πâ·(>₧O
πs·□╛Ü□┼7≤φ$#ÅσS°l╛ê»□▓ò∩Γ□·I~æ▀Σ□□□ªH#éD□æCäèB"Rö□UE┤h(ÜëV╛.äC¼:·║óv≥uCì
±}î┌╫□┤#ⁿ╘□╡ú»□j'_o╘□_╘╬╛O@;┴v²P;·>EφΣδÅ□π□Ç┌≈□h

l7□╡ú∩s╘N╛┴¿1╛!¿¥}├@;├v├Q; ·╛@φΣ□ü□π□ë┌┘╫¥0°▀╛`;·□éì±
□█┘7□╦▀┴7╞≤∩o₧□c= ╛⌠ⁿï⌡ⁿ□τ·5▐≤kéτ╫D╧»I₧_ô=┐ªx~M⌡ⁿÜε·□∩·5├≤kªτ╫,╧»9₧_s=┐µy
~%x~═≈ⁿ□□^t≡┼í_╙╨»┘Φ╫B╧»E₧_ï=┐ûx~-
⌡ⁿZµ·╡▄½╖□₧□+= Vy■¡÷ⁿ[π·ùΣ·╡╓≤k╝τ╫W₧_□<┐╛÷ⁿ·╞≤kôτ╫f╧»-
₧_[=┐╛⌡ⁿZÇ~%b¡¡C┐6ó_▀y~m≈ⁿ┌ß·╡╙≤δ{╧»-
<┐v{~φ±ⁿ┌δ·╡╧≤δÇτ╫A╧»C^╜□÷ⁿ;Γ·w╘≤∩ÿτ▀q╧┐□₧_°<┐N{~¥±ⁿ:δ·u╬≤k□·╡
²┌Å╡v□²║α·u╤≤δÆτ╫e╧»+₧_╫<┐«{~▌≡ⁿ║Θ·⌡úτ╫m╧»;₧_w=┐εy~▌≈ⁿzΦ·⌡╙≤δ▒τW▓WoORⁿs▀█
Ω·g╥□ LûΓƒ╔= ╬ú_W╤»[Φ╫□╖╓áÅ¡M▓╙]l"⌠O∩≥fⁿ□▐Æ□╙;≡N╝3∩╧{≡üⁿ3>ê□

╜╒□□e₧Σ°°i~åƒστ°y~□zªKⁿ2┐┬»≥kⁿ:┐□²╘Åⁿ□┐φw»Θd°;Y□»└+Aƒ╫ö7τ-
x{Φ·bx□Φ²z≥^╝7∩├√≥O°8ΦδVA□╕å'±╡|□_□=▀□■5 åoΣ¢áW▄□²α╖|□ ╬ □╬ÄB▌╝□▀z¢□□▀□Æ°
╧╫□2
gN¡▄-D&±ª╖à¢1â╜-ª├ qΦâ[□"╥èW`╖`□L¼□÷p┐#°à∩p∩²O□=∩KB□║É┬□>Φë-a
┐P"òpä;╥□«O≡uε>L□□□─□%D ÿ□2□N2≡x>□z··┐µ╫»E0├*╪O≥Θ|:□s□ƒ□ÑÖ├τ□╞□8⌠0□▒
≡ì'·%ó2¼╠░□b└H□ äφg└v ┐□╤_⌐;╪KçÆ┼├w╟Σ3ß;µ≥╣≡□≤9⌠^P╥∩α;\□≡□Ωô²□ôô□ôτ1Z
[Aî┤Å╪Æ_<Kò≥T┬╙;δGÉæd□□
|╩┐ßSu▒d□□O&└£╘}f`2î┘ªB²Mç□Ω

2ô╠é9Ω□2ù╠âYΩ|ÿ¡.$ï╙bÿ».%ëd□╠XW└╠u□YM╔└▄u-
YG╔├∞u□╠b┐!□╔&ÿ╠n![╔╗d□·Äl';╙N≥=┘E~ ╗╔-▓ù∞#√╔□rÉ□"ç╔□rö□#╠╔
rÆ£"°╔□rû£#τ╔□ræ\"ù╔□≈ì*Σ:╣AnÆ╔-r¢▄!w╔=rƒ<
□╔#≥ÿ$ô'╨Φ(½┬¬▓j¼:½┴j▓Z0₧xÅE│:¼.½□πê□0║xƒ5bìY□╔□F□-
└Xú□k╔>d¡Xk□c┤üæG;6ëφg□╪Avê□fG`tqî□g'╪Ivè¥fg`¼qÄ¥g□╪Evë]µ&╗┬«rï]c╫┘
vô²╙n▒█0□╣╥ε▒√∞□{╙-▒╟,Ö=ü

Çr╞9îH4□°Hnp»┬½≥j8─⌐╟□≥≈yk▌

F$ƒ≥■|□╔┐äq╔□╛É/åæ╔r╛éoτ;°N■=îF~α╖·─╛ù∩π√·□□¢□Γç·□~öπ╟·
QX□□╖─□b╖╪#÷è}b┐8

èCΓ░8"ÄècΓ┐8!NèSΓ┤8#╬èsΓ╝┐ .èKΓ▓┐"«èkΓ║┐!nè┼-
q[▄□w┼=q_<□□┼#±X$ï'Ü_K#K╚HYRûÆ∩╚╥▓î,+╤╔≥▓éîÆ□e%·«¼,½╚¬▓Ü¼.k╚Ü▓û¼-
▀ô╤▓Ä¼+δ╔·▓ül(▀ùìdc°4à╧□≡i![╨□e+┘Z~$█╙╚╢▓¥l/;╚Ä▓ôîæ¥e□┘UvâOw┘C÷ö╜do┘G÷òƒ╚~
≥S┘_

É□σgrÉ$^{n}$\

ûCΣP9L

ù_╚□rñ□%G╦1≥or¼ⁿR╞╚qr╝£ πΣD9INûSΣT9M╬æsσ<Ö
τ╦□rí\$□╦%r⌐Lö╦Σr╣B«ö½Σj╣F&╔╡r¥\/┐Æ□Σ╫≥□╣QnÆ¢σ□╣U~+╖╓∩Σv╣Cεö▀╦]≥□╣[εæ{σ>╣
_-É□σ!yX-æGσ1y\₧É'σ)yZ₧ægσ9y^^É□σ%yY^æWσ5y] ▌É7σ=y_>É□σ#·X&╦'□1¿£.πσ

9S╫Æ│σÅ≥û╝-∩╚╕f□│½┘=ⁿ╪∞n÷0{Ü╜╟▌f-
│»·ë┘╧ⁿ╘∞o}lu╖zX=¡^Vo½Å╔╫ ·─Ωg⌡╖□X□¡╧¼A╓τ╓`kê5╘□f
╖b¡q╓xké□gM┤&Yô¡)╓TkÜ5▌è╖fX3¡Y╓lkÄ5╧J░µ[
¼à╓"k▒┤─Zj¡╡╓Yδ¡»¼
╓╫╓7╓Fk½⌡¡⌡¥╡▌┌aφ┤╛╖vY?X╖¡=╓^k┐u┬:e¥▒╬Y□¼K╓5δå⌡úu╦║m▌▒εZ≈¼√╓□δí⌡╙J╢₧╪─ª6│
╣-l═╓φS÷i√î}╓>gƒ╖/╪□φK÷e√è}╒╛f_╖o╪7φφ[÷m√Ä}╫╛g▀╖-
╪□φG÷c;┘~Γ'~Ωg~ε□~═»√Ñ▀≡√ⁿª▀≥█~┐_·S·□□j□□ K■┤■t■□□z□á?â?╙ ▓?ú □□& ½■╟■╫ⁿY
ⁿ»√│·▀≡g≤Å≤Å≈O≡╟·'·'·'√°°°.°.°√π²3ⁿ3í▀}-e

╫Æz▒ë

zP\!Ü╟╤≤□d□»╙+æ}<Ü╪!□x} ▌Ç□┬îwä╖σm╓Q╙T}╙1>éÅ
°°X>û£å□<Ä£┴ls□│═9╠6τ1█\αë|□╣ê= eQP□ó□WÜÿfj&═½9ÜC≤ßZR¿~B?K╧╤╝▓□╜èδJ?Ü□╠qî
Ö╙═╡,╜╣┼╜╟Bqu⌐□«+┼c÷÷æ□╙Θañ8)ào□w»τSΓ├╣YaVÜòc∩Ü#═eµuB±εë╥α{Y|U┐{ÅXMb└□ª>i
□{╡⌡V═▌'ƒ8ΘBzB>Γ≡√@╙A□r╤□2╓·ö0╡┼Üiv□╪}╓f░□¼-
`□Y█É∩□╖▐:Ç÷□╪o¡#`┐│Äü▌a]q╖QΘ▄}UÇ╖»JÅ√>Fε□√ì2┴nR□ÆTH□$⌐æ□"╓Ç$╙%É?g▓∩ E·≤°
┘≤┐;w■{▓°¢-
□oNⁿWµ├4▓ël&¢╦Å!╗╕Y±□╙çQÿ⌐¬@╓□è9░6Σ?7≤ÑΣ╜ª┐3πu □r▌/3▌ùÉπ₧g╖□3╟□Zª{û╓
τìà▄ⁿb╞+□#
w\æ2¬p╟□òaTq▀□S<ä□┼{0Üê├±─D□M<ÇV[□Zj□╖]>═ï¼⌡Osóφ╪⌐φ4÷KvZ;¥□`°╖□φ

vÉ²▓¥╤~┼╫d┐jg╢_││╪»█Yφ7∞l÷¢v░¥▌╬±½Ö⌠╙_╧Ñ╨°Le²«î:≈ù9UÑRÄJ²ï╠║╓┌lm┴ⁿ║φW3∞>╙
▒□¼C╓□δ╪╘\½□Tz╠╖W~3π>■e╬Uü*â□·CÖ≈'y╫~ⁿo╚╝ò(úΘ`□□DâIZZÖV'»πuú`Zƒ6%9Θ□⌠□Æƒ╢
á-H□·!mM▐ómh7□N╗╙╤ñ□iÑ□H}║öε ìX{╓æ⌠`1¼□Θ═z▒>d √ä
ƒ│╧╪□2£
c#╚h╝□⌠%□├á╖gq,Ä─q¢°!□yZ₧û─≤□₧â╠α╣x-▓Üτ πÑ╚:╠µ╗1¢∩┴Ö┘^1E∞ □╡╘Zj□¿▌╤ε╨

┌=φ-
╥-h□Φ╥:äïf╘?╙ç╨W⌠a·HÜE¡ÅÑoΩ▒·□ÜSƒ¿╧ªy⌠╣·□ZXO╘7╥R·f}'¡í∩╒≈╥··□²□má╤Å╤FÉ≈-
╙ª·□╙√}eÿ,Lù╥ó▓□M2▓□9Φz#ùæçn0≥□·Φ&#╞□ú¢ìéFA║┼ê0"ΦVú╕Q£~kD□æt¢Q┌(M┐3╧□σΦv#
╨êó;îΩFu║╙¿e╘ó▀□╤F4▌e40□╙î□F
║▀□Sqz└ld6ª□═ªfsz╪livñ╟═□3å^éQ├8z□F

kΘm≤‖yÅ&[╠¬├ñU╧Ω╞▌╗'┌'Y/ □□,█ÇwÖ1ÿi&αèz=┌╠#ë/□w□A'Y`▐δ▐[δ▌╔Is╥□□N╤╩□t,l?□
╫□ª├^□□N└▀╓x┐¡ü▀Ä└çAÄ}_
╥^╓2j»hÖ┤W╡╠┌kZ□φu-½÷åûM{S
╓▓k9┤£Z.-╖□óσ╤≥j·┤P-
?²üεª{Φ^‖Åε°□ΦAzê-ªGΦQzî-°'ΦIzè₧ªgΦYzÄ₧°□ΦEzë^□\□~ç▀σ≈°}■Ç?ΣÅ°c₧╠ƒⁿ3L□Aà√
╠┴√0□K
q
$□±]□╢H-R□b□ü□|¿□⌐à∩úpGg═±}□ε□Xz┴□öè:p□eó┴p□ECt[╨Vp₧tó1┤□²î~N□╙ßp«,
┼┐≥▒ænó█p┼Φw«□²▐U*wòFöx╢B4▄[!rGÑEY>□╞J▒≥0.⌐□úÄ□═üµgµ s░9─□j

3ç¢±µ

sû9█£c╬5≈Ü√╠□µA≤Éy╪<f-7Oÿ'=S^∩²╝∩v√µ°=│█ô>φG▌■≤·Lσ□ ▌
Pÿ╒}V«$»\3└V╟r□äÆU╞ÆEC┘┌²▌▓%ÿ≤═□µ"s▒╡─\j&■ñ¼τ╠≤µ□≤Æy┘╝b^5»²▌□°dÆ□≤╚
ï>ñ⌠≤/⌠≥ΦE9╔╦╗fU╚≤à□╖□╒al▀Ä□wå@"▌Y$⌐Γ^□&╒¼ÅφW╚v╚f»╨/a╗□≡6-
╠r┬X□íαuA8f□Väp≡┐□□P;σë□æ¿

G¼╩¬□ □⌐E

êJ4╟-
Ω▒z─ä°4%□î□?$6î□█□┐·₧·-Q0sφIR┴∞d□q╟Qµ8Æ□µ(ë$½╣▄\K┬`ªrìDÿ7╟{ñ)Σß□ñ5d▄ßñ□d╪
╣ñ/Σ╧Ñd4Σ╦□d□╘▌□▓□j≡□Y□Y≡□Y  uyè¼é·<CVC¥₧#ká^/É$¿█Kd-
d╞+d□d╟kd=d╚□Σ+╚ÆÅ╔□╚ë:┘

πä@▓□╞□»æú0-╙A╬B,,r□2tjr□≥d□îóƒ╢ïB^╖°_D ·ê║æîä°ñ⌡«¼╬─{p° "√OG÷iL#ƒ╡╓ E÷╧k
│┴╧·┌Yεu
\□JëΦk□╤╝.5╟¢╙Ç«37□ƒy■r≈°·Uæ□tow»╜/£/│₧╒Ω?rö°√τ}Vè□ⁿ(

·┴á?2.r∩ó°##□Γ>╣┬I□□K▄cp╪36□!┼╔ArÆ\─wÄ3j╥4p╘▀□╧┬f║□<8□Ñ=K/╙¢□╞¬L0ô9,Çet∩π
`!¼□+─è│╥,□ZymVƒ5a-Y[ÿ?tg}┘@6ödgi≡₧Å□,□╢î─▒K4k□[╡g▌`¢Al□ïeS╪l╢ê-
gI∞k╢òφd{┘avÆ¥gW┘-÷Ç□«╗3

-ÿr/□£oçi

⌠1□═ì□M├Qδ╤é¿⌡i!╘□┤0jCZ□⌡}Z□╡□ì@mLïí6í┼Q¢╥□¿═h)╘□┤4j+Z□╡
}7%╢ZzWEó□ê║L╦α¬zhX«j/□╢½·4├Å║╞P¿IF*╘╟åâÜlñF}bñq□µ-/í□KòRç-
ivΓ╢å£`âi.░╤4□G■y└╓°╨■└╟|`□╥P░∩╙ⁿ`□╤□`□╙╖└6ía`¢╥╥┴6úæε¥9┤$╪□Θ;`[╤2`▐╨▒`▐╥
r`▐╤≥`▐╙(░▒┤"╪±┤□╪qZZ┬└▀t`ùiε□πCâ□□₧┬Ö□~□░k

lÆíâ}lH░╓å□÷ëß#

|â╣ïQî╬□╧b┴¢9¿⌡Φ\╘·t-j□ÜÇ┌É╬GmD□á6ª
Q■°ïP¢╨┼¿MΘ□╘f╞+«By2╣□%z□uìæ□5╔x
⌡▒æ□5┘x□⌡ëæ╒U(▌□¿┼h□╞z"Fy□Fy2Fy□Fy*╞w□╞w:F<-ú<□ú<□ú<╤ì¥æ□úô

ú□Ç╤IÅ╤      ─Φd└Φ□at^╞Φdtúôè`oπ▐╖δ>àòì╕OV       Φ
╥Aiå├□ε÷Mε▌dÖ╝╗╧▓╨□l□Θ▒6□▒╓2ámÄu=╞²ÖP£7ª┴'KYP¥áª°┤α╙ce

j□┤^P{░QA□└V
Ω□╢zPGù├□□╓g┤□¡E▀ú╡iM┌▄¼
Ö*:σJ
δ─z┬Ö>Ü╟≥Y|æzñ-½d⌡□·─  f£9╤£dN6°ÿS=i╨?«7┐27ÿ_¢▀ÿ□=MµfuW1┼òPÜ╥òTåy▀|`>4-
ÖÅ=d≤ëE,j}aì░FZú¼╤╔□δo╓XδK+╤Zf-╖VX+¡U╓jkìòd□┤

[G¡π╓Iδ┤u╓:o]┤.[W¡δ╓M[┌åφ│M█▓m█o+;ò¥╙╬eτ╢C∞<v^;ƒ□jτ╖
╪o┘a÷█v╕]╨.d□╢ï╪Eφ□╖ÿ]▄.aG┌%φR÷;╩V~ÑT□⌡ÆJ½ε⌐√ΩüzYeT»@D│ßZ
┴⌡□
f□□ █╥d¡ ├wd□!½≈`=ê∩¢U╕jÆ□╫B□╝ÜæÜ/Σ
I□}╛╛Ç╝ñ/╙ùæt·]².pW□HzwàÇd6ÅÜgHvw¥ÇΣ│□B₧/d═â

_╥:□□>╨:d□"□1╧W┬< .µ·╨ÿτ½`₧»èy╛□µ·Ωÿτk`₧»ëy╛□µ·┌V2d°≈l□▓z#╟Ω=0½≈VΘ
½□□~«$╤┐°F X
■KΩΘi
Ö□Mé╤⌠a□╙`□_╞8fE╧súτaΦy□⌠╝:ÄgjÑ¼╖hªµ╟{≡╦ô-`#Iª□█ ╧[±o╖╟ö╢π₧╖╨R╧│□)W
áåƒ═╬═╙µÖƒ»^>_╣ä┌□p»Ω`Ö,M=+╙╙╗≤□┤║-╨┌Fy█╪Z¬□╩□H|╧╧ß°τ⌐{M
┌p□□ Tú╒▌╢□-±.l¥ùñ√╜W□□wÑèÑ▄sß»α»@╥zO!╕%ñNC╖d╬z┬£╢┐╩╣┐□ⁿO╩Φ¡╘|æú√╘rz'½ô▌
q▐r┬£╖¥p°áS╙)ΩötJ9∩8Ñ¥2NY°£S▐⌐αTr¬9u£□x╘.▐æ┬╨ç┤╧Äe Ω▒`?▄+Ω'√få}â^╪ù
:┼□╫V╣□╢□#▌pÑn&>3ßn7□ÿú·4!NC7□N#°)Dµ□°%╤¥VNkb:m£vP┌

N□Æ╨ΘΣt%Ä≤▒≤1Iτ⌠pzæ□°Å╙ùdp·9²╚╤N□g ╓Φ

q╞ÉW¥Xg□╔ε━C<
╕ ≈ΦÇ╖Q≡□ò£J@½: ╒H

°åSâΣró¥z$╖╙□óò╧□∞

&íX┌\h▌Zαñ;|▄°qg{w1²UJφ╛»δ»Rj□█╒xVzAz┬g□qƒªvƒ÷

·ïò▀} ª√₧NΩ¥
n╔S{%w╤L▒╟□û┘ çe╢░╟□╤£□╤£□╤£

╥□êe

┬2┐∞|ε|N2=+·T,·□Æ▀Ö□σ/°B;ê┬3«7q▀3ò≡ô3ε▀Uû?┌éú0åσ▒ cñ≥
% ú╟KΘ_9÷·εoÑë√nε└?φ╕)πOÿ√└°4/╔Ω□≈τ[V}╔╧C╘▒4A°µÅ▓≡s4iH¬ )σ··╖</[V(]ª▀(█»ù
╨╜{ú╥ƒ□Ñτσ·O8;*■╔m¼Γ
m!δ
mß╖Γ]±□Q·√-
¿Γo┤á┐Γ·]±O?□ƒGƒßô)╧╧⌐?°⌡■╝╢■∞3≈╖k╫}cH╖□≤xΘy╣júφï#èüñ/□J▄wQ {G□╧KS⌡□eJ╣k∞
?½Lε╚╦}Å╙_cΣ⌡b┘ƒÄ╖▄qπ_c▄⌡≤╥/ç╥'²àJ ≤╔ú┴□▀□╞╝K╙:»W╧°□⌠Bx¡Φ»_□εô≈□▌Üp╫A≡φ□Ä
√L╬≤ò□≈═[┬□Bä┐□ö╪□□Ñ\+ ┬1□OYO
)∩■r≈p£w┴├jNe┤U\√w≈q▀vò□÷qW2▄╝R┤Jè╛X□êb~╥£|D:BÄss□▌²KΓ╝w$Æ╒)o;
╗╔Ar££%ù╔MrÅ$SAMΩ╜□Ω>q□LCh□Zê□°Ñi□¡Jk╙·┤ mI█╥□┌¥÷Ñ□ΘP:╧╔□Ñ╬Ω╝□÷
'□╪l╬¢`▀té┴□;┘┴fwrÇ═ßΣ□¢□Γ╔£\Nn░╣¥□░!N-░y£╝`≤:·└µsB┴å:·┴µwá╬£□□gµ«¿Ç
s▌□√╢□

6▄ü▒Æ╗┬□╢ÉS□la°□╪"NQ░E¥□░□N1░┼£Γ`ï;%└ûp"┴F:%┴ût`öΩ╞□¼□gµ«≈Ç-
πö□[╓)□╢□╘$s╫□└VÇ╢├£(h;

ZPU£ƒV┣┏½□┫║å□
6┏┌─□┫║ÄS□l]hS╟┌─□mèA╦□

v≡
╧1°d1≈LN╣ε≈Å╡═τφ╬}ÄMç3ú>a┌°«⌡C&Ñ6·□jh

ìú±4ü&╖$║æn°{ΘQzû^Ñwh2╙Öb□,□╦╞BX□ï`ÑY%V≤□<≈¢ìç≡0-┴K≤J╝&»╧¢±Åx

∩╔√≤í│

Åπ±<ü'≥$╛æoτ{·Q~û σwx▓╨à□□"ô╚&BDÿê□ÑE%QS╘□═─G"F⌠□²┼P1F─ëxæ
□EÆ╪(╢ï╜Γ¿8+«è;"Y╙5Ñ□hÖ┤lZê□ªEhÑ╡JZM¡╛╓L√Hï╤zj²╡í┌□-

óô╬δY■°Rîo

îoNîonîo□─╖!
┼~°□÷;ß╪∩□┴~'□√¥□╪∩Db┐S□√¥▓╪∩T└~°"÷;òáå╞É*╪∩╘┼~□╞£"□ⁿ╠□ÜE□ï□╨\"Täâå
ë""□┤8x□≤sQ^T□5A½╗╧6â╓□MDK╨µ□ôÄáφE□╤□┤╗Φ+□é÷□â┼□╨ß□⌐±á▒bÆê□¥&fï□á    bëX
║□Γ≈5ΦWb│╪

║M∞□√A≈è├Γ$ΦqêΩe╨ïΓ║╕□zK<╨□h▓&4ôp═ÇX°□Mú□jÖ@3jY┤`╨lZ.-
□4/╘@□╨BZq¡4h)¡╝□=□╘Hu-□┤6╘K□╨FZsφ#╨VZ{¡
hî╓]â╝á⌡å┌ésC□ñ
╫╞ÇÄ╥b╡Iáq┌4m6ΦL-A[□║H[«%ü«╓╛╥6ân╘╣i╗@wj{╡├á□╡π┌Y╨╙┌Eφ:ΦUφû÷□⌠₧û¼
┬uª□║□╡⌡4:╠□⌡□=ú₧□4│₧M╧□ÜC╧½çü□╨
Θ┼A#⌠Rzy╨▓z%╜:hU╜╢^┤«▌Ho

┌LoÑ╖□m½╟Φ▌A╖Θ╜⌡■á²⌠A·p╨í·(=□t¼−°O□¥ó╧╘□@τΩï⌠σáë·j²+╨u·F}□ΦV}°╛□t╖~P?

zT?¡_□=»_┌oü▐╘∩Θ╔áÅ$ô□ßRù╣L□Ω╚□Ö□4Hfû┘@│╨□2/hê,
üå╤□Y□4Rûòò@údUY□┤ª¼+□ü6ö═d+╨û▓¡î□φ(╗╔▐á=e?9□tá□*GüÄÉce□Φx9E╬□iùsσ"╨□2Q«□
])╫╓ìá_╥¡r'Φv╡[-□▌/Å╧╙á'σyy□⌠▓√╘□Φ□·╚`ä□─╨▌k┘åi8F□hZ#╚╝ójd2▓□9@âì□ú□h¿□nD
Ç□1"
ÿ%□Ñì(ú*heúªQ□4┌hh4□mb┤4┌é~dt4║üv1z□²@√□□ìíáâì□╞X╨1╞xc□Φ$#▐ÿ
:█X`$é.1V□δ@ôî»ì¡á¢ìφ╞n╨]╞~π(Φaπñq-⌠¼q┘╕
z▌╕c<□}α#>¥pƒ≡Ö>□T·╥·é@□}Ö|p■√▓°é}p■√r·B}ß╨'AÅφZ□□┤·▌|□}xVùá═∩µV o
y□I6$┘ÉdC≥&Æ7æ╝ë$□I0Æ`$┘ædGÆ□I

$9ÉΣ@Æ□IN$9æΣBÆ
I.$╢æΣFÆ□I□Æ□$!H≥ ╓â$□Æ╝H≥"╓ï$Æ|H≥! E□è$□I~$·æΣGR□I□$□É╝àΣ-$o! C□å$

╓█H ▌F≥6Æp$ßH┬æ□DR□IA$àÉ□BR□Ia$àæ□FR□I□$EÉ□ER□IQ$□H"ÉD )åñ□ÆbHè#)Äñ8Æ□HJ
)ü$□I$ÆH$%æöDR□I)$ÑÉöB≥

ÆwÉ╝âñ4Æ╥HJ#)âñ

Æ2H╩")ïñ,ÆrH╨!)çñ<Æ≥H╩#⌐Çñ□Æ□HóÉD!ëBR I%$òÉTER□IU$╒ÉTCR
I
$5É╘@□ì$□I4Æ:HΩ ⌐âñ.Æ║HΩ"⌐çñ-ÆzH□ iÇñ□ÆåH□"iêñ□ÆFH□!iîñ1Æ╞HÜ iéñ
Æ ªHÜ"ièΣ□$ ·□Is$═æ4G╥□I
$-É┤D╥□IK$¡É┤B╥□Ik$¡æ┤F╥□I□$mÉ┤E╥□I[$φÉ┤C╥

I□$□Ét@╥    I'$¥É— ëA□âñ3Æ╬H:#Θéñ
Æ.H║"Θèñ+ÆÅæ|îΣc$=É⌠@╥□IO$=æ⌠D╥
I/$╜É⌠A╥□I$}æ⌠E╥□I?$²É⌠C╥I□$²æ

@2□╔□$□æ

D2□╔`$âæ

F2□╔□$CÉ

E2□╔P$├É

C2

╔p$├æ

G≥□Æ/É|üd□Æ□HF □ëd$ÆæHF!□àd□Æ╤HF#□ìd

Æ1H╞ ëE□ï$□╔8$πÉîC2-╒x$πæL@2□╔□$qHΓÉ─!Öêd"ÆëH&!Öäd□Æ╔H&#Öîd□Æ)Hª
Öèd*Æ⌐Hª!Öåd□Æx$±HΓæ╠@2□╔

$3æ╠D2□╔,$┃É╠B2□╔l$┃æ╠A2□╔□$sæ╠E2□╔<$≤É╠CÆÇ$□I□Æ·Hµ#ÖÅd□Æ□H□
Yêd!ÆàH□!Yäd□Æ┼H□#Yîd  Æ%Hû Yèd)ÆÑH□æ$"ID▓

╔2$╦É,G▓□╔r$+É¼@▓□╔J$+æ¼D▓□╔*$½É¼F▓□╔j$kÉ¼A▓□I□Æ$$IH╔"Yïd-
ÆuH╔!Yçd=Æ⌡H\δ╛-23±ô╝ñ□
'E≡╔Θ▓$èT&╒ImRù4t∩□üÖ}%ÿ∩╣3fwσ─]Yv▀╨□NR₧╞p▀yε╛□═╝▀┬²ï□)OV╗╫□R«]E²S½
□□┴V⌠╓□pµ□G

%□ñ□iFZæ÷°lv_╥ƒ

"Cq]f<ÖDªæÖd.YD□╔J∩o□8
a.┌╪iΓ4à┘hs°Ñ;□à╣h[ÿë╞8¥¥«0
φΘ⌠rτƒÄ{σ□2□-¥ß70ⁿ□åOü°\□t»x

$□▀□□=□|╖√vu≈▀□Γ■àI≈/K■{╫□U▒⌡╧╝[□zv╨-K
□Θ□ñëêêê<$┤äPñ□ñ□:(MΣ·P|□□□□□H□░╨{/R∞T□i"ó□□Q! σ;s÷ε▓íH□y
▀ô²1;╡{gµö) sµ▄╣ΩìÆ:·ë4·»₧□BöO^#∞□╨≈¿µ
┤¥'R:ë╜-oQ~2σ°P~*σ▀ªⁿ4╨O°ⁿ,JgS:ç╥w(¥OΘ╗tτ{ö_@ΘBJ□Q║ÿ╥%ö.Ñ;ùQ~9Ñ+(]IΘ□pE5┬
o┤□ú□≡{□]]»╨@0BQ·^tö□û└─┬□╫T_╞Æ╪□▒▄□╧»U·□▐ûvΣσ¢í4¡<üW«@p∩M¥û≤b╪

∞╗ σ□╪┌─S╖¿≈□Zªæ6□;{\ MjtöP>─¿□╒□±[ε¿ªê╡ó╔sò≈åö╬└>°v¥å8>▒⌠Lδ,□¡n╘│·°╙═╡□□
¡6Ü}éö║p^
z□3╥

Ö)T2░K□^>-τC□jÑ.═íü□nN┴φ╓₧Ç≤╢è@÷C}Üƒ Ω·Uöà□r╨°!═■□u
ü╚╝□°µö□P□M□ΘA¥▄e╨ès+□n^K%╘é╥åE½╓¥╫S□Ñ□É╗ƒ╓╛;»)□y╥Q║à»æ@╕ñ╘_3n≤■Θ╬²U□Ä+╤┌
£Z:o╓·_S[É╢j!)V&╘p╖╘²U⌡□Θ½Ä╜.╪√½PT╙▌R°╫╒□ñ1Üµ╨αzÖ2>$|£╒Eë¿±s╡╖▐y▄Ix╝╫╖ Φ=
j ·z╒8(H╡□OΦ&┘x2╨¥ÅQ·□ÑâR É.╬~╧\┬¿≤□≥ñ┌jüÆ▒:
»¡3[┐□□□æ□ÿδ■ß≡Éü(V∩□╦αD>_ì{

━<╗╞4□!RàG;½□

╫»╖óªDM╟5jN╘rDa.Zm╘ΦR+N¥TP±τφE µ┼U3Ω¥¿╣Q≤□□□°□Γ≡┘≥-≡y+

□eo9|6q°¼y□|^╦ß╒·╙₧┴v□⌐>█Ñ[}N┐╟·£₧N°∞ⁿùΩ│s║╒τ£{¼╧9Θä╧>≈ÿ╧>Θä╧à≈ÿ╧àΘä╧÷□Θ°
lƒn╟τ¼{¼╧Y□1╩≡ú▐÷]□Ç≡╒φ?τò⌡&%j9□yò□)jU╘Ω¿5╩█□ΩÖ~à%k8xεâ{╥j└âÑΣªóì╘[,□6îT9
è7jµ─□YhM|□□≡≤□Q│ □D┬▄┘╙vU⌐g┤vzU∩¿>╪°&;}jß ;nïÑé[□\Σ5□ƒÄx
>┐X∩╛Ñ┐╪}N□@■▒≈-
²┼εs·□≥»{▀╥ ∞>° qJ□▄╥⌠W┐╧Θ»æ□·╒~¢╦ji╡▓╘[£]╛Ä╛ü>╡Së¿╞jm)╝ª√:°║Éo=ú║Γ[f/÷=+
╗╦▐bàX&ƒS□H▌ß{╬≈▓∩□╤Wⁿ S,□+ΣC□ÅE¿□(5▀┐|»æör@&DÖ□ΦD╞ZP□Γí-tñs▓□╙2-
│□ÿƒeçHûƒσ'\ÑN╓╓Θ·L¥₧@S□╣-
y╘╘{5╚■jä│â√"╝□#i∩⌠U≡╨nΩ□ÿ□│≡«┌┤o:ï!□c&kü⌐ƒ5ó|+╩'P╛
ª┘Yc╩7°δM(▀Æ≥M)▀ÜεQe□òΓt┐á;%▌c░fÿµ°╢|,æ╩6°{Z╨òûtg+¬A²jQ¥□╡╬⌐¼
)÷DYé╙6°╘□Φ╦UO╝%AΘ╦U╦╒┘□x□[╝≤:°ε□Ñ╫└√2└╫╞╫-
5⌠"Ω@╪àφ╥⌠$âε{┴o□B.G∩└□⌐,°·^P║≥}∩╗□`?n?□╢╝╟^□1ä≡+╨╛è□
w_[J~□▌ó&ù∩i_K_+Γ¿R□ÄR[R╤±ë∩8╥Q┘«Ät,╡ù:\U╞O8Ww[ú□√┐·ö%öⁿ╪╘ÖRZïo⌐²µ╛□$½Æ□g
!RUε╞m½>¬1▌⌐¡~XmKnQ¢'┼o8w8┐Ö÷b{1¥Pñ√□Ö'låìªÅ¬{i*d□┤Bb£OΩ╩⌐]⌐£╛σ╛□╛ò╛U╛╒╛5
╛╛╛u╛⌡╛
╛ì╛M╛═╛─╛¡╛□}█|█};|;φ2vY√A╗£]▐«`W┤+┘□Q¡è# n3□G┬⌐┤ç»°»ù»╖»Å»»∩Y£/√··π] ≥
⌡╝äⁿ□≤╝Γ√╖∩U▀kXªê]╘.f□╗K╪%φR╢┌½╘æª¥Hk□lC□f□▒N¼□ΩΘ3ùxWl□¢@╫6├!╓òuc▌Y□╓ô⌡b╝
Y╓ù=╦û│□l%[┼V⌡5l-[╟╓│
l#█─6│-l+√Émc█┘

╢║ô}─]▄═=▄╥#x$╧└3≥L<3╧┬│≥l<è√8π£
₧Å?└≤≤□╝ /─
≤"╝(/╞ï≤□╝$/┼K≤╟y
■□»╔ƒΣ╡│°?xm■□»├cy]^Å╫τ
xC-╟π·│ⁿ9▐Å≈τ□°@>ê?╧□≤□°□■□╦╟±±ⁿM>ü╧µs°;|.▀─7≤-|+ Éoπ█·

■□ æ çƒΣ?±Sⁿg~Ü ┬╧□]╕ä[xäW<&¬ï╟E
±ä¿)₧□╡─?Dm±ö¿#bE]QO╘□
DC□'ΓE□╤Mt□=DO╤K⌠□}p═zQⁿK

□╙─t1C╠□│─l1G╝#µΓZ╢□╫╣òb╜╪ 6π ⌡b»╪'÷ï/┼WΓkq@|#

èCΓ░8"Äèo┼1±¥8.²2╗╠!s╩\2╖îûydî╠+≤╔□d~Y@□öàdaYTûÉ%eyY┴▐bo╡?┤╖█ƒ█{∞/Q│╣┘3,
W┬τ□÷÷1;□╣╣Σyí□/├½CU▐ê╖é·ⁿƒⁿexÜOΣôí5ƒ╟□B;╛ô∩å«ⁿW~

₧□□┬□âD#╤□Fëªó□î╞u╡L□/ë□0Y╟□∩┴l▒J¼åy╪ç╔Aá≈╝'╢ê=≡ü°^£â
▓ê,□_╘b▓8∞ÆÑdi╪c»╣╫┬>{¢╝
╛▓w╪;αk√□√□8`□a□□▀╪{φ╝p□{`%ÿ└ª│□l&¢┼f│9∞□6ù═c≤┘╗∞=÷>√Ç-`
┘"╢ÿ-aK┘2÷
;├~e┐▒▀┘YvÄ%│≤∞□√â]dù╪evà□╫╕╬╗≡$▐òwπ▌y□▐ô≈Γ╜y▐ùÅα#·δ|□-
═╟≡Θ|□ƒ╔g±U|5_├╫≥u|═▀└7≥Cⁿ0?┬Å≥o·1■□?╬┐τ'°%~Ö_□ 4QBö□ÑDiQFö□□èró╝¿
*èJΓ!QY<,¬êGDU±¿¿&□ï

óúΦ$:ï."It□╧ï┴Γ□1D╝)&êëbÆxKL□S─T±╢X$□ï%b⌐╟
3╨L2│╟"│╨l2J·$ô\□)Ñ!MiI[>(╦┘□φM÷f√#√3{╖¡▐g%Iπ@□ùñσ□ñσ╥ñσ□ñσ╨ñσΩñσ:ñσ□ñσvñ
σ÷ñσ«ñσ₧ñσ□ñ=┴ñ┴╫IâúIwcIwπHwπIwo*╝µó°▀─"GÅ├┘□6Æ╜╬F▒╤l

{âìeπ╪x÷&¢└&▓I∞-6ÖMaS┘█l□;—

│#∞(√û□c▀▒π∞{vé²└~d a'┘○∞□√Ö¥µë╝5o├ƒßmy;▐₧wα□y'▐Ö□π»≡□≤W·k|8ƒ┬°≥╖·4╛ê/µK°
R╛î/τ+°J╛ ç∩σ√°~■% è□═□≡o°A₧╟╧≤
ⁿ□~Qd□9DNæKΣ□╤"Åê□yE>±Ç╚/□êéóÉ(,èêóóÿ(.□Dóh-┌êgD[╤N┤□²┼□1P

□#┼δbö□-╞ê7─X1Nî□∩ï□Σ□▒P$ï≤Γé°C\□ù─eqEé╘ñ.]╥-
=╥+#dñ,#╦┌δ∞⌡÷□{º²┌╝╦▐ù.⌠□vΩ-
≥▄□╫└└Ö□·i5Tg╞ùò⌡q▌ε*□`┌M"╞f▌=ïá¢o╔x║▐ê«'╨⌡KΩ║│∩■*┼-
FQ₧3/╤ê|ff┘└â╪ûA□─╢□2!N⌡C□ûôσ□╥╥≥üAx▐b┼X)░Y□÷
ΣbσY□╚├-bò!/½┬-ü□╪□¼&□` `╡í□½├bí□½Å□╢ÿ¥l_ä*÷e┐□h□₧⌡□±□á╔□ß₧e7°=≡Dsαt1□3⌐ß
·°,ò╚▒7┤□└▒▌□╝äíb┬0╘□u□█□9∞╔₧Ñ▀▜¬VÖ$╗╥_ú
□╜□êVXÖf□╤Y{Γ9£:□┼0ålíαÖ□Θë╛αIù¬G□M±ï╥ª┬å╢ -
ΦtÆÅ«Nè▒Éz╓├Z□║∞l¡Ñⁿ:╩»ªⁿ□╩»°ⁿ□òçαÖ┬▌□m¬7□f├u□5Åki<□\╫ùA<«∞+á□«ä□
□W≈π╨□╫╜╥0□Wµè≡è¼,ƒÇ╫d□┘□▐öφ░╓Ör║£□≤╠╤µ□Xl¡┤v┬jδg√□°äf=yN□¼φ`j╒εjL╒┘}╩bkΣ
Jáw█⌡S∩□≡5C¢╣#Z=]}▌ö┼φ□∞¢Θ¢e5│₧&╗¿;╒°■uO'»q~E⌠□╥e~╟□cU!□⌡╤□╧┌╓(≈·NI□¿∩»%M
°□╒╧₧ I╚∩HΦ·▀╒ε╓HΩπ┴;╥n╟?úf4np□⌡ττ┘Aτ9y-p≤▓╝□dα□±*ÉÖWσ╒
èWGî─□ü╓□ü╚│.ÿê:□Bv─£±É
□╬□╚->□█ Z∞□╗ □╤▄^(ó≡□□E4w

è#┬┘

σ╔≥3≡3èF╤ ª□R÷áu) zH╫δz╨⌡┐+ µ
f╪¿░{□²7¡Σ¿fƒaäk□¬Qÿf<ó□àeª"ÄQ╕UíVàY▀E┤¬░Ω□╢,

#□□┬U∞□@~A£p□□éB□□□□1`□ó?àⁿ□εC╝á░ƒ▄/□╙â≥É<*┐ò╟ΣqyB■
□ö?╓╙≥□·½ⁿM■.╧╨≤≥Æ╝é]╨e╕
Å□ad02□Öî¼å╧`å0ña□ªß7r□╤F-#ƒ±Çæ▀(h□6è□Eì□Fiúî±áQ╬(oTp≡I□mεB¼╢╧▐o-
░┐▒□┌ç∞├÷□√¿²¡}îⁿ□jε═E╧□î·[_Θ^_ü°D╘│□/╙√b╥n}□Σ╡T~╧≥°□I8-m2Dµ%□□ª#n□σ
╟┤ú╚═ÜZQV]½■M¿╘Φö-u«╥╨4òτ═σ□xf(êQ╫°□ì:Σt░╘$┤h□R9
WÖ□|□°·□>□rè,ó□«/Oè&ê▒□□_╖Gⁿ╘-·□W╞Q≡¼Ñ[^-
│□ie╥|V□+½&Q+>═┤╕eh╢e[9╡£Vn+Zï▒≡úσ│□Y┼┤□¼□V)¡░U╞*½□│╨[□┤□V%δ!¡öU┼zD+cU│-╙
-┤-╖jhσ¡'¡╞ZE┐╫ƒQ½σ╧∞τΩ⌠■□Äz9ì%╟□≈□è{≡(ε!âΓ-2)ε!ïΓ-|è{0□≈`)ε┴V▄C.┼=ΣQ▄C^┼
=<á╕ç□è{(ñ╕ç"è{(ª╕ç*è{lU⌡¢f╬\Q╒Θ=ÆE1╬

û¥σ`1∞□VÜòc□Y%÷0½╩₧d╡╪S¼.½╟Z│6╢z□Rf─≡□XG╓ÖuaI!;□¡hσπσe□Y□"ïπÉ╧┌ê¿╕□!ê║╘n╒
░■║ß6█╒Q┬╨è□εΓ□╤O□┤Ñ≤±g╤ûn└▀α□á
┘╥□·&╛□zêib.⌠C*╬┴?ÑQⁿRσ_âOe?┘>ù/╚i░╦□g^Ç▀¡╟Vf¡▒ò═╙ª5▒ÿ┼┤ªûiYZ3+ùòKknσ▒≥h-
¼ΓVq¡ÑU┌*¡╡▓─┤╩iëVE½ó╓╞¬lU╓₧▒¬ZÅjm¡ΩVu¡╝⌡äUSδ`5▒Üj¥∞φ÷v¡ï╜╟▐ú%┘_┌_i]²ÖⁿÖ┤
εa²»Ω□I□∩F¬│!╕H)╥Ö□Θ+¡ΦB¬*+jÉ□ñCQß╧Dr»Θ╪ ┌ε░=ìU$;║»°g□⌡ü'¼6åÉ₧▓æi&á±-
£;πCÿq□ª╤Σæε┴z□ⁿ╞8ùò□□ï╟─?D=≥¼%æ/╢╖╟N▐╥ÿ½╛Q□/□╔□┤╛É□9└┌╗□┘%%C╡g

≤6╬╟u~"«π╩δµ├▀<Σï«â=cF¿Ä└S°î÷□□¿F-
□N▐¡qä□□êdqYBÿƒ□1Ç▓αB╝|7ΣC╠ⁿ□¥_ª±D▐Öjq□⌐Vmh:┘▌:T╓□v█µT╢úA½□zOùä<ÑW²ñA□i└√
⌐|ƒ□□τ╖ÄÅ│ä(□≥i>∞x1ô─≤JF!□┤H,&o%I,╟Gëÿ╟Φdt6║□IFWú¢╤▌Φa⌠4z□□ì┴╞□πEc¿±Æ±▓1╠
x┼□nî0^7F□cî7î▒╞8cé1±&╥║FF┤ôúzh ¬rï·□-
¢<╝□W∩╫ë└□¬ß╚»Ä╢K□┘.y¿┤║□¬¡4ìτj8█lÑÿB╗≥Tôw∞z╗uδ▀Φ5□ó╫δ¡δ┐⌡ö-⌡□8▒9╕é□H(║□δ─
║│▐╨Oçs┌p╘□╬k(┼ql□╕╘■□▐3ëM□/{¢Mçîj□C▌═g≤!□{Å-
å(╡≈âêQ═□6╩≡uxX╔□¬áⁿ&└úJ~Pì≡─π8K&C;öπEh/.ï╦╨ τ╠

╨□Ñÿ□ ·α¼ò
₧├Ö╦å~(┼r╕≥ƒ3*└Kè;╪«╕â¥äE>Vⁿ┴°╚ß1°▄YIó(6d.σk□τµkv7▐f╙╕┼m£ùrα╟□├≤≥□╝□ ÿ□┬?
π_≡]|7φK|σ∞I\□╣D┤ê□□h╟í¡□ê╝□v□╘lΦ
ùo╝ï {╩□ñô¥÷>P;║4[_3g*J╒3gô╙!═:x□u□F$PT□□ƒó□□╤-□ }I√≈□L
δK█╥!□εδ╫-Ω?╒□Θ╟äÉìz╖èN╕□□□¬½-╣□G0Ä^√└u≈∩╕ε~%▒}r-
J∞K·5óôoΣ□╘÷w≥,dPπ□▓C≡ì#┴:v¬6hùq⌐X.öG_╟□╧□`┐>□y£√╦╥□XöJ¿╪δîj□eìY□╓\Y□h ╓│
Γ□┴ò▓TäaIgƒÅp□╬ioΓ╝u σ□@□{σ□pLe6,úÿQ╓x╙¿l─□ìì°ìµ

>□∞`ÇI╞□cì±àq╨° ╒£b╬6?0ùÖδ═mµτµ~ç« ╒HWö□Iûu

_wf┌YAd¿vlX/£7· Ä⌠▓σ∞#£≤□ß8╖ ^,
%y□>□-ß#□∩╟!╓Ö
¡□δlä

êv╛ àε$^{n}$;■□⌠ß▀≤ƒá/ Ö

âTD□<≛/
^@

□□Cq,uå≈□√⌠üàê□å┬*≥■o□δ┼f╪î(│1l'ⁿ·▒∞-ƒàO¡╡╔□╪eo▒?□5□≤▌╝Åå·┘-
z□⌡░}7□═kC║²°□V*≈{∩ÿN(}■5±
╕┬ßjF▒□ê░□k~╢?ë□╪ °╜πô Y⌐╕£5»ªπ9╠Ç5/├94æ≈□╔ç±1P□g¼╖í<╔gCUU□Ωa┘═╨□Q=nêG\│ƒ
▐ΘÑú5ês╗▓╤C∩
-□Æ≡°┤°□≡6(»□ô╙qf⌡¢□═uÉd□ì≤δΦ[╓±Y¬Ω╨h]ü░⌡<qµ□┘□Z<rà\
□σ

·)□O╒¬JX
D├τX&2░╟MÜ╠êÜ∞□ÑIçUIo5e{╘[mô¢·ß)╥[½¢┤~ò┬Ü□┘ï,ó┐sOî▄,╖CAπè╔á┐u└:Ç╓╪%δ□<f¢╢
╒φ╞vcx▄N▓ôΦ▌□⌐┴□è■□

6°□╦°□#□□□æÇ⌡¥ôτ□□□A-░·â╝□°ye■0Σá¥»\┤≤òç7Dï1å,╞╝▓>≥¢O╬ö3í¿£-
gC1≤î·+╥╖□╣.I+{à╗α+`k¬□Ç═at°ì_1Chw;/JKíÿ╝äáƒ
□tá¥δεµ/╚}?┌╣-H▄Å┐+ε□6£è1╪·?╔□ptTú╤□ëπ
sΓI□uéFWîu╨:□σ¼+╓□(oπ?¿`7╡¢BE╗¢▌
*Ñr\╘□├╠╗ε¿□ñ≡Ü╪°□□mr¢▄.w╥═&ÿÜΘ2=f□3úÖ┼╟jf3úLƒ╔LaJ╙D□Σ4sÖy╠□3»Ö╧|└╟o╓5δÖ
╠8│▒┘─|┌ln╢0[Ü¡╠D│ì·î┘╬∞`v1ô╠εf□│°┘╥∞m÷╣:^┤æaⁿñM□)é°E¡#ïp],╠≈≡□Pë'π·Wç÷+□
D□1□┌"□~□Æ─√b!

□╓╛╝HVµ,9P

é╣rí\

≤σz╣□□) □┬□╣█╕

╥ö□╡ú(Iùv

ÑΘ╤╛S□╒ÄúLÖvBIS;à≥4╡╙(S[√E╔U;âÆ═»²ªd¬]@⌐6╨.ódπ┤KJ║┌eöoó

J▓║□e█NÅD·v╨3(□δ□Q╩}⌠╟ê╕□δ╞<o^╨□"╢┌¼╟úU≤æ▐╟■╘■Lo«¼|╜Ñ▓â⌡D\ï╬Θmε¿/àìzm╚▀Z╝
o╡¿╬┌)□Wú⌡w°≡óóuh╜c═Uhà╓J□¡╨cB≈½H╟v¼□Ω╝□δäê┤;δÄ6eo╓□\h╖[Σ═⌡âçVk//└
@□/─
A$/╓KB□≥∩f$ «ƒVδ"du□%tTY6ö
í╢LÉ  ≡ö|ANç:┤èw°U╝□φ2⌡╝ü▀╖N□å╪φ¼□I┤*ÿ╪VvPts□▓
åêå¼╕*4╚╣6N╞í─&Üô@╥┌`"╖k└□□■τ¥7⌠?½╙,KçI6ï│□ób≥)"-
1X□┘┘|╦£|ì▄[ó┼╜Oεù_Æ□≡{┤╛O╩S≥g≥□Pts!□α9Ö,╧╦
Σ²ïD╤<ïæ═ê" -
'∩ƒe╪å▀╚NPts╕Bh╡□7J□Ñ╚≈WVy■╨R□╥ÿlL1µ□≤ìw╤j_h,6û□Kìe╞rcò▒□¡°╡╞:c╝▒╙°╪°□-
·]╞-c»▒╧╪o|i□0╛A
 Éq╪8b£4N□?□┐□┐□gìsF▓q▐╕`\┬æ□ÿ!╧┬□£!□ªZ□¡┬bì╟▓┌8fGæO⌡╝╕äk≈

╘■,╘²□·╬]≤»|□□□UîGîjFm#╓¿g─□ì╙°q╖£▀m»╔E╓─┌▌½a∩φH□g└□Ñ÷F□¿▌□ö╟L□□)╤□Fg┤SzO
╙S┤D,╡Z▄└║Tw╫°{≈bK□□≤(═öàiÄ¼Lsds╢╫¿□ë8'¡â-
j6éπj□éôΣO=E■╘╙d□)ï□╞d39dô¿□║óα┼□V[□e╡æ╜□M÷Z!▓╫*Q╣╧d╡=LV[U▓┌-╜ZÆ╧H*ôï╧ö@=
WÇRTª>òëú2±÷QñI┘{æ□[ì÷à□ß¼∞ä░èÆ□èô
WRφ╒b-□mò

┼W╘ü`ⁿµ>çéÅ□□□□□eêéΩDA
ñαChG□t!□Æêé«wE╗ï∞[ √╓ Θ□₧≥RT⌐╖≡
'Ω÷;Ü¢FÜ□ÅÆMûæ╪å┌I,üÜ3í□i«'jn□îA═φéq¿╣}0-5w□▐╜+□u≥;□┘ⁿ╥╤y□B─@tæ
J■êîdë┌dë>è╜r%─Æíæ╤╓ÿ□═p\]å╫∞-
φS≡₧²╗}Ä₧å╗S║ετ╛ùR╗_■□π≥╬9ê╕EIÑ¡·ñ¡F╬∞òƒ÷è□≤╧Wj□□±_c╓□┬#Ü╘3
Ωy-⌡╘╠□>╥)Y0¼Σ╫)J║TIK¥1▌,⌠4Jp□<≡╟ φï□#ÑΦ╔□╡□-xb$░-xR$░'εDR!.|₧<▌∩co\r
□≈├╫x╝ôR°╜□Γ:p□1W□≤!3╓l□≥}-
1□P□╬jß≡¿v□r8<- ÖHK┼(hL╜╡L□#ì╪╨,-|*⌠□⌡╗z{□xÖƒòe□▓µ¼□
╜}╒f□-

╚╪□<ì┘▄è├;╝╪Z9l╡□□°≤╡â+±!jSP*)-FÑJZt"#-
íRæHΘ≤á▌□ù▓ûα□Γn»₧α╓╠║°IFS¼i~zVXφm¿╜□?□E╟□TÅAi{□>╥í±|╫╘ÑëU¬FqB£G¡eGφ╝Bö¬≈
╨VC:
╙LΣå|H{.≥]]Ñ⌡NkWutäáùφ0ªàS□Oô┌╚□√Æ?╙u-□▀@ùï╞ùï╡τ{ÉVujj

╩╟àz⌐Ç<╗Pè╡íìl□xûB,
σ6çr+ⁿ6µ:¿_C╣═í▄□┘öv□ù9▀¢¥ouBTαy"╒πt°⌡(ⁿ+-
~τ╟÷╤·²íz²íz²ízƒvΩ}┌⌐≈i°^╒ïâ╧╧î%⌐-I├╚╨7╪\1F╝Ñµ╞□╠▌╨?CqIPîf≡□4âùó□╝4═αeR≡3
Ö°9ÜfⁿD▓ël□√Ç-□»ï
╕>l╝K╛û;|╜Æª|)|M<□⌡î¿╧K╘τ#Ω≤□⌡□ëεBj$±6╝□∩╨{≡╫°Φ□·Öû╞·╓╙Ns¥/σk·~~É-
σ'°EíÑ╥□Σa#±≡mÜ±αgô┘"1VL ∞≥à┼╕□÷u«ïo!l≥╫î»Q─√▒4î╤~ï-╝K^6°□S▀Ñ¥τⁿ

y@)╪δφM7ÿ╥Äù█¢t▐ûw┐A┐J+·U┐ ·ü²æ╨y@╤[%ä╟Ä□║*,¬ñót┌Eí°ñ·{ó╡¿¿z-
╤|éh.&-M╟4°î╔■ü·╒\┬6⌐█MVekP┘i7y:¢ë#a6┌â.▓□□┘â9╔-,N6dI▓!╦É
Y÷«µE╒~╒□╬■╧mÄiσëj¡"▓╤╞∞ï╕⌡┌·N▐A}»≡ßX▀H>□<a≥
£'≤□\√□·φ╦°□╔x8□ë2-┼ªíîò/GÉ/''·rèô ª$·o╩É ª∞m╡SÉ┌·Y·ò╨j>äφá▌î÷╧»\wΓ
╨R[SÇδ╙ä¡□g5╨╔□wP╟/$█┼|%╩v5▀□□╖UG%¬π

û±╥«□áòƒ

B┘· ╤╬╟╫└V╖&ÉCφÜ□N□║M·~%═µP;├óñ¿□ⁿ

Ω°ì¼╣└)□tf╙m╒□xrαw▓H□▐V╔└╕?K3╒R⌐Nss°(█_÷□FÑ°uφ¥ú÷□@JÅ▒N°½]▌-
JN│□R□¬╖╣¼v╓□□╗á]□□°à▀ä8┌wXïrP╪╛h□¥.ñ│
╬┴║╒□Z╪╔8G«H3|Γf│╪,ñEQ»□⌡
ë·xç│δ%}>
%=²6$}■╛Æ⌠tG╥c┬$}!=$φ□s╘⌐P╖!φ
≈ò┤Θ|~¡▓â▓╙V┌Ω┘╒╫┘δ╪¬óXcç┘aDGg╪□┌sI□7∩┬╗Çç□π├pm□┴G
^ÿ┬°@$ƒ╬°#«Y─□AF╛è»éLⁿ<?□Ö·e~□▓ê∞";d□%D
╚&□Dcê□ë"□ÿ≥ü□□²E□\╤ƒ□╧â□∩π∞j(┐3╪tµI

@┤╫┬j□╣Ié╤$┴₧$┴▐j□°▐ü²@kÿ╬√┴²+█ö√°□`Ωa│ÉΩ.Hφ□┌æ½┴□"╬P{║┘□#F]τ»┐¿z╗Γ□2╨Ira
τ╚!╒
▐¥α▄¡₧▓╟â≥Üàr:□l σ1□σ░□·┐L╗□╚oh╟Γ}±üX□╪╒U▄!o□æ»└Ω₧
W≈hΣ─Ck|□¡ε╛P¢u£6/+;µN┌t╬φq9ê@EäµíSV╒Θ¬≤a)¼ç□░□╛é├hë₧Ddû

ù5╖ûQï╥

-ç□ú□┕èke┥JZ□¡ÜVC½¡┍╫Γ╡&w/□┃□Iα3╪ïΦ|/□Çúhí)D□HO!□sπ
¿□/╕5]ï┕▒j□s

]ÿ╤Ñσ╙□k%qå*¬ò╞┤┤V□╙□□Z╥HmuL½k╡0¡Ñ╒┼┤«□Åi╝╓

╙fZ"ªëZ{L█kIÿ&i╜0φÑ⌡├┤ƒ6□╨┴┌PLçj»b·¬6èóª4
╫□m,^□»M╞t▓6□ V ▒∩hs¼Å▒Wµ╢□»╤□╓F□sl}êi£⌡□Γ╣□╓'⌠δi·⌡3╧ B·╧)□å≥¢¿╘6*⌡□] ò«o
ªδ█Θ·.║■□]▀B╫w╨⌡▌t²w║╛ò«∩ñδ{Φ·Y║╛ù≥τ(┐Å≥╓ö▀O·≤ö Æ≥□( □σ áⁿ╫ö┐H·□ö┐D·âö┐B·
ƒT▐╬D·Sö╧L·├ö╫(□ö≥.╨ú╝ç≥▀Q▐K·πöÅáⁿ□öÅTy;#Ñ·⌐┼∩)²æ╥ôö-í;u*⌡-σ▌a≈□CΘ
ò╥°Roo¥C°l¡Ñ1v%φVaK│4Zà□éf=oì□ïµ╣bä,FX#≡╖1í□║Ç▓pΦ∩□▐╚O▄□─ìèDÜú╕╤α□⌡
ªàçπ╞▄(Ω□▒°D╢åC─▓Yc¡q╓xké5┘ÜbM╡ªY≤¼w¡≈¡□╓bkÖEτ┴╤ ^┴hⁿ╨iG⌠□╖n-
╢òφî≈\□í«)ⁿ°▀èPWö□└Qr5▐|m□□i╙╫")╥□(╥:□EZù!^Γ(╥·ièªkKæ╓□êï■a\\ì□_□_≤íhW'□□
"^nefπΩi□┤«╪v¿/Mp╕wCzë╨

°1□½¥9DeZΘH▌4╨W┼≈&PtLKè∩φA12}(Ff<┼≈Nó°▌╓□▀;Sε5.├l□a½}F□╢î"l□E╪·)┬6□E╪µó□█
hè░ìI!ò½╤╧Y û╥5╜Ey╚≤;R±└⌡1─□DY?[┐X┐Z┐[τ¼≤╓╓%δ▓¡┘║φ▒#∞H;ú¥)46åçΩ┴Ü kq╞╙á

Ä°│╨ìb4√ñÆb┼{]╝wP□∩9pmhït□╬═Θ□89□τ□□Wï∞╕JΣΓ╣yt ÆS┼c½8∩¢□═█Å"M-à╕ªú
~ò┬?Å╟²τ¡O+A√π^╟*W4*□UìGì╟î□F£æ`47Z□ëFkúì±î╤╓Φ`t╝τ╤╞─ôo Γ·$J╪ƒj
7·ôêτ ?1╬╒--±┌1Gr^L┐□=]□|Ä⌠□¥,¡╬òV'J ╞~íôpéταh╝
∩╩≈±#"Z□□e┼ú╘k╒9□
éQ╨╫⌡α

╫£y□<àcÇ▄ 7ÑâР▌ëN▌ΘE°φ

╖■ö¥{▌≤═ófE│¬·□«□S═╖═iµtså9╙£e╬6τÿ∩ÿs═yµ|≤]≤=≤}≤□sü╣╨\d.6ùÿK═eµrså╡╥\e«6╫
ÿk═u□□₧┴:m]░.Γj□╢╦v█^;□y□□⌐²U╜eÿε□]½√0]ª^ç□δ∩□ZAIw╜±YS8~□gEà¥□u╙Són%°;>EΩ
ûⁿY⌐α≡&╜

√£≥ó~□Ωu:ÿ8□ÅFî1ûìE□7òMÑ¥┼â8█%≤ïα□┘Eq0□ú╖ç┬ê«□A)£»G┬â8gÅérj▐å≥8sƒçèΓÆ╕□*r
°□x□g▀jP□gα┌≡(╬┬▒P
gΓz≡ÿÜìí:╬╟ìí□╬╔O├□⌠─iM─^τí┘m╧▒7π▓ ┼.t

□[æ┴│{□≈$╨sOΩ|╢öδÉ▓C┌9= lM║·ÜrS□R£V⌐Eñatτ`╢Nⁿ¢÷╫R╥□Öf4E▒í∞5╣à}─□≤f╝-
╚_Γo≥O°n~Æƒ▒W█[«ú5CÜ╤ΩστäW╛,ª▀É«îiFW□□╞╢ë╫─Yq╬▌ï6÷╡öeJ3╧<ü□╜□Rò9
ú╦^f^GOû┤□ù╝)`»▒╖"·█□G⌡┌z}=Ao«'Ωφ⌡$╜ù>@ó┐¼□╫╟Φ□⌠i·□²□}╣╛V▀¼∩╨┐╨≈δ□⌡c·
²ö■¢~┴Ñ╗"]Y]┬σwE╗□║è╗╩╣¬╕¬╗j╣Ω║□\═]ë«÷«n«>«□«!«ù]├]c]ô\S]│\∩╡□╗V║╓╖
╡║>rφv}σ:Φ:µ·╤u╞ò∞║Φ╓▌æε(╖p√▌╤εⁿεóε╥ε□ε*εjεÜε:εåε&εûε╣ε$w≈ ≈P≈p≈(≈x≈d≈

≈\≈□εÑε╒ε⌡ε¡εÅ▄_╕≈╗□║Å╣O║□s_⌠╕=Ö=Q-├ô├□π)Φ)ε)δ⌐α⌐Γ⌐ε⌐σ⌐δë≈4≤$z:zÆ<╝<□<C<»
xFy&xªzµxµ{□zVz╓{┤║y>≤∞≈□⌠□≤ⁿΦ9φ9δ╣α□╧Y»╫¢┘╤╝û7ù7ƒ╖░╖┤╖Æ╖¬╖å╖╢╖╛7┴█╟¢êi{oÆ
╖ù╖ƒw░w¿≈U∩□ÆEƒÇ□╝ô╝╚╝s╝≤╝
╜╤╜k╜¢╜;╝ƒy≈{□{OxO{ô╝ù#▄□æ□Y#Dä?":"□D╤ê▓□ò#¬G╘Äh□╤,óMD╟ê-□²P║ôPr╓H√£ê┴□/├
╒≈ƒ¿°├$╜τñ0 üƒâ▓B□~xL<&Γí□╝τñ¢Φ&·@_z╧╓
è□{ë▌s2Æ<io╥·r□,4√Üú`9╜≤dwα|□z≤╓W÷╟÷'≡╡²9╜}hÅ╜□

æƒφ□Φv□k

Ñφ)φ□Ü]çr/R┌ü╥╓αRwXKΦ»╬öNÑ4ë╥iö╛IwurΩ¢Γö
|╖|▐á½∩R:ÿ╥y⌠╦@‖?╓I█S¬¿êÑ▄ïöváTQ□δP□KT─□□▒DE,Q□KT─:T─Rδ▒

□▒DE,Q□KT─:T4┤┌a╦
)7□»π▀╬⌡▐öNÑ⌠YJ▀át□▌┌¢εèú╥qöSÑπ£╥qT:ÄJ╟QΘ8*□GÑπ£╥±╚╗f╟S«□Ñ╜±W╜jM─┐□╤»ì(╫Ä
R⌡k#·U│□≡Jé╒ò╥~öNÑ┤□Ñ¬÷╞VGJ°Γ▌ì)7é╥εö÷Ñt□Ñ├(} k╟□∩□|≈r╛▀p╛';▀}▒╛&Xó
╡▐äZoB¡7í╓¢PδM⌐⌡ª╘zS╨ìá┤;Ñ})¥BΘ0JUδM¥╓¢:¡ {g□-
Eæ φΩ╬$B&□s⌡Q□"F□!r⌐Çêê╨%□"r╔-
á$!É□Ää□é□#ù□□□□æ>D□.""╖óêΦ▓«δóóé"╖èèé□╨Γ□╛U▀⌐ä áε▀┐╫√╛0-
₧¬εΘ«¬ε~║╗z╥²δ^▓÷^▓÷^▓v▐□d□╟qα▌áX█╜╤å▐µâαT>]-
₧╗□╙▌ëΘε─t}╟EαâαT╬╛|²&□b═÷┼ÿ"9f□å□╤íYH∩6É⌐(√

╠}ç£√

î)ÆcVah░□▓æ╞µ╛C╬▌╧\□Ä□╟âÖá╪#·!7
□
□é

Φü3└b≡Y^2/
φ∞'▌∩Å□·úå■¿í?jΦÅ□·#gâc┴Bp!╕□£□□âó□^□ûÑ?ûIe□▒¡□b[□Dn:8□□
║αdPlδür[□ö█zá▄╓□σ┤║-êm¡▓tö¥Ä▓╙æ¢

□cS╕αdPö¥.╤Nùe°╤▓╙e┘Θ(¢ëó□∞╡"┼▒-
¬ë#â□{½□{+╞÷ï□(ú?R±□m*▐╡ÜC□H!εöÜG□É%d%y₧l─▀·wÉ²Σ09í≡^┤□PLÑ¬RC⌐½4RnP┌(□önJ
_%]╔┼¥□Γ□X□q╗²ê█]à╫┌æy╝╢Äµ`₧·x╩Å+rî'╟

Ä╦ò~ù#╙A2¥ ╙Ö2□╧─□▌╞-πC╗±▓π≤▀å▓cc<9fp\«⌠╗□Ö

ÆΘ□Ö╬öΘx₧¬¼□jΩdf╚tûLc╥╥⌐l9:í⌡*δîí╫r ·╫r ·╫r ·╫e╙w╞⌠τ√Aτ√A┐^?(v«╫└╤0□G├

Σè└Aá8^d╚-H□z □8*dá□Æü-H□z □▓□Æüs|å∞üd╚úp□z □8║fá□Æ!{ ÖÆÖáhE,W□

□E+2e+2╤èL┤"□¡╚D+2╤èL┘èXφÖfi□kE&ZæëVdó□Ö▓□â╨ô□ä£8·
Æ=ÖA8g

Bmâ╨ô□äÆ□í▄A▓'ôà╡│É□sg╓║│0w□µ╬┬▄Yÿ;
sg╓║□ú°3□╡

P⌠t□╥~╨□|;□╣

P|;D÷â▓·ÿl£▌│╤□╔F]┘Φëdú⌠□£Arp□╔An:8□□
║αdP£ArΣ□$G₧ArΣ□$G₧ArdOD╘{╥□π(╨□çüó÷\╘₧ï┌sæ¢

□Gé.8□□╡τ╨┌seφ╣▓÷\Y{«¼]ö1□╜êæh├H¼φíh├P⌠oåó$ZSÇΘ□0]□ª□å│√0L7

² áßΦ╔

ù=Öß□S$╟¼┬╨`94
i¼'3\÷dF`ε□rε□□S$╟¼┬╨`9d#ì══B╬¥ç^J-z)yΦÑΣaÅ╚├-æç▄,p,X□:á□╬□ïA╤K╔ô²á<Θ~>j╚
G
·¿!5Σúå|Σlp,X□.□□ü3└bP╘É/√A·▓4□█z□╢║⌡(ΣªâC┴æá
N□┼║-%╖⌡(╣¡G╔m=JnδQ▓4□eÅF┘úæ¢

□cS╕αdPö=Zû=Zû=Zû=Zû=Z÷â2dÅ'C÷x2╨π╓öc3σ╪Lî═ô²áⁿ■¡-
T"√A%e²á□┘□*)δ□òö⌡âJd?¿D÷âJd?¿D÷âJd?¿äê~╨"┘□ZT╓□Z$√Aï╧·Aï╧·Aïd?hæ∞□-
Æ²áE▓┤H÷âä-¥PôΦ□ò╚~Pë∞□òö⌡âJd?H┤Ñ3å:╥Θ;╥Θ;╥Θ;ùM▀□╙ù┐».v'□²S▐WW■

:±VæΩ∞^6åìe≈▒B6Äìg≈│"÷□¢└&▓Il2¢┬-dS┘Cl□¢╬f░b6ô=╠,6ï=f□es╪#l.{ö=ck┘│╠FΘ»¢ç
xΘ5X
rΘ┘w□┼▀·ì╗¢ƒ□φPR╪ë¿ü⌡Ñ≡╣╪ew╖□Σ/~ò╧}#■□Æ╦╟°╔o□?zO╣ ╟▐9╔╫O□ΘdV0SIw│û┘ö

7¢±9'óM
▒nNê6ëÿ`▄à┐▓G▒à╗╟²□□° ╒╥?s╢é┐±r±5z╓R□□σ╥╧\ª╨o╛¡╞░qg-
UΦW/ ╟σ□ ╞╥ò╟≈╤"╛/Nì▀□╧Z╬≡o^▀Ö╦□·ìù√□y\Ü─Å9≤ÿ}╓□G~├Ü╫\Vφ7^╓□~Σì□w'≤cεC8╞Γ
□{╓Rk┐C¥ w£□⌠ƒ<3□□╙Ö┴°═╧

µpf0□π3□√C╬

∞7?3D □gåΦ∩~f¿ⁿ□ƒ□* Ägå*□Ü3Cò▀⌐╬╕h~╩E┬~~σ:Ö°IµN≤□□╚╥┘(~U,Ä╒>╥à_Åv
Γ┘╣□ë┼╖ⁿ▒÷⌐eφ╗□φ□å÷M└┌─□□e□<□╦Gëêxïè÷╣¼Ñ□⌐╔»╝Re¢¬■─└▒╚□Ñ¡┐ÿ─▐V«É□"╞▓xçú╣╬
|┴|╤|Iû▄£4"m□M0Rv«·⌐7┘ï≥ÄÆ╙æ·Fô╥╪äó┤T▐╨ÜÑæ□òj?9⌡□|¡Ñ≤u▌ô

πδp□)}▌Süâ ¢⌡êhê2å`.╕□δ°ç¼7ò╘σmL%Mx[eä_σ□Y≤╧╧&╤H□ÖM«$≤· ad□□K□╚

2/nΦ  ─╨kⁿ+-O,>Γ-¼╟V`v\
cQ□∩□τú¥┬FU▄²'å⌠□d½
H{╥ù/o□ ▀äo╓╓Σ□╛w▌□7ö╥╖Å¡ì╘╕╡±k╘NI

±°┌₧╔ Å
ú╚□2üτªÆL₧/Σ╚╒²u╓T□m□O□WáMâΓ┌ç¡□┘tv+═}▓òìxï╥╕æi|ÅhÄ=;ì∩ß⌡y╛ì\3ù₧^3 ╦┌┬■ö╒
▄□±|_□≈g)

Ü□çb9a╒»Θ╤└i⌡Ç_╨÷╔╝⌡
╕1
╕U▌ I><╧éI▒£p½╘ªΩq6²zmê╜φ#□┴@╤0┐vτ□=Φ┐╚∩T~T¼¿
╒åi╞╡□Z₧û»ì╘□┤{êx6|dñ
rOdTΣ■HQΣü╚□φZYå╕ ·¬□ï□úêhƒ▒╖∩è·Cáê□¢ö$Z⌐□□□KΣÖS□I□╔┌┼3¥ë _$|MHpXp4I

N□N!□û╞«$□[╓V"Γ¬□\□ûö=╧╘¼l¢ Æ2||Y┼τ□""≤ⁿ╥R□·æ9÷□∩,n·ï╤□æoK?-
ë"π╩■ÆÆ□╓lΘ:&J═ ▓ä□l¡ █ä╞qδ°ùû╤ñ▄:■ÑÑ4=c□ ╥rÜ¥╡Ä τ%ë╓Tµ{±╝d<ÖäH□se,üσd-
YO^!»ß⌐■╥'·σ3ⁿJè"÷à╩╝□ÿÄg½b╧╫∞
δßΩß½┬¡┬δ┬∩åO ^└─╙Ü╙&¡úv¢╓U╤╥S⌠-å0 ü-&j(□╠□□ü╣α0p8╕ù-%jXgK8½│°8»B╛□╕

|ù=╓yB02@╟□Aiæëb▐╚□1Mdôá╓Qî╫n□╗èo╡,æ╫S└─
é╞e|J╤ôJΓ╟Ç4~<n┬╥R[▒╧çÄäÄë°□¼/□╬ùⁿ□[╩₧µΘ▒X□─ ╙∞X╨°Z╨ûë┐\±⌠□R□┐WⁿÆ«j╖kΓ~φ□
D⌠HW≡2Ωç"l9±àt│cH\²┐J·+Göπ╩w*Qô╘□5¿Ωje⌡R╡V┬═ëw&

L╟H□ö8<1/q$╜ä^J B/ú╡Φσ┤

¡G»á
h#┌ÿ6íMi3z□mN[╥6┤/╝ïª╙LÜM∩ªCi-□I∩í≈╤q⌠□:ëNí□╤Θt&╡Φl:ç╬Ñ≤¿C=:ƒ.á%⌠
║ÿ.íKΘ3t□]E╫╨gΘ:║₧╛D_ª□° á ñ ó ªo╨╖Φ█⌠]║ì╛O?á╗ΘAzÿ-Ñ_Q□·¬┐rX·Z·V·AM
T²j@╒╘¿Ü¬╓ΣK1 ±«─⌠─╠─aë#□≤i5ÜJ½╙□┤&Mú╡i]Zƒ^E□╥½Θ5⌠Zz□╝₧▌H[╨¢h-
z'□H3Φ□ÜCsΘ□ÜO
ΦXZHïΦD:ÖNÑ╙h1}ÿ╬ó□ÑÅ╨G⌐M]·□}£.ñïΦ▀Φô⌠)║î.°+Θj║û>G_á□ΦF·□▌D_Ñ»╤╫Θf·&▌B▀í[
Θ{t;▌E┐á_╥#⌠□²Üê□÷ëJzbÿ~ùt¬┬⌡|⌐□ò>╩@▀Üñô□─╤$ g9U□ö⌠□å₧□├ó_¢⌠├q±KÄ»¬∩2∞▒Γ
□>Ñz¡┌;nΦf5ï╧²□·ÅZ_m¿6µ▀╢αππUoS╗⌐=╘▌Ω]jûÜ¢▄=y

■£ƒΣç╦□x)σ?ì╧■$») ß╡₧√╙ΓîOk▐╢≥ƒ[╧■$. ß╦≥# ñ≥-
╛╟σ?=╬⌡±?U■├╫R·O_|N□▀u╞'â□▓~Σô{«Å T·OJαî□;πôZ■#ù/╓^ö└╖~]⌡□⌡□~±┌HmD□⌡□⌡□nC
S╡)IP[¬-ëOm½║%ëjG╡#IR;⌐¥╚□ΩφΩφñé┌SφI*¬}╘>$Y□¿

$~u░:ÿñ¿C╒íΣ┬Σ-╓=□M-¢<ûTJ₧ÿ<ô□Æ¡ΣGIòΣà╓½I⌡Σ╡╓/ÆF╓ L■Æ4K>Æ|é

⌠Å÷O$┘■╔■□dö□«□ □∩_Ω_Ef·_⌠┐Ol N N▓╓┌█┐¢<δ▀δ▀K₧≤□Φ É¼≤□∞ ÿ<∩ ─
y┴ Ö 3▓▌ ╡ s≥ó á ┘α ╥ %y╔□─□äl⌡-
≤ƒ$/√□HI"¢Sé)&┘Ü□M╡ä∞J╡4%ì∞°□⌐ƒ□óòhÉ□í&ì"Zƒ8'u:δ╙²¼O▀|>ⁿ╞Æ</y□!╔□Æ_%□ô┐±ï
#╣╩≈Æk9□â╖rè╝□1Φ□□╚WB^─╟OJL*δ╡╞□▒□╡üÑ≥ê¥□xÜ□╖□,□öπDÅTI¼╚Θ
▄─╟▀D─╗□R°╣π□▐cnÅ╓≈σ=Σ,▐s□g[~▄□T□╘□╘
4

4□\□h□╕ &╨$p]ásá□ƒ╗ ·àWp╓□δé
┴Fα╒`c≡□░   x□╪□∞┼)Γ-
R\┴tα╫2=±≈≤!ⁿ*úÇ≈αïd|;z╬V┤□┤ ²□Z╥□l{f½ö□r┐2M▒ö╢è°ö(Köσ╧Ze╜≥è≥Ü≥ª▓U┘í∞S>U

)_)▀╨s_H5╒¬ju5ìo┼F| h«╢Q;¿]°^╨OMWç¿├╘□u¼ZñNQg¿|╒y╕7o⌐‖R}N▌ánR_/╗;∩ÇzX=«~_
εε╜□ ╡□«Lh\v□^╗ä▐╕'-'aD┬¿ä┬ä S□f&╟Ip□□$,NXû░:ß·äì
»&l╞□z{□÷≤╛û╕C∩ö╧τK÷□|‖»è/╒W╙W╫╫└╫─wâ»╡»╝»ô»╗»»∩._û/╫ù∩╗╫7▐7╔7=g·─_
#┤─è▄□┬╥□7)╨!└»;□¥□¥╔σü«ü«ñNágá□⌐□╕#╨Å\□□□□H□□2□Yñq`H ¢4
▄□□JÜ□å□åô□□yü{H≤└Φ└hrS`Lα>╥&0.0₧┤
□□è╙-ü üIñC`j`6╣-07░Ç⌠

ö□^"□DM¼xa}lg┴┐Ç5└╦└Ü`-0
╝□¼
╓□δé⌡└·α□αòαU`□░!╪□╝□l

^□6□»□¢é╫ü=└δ┴□└□┴µ`
░%╪□l
▌□╢□o□█éφ└÷`□░□╪□∞□÷□{ü╜┴>α□`?p□x'x□8□╠□3┴A`□8□╠□∩□s┴íαp0□╠□Gé□α=αhp

8□╝□□□Ä□ï└
αDp□8□|□£□N□gÇ┼αL≡a╨□gü│┴╣αúα<╨□□╨□=≡1p>°8╕□,□□üOÇ□□□âOéK└° └Ñα╙α2≡□
p9╕□\ «□Wâk└╡α│αsα:≡y≡□p=°"╕□|IÉ+/
-□■'x"°M≡█αw┴∩â'â°é?□? ~□ⁿ,x
°y≡ïα┴αíαù┴├eπÄ□Å□Å┼╞▒V¼5╗ë╡a7│╢¼□k╧n□≈«ö╗&+□>$»╔¬╨·v□w□w□≈□≈□≈□?

~□n8╕?╕9°F≡═α[┴-┴╖â∩□▀
n
n+□≈^≡²α÷╪8╓ö]╟Ü▒δ┘
∞F╓£╡`-
□▓>±nú╦ΣYΦτ»·J╟┼«²bπ╩«□qùUu%φ°ù/ñ□wé╖┖▌α-p/╕□n□n□n□▄□n□▀□▀□▀□╖ÇoâΩÇΩé[┴mα
{α√αvA╓□╜

l□^□▐□▐□6□[Ç- Ç⌡┼»▀┴âα!≡K≡0x□<□-□┐□┐□

□çâ#└<0□    □Ç≈Çú└╤α$p28EÉÑüùâ╡┴:`]░-X╝□□□╥bo╨ë²éû□∞□∞□∞╧┐#┴>£
┴~£>>ª\∩JⁿRP┌╖╥║è_□┤-é▓□U□┐Uƒ▌ô:+Ω╖û#ε≥╘p╫-g₧(Y▄U╚· 8òÅ╟¥á£CA1
ε;µ|□□□▒LRæ╘<½^±╢╡╖CC
□□ê▀²┤!ª□Ä□¡5 åⁿ╜α}□â_3─¥₧|╠

░ÿù"Γ▌
╓─□ó□╨─R&ß□q%≥d|□2≥┤ΦO≥qjp(«&OΓ╖□Σ□╘▓_Uô╘oÉ□∩║¢G┌Ö¬Ö`·╞D3╓¼`V4ôM┐ê□'ó┴Öa3
bjªn23jV6½ÿ□ëêpf¬Y╤L3/7k¢u╞zf}≤□□□NDâ□▒αD$8□□NDü3o6█Üφ╠÷µ-
f□≤V│úy¢┘╓∞lv1╖Ü▌╟█═εfÅhR⌠éhàh┼hr4%zaöF+E□╤`4□
aw
╓'┌□√µ'╝≈I√o▀'┼N╞╣$_╖]░%âí>╪J
±█≤╜x≥?°≤q;°╘┴ƒÅ≥├Kú─çΦDG
±╦è□oó¼□«oîp 8□&ê⌐ED#·n╙╥ê□ⁿ¢ÖñuY\Äφ┌□┌

m°╢K█¡φ╤÷j√╫x╥τbφImë÷ö╢T{Z[ª=ú-╫Vh+╡U┌jmì╢V{V{N[°=»╝á¡╫^╘6hji-
k√╡ù┤O┤O╡╧┤□ê°q0.µ╟1φ+─²╪¿╝╠v░¥∞□&▐Ü╓║,╓╚#í╣íGC≤Bv╚
╣í╟B≤├fÿ±%¡┬ù55|i╕q╕I°▀ß═ß7├[┬oç▀ o
┐□▐-▐□▐□▐□■>R5R-Æ□╣,R;R'Æ-□□)µδsû\úx;

╓½□╖f┼{rD┤Æß┌╜∞E╢ü²ïm├_□□±ª»║ñ□~/NΣδk□_7▓□$A█□□╩≈┴□mçL≈╔⌠├X╩bπ┼√

."□H

□E&≡uεÉ┼d5┘H6ôφd?9JN)╔è«ñ*uò&JkÑô╥W╔R≥ò±ⁿ╨h.┐*èEÜ■ªm#äoÑ□╕O;╡╜─╟╫εqRæ»╖]ⁿ
□Ö□/jBmë┌√q·φq·□Γ≥;π≥╗Γ≥╗π≥{Γ≥{π≥║σ≈╟σ?ë╦□□ù ,.□
. y\■ï╕ⁿ┴╕ⁿí╕ⁿùq·├q·#q·úq·cq·»Γ≥_╟σÅƒ╬│╕u┬N»□□½╗:?╧ê;╫≤·⌡≥□~▐ƒ═╗a
Y╧╗Γz▓ëo╟¡d□■Æç╚q╛-ô□╧╗fΘ=τ═°□─╝τ<G╔□τ
n╨qB╕≤╗╚%q

môΘ{2}_ª█e·┴□«φöΘ.ÖεûΘ-Öε=╖ô┌G2²Xª√e·ëL?òΘg2=
╙╧e·àL□╨│ÉL┐öΘaÖ-æΘQÖ-ôΘW2²Zª╟╤φ+  L.Wl
≡╡ƒ╬Å█»æ-|_┘G

≡╜σ[Eσ√KHë≥=&M╣ÆΩ3═ò┤║|»Θ⌐

α√═0e□▀sª(3·╛3_Y¼,W₧S6*»)[öφ╨>σÇrT·VU╒d▐í□┐>°⌐W¬MT±ùù·≥mûê┐â7Q■⌠;(╥┐ü2■²ô
/Φ[─;(╥▐;╣┴x┍x╦xÅ$äª┼û,4]ª3d·┐L□╚ta,ì╚∩#Å╚⌠□Ö₧öΘ)╣□ôeJeZIª[╧╪r ëÑzèLMÖ╓æi
□Ö^+╙ª2╜Uª=d┌Gª}e:XªCdÜ-
╙BÖNæ⌐\~].┐>Oª╧╚⌠□Önæ⌐4[ùµ□«L□╔tÖL╫╚tâL_òΘ[2ìφQ╕▀▌4╩ö÷¥÷╜vR;Ñ²á□]╤U=A≈Θëz
□bOU╘ôu?"P╤┐□T□]□q¿╪n╢çφσ╞T ±3U┘┘µg╬58╧lï╡¼,nS□²"╜¬~▒^M┐DO╒/╒½δ□╤kΦù!ÜSÜ~
╣^□1¥Ω┼Euj¿7□æ¥╪>÷!√□-
+ì╤║4⌠thYΦÖ╨≥╨è╨╧╨Ω╨Ü╚▄ê□q"«VQ╛□N─Å□┼EÉ□oç□oå█╙^╞╗ßjô░6N□»▌»□i□h□┤ë┌$m▓6E
{P¢¬=ñM╙ªk3┤bm&█.Äl▒≈° □{□═┘□─▀5□!ë≡\

iá Çê⌐¬æ`°îD#╔╗ └¿Ç╪⌐~#Ñ\ⁿ╘0"¿Ωåaÿ□3óFeú□b⌐^lTïÅ°¬╝ó²]█ñ²□±X▀╘▌╥╢hoï╕¼Γ╫▀H
σHs~╬|V[Ojj»io≡s╕╧»╪n2F□□╞=╞(c┤q»1╞□k▄g□□πî±╞²FæqÖQ╙¿eñ□ù□╡ì:F]ú₧Q▀╕┬╕╥╕╧
h`44□□W□ììkî&╝╝□i»k ╓6ko─╫k▄è□»¥î╬F□ú½╤══╕▌ΦÄX»╝î ▌±±^q□Ö╓□è°╢H┼╙àΓ¡6hi*_ó□
ÆÜê>U╫▄aε ══ôµI╥é±╒@Z▓-¼□i┼rX

┐□Q·uΦ╡ ╞╡FSπ:úÖq╜qâqú╤▄ha┤4Z□¡ì¢î6╞═F[ú¥╤▐╕┼Φ`<`L0&□ôî╔╞□πAc¬±É1═ÿn╟0èìÖ╞
¥ê?¢nd□Ö╞ #╥□l

A$┌╖ì▄°h┤µnFXöÑïτ┴HàH=▒~#7G║æ□▀^5H¬╣╥▄E□Ö?ÿ?É½□ G□│^¼□╡åo▓\~⌡□δë>lX╞,C▄╙r
·mΘ▒o■j╟1-1µÆ°7▌¿╨
≥M┘{□E¼+□π¡╣▐Bo⌐╖╥[δ7Θm⌠¢⌡╢z;╜╜~ï▐ß£╤═≥⌠|}ñ^á▀úÅ╥Gδ≈Ωc⌠▒·}ⁿ□▐Q┐M∩ñw
╓╖Φ]□9½P°Å╫∩╫ï⌠□d$┤εⁿ(▀S∩Ñ≈╞▒■

╜ƒ▐?.ª\å₧ëXi□ΩS⌡çpⁿƒí□δ3πóª═╤-
AΣ╣°Φr₧■ÿ>_□_á/╘K⌠E·□·▀⌠┼·ôê9╖T□Z_å╙s+Γb╧=½?W.▄▄K·F²e²□²∩·&²-
·½·?⌡╫⌠□Θ»#*▌□·¢·[êM≈N\t║φ·□σ"╘φ╙?╘?╥?╓≈δƒΦƒΩƒΘ□⌠╧⌡/⌠âêδvX?ó-
Et╖»πΓ╖}°□Å□o□îùîì╞╤╞+╞▀ìM╞?°╣φƒ╞k╞┐î╫ì□□¢ì7î7·╣nï±╢±Ä±«▒╒╪f╝g╝ol7>0v□;ì]
╞nc□√ÿφgf░□∞s÷□√ö□dƒ▒C∞Kvÿ□aG┘1÷□√Ü□g ! √7m?Gz■□l8{█ ⁿûìÅë≈ï╫¿\□□·Z(]≥_■Ä
≡≤╦ⁿ ├2ù■^fÆ╥7╣½┴£P
≡Ω3□uô∩kÅƒé□op?²[ACóÜ□∩=Å▌δr·≈«3▀t.ªïé
Σ<é}┴;└~g╢╧L□

µ≡oS9E┐ □╡╓9w}σ▐╢¡F╛□Uµ<)¿Q≡bA╜□8ìS□┐u¥■MU⌐╧»╗Ü≥½□±┤o□~]╨Å_{
ßW□□╨XÑê_□╟Pf+≤°╒┴□╨Re%┐>╪álR^τW□∩)╗öÅ°5┬aσ╕≥=┐J¿áR5┬»□¬⌐5╘┌ⁿJí▒┌Lm┤║U;¬▌
╘▐e▒@F⌐àΩ□u¬:S¥ú:Ω□u▒║L]¡>»nT_U7 Q┐uàÆ━□≡í
└□`E0□L□/□)X

é!0□já□Ü □úαE`U≡b░□x
x)X□¼□╓□δé⌡└·α□αU`□░!x5╪□╝□l□6□»□o□█Çφ└÷`□≡V░□╪□∞□v□╖ü╖â▌┴-`O░□╪□∞□
÷□∩□√ü²┴□ê7p□ⁿ□ⁿ□ⁿ

n-<□■ □%á□¬`□Φ□□9╧;}.°-8m┴i
N[p┌é╙□£╡α┤□°-8m┴i
N[p┌é╙□£╡α┤□°-8m┴i
N[p┌é╙□£╡α┤□°-8m┴i
N[p┌é╙□£╡α┤□°-8m┴i
N[p┌é╙□£╡α┤□°-8m┴i
N[p┌é╙□£╡α┤□°-8m┴i
N[p┌é╙□£╡α┤□°-8m┴i
N[p┌é╙□£╡α┤□°-8m┴i
N[p┌é╙□£╡α┤□°-8m┴iδ╝╙τt┌å╙6£╡ß┤
°m8m├i□N█p┌å╙6£╡ß┤
°m8m├i□N█p┌å╙6£╡ß┤
°m8m├i□N█p┌å╙6£╡ß┤
°m8m├i□N█p┌å╙6£╡ß┤
°m8m├i□N█p┌å╙6£╡ß┤
°m8m├i□N█p┌å╙6£╡ß┤
°m8m├i□N█p┌å╙6£╡ß┤
°m8m├i□N█p┌å╙6£╡ß┤
°m8m├i□N█p┌å╙6£╡╧;}N°]8φ┬i□N╗p┌à╙.£vß┤
°]8φ┬i□N╗p┌à╙.£vß┤
°]8φ┬i□N╗p┌à╙.£vß┤
°]8φ┬i□N╗p┌à╙.£vß┤
°]8φ┬i□N╗p┌à╙.£vß┤
°]8φ┬i□N╗p┌à╙.£vß┤
°]8φ┬i□N╗p┌à╙.£vß┤
°]8φ┬i□N╗p┌à╙.£vß┤
°]8φ┬i□N╗p┌à╙.£vß┤
°]8φ┬i≈╝╙τt┌â╙-£÷α┤□°=8φ┴i□N{p┌â╙-£÷α┤□°=8φ┴i□N{p┌â╙-£÷α┤□°=8φ┴i□N{p┌â╙-£
÷α┤□°=8φ┴i□N{p┌â╙-£÷α┤□°=8φ┴i□N{p┌â╙-£÷α┤□°=8φ┴i□N{p┌â╙-£÷α┤□°=8φ┴i□N{p┌â
╙-£÷α┤□°=8φ┴i□N{p┌â╙-£÷α┤□°=8φ┴i□N{p┌â╙-£÷■∞N □∩∞πuN'Γ

□Aÿ□&ü□└è`2Φ□/□)□□â`□îÇ□¿â

îéò┴*αEα┼`50□¼□ªüùâ╡┴:`=░>x□x□╪□╝□l

6□»□¢ü╫â=┴□`+░5x3╪□l□╢□o□;Ç╖é□┴█└N`g░
╪□∞□▌

v□□
9èu□╜□─□Äb
G▒åú) ╓p□k8Z ─zÄb=GCáX█τ¥>ù╙6£╢ß┤
°m8m├i□N█p┌å╙6£╢ß┤
°m8m├i□N█p┌å╙6£╢ß┤
°m8m├i□N█p┌å╙6£╢ß┤
°m8m├i□N█p┌å╙6£╢ß┤
°m8m├i□N█p┌å╙6£╢ß┤
°m8m├i□N█p┌å╙6£╢ß┤
°m8m├i□N█p┌å╙6£╢ß┤
°m8m├i□N█p┌å╙6£╢ß┤
°m8m├i□N█p┌å╙6£╢ß⌠ƒ╛?²□9φ└i□N;p┌ü╙

£vα┤□°□8φ└i□N;p┌ü╙

£vα┤□°□8φ└i□N;p┌ü╙

£vα┤□°□8φ└i□N;p┌ü╙

£vα┤□°□8φ└i□N;p┌ü╙

£vα┤□°□8φ└i□N;p┌ü╙

£vα┤□°□8φ└i□N;p┌ü╙

£vα┤□°□8φ└i□N;p┌ü╙

£vα┤□°□8φ└i□N;p┌ü╙

£vα┤□°□8φ└i□N;e┐çG±{°ΓêM□B%fª╣□i□O□ÆK□Æ□d1YFV#Äσ½σ₧╡=N╛W|e≈
╫Pj+5╙jÑ□┘╨╟6UZΓφ▌▐ ·╛æ⌐Σèw]≤ à╝î¡╩$╛□╟Q<>ò╚=í,S╔≥╣╫ï┐▐å-□-Σ╜ΦEí5Γ

ô╚c$é┐@\ä┐@╘┼▀─Ωπo□Wßo□
x╦K≡╗Pëêa╩Ö□□ü├AG.╫*

σâ-X□╬□├Ty▓,W╬π╨u1
cƒ□
┴'±═□9}å£╬ÆΘ*ñCEdWñπ─T¼□ª□âqπE⌠P╘1K╓<
∩ä□S─j□ï±à≥√□2-ûΘ,□y□-└p,F)R,[T╟P,6i]□!═HK▄#▄ô

Ö$□▒.□q╫X,RiΘ6□≈K▄□-BH8'<R□□- Γ╒r"ÆqX⌠ü┼╨α▓466□□□N□génπ9│QB╢,!□sf╤□▓e
┘(!□%dúälöÉ═K□qoÆI□⌐O□æª|9┌ÆÄñ□ΘMεBk°ä°±╓╬□[$æ□M□▒æ9güó▐╓▓▐╓µ□╬Θ°~:
╛ƒÄ∩°╤∩°≤∩ ¿╗╣□ ²lZh□▀╧fåµï;║"sH$rB½└≈3q'o]÷"□oò~¢╝═≈│ml□÷│b╕^î²¼□√Y1÷│b∞
g┼╪╧èqⁿ□C·á□□Ç≤┴╟0U₧,╤ò≤─RD□µΘS`!°$╛□#°╧É╙Y2]ì4╢ƒ□╤²¼□√Y1÷│b╛ƒ┘▓

[╓╟╧µíbL□½m,╞□╨∩g╚╡Xª6÷│ΓXäπP▒▄╧èσ~Vî²¼°î□└σƒa□we╞o█s┼+¿áP%óDòjbδ"¬╒ε╨n╛}
÷åÄ≡╓─
ïπ┤□┌â╡░G□╠9□□

╬□□0□⌠@╛u┼⌠r(□t┴°┴σ`□°,ªE□÷╨Q╔

p

8□£□:`-Φüóªú▓ªú¿Θ(j:èÜÄóªú¿Θ(j:èÜ╬·ù╓░□~,<?╜ ╜0╟Åça□{¼+jπΣ%ç-
╟ÿ╟1µq□□□╧æ^I□≤ú\·}╢t;êπ─╨≡sⁿ8±|x#╣Ç□¡@□+D

uæ╟□`□J_!▄σ□

z"å□ª]çi╫╓i╫a┌uÿv□ª]çδ·╓ñ□Ä«□╕'w*ÖIµ`√ïτn─÷Å□□τ□ƒréê ═┐}à£Γ≤¼µcû≡╓&ß-
═$|"|éD┬▀åO□-ⁿCD!L╜iÖT□oZ□OΓä┐□±µ├▀
≈(o▌7ê:.å≥@□,□m╨┼TyÿΩ□µ>à£ÿ√ö£√□µ╞/□£□á
║ÿ*O╓æë▓2σ<Ö°

╤Φ·□¿□w₧□┴╙B╛□─ÿ╪-q:6÷Θ⌡▒ò°√╚~î·é□σkÑ∞«e□y½ê╗╤"⌡#═Iÿ□U□╟S╬æá□Ä□y²æ╩╚M□]p2
8Q▄□□ï□ÄtäLmÖ:2□Ñ╓C
⌡d▐□GÇóåz╚M□]p2(j¿'k¿'k¿'k¿'k¿□ï[□╬╗

εìîëîì□F╞E°Ö32Zÿ╟iüΓ▄q╞▄ç1≈±1±Oπ≡},▓

τ╤╕;□"½╡┃▒£+┼ÖX□âoO╧±

ÄÇ+ ∞╫7τ∩U τ∩U8□»┬·{□╬▀½p ▌Θ≤≈*╪Σ ¡{□jÆ ~ƒ(╜«P□àµ⌠≤∞≤┼UQ╕u╕
∩□ï≈\W┬≤□□±t□zτ¡─╡□g!╕□|□\┬τke ▌─ç2┴┴`

8-\□

□╟Ç□0_åy┐□è□≥Ök╤Wkèxk□·UF?"«>*FµG-Å,îöD□E₧ê ⁿ-▓ÿ╜╥{\
─╒¬ ·j
─;Ç0┤□Câσ╨,ñx□□╥q╜W&bbª±■█Ö┐?ö■f□╖¬≡╚□d⌐xR_ïjU
╤¬ii$Q½¡╒&□╜┘+(εI□∩·ß□□Ä□3┴┴á
Ä%èv1ràá□zα

░□Σ╫mó,╤ùσiå£√a1□5░%╥▌π[·¼äV□⌡╫CY⌡xY"vr-
Æ├{≈Ä┌H▌£ÉPtsp╩7;▒nΓªñ╛I╟/ÿVíFà⌡□╗U<ö<┴_╒┐:ÑC╩■
╟R¥.¡╘‖╥«@~É□K°±ak8'Æ□q┤F┌f=]?e╞6┼s4
µt▐≤&x╫èé╚╚ùævfw│»yùÖeµÜ·µ╜µxsÆ9ìO1╫⌠╠□së╣▄\k«7_1_3▀4╖Ü;╠}µ°µ!≤+≤[F
X□Kaⁿ0└¬▓Ω,ì╒gìXS╓£╡a□X□╓ô⌡cΘl□□╞□╪X─µPts┴f│yl>{é-
e+┘sl□█─^g[╪{l□√ê□`ç┘q÷}TìVê╥hä┤cπ┘$6ìYl.≤X [┬û│╡l={à╜╞▐d[┘

╢Å}╨

▒»╪┐Q□Mèª D·í(Z5Z=Ü□¡m□m□²?∞▌φK□
╛'≡╢Å╟±8-Å°╞q¬½╗½½╗½½ƒ¬ƒƒ╗½ƒ¬»z□»π8Ä╫δz%ê□æ □DéH□
"AB╡+i█.─u%δ║np│ABÉ□$êHÉ □DBÉ□$Σ║Y╫u□»│┌┐□≈┌·□ûK╜·µ□CÆ·~
4▀P?□╥b=X□╓ë⌡`}╪□6äì`c╪□6à=`│╪<╢ê¡`k╪□╣ëma█╪.╢Å□b╟╪
vè¥a□╪╡*»ö╫╙δΣÿ£ÉSrZεö√σ□I#╢è=┼₧c/▒╫╪

÷□;└Ä░Å╪g∞+vÄ]╔%≥□y╡□æúr\N╩Mr╗▄+gΣ¼<+oû╖╔;σ=≥>·Ç|H>"-
ôO╙°Σ3≥Y·╝|Q╛"_ôo╙7σ[≥m·«|_~(?ûƒ╙Oσg≥
·╡B¬¿T╘(Ω□ÿéPP□ZßT°□□ER╤áhQ┤+║□╝è~Iúⁿ@~$ ( , *?ù_)$è□E╡□Qá□\A*L□╗┬½`□¼"½h
V┤):□=è>┼ÇbH1ó□SL(ª□3èY┼╝bQ▒óXSl(6□[èm┼«b_q¿8V£(N□gè
┼╡R¬¼T╓(δöÿÆPRJZΘT·ò□eR┘álQ╢+╗ö╜╧~σárX9¬□WN*°òÅ$ìJë▓BY¡□■ëPΓJRiR┌ò^%údòYe
│▓M┘⌐∞Q÷)□öC╨□σÿrB9Ñ£Q╬*τòï╧□σÜrC╣⌐▄Rn+wò√╨Cσ▒≥Dy¬<S^(»q)^ë╫αu8å□8à╙╕□≈π□
<ë7α-x;▐à≈Γ²° >îÅΓπ°$>ì?┬τ≡□|　　　□é»π╧≡□ÆF▄ä█q/╬α,₧┼¢±6╝□∩┴√≡□|□┴╟≡
　　|□ƒ┴g±y|□_┴╫≡
|□▀┬╥±]|?─Å±□ⁿ□?├/≡kòTU⌐¬Q╒⌐0□íóT┤╨⌐≥½"¬ñ¬A╒ójWu⌐zU²¬A╒░jT5«ÜTM½-⌐µT
¬%╒□╒║ΩÖΩàΩòΩìjO⌡N⌡^⌡A⌡I╥¿jSu¬zT}¬□╒ÉjD5ªÜPM⌐fT│¬y╒ójE╡ª┌Pm¬╢T█¬]╒╛ΩPu¼:Q
¥¬╬T□¬kBJT□5D□ü□□A□4ß$ⁿDäH□
D
╤Nt□╜D?1H

□ú─81IL□Åê9büX"₧□δ─3Γ□±èxC∞□∩ê≈─□Γ□±à°F\□7Ωruò║V╥Hî□□─□1C╞□≤─"▒B¼□□─&▒El□
╗─>qH□□'─)qF\□╫j⌐║R]ú«ScjBM⌐i╡SφWG╘Iuâ║E▌«εR≈¬√╒âΩa⌡¿z\=⌐₧V?R╧⌐□╘KΩ'Ωu⌡3⌡
⌡+⌡□⌡₧·¥·╝·â·ô·ï·¢·R}ú)╫Tij5⌡□àFú1h¼□╖&(iT»¿╫╘□ΩM⌡ûz[╜½▌W-
¬Å╒'ΩS⌡Ö·B}¡æj*55Ü:
ª!4öå╓85~MDô╘4hZ4φÜ.M»ª 3¿□╓îj╞5ôÜi=#=£fA│ñyóY╫<╚╝╨╝╥╛╤∞i▐i▐k>h>i╛h╛i.57┌
rmò╢V[»Uh5Zâ╓¬ukâ┌ÿ6¡m╥╢j;┤▌┌[ÆF═╛µPs¼9╤£j╬4□Ük¡T[⌐¡╤╓i1-
íÑ┤┤╓⌐⌡k#┌ñ╢A█óm╫vi{╡²┌Aφ░vT;«¥╘Nkiτ┤
┌%φ□φ║÷Ö÷à÷ò÷ìvO√N√^√A√I√E√M{⌐╜!╤╓*▓û¼'□ñå4ÉV╥M□╓□Ö&¢╚V▓â∞&oæ╖╓;Σ]≥-yƒ|@>
ö4j»I)YI╓Éu$F□$E╥ñô⌠ô□2I6É-d;┘E÷Æ²Σ 9LÄÆπΣ$9M>"τ╚□rë|B«ô╧╚□Σ+≥
╣G╛#▀ô╚OΣ□≥□yI▐Φ╧uU║Z]╜Ní╚Φ

:½╫¡
Ωb║┤«I╫¬δ╨uδnΘnδεΦεΩεΘεδ-Φ-Ω8▌c□»[╓¡Ω₧Ω₧K□uöÄ╓9u~]Dù╘5ΦZtφ║.]»«_7¿□╓ìΩ╞uô
║i▌#▌£nA╖ñ{ó[╫=╨╨╥╜╤φΘ▐Θ▐δ>Φ>Θ╛Φ╛Θ.u7T9UE╒R⌡öé╥P□╩J╣⌐ □ú╥T□╒JuP▌╘─
Ω6uç║K▌úεS□¿ç□G=ªxjÖZÑ₧R╧⌐ù╘kjçzK□PG╘GI#╒B╡S]T/╒O
R├╘(5NMR╙╘#jÄZáû¿'╘:⌡îzA╝ó▐P{╘;Ω=⌡ü·D}í╛Qù╘ì╛_Ñ»╒╫δ□zì▐á╖Ω▌·á>ªOδ¢⌠¡·

}╥■û■╢■Ä■«■₧■╛■ü■í₧╙?╓≤·e²¬■⌐■╢■Ñ■╡~G V□á?╥□╘□╓□╒ƒδ»

□CàíZ╥¿╒Åδ'⌡╙·G·9²é~I D┐«□ª□í□Ñ□ú▀╙┐╙┐╫□╨□╥□╤□╙_Ωo

σå*C¡í █á0h

□â╒α6□
1C┌╨dh5t□║

╥

T
W

W
≈

≈

□

□
£ß▒ü7,□V
O
╧
/
»
;å╖å□├æßúß│ß½ß▄peö□+î╒F─ê□q#i4□φF»ñ╤░dxbX7<3╝0╝2╝1∞□▐□▐□>□>□╛□╛□.
7╞rcò▒╓XoT□5Fâ╤jt□â╞ÿ1ml2╢□;î▌╞[╞█╞;╠╗╞{╞√╞□╞çF╬°╪╚□ùì½╞°╞τ╞ù╞╫╞□π[πü±╚°╤
°┘°╒xn╝2IL□ªj□bBM╕ë4ÖLvô╫─ÿXS╓╘lj3uÜz$ì╞=π;π{π□π'π□π7πÑ±╞Tn¬2╒ÜΩM□ô╞d0YMn
S╨□3ÑMMªVSç┌█t╥t█t╟t╫t╧t▀⌠└⌠╨─Ö-¢x╙▓i╒⌠╘⌠▄⌠╥⌠┌┤czk:0□Ö>Ü>¢╛Ü╬MWfë╣┬\mF╠¿□
7ôfô┘n÷Ü□3k╬Ü¢═mµNsÅ╡╧<`-2Åÿ╟╠□µ)IúΘ╥tc.7WÖk═⌡fàYc6ÿ¡f╖9hÄÖ╙µ&s½╣╞▄m╛e╛m╛
c╛k╛g╛o~`~hµ╠Å═╝y┘╝j~j~n~i~m▐1┐5ÿÅ╠─
═ƒ═_═τµ+ZBW╨╒4Bú4Nô┤ë╢╙^ÜíY:K7╙mt'▌C≈╤□⌠□=BÅ╤□⌠□=C╧╥≤⌠"╝B»╤□ÆFZC□h+φªâtîN
╙Mt+▌Aw╙╖Φ█⌠□·.}Å╛O?á╥□²ÿµΘezò~J?°_╥»Θ□·─}@╤Θ╧⌠W·£╛▓H,□ûjï≡í╧é[Hï╓b╖x-
îà╡d-═û6K°Ñ╟╥g□░

YF,cû ╦öe╞2kÖ╖,ZV,kû
╦ªe╦▓m┘╡∞[

─╟ÆFKôÑ╒╥aΘ╢▄▓▄╢▄▒▄╡▄│▄╖<░<┤pû╟□ ▐▓lY╡<╡<╖╜┤╜╢∞X ▌Z

,GûÅû╧û»ûs╦òUb¡░V[à□₧V▄JZMV╗╒ke¼¼5km╢╡Y;¡=╓>δÇu╙:b□│NX°¼3╓Yδ╝u╤║b]│nX7¡[╓
mδ«u▀zh=╡║₧XO¡g╓
δ╡Mj½ö4Z∩Z∩Y∩[X-
Z9δc+o]╡«ZƒZƒ[_Z_[w¼o¡□╓#δGδgδWδ╣⌡╩&▒U╪¬m┬ç_□n#m&¢▌µ╡16╓û╡5█┌l¥╢-[ƒm└6d□▒
ì┘&lS╢□█¼m▐║h[▒¡┘6l¢╢-
█║m╫║o;┤□█Nl°╢3█àφ┌.╡W┌k∞uv╟N╪);mwJ□m╝m┘╢j{j{n{i{m█▒╜╡□╪Älmƒm_mτ╢+╗─^a»╢
─
└φ╕¥┤¢∞v╗╫╣╪Y{╓▐lo│w┌{∞}÷□√É}─>fƒ░O┘g∞│÷y√ó}┼╛f▀░o┌╖∞█÷]√╛²╨~l?▒ƒ┌╧∞□÷kç╘
QΘ¿q╘90□ßá□┤├Θ≡;"Äñú┴╤ΓhwtI□φ;÷╖÷□√æ²ú²│²½²▄~σÉ8*□╒

—ü:p□Θ09∞

»âq░Ä¼ú┘╤µΦt⌠8·□□Ä!╟êc╟1ßÿr╟8f□≤ÄE║èc═▒ß╪tl9╢□╗Ä}╟íπ╪qΓ8u£9.□╫N⌐│╥Yπ¼sbN┬
I9i°╙ΘwF£Igâ│┼┘ε∞r÷:√¥â╬aτ¿s▄9)it£;«£□gà│┌ë8Q'ε$¥&°▌Θu2N╓Öu6;█£¥╬-gƒs└9Σ□
qÄ9'£S╬□τ¼s ▐╢Φ\q«97£¢╬-
τ╢s╫╣∩<t-;O£°╬3τà≤┌%uU║j\u.╟E╕(□φr║ⁿ«ê+Θjp╡╕┌]]«^W┐k╨5∞□uì╗&]╙«G«9╫ék╔⌡─╡
.itß.╥er┘]^□πb]YW│½═┌ΘΩq⌡╣□\C«□╫ÿk┬5σÜq═║µ]ï«□╫Ük├╡Θ┌rm╗v]√«C╫▒δ─uΩ:s]╕«▌
RwÑ╗╞]τ╞▄ä¢r╙n°█∩Ä╕ôε□wï╗▌▌σεu≈╗□▌├εQ≈╕{╥=φ~ΣPtss/╕ù▄O▄δεgε□εWε7ε=≈;≈{Iú;δn
v╗╣;▌=ε>≈Ç{╚=Γ-sO╕°▄3εY≈╝{╤╝Γ^so╕7▌[εm≈«{▀}Φ>vƒ╕O▌gε
≈╡GΩ⌐⌠╘xΩ<ÿç≡P-┌π⌠°=□O╥╙αi±┤{║<╝Pts~╧ág╪3Ω□≈Lzª=Å<sPts□╧Æτëg▌≤╟≤┬≤╧≤╞│τyτy∩·α
·Σ·Γ·µ╣⌠▄x╤%iPts!╧êg╟3ßÖ≥╟xf=≤PtsE╧èg═⌡ß┘⌠ly╢=╗Pts}╧íτ╪sΓ9⌡£y.<╫^⌐╗╥[π¡≤b^┬Kyi»
╙δ≈F╝Ioâ╗┼█εφ≥÷z√╝â▐a∩¿w▄;Θ¥÷>≥╬y□╝K▐'▐u∩3∩
∩+∩□∩Pts≈¥≈╝≈â≈ô≈ï≈¢≈╥{π+≈U·j}⌡>àOπ3°¼ÆF∩╝w╤╗Γ]≤nx7╝[▐m∩«w▀{Φ=÷PtsxO╝g▐
∩╡OΩ½⌠╒°Ω|ÿÅ≡Q>┌τ⌠·}□_╥╫αk±╡√║|╝┘~▀áo╪7Ω□≈M·ª}Å|s┘□▀Æ∩ëo▌≈╟≈┬≈╧≈╞╗τ{τ{∩√α
√Σ√Γ√µ╗⌠▌°╤²U■Z□╝_ß╫°
~½▀φ□·c▀┤┐╔▀Ω∩É4·╢}╗┘}▀í∩╪wΓ;⌡¥·.|╫~⌐┐╥_π»≤c~┬O·i┐╙∩≈GⁿI□â┐┼▀ε∩≥≈·√²â■a ¿
□▄?Θƒ÷?≥╧·□ⁿK■'■u 3
+ □ Pts ¥ ╝ â ô ï ¢ ╥□□(□T□j□⌡□E@□0□¼□w
□ê□╥üª@ká#╨□╕□╕□╕□╕□╕□╕/i⌠ƒ·/ⁿ╫□iá2P□¿
`□"@□ΦÇ3α□D□╔@Cá%╨-Φ□⌠□·□âüß└h`<0□ÿ

<□╠□□□Kü'ü⌡└|└ï└½└¢└^α]α}αCαSαKα[α2p□,□V□kâ⌡AEP□4□¡Aw0□î□╙┴ª`k░#╪□╝□╝□╝□╝
□╝□╝|□|□ΣéÅâ|p9╕*i

bA"H□Θá3Φ□Fé╔`C░%╪−∞□÷□√ââ┴ßαhp<8□£

>□╬□□éK┴'┴⌡α│αïα½α¢α^≡]≡}≡C≡S≡K≡[≡2x□*□UàjC⌡!EH□2ä¼!w(□èà╥íªPk¿#╘□║□║□║□║
□║□║z□z□ΓBÅC|h9┤□z□z-z□z□┌ ╜
□H□C╔PC¿%╘-Ω□⌡å·Câíß╨hh<4□Ü

=□═à□BKí'í⌡╨│╨ï╨½╨¢╨^Φ]Φ}ΦCΦSΦKΦ[Φ2t├ö3UL-S╧(□
c`¼î¢
21&═41¡L□╙═▄bn3wÿ╗╠=µ>≤Çy╙p╠cåġûÖUµ)≤£y╔╝fvÿ╗╠□s─|d>3_Ösµ*,æ42â╠03╩
î3ô╠4≤êÖc□ÿ%µ
│╬<c^0»ÿ7╠-≤Äy╧|`>1_ÿo╠%s□.□Wàk├⌡aEX□6ä¡aw8□Äà╙ßªpk┐#▄□╛□╛□╛□╛□╛□╛-
~□~□µ┬Å├|x9╝□~□~-~□~□▐ ┐
äÅ┬├ƒ├_├τß½ê$R□⌐Ä □4éG╚êI╥□₧
/äù┬O┬δßgß□ßWß7ß╜≡╗≡√≡ç≡°≡ù≡╗≡e°&R-⌐è╘FΩ#èê&bêX#εH0□ïñ#Mæ╓HGñ;r+r;r'r7r/r
?≥
≥0┬E-G°╙rd5≥4≥<≥2≥:▓□y□9ê□E>F>G╛F╬#WQI┤"Z□Eóh□ÅÆQS╘-│FÖ(□═F¢úmÆ╞╙½╙¢╙^Σ]Σ
}ΣCΣSΣKΣ[Σ2r□-ÅVEkú⌡QET□5D¡Qw4□iE╙╤ªhk┤#┌□╜□╜□╜□╜□╜□╜-
}□}□σóÅú|t9║□}□}-}□}□▌ë╛ì-DÅóúƒú_úτ╤½ÿ$V□½Ä!14å╟╙ÿ)fÅycLîìec═▒╢Xg¼'╓□□ê
┼FbcÆ╞ΦùΦ╗Φe⌠&V-½è╒╞Ωcèÿ&fêYcεX0□ïÑcM▒╓XG¼;v+v;v'v7v/v?÷
÷0╞┼-╟°╪rl5÷4÷<÷2÷:┨□{□;ê□┼>╞>╟╛ ╟╬cWqI╝"^□GΓh□ÅôqS▄-≈╞Ö8□╧╞¢πm±╬xO╝/>□-
èÅ─╟Γ□±⌐°L|6>_î»H□π⌡qE\□7─¡qw<□Å┼╙±ªxk╝#▐□┐□┐□┐□┐□┐□┐-
□□□□τΓÅπ||9╛□□□□-□□□□▀ë┐ì─ÅΓ-
πƒπ_πτ±+V┬V░╒,┬ó,╬Æ¼ë┤│^ûaY6╤6│ml'█├÷▒□∞□;┬Ä▒□∞□;├╬▓≤∞"╗┬«▒□∞&╗┼n│╗∞╛ñæì▒
i╢ëme;╪n÷□{¢╜├▐e∩▒≈┘□∞Cûc│<╗╟«▓O┘τ∞K÷5╗├╛e□╪#÷#√Ö²╧₧│W Ió"Q¥@□h□OÉ
S┬₧≡&ÿ□¢╙&Ü□më╬DOó/1É□Jî$╞□□ë⌐─Lb61ƒXL¼$╓□□ë═─Vb;▒¢╪O□&Ä□'ë╙─YΓ"q-
iL▄N▄I▄M▄K▄O<H<Lpë╟ >▒£XM<M<O╝L╝N∞$▐&

□GëÅë╧ë»ë≤─URÆ¼HV'æ$Ü─ôd╥ö┤'╜I&╔&│╔µd[▓3┘ô∞K

$çÆ#╓░ΣDr*9ô£M╬'□ô+╔╡ΣFr3╣ò▄Nε&≈ôç╓πΣI≥4yû╝H^°ñ⌐╨TM¬.àÑê□%iLr╓╟I>╣£\M>M>O
╛L╛Nε$▀&□ÆG╓Å╔╧╓»╓≤ΣUJÆ¬HU°É□Ü┬Sd╨ö▓°╝)&┼ª▓⌐µT[¬3╒ôΩK
ñåR#⌐░╘Dj*5ôÜM═°□S+⌐╡╘Fj3╡ò┌Nφª÷Sç⌐π╘IΩ4uû║H]°ÑΘ╨tM║.ìÑë4òª╚╬┤?□I'╙
Θ□IcΩeΩuj'⌡6uÉ:J}L}N}M¥°«╥ÆtE║:ìñ╤4₧&╙ª┤=φM3i6¥M7°█╥¥Θ₧t_z
=ö-IÅÑ'╥SΘÖ⌠lz>╝ÿ^I»Ñ7╥¢Θ¡⌠vz7╝ƒ>L°O╥°Θ│⌠E·:#═Tfj2u□,Cd¿

¥qfⁿÖH&Öi╚┤d┌3]Ö▐L□f03£□ò4ª?°┐ª╧╙W□Iª"S¥A2h□╧É□S╞₧±fÿ

¢╓fÜ3mÖ╬LOª/3É□╩îd╞2□Ö⌐╠Lf63ƒY╠¼d╓2□Ö═╠Vf;│¢┘╧□fÄ3'Ö╙╠Yµ"s¥òf+│5┘║,û%▓Tû╬
:│■l$¢╠6d[▓φ┘«lo╢?;ÿ□╬Äf╟│ô┘Θ∞ú∞\v!╗äTö╢GΣWÑφ□⌐,mÅ╙□Ñφ□⌐*mÅHui{D~,mÅHMi{D
~*mÅ╚╧Ñφ□)²o>□⌐+mÅ╙oK█#≥╗╥÷êáÑφ□æò╢║╓√L□

Qû╢Gñ⌠╓e□Qò╢Gä(mÅê║┤=" ┌╥÷êÉÑφ▒⌠-□äB,Ñφ□)╜²╪è╪J█#Rz√▒□)╜²╪ü╕J█cΘ□)Ñw°ö╢║╥{
W`yò"~$T┌-æ╥□¿□ñ⌠εΘ╥□+EóHª┤="┘╥÷ê4û╢Gñ┌┤="═Ñφ□·}i{DZK█#≥ç╥÷ê┤ò╢GΣÅÑφ□i/mÅ
╙ƒJ█#╥Q┌-æ╥{ô îtû╢GΣK█#╥U┌-æ□*mÅÑw╢ö▐ΘR┌-K∩uAzÉ╥╗Ö □Θ-
mÅ▓╥╥÷(√Ki{ö]ò╢G┘_K█ú∞║┤=╧nJ█ú∞oÑφ□ôö╢G¼¼┤=b╥╥÷ê}W┌-▒≥╥÷ê}_┌-┼□┼□┼□┼□┼□
┼□┼□┼□┼□┼□┼□┼□┼□┼□┼□┼□┼□┼□┼□┼□┼□┼□┼□┼□┼□┼□┼□┼□┼□┼□┼□┼□┼□┼□┼□┼□┼□┼□┼□┼□┼□┼□┼□┼
□┼□┼□┼□┼□┼□┼□┼□┼□┼□┼□┼□┼□┼□┼□┼□┼□┼□┼□┼□┼□┼□┼□┼□┼□┼□┼□┼□┼□ }4(▐5□∩□êw
─╗□Γ]□±«üx╫@╜k ▐5□∩□êw
─╗□Γ]□±«üx╫@╜k ▐5□∩□êw
─╗□Γ]□±«üx╫@╜k≡ τ]□T*æóR⌠;!┐C╦à,G┐□≥{┤B╙□┤R╙J⌠□!□@½ä¼B□-Σ»╤-
àⁿ□¡□▓□¡□▓□²Y╙ƒ╤▀□·□┤N╙:⌠╖B■□¡□▓-ò)C1!1T.ñ□U□⌐@òB*QòÉ*ö□Æ@5BjPúÉF╘$ñ5
iFi!i╘"ñ□╡ iCφB┌QçÉ

╘%ñ

u

ΘF}B·P┐É~4(d□

□B#BF╨¿ÉQö□ÆE□B&╨öÉ)4-d□$^2$;!

m□▓□m□▓□m□▓ ²{! -m□▓□²╜É┐G[älA A╙□@[àlE Σ╨6!█╨?□·G┤]╙v⌠OB■
φ□▓□²│É□╞ägïU`┐□≥Wÿ≡ä▒JLx┬╪□ÿ≡ä▒*¼Z╙jLx┬╪Åÿ≡ä▒□∞'!□┬äτî╒b┬s╞~╞□!□Lx
┌╪○─□┼□┼□┼□┼□┼□┼□┼□┼□┼□┼□┼□┼□┼□┼□┼□┼□┼□┼□┼□┼□┼□┼□┼□┼□┼□┼□┼□┼□┼□┼□┼□┼□┼□┼□┼□
┼□┼□┼□┼□┼□┼□┼□┼□┼□┼□┼□┼□┼□┼□┼□┼□┼□┼□┼□┼□┼□┼□┼□┼□┼□┼□┼□┼□┼□┼□┼□┼□┼□┼□┼□┼□ }4(æ
JH╔OΦ□Bù╤ î«ⁿ█╖½Φ□║Ä>-]#(]%□╛b□²ÅΦ□·□]@ï(Å.ó □²»Φ□C ;║ü■O⌠╡≡□□□╣ÉeΦ┐└≈à∩
□╓#⌠_ φG▀í╟Φ▀dBô□JR+√Q÷ô¼V÷│

æ²F÷[┘∩d¿L.S╙T2╡L##e:ßδk╤□A □·┐╤ â■_⌠/Φ_╤□Eode2⌐∞{┘»dò▓*Ö IéJ~É╒╙Ωd⌡2Ö

ô)e╕îÉià √□zå^í╫┬∩'æ}'+ùU╚~□~v^╥Xåöíex□Yf*│ùy╦ÿ2╣,[╓\╓V╓Y╓S╓W6P6T6R6V6Q6U
6S6[6_╢X╢R╢V╢Q╢Y╢U╢]╢[╢_vXv\vRvZvVvQv-
òJ+Ñ5╥:)&%ñööû:Ñ~iDÜö6H[ñφ╥.i»┤_:(□ûÄJ╟Ñô╥iΘ#Θ£tA║$}"]ù>ô╛É╛Æ╛æε □ô■
□HòDéT#?I╛ç¢?╘╡Å_#┐C0╔Åê□QK~åδ□⌡□àP□□▒

ë

q!-

79╘pìC
w8tpüC□╖7îpu├î┤"╜□ï∞Rv-ë╙n░∩%ë╥E□⌠□ö.Æ@VB■□Y□Y
·#d
ΣOÉ?C"ÉuÉ┐àⁿ□$□)â─ òÉ8ñ□ÆÇTCj!IH□╥□ià┤A┌!□É.H7ñ□╥□Θç

A2É□╚(d□2
┘□┘□┘

·{╚V╚?@╢A■□▓□≥OÉ□É□å∞äⁿG╚.╚□é∞åⁿ□É=É

┘[J┘%Σ_ » □y
y□·╖Rb‾□╚2H)ΣwÉσÉ■
)Ü■%╙□ÿµ└4□ª90═üi

Ls`Ü□╙□ÿµ└4□ª90═üi

Ls`Ü□╙□ÿµ└4□ª90═üi

Ls`Ü□╙□ÿµ└4□ª90═üi

Ls`Ü□╙□ÿµ└4□ª90═üi

Ls`Ü□╙□ÿµ└4□ª90═üi

Ls`Ü□╙□ÿµ└4□ª90═üi

Ls`Ü□╙□ÿµ└4□ª90═üi

Ls`Ü□╙□ÿµ└4□ª90═üi

Ls`Ü□╙□ÿµ└4'Ü■E╙y0¥□╙y0¥□╙y0¥□╙y0¥□╙y0¥□╙y0¥□╙y0¥□╙y0¥□╙y0¥□╙y0¥□╙y0¥□╙y0
¥□╙y0¥□╙y0¥□╙y0¥□╙y0¥□╙y0¥□╙y0¥□╙y0¥□╙y0¥□╙y0¥□╙y0¥□╙y0¥□╙y0¥□╙y0¥□╙y0¥□╙
y0¥□╙y0¥□╙y0¥□╙y0¥□╙y0¥□╙y0¥□╙y0¥□╙y0¥□╙y0¥□╙y0¥□╙y0¥□╙y╤⌠/Ü╬üΘ□ÿ╬üΘ□ÿ╬üΘ
□ÿ╬üΘ□ÿ╬üΘ□ÿ╬üΘ□ÿ╬üΘ□ÿ╬üΘ□ÿ╬üΘ□ÿ╬üΘ□ÿ╬üΘ□ÿ╬üΘ□ÿ╬üΘ□ÿ╬üΘ□ÿ╬üΘ□ÿ╬üΘ□ÿ╬üΘ□ÿ╬
üΘ□ÿ╬üΘ□ÿ╬üΘ□ÿ╬üΘ□ÿ╬üΘ□ÿ╬üΘ□ÿ╬üΘ□ÿ╬üΘ□ÿ╬üΘ□ÿ╬üΘ□ÿ╬üΘ□ÿ╬üΘ□ÿ╬üΘ□ÿ╬üΘ□ÿ╬üΘ□
ÿ╬üΘ□ÿ╬üΘ□ÿ╬üΘ□ÿ╬üΘ□ÿ╬üΘ□ÿ╬ëª□╤t□L□└t□L□└t□L□└t□L□└t□L□└t□L□└t□L□└t□L□└t□
L□└t□L□└t□L□└t□L□└t□L□└t□L□└t□L□└t□L□└t□L□└t□L□└t□L□└t□L□└t□L□└t□L□└
t□L□└t□L□└t□L□└t□L□└t□L□└t□L□└t□L□└t□L□└t□L□└t□L□└t□L□└t□L□└t□L□└t□L□└tA4
²ïªï`║□ªï`║□ªï`║□ªï`║□ªï`║□ªï`║□ªï`║□ªï`║□ªï`║□ªï`║□ªï`║□ªï`║□ªï`║□ªï`║□ªï`║□ª
ï`║□ªï`║□ªï`║□ªï`║□ªï`║□ªï`║□ªï`║□ªï`║□ªï`║□ªï`║□ªï`║□ªï`║□ªï`║□ªï`║□ªï`║
□ªï`║□ªï`║□ªï`║□ªï`║□ªï`║□ªï`║□ªï`║□ªï`║□ªïóΘ_4=âi-L≤`Ü□╙<ÿµ┴4□ªy0=âi-L≤`
Ü□╙<ÿµ┴4□ªy0=âi-L≤`Ü□╙<ÿµ┴4□ªy0=âi-L≤`Ü□╙<ÿµ┴4□ªy0=âi-L≤`Ü□╙<ÿµ┴4□ªy0=âi-
L≤`Ü□╙<ÿµ┴4□ªy0=âi-L≤`Ü□╙<ÿµ┴4□ªy0=âi-L≤`Ü□╙<ÿµ┴4□ªy0=âi-L≤`Ü□╙<ÿµ┴4□ªy0=
âi-L≤éΘvëA╥'╣)¢òZÑ▛u□wQ■≡{Ω√ù Å╜│ìàφ┴≤ⁿ·SJ²╡╤║Z╗°N¥z:⌡ⁿⁿ\uΩΘ╘┌¬S5□k¼1╞hc
î□mî1F□cî1Zk¡╡╔┌h¡╡v┤╔╔Zk¡╡╔êêêêêêêêêêêX□□□æ□▒╔ÜU┐₧∞╦}╗○Ω═τ₧ê{%╝▀√½□▀□┼─
ß>ñ÷≡─╜ò/
╛╜Iδ°J÷W□╥cΘ□┐╙Ææ1≤UΩ½'Ö
_π~mé∩Γ|╜µδ∩Y#▀0}c'╗"√·╙□□ ]vⁿ=X□x□V□Æ≈i≤╙╬°²OGƒN?]|║■t≈Θ±╙≤°74■;φ

4
┼P)¬B
¿
%Q□e╨\┤□-A╥╥*┤□m@¢╤6┤□φA√╤!t□¥@°╤9t□]A╫╤-t□=@Å╤3⌠□╜A∩╤'⌠□}□p□iéLA╢□□□-
_■$p□╝□Z□□Σ
è□Ñé□A╡áN╨(h□┤#yΦ□·ê>úoé$□□~ ë ñ□ò└ ░
H□%`□╣éBAëá\P%¿□4□Ü□méNAÅá_0$□□L□ª□séE┴è`]░%╪□□□Ä□géK┴ìα^≡⌠± Σ□π`iX
&ûìß□üi0□µ╚╝□ì┼░|¼□+┼*░j¼

k‒Z░v¼
δ┼□░al

¢━fÉ<,
┣0) ª┳

ÿ
#1□c░\¼□+┴╨▒*¼□k└Ü▒6¼□δ┴·▒!l□¢└ª▒9l□[┴╓▒-
l□;└Ä▒3∞□╗┴ε▒'∞□{□räi┬La╢□□□Bì╨$t□╝BZ□□µ
ïäÑ┬□a╡░N╪(l□╢
╗ä╜┬□ß░pL8)£□╬
ùä½┬
ß╢pOxêΣ      )!#╠□□□Käσ┬*a¡░A╪,l□v□{ä²┬!ß¿pB8-£□.□Wäδ┬-
ß«≡@x,<□^□oä≈┬'ßï≡□ταix&₧ìπ8ükp□ε└╜8ì╟≡|╝□/┼+≡j╝

○━［≡v╝
∩╋□≡a｜

ƒ─g≡y|        _┼7≡m|□?─O≡sn□┐┼≡╧°+Æç╫Γ
x3 ▌åwΓ=x?>äÅΓ□°4>ç/Γ+°:╛à∩Γ□°1~å_Γ7°=■ä┐α∩"Ä(Mö)╩□ß"Bñ□ÖD

æWDïbó|Qæ¿TT!¬□╒ë□E-óvQù¿W4 □□ìë&E3óy╤ÆhU┤!┌□φë

E'ós╤òΦV⌠ ·,z□#b«8]ⁿq¬─b▒□╔□
ëFE□ói╤£hQ┤"Z□mëvE□óc╤ÖΦRt#║□=ë^D∩bÄ8M£)╬□πbB¼□¢─

▒WLïcΓ|qæ╕T\!«□╫ë□┼-Γvqù╕W< -□Åë'┼3Γy±ÆxU╝! ▌□∩ë□┼'Γs±ò°Vⁿ ■,~ò
□«$]≥q.%bëBóôX$.ë_□ûΣH□É<±‖xK╝+>□-
ï╧─ùΓ□±╜°Iⁿ"~ùp$iÆLI╢□ù□□ì─$qH╝□Z□ôΣKè$ÑÆ□I╡ñN╥(iæ┤K‖$╜Æ□╓░dL2)Öæ╞Kû$½Æ
╔╢dOr(9æ£K«$╖Æ□╓g╓½□ærÑΘ╥Åô-□K□R¥╘"uI²╥░4GZ -
ûûI+Ñ5╥ziô┤□╓ô▄Hε%OÆ□╔╗ö#MôfJ│Ñ╕öÉjñ&⌐CΩò╥╥ÿ4_Z$-òVH½Ñu╥Fiï┤]┌%φò

HçÑc╥IΘît^‖$]ònH╖Ñ{╥CΘë⌠\z%╜ò>H?K_eêî+Kù}╛l╙─2àL'│╙\2┐,,╦æ□╙èee▓JYì¼^╓$kò
u╙‖e}▓A┘êl\6àΣ╔2e┘2\F╙42ô╟!≤╨hYLû/+Æò╨*d╒▓:Yú¼E╓.δÆ⌡╨□d├▓1┘ñlF6/[Æ¡╨6d█▓=
┘í∞Dv.╕Æ▌╨-dfe»□BpëtéOáäÿP□:┬B╕□?□&rê□óÿ(#*ë□ó₧h"Zë

ó¢Φ#□ë□b£ÿ"fë□bÖX#6ë□b-
╓#╝□M─ê|óê(%*êjóÄh$ZêvóïΦ%□êabîÿ$fêybëX%6êmbÅ8$NêsΓè┐%-ê╧─½□æsσΘ≥Å□Q╡X«Éδ
Σ□╡Kεùçσ9≥□y▒╝L^)»æ╫╥¢Σ¡≥

y╖╝O>(æÅ╦°Σ|≥□·▓|M╛)▀æ∩╦ÅΣ°≥
·╡ⁿN■(□F≥Σ╒≥:yú╝E▐.∩Æ≈╧□Σ├≥1·ñ|F>/_Æ»╧7Σ█≥=·íⁿD~.┐Æ▀╧-
Σƒσ»□D┴Uñ+>^╙□bàBíSX□.à_□VΣ(□□┼è2EÑóFQ»hR┤*:□▌è>┼ábD1«ÿR╞*□□╦è5┼ªbG▒»8R£*
.□╫è;┼úΓY±ªLR≥ö□╨,%ªö"yè□┼░bL1⌐ÿQ╟+û□½è
┼║bOq¿8Q£+«□╖è□┼g┼½□QròΘJ╛□Uèò□ÑNiQ║ö~eXÖú,P□+╦öò╧□e╜▓I┘¬∞Pv+√öâ╧□σ╕rJ9½\
P.+╫ö¢╧□σ╛≥Hy¬╝P^+∩öÅ╧gσ¢*I┼Se¿▓TÿJ¬R⌐

*¢èTQ*FòïΣ)Wò□╧mσ₧≥Pyó<W^)oò□╜╧╧╙W□óΓ¬╥?t
UëU□òNeQ╣T~UXòú*P□½╨Tò¬□U╝¬I╒¬ΩPu½·Tâ¬□╤jJ5½ZP-
½╗T¢¬□╒╛ΩHu¬║P]½εTÅ¬g╒¢:I═Sg¿│╘ÿZ¬V⌐
j¢ÜTSjF¥½.Tù¿╤╒UΩZuâ║□╔S]⌐nU□¬╧¬W5óµ¬╙╒|5¬□½□j¥┌óv⌐²Ω░:G]á.Vù⌐+╒5Ωzuô║U▌í
εV≈⌐□╒#Ωq⌡özV╜á^V»⌐7╒;Ω}⌡æ·T}í╛V▀⌐╒╧Ω7MÆå°╔╨di0ìTú╥□46
⌐í4î&WS¿)╤ök¬4╡Ü□M│ªM╙⌐Θ╤⌠kå4úÜ $Oô«ßkPìXú╨Φ4□ìKπ╫ä59Ü□M▒ªLS⌐⌐╤╘kÜ4¡Ü

M╖ªO3¿□╤îkª4│Ü□═▓fM│⌐┘╤∞kÄ4°Ü
═╡µN≤¿y╓╝iô┤<må6KïiÑZò╓á╡iI-
Ñe┤╣┌Bmë╢\[Ñ¡╒6h¢╡m┌NmÅ╢_;ñ□╒Nh°╡s┌Eφèv]╗Ñ▌E≥┤.¡_□╓µh
┤┼┌2mÑ╢F[»m╥║j;┤▌┌>φávD;«¥╥╬j□┤╤┌5φªvG╗»=╥₧j/┤╫┌;φú÷Y√ªK╥±t□║,□ªôΩT:â╬ª#u
öÄ╤σΩ□u%║r]ò«V╫ák╓┤Θ:u=║~▌ÉnT7í¢╓═Θ□u+║u▌ûnWwá;╓¥Θ.u7║{▌□Æ°½╘╒ΦΩuM║V]ç«[╫
°□╘iΦ╞uS║Y▌énY╖ª█╘φΦ÷uG║S▌àεZw°{╘=δ▐⌠Iz₧>CƒÑ╟⌠R╝Jo╨█⌠ñ₧╥3·\}í╛D_«»╥╫Ω□⌠=·
6}°╛G▀»╥ÅΩ'⌠╙·9²ó~E┐«▀╥∩Ω□⌠╟·3²Ñ■F□»□╥┐Φ▀
□CÜ!╙Ém└æ<}ƒ~P?ó╫OΘg⌡
·e²Ü~S┐ú▀╫ΘO⌡□·k²¥■Q ¼□3$□xå

Cû□3H
*â┴`3É□╩└□r
àå□C╣í╩Pkh04□┌

¥å-C┐a╚0jÿ0L□µ

ïå□├║a╦░k80□□╬

ùå□├╜ß╓≡bx7rîi╞Lc╢□7□Fì╤dt□╝F┌□C≥

╥å5├ªa╟░o82£□.

╫å;├úß┘≡fL2≥î□╞,#fö□UFâ╤f$ìöæ1µ□
ì%╞rcò▒╔╪`l6║□;ì=╞~πÉq╘8a£6╬□□ì+╞uπûq╫x`<6₧□/ì7╞{πô±┼°nΓÿ╥LÖªl□n"L□ô╔Σ0yM
┤)f╩7□ÖJM□ªjS¥⌐□╔3^□»ìw╞Gπ│±═ödΓÖ2LY&╟$5⌐L□ô═DÜ(□c╩5□ÜJLσª*S¡⌐┴╘lj3uÜzL²ª
!╙¿i┬4mÜ3-ÜVLδª-
╙«Θ└tl:3]ÜnL≈ª'╙ïΘ▌╠1°Ö3═┘f▄Lÿ5fô┘a÷Üis╠£o.2ùÜ+╠╒µ:sú╡┼▄nε2≈Ü□╠├µ1$╧╠3gÿ│
╠ÿYjVÖ
f¢Ö4Sf╞£k.4ùÿ╦═UµZsâ╡┘▄fε4≈ÿ√═CµQ≤äy┌<g^4»ÿ╫═[µ]≤ü·╪|f╛4▀ÿ∩═Oµ□≤╗àcI│dZ▓-
╕à░h,&ï├Γ╡╨ûÿ%▀Rd)╡TX¬-uûFKïÑ▌╥eΘ╡

Xå-cûI╦îe ▓dY╡lX╢æ<ï=BZ(
c╔┤□ZJ,σû*K¡Ñ┴╥li│tZz,²û!╦¿e┬2mÖ│,ZV,δû-
╦«σ└rl9│\Zn,≈û'╦ïσ▌╨▒ªY3¡┘V▄JX5Vô╒a⌡Zik╟Üo-
▓ûZ+¼╒╓:kú╡┼┌nφ▓÷Z□¼├╓1δñu╞:o]▓«Z7¼█╓=δí⌡─zn╝▓▐Z-
É<k╣╡╧Zkm░6[█¼¥╓-k┐u╚:j¥░N[τ¼ï╓□δ║u╤║k=░-[╧¼ù╓□δ╝⌡╓·b}╖qli╢L[╡
╖□6ì=ds╪╝6┌□│σ█èlÑ╢□[╡¡╬╓hk▒╡█║l╜╢□█░m╟6i¢▒═█ûl½╢
█║m╧vh;▒¥█«l┐┤□█g█½□▒sφΘ÷Å-
ïæ<[Å¡▀6d□╡M╪ªms╢E█èm▌┤e█╡□╪Ämg╢K█ìφ▐÷d{▒╝█9÷4{ª=█Ä█
╗╞n▓;∞^;mÅ┘≤φE÷R{à╝┌^go┤╖╪█φ]÷^√Ç}╪>ƒƒ┤╧╪τφK÷U√å}█╛g?┤ƒ╪╧φW÷[√â²│²╒
ü8╕Ät╟╟Åµ

▒Cß╨9,

ù├∩□#y÷E√è}▌╛e▀╡-
╪Åφg÷K√ì²▛d□▒┐;8Ä4Gª#█ü;□ç╞ar8□^□φê9≥□EÄRGàú┌Qτht┤8┌□]Ä^╟Çc╪1µÿt╟8µ□KÄU╟
åc█▒τ8t£8╬□WÄ[╟âπ│π╒ë8╣╬t'▀ë:┼NàSτ┤8]N┐3∞╟q□8ï¥e╬Jgì│-╒s£9.□7Ä{╟ôπ┼±εΣ8╙£
Ö╬l'ε$£□°╒Θpz¥┤3µ╟w□9K¥□╬jg¥│╤┘Γlwv9{¥□╬aτÿs╥9π£w.9W¥□╪mτ₧≤╨yΓ<w^9o¥□╬╧╧╬W
□ΓΓ║╥]|□Ω□╗□.¥╦Γr╣ⁿ«░+╟Uα*vò╣*]5«zWô½╒╒ßΩv⌡╣□]#H₧ïπJse║▓]╕ïpi\&ù├σu╤«ÿ+▀U
Σ*uU╕¬]u«FWï½▌╒σΩu
╕å]c«I╫îk╠ΣZum╕╢]{«C╫ëδ▄uσ║u=╕>╗^▌ê¢δNw≤▌¿[∞V╕unï█σ÷╗├ε□wü╗╪]µ«t╫╕δ▌MεVw
ç╗█τ-tÅ╕╟SεY≈é{┘╜µ▐D≥▄&╗├φu╙εÿ;▀]Σ.uW╕½▌uεFwï╗▌▌σεu□╕ç▌cεI≈î{▐╜Σ^uo╕╗▌{
εC≈ë√▄}σ╛u?╕?╗_IäΣÆΘ$ƒDI1⌐ uñàtæ~2LµÉ□d1YFVÆ5d=┘D╢Æ□d7┘G

Æ#Σ89E╬Æ
Σ2╣FnÆ;Σ>yD₧Æ□Σ5yçΣæÑd□YM╓æìd
┘Nvæ╜Σ□9LÄæôΣ

9O.æ½Σ□╣║MεæçΣ yN^æ╖Σ□·Ö|⌡ -«'▌├≈á-▒Gß╤y,-ù╟∩
{r<□₧bOÖ°╥Sπ⌐≈4yZ=□₧nOƒg╨3Γ□≈Lyf=
₧e╧Üg╙|π┘≈□yN=□₧k╧¥τ╤≤∞y≤&yy▌

o□ÆτΘ≥⌠z□<├₧1╧ñg╞3∩Y≥¼z6<█₧=╧ίτ─sε╣≥▄z-<ƒ=»^─╥⌡ª{·^╘+÷*╝:»┼δ≥·╜aoÄ╖└[∞-
≤Vzk╝⌡▌&o½╖├█φφ≤

zG╝π ▌)∩¼w┴╗∞]≤nzw╝√ ▐#∩⌐≈┬{φ╝≤>zƒ╜○╛$-
╧ùß╤≥a>⌐Oσ3°l>╥G!y ▐y∩Æw╔╗ß▌ ÷εy□╜' ▐s∩ò≈╓√α²∞}⌡!>«/▌╟≈í>▒Oß╙·,>ù╧∩
√r|□╛b_Ö»╥Wπ½≈5·Z}□╛n_ƒo╨7Γ□≈M·f}
╛e▀Üo╙╗π█≈□·N}□╛k▀¥∩╤≈∞{≤'·y■

□û≤K²*┐┴o≤ô~╨╧°s²à■□□╡┐╧_ïΣ·N|τ╛+▀¡∩┴≈┘≈ΩGⁿ\□║ƒ∩G²b┐┬»≤[ⁿ.┐▀÷τ°
ⁿ┼■2□Ñ┐╞_∩o≥╖·;ⁿ▌■> á□─?εƒ≥╧·□ⁿ╤■5 ª□╟┐∩?≥ƒ·/ⁿ╫■; ú ┘ □H□≡□□ü¼□□É□T□C└□
□TÇ   Σ□□□%ü≥@Uá6╨□h

┤□:□=ü■└□Æ□@□▄@zÇ@□ΓÇ"á
X□«Ç?□

Σ□□□┼ü▓@eá&Ph□┤□:□▌ü╛└``$0-ÿ□╟□□□╥ü╡└f`'░-
8□£□.□╫ü╗└cα9≡F%Q<*â╨ó0JJ⌐(□eúHèó□*ù*ñJ¿r¬è¬Ñ□¿f¬ìΩñz¿~jê□Ñ&¿ijÄZñV¿u$Å╥Q
□╨E·⌐0òC□P┼T□UI╒P⌡T□╒JuP▌T5HìPπ╘□5K-P╤╘□╡IφP√╘□uJ]P╫╘□⌡H=So┴ñ
/ÿ□╟□bAiP□4□mA2H□Ö`n░0X□,□V□kâ
┴µ`[░3╪□∞□

□Gâ□┴Θα\p1╕□\□n□wâ□┴παY≡2xâΣ□ïâe┴╨`M░>╪□l
v□╗â}┴┴αHp<8□£
.□ùâk┴═αNp?x□<
^□»âw┴╟αs≡ìNóyt□¥Ec┤öV╤□┌Fô4E3t.]Hù╨σt□]K7╨═t□▌I≈╨²⌠□=JO╨╙⌠□╜H»╨δ⌠□╜K-
╨╠⌠□}I▀╨≈⌠□²B┐ç8í┤P&ÆGw╨▌t-
=HÅ╨π⌠□=K/╨╦⌠□╜I∩╨√⌠□}J_╨╫⌠□²H?╙oíñ□/ö□╧□a!iH□2äl!2DàÿPn¿0T□*□UàjC
íµP[¿3╘□Ω□
àFC□íΘ╨\h1┤□Z□màvC□íπ╨YΦ2t□║□=à^B∩aN8-£□╬

πa"¼  ¢┬Ä░□╔
═å□B╦í╡╨fh'┤:□¥å.B╫í╗╨cΦ9⌠□N□≤┬□ß¼0□ûåUaC╪□&├Tÿ τå
├%ß≥pU╕6▄□n

╖à;├=ß■≡Px4<□₧

╧à□├+ß⌡≡Vx7│□>

ƒà/├7ß√≡S°%ⁿ-ßD╥"Öæ∞□-!"Üê)Γêx#t$□╓Å□EJ#□æj$/|□>
_ä»├wß╟≡s°-Æ□ßE2"Y□,"ì¿"åê-
BF¿□□╓ì□FJ"σæ¬Hmñ!╥□iïtFz"²æí╙hd"2□Öï,FV"δæ¡╙nΣ r□9ï\Fn"≈æº╙KΣ¥ß0iL&ô═α

┴h□□п`╝

═━ÿ|ªê)e*ÿjªÄidZÿvªïΘe□É╝╙□ô━≡ÿ

îì!□èaÿ\ªÉ)a╨Ö*ªûi`ÜÖ6ªôΘa·Ö!föÖ`ªÖ9fæYa╓Ö-
fù9`ÄÖ3µÆ╣aεÖ'µàyÅrói╤╟hv□Å□QM╘□uD╝Q:□ïµGïóÑ╤èhu┤.┌□më╢G╗ó╜╤üΦpt,:□¥ë╬Gùó
½H^T□5DmQ2JEÖhn┤0Z□-ÅVEkú
╤µh[┤3┌□φÅ

EGú□╤ΘΦ\t1║□]ÅnEwú□╤πΦY⌠2z□╜Å>E_ó∩1N,-û□╥Äß1"ªëÖbÄÿ7F╟b▒ⁿXQ¼4V□½Ä╒┼□c-
▒÷XW¼76□□Äì┼&c3▒·╪Rl5╢□█Äφ┼

c'▒≤╪□ƒ□_z°⌐±Ñç╧ï/=ⁿ/πK□?-
╛⌠≡╙πK□ wΓK□?#╛⌠≡┐□_z°_ï/=|~|Θßg┼ù-■7ΓK□ S|Θßú±Ñç/ê/=|,╛⌠≡E±Ñç/Ä/=|I|ΘßKπ
K□_□_z°≥°╥├W─ù-╛*╛⌠≡ì±Ñçoè/=|s|Θß[ΓK□▀□_z°÷°╥├w─ù-╛3╛⌠≡▌±ÑçO╞ù-╛/╛⌠≡²±ÑçO
┼ù-~0╛⌠≡□7╛⌠≡sΓK□?/╛⌠≡≤πK□┐
╛⌠≡□?╛⌠≡ïΓK□ □ΓK□┐8╛⌠≡ 0╛⌠≡KΓK□ ÅΓK□┐4╛⌠≡ 8╛⌠≡╦ΓK□ ¢±Ñç_-_z°□□_z°□±Ñç °±Ñ
ç_□_z°□□_z°U±ÑG≡□_z□ 3╛⌠□^πKÅα□┼ù-┴[|Θ□╝╟ù-┴┐─ù-

ë/=╪□±Ñ□Kè/=Xr|Θ┴8±Ñ□Kë/=ë□□ &□L$ÿH0æ`"┴Déë□□ &□L$ÿH0æ`"┴Déë□□
&□L$ÿH0æ`"┴Déë□□ &□L$ÿH0æ`"┴Déë□□ &□L$ÿH0æ`"┴Déë□□ &□L$ÿH0æ`"┴Déë□□
&□L$ÿH0æ`"┴Déë□□ &□L$ÿH0æ`"┴Déë□□
&□L$°█□æ/É\ä@èæ2ñ□⌐AΩæ&ñ□Θ@║æ>d□□A╞æ)d□Y@ûæ5d□┘A÷æ#Σ□╣@«æ;Σ□yF▐┘H·é
≈E╞□Y_|$à|!h□ⁿ□·°¬□▓ⁿ□┴Σ□;□SⁿG`'≡;≡±.┴»>°]┴»?╪
╧▀âτ-α≈ü╜≡·?Çτ>°£-
┬s?< □8□ⁿ1p□°□αP£Φ Çτa°w~□□w□°3┴─□Gß·τ└1α/αππ≡·┐ⁿα□I▐Åo▌π²-ÆÄô╬Æ.ônÆεô₧Æ
^Æ▐ô9╓i╓Ö╓┘╓x2æ¼I6%;Æ╜╓tr,9?╣(╣4╣"╣:╣.╣1╣%╣=╣+╣7y
y8y,y2y&y>y)y5y#y;y/·0·$·<·*·6·!·s≥+□ßp9Θ□>□σê9□ÄÄcß╕8~Nÿô├)α□s╩8ò£□N=°ë╙
╩Θαts·8â£□╬8gè3╦Yα,s╓8¢£□╬>τês╩╣α\sε8Å£g╬[JR□/%#%+□Kæª¿R

)╢□2àJaRrS□SJR╩S¬RjS□RÜS┌R:SzR·SåRFS&RªSµR□SVR╓S╢RvS

RÄS╬R.SnRεS₧R^R ▌╣□n□7ô¢═┼╣□W├5q□\/ùµ╞╕ ·■"n)╖é[═¡π6r[╕φ■.n/wÇ;╟□πNrg╕≤■%ε*
wâ╖═▌ π-rO╕ τ■+ε-
≈ü√Ö√ÜèñrS╙S·⌐h¬8Uæ¬K╡ñ║R²⌐ß╘£╘é╘Γ╘▓╘╧╘Ü╘·╘ª╘╓╘Ä╘ε╘╛╘┴╘æ╘±╘⌐╘╛╘à╘σ╘╡╘═╘¥╘
²╘ú╘╙╘ï╘δ╘╗╘╟╘τ╘7^□Å╟╤αe±0₧ö°Γ□x6-╓úx

/ùW╚+ßò≤¬x╡╝□^3»ì╫╔δß⌡≤åxú╝
▐4oÄ╖╚[ß¡≤╢x╗╝□ ▐1∩îw╔╗ß▌ ≤₧x/╝≈/9_ª}Ö·e÷╟ ç»ⁿ▀≈□Q!)□ëFñ-
7ñ□qá_á\ñ□σí□RèjQ?≥möB┐ë|²□ ·╧╚oα¥□^□/╪'Σ5~_πìΣ°□y└/üi└tα∩□3Ç_□f□┐□Σ□┐

╟□~□ÿ
ⁿ□Dü□ □□□qá□(□JÇRá

H□σ@□P T□ì@□╨

┤□¡@□╨

t□¥@□╨
$ü–á□Φ□·ü□ □

□iα∩□sÇ┐□╟□µ□≤ü □X□ⁿ}`!≡▀□ïÇ□□,□■!░□°G└Rα-
□╥Ç▀□û□ □X□ⁿS`%≡╧ÇU└□□¼å╗3ü~□□∩Θ°3Ü

Σ□SÇ╨□□:ÇB□PΦ□·□ 4□à&á╨□□ÜÇB□Ph□□M@í (4□à&á╨□□ÜÇB□Ph□□M@í
(4□à&á╨□□ÜÇB□Ph□□M@╡@□P□4□í (4□à&á╨□□ÜÇB□Ph□□M@í
(4□à&á╨□□ÜÇB□Ph□□M@í□(t□à

á!`□□□2└(0□ä₧á╨□□zéBOPΦ □=Aí'(⌠□à₧á╨□□zéBOPΦ
□=Aí'(⌠□à₧á╨□□zéBO╨o■kΓ▀□╓□ □X□ⁿ
`=≡/ü
└┐□6□ □╪□ⁿ6░□°7└□α▀□[ü□□l□■=░□°□└

α?□;ü▀□v□┐
∞□~□╪□ⁿ>░□°□`≡ç└~αÅÇ□└□□ü?□

□ 8

ⁿ)p□°3α(≡τ└1α/Çπ└_□□√²düô└_□°Ç┐□N□□□£□■{α,≡?□τÇ □8□ⁿO└□α□□.□
p °_ü╤└ □\□■wα*≡ƒπ□hÇZ≡╕ ≡╕ ≡╕ ≡╕ ≡╕8 7≡□°/qb□≡
\

«□□W□â+ü┴ò└α⌡□â[ü┴¡└αV`p+0╕□□▄□

n□□╖□â[ü┴¡└αV`_ mK▒,α7Ç┘└O

7J┐╤ w7b┌ìXp#□▄ê□7b┴ìXp#□▄ê□7b┴ìXp#□▄ê□7b┴ìXp#□▄ê□7b┴ìXp#□▄ê□7b┴ìXp
#□▄ê□7b┴ìXp#□▄ê□7b┴ìXp#□▄ê□7b┴ìXp#□▄ê□7b┴ìXp#□▄ê□7b┴ìXp#□▄ê□7b┴ìXp#□▄ê□7b
┴ìXp#□▄ê□7b┴ìXp#□▄ê□7b┴ìXp#□▄ê□7b┴ìXp#□▄ê□7b┴ìXp#□▄ê□7b┴ìXp#□▄ê□7b┴ìXp#□▄
ê□7b┴ìXp#□▄ê□7b┴ìΓ7¥²W7b┴ìXp#□▄ê□7b┴ìXp#□▄ê□7b┴ìXp#□▄ê□7b┴ìXp#□▄ê□7b┴ìXp#
□▄ê□7b┴ìXp#□▄ê□7b┴ìXp#□▄ê□7b┴ìXp#□▄ê□7b┴ìXp#□▄ê□7b┴ìXp#□▄ê□7b┴ìXp#□▄ê□7b┴
ìXp#□▄ê□7b┴ìXp#□▄ê□7b┴ìXp#□▄ê□7b┴ìXp#□▄ê□7b┴ìXp#□▄ê□7b┴ìXp#□▄ê□7b┴ìXp#□▄ê
□7b┴ìXp#□▄ê□7b┴ìXp#□▄ê□7b┴ì~¢°╖Ç╡└?□╓□ □X□ⁿK`□≡»Çì└┐□6□┐
l□■
░□°╖└Vα▀□█Ç□□l□■□░□°Å└NαwÇ]└∩□╖ü▀□÷□┐□∞□■□╪□ⁿ!░°#α□≡╟└AαOÇC└□□

□□□□□■

8□$^{n}$ 9p

°
α8≡ù└ αo┐½ô└_□°Ç┐□N□□□£□■÷ε<╞╬│>⌠°{╬°Lb╧°⌡d╓33&8!ÿ$$n╚Γ,É
┘LÆ▓□□ªI!!N╝‾∩c{╝∩÷x▀φ±ε≤∞ï≈u∞FÑ□□╡□B(B(èPEQ[E¿□Qá□╚BÜ▄τ|g━M
║≈V⌠Ωµ□
·δ▀Yµ╟Öwyµs□µ⌡4╨HÅ╤π⌠□=IO╤╙⌠

=K╧╤≤⌠□╝H√Φ%zÖVmT┴Fò□□U░Q□□U░Q□□U░Q□□U░Q□□U░Q□□U░Q□□U░Q□□U░Q□□U░Q□□U░Q□□U
░Q□□U░Q□□U░Q□□U░□█□□U░Q□□U«┘ΦÜì▐□6□╪H`#üì□6□╪H`#üì□6□╪H`#üì□6□╪H`#üì□6□╪H
`#üì□6□╪H`#üì□6□╪H`#üì□6□╪H`#üì□6□╪H`#üì□6□╪H`#üì□6□╪H`#üì□6□╪H`#üì□6□╪H`
#üì□6□╪H`#üì□6□╪H`#üì□6□╪H`#üì□6□╪H`#üì□6□╪H`#üì□6□╪H`#üì□6□╪H`#üì□6□╪¿║Ü
ï□□ l$░æ└F□□ l$░æ└F□□ l$░æ└F□□ l$░æ└F□□ l$░æ└F□□ l$░æ└F□□
l$░æ└F□□ l$░æ└F□□ l$░æ└F□□ l$░æ└F□□ l$░æ└F□□ l$░æ└F□□
l$░æ└F□□ l$░æ└F□□ l$░æ└F□□ l$░æ└F□□ l$░æ└F□□ l$░æ└F□□
l$░æ└F□□ l$░æ└F□□ l╘┐╟'╤╓t□¥J°╤Θt□¥Ig╤┘t

φósΘ<:ƒv╙□t!]D□╙%t)]Fù╙□t%]EW╙5t-]G{Φz‖ünñ¢Φf‖ànÑ█Φv‖âεñ╖Φn‖çεÑ╜t-
▌O□╨â⌠□=LÅ╨ú┤B√╖¡ñèjj¿ÑÄz□hñ╟Φqzé₧ñ°Φizå₧ÑτΦyzü^ñ}⌠□╜L½6□╪H

╪H`#üì□6□╪H`#üì□6□╪H`#üì□6□╪H`#üì□6□╪H`#üì□6□╪H`#üì□6□╪H`#üì□6bKb#üì□6□╫l
t=F∩□□Il$▒æ─F□□Il$▒æ─F□□Il$▒æ─F□□Il$▒æ─F□□Il$▒æ─F□□Il$▒æ─F□□Il$▒æ─F□□Il$▒
æ─F□□Il$▒æ─F□□Il$▒æ─F□□Il$▒æ─F□□Il$▒æ─F□□Il$▒æ─F□□Il$▒æ─F□□Il$▒æ─F□□Il$▒æ
─F□□Il$▒æ─F□□Il$▒æ─F□□Il$▒æ─F□□Il$▒æ─F□□IlT]╟σÇì$6Æ╪Hb#ëì$6Æ╪Hb#ëì$6Æ╪Hb#
ëì$6Æ╪Hb#ëì$6Æ╪Hb#ëì$6Æ╪Hb#ëì$6Æ╪Hb#ëì$6Æ╪Hb#ëì$6Æ╪Hb#ëì$6Æ╪Hb#ëì$6Æ╪Hb#ë
ì$6Æ╪Hb#ëì$6Æ╪Hb#ëì$6Æ╪Hb#ëì$6Æ╪Hb#ëì$6Æ╪Hb#ëì$6Æ╪Hb#ëì$6Æ╪Hb#ëì$6Æ╪Hb#ëì
$6Æ╪Hb#ëì$6Ω▀πôΦd:àNÑ╙Φt:â╬ñ│Φl:çv╤╢t-¥O╗Θ□║É.óïΘ□║ö.ú╦Θ□║Æ«ó½Θ□║û«ú=t=▌@
7╥Mt3▌B╥╥mt;▌Aw╥]t7▌C≈╥^║Åε°□ΦAzê-ªGΦQZíé÷oaE55╘RG=
4╥c⌠8=AO╥S⌠4=C╧╥s⌠<╜@/╥>zë^ªU□Il$□l$▒æ─F□□Il$▒æ─F□□Il$▒æ─F□□Il$▒æ─F□□Il$▒
æ─F□□Il$▒æ─F□□Il$▒æ─F□□▒
▒æ─F□□╓k6║fú≈üì□6R╪Ha#àì□6R╪Ha#àì□6R╪Ha#àì□6R╪Ha#àì□6R╪Ha#àì□6R╪Ha#àì□6R╪
Ha#àì□6R╪Ha#àì□6R╪Ha#àì□6R╪Ha#àì□6R╪Ha#àì□6R╪Ha#àì□6R╪Ha#àì□6R╪Ha#àì□6R╪H
a#àì□6R╪Ha#àì□6R╪Ha#àì□6R╪Ha#àì□6R╪Ha#àì□6R╪Ha#àì□6¬«αj└F□□)lñ░æ┬F□□)lñ░æ
┬F□□)lñ░æ┬F□□)lñ░æ┬F□□)lñ░æ┬F□□)lñ░æ┬F□□)lñ░æ┬F□□)lñ░æ┬F□□)lñ░æ┬F□□)lñ░æ┬
F□□)lñ░æ┬F□□)lñ░æ┬F□□)lñ░æ┬F□□)lñ░æ┬F□□)lñ░æ┬F□□)lñ░æ┬F□□)lñ░æ┬F□□)lñ░æ┬F
□□)lñ░æ┬F□□)lñ░æ┬F□□⌡∩±It2¥B°╥it:¥Ag╥Yt6¥C╗Φ\:Å╬°▌t□]H□╤┼t
]Jù╤σt□]IW╤╒t
]K╫╤-║₧ná□Θ&║Öní[Θ6║¥εá;Θ.║¢εí{i/▌G≈╙□⌠
=D□╙#⌠(¡PA%φ▀╬Ü□j⌐ú₧□□Θ1z£₧á'Θ)zÜ₧ígΘ9z₧^á□i-
╜D/╙¬ì□6R□6R╪Ha#àì□6R╪Ha#àì□6R╪Ha#àì□6R╪Ha#àì□6R╪Ha#àì□6R╪Ha#àì□6R╪Ha#àì╪
z╪Ha#àì╘5□]⌡╤√└F□□ilñ▒æ╞F□□ilñ▒æ╞F□□ilñ▒æ╞F□□ilñ▒æ╞F□□ilñ▒æ╞F□□ilñ▒æ╞F□□i
lñ▒æ╞F□□ilñ▒æ╞F□□ilñ▒æ╞F□□ilñ▒æ╞F□□ilñ▒æ╞F□□ilñ▒æ╞F□□ilñ▒æ╞F□□ilñ▒æ╞F□□il
ñ▒æ╞F□□ilñ▒æ╞F□□ilñ▒æ╞F□□ilñ▒æ╞F□□ilñ▒æ╞F□□ilñ▒æ╞F□□U╫n=`#ìì46╥╪Hc#ìì46╥╪
Hc#ìì46╥╪Hc#ìì46╥╪Hc#ìì46╥╪Hc#ìì46╥╪Hc#ìì46╥╪Hc#ìì46╥╪Hc#ìì46╥╪Hc#ìì46╥╪H
c#ìì46╥╪Hc#ìì46╥╪Hc#ìì46╥╪Hc#ìì46╥╪Hc#ìì46╥╪Hc#ìì46╥╪Hc#ìì46╥╪Hc#ìì46╥╪Hc
#ìì46╥╪Hc#ìì46╥╪Hc#ìì·≈°$:ÖNíSΘ4:¥╬á3Θ,:¢╬í]t.¥Gτ╙n║Ç.ñïΦb║ä.Ñ╦Φr║é«ñ½Φj║
å«Ñδh□]O7╨ìt□▌L╖╨¡t□▌Nw╨¥t□▌M≈╨╨┤ùεú√Θ□zÉ-óçΘ□zöV¿áÆ*┌┐╡
╡╘QO□ì⌠□=NO╨ô⌠□=M╧╨│⌠□=O/╨ï┤Å^óùì╒F□□Θ□□ilñ▒æ╞F□□ilñ▒æ╞F□□ilñ▒æ╞F□□ilñ▒æ╞
F□□ilñ▒æ╞F□□ilñ▒æ╞F□□ilñ▒æ╞Fl7lñ▒æ╞F║jú G Ä»║wû╡W≈╬≥÷Ω~YAW╥U▄┐║╜║╫╓┤W≈╫Zµ
u╟=t=▌└≤72oΓ9¢Ö╖0oÑ█Φv║âεñ╗¬- =≤-^g/_█K≈╡WÅó²╟□ΦAzê√□≤ⁿ#⌐╫∞x═Ä ┐φh░ú┴Ä□;□
∞h░ú┴Ä□;□∞h░ú┴Ä□;□∞h░ú┴Ä□;□∞h░ú┴Ä□;□∞h░ú┴Ä□;□∞h░ú┴Ä□;□∞h░ú┴Ä□;□∞h░ú┴Ä□;□∞
h░ú┴Ä□;□∞h░ú┴Ä□;□∞h░ú┴Ä□;□∞h░ú┴Ä□;□∞h░ú┴Ä□;□∞h░ú┴Ä□;□∞h░ú┴Ä□;□∞h░ú┴Ä□;□∞h
░ú┴Ä□;□∞h░ú┴Ä□;□∞h░ú┴Ä□;□∞h░cu▌1□v4╪╤`Gâ□
v4╪╤`Gâ□
v4╪╤`Gâ□
v4╪╤`Gâ□
v4╪╤`Gâ□
v4╪╤`Gâ□
v4╪╤`Gâ□
v4╪╤`Gâ□
v4╪╤`Gâ□
v4╪╤`Gâ□
v4╪╤`Gâ□
v4╪╤`Gâ□
v4╪╤`Gâ□
v4╪╤`Gâ□
v4╪╤`Gâ□
v4╪╤`Gâ□
v4╪╤`Gâ□
v4╪╤`Gâ□
v4╪╤`Gâ□
v4╪▒□ÅOóôΘ□:òNú╙Θ

:ô╫ó│Θ□┌Eτ╥yt>φª
ΦB║ê.ªKΦR║î.°+ΦJ║è«ªkΦZ║Ä÷╨⌡t□▌H7╤t
▌J╖╤φt□▌Iw╤▌t□▌K{Θ>║ƒ-á□Θ!zÿ-íGià□*⌐óÜ÷osK□⌡4╨HÅ╤π⌠□=IO╤╙⌠

=K╧╤≤⌠□╜H√Φ%zÖVφh░ú□░c⌡²n+²
²▬

}¢■□}ç╛[m▓ú┴Ä□;□∞h░ú┴Ä□;□∞h░ú┴Ä□;□∞h░ú┴Ä□;□∞h░ú┴Ä□;□∞╚□├Ä□;□∞h«²≈j╫l⌠>░æ┼F□□Yld▒æ┼F□□Yld▒æ┼F□□Yld▒æ┼F□□Yld▒æ┼F□□Yld▒æ┼F□□Yld▒æ┼F□□Yld▒æ┼F□□Yld▒æ┼F□□Yld▒æ┼F□□Yld▒æ┼F□□Yld▒æ┼F□□Yld▒æ┼F□□Yld▒æ┼F□□Yld▒æ┼F□□Yld▒æ┼F□□Yld▒æ┼F□□Yld▒æ┼F□□Yld▒æ┼F□□Yld▒æ┼F□□Yld▒æ┼F╒⌡┌

╪╙b#ïì,6▓╪╙b#ïì,6▓╪╙b#ïì,6▓╪╙b#ïì,6▓╪╙b#ïì,6▓╪╙b#ïì,6▓╪╙b#ïì,6▓╪╙b#ïì,6▓╪
╙b#ïì,6▓╪╙b#ïì,6▓╪╙b#ïì,6▓╪╙b#ïì,6▓╪╙b#ïì,6▓╪╙b#ïì,6▓╪╙b#ïì,6▓╪╙b#ïì,6▓╪╙
b#ïì,6▓╪╙b#ïì,6▓╪╙b#ïì,6▓╪╙b#ïì,6▓╪╙bú■=>ëNªSΦT:ìN°3ΦL:ï╬ªsh□¥Kτ╤·┤¢.á
Θ"║ÿ.íKΘ2║£«á+Θ*║Ü«íkΘ:┌C╫╙
t#▌D7╙-t+▌F╖╙□t'▌Ew╙=t/φÑ√Φ~zÇ-ñçΦazä-Ñ□*¿ñèjjh ûw╘╙@#=FÅ╙□⌠$=EO╙3⌠,=G╧╙
⌠"φúùΦeZ╡æ┼Fv└F□□Yld▒æ┼F□□Yld▒æ┼F□□Yld▒æ┼F□□Yld▒æ┼F□□Yld▒æ┼F□□Yld▒æ┼F□□Yl
d▒□█□□Yld▒æ╜fúk6z-
╪╙a#çì□6r╪╙a#çì□6r╪╙a#çì□6r╪╙a#çì□6r╪╙a#çì□6r╪╙a#çì□6r╪╙a#çì□6r╪╙a#çì□6r╪
╙a#çì□6r╪╙a#çì□6r╪╙a#çì□6r╪╙a#çì□6r╪╙a#çì□6r╪╙a#çì□6r╪╙a#çì□6r╪╙a#çì□6r╪╙
a#çì□6r╪╙a#çì□6r╪╙a#çì□6r╪╙a#çì□6r╪╙aúΩJφ□lΣ░æ├F

□91Σ░æ├F

□91Σ░æ┣F

□91Σ░æ├F

□91Σ░æ├F

□91Σ░æ┣F

□91Σ░æ┣F

□91Σ░æ├F

□91Σ░æ┣F

□91Σ░æ┣F

□91Σ░æ├F

□91Σ░æ┣F

□91Σ░æ├F

□91Σ░æ├F

□91Σ░æ┣F

□91Σ░æ├F

□91Σ░æ├F

□91Σ░æ├F

□91Σ░æ┣F

□91Σ░æ├F

□9lΣ░Q -ƒD'╙)t*¥F°╙□t&¥Eg╙9┤ï╬Ñ≤Φ|┌M□╨àt□]Lù╨Ñt□]NW╨òt□]M╫╨╡t□φíδΘ□║ænó¢Θ
□║ònú█Θ

║ôεó╗Θ-║ù÷╥}t?=@□╥C⌠0=BÅ╥□□TRE55╘╥■φ∩iáæ-ú╟Θ
zÆ₧ó°Θ□zû₧úτΘ□zæ÷╤K⌠2¡┌╙a#7`#çì□6r╪╙a#çì□6r╪╙a#çì□6r╪╙a#çì□6r╪╙a#çì
□6r╪╙a#çì□6r╪╙a#çì□6r╪ê¡äì□6r╪╙]⌡╤5□╝□lΣ▒æ╟F-□ylΣ▒æ╟F-□ylΣ▒æ╟F-□ylΣ▒æ╟F-□
ylΣ▒æ╟F-□ylΣ▒æ╟F-□ylΣ▒æ╟F-□ylΣ▒æ╟F-□ylΣ▒æ╟F-□ylΣ▒æ╟F-□ylΣ▒æ╟F-□ylΣ▒æ╟F-□y
lΣ▒æ╟F-□ylΣ▒æ╟F-□ylΣ▒æ╟F-□ylΣ▒æ╟F-□ylΣ▒æ╟F-□ylΣ▒æ╟F-□ylΣ▒æ╟F-□ylΣ▒æ╟F-□yl
Σ▒Quì÷□6≥╪╙c#Åì<6≥╪╙c#Åì<6≥╪╙c#Åì<6≥╪╙c#Åì<6≥╪╙c#Åì<6≥╪╙c#Åì<6≥╪╙c#Åì<6≥╪
╙c#Åì<6≥╪╙c#Åì<6≥╪╙c#Åì<6≥╪╙c#Åì<6≥╪╙c#Åì<6≥╪╙c#Åì<6≥╪╙c#Åì<6≥╪╙c#Åì<6≥╪╙
c#Åì<6≥╪╙c#Åì<6≥╪╙c#Åì<6≥╪╙c#Åì<6≥╪╙c#Åì<6≥╪¿□ÅOóôΘ□:òNú╙Θ

:ô╫ó│Θ□┌Eτ╥yt>φª
ΦB║ê.ªKΦR║î.°+ΦJ║è«ªkΦZ║Ä÷╨⌡t□▌H7╤t
▌J╖╤φt□▌Iw╤▌t□▌K{Θ>║ƒ-á□Θ!zÿ-íGià□*⌐óÜ□j⌐ú²{!╨HÅ╤π⌠□=IO╤╙⌠

=K╧╤≤⌠□╜H√Φ%zÖVmΣ▒æ-
▒æ╟F-□ylΣ▒æ╟F-□ylΣ▒æ╟F-□ylΣ▒æ╟F-□ylΣ▒æ╟F-□ylΣ▒æ╟F-□ylΣ▒æ╟F-□yl─÷┴F-□ylΣ»┘
ΦÜì█□6□╪(`úÇì□6□╪(`úÇì□6□╪(`úÇì□6□╪(`úÇì□6□╪(`úÇì□6□╪(`úÇì□6□╪(`úÇì□6□╪(`
úÇì□6□╪(`úÇì□6□╪(`úÇì□6□╪(`úÇì□6□╪(`úÇì□6□╪(`úÇì□6□╪(`úÇì□6□╪(`úÇì□6□╪(`ú
Çì□6□╪(`úÇì□6□╪(`úÇì□6□╪(`úÇì□6□╪(`úÇì□6□╪¿║:ç□□□l□░Q└F□□□l□░Q└F□□□l□░Q└F
□□□l□░Q└F□□□l□░Q└F□□□l□░Q└F□□□l□░Q└F□□□l□░Q└F□□□l□░Q└F□□□l□░Q└F□□□l□░Q└F□
□□l□░Q└F□□□l□░Q└F□□□l□░Q└F□□□l□░Q└F□□□l□░Q└F□□□l□░Q└F□□□l□░Q└F□□□l□░Q└F□□
□l□░Q└F□□□l╘╕╟'╤╓t□¥J°╤Θt□¥Ig╤┘t

φósΘ<:ƒv╙□t!]D□╙%t)]Fù╙□t%]EW╙5t-]G{Φz║ünñ¢Φf║ànÑ█Φv║âεñ╗Φn║çεÑ╜t-
▌O□╨â⌠□=LÅ╨ú┤B□òTQM
╡╘QO√≈Eñ╟Φqzé₧ñ°Φizå₧ÑτΦyzü^ñ}⌠□╜L½6□╪(

╪(`úÇì□6□╪(`úÇì□6□╪(`úÇì□6□╪(`úÇì□6□╪(`úÇì□6□╪(`úÇì□6□╪(`úÇì□6b╥`úÇì□6□╫l
t=F∩□□El□▒Q─F□□El□▒Q─F□□El□▒Q─F□□El□▒Q─F□□El□▒Q─F□□El□▒Q─F□□El□▒Q─F□□El□▒
Q─F□□El□▒Q─F□□El□▒Q─F□□El□▒Q─F□□El□▒Q─F□□El□▒Q─F□□El□▒Q─F□□El□▒Q─F□□El□▒Q
─F□□El□▒Q─F□□El□▒Q─F□□El□▒Q─F□□El□▒Q─F□□ElT]ùπÇì"6è╪(búêì"6è╪(búêì"6è╪(bú
êì"6è╪(búêì"6è╪(búêì"6è╪(búêì"6è╪(búêì"6è╪(búêì"6è╪(búêì"6è╪(búêì"6è╪(búê
ì"6è╪(búêì"6è╪(búêì"6è╪(búêì"6è╪(búêì"6è╪(búêì"6è╪(búêì"6è╪(búêì"6è╪(búêì
"6è╪(búêì"6Ω▀πôΦd:àNÑ╙Φt:â╬ñ│Φl:çv╤╢t-¥O╗Θ□║É.óïΘ□║ö.ú╤Θ□║Æ«ó½Θ□║û«ú=t=▌@
7╥Mt3▌B╖╥mt;▌Aw╥]t7▌C≈╥^║Åε°□ΦAzê-ªGΦQZíéJ¬¿ªåZΩ¿°ü÷∩æc⌠8=AO╥S⌠4=C╧╥s⌠<╛@
/╥>zë^ª□Q-
6è□6è╪(búêì"6è╪(búêì"6è╪(búêì"6è╪(búêì"6è╪(búêì"6è╪(búêì"6è╪(búêì╪&╪(búêì
" Ä»6²⌐«Xδ╙₧/d╫°?y⌡₧ª{R□╡╝ÿ▀ⁿ≈╞«µ?»>│ªX}ñq┬{ƒ╫╝äτ=ƒ
jⁿe≤èª[▐√Xπ▀≥╪╪│δÜgg5ì□▐≥l≤°µK∩}Fδ7╥∩╪┌lT╤°¼⌐iF≤Ü¼ªσ╣⌠╠╒═π¢₧hⁿq≤èf⌡┐n╡▐╜╝
µ Ωyú╥π∩²I6≥=niy!╤╤□╫ òMτ¢°ñ╫╗ö╒4_i□▀<%╜≥□|t╘{-
²□»7¬ⁿ|╓⌠ç^»q□?σ∩╛÷≈₧7Ω□?∩╝▀#²£╡-π[^jy╢eB╤─÷Åñ∩8$+╢╠iÖ_▌W═
xvmmu[□n░∩Ö]ûÑ┐│¼>╤Æx╙□¥MNw┤dàBkí=+□:□╖ª·╢┬τ╙ⁿLaoÜ{
Θ∞,∞/□I≤╤B:÷
□
 öµƒ□~æ╒□~Y°i■UßjÜ□]°╫4 ª≡▐4┐]x'=∩□
YM▒X□öî]*╓ª·║ΓÉ4╫□δ╙<┤8,═
┼□╥▄XlNsK▒-═ΘùcÜ;è¥i-^╝)=7□?öµ[èI≤¡┼▐╥|{±÷4□┤°╤4▀Q╝#═w□∩L≤¿Γ┴4*-J≤ßΓß4-
)-I≤╤ÜOgàÜ'k₧╩jj₧.5eàdΓ⌠≤&□?æµ1ÑOg5Ñ'K/ªy|iJÜ°ûµñ╣½┤ ═
Kk╙╝«┤.═=ÑW╙ⁿ═╥7╙ⁿ┌⌡iï&a~8+^?r≡┤¼0x·αΘY═α□CcV□zlΦ▒¼fΦ±í▀L≤kC :═W≥□dàⁿ╞<m½
ⁿ╫·╗YaX6,φ¡a┼å□eàå[□╛ÿ╒4|⌐ßKi■r┤ù╙<«a\Ü┐╥≡gi~╢ß┘4?╫≡┤4?▀≡|Ü_hx!=_o╪£µ-
[╥╝╡▒ö╬⌐┌ñ╧Ü�together    -_H╗ul:∩è-/ñ3¼╨≥b:fè-/╡²6+┤╜▌÷n:ú│r┌⌡╓⌐ƒ╙j╨□ù-
NsU¿5σ»û'ª╣Ω╘Ü≥+φ#╙J≡ætä□0kM√φφK□■En⌡¬QΘ'm?╓~6▌□«═□aφù▐½δ▀+φ┐NsU╖5□σÄrVΦh
∩ΦH+Mgτm╒3~α□,f□æÄóƒsⁿⁿè#τ7╒cª≡.GKëππÄ#ñücúàúóâπßfÄät¼╓<ò÷iu»ìO}╣⌠rΩ─Rz Θ
s=d÷`WΩ╝╥╝╘εR≈└╛╕>-
æÅH√Γµ<╝N~K₧VÑ|d>2⌡╓<╝f~{₧Ä«ⁿÄ<□W·¿|TΩ]·]⌐≈Σi╒╤∩╤∩K╝?┐?⌡┴ⁿ┴╘Åτ-
O}8O▐0 d■╓╘G≥GR╤K²t₧Ä╝ⁿk·╫R_╧_J¥É°52fòOJ¥₧OO¥Ö╧L¥¥╧N¥ù°w¢wτΘ▌µ
≤à⌐╤≤σ⌐+≤ò⌐k≤t4µδ≤⌡⌐□≤┤&τ[≥-
⌐▐≥m⌐;≤¥⌐╗≤▌⌐{≤t■µ√≥}⌐□≥□⌐ç≥C⌐G≥t.τò╝Æ*sÖ¬s¥j≤┤RΣ>≈⌐1O{6?₧-
O=ÖfL=ôƒI=ùƒK╝É_Hφ╤√R/τùS_╓_I²F■ì╘W≤tÄΣ»σ»Ñ~+ VΩòⁿJΩ╖≤o°~' NΩw≤∩ª~/ ^Ωδ·δ
⌐▀╧┐ƒ·âⁿ□⌐oΣoñ╛Ö┐Ö·├ⁿç⌐?╨□ö·╙ⁿ°⌐ ö°⌡' E■ï╘□╬ 9⌡W·»8≥½╫·¡τ¬╛
ì
Θ│Y U}¢□¢╥▄□U▀╓╞╓4╖q%▀╬╞ßi■□╫φ╝╣±µ4≈_╖≈├ì-
N≤H«▐{'WΘ╛╗±εt╧=\ƒwtπΦ4≈_ƒ≈┴╞ç╥▄□}▐ç□Ns ⌡y?ò>□╓
\ƒ≈⌐╞°╙▄□}▐╧6~6=²╫τ}ª±
i-╤Ut_Jτl]:□╙:]Nƒ□▓Aσ⌠18□Æ>=╓Ñ╣╢\ƒ]W-Z╬╙<¼<,═
σ□╥▄XnLsS╢5═mσrÜ▐╤H≤ìσ□╙ⁿ┴≥ê4▀T╛)=7°Oíâ╨-
*▀Ü^≤╢≥¥i-U-òε ô≥╟╥|w·ε4▀S╛7=≈òG°·■≥□i~░ⁿ`Ü*□<=ƒH½─ui}°dÜ?U~$=Åû-
K≤πσ╟╙ⁿDyLÜ?]~2=OòƒM≤si%╣.¡!╤╥O┤<⌐spyE╥_m╢7)»T>ÿ|W*W»èW*W ⌐JσΩ □╝T«║¼ª|<
i½T╛É£U*_J½Pëò°ö$⌡6b·╖¼«²□«□\▐1$□╘Q╫Qù]╫Q▀╤öµµ┤µ╘

¼/C□π»□¢]≈b╫ïπ│;^∞Ü13█≡╥ó«ΘY∩ñ      │║▓╫'uMÿû²═Σ
π╗▓ƒM□q▐╟B}6<□4µæ▒#▓;>√ÖgGd□|±sÅÅ╚─ ≥╪╘g▓∞▌w│R·
▄₧▌Ü▌ùìµvM68δ╚n□ÿçd¥┘φ□s]z¡Å

╟╞┘□▓;X⌡¬╖iΘ»íYCV|⌠≤_□æ▌°┼▒Åª∩╟#â╥c├▓□fú□nU□╫▀ÿ▌9p½&}═ê∞O~≈:à∞å▀═â╥□□│¢▓╗
╥{(<⌡ò⌠□4□|»ª∞µü⌐9√P╓Ü╒<⌡╠3Of╖î²ⁿgGdw}i∞ƒÄ╚▄█└+ö▓û∞û∞c□∩╣5√pv≈└#╡Y[62╗gα╓
uΘ╫╞G▓{│°^Ü=wvß2²+·-·□·:}â■
²╗i□║f□~Rmq□²□¥I╗╤∩╤╨╥-
╚╗ΦOΘ/Θ┐╨w¬¡□Dç╨å□/vM½iÑcΦ╦t5□⌠U·□·□·v╡â°┌AcΘ-□Φ_╥╫gL¢1m╨?╨_╥╖½-
╒╥□┌Aoª╖╚╗ΘC⌠Q·4□;wε]≈ùPtsK}á4s±]ú∩-¡M╝»┤)utiGΩ²Ñ▐╘□JçS,⌐╘GJ!⌡╤╥⌐╘╟J□R-
/╜Æ·DΘ╒╘1Ñ+ï∩║ «╥wS?V·~Ω▌Ñ7S∩)²(⌡▐╥?ñ▐W·╟üsπ?╙¢╥±9*□ywÑúπεt□▄╤±□□÷@÷`÷P÷±
∞□┘╫│□│±┘K┘╦┘älb6)ÖrJ65¢ûM╧fd3│Y┘∞⌠*·┐{ò °5□ⁿπ7■╗╟ ≤∩»É═íà▀k╫{µ·≈⌠≈ƒYmk:c
Ng╒'│OeÅdÅfÅeÅgOdc▓OgOfOg□Ü}&√l÷╢∞≤iE·B6û╡á ┘□ΦY□▄ú· µ╤╧°╟»O½B
g~m:½»Ok┘w▀□╙√╔╙□╓ÉVª╞┤≥4°□Ñ5¡□σ┤fvñ╡qxZ□oL╧□æÄ¿¢╙jtKZcFª╡Σ╓┤é▐^]-
^ß┐·g*eOe_═╛û=ƒ╝Én╒

▄·?M╒Ocu□─▀╫g_╩╝£ì╤╛Æ²Y÷l÷\÷τΘ╛┴┘□ ╚╝àtdW;₧╛D ª□ΦD:ëNªSΦT:ìN°3ΦL:ï╛≈¼Φ╨~[
°ùΓ⌡Θ|╓'èƒ)~╡8╜╕┤╕%}┬:Qⁿ╥Γwïo□»╓╘╓┤╓<T≤t═┬A3K▀.╜Y·Iφ─┌M╡∩\w╟αòâw

□â┴

~u≡δâ□<°τC▓!╖

yr╚╩!ùç|{╚¢u╡u¡u#δF╫ì⌐□W7▒«╗«°«╖εj}C²ê·çΩƒ«□«~j╜¬?W Z²[⌡Wç╓

m□:rΦ°å>3t·╨╛⌠yΩ±ⁿ·$τƒµo□½-
÷╢a;åëa»4<╨≡d├⌠å╫n╙oXx├å□TπΓ╞ƒ5▌╒⌠t╙sM╦¢╢5□m:╒⌠WM▀k·Q╙╧Ü□¢G7?╫╝╜Y5_i■╟û╝σ
εûq-
□[║[zZz[\K_╦òû7Z▐j╣┌Z█┌┌:▓utδÿ╓q¡□[╗[{Z{[]k_δò╓7Z▀j╜┌V█╓┌6▓mt█ÿ╢qm□█║█z┌z
█\[_█ò╢7┌▐j╗Z«-
╗ûGûGù╟ö╟ò'û╗╦═IA«▄W╛R~úⁿV·j{m{k√╚÷╤φc┌╟╡Ol∩n∩i∩mwφ}φW┌▀h□½²jRNk╟╙Ä╤□c:╞u
L∞ΦεΦΘΦφp□}□W:▐Φx½πjgmgkτ╚╬╤¥c:╟uN∞∞ε∞Θ∞φt¥}¥W:▀Φ|½≤Ω≡┌ß¡├G

=|╠≡q├'

∩-▐3╝w╕√ƒ£¥
|
╫┌ ╫¼5{f÷╬▐Ö┘▒E□□≈HΓ-qiä*íÄk▄╥□□E□╒T╒!r"B#T□╘Q╒6UUU┼QG□mUU□UU╘e╗öTâ╕4RéPQ
≤■ff!{ó ₧≤□ƒ╧7╧<3k¡Y│╓│₧τ┘}╜ƒ7rSΣ«H□dQdY
⌐FhìΦ□¡jt⌐æZcTìI5≥j,«▒▓╞ª□╗j°k□Ö°└·/□ên╔╦`+fMπ├i□îjªf░ÑW«▓⌠τΘ□·áè□K□φö░@╗
╧▒@{└ε@{≥≡@;≤@á¥òA£J□{┴ä□╧%"|x"╨▐8□╢╙<k!µîàOzY·ïU╕/<□Θ¢□¢╜╡é
÷GÜ÷â╒0ε⌡α:ïδ
üc∞?m┌"Γ\(bY┤⌡Σçσ\□╩u▒Ñ□╓1[ç!ƒ╡G

∩à╙?□g:¥L"┘$Å╟#ï╔R▓Æ¼#¢╙V▓ï∞#~R@èH∩═ k█┴╛\Åτz-
╫{,}HΓÜ□├í╤\o▓⌠a■ⁿpG«s╕▐╩⌡]Ki╧5□~d
╫╖-φo├u□╫ⁿk²ⁿ·╤&\Å4┐║

≥U_─╢ßêV□H&ÖAµÉàΣM▓£¼&□╙f▓¥∞!□╓   RH.æRr[áéK□□"ä:B£□/$□□àn□ ≥ú≈⌠□K-
π+p|,╫|ª'□q╜öδcû■░□╫s╕▐kΘô╜²╓y\░⌠)▐■╘BK□⌠▓⌠On«y ╙I\»│⌠╧u╕╫Σ·░Ñ
·xà|╜┬2Kƒ□╟5ƒ ┘(«gp]hΘs╓\█<ⁿ▄ª@Å╛b┘n╙3¢<─δ╟VW├╕µ_]╨g]╨≈│ö»┬5ε7╫╕_\Kσz.╫;╕
>iΘδ!ò╧╙u▐≈·v«o¢mî▀W⌡P±%á║KFM╙Öw$2Ö┘µF(╫ⁿ=e±\ƒ░⌠═lK▀Γτπ□▀ì▀║r}▌╥╥·⌐,Åz╪Ö-
τú▀qq═┐■≈╤fδ╢║$ïΣÆ╣║d□╓'+╙Z▓æl!;╙^rÿ£$gI1╡║Nε□óα□|BñPOh$$□IB▓╨CΦ/

□F□πäëBûÉ+╠□□ ·┬□a¡░Q╪"∞□÷□çàô┬YíX╕.▄í"uS-
ìñ⌡Éù□ÉÖô)Å>┐s ╜╥┐@_ojè≤bj┴·B╧¼¥ñóσç╘°»håû¼¥Ñ≥fK+□,φ▓Vû□uß┌:·╚╜═╖₧┼û

╢║vé¬û▀Q-
Æk╥o⌐╫oΘ□+┬╨*K,φ│ⁿ₧V□bΘPëkδ|╨j├,□□┬╡u>ì ▀≤ªÄ□eΘΩM╕╛eΘH+R╤□╣║ûÄ▓╓ë╓⌠q]`ΘZⁿ=
╡-□íu¼sKδ╓Γ┌èΩ┤-∩_ƒ□W}+Γ╥h╛

°<c\\∩╢tl₧Ñπ° ·5Σ▀╫É?o┤ └╥ì╙,▌ñ

╫┼□τû6]pniï╥@;A┤┘æ□9É&─Wxn╪K□φû■@╗UQÑôI[oτ┌è⌐┤MTσ6m:pmE

·ê█lπ@àå*>å4π≈π╡│-═5▀╧G¼°G□]□∩ùxO≤}O╠τÜ√y[%p□┌6
ⁿ«╢□r▓iÆ□┐h;εφφ╕7╢
î₧┤]▒YIX6*ê÷Zàτå□Vß9·╖Å¿`S╪sm÷ë@╗K\á▌u□q:+|M∩□Γñ□Z▄[ô6°═╛□┐,╣≈⌠╢≥*φ├Oy_+û
╥~Θû-╙╜?╛¢Ñƒα▐¥fE2:pl@Ñä;â¼xB□°□╬l≡ {╦íⁿ□

=□╕□├Æl÷°└o□╢ÿ8Ñç∞⌠░ì\[1£>┼=δ⌐┘\-
4+╙√3z¬,p»₧no≤N▄□╙╟╥y□,²╬ñ□}□░1wWà1>n_┴╞≤O□□╝k≤n╙«░□ƒ°XzK`%I╥▄~ÿ▀}═╟·ÜG¥»
╣εêyê≈π²;╓Z·¢Ñû■n╖Ñ┐/░┤□Fα≈£

²/▓;=oeezañÑ/≥ò╛┤╙╥┐≡XZlUô⌠r=«≈Y║ä∩╘□╛□Wy¼+Ñ□½â╙W║╔╥╫·□gPàu╝ûg┌□2O^5╨δ|╡x
à@o≡ÿQ╞=°lÖÑoro┐Ö├5Å┘┐□«S╣▐H£rà┘▄Ä░┘┘µ∞$|E□j╘8□O□·□₧ì╧·Lε≡l≥;╧nwy÷╤ú h□},
╫ûù3┴╨CîI\Åµ·dÇτ1▒¢═≤ÿ8æδyfî°□»Ñ╤╧╥□╕╢b:sär▌òδL«□p╜Ækδù□ôFqm┼
&τ□╬S▐X)G0Ö┐Sµ∩T4--
Bms╡₧Fp▌âδ9\oß┌┐Tÿô»âô╧▄ÖE£ó@d≤╜□*Gδ┘□«¡*Ç╣é╣n╧╡σΘ,ê»FP¬9CWà▌δx ╖è╒"¥δ|«·
·≡zë╣□q=Ék>□╖uåÿçr▌&α□3╧H√>½|]╘±µô¬□Y`□□M╞ô

ÆCfô□d YFVæ⌡ΣS▓ì∞&|╒╒a\≤□U·:¬╓Θ`Z□╫|¡4■e┌b«wq]ji/▀U/o∩σφ╜ⁿ
╜█╡µ╕□┬█ç≡÷!╛}□-
?d╫ƒ╓√,─:±¼□ß:ÄδT«gp╜ëkε#>■m>■n▀□«ùÖ∩¼|>ÖÅÅPò╧Ü¢k½«ea|□├▌╡X╟┬ç[║V┼╨
v▌□≤|□£êz|-⌡VX║~»□╒¢a/▓┘□l÷A¢]□hG√lv+¢¥f┌□sèµsèµ√□╜⌡!m¼°╟□≡}h□S╡M□╛╓
°▐5X⌠É6δ╡µQ0F¼▄&ª

╫<

━LzH¢E\oσ·Bσ6▒<▓╞╞s=Σ!mx□Ä]═╡ !m╕□━±y┼⌡¬▄&Ä√v▄¢\∩□¿YX▄Ñ@╖yúÇ¬ü⌡_□h□∞□h□₧□
h 5?╨-:╥f

┤çù□┌O'┌∞uü÷3╫□φ╤├□φ1⌡□φgç□┌╧-      ┤ƒ»8£ì
]m÷÷@√┼Äü²_▄□hO□□Pƒ│╔3*╘ÜX▀╠
µ┘╗o =9╨╬«□Pσ▓Θ$╨₧Q□h┐Σ□┤sw┘∞█□▐Å·╠î1k▌?╢□lvF└∩
63╫f╧╡┘ïlv~à:
v^ñ═N╢┘cm÷□¢╝╔fƒ

┤gQ¢□c┃{┘∞ë6√═□òùao╖┘ù□█╧

░┘ml÷0¢¥g|╫┌∞âüδ7√Vá²▓T┴_

█e║▌□≥ⁿ□╡ò?□·#[¼l╧I□┤ □□╪□n╛=^a│╫┌∞M6{¢Ys▌□εPts@√-
□6;╒fτ█∞Γ└±τ5│┘mlvG¢▌├f°□VW≤å┘∞]üφτ7▓┘
6;╔f'█l█√τ≈╖┘â□π█ⁿæü÷ó:ü√╜xTe╖┬ ╢ª▓=╔fτ┌∞E6{╣=^o│╖┘∞â6╖╨fù□┌»S¢¡┘∞(
¢▌╙f'┘∞-6{░═-g││l÷\¢¥o│╫┌∞-
6{»═>i│ïm÷φ@{ëb│Cmv=¢□o│;╪∞^6█║-K╞┌∞L¢mⁿ╢rß$─α╫□%3q□g□B¢╤□2ì«áG╔2÷6{¢□□'è
∩Æ#ÄC╥lü╣·╣Pts□>s═□□ä▌n═▌Övr□r/ÑS<#=cΘ□PtsiPts╡tG0
VΦ┴α¢┴7Θq╡ÄZçPtsP□½ìΘÅjS╡)=⌐6W¢╙Sj
╡%-
Pô╘v⌠g╡ú┌æPtsQô╒dzVφóvíτ╘«jWZñvS╖╤≤j/╡□╜áª¿)⌠ó┌WφK/⌐²╒■⌠□5UMÑ┼jÜÜF/½□╒ü┤D□¼

ª┐¬C╘!⌠è:L□F»¬├╒ß┤T□⌐Äñ╫╘QΩ(z]□¡Äª7╘▒ΩXZªÄS╟╤¢j║ÜNo⌐π╒±⌠7ué:ü▌V'¬□i╣:I¥D∩
¿□j□²]=T3Θ]5K=ó║Ü¡f3óµ¿9LPg¿3□Us╒\╞╘<5ÅëΩlu6s¿s╘9LR_S_c▓·║·:S╘7╘7ÿS=W≤ÖK]
¬.eAΩ2u□s½╦╒σ╠ú«PW░`uÑ║Æ⌐Ω*u□╙╘╡ΩZµU╫⌐δXê║^]╧¬¿□╘
╠°nT7▓¬Ω&u□
U?U?e╒╘═Ωf□ªnQ╖░pu½║òE¿█╘¥¼║║G▌╟jké&░hìiî5╨□Üâ┼h▓&│X=⌐9Y£□ñ□▒åÜG≤░FÜ¬⌐¼▒µ
╒╝¼ëVE½┬ÜjU╡¬¼ÖVM½╞ÜkßZ8ï╫¬k╒Y
mç╢â%h╗┤]¼Ñ╢[█═Zi{┤=¼╡╢W█╦┌h√┤}∞□φÇvÇ%j□╡â¼¡vX;╠Æ4┐µgφ┤c┌1╓^;í¥`Åj'╡ô¼âVá
□░╟┤╙┌i╓Q+╘□Y'φ¼vû%kEZ□δ¼]╨.░.┌%φ□{\+╓èYW¡D+a□╤«hWX7»Φ□Yw»ΣòX□»ΓUXO»╦δb╜╝
n»¢⌡÷□{âY□>Hc}╝!▐□╓╫δ≤·X?o¿7ö⌡≈åy├╪□oä7éÑz#╜æ∞ oö7èÑyδ{δ│'╜
╝
╪@o¼7Ä
è¿-╤ü²ò□₧⌡╞┐╙ô

·ΦC%□□φkVAZqë|êt≡Ñ▀ùëÉ<╚□╚&S╬VÆH_IU□Z╚Ñ╕éö□R¡╟C%╡Z■}±W;}O┬:r□²□╔äΣD□UÉ+ûÿ
Ol□q╖║∩╛DD6é─¢Æ≥0
╦î□□9▒åƒ╦╔□RdHö∩íR'¬╟}□□5ε╛,µÆ PYn╨*«□σ  ïv5├Lë╛'V∩Ü═jv¼ÖUsS═+5o□b-
╜□yÿXú╫r╒Jα╥σü□o⌐╒├°□u╨áε╥╪£√▓0v╔}Y═e=dW∞·╕

H^┣ë
τ━«╟▀ëìö╞┸M"MIl▓

▓⌐Θ8╙äf1═┌âÿf╔═â¢ƒ5ñYL<ìo□Θ□▀?~`
□ëH╙à,NX╓▓╨ÆVbδ]ÅîKlÆx,)□Æ█nH╕eφ╢╢┐e╔ú⌠Q≈ú□□╞C▓;∞ε□╒⌐½)√Æë)ë╔πôï╕\üòÿ\╘9╬
░:╟uNΩ£□=¬╦ε.eÅÅε╛░√┬-a=ùú°┘║╙>ú¥╤¬wå╤«w^╨αö▒)□)à}åÖ2°╧□C·vΘ█┐∩╪□í╗⌠]

·┤_■~i²6⌡∩□I╨□φµ

X9`e▀σ°╗╤╕él-░c└┴╘4S▓SùÖ▓!u□╪É║'⌡pΩ-<▀±D▄□kƒ╪Éû

□₧v□φ▓±╟|≥d╖╘=On|rδαZ□]<4fDßêΓ□e╧□NÅK_ò╛τ₧N┐ô~τ·□□OO,¥▄ur▀╔
&oÿ╝e≥«╔ç3hå/úUFçîn□i□ú2▓2≥2□e,ƒ╥a╩≡)□S╢O90Ñ□G&>snµ╩┐ï□Å √╕¼Φ¼-Y3▓>═┌ôU<U
Ü:nΩ╩l1{s÷╔i╥┤Z╙Γ°
₧û>m╔┤┬£╙£I9Kr6τ∞═±τ▄ÜN°╫¢>j·ÆΘùfê3ófñ═□;#□╞ªù╙Kë/e╜┤+ùΣ╞τÄ╧══╦▌Æ{bf╠╟Q3╫╬
╝¥╫&/}û÷□QïVèL□qgV½□bDöY├-ê□K■α⌠σ█╧\αI▒|²í±τ^

¬ üQdV▌□1Γ├¼E□━è

F4
─½□Vú□□9‖s□╛─è╞åFΣ¡ûÅH{╓W□ZΦï╝-
=O√ó├:╞«6#1⌡¥}□E·*Ñ¢}╬≥VΘ╛Æ√½ç╗FT6█F□╧═√|□1.┼[7í}ë/┌□-□│+äÄ6σA₧he╦□
*dä□9í─ÿwÑ<PV)□ñ≥Φ∩7"┐□∩═qî»^Çδ│≈ba-
b╞7\□╤╓è@Vä│÷╤îï╖═][}/>▛QD╡¿|ú²â□Ä]é°f<∩é√╦k%─.⌐∞
êü╤□|σa1╢bL¡□Oy╘

3²╚èá
∩┼N#ªπ╬□c\╪Kóû'e

X□Öbe2S#kE⌡@«Jë□bⁿ+8₧yxF □□Ö≥ √X■hΣ7│}è╤□╝}æCî'╞□#ù□-ìgaú}æ≈ⁿ4j□₧╟G

1╞@ïqVδ{≤░τTcNf■╝ùAΣ╨!µ,φ9│¿R╬¼├3σ(3KÜ▀éτ=°<ΓìQ·╟ël□╡□s
╪
c□φ'≈▐è['╥X[╤cbW╫Ωé▌┴▐□δ□╡<ó╚╪y├?*z@lN-
□6·^«5≈Ü_□■`∞ïσ_╡H├ëq□□VVï╥03Q□1▓Üò╤╟£° )f-¡
ò[□┘╡ó≡,{_*≈0│δ $f■²»σ^û■#▒»ö!≈r≈-
ëÖ═ k1+î R∞½c╓%□ñ≥·Ö⌡J□1<▌┌Θ M*Åⁿτ│√∩─Zgú^±òtΩÜ289╤□mV:ÉN√î;¥║□╒ìiφK□l╓=╓
3□¬ªlúR▓εÜ╣(╓□úO≈àF5e┤F%□g╝├¼¢ΓÆI°}f=Æv┐n1$;5m└╞╘4úf1¡l^┘X╫┘¿{6□wî□╞ΦghC╟
□'┘¼å╨╓|Ümⁿ¡E╨:█¿ƒ"ç⌠□6`úYku▒□u□ε⌠□f╘Yª5g└F#□±g□Tkc═è╟Φ╒▀╝éÿuYWúé3Z□╒╪â·¼
╧£╬Iµz$□+æûl¡C°«µ╫`╛╓<√.7G6f╤▀╎╗9«φ$V┌╧è^╨$╥▓□□<°╜╓ÿ□ÆH-%qΣ1H3╥¥⌠#══□eƒH₧
$úH□□MµÆnd-YM₧'¢╓VX█ »Æ=─O□ÆcΣ□yç£□T≥/í║P¥°à(í19*⌠□zΓnoí?╣(

□□ô_àí┬PR*<%î$╫äqB:╣)ⁿMXBn
oBóä|HMa)ñû≡í░Z¿-l□÷
ui3□/┤á
┤╡╨è&╥D!æ║°Å□mi'Ü,┤ú]h□ßQ┌òv□:╨₧┤°ÉL√╨~BgÜJ╙ä«t□□$t°CΘPí□□Iƒ□z╥gΦ3B
o:åª
)t□¥$ñ╥

:K□D_ª □₧Ñ≤Θba<]B▀□2Φ□·o!ô~Lw□│Θ7╘/╝EÅ╤3┬□zü■"|B□ÑWä╧i)╜)|A□úσ┬╫TgD╪┼(c┬n
&3Å░ç⌐,D°ü·ÿO8┬BYäαg╡Y□ßGVÅ╒□N▒□,V°ë5bìàB╓ö5□╬▓µ,^8╟□X+ß<KdmàK¼□k/□│

¼âP┬:▓Ä┬»,Ö%
WXO╓[╕╧·│4ß:□╙F□┐▒q∞9╝z□¢L□,ïe╤ û═▓⌐¢-
f»S□[╟╓Qòmd□⌐╞>aƒP/√î}MC╪>vö╓`à∞□┌Çò1¥6□□b0m+·─□┌Yl'╢ú□┼ëΓ,:H£#■ç>/~*nÑoë
■ï√Θr±áxÄ╛/^□u·Ö├σp╤□nç¢-thÄ□z╚q╚q£·□'□°Θ)╟□╟□ZΦ(r□╤3Ä
Äï⌠¼π□╟□ZΣ(uö╥b╟
╟Mz┘±¢π7z┼Qε(°W□┐K

Z*╔R0-
ù4IcL□æ¬2Q□ôóÿ"┌ûZ0Uj)╡duñ╓╥π¼«╘[□└□ñ┐J3X[)W╨cOI/K»░æ╥|i>{FzUZ╙FK»I»▒╥δR
>{VZ&-c/H∩I∩▒ ╥√╥√∞Eiì⌠1¢(mÆ╢░┌╥ù╥vû+φÆv▒┘╥╖╥□÷▓tH:┬^òÄJ╟╪"Θäté-
û□ñƒ╪δ╥γΘ□{C║*▌ao╤Dª∞CYûk▒╡r┤£└v╔ër;vDε
w`╟σN≥π∞ä▄Mε┼~Æ√╚}╪Y╣┐▄ƒ¥ôSσ'Xæ<P-╨.╙#Σæ¼D-#ÅaWΣgσ

vU╬ö│Ö.τ╚╚EQ╬ô_□%y╛╝Dt╥o╨oèír╛£/Vôù╨∩êa≥{≥□1B^#□.F╨_╥▀èìσ-
ΣR1A╛.δb¬□¡Dï├ò□%N□í4QÜèúö□%A□¡<ó$ècö$Ñ¥°¼╥UΘ&>°⌠Pzêπò^Jo±□Ñƒ2@|QyRyR£ñîP
₧□'+╧+/êYJªÆ)NSª*S┼□%GÖ!NWf)/ï╣╨+╨\qû2_Ö/╛¼,T□èsö┼╨[Γ+╨□╨*±Ueì▓Fⁿ°▓NY'.RJ
òkΓk╨
σå°║rK╣%.q□° ╛ß□¥ó°ûSv╩b╛□ #╛φT¥Ü╕╘Y┼YU\µ

wåï∩9½;#┼□╬(gö╕╥╒╧5Pⁿ└5╟5Lⁿ╚5╥5R\∩□φ□#▄█⌡¼δY±c╫s«tqúk╝k╝╕╔5╔5Iⁿ─òΘ╩□?ue╣ª
ëƒ╣f╣╔è_╕╢╣vïg\G\'┼bWüδ£x▌⌡[Päx'¿n╨□GT╨┬áw□sâ6□muΣ□φ□*u╝∩û▌aÄo▌
▌¥□?║╙▄ú□eεg▌π%┘=┴=Q≥╕'╣3$═¥Θ╬ö¬╕│▄3%ƒ{╢{₧□σ^α^
╒w/t/Æó▌ï▌╦ñX≈r≈r)┴╜┬╜VjΘ■╚╝Qj∩■─²╣ö∞■┬²à⌠□≈ùε/Ñnε»▄╗Ñεε╝εâR?≈a≈aiá█∩>&
rƒp $
q ∞╛"ìt_s▀Æ&║o╗∩HÖε╗-"M⌡P□òr<óGÆª{£-Åöδ╤<í╥╦₧0Oÿ┤└□ßëö^⌡DyΩIï<╤₧hΘ-
╧4┴4)▀3▐3Sz█3█≤□i╣τU╧?ÑU₧╫<ïÑ5₧7<oH ≥╝σyKZτy█≤«⌠æτ=╧□╥╞`□□,}□□□\M┌□\=╕å┤7
°f≡mi?æäm·>╓S_╦□ÉpûFT6A?╧╢ÆXΣÜm·w░ÄÖW┼lÇ~₧□°√□í°{êÑΘçH□Yúùæ□zÖ0î╘□₧"□äß╨#
H

2Fö≡,ëB╥>h9öì╚╕%□╞9GD┤U╤6□mU┤uÖπ□ú╒U8╒□□üτì±|(₧7┼≤╞□+-c┼á≈□µ|ép╡□≤ìbY·Wl¬
■

µ█é¥╤▀egIcvÄ─│≤xvQ?╞.□ åÉ╧╢Éê╕¬ë½(╟f
F:D2êJZ□
$Æ┌ñ-□ë±ƒ□ú└ï ·)2□│·□ÿ□&â

0□98S■I■

▓└TÉ
^"ß$□╠□y`□ÿ
^□s└+`.°î─ +╟É[╕╛
t□.□ Ç□╥FΦ□·é~á?□Cz
╖H5|±PûJÆ╪ ΓfC┴8Æ╬ªßK°ô║∞%□%╛ú∩□ùüw┴A□.-□ç┴□α□G┴1p□£□?éôα□
wh·1╟i}°π□":èq}□\╤wJ

╥RèànAjK¡á╟Θ╟ñτ@:x-ⁿM?%M□X□  k#amñLÇ╡æ>"mñ⌡α□pô┤æπH5╣!□J┬σa`8x□L□S@

ÿ

░F≥□≡*x□╝KbΣ5╨ùA ╕□«éRp□`
ò□`$x□ⁿìTs□╥╞Θ#╒L▀-ü_╗╟½ï╪⌡[ñ*╝v□╝v□╝¡.╝¡;╝m□╝m ╝m(╝-
□▌╓□¡╖┴_:▓T°╨□·j°═□°═lî0æm╒▀dgαgτêï□Θ_▓ï¿┴

?;ÅVE—{ T

!I□╞è±_─°□0■ch=£Å╝□╝┌a∞e□{
/à□W□┼àQZcötîÆäQÆ°ÖhìY₧╟H²0╥½□%□#|i~Θ'µU□╞°□c|ü1bäí·fîôäq╞`£ε□g
╞Θ"î╤□b¼$aë■-
⌠ⁿ□πà`╝)ÿ┘ï□3□3¢é╤□▓B²*f╖â]└╔║□ƒ╖─O¼°┬ëmîQπ·Θ7N∞□⌠<àô╫S□□■□dE□π µ-
≈Åô7╚Kz1╔□3A-ÿ□fâù┴□≡□ÿ
÷Φσ∑;░□|□÷ü²α□°□□□ç└ap□□□°⌠╖ñ□ⁿ□NâƒA!8ú g┴9P¬-
%╫⌠ƒ╔up□öü¢αû~äⁿå3}□öâ;αwp□s╤⌡bü□┴îèElá~à
╔╦╪□ΦazÖxP/□□ü├α□≡âúα□8

NÇ-
┴Ip□\╨╦┼ïα□°□□â╦á□ⁿ□«Ç½á□\□╫□µ" ▌□:╬lê~@εáù╦¥A7╨□⌠╥□û√C□□□±|□□ó∩öçΩ┼≥00□<ï
g/@O□□q=□dÇ)░│ásá°â<\╧□╪□y>⌠□ΦW┴?q╜□╝□□â╫1■;╕┐□╫+p╜□╫-
ß·sÇ=Æ▒G2÷H╞-╔?Ωwσô□{$cÅd∞æ|□sⁿ□□□∞æ|Q?*_□┐α[è┴e²ê\□~┼╪W0÷UP□«ú-
÷N.├²¢░▒G╩□0□<ì²ó°%Σ├N▌&î╠╙Å▀╧^

Xƒ┴Ü
k*╝n□█Oî□
7□τ.□₧Θçg·ßÖ~xª₧Θçg·ßÖ~xª₧Θçg·╤║□₧V

O+ç°ò┣╙╨ßiσ≡┫rxQ1<ª

−S□Å) â╟öß }▀π }°┘ _q□₧□├⌡slä~

^πç╫°ß5~xì^πç╫°ß5~xì^πç╫°ß5~xì;Yå¥,├Nûa□²╪E?v«

╗µ╟«·▒[e╪⌐2∞ö╗Γ╟n°▒ΩσX⌡r¼z9V╜□½^ÄU-╞¬□cE╦░óeX╤2¼ó½XåU⌠c□²XE┐yb┐'2╓▓
N▓ä▄√6r∩□vÇ╘f?É□ålc«∩y╛╛?¢δ√2¼G`u┬·f□╡□ICPts⌠!O·É'}╙ô>ΣI-
≥ñ□y╥ç<ΘCPts⌠!O·≡ª╞╙ò□╙ò□8│□8│□8│□8│°pfoα╠▌└Ö╜ü3{□g÷□≥⌐è3{□g÷8╬∞q£┘π8│╪oD█T
□âsz□τ┤□τ⌠2╬i1□N□▒□`□□╔≤hMΣQr°□╣╙ç▄ΘCε⌠!w·É;}╙¥>ΣN-
r°□╣╙ç▄ΘCε⌠ß,-╟Y<Ä│x□g▒□g∩□╬\□╬\□╬▄qΣ8r£□·═çⁿµC^≤ß¼□Gn≤!╖Eα¼□G~≤┴
α □≡ □°□□ⁿ □ⁿ □ⁿ □ⁿ □≥ƒèⁿ°┬ Å├τ
α≤7α≤╟æ□}╙□>Σ?≥ƒ□;òª_6╜-▀ê│ì*m-ó≈□Σ«T╜□Q²-
<ƒì²°□Pts«ä╧╟│â╕╞⌐dGÉ╟î=<è╓°╨Ω□"⌡<=□╓□⌠=Ä╛╞▌æ<□~Å╛ì╤w-
·u!□Z«D╦⌐hYêû?íσX│╩2<g╡9╥ <∩ëτ√≡▄≡æÄ□i.Pts╛ïæb0╥

î┕╚l_lVïg╠┐e╚□*j┸ü`□x

<□╞â□└□0□╠!MëW╪f₧⌡|î╛╨x╗╣|╤└τ$ü}□□Qτ₧!]P+¬╚■>╘èß∞□⌠ETVùp∩□Tf°]Ä9~NBQYå□Ö□
²╟æ$Σ▒ü¿╗□æ□6─¼┴ÉÑ1|□╠,□3ï┴╠b0|□╠,□3ï┴╠b0|□╠

ÇW

B┼6□z(I7{ ·╨╙Ç₧>⌠⌠í°□=}ΦΘCO-
z·╨╙Ç₧±Φ·□z╞úτcfO□=U⌠T╤SEO□=U⌡T╤SEO□=U▐│;∩i╘(â░cCq«î5▐lV□╖▒ZàXß□Σ≥>á/Φ□·□
'*8'*8'*8'*8°╙°╫ñ"V8□}╞bà{ÿ⌡╕▒Gg╓a!F?#─é8╨□4□ìA□╨□4□═A<h□□@K╨□┤□m└#
□┤□Iá□h□-□□└cá#Φ□ÆAg╨□<

║é┐Çná;Φ□zé^á7xS/□▐□·`)x□,□∩éσα=░□╝□Vé□└*≡!X
╓Ç╡α_`□°□¼□ □□└╟`#°Å~
+R(|Ñƒ□╢â»┴

░□∞┬²ot┐░□|
÷Ç∩└^╘□▀â}`?╬φ@xε□²É╕S┐&ε□▀Ç▌α[░□|□÷é∩æ
÷ü²║▀ß╒
□>²îú*□□╒@□□╫╧H≤┴□zíä5ÉûΩ┼╥J²Ü⌠□X□>□½┴F▄ □z□╪ëδ□║_:ä÷¿[ñ2²î\C/öú@MP
╘╓»╔u@]P□╘□╤╚□
@

ΓV,êC╗åá9êç▌ □╧┌"█$A≈╒»)T?ú0 □□ÉÇ

□α□.□□▄└□éü□4α□!á□≡ΘàJU□□¬ü0□

"@u□  0□□≤W0□□≤Wjâ:á.¿□Ωâh╠)-uC
≡□2_"hï{□@□≡8□è≈
ç-àg╧á▌h0□î□□├□SA6ÿ□r╨v>ε┐ç÷-
á²*²ö≥!∞╒á□≈nΦg£é^Φ─╖:½Φ~'╛├YU/v╓é□e□□ ▌┬Ç□□@□2PÇ□╕@□≡□M?/xA□¿□|á*□□╒@□□□□
≡░(²▓P□╘□╡A□P□╘□⌡A4h□b□kbA□h□□ü╞á h□Üüµ -┤□  á%h□Zâ6α□É□┌é$╨

┤□Åé

└êg□A'É

:â.αq╨□ⁿ□t□▌A□╨□⌠□╝Aè~QΦ□·é~á?□Ç∩K□OÇ4≡$ÿèo╔□╙@

ÿ

fÇù@.ÿ ≥└,0□αWç░@┐─╝□□é□éEα5░□╝

▌D╟|
ΣâÑα□░

╝
ûâ≈└□≡>X >□½└ç□┘PX□╓é□üuα#░-ⁿ□l□âì`□b·W`;°□∞□;┴7`7°□∞□■ü╜z óH óH
óH
ó⌠,DΘτæ□┬□·ôÉ□┬□²ô□╡Åèêx""₧êê'"Γëêx""₧êê'"Γëêx""₧êê'"Γëδ⌠╥ΓG`=°7╪□>
□□┴□└°α3░□|

╢Ç/└V≡%╪□╛ □█┴╫`□°₧¿Γ>░ƒ¿

/q9|$╪Q□äéj

äô`i«~Y·□ó╨|\/╞⌡□²╝⌠□qI╪□D▌□i□₧ß[ñ≈±

sû0g  sû□ÑÑÅ⌠ï╥zÇ·Jÿ/ó\ë┤     φ?┴╜╧≡|3└|%╟W┬<%╟□╤»D·□m÷α┘w░≈é∩┴>░□ ¬t□∩╞/<
┐≡$?ε□╒o#RûH'07ⁿ¬ô╬ú∩/╕.╞5jl 5╢⌠+└/□Θ*┌ùékα:╕□╨≡m7⌡ïr░~YVü□╝ L┐-
çâ□P□Dé□─%GüÜá□êFU╪□─ÇX╨□≈Γí[Ç□D▌╓á¡^"'□Uí$Xa@□

□□(└ \ □╕ü□□□□h└
B@□α#.Ñ*□□╒@□□□□á:ê□ÿ°éy*ÿ°éy*╡A□P□╘□⌡□Γî╥□4BDl

Üα║□"gs\╟δ%ê─%J□«[ü╓ ╟█¥ç╟U▀≈-?₧æ%{$í□╠□┬R(□RBB ICÜöª-┘╙╥6╡6╣Mtoü`C ï-
□ëYb□┬f│┘ÿÿ┼□a≡Rû□╙b▌Γ*Çcßx,,$Å□╦Æegfä╞3-ΘXÄE┬∩╛fópin√⌠÷yε╙?▐ÖEg╬·}?ƒ∩≥;â
Ñαâò╬,Ä□ß╙₧_Ç╧ä▄┤╧·▄¼01φ+╓vëƒ}╒τ.┼l╟ü;▌i÷ò╙«┬|╫╜□╫ßz╟▀∞zj^°.N[ΩqÖs▌ç-
`9-s╛║▒╝╧Wcì≈b╟φ≈┘╫├─⌠(

Of□Ñª0╚╣║i8=σ±φ▐?,j╥═ï╙MÑΘGz∩(□□÷N?□╟V╛æ¼T≈Σ^ΩfU9T▌ùmx≤²à▐_P²□Ñ▓╟*GS□-
□_L^P·f*JU╛╒¬■∞Å□∩
┘┴┘°@╚%>Ω±πß╒─'┬KëOßé╨σL□;è¼□E653╝ö║□7y■}▄î[p+n├φXä┼╕□wΓ.▄ì{░□Kq/ûß
>ⁿ□╦q?-└âx□+≡0~êG╨□▓
∩□┘(iÑπëÖεå+δ?╫·cδÅ□□□δÅ□≤xs╪ò╕%∞╥╖N╨│NpΣK⌐┐ Ö╘▀Γï°∩°√░+5□ùπ□|□sqSê┼□ï-
□[,╢Xl▒╪b▒┼bï┼□ï-□[,╢Xl▒╪b▒┼bï┼□ï-□[,╢Xl▒╪b▒┼bï┼□ï-
□[,╢Xlq²'├«·Oß╚╕□f┴g±9|>∞□{╠├□äφ□╩$¬>å÷Ωw□╟ï}ì╕╫$╛□┌□□ßk╕9t╥á│r7"÷5b_#÷5b
_#÷N▒wè╝S∞¥b∩□{gΩΩ╨₧j┼╖q□╛□┌¡½╙‖:¡½╙‖:¡½╙‖:¡½╙‖:ú≤8╨┬ü□k╦rá┼·&dPY□ò¡≤τV2d
%C╔/╝q└z¢&∩f╬ÿ╝¢9c≥;┬î∞*╦«▓╒
Y▌É╒
Y▌É╒
Y▌□gZ8╚┬Ö□╬┤pªà3-£ißL
gZ8╚┬Ö□╬┤pªà3-£ißL
gZ8╚┬Ö□╬┤pªà3-£ißL
gZ8╚┬Ö□╬┤pªà3-□□ó└□□å(0Dü!□

Q`ê□C£ië>Fàf*4≤b+□Ü·▒5±±Φ□╤╧□²,n╜█ δ├ô≈╨∩¢£½gN╬╒3'∩ï¢y╡òW[y╡òW[⌐1ï□│¿1ï□│
¿1ï□│¿╤Lìfj4SúÖ□═╘hªF35Ü⌐╤Lìfj4SúÖ□═╘hªF35Ü⌐╤Lìfj4SúÖ□═╘hªF35Ü⌐╤Lìfj4SúÖ□
═╘hª╞,j╟ó╞,j╟ó╞,j╟ó╞,j╟óFsT'□╩">M─╫èx╛ê□□ßò"ⁿRt4ì₧ª╧╙┤ΘóM□□ÜhP·∩G½┼ ┤°ƒ□
╙Γ□Zⁿ]Γ∩□□ù°╗─▀%■.δΦ▓Ä.δΦ▓Ä.δΦ▓Ä.δΦ▓Ä.╡2¢╥ ║▀ìFg$>/KgΩu│⌡╡9z▄e╕□Wä₧Ω7□┐δu
≤⌡îδ┬K⌡▀

┘·∩`>«┼u╕-7α╖XÇà°-nä▐X»7╓δì⌡zc╜▐X»7╓δì⌡zc╜▐X»7╓δï⌡·b╜╛X»/╓δï⌡·b╜╛X»/-2□)╘
δyS¬▀~U╓-½±>5▐°╞√ΦVO╖·j⌡\□·╘nƒ┌φS╖}j╥╧┌ckÅ¡=╢÷╪┌ckÅ¡=╢÷╪┌ckÅ¡=╢÷╪┌ckÅ¡=╢÷
╪┌ckÅ¡=╢÷╪┌ckÅ¡=╢÷╪┌ckÅ¡=╢÷╪┌ckÅ¡=╢÷╪┌+=kf╪Aφ

à▛▀∞YòêvDgë¿═╧□Θτ□▄□τ╞87╞□√s╟╛║▒-
V))æ₧¬RRó=U-▌^Θ²□□τ╜╕(█D┘&╨6Q╢ë▓Mömól□e¢(█D┘&╨6Q╢ë▓Mömól□e¢(█D┘&╨6Q╢ë▓Möm
ól□e¢(█D┘&╨6Q╢ë▓Mömól□e¢(█ósD╥╨¢-╝┘Æÿ□□┴ƒ-
"╕X□□T└næ▄.ÆcEr║HÄ□╔Θ"Y$Æ°x╖àw[x╖àw[x╖ET¡ój□U½¿ZE╒*¬VQ╡è¬UT¡ój□U½¿ZE╒*¬VQ
╡è¬UT¡ój□U½¿ZE╒*¬VQ╡è¬UT¡ój□U½¿ZE╒*¬VQ╡è¬UT¡Ωxf╡Ä?(èmóxf≥┐╟V÷□+úz±vè╖S¼¥Γ
:\Lç√╔ôΓΘ□O°x:┼╙)₧╬¿61Å╟-
2°¬0£XΦ╙╖¢□K*▀▒{≈`ba□Åª°▀□╤iÄ8É╕┌{¡╒≈╖&nîª'n≥i{·─╥Φmëe▐┐/□¼□□Ä┼;q□Ä╟
8□□Çïp1.┴Wq)fc

.├σ°□«└ò°:╛üoΓ[ÿïy░╛ ·½`M⌡╓T□M8Xìτáòf□≤CI,╣─=íÿ╕╫·/L|S_√□µy≈jQ╢Γ║░-
q=n└w▒0zgΓ╞≡|b▒πε□²ë;q□ε╞▓░I|¢Ω□zY□5ÿèZ╘a□ª#àz4á□çá o╞█q(

□Ä#p$Ä╤╤8□∩□e□ûiXªaÖåe□ûiXªa╣■Ca[²╣°0■□-
┴G±g8□□ÄÅß/≡ù°+£ÅÅπ□╕H□□π□|□ùb6µα2\Ä»ß□\ë»π□°&╛à╣ÿç□\à½╤èk┬ª¿Fµ∞óΓ
□ç□K├δriaxM₧□ê>╟àÿ
±[2⌐╟─)Ü8EG□⌐□'*╗┤┐□E□ªh┬□Mÿó S4aè╘Å⌐S?ª~L²ÿ·1⌡cΩ╟╘Å⌐-
S?ª~L²ÿ·1⌡cΩ╟╘Å⌐S?ª~L²ÿ·1⌡cΩ╟╘Å ├

■ñu|□ƒ╞□°

>ï╧ß≤╕╙9.╞%°*.┼l╠┴e╕□_├□╕□_╟7@□Ω╞╘ì⌐□S7ªnL▌ÿ║1uπh□u□d°╕

/$«ò├
ú□╘─ó÷□╡╥┬╫i▄Aπ

Ö₧udÜ╓YZg□╫¿╘·£╕╓'»
√d■>Ö┐Oµ∩sûZ>lµ├f>ö□ït╟;┬n□░[□∞V□╗╒╥½zC'Åzx╘├ú=<┌╟ú=<┌╟ú=<┌╟ú

-u≡¿âG□<ΩαQ□Å:x╘┴ú

─u≡¿âG□<ΩαQ□Å:x╘┴ú

-u≡¿âG□<ΩαQ□Å:x╘┴ú

-eyöσQûGY-eyöσQûGY□▓Oà∞S!√T╚>□▓Oà∞S!√T╚>□▓Oà∞S!√T╚>□▓Oà∞S!√T╚>-oµ±f-oµ±f-oµ±f-oµ±f-≈≡╕ç╟=<εßq□Å{x▄├π--≈≡╕ç╟=<εßq□Å{x▄├π--≈≡╕ç╟=<εßq□Å{x▄□═µ`üâ□

╞ⁿnτb╟╢□£+q«╟╢2τ╩£½°□$ ƒπ^ü{à─¡▐╗¥╙ï├□□▄╦┘╜□▄╦┘╜□▄╟┴1y╥═┼<□≤\,p▒└┼□□
\,p▒└┼□□
\,p▒└┼□□
\,p▒└┼□□
\,p▒└┼□□
\,p▒└┼□□
\,p▒└┼□□
\,p▒└┼□ù╩*s⌐╠Ñ2ù╩*s⌐╠Ñ2ù╩*s⌐╠Ñ2ù╩*s⌐╠Ñ2ù□*p⌐└Ñ□ù□*p⌐└Ñ□ù≥\╨s)╧Ñ<ù≥\
╨s)┴Ñ<ù≥\╨s)┴Ñ<ù≥\╨s)╧Ñ<ù≥\╨s)╧Ñ<ù≥\╨s)╜çKπ\□»Vπ┬¿ë
e.îqaî□π□¿▄7ìQwî║c╘□úε□u╟¿;N▌qΩÄSw£║π╘□°ε8u╟⌐;N▌qΩÄSw£║π╘□°ε8u╟⌐;N▌qΩÄSw£
║π╘□°ε8u╟⌐;N¥1ΩîQgî:c╘□ú╤□u╞¿3□¥«3LΦ

□║≡-≤<ò╕U□╖U≤╟Ω=_èe~~_ÿPq□*nB┼M¿╕ □7íΓ&T▄äè¢á⌡□¡'h=Aδ
ZO╨zé╓□┤₧á⌠□¡'h=Aδ ZO╨zé╓□┤₧á⌡□¡'h=Aδ
ZO╨zé╓□╤Ñ┤-ñ⌡á□□¼╕╥┐r¬ °□r¬ W╒ w□░Xû▀í□▐ë╗p7∞α□òo6■²lΣ╟ ?□·1╚ÅA~

≥cÉâ$^{n}$□Σ╟ ?□·1╚ÅA~

≥cÉâⁿ□Σ╟ ?□·1╚ÅA~

≥cÉâⁿ□ñ`üé□□□(Xá`üé□□□(X⌐å£j╙⌐å£j╙⌐å£j╙⌐å£j╙⌐å£j╙⌐å£j╙⌐å£j╙⌐å£j╙²?TCûCY

e9öσPûCY

e9öσPûCY

e9öσPûCY

e9öσPûCY

e9öσPûCY

e½3╛dW║+z ¢▌δ-□╟^Æ÷□┌ ╫töïp1.┴Wq)fâτb,ê▒ ╞é□
b,ê▒ ╞é□
b,╘Wra-Zp□Σ¢□
b,╪π╣êΦ ╘LA┼╟·mÑ╥╟⌡╘± ¿F∞▌[∞▒□╩π□σδ¡₧▀f»┤╪▌≈╥Φ╨Φ3ö+R«X▌ò╧╟╡ÄZΦ±f} □╕∩S¢òΘ
\÷⌐╙¬╗█%₧/
ú□-ò▌%┘]Æ▌%┘]Æ▌%┘]ó|æ≥E╩□)_ñ|æ≥E╩□)_ñ|æ≥E╩□)_ñ|æ≥E╩□)_ñ|æ≥E╩□)_ñ|æ≥E╩□)_ñ
|Q÷òd_I÷òd_I÷òd_I÷òd_ë3ú£□σ╟(gF93╩ÖQ╬îrfö3ú£□σ╟(gF93╩ÖQ╬îrfö3ú£□σ╟(gF93╨Ö
Q╬îV∩V

Pj≤¢≈-σ(Y╜»q'=Ñ╫ú/╨║ù╢╜ⁿ+±»dûε≈╙□£¿°o₧╛·j [╠Ñ{tö%vJ╦∞`∩
├t=╙5O╫<]≤t=°*│!□zΘ┌K╪^║÷╥╡ù«╜tφÑk/]{Θ┌K╫^║÷╥╡ù«╜tφÑk/]{Θ┌K╫^║÷╥╡ù«╜tφÑk/
]{σTINòΣTINòΣTINòΣTINòΦ₧°{₧εy║τΘ₧°{₧εy║τΘ>L≈a║□╙}ÿε├tª√0▌çΘ>L≈a║□╙}ÿε├t-
ª√0▌çΘ>L≈a║□╙}ÿε├tª√pUπèε#4■Uthb¥Lε□/%~"/_□s□?
Å$╞┬╧□√├-ëâßòdc□J₧□FÆgå╟ôgç┴7 ¥≥□úcÆ -
jÜⁿ≈╩C▄jπ╞□*∞'▓ □{╪□9±□~¬╥6qf│τi{╤W9┘π▒□·Φ≡─░)╢▀τ╞}■□&\-
□□╔:Lâ┘ΦΩ╣Σ{╜□□▐çs┬╛Σ╣awCs(4\□:□.â■╜pÑGj4PúA?h°╢╟·!▀p-
«├□∩▌µ╜█▒□εw□ε÷▐═╕╫s┘╙p┐s┤à±åU╬ $₧□#
OπG▐√▒╫╧y□SCù≈^┴6l≈:â_x╛□âÄ█□□□╞p

4╬□·╞├q□Ä╟ 8┘√sBgπ
₧[WπMa╕±÷0╥╕□≈ß□;ûON¬║ïG»Su;U√⌐┌O╒▀Pu□UsT▌N╒Q¬n°Ωvj□⌐YáfüÆ□J□(YáΓ□*û⌐XªbÖ
é%□εóαv□n°α.□n°`Äé9□εó`ε≈□▄E┴~□÷S░ƒé9□εóα.□÷S░ƒé█⌐Wó^ëzeΩò)WóXÖbeèò)UªTÖR
%J□(UáTüR□J□(UáTüR□J□(╡}R⌐]öΩ°TÖReJò)UêNL¼

│□δ┬ZJmöâ┐ª╨□T╓'våo╩│δ□├aÑ╠₧¥ê├zÖ}í<δO&C&Y□û'□┬≈½Ö>#£Ö<>Üôⁿ├pô¼ ≤Σ╖├▀Sφ'2
╙r«=·æ≡H≥╝p╤Σ7R²ô *yNrv╪á□┌ú□W∩σS»½ ╟╒÷≡"φjC╬^p╞1gδu╢▓□:W
¥□□b▌π>╡═°

·TÑ>╞¡≈,ƒ╬LV` ▌║÷Z╫▒╬╨δ

YgΦë□½æ╛`τ⌠╙≡öO╝╧'v╣▐

ƒΩ□╤δ>╣╥°Äƒⁿ T╞°□ów╧¿ÆO□e╥ÿL□ôE#▓(ûE├«╜_□
╥óaY1,+åe─░îêeD,□b┘PÆ
%┘PÆ  c2aL&î╓äX□î╓Ç1□0╠▒aÄò╕5ª╟τúôô╒ °-╫╧èy,±O╓≡□6à_U
∩L□pu(:□╓·│╬ƒm╕╧δ□C╤y▓QìO□┤≥K|ó°Γ¼┘▒:╝╟≤□∩÷x7¥É]U²vΩ□3h≈à╨π╝=╤LW]ΣΦδ╒R╓'₧
r⌡·«>▀'□Pb?%÷;├÷─f≈µi╫yò"=-{æ      k£q¥

┌û(╚å□fä½ôfj╥LMÜ⌐╓ô┬éΣ╓°C-ƒΩ⌡i8├■Ωl╛□╘≤≤Bl5ƒ░ÜO¿╢,u□R≈áÜ╦R°`├╫ú□
▀Ç¥□□µ7|█≤·a□%□Qbæ║╦R{?╡≈S{□├b?┐█{≈α^»ùß>ƒ╗▀╣-⌠°□ö{□φaA├ï-□å-Hú□?G┐ƒ
x▄àí░á1□?i£□╓4╓ó

'z}□µäâ□Xñ÷▓▄▄▀┐ö#≈b□~Ç□┬□□╣úÜëC£■+]τ
]τ
]τ
«□Là┐í┬▀Pßo¿µ7óc·Qª}ü÷Y┌g}¬±¡╜I∞e▒ù┼^□wV▄YqWb═è5√f_·7zè╡û¡3√╓-1%σè≤d└≈╕┐₧
√
╕┐ ±╧□}-□¬⌡┼Φê─K°⌐-▓Y₧n≤~Ñ□dL┼>w▀?╟/░□²╪□nJ

x□┬n·╖╟π/æC>║A╢ⁿ(±Üτ#(8╟^ÅEö\w╧₧Åb,\¡'uδ╪9□;°zgWzSΓu∩²□┐
»&▐≡□T⌡□$PΘ[5▓m¬τ╡ßI□97Y_¡·δT²`▓)▄ô|□▐ÄC1#£'[/ö¡□╧╓
=╡╓c┬èΣ;ⁿ∞X□-
²]≥DÅ□Çô┬□2·□Ö|m≥□» □°åÖ2zf≥t╧ □gä┐╓□τΩ*[╕╢Ük½╣╢Z╢□Nƒ\ƒ|┐c>Ç□å-
' ─πçpnhK~╪πƒΓ#aæ¬╕0·g₧ƒ□«W□ùΦ°╗⌠╙╧┐╠╛ &yat\≥╦ÿ□╢V╛#oÿ□╢5╠┴╫úCT╓!*dü□9Dû╠ô
%≤d╔╝å□ⁿⁿ╗°>n╞─╕-:óßv,┬b╟/±▐R▄δ⌡2▄τ<╦╜~╨πCß₧åç±□┌┬┌åG├□S¼¡a╡╫k░□ □f¬¬Ö&[¢

\-□W█□¼5▌ ┌□₧ ?nXçg□≈£≈┌├□
╥≤□╞≤▐□╤τΣV├&τ}┘{¢±3∩mA□]╬⌡□╢í█±█□¢Aƒƒ²□┐≡■

⌠;∩╬╨¡rgÜ₧m¬≈B╒{A├n∩╓┴□9╪Éà<l╙c8⌡4╙├□y╪PÇ□l(a-
╩Γ-┼╕τ┐□»6□─äτ┐ü£kÉs║┬■Fy╫(∩□ôß╒╞□ÅS╜Wï:L≤z║εæé□ll□=ìì8─≤&╛═√o╟í8╞√3B╬ä╧Ö
≡╣╞#¥∩(╟□ìc≡

□ïw:÷x??□'║╞-
xOç╒ìµ6^□╢║⌐≡yì7EG4≥║æ╫ì╝n╝□╖ß÷░║±ε░Bσ»╓⌐fΩT3u¬Ö║└j▌jfπrτy└y-r╬G£┐═δG▒□Åà□
╒¥─Ñ║─ÅuàN;ë□□ßƒué_¿°¢U÷╖T÷□U╕V╒vÿ╖▒è²G□╗GUnWì/¬┬°Tß6U≈
òu▒JzD┼▄¬b~¼bv⌐Æ[U╔fU≡╝∞_>·;N╧╩■g½ M√¢ak⌠?⌡½òV▓╥─┌öx╥î^□6δ[ÅΦ[ÅXUÑ{
■ôε·éε·é╔╡jråwÿüy½▌czuÿ^□·╫*+□Iƒ╩Zy║2┴¼:°■∞╤o÷X·N²║■╨╟⌡∞~═╗□r┬=ª□¼╥
VYσ~½╝▓≥[□ª╫ªå aÅ{qΦ0┴:L░M&X╟¢{ä□»»

ÅLε□V¬╧òΩsÑ╢┌┴}G├≈p+n
/ΦΩ/ΦΩ/T≈

```
w√·═╕╫δe╕╧9εw ▌□═╢çU≥~ò<_%°│µI┐y╥/o│fJ┐\═NN»U≥rò╝\%□│rmÅ\█#╫÷╙¡¼▄╜╜╙½═≥jOu║
¥l'·█ ╫!°VÜp¢LÄ□Σ╟*·æò{óyª─FSbú|╪
□─Ñt╔t╪(□>½¢wδµò.■□U√⌐║ì¬█Σ─Åtε□╨vΘ╘▌ öφólù▄(W;⌠□ßU▌°U▌°U9rû□9¿╥÷Θ▓}ô√╡.¥╡
]gm╫Y█σ╟V▌⌠□]tô╬·¬Ä╕QG▄H⌡□╒K╘.ΘÇ□u└ì:αF□pú
```

╕æ▓%]oú«╖Q°█¿úm╥┼·t▒>]lô.╓«ï╡δ`¢t░Wt░Wt½Wt½>▌⌐OwΩ╙¥·t°v▌⌐]wj╫¥^╤ò·tÑ>]⌐]W
j╫ì·túM║╤½▄Θ╥Y║uûn.uq¿Kw□╨]□tÉ□▌ó[╖¿tån¥í[gΦµ╘6Nmπ╘6]a@□Φµ╘6NmS·▌£ΩR·□UⁿF
□┐Q┼oT±□UⁿF□▀«┌█U{ƒj∩Sφ}¬╝]╡≈⌐÷JòoSσ▌¬╝[òw½≥n≈┴y;π╩₧·∞0□¥ú╩b□╒¼óû¿¿%*Ω°|n
S5□°║Æ»+·║R╡Σ°ZΣδ□₧«ßΘ□□□½éÿ□m╝hS□ò¥r¢îÅe·□Y╛Dû/ßE¢,Åeye°╝Dû/æ═□Φ╡åNkd≤□Z
¡íUæVEY}Ç^EÖ|Ç>+Θ│Æ>+ΘSö═□d≤□□¡ñ╤J·¼æ╝▒∞]"s□êyÑ□_□7╩╪²"x╥½1k▀─
-ö¢ÖΦ□æò╝Ω□┘Ç╚□Dû□╒╓·@Nd/ï∞e½½▄¥╜lu/[]╓Ω^╢¬Æ□ò¼h╘è□¼h└jJVS▓Ü□½□░Üù¡ód□□╤±
«4V╝/□w╡□ÿ░Kⁿì}rT▌╜ö]¡█╒*╙j╠╒*9╙φjc«VÖJc┤□s╒1Zî╡≥ÿ+≈╢rƒ+≈╤b╠╒╟\}╠╒√\╜╧╔╗]
}╠╒√▄#∞

≈ï|½¿╖║r┘□|z┘□u▄φ:εv=φ□□wsTδ¿±╓√°≥Σo,¥æ£□¥□¥¬╧s¬<τê□G∞·▌▌╡#□D2.Æ┤*»Φû□IZ□
i□ÉS□9╤ñEÆ□╓┐H╞E1«□r* °□r*
°□r Ω╬≈H╟╝╙{┐╖□>╓≤ôCZ6τ*w╖▓9'¢s▓9'¢sUo□ae┐¬z;╒½╤Ωw*□1íô╘V~□╓«Ω²vU∩╖W╧êí□÷
·YA»▀½wε╒;≈Φ¥{⌠╫Jo▄½/ε╒□≈8█╫j▐╝Z=S▓¬`9:┼9╓·╓s▄□q«⌡Ä╪≈ª.÷□4□í╟□=F\c²Σ┐▒lσ≥
□}FΦ2┬σ□┌îpw─□╓[├:kXg
δ8=≥»4yç╫╟ΓwÜ£Φ°ô╜>┼π□Ä□¿·¥I!Ü"·rtñ⌡iL╬╣□┌┤úR╣╓4d┘┐┤«!δ□▓Ä!δ□▓å!╫─qφ□╫«\w
çδεp▌□«╖├⌡v╕╓ÉδT«▒#:┘┘□²zæ╖┐e□Tε⌡╫╗R▒┌≤S╒□⌐s≈dªφ¿εl┐⌐?N÷F□╖╖Ωc«·ÿ½>÷o÷┼J─
<╤qò─xè╟J?{└▒┐▀╧ª[═?Z┴╬Ω╖
╡╒▀ï¥π╧[]yδΣ∩ mî╬▓εî#_αZ┌]K╓·7Qi□ò╓S⌐▓÷°etE⌐gx]┘□□⌐⌡

╡₧□╧&g}╪┘ ╓s1mgYÖ─╧P≡□NV▓ⁿ□Y₧ôσ9Äª┼╖I╢τ─ÿ□cFî□«ªφ□│vêY╗┴╨ä^OΘ⌡ö^/δs\Ns9M⌡⌡
T_/÷Mö□F∞¢─¥ßrÜ□δúwP╜ïΩ]bε□AI▄ b╒□σ╗¼╕h┼E½+R╗ï┌]VY┤┬"ò╗¿▄Eσ.*wQ╣ï╨]□εrÑ"à
╗¿█E▌.ΩvQ╖K}φ□w╤f□=åeÿëá₧╬4│╧
┐èÆ÷J[¬▀«¥□vF'z╡┐·¡σIz▄╔xo□5╟G═±QGî¢ß#vTÑ╔o□G╠ß□sx╘□-¥ⁿûqñ·-
c╗╛≈█o□G═▐Q│w⌠-▀4ÄÜ╗úvEcµεê¥╤ÿ98j

ÄÜ}ú╤t;ì□Vr┐¥E╣·
ε┘!∩¬ò▀Hx£âÅW┐╡¥f/RN╬░µ3¬▀□ε«~_qÄO□!·K²∩╕¿╞9vW╧qfx╜≥╜½h·τ°!╟εó┬

□¥□

T⌡x▌│bt╕gσ▀√ª▒ÿ╝╨╫≈╤aùêï".╛σ¢┴Γ┐≤═`±¡w≡╤
«T·6x/]≈╨u╧∩}#£wò╜4▌δ□{]a∩[╛╣▌δ*{i║ùª{h║≈≈╛╜▌K╚╝○~{█∩ÿA»çt┬╥|#□M□u□
¥£l¼:■¿=▄ÿ=▄ÿ=▄ÿ5=kM╧RΩÇ}\╔>«ΣΦ╤Ωw}─
⌡≤≤¬┐σ╖Ä≥δ⌠ß□⌠ß╨┐°╫┘ïò∞┼J╓⌠¼=W╔₧½d╧U▓τ*┘cò∞▒J╓≤¼²U╔▐j╟Ü₧╡╧)┘τö∞sJ÷8Ñ¿╫j~Σ
╨q⌡□╞èâτ╣≥□Bç½uD'·Θ.║φ┤╞□╓╕├æòo╘_ú▀0²åΘ7L┐A·□¿|OE├¥4<@├□4□ªß0
w╥≡□
wZδ

□εñß0
çi8L├¥4▄I├a□

[≤

□-░▐□4□ªß0
çú#¿6@╡□¬
P¬ƒR²╓╜├║3ö□áH?E·┌╤Oì~j⌠Súƒ□²╘Φ°D?%□¿╨Oà~*⌠Sí?:F£y1µ┼ÿ»¬qª3┐╫D>
∩├□╔╦S·╘╙x╞≤uh□y√▌Q▒ñ┼Æ□K┌■vT□iqñ┼æ□C^

i1ñ┼É«■

gσ_□□-ï.╥ .╞%°Vx<‖&▄□}□▀┴|\ï▌ß╤h□~ëQ╟□

ïú    ╝Ä_π7a±öSC≈ö╙p:▐à?╞□x7╬─{≡^£à≈ßl£â≈π□° ■□┬╢°0■□-
┴G±g8□□ÄÅß/≡ù°+£ÅÅπ□°$>àOπ□|□ƒ┼∞Φ╕) □:°tä□°ⁿ□/αE╝äƒb□:±26ç□k-□w╘¼└├╪Γu□[!
╓Ü7□┬Γ⌐o
+°-□-¥:#tO=□GαH□àú1□εÿZp╟^∞
w╘₧å≈π≥░▓÷k╕□Wb^x╝╣□t»]□║k╗┬ï╡πí╗εö≡b▌ßT£å│≡>|□□åGδ╛ä/ç┼u≈ó
â^∩┬□xV7□-»{
%?ï╜-□ï°%B≈┤$╠≈iSQ
√╫i÷»╙╠∩iµ≈┤z4á□çá      f·43}ÜÖ>φ0ⁿIxq┌ç≡□╧/±x╜╟╟<>Ä²í{║sM?,╝□²]t¿î;

3p8Ä└æ°#£è╙p:▐àOß╙╕□ƒ┴g±9|-□ì┐┴□1
□à╡2w¡╟]+soëµ║Gÿç□\à½qMxB6?!¢ƒÉ═O╙µ'jn
ΘÜ[q□TE=",╞□╕□wßn▄□□S│□□·▄□<□₧α·┌┌█Cz¬ΩÜ┌Å□

z?δ1çéƒ∩┼>∩²&ñkka_];□)□àú±ç8□t¿Ñâ∞xó÷lÅ∩≈x«╟≤±w°2╛éf\-╓╩£╡2g¡╞Y+snæ9╖╘è╖V
╝2ΦëiWV┤ëε┤°║
wπ-,┴R╪oEò²╓πXà┌x□¢±3lA□[╤àW░
▌x□=╚`wX°'¼╙□╓Θ ▌æ{₧(□∩#╣□╣≈╤'6Φ□□⌠ë
·─□}bCM>t╫

π5îá□≈L5E╪ç╓╪ç╓╪_╓8gìs╓8gMσso ä
Ωm]¥^P°÷δ╘z¥Z»SτuΩ╜εo±□\Φÿ/ß╦aC▌e^╧┼<\à½±□▄ê¢á▐ΩhTGú:□╒╤H=m¿√í╟6ÅOzl□□ΩΦP
Gç::¿╡ujm¥Z[°╓╓┌┤n╡╓]'ª:1┌╢
jn]□=╘▌å)∩Äj∞Fªó□uÿ□U■ƒ>ÄïR¿Gσ ¡░□çαC╤□╤╢╕(,ùπ╦σ°r9╛\Ä»Éπ+Σ°□9╛BÄ»êZúCσ·B
y╛P₧/öτ
σ·┬ ─▀Æ:+ZÅ▌a)Gùrt)G╫p⌠yÄ>╧╤τ9·<GƒÅ~□╜¥½ï╕║ê½ï╕║ê½ï■½~/>±₧ΦΦ─{ú3□g{ⁿ(>-û'
>□û&>à╧GG%fç╒ë9ß╖ë╦py°«=█□╓/à∩█╖]æⁿè╟╣εdµÖ╙]QS≥òhF▓□=ªlot\rw╪É▄π⌡/úSô┘Ω_u
8)·Ü╟æ¿⌐fnt\=<┤α*\ìV\âoπ;ÿÅkq]⌡∩h-╘/□Ω□
⌠□GK╢/Æφïd√"╜fy⌡w≥□
K⌡ÿàSGóC⌡ùσ·╦r²eß╘╪úπjôÉ[╡çΓ0£ä╙┬┬┌╙=╛□∩ï╬╨S□╓~└≤╦├r²c╢■░\ X«□,╫?ûδ-
+⌠Å□╡r⌐÷□╚Ñ7□╫┐;

²_┐╥_·]ⁿ╧äτU┌Rò╢TÑ-z≤∩p²εopU■÷╓╝▐ φ▀:K5-¬■
«Ä╟∩┬□Σ£╨Yúr╓¿£τU╬≤u{ú╖╫□Qr|∞τ≥O□-
¬ⁿ¥« o┐ú ╓┐⌡⌡û▀╡»ⁿ-}jfXÜ□Wj~°nΩ:¿¢ö║I⌐¢ö║I⌐¢ö║I▌ÄEXî; ▐╘]╕□≈`
ûΓ^,├}°□ûπ~<Ç□Aƒ╘□<îΓ□┤EG╫□;:¬■;ÿÅkq□«╟
°.□`!╛ç□q□╛Å¢q
n┼m╕□ï░□wα.▄ì{░□Kq/ûß╛Φ¿åwEG-
2=:ΩÉ□Ωúú∞□╖¬é▌╒┐b▓╡·ùOÄK\Ñ¢5ΘfM║Yôn╓ñ¢5ΘfM║Yôn╓ñ¢5ΘfM║Yô□@┐□@┐□@┐□@┐□@┐□
@┐□@┐□@┐□@┐□@┐□@┐╬wÆ╬wÆ¥@╬N g'É│□╚╛ Σ∞□rv□9;ü£¥@╬N g'É╙%τΩÆsu╔╣╤Ñε┤fc

.├σ°□«└òò□½Äoα¢°Vh²7;Ω5ß|▌⌠|▌⌠|▌⌠|▌⌠|▌4Ñ¢ªt╙önÜ╥MS║iJ7MΘª)▌4Ñ¢ª╟▌¼╣¢5w|µn
╓▄═Ü╗Ys7kεf═▌¼╣¢5w|:∩I:∩Iµo┘ⁿ-¢┐e≤╖l■û═▀▓·[6□╤µo┘ⁿ-¢┐e≤╖l■ûuδ;uδ;uδ;ú\(Dy

π5îáÇ╝(óä}(c4ⁿHg□Ng□Ng□Ng□Ng□NW_á½/╨╒□ΦΩ
t⌡□÷⌠□{·î=}╞₧>cOƒ▒°╧╪╙g∞Θ3÷⌠□{·î=}╞₧>cOƒ▒°╧╪╙g∞Θ3÷⌠□{·î=}╞₧>cOƒ▒°╧╪╙g∞Θ3÷
⌠□{·î=}╞₧>cOƒ▒°╧╪╙g∞Θ3÷⌠□{·î=}╞₧>cOƒ▒°╧╪╙g∞Θ3S>□□=σ≤°kⁿ
■□?□iô(m□ÑMó┤Iö6ë╥&Q┌$J¢Diô(m□ÑMó┤Iö6ë╥&Q┌$J¢Diô(m□ÑMó┤Iö6ë╥&Q┌$J¢Diô(φ^b
╜{ë
ε%6╕ù╪α^bâ{ë
ε%╓╗ùX∩^b╜{ë⌡ε%╓O·YöÜ▓□ilìRªXô)vê)╓öp┐cÆ5%▄╙ÿf╧Öf□Öf□Uº┘ùB!q□fç{▀:╒□_½■uù
≤M╢║9&█·&[σ»$=ÖⁿVx,┘nè=-
5&;┬M╓¡ß)S«╓öKÖr9S.ò▄-åL║5ô□╕Φ╕Ω▀╣|═√#╤TS«╓ök2σÜL╡&S«╓ök2σÜL╡&S«╓ök2σÜL╡&
;Θ£¥t╪N:g'¥│ô╬╛Iτ∞ñsv╥9;Θ£¥t╫N:g'¥│ô╬╒▄□╩5╥p~Çσ╕-
□αA<□╪79╧79╧w▀╕▐}╫z≈]δM╤ö)Ü2ESªh╩□MÖó)S4eèªL╤ö)Ü2ESªh╩>│lƒY╢╧,█gûφ3╥÷Öe√╟
▓}f┘>│lƒY╢╧,█gûφ3╥5√Cíf□□≡+□─□^╟»í&Lµ□&≤□ôy«╓£6Ö∩t ùq ùq ùq ùq ùq ùqù≡┐⌐;
□°(è┤ W╙╒se□ä#ê▄α⌡ΓJt╫ z╜-δΩ╛▐¼╟*(óé«.Ω«□□ΣP□□E□T`□Q□─]π½■]Q
°ó«Φ:□□ê╚xä#ÇßhÄp□ª▄▀«Θäp□H@▐≈▀≤·uWW╫±t╒S┐z₧Ω₧Ö□^B□/í□/!┼

~Ihà≥≡□Rx□)f≥|f≥ⁿ□2àÉë□²□f⌠□^C*öµ\)╧□└□&░DéÖ>üGæ┬úHßQñ≡(R╠ⁿ
f■□₧E□╧"σ4#φ±á
qφ8o□αZ╜î□û┴%X□ τ4«wΣx║hâ╫æ┬B╕□
!üτæ┬≤Hßyñ≡<Rx-)<Å□ûC>ûC>ûC>ûC╛□Å:≡¿□Å:□Ç|╨K⌡╞ÜΦ]eM└í°│┼X□I,ëñ3^D£╖E«≤

xÅ≡□□?σ8 □be$□·□┐╖╪±□æ≤xò─ΓHbq$▒8Æ°┬à°┬à°┬│≡àgaü$±çgß□□╨│E□ƒ╕□┐└├/≡≡
<ⁿ□□┐`□V╧Tⁿ□□┐└├Z□ë╡2R▐ñ╨Σ═á½□ê□α╓₧ä□S≥^p°3°
e▐□╕/|çU°

-╛âç∩αaßD░p"°□->ä'ƒ"²╙·ù
=¼₧□■äç?ßßOx°□-V╨@¼á□VP□ⁿ□□Kh ûP□▀┬├╖≡≡-<|
□▀┬├╖≡░ÉFb!ì─B□ëà4R«á∞ò`□Çδ%\Å╒⌠.V╙╗XMS▒Üªb-
─Z□ë╡4□ki
╓R□_┐□_┐□_┐□_┐□_┐□_┐□_┐□_┐□_┐□_┐□_┐□_┐□_┐□_┐□_┐□_┐□_┐□_┐□½+ë╒ò─ΩJbu%▒║ÆX]
I¼«$VW□½+ë╒ò─ΩJbu%▒║ÆX]I¼«$VW□½+ë╒òt≤ÉΘt≡kUΦ₧□ní∞█9∩

zÇ;ê╗ôπ┴]αnpƒ*┼BKbí%░╨ÆεúΣyû°╫I√w5╦²□ß7└VU□□"□
.□µ▐╤
Ta╕íêD«QE□ⁿ┬╚⌡α□╒□╦«{Σ&┬□½▓Ho≡□¿┤⌠-#<□

□ ,╛□□_□ï/ü┼ù└ΓK`±%░°□X| ,╛□□_□ï/ü┼ù└ΓK`±%░°□X|
,╛□□_□ï/ü┼ù└ΓK`±%░°□X|
,╛□□_□ï/ü┼ù└ΓKⁿ□Z|ë╜,╛åb╕‖╪Φ*:□▌└¡Γaπ6q½q╗╕┌Φ.‖¢┐□□Üwës¼δ╘╛╓
¬ôU¿□¡Ö¬╗Uóè░
s¼□·7^'X½U╥Zâ/╡□□δ'U.ZêßΘ╒ó@¡□ƒ¿□ö~n≡ï┤WS·□ö~A≡K▓σ■○ESK.╡D¿σ\j╣äZFX╙╒□╓

0SE¼┘□?R╦¡Å)}Äzà┌'Psà╡R╫~□╡Åú÷□╡Oí÷"ßZIR╟G&<yk!▓□⌐╣╓╫─-
fF\Bè□▓}ël_Æ▓□sgÆ╘□H=ä╘
I]@ΩδÖGgæú?9□èV■∩K"φ½╠µ ┴∞}ùy□3·]jÿy» n°he╬Q╜╟□⌐    µwΓls+■h

÷≤/╘√╓tf▀ÖΓ4εαsj*─ìX
╡/ÜdûNPz□w⌠□3⌡É`ªÄ□>iä;≤¼5▄ò■ÑA╡┴°â░╒d□□

É└□a□±┐¥
b

▓@□╧>□£ÑÆΓl0P
□OÇAαI0□

□C┴Sαi0

W┃┼T⌡₧(T∩□&÷Å□1□□

É└□a□□Q□□┘Çy╥¿□□□╕─ÇK

ꟷ ―ÇK

ꟻ —ÇK

╕├Ç;

ㅋㅏÇ;

╕├Ç;î÷α□p╡*2:ü▀□├╢┴╪6·ü■`□x□<□-□□┴□`□x□

□C└□5╫□ Füτ└≤α□0□îQs═╙╘P≤

p-ΦD∩
UI≤)zfª·=╜Rå₧òúc∩╨□eÖ▀|Σ╜<²▒╡MσX█╙)kG║╚┌Ö~├¬H□[╗╥S¡▌*jÑëWΘ2;ö■╪vTÄ-
╙)█M□┘ß⌠□v$]lG╙Sφÿè┌qΓ│Hù»&█╜└âα!≡0Φ
·ÇG@_╨□⌠□□□╢¡ìmkc█┌╪║6╢¡ìmkc█┌╪║6╢¡ìmkc█┌╪║6╢¡ìmkc█┌╪║6╢¡ìmkc█┌S└ç¬╙₧□□┴4
0□╠□3┴,0□|□>□s└'`í□j□ü»┴"░□,□┼α□░□|
ûüö□□¬Pô□
á┐NH□8⌡96□m└╔á#8□╗αLÄ├Tæ3□îσ£√t^#╠²8▄Å├²8▄Å≤6q∩Çw┴?┴□`*±à`□ÿ

É▌ Avτ
┬_é□□■□$┴<░□,Qs¥Ñ\+□?□□l□¢┴□░□lSE2
$@6¿□Ü¿╣2□4□╟üfα

∞ö3┴_╘Py?x□<□FéW└□⌡₧,α╕M
uOPEε)╟q°r<ìπòα*┬7¬╣εφ\∩

z□⌠╤□Kⁿ_┴ïα%P□*╘▄░PEßz□□_a╞Uÿ9:╠ⁿ□╣□▄
zé{┴ƒA>`╝G□∩□╞{ä±-a╝G□∩æg└p≡,□□É72□<□₧□/Ç╤`

□
■□^□/üqαe0-pÅæW┴□≡70□LRCúù¬d⌠2p9╕□p»╤½└╒á□xDMê÷□²@□0□<□-□Ååâüα  0□<
□â!`(x□<
åüg└p≡,□□Füτ└≤α□0□î□c┴_╒ä╪)jhVXM╙èÇ¿Ü l╪ □ÿ┐╘Z─\╣äy∞□╤□■|□⌠□²@□░□.▌
⋆└.░□«:Qy°╧─■│ç ∞ß?{°╧─■│ç ∞ß?{°╧─■│ç ∞ß?{°╧─■│ç ∞ß?{°╧─■│ç ∞ß?{°╧─
■│ç ∞ß?{°╧─■│ç ∞ß?{°╧─■│ç ∞ß?{°╧─■│ç ∞ß?{°╧─■│ç ∞·┐□f|ªR°¼e°¼e°¼e°¼e°¼e°í
»ßç╛å▀Ö┬∩Lßwª╟I¬ä□m23┘j│\¡3╖⌐u·¢M-
ßw╫c6Ü»R╟`ô±ß□≡ß□≡ß□≡ß╩≡ß╩≡ß| )ë ö─□JΓ3y°L->ôç╧Σß3y°L->R□~P□~J□>I□>D□>äçÅ
α é¿çPåP&OV)yè■5P ù@}[>ë¥¥─╢Nb
'▒üô╪┐-÷»ç²δa z╪┐-÷»ç²δa z╪┐-÷»ç²δa z╪┐-÷»ç²δa z╪┐-÷»ç╝Zå╝Zå╝Ωaú·┐╨Ö┬

⌡░A╦░;=∞M□{⌠,£úR╪ÿ»ac╛åMÖ┬ªL┼·⌐ÆX□0@ò─s╘║xC╩□┤□-
┴c─O╘o7¡PôÖ╫▒1¡Bq║5M▄n═□m¡┘ó)φ√oδc╤╨Ü#N░ÆΓ2┌·2φ╫/□□α█'¼»E-φ^µ»bcτö□╗\t└^╕
L»a√▀gXâ╒ÆY╥╥ú ªÅ╘T╥O╒u╛├╡▀┬ó╛□ë+≥Sè¿q╡ê□¥└∩┴5αZpù╚├{ïα╝·₧[□/-
é┼□aä╟□═□□τΦ▀Df>DåLLsf╤RbOd╢,`╢,╥÷
▐85/╟□Z#.╨kè~┌<d≡ □a□□g~?Y ¬┤o□·╧M⌠∩╧▌á□X·┤═GΦ╩╣"A▐□╘B╬ûæz□╢αl╡ò│□╬zÆo╢┌┴
┘Bqé░)=□□ ü+ⁿ □╟□□Q□□qÉEì╫ëzVg⌡/½+ΦI+NSï)Θ{JÜoτï<╗□x□<□-□╝A≡□Φ
·ü▀`Ç╚├ù╧├g╧├g╧├G╧├G╧├'╧├ ╬├≈╬├▀F□-k!6▌4┌jå·╤Ü┼(Ü¡╛í╞iX╖δ╣≈|q□:QÅ½₧»
▄{Ä¿o╟□╟□
D╗α╝┤-VgRe~⌐·□ ùÜ¡₧·;]_Z□b▀Ä□'Yc@íZCO╖╞Æy╫▀╡8┘>K┤ú╡║ê,rdQ╧/Φ═|z`åZOM_ΩÜΓ╘
≡□5$¡¢¿ f,╨n□oσÿO-
≤╒2lΣ2∞π]Z□□ï□╣"┬± ìà╛╣ñ╟%e.)=Rl□ì─rX□□J¼╟ⁿzƒ«±AÄ≡□╜-éqï)o
¼╗ò□₧ ªo□çΩ½r|°r|°r|Σr|Σr|Σr|Σr|▀rΩ╝Ä{╜üR≥Θ╣$╣ⁿ╥ⁿ□╙╞{╒y□σw□≈□C╫=ÅûƒOⁿ□Ω[H
;□í9ï░╟□ïΦa╒□
Ω-í┤□wQAë%öXFë-%:┴Ω[H╧Yñ÷¼□┤□)ΣHY≈δ>nâ─╥≥□╣9#K99ú╚RAn▀C±─⌐b╢8S¼□+┴

╤^∞□□`□╪-┌Sr7φ-▌─8╗Y\guπx+╟{≡dε°Σ□╒□½/=9□M-
├ê┼Ωíì┌Ω╛Y¿▐╒╡}¡û0µr≡rví#yΦH₧M┘v□(╤>T_£);â.á½h/╟ÇIα□╬□□%□9σ□Γ╢p,G╢0Æò#Q□ñ
Θ└╜µ□╝├∞╩□≡√x :πk┌,ΣƒE╥öÆ:ç╓)%G

9≥H□F╬u┤╟fd⌡Éu╗▀«:WRδ'}ä.┐a∞ûú╧m¼^0aëh£▒╫╤╫Rz╟ ₧╓□5G ôÅ▀g)REê┘è□ò┐□□╝□c=Ç
Ä<─°_ì>¼í²¥α7φK╔□╗q□½└□ò□╣ó;Æ⌠□wÇ□⌠?□ö#O□YÆñ╬╤⌐ùSú÷Γ╕╢□F╘δ«╞ïτèµílU□*□δT⌐
╙□▄□ε□≈ü^αA╩═□■□┴ %╬□%°¼□╕ú^▄i
²┤\¡σNwdεTmCΩ□jÖ½}∩╞╚τ!ƒç|^╒(ΘLI]┴□╙╓ï~)!τrd≈²Φî╖Θ▀▌□■□
!ƒç|-≥y╚τ!ƒç|₧π?SΘ ≡▄E□p□Φ├·#á/Φ□·Srµ_ôNéú▓é▀í≈□τ□8j

¡<àV■□╜,D/╧A//╢▐@_KÉl9≈ªÑa₧*Ñ╧V½□:y&:yª}«*╢_□□∞W┴□╤!ö-.

²└▒îπ:░QtpN≥ƒ}é₧Γbτ-p/╕□°≥╣A·:□□t&ñ√jò╓□O»>□ ≈Σ
UnÉ*□╣=Rµi┘ⁿ■w¼₧Θ╫¡φj□╛^╨ûj□╛\╧>1²92≈L @l91σ÷ëΩ?(╡gzëUNOUÉ{□%φV%vHφ░#¬┬╞-
!e );Ω╝oq╡ÿÿbJ█¬≤&¡¥≡äƒw7┌á╙□□Rτìßâeq<Q5□⌡I·9╡TαòzHVf·oàWPδ.╡ô£
╙YN¡□xú-□ù┘XEö▓□ vR╥□JB▐⌠Å⌠TOⁿ╪L)[)ÑéR╥╛╠║εLε¡Σ«
wZ╤₧æ!$□æ│'2öX█h│φ□w╨~X╓┴¥□[╗□╙i╡ÆÆv
Këφê\J+í┤r;╟,ƒi□ε_äφ╕ZI╓;ÉΘ□□╓LùPó▀□ÑVÜ9GΩ√/╡πäOTB°x[≈╙N¥*╙+a¥╩∩Öà┤ε>²à=□
⌠□╣□╤?:¡ε□╥-ó?D÷æ÷âê┌┤²╤Γú▄εΦx
φ¡»□░¥Eû¥#\╗!Ñ6□□╗)8Ä<═╚□<a¼U╗□╫Z□n
┌q¡=╫N≡¡J╗□e□╟╒û□█·m`τpå╧`7&MS}╒╙e5'╛□ßVä█Ω╘₧_ÄptΩ&║╓¡:Ek]╤VQ╣B\-
│□□╙□4□═æ/A╨2╧lÄ|ö
Zp▐Æδ¡@kΓ█Æª□qφ ƒ@□YöRè¼■□å∞\jo*¼á□?w)≥√w□▓█p¡-╫2╣C"□□"Σ^°∩┤
σ6%╒q┤^3Γ3⌡G(a¥nü╓\oK\;«╗'▐»¢╗áⁿå\mñ6┌ì²{Eπ┤

⌠e3Ω=₧╕┐µñiA\K╥┤≥█Ç4Z□╥┤'═ 0¥▀O ▌«MDN╨O□╚æâ□Y╚æ╨m█Ü≤L?U C

2d·╜ó[/□Σ┌▓ù⌠■}grl⌐Æ:QW¥`╘~Mh-
╜`┤╖□±┌Ω□╣┌0Jk╨□«Üó┴╤╖□JkHL□⌡ä▄1Q∩HuàR□·wtt⌠à₧ÿñ√▒N:ú∩(^[╜í╬φX│σΘ□pa□□╟å╒
:Z;╙3a╡π¼]Θ9░╧»¡t║□V╦╢CΘ□pc□╪╙å╒:┌ß⌠LXφ8;Ü₧□3²┌Ä°+`5╞`·□Zñ)-
□°EΓvô⌠τ┤HC╕iz□R╡ÑUlZ┼┤¢ô«□ΘZÆª□hM║6ñkK║vñkO║□╨Ü0₧Z□-
δb╦ □í9┌¬╧┴╨mÄUæτ»█cφσΩ□2*4║è│ìnΓbπV±┤q□╟█╔σ ∩╨⌡ΩSδ□XC7¿q·▀±N:H¬Ou¬╩□\□Wu
÷N╒Ö□~□□XD
│°π
╖□GeS▀⌐∩E□µAí□□≡zà□«▐ΓS«-µ∞□╒C╜⌐ª□*╤WK░fà·1H[╛_n j⌐≥°∞╢û│_¬
αëCJ:□ⁿ▀jτ┼ö▐╨»í╞-
óv·╥⌐═ä²7Ü.┴â□╠fò%¼«□ò□á╛□jÖZú╛ΣSó6┬¡G║5ª╟Wu╔╤UÖÜ[Y╖*█»µ2▌je*Eδ▀*Äú┼N≥%□«
V□¬"╡U¡W¢╘j╡▓*¬□▒δ⌡╡≈Φ╜äzƒ╨è□µ%ò┬wí┐╫□┐╒Üï6Γⁿî⌠\Y¼□ú-
▀√í□Ω-»╞·w⌐zü+╒àΩ15ÿ╨≈U╫□¬~ù√Σ¡á¡┐úεÅ╘°▄╜GOàé+▀∞ô≥│C╢┴□□hÜ□í≈₧┌@Θü□VkÖ╩⌠[
i▒Mj╖ZD║K⌡▌₧M╤□R¬╡j-
√5A┌φ√σ▐@¢¡≥u$□□σó⌐>□╒│╖5╙¥┌δ∞O╒┬╙□»□╢S≈╘HÅ□ëÉZtêZ²□╕689Y£q╩┤ô╒ï╛₧°:T√M¡⌠
∩□φZ╢▀ò□Öw#□¿Coε█â>;□"≈rP¿□iΘ₧æ□╕□Z╜U∩ï

p1qX%l□?╘╣▌∩∞α8Ñ

y_╓√╤ⁿ√?╨█Yç¼{uª_╒

╕t}-K?x½₧
«╙uⁿÿ┘g>┴╒□═Ä'±i┴τñ╝$£¼≈≤2ƒâΣεx╘▄½⌠~¥┌□wm⌐IT«·¼╢V}δÅC?Oå├3s-l≈ë·B²½
╞▄╒fU5T┤éæ»□W□■çÄ)b₧ÜÄ≈ZSεj≤û□┼<É+~+n⌠GÉÄ·û▒≡╓-v«⌐n□□EÅⁿ▄gê;*π╒T⌡□slì╝┤çδ
â-A√u&┘╥┘:C}¿f½ÖA┌u√σ«6│╙R =□·│╧σ:µ□j/Tà5╓]â]É÷-é/╒ì¬ô·ô║.H╖-
ô⌐í┤δτΩ+⌡²^<cè«bá□Fh╕x╓□GX╝)bΓ-
±ü8QL□3E₧ÿ═τWbÄXî~~#Vè╤D⌐aê□înX~□ÿ¥╠▀ï│ï┘E<hv7{êç╠╗={Do│╪,□²╠o═∩D□│─\.-7
W¢½┼□µZ≤'1╚▄`n□â=rs¢□jVÿ□ΓiSYB

│L
√╨ÆVTî░ⁿ_Éx┴║╔║Yî╢║Y╖è▒÷√÷√ΓE{í¡─Kí·í·b«3┼Ö"╛pf83┼ù╬╖╬2±òú□%µ╔▀╙K─|y⌐╝R,Æ
W╤Nb⌐╝V^+û╔δσDJvû╖ê∩σφ▓╕X)∩ÆwëR·'·░X-√╙■ó\>*□□≥I·┤╪-ƒò#
K>'╟□Ä|Q╛h─Σ89╬ê╤±≥□#KNöôîlY °□⌡σ□9╫h#□╙M╞╔rïT╞σ«σåî□\╫ì□]▄ÿ¢etuδ╣
î[▌åncú╖█╘mf▄Θ6w[□w╖m▄v╞═ε□║τ□□vow{□≈╖â▌!F/≈i≈iπ!≈-≈-πßHƒH-
úwñodÇ╤'·Htä╤?:/║╔x>û□kb|□{3÷ª±Qlylú±qlsl╖1?╢3╢╦X□K╟à±M▄î¢╞▓╕□wîT<□Å□?─│π
ìîò±&±&FY╝i╝Ö▒.▐<▐╓╪□oooliï┐□□╔┼-□9>┴╪□ƒ□□▌Hgà│óªò╒0½▒Θdm╧┌iå╤¢EZo

¡7&z3□K~îx□√µ%1ëÿ╫°H1Y╝!\QÇV9Z½□┤j║□ï□ΦVDδV□▌ÜKⁿ□Γk□Ñ╘EΣ]╟'Ä╢-
├ïOë□╞╪r4»Ñ(□-úf□ƒVb│╪&Zïφ|┌ê□b╖h+╥Φe=¡ù═┤^ZZ/cZ/cΦeOæm▐âv╞┤v╓G;Sóæ·□:┌□□
-□ì=σhΩqZS¢jMm¼5╡í╓╘\¡⌐
Le*╤└┬æ┴w2-
ô=¢hê╓J┬t╗hbà╤α□¡┴M╤α¢D;δf⌠╕=z▄ì≡¡hs{¡══╨µö0∞∩∞ò┬┤W┘Ñ┬▒W█δE╘▐`o□╟█[φr|╒m÷
.╝⌡▌Φ}[¡≈-╡▐7╙z▀Lδ}3¡≈═╨√▀ê□yæ╝HDσoσoà-/f$ä□
ù□sÖ╝îÿ╥σσB╧+Σ□┬òW2BZ3B«&o'╞IXÅô(πΣz□ù□`┤d1Z║êû≥&y│H╙«▓½h+oaⁿ╘╙π°₧-
?□πτOΣΩ) LÜ┐╙√ëy@> LÖ/{Q╤â≥AJ~ê1□eî=B«┘▓/±²d?╥≈g╘┼⌡¿3□uâI3D

Ñ▌°□ü Fα│─îÉ#╚5RÄ$=sr41cΣ□$□+╟□├╚□□□d□□dÄ'╫+≥□Γ'╩ëö3IN"eü, µM·□y▀ûo╚

∩╚≈hÖ)r*r□╩B┌dÜ£åTsΣ°H√Ö£KÖ
$:)□I┤Q.æK)φ[·╜h!□É╦iô□r5u¡ækE+·ô,ú%╫╔⌡óì▄ 7PπF╢ Ö╖╚-
ñ▄*╖r╡\û□┐MnCÆφr□σ∩ö;)╣BVP≥.╣K4É╗σnjO╦4yòT"Ω≤êhµ≤□{xä=<┬--a□Å░çG╪├#∞ß□÷≡ê
0αæ┴∞ç╕CäΘ|ë░}6□å╧&"□¢⌠e▀/2@d√£",8e▒êEùDïE<·Mtô╚÷·EX>┐ê&≡╦r╤
╢"╢BΣ─V╞Vèx1Ulòh□+iòruul╡h□[□[#Äï¡ì¡#╜>╢₧⌠□b□H|1╢æ4¢c¢
oëm□╣▒≥X9i╢┼╢ôfgl'W+b╗D4ûÄ)╤8╬≡□
|µbo╟m÷í╕#Ω├_□╤0-ìGI□ï╟┼qpY□brΓìD«╧hó□î╓ö²q±fñi-o!rΓ-π-
)íU╝5ß6±6ño□oK□╛#-╛#µσ°x╩□%■*╣&─'P≥─°$╩|-■║hΦ3á╨

8éÅU┼Çc ┐□≈YÜ√B0▀¢ä▀□▓ƒ*□5□╬&ⁿ1╝gëOß>
ε[□W.□K□□≤æÜ√,═}9Ü√□jε
kεkñ╣»▒µ╛&Ü√r5≈Eìäæ□1ú│╤Ö}O□ª3ε3εgƒoΣ│□╧x□εδdv□ªfF□f∞╬▐g╞êfFW3c\│a□│╟⌠□σ█
g└zÜ□δ¢╗═▙"Ks_┬▓-[╘â⌡\┬□+"▓¡╫Vgq£╒┼Ω"Ä╫¼╫L│^s½½╒ò°[¼[ê≈□░Öf└µ╓m╓φói□□û□
ε█"$¼╖Kä5▀σj╛k(╧ùτ3>/ö□□K≤Üä╤.c∩sÖÑ╣,ñ╣¼▒╝J^Eî╧eû╝F^├■Zy□)}□k¿Y,¼Y,□□δ╞╪╛
M▐╞■vy;){╙-∞∩öw▓≈□MjF
□îû/≤ëΘ□úà4ùI┘[÷╓î╓ç⌠>úI□m□ß

ù=.□□÷□MjF│4úàσ09î\╧╙ß─°∞&5╗E□v□%G Ks£╘□ù½┘=Æ/├kV└k»╩W
OÉ□ä# & FJƒΘ,=t╣╒ÿ╬╥L'a║B┬>╗I9]~DxÄ£╧▐g7 ╗-
%∞≤ZÄµ╡åÜ╫┬Ü╫□i^k¼y¡ëµ╡\=kQ╢Yn&ù╧n
5╗5╓∞û□░█.X╠╥,□u
╫□VåÅ"□Gz
7≥HΣ□÷²"²D$2□÷ëD-ï<F╠á╚ ßj&2úúó□□ªµö□▒u░I"µ┼αS= =□
αÄmä╗╟vê,X#=H÷Y#;n┼-
æ□_H□┼|QO≤E□ÿó>aƒ)Ω╟□╟□ô╞τê□±ππ╟□▀□Ä¿□G┤ó□ƒ#ΩiÄHhÄ╚╓□Q□ÄxÖ2_ë┐B«ë±ëñƒ□;╘╙
∞`□│├
■jf╟¥τ<üGrmMvⁿ µMmR%>t╪█{σª*M╣Zy╨5╧Ü╧÷Wd┐□s⌡┘wòq╛≈óW□+ⁿ□▓╟z□Rx{»`╓∞□□╫□□╟
;j/┘╤┌T□5N7□VΩ□ò⌠╜╜├]G½▒£▓╜├■:k╒Z┘&╝╛□ò≥[S-
⌐J╡°≈éòk▌µ■w7¢ïäƒZ╟φ╗÷²│næ@ÆΩ╡&─□Ω╕÷φ}╡~ ⌡.┤τ+5Wm½ïn-zS≤âπ≥@ô7V╗╢╣Rz-
┼□·S-;≡X:*Æ╒║d⌡¬□úÅσj>Ü1□╝Ñ₧W
â~»Æ_»,╬Gç>»╙x/□╒₧Bd₧¢T╗:LmäG╧é□]φKR-
sÑ6l=îz┤║ï□>φ8╥ì₧▄#²□┌j=≡Wì╢φòjφ■9 ╗mUk^k

OWÄöæ

Z÷üV¢kN²ÖÜó>Q∩°<E8▌▓Y□¼Q«⌐J╡j□╖╒ó∞○²⌡╤Ç√╓Ω'@-

Γ?□y+S>τs8■╤□ß╜ ╓3Uü≡·)»≥«`▌"XΩ<╤J-╓<      P╤UR-
çW«≡□┘V²ΘVµΘæ·∩¬≤ù╒▌j¿Ωªf□╛⌐*÷B╒SM╒3═>¡~ ûΓ

□╒,t╝╞╡╙:╨╜I3M ╜/ën±Ω┃ûW}e\-=hiƒ]Θj┃┴F┴≤7ò┐╧òO╘á¬p╒

åF°|▒éÖ⌡á≈TCm>c·}í█Fδτ┌á¥╪½^║-⌐ƒ□∩;Sτê

√öσ[□▀1gEⁿÆ□█`Gp=;T¢-
å¼{ÿ▓┌S░Jn╟╪#p|⌐«k/=╙π¡t┐·╜¼«╧òΩ║e¼╥jτ5Z?╒ƒ`Vï¥vtσ⌐V≥u╡H¼ƒ≤¿!┴3┼rF⌠*
ízG□d₧□ε5┐{üû╜»▐¡â\╙▒
>□┬ƒ├╤·y«?>}□└╞( ₧⌐ökf-

¼ï

ï╞≈)kûµ₧)Üτge₧ü¿/÷J▒╖÷□□9□èjO█□µ\¿9hû

┠àÜ7?╩hAµëdft□W~½.╥g3╘□┤Σ▌αq⌡

╟├□Ω╪O÷¬φƒ┤z┘ ·}□Σ╝Oì≤╣¢√ æP□BÅß!îSo0□ÄP¥╘(▀c ╓≈□▐V□3cF▌⌐3τT>O
╧*b┤c·ï□u8πe□╓ù TO┐?ΓδG□▐□╤ZS⌡d;3□□ßö□|ƒ=~£╪█6k╣∩{□? V▌å⌠ƒ╓⌐u■¼□╨□√╪≈╟f█δ
╡ª~▓«╓□▄□╙¡|l╜4Q╜=╤ƒφ┌Ä┌zp @sL□Σ¼··s-╩8ªφú╞½'╘p⌡á

ùαìNV□
«ö⌐»⌡q□L╝nÅσV°Z.TπÅP╬o±╜Æ┴J╟□╡X²╖┌;d┌«╞πÖ°6W╜?P╖Z

▒fs╨╝╦}█¢c□ⁿ□√<ÿ
⌠√□■╗=┌Γ»Θ¥¡c╖┴┌▌ö
öM⌠┘C£?Ç°ó=g┐□Tà·@ìTg1ç$ß≡Wδ╓sj¼>┤9"I3²:'8
╝╪╟JÇ¿µM□·Vï≈║j*aúnAƒç╫`»ε╫╦_Ω{}G█W⌐φåe╡□)2■ΦZ⌠tc╡kzûAÅ ═□√ΓÇ┘Å┘å£□╒▀]üù
µⁿ╧IsáM⌡P7·

Θ√3∞çs■Ä·Jç□Å□=°@]¡å ■ ·ín:v¼√□φ╪qlk¼▌V╔·Ω°²▀¡E)?δ□X`Qû1gm8▓u╛‖«□°╧'

3σ█ ·mπ}▀□½φ┌Ω□≤÷╞□□□k}jΣ╤ôñå□□~WδÅñτÅµ▄Vc□)╡≤X»Y╘~S-
jƒßH█πäú"╠╧╢□Θ7□ÿiΩ≡┤F»%W¡~Θwä+╟VñµQªmΣ6ó│Éu¿▒¼.¼φ≈■-□-X
<╝╖╟c·□σ -
╢▄║d≥╫≡δÉka⌡Ö┼ -□≤T·╧≤□≥τ╪░_╖□z╞R╗ΩPrQ]▐╨╫û Ü╜╬*█2|É\╛□τè╦╨╤c╝· ▐hUxì÷□~<8
□Θ⌡≡c╛nS]╩#*τ╟□ƒεwΘñα╗□9╒╛wP¢Æτ╤n≤*k±C□ò▀à¿¼∩l]╙^≥T;□╝°┤□ô3╟j¢ ¥çÄ■Q□f ▐╫¿
Ñ£ô╔79□δÉ^√.

εíRéÄ√╚9╣÷5Uσ²ß└▀d<D«o¬▀╣_┬■O_j▄Ω┤╥@/¡:t¬²r¡
╞╗~µ»ƒ□U╛O□9╚7Pⁿ√╚□□╘e╝½UçZ□>`«Ñ□2O5ⁿ╒φ⌡"x║qÉ\Ö╒╥▄╝╟ƒ*V½⌡╖=O□═8Ωgú╠
>┌Ω╨┌tcφσ;¿∞Θ}ò╧»·¿njé□úƒ

∩□3] ╘■⌠▒]b ≈.

≡
AO¡√yV≤⌡□!ÖgU┼╪8Ex°┼╪╫U▀î╤Olⁿòⁿ≤╒⌡·ⁿ
u?⌐εVƒsG-
¿?□δÜ{=╙╥≤H□ue□ñΘI⌐W□a□╥▀□-úª¿┘Ω□uï·DkD«~▓╜░╥úR≈°qó¥ tH=áε╙qσ┤·≈ΩUεeèzG²#
xé│╫□û₧□₧U╧╒A╬IΩ│¬╔╧╘□÷o□÷╙r⌡«zÄ╕ìA╥p5╧?├Çmk_▀▒▐Ä┼□□¡UÖ≈□÷╙≈cP{¬N╧π╓êj+0
ü÷□║£zá╛╕Xç█b╫╖□¡²√gd· ╟≡□─ë≡Q
(e⌠ò2r.ç'□Ωt¥>ZU[?uq□╠<y■ñΩ√£2≤÷KÉε├□d╧0▐□°^╧8jÇΩñ■□□ë╓Ωl¥$αw²
∞s╒àΩNu3í□>É∩U⌡åÜ½▀╜╓╘╓R┤□Y□⌡w╤╤°éC╢├■2╝ôApV╚═U{Ä□╝]ôçÑ┘B° £T·=≥Ö╒╥4JoR1⌡
□╡□^ÜÑεúî▒j8≈U¿₧⌐▐*ó≥√▄Åg°íûr÷F_2▀□

□║O▌ÑPts╤:T¼▀°îg8┐Ü'ñ┐yPtsy3α░φÇ╜k\╗ w□□#ù□î]φßΩg7¢àú/%

2┐√9r┼9⌠┐)>Ñ¥²7Z╬□τëôÖ≤/□°╤■╫`c]/■@lgq□) (-□ eÿFC╤▌hnt□□□ù□Wê!╞U╡Γ)ú│q│□
a▄e▄%₧7z□≈è□î^╞Sb¼1╠□#▐2^4^□à╞8c£ÿfî7╞ïΘ╞?î7─

π#c₧ÿe₧fv□I≤

≤Wb╛ yûyû(2╧3╧□ ¢┐1/□ï╟ 2/□K╟√═□─R≤a│╖XfÄ0ƒ□▀Öô╟Ió─|▌|K,7▀7?□?ÖS═⌐b¥9▌£)╓¢
ƒÿƒ□╧£k╫□¢═»╟ñ╤b╫7□êr│╙,□█═┼µb▒├èYq▒╙╨╢Ωï]VÄò#ö╒╙jj□½ò╒┌□Ym¡╢å┤N░N2\½úògD
¡_Y┐2Γ╓Y╓┘Fûu«⌡ƒF╢u╛u╛Q▀║╙║╙h`u╢║□9VWδ□úæ ▐
úë¥o□6Ü┌O┘SîSφ□∞ÖFg√+{₧q[(□è□=B▒P╟╤ #ö□¬g▄□╨ σ□wàèB▀□≈àûà~0-

-□-7-

¡□¡2·åVç╓□²B?à6□□B¢B¢îAí¡í□π╔╨n'd<πH'╦□φd;┘╞x°╛╙╨x┼iΓ47&:¡£╙ì7£_:┐4 f8g:ù□3¥½£δîy╬╧╪╬π╞bτ

τIcù3╘y┌P╬│╬│ªΘîtFÖû≤é≤é□r╞8πL╟y╒Öh╞£╫£╫╠l°└·°Y╧y■Ön6uf9- ¢¡£╧£╧╟v╬\g╛┘▌)rè═S¥Ñ╬R≤

τ;τ{≤ùN⌐≤ôyªπ9╖╠sñÉª·;)eK≤2┘^₧a÷Ég╔s═╷ █□µ├«ΘÜfo╫qÑ┘╟ì║Q┃»¢p┃═~nÄ¢c

p¢╕╣║µúε±ε±µπn+╖╡9╨m∩╢7□╣'║'¢O║°╖0ç║□▌Äµ╙ε□ε□µ0≈╫εYµ3ε9ε╣µ│ε□ε□µH≈wεÑµ(≈r
≈r≤□≈J≈*s┤{ì{¥9╔╝╤╜╤│╔╝├╜╙□τ▐τ■┼□∩µ╗·µ□╗Å█╟ⁿ¢█╧φgNtu-
7'╣C▌íµδε0wÿ·w≈Ywä·□wö;╧,pG╗/Öo║»╗□7▀u
▄□≤=wô╗┘£Γnu╗Ü-
╕█ φµça□6╠⌐a;l¢╙┬2,═Θa6sF8□╬6gå□ä□Ü│├╣ßfµ£p≤ps≤_æk"¥═╧#▌"▌╟·æ┴æ7═□æ┘æ╧═mæ
Eæeûê|□Yi╡æ□╤ªVv⌠┌ΦφV₧ M

δ┬Φ√╤Ö╓Ñ1□KX╫─Në²╓║)╓%÷Gδí╪ƒb□╢□┼εÅσ[O╟-î=l
Å⌡ë⌡▒F╞·╞□Y Å▓│ÅÅ≥¬÷²₧gf÷L┬₧!@□!ÖÖ╟√√[f&≤■╞ï)"RL#r□□æC9╙Eñ□1"Mæ"Ñ□)╥ö"Rñê
H)EDèêörÉr(□╣4RDJ□╣ê╚ADJæ"╝┐╝&DÄ ]≤Y┐Y«g?{&ô╔┌▀╒~≥δ*▒T¼P»□+┼J⌡KbòX¡▐$║┼□⌡
Å─F▒Q╝Sl□/½□*^□╗╘┐□»ë_¿▀□┐□┐T□»ï╫╒oè7─□⌡□qL£Tƒ□=óG²Ä8%N½Oï3Γ=⌡∩─∩┼□⌠∩
Å□-W ┴≡äßI⌡E├R├3Ω?□₧5|W²□├sån⌡
├
å□╘7ìèQ»~▀(îF⌡]π@c¡·#cú╤¼Qî□?╨h√│■*
gèΩ
t(#:Q□V├T8[□05N╫!¿╓│Ft^□síε╞ùÄyXÇΘY□□¡□wΣp÷σY□gj□▌MPw□╘▌

Φnπq╫g≡�together=εs╪{2√"εÿ╓█∩µΓy-├Wü═c≤┘ ÷u|╒▓N╢É
fÅú□╓í□□6DeP□Y=²u╪PU
·c□·ú□□»╧╤B*ƒ╨Åz@□@-D▀□B}3î╛·0t□║g□▌s□ΩƒC□ìP□ìP□ìóç.@²¢¬o│f╒R╒R∞·□║ΩPt╒∩░
ÿjÑΩ9□Wuú├å⌐├å⌐├å⌐├å╨a□î|+·l□}÷WlñΩ░Ω0K⌐▐T╜┼╥¬cΦ╜□Ω╜□:o□┌é■╦⌐ □⌐ *╘□ì╘□□R
-R
R MP mD ²1│([ò¡╞ñ╜¼╜┬¼╩vtd□ud□uΣ&tΣ}╨_ó/¢⌐/;¿/¢╨ù 7⌠8║s□║≤
Φ AÅ6SÅ6SÅ╢úG
·▀─F°vQ°÷P°vúS╫3ƒz¿z(≤½□╘
¼$╗6rtmµE╫÷@╝jεB∩f□┘╗qWV¥àµ╘9\-¿
╨ó║ê5ΦπP⌠qTΣ▀┘
°┐│□A□[7£■╢n□²═▌0⌠⌠╟YV╙Ñ·6Sí│»d□═│Ün╓óy^│å
╨╜áY╧ÆÜ□5?`â5□5»░!ÜφÜƒ▒zt ╫XD│G│ƒ5╦3ÇÑσ□└¬σ□□¡╤╓░╝vÇv□
╦ôÇEp□⌠0╡÷7┌▀░&φ)φ)f╨┘ú}çi┤°╡┐eZ£□gQyW√.*τ┤ τÿN√₧÷=ª╫₧╫₧gâΣ╓┴·╓ô□k.k/│■┌?i
─jp~ⁿÖ⌐┤W╡ ìτ║ª²
□á╜«╜╬□╦□□╧│╛÷}V°╜Ñ╜┼2┌□┤-
αU▌╓▐╞+·╗÷∩╚∩h∩ P√!╦j í²□v╛╟□6Ç½╣åe╡ûkÖ□τÉÄíìs=δ╟½x53≡~╜-
Ss┴□½π□n`□nΣF¼┴Y┼·π¼□ä{k·`▄[╧çb}□od5▄─═╪┘┬─╕╫╞mP;╗c□□w`╜ô;▒▐├╜X∩π>6ÿ√╢-
⌡□□0
□≥
□<─├╪?┬#╕7╧ú╪¡Ö7cMî╟po£╟Y╡<□±\)₧B══│XÖπ9∞Éτeªσ├°╟░▓ò╖2□□ê?ä╫ⁿ0 □╛»6■i∞ 9>
□╧■□>□╧≥E>□√<╧┐─r|□ 2╦≤9|-₧±½╜â□°╫8·□ :∩d╡ⁿ□ⁿ□x╡
°B|/Å≤.∞≤□□□;,Γï░├b╛□√?╓ƒ─╒%| ÷╟┘╟å╧│Öàp6?╦Ü·*╛èEσ
=åαä~-W╫≡5¼₧┐└±╗╧┐╟┐╟╥|□_ç≈y□▀□²□▀╚"ⁿ%■□╓π□╟

/≤ùí█8>Ö|;▀Ä{_σ;XÖ ä □;∩Σ?┼╔▌|7ε}ì┐å·-╛□+□┴≈aσδⁿ□«╛┴□▓ÿ<√Q /■_XyöE■□□
kÄ±_c═ ~□»Σm■6^U□
^τ)~è5≡w°;,╬O≤╙╕
¼Ç⌡τ°9∞÷-□□δ ╙ ê}.≤+X g■g¼ +□kn±[x□>αα⌡▄µw┘□╔□,□₧0 7Ω□░f▌@▌
6TW½□┬b║z¥ë┼uf¥òàA□-û╓yu>6Rτ╫□XJ□╘□Q Θ",úïΩó╪íY╫îò1]

kΓ║8«&t ╘|║,₧%°╥ae^ùG╜á+αYΣ▀É¬$╡░êñ□(¿□□jüéZáá□(¿□□jüéZX╜ñ□6TR
□╘┬□$╡ □╡░┤ñ□6DR
╓âZÉâZp□╘□□╡░ÿñ□□□╡L╟·G⌡Å▓

╪σ╥╟áƒú □╓Ç`p/□□u□

VvΘ╗░╧□·'É/╥/B□4âW□Ü┴·∩Φ┐├Ü⌡+⌡+q□ÿåE┴4▌¿<»╟°K┐F =Σ?╥ □╧╡E┐àìööâ╨
²
∞≡7²▀░□¼├BÆu╪╨*·□>╧U¬*□□"ë□□□□□ c!□□╬╟¬Ü¬□□□≈

bΘ¬┌¬Z□¡□\5ÿe└@CYsUCU□k¿j¼jDn¬2aP□k□□=┬î╒φ╒φîW□║·╙╙╟WÅG■ÖΩ╧ ƒP=æ
ö╞ä╧╖½71Ñ·ç╒█Éâ£Éâ£░□Σä5□∩°bJ?Ñ_□+J~bë╩_┬J~bèΣ'(°
·Y±Yf□ô─$╓$>'>╟·ï╓b2│ê╧ï╧3╖ÿ"ª0¢°é°□Sï⌐Γ▀æO□╙▒■Q±(╓╞□3░µKΓK╙gë ┼□b╢
ÿì5_□s░f«ÿï½Åëy╞

&√□Ω≤┼|╘Af╨□b□⌠¢b!k□Åï.f□OêEX·-±-
¼\,₧─3.□Oú▓\¼└╬á7<╥*▒□·]▒□k║┼≤x=k─□∞≤éXïⁿ{Γ{X┐N¼C■}±}∞╢^¼╟┌□┼ï╟-6ê
╟+ÖÅy└|¢ÿ_ⁿPⁿÉò─f±cΣ[┼V¼yY╝î½»èWí;─OX@∞□;q⌡°b□«╛&÷0ƒ°╣╪ï╨/─/P□)BAè╨7─Aµ□
)

a═»─aµ□oè7▒≥ê8ég9&~ì╨  q□{é#▒ )q□·Ä8ì5g─∩p⌡¼8ï}▌□τÉ┐'▌c═α╦▀c╖
Γ□sK╨dfPµ"╓h°ûa1│□₧4α]□q.e□├S□╝Wåσåσ╠bx╞≡

*╧□V1┐ß╖å∩▓Æ$QT@ó, Iö
ö$╩□IóPÉ(#□e□%ë▓□ÿ(H$:éHT!□¡□gà5√=@û□÷o°2□S~îÿ≥í□ÿ≥π─ö╡─öâë)δê)δ-
p=╨Æδ□'╫□-╣-h{□_ñδüû\□┤ΣzPM«□Zr=╨Æδüû\□□╣-h╓⌡@Éδüû\□FÆδA+╣-╘Éδ┴(r=□M«□ƒ
╫â1Σz0□î█□─iP□êñçén±┼□─┐I0ε├áII▒□½>¡·7╘%┼fT╙U╙Y
°⌡½╨

U'╥¬□Çe[└▓KY

□√□≥°UOc╜d┘□░∞≤¼□è]╟èα╫]╨ƒ⌐~╞J¬▌¬╫qU≥δ#─»eΓ╫a─»├┴»Qª!~╒□╣÷'r╒Ç\±□□╣~£
R>□~□D╛

□╟□#·2□╓ùa ·2□ën?It¢R₧Rû▒╝▓Fyüì#╞5□╤□öWòWÖO┘□óu□╥║êe=╨[╨[
WI▒6σñr□⌡▀Ç\mΣ⌡╨¿ⁿVy□,√₧≥-T·>°╓□╟½\T■/*□T■□ò^8f≥â░+ ¡\C.]!£╧_ò□╚Ñ7ä[·P╣ï\
:DXö{╩G╠L>□V╡J¡
ùn□N╡V¡E.=#¼Σ□aW≈S≈CÑ?╕9D─▄L─□'b-½nT¢Pù▄□R;└═a╡□▄□"nÄ¿²j?≥á□ô□□:╬b`Φ□≥┤:=
éΩ

H:D$□UτA╥!uI]┬■ÆñC─╨ƒ"ån#å■□1t□╤≤□ps7╕·y░≥□bσ:bσí─╧I═n░r□¼|êσ4┐╥□c%"µa□8Y
h╔╔BÉôE
9Yî!å~ê□║H«□¡D╥iΓf□□│Äê┘@¼¼#V«╙^╘^□□_╥■□□╔╟âëÅzÇÅδêÅδ╡7╡7íÆÇG□□δ- α□D└□τ
`□▒»Ä╪╖₧□w□╤¡ε□«¡'û□A□½#è¡#è□□r
ßΩ?Öu□╤j?₧α¼L≥$VJf□A┤ZaS□±¿Ä□⌠c─á□=└á'□¡%□□L

ZG

ZO¼Y╧ù≤σ ╫g°3,A¼Ö&╛╠≥n▐ì║Σ╦□Γ╦"_╧╪│ßDû
╛□dÖ%▓□JdÖπ¢·VV□_nGE2σ├Dô9╛ï∩┬]Æ)□─ö□â)≈α▐ƒâ,ç□Y&ë,sⁿ?·!∞≡+■+¼□ô┐ë⌡
Æ,ç□Y&ë,sDû├°I~□;H╛,□_&ê/s─ù□Γ╦ß─ù
ⁿ]■.«J▓╜╧öW·uT$Y&ë,╙Dû□≤{ⁿ-╦□Sfë)s`╩!╚%M□ê&ï:¢╬┼J─ö├ê)!ª,□A□ë -
!é□F□9Tù╥ÑáÆ ç□A

╙òt%∞)²V□·¡h╓oEÉ▀è ┐□φ□▌Qú╓oEK~+Z]¢«
╧.]W┤Σ║"╙uÑò\Wj╙ue

╣«

!╫ò!Σ║ó%╫□−╣«h╔uEÉδJ═□«+é\W⌠Σ║"╚ue□╣«h╔uEÉδè÷□╫□−╣«□r]╤ÆδJ
╣«

!╫□-╣«□r]□≥Çδèû\W□╣«î!╫□-╣«h-
p]╤ÆδJ5╣«□r]╤Æδ╩ÿ□\W┤Σ║"╚uEK«+é\W┤Σ║ó%╫□A«+Zr]□I«+¡Σ║RC«+ú╚ue4╣«|é\W╞Éδ╩□
r]╤ÆδJ+╣«î&╫ò1□╕«h╔ue□╣«h1□ÇbAⁿ.V$╛/Θ=z□╥ü≥╜,½□Φ□,⌐□ΩC,□Γ□ú-╒G{╣?íÅΘπl8╤□
Bƒ╘°ír□□ª╧Θs╪°¼/C[⌡□AGΘ?ü▌╞Φ?ë5c⌡c13<îy
° î■3¿╥yáá ╝■≤x%S⌡S▒╛ΓM%'äaÿ□fΓY*□┬W⌠s▒├c·╟p╫W⌡_ee²╫⌠_Cσ¢·╟±·σ£Éª┘`(yY%hB
╚ΩWΦW@σ£0£µä¼■9=·□═ Ü□r·□⌡/ó≥Æ■%<╗£□å╤┤≡ê■╟·¡╕K╬

$9^2$+ ·W░µ$U^2$

¿£J·¢·¢╪A╬□i²ç·□Yüµççi~(╥ⁿÉ¡╥WΘYéµçtUuU5r□µçl╒Ç¬□X/°êa4Eöiè□^UWUç□cHU=V

┼,æñ)bhò╡╩╚╩Jÿ"┌Y□Ü□ ·cfÿ└□UO─Σ0¿zR⌡$T╛X²Eû»₧Y=□:½z□tv⌡lΦ£Ω9╨y╒≤á╥a╟H

;Fr╪□H

;□╔a╟H

;FÜ@44c|▓_c?;K⌡□▌∩S,▀oZ┐N6«╫
LN□jL□□ªíY"@│äOⁿ;=□ !fétσⁿ`ú╓!Ç╓ß╥╙τê»Çα;D□*rfpêoêoá≥M±8h^╬
.Ü□□4'°0',CσiL
>Ü□<Γ;Γ;X/τäÇxNtπΩ≤ÿ□<ÿ□^└nrNp╤£`ú┴A□BHⁿ@ⁿ□·Æx
*'ä8M□c┼Å1!D1!lC²□▒¥EhBê╥ä□ú !Ä
ß°¿∞□?cA▒[∞╞╩ƒïƒú.τä░╪ç9!$÷ï²╕z□□Bäfâ8═□c┼Q±□«-□╟Qù□BL╝-
▐╞J9□─┼o┼□╘□ç┘ å┘α]∞v

□éÖ&äê8/╬πyσ£╨LsBXⁿAÇ╡╙≤╙O>j^qE\EE·Y┼5q□╣tArÆ
Æò\ÉⁿΣéd%□$
·¿Ö┼?─?á╥□╔/>□ 1≥E▓□ÉAbΣÄd!O53y$5□⌠□=rΘöΣ$°$?9½y
FC□╘ÑkÆ╙0╙0□□Θ¥Σ&∩$ïí▐╨Ç½╥A╔O

JNrPrôâÆ▌Ç/\ò>JN≥Q▓ÆÅÆ▌0╙0□≤Å£ê\ÿê₧`&LD°<□╛m°6≤`"ZÄ║£éb4 î┼ⁿ≤□≥n├□□í)(fXk
Xï\·19╔Å⌐æⁿÿⁿΣ╟Σ&?&g┼¡
3è`k┴╘ï┴A□µäδ¬┐2ªz_uï)¬;¬□ÖF⌡æób\╤*£UaΦ□¼ƒRú

`□e░R□:oP□┘□┼«8@τ^┼╟□+▀W╛╧å¿G⌐?

╥j ╒>─□┤s╡Å1ô÷
φ□╠b─□k2ZìƒdVπ8π$6╓°yπô∞│╞gì»│E╞#╞½∞'╞k╞[ÿx□÷)Li≥▀l□1°UaΓj╟d6₧Me□c

{ÜMb╧░□l1&¼╖┘□LI┐╟¼⌠□U5{G%0σ|ñΩ»□¼Ra╨±ó═c▓Q
QMT=¬2⌐■C╡DσW-U¡VìR¡Q}≤╬╧T┐V}V²è·□U□╪÷½¬»i║4ïT_╫,╒<¡ZáyV≤¼¬
£√éΩ pεK¬┼ ▄□¬°@╕?W-
╫╝«y]╡□ä√ªΩY·≈é½A╕o½₧□ß₧W╜á╢ñ·ôj¥µ/Ü┐¿6Çs?P²@r«jôvÉvÉΩG┌╖╡≈T[@¥NU□≈pÅΩ&(2
¼zlÿU}( □ªΩ#>éÅT4╝ò□□`·0ƒñ□·□°4┼─°≤╢è□l╕P
±°°□%┼W≥uJü┐╙7+úσ?!T┌°v■û≥□?╬Å+_□s¥Vµ≥│ⁿ¼≥
~₧ƒW□Ç°«(▀ö$Ñ<┴ ╞o*K@R≈öÑÇ_â≥,╕i░≥óné╬í╜□VjQvΦåΘf)□tÅΘV)Wu╧Θ₧SK□X°6αT▀«-$
¥"╒Ct»Θ÷¿M║╜║7╘□yÆ½▌8├O½□║3║KΩ4╬φ□╘├□╗S▌« [òM²{πç╞□55∞═╞□/!«"n
n#ε1f╥⌠=¬L╒x¼⌐╘Lu□□┬Ä≡"┬ê□"ï(#Z□c□mê ê╔êiêÖL1═í`ª

□┼┤□▒□·R─□─j─Z─□─f─6─╬▐τ▐â╪Å8⌠└π╤□■ ë▐╜N!╬". .?≡x
q□q°≈-<Ü±±6s□>τµüê·╛G┼líPÖ¥□?≥h_¡□ID╛Æπy□≤≡▐·¿▐□█□φêëê)êΘêY╜kτ╥zf₧ÅXêXîXå
XI»½▓╢¢╓l≤:─F─□─v─.─▐▐τ;Çⁿ0Γ□Γ$Γ4Γ□Γb∩⌡+╜q□╡[ê╗LeQ□·╛∩□ì□Qïh@X□nD□□½╝∩û4ó
╪√8≥ü╟√δGW>□⌠╕°≈╛─□╫╟!╞#&!ª"f
f ≤Q■ⁿ,≤□¥□<v!û<≡╕□▒¬∩Q▒¼Θ}▌δ+▀¢e□b+b╟ ▀úⁿl²ÅG|╛-╗+»â^╫²⌡≤■%÷!

÷>εδ²î■3□╦□╘Å#z*?□╦□─·□-±;g╣¬□╨8»▒|▒½qIπr╥Uñkáδ□7A╖6εÇεn▄□=╪x□z╝▒□*∩:╙x╛±
Rπ╒╞□ì╖□∩Ö4ªjSì⌐╬d"╡≈σ^S□*»&LYS┘╘j□cjâV≥èN0M6M3=4=!φ°ù|üi□t⌐i□t╡i-
tâi3T▐╡═┤╚┤║╟■t╙t╘t┬t╨t□z┴t□z═t□*δw╟°-3
≤@s=╘bvÜ²µ¿9i╬¢ç¢GæÄ%mçN4OüN7╧é╬5╧ç.4/å.3»ävπ«uµìµ-
µφµ]µ╜µ□µ├µc╨ôµ╙µsµï╨+µδµ[µ╗□┼óç□-╡╨□≤-ï╒Γªz□i╨□│ñ-
E╦H╦h╦8╦°>¥dÖ□ò⌡□╝:█2╧╥iΘ┬U⌐K·≥σûU╨5û⌡╨Mû¡╨□û▌}║╧r╨r─r▄╥c9c9o╣d╣j╣a╣
ò╗▌½TÜ4M╒╨□⌐▓╥T╫dj▓7y¢┬ª

╥9}╡║¼'Ü▓Mσª╓ª1MmPÖOhÜ▄4
·╟ª9M□M
Ü□æ.φ╦W4¡å«m┌□▌▄┤
║│i□t□╙!╧ÅBOα▐SMg¢.4]n║╓t│ΘÄòY╣Uⁿ□□h¡çZ¼N½▀□╡&¡yδpδ(╥▒}y╖u"tèu║uûu«u~ƒ.┤.
å.│«┤v[╫Y7Æn!▌

[redacted] e [redacted]
= ` =

=f=    =m=□╜h╜□òw]╖ ▐▓▐╡)6╜═h½╡5╪¼6╖-hïA╚╢"⌐╟G┌FCσ╒q╢±╢Iµs╢┌╢□╨J^╤┘╢y╢N[ùm
Θ≥□╓W┘╓@╫█6A╖┌v@w█÷Aσ]□mGl╟m=╢3╢≤╢K╢½╢□╨█╢{╢Kvì╜□9Ω÷□{¥▌d╖█╜÷04a╧┌╥
÷V√□{¢}é}2Θ4╥Ö╨9÷

Φ□√"ΦR√□Φj√ZΦ□√f¿╝k¢}°}Å}┐²É²¿²ä²ö²,⌠é²▓²□ΘM√□□spçp

ä╓;,

N□gÑ ▌{ ╒∩ê:ÆÄ╝c╕cöc¼ú╜O':ª@e}z»╬r╟u╟w,t,&]╓ù»ttC╫96B╖8╢Cw9÷B□8

CÅ9N:N;╫9.:«8«;n9ε:□° ▌qWεµ4÷Vj¥
≈UV£V°█□t╞£°┘Iu□√rYΘ□φ□'┐/τx□°▄Θ£Σ£è|ås╣s₧│╙┘E║ñ/_ε\□]π\□▌Σ▄□▌ß▄
▌τ<□=Γ<

ò≈÷8╧8╧;/9»:o8o;∩╣4≈╒UMZπ¬s ▌╫_pÖ≡

u┘]^W╪ò
=÷σeW½╦k;Γ□cδq╡╣&⌠Θd╫4ΦL╫□Wçkük□Θ╥╛|àk5t¡k□t▏k□t°k□t┐δ□T▐u╘u┬u╩u╓u┴u┘u═u╙
u╟╤▄▄-á□IδI-n°¢╤U┐;ΩN‖≤╨ßñ2-
σ-δnwOtO1φwOw╧"¥√/·|≈BΦb≈2ΦJw7t¥{#□w╣╖╕╖╖w╣≈‖□╕□╗Å╣O‖OC╧╢/B»╕»Co╣∩‖Åy□Å █
ε1Æ╓z□<VÅ█□⌠─<iOæt$Θh¿█3

: ▌3    : ╒3□: █3ÅΩAO°°╥|□·r╧*╧□╧z╧&╧V╧

╧nΦ>╧AΦ□╧qOÅτ

⌠╝τÆτ*≥□₧█X□╧½±─╝ ╒▌□Σ▓╥) ╒[W╣Ω5yφ^»7∞M╕█╜Yo╣O[╜cá¿{█╝□╝ô╜╙z≤Ö▐9▐

∩□∩"╥Ñ}║┬╗□║╓╗□║┘╗
║╙╗□║▀{□z╘{┬{╧{╔{┴{□√\≤▌⌠▐±1h╪╟╜7}┬7╨□≤╒√,ñN¿¼t·ⁿ╛¿/Θ╥√å ï╧·(▀X_╗oóoèo║╜═
7╥7╫7▀╖╙╖□*≤e╨ò╚╗}δΣwΣ█H║Ñ/o⌡mw╖√v·÷B□°

CÅ·NBO√╬ß≤0╩w□9ε⌡╢·«°«7y}╖|w²è_∩7÷i-iâ▀□u√â■ÿ?φ/╜╧Ç□$ΘΦ√Ωτ-
□¥ΣƒΩƒßƒφƒ╫°¥■.Φ□ r * Ü╞N z & ╓╞.╒□■□■▌■}ªi■â■#╨π■₧╜
ε?#╧A y % UK° å ╢ÑS₧D■{□MáZ₧JüÜªÄ@¥5□0□∞□»5□□□╓╝ⁿ} d□σ@½½C~n□c□mü
ª²ü╒üi╨Öü9ò╧XáC■|□
□ï□≥g:3░┬█!▀ç└Ω└┌└□·₧□6╗█+▀i`[`'tO`┐√ÿ<q□ç□G□'¼▌▓≤□N□╬□.╪:□ù□╫á7□w*²9╚dù
≥á□

t╠□╓□-Ä·▓╧□¥A□0*{N0Θ£';I0-
□-□σ≥□╟□█]^·Σw□'□°8Æ┴Θ┴Y┴╣┴·┴à┴┼┴e┴òM¡┴εα:SMpcpï⌐5╕=╕+╕□k

`═ßα▒α╓αΘα9│?x1x┼═é╫â╖éwCJHo:□2åj¢.ç□B╓É;□

┼BΘP14╥|.4:4.4>4)454#4;□å　　uå║BKB╦C½BkBδC¢B[+╝□┌□┌□┌□:□:□:ND1!╘□:□:-
║□║*□□í□í█òô=t/¼▒╬□WçkáuaS╪-÷å├ßD8□.ç[├c┬mß
ß╔ßißÖß9ßÄ≡é≡ó≡╥≡è□╙ÜççW¢≤□v¬PJxmxCx≤}n

o3¥□∩

∩1ƒôƒì≡~╦ª≡í≡■≡╤≡ë≡⌐≡┘≡à≡σ≡┤≡M<╦□╣2┬"▄2:""□#⌡□K─ë▄-
ë6vEÆæ<txdöiZdlñ□:1□₧îLÅÇ'#s#≤-
¥æàæ┼╨eæòû½æε╙:Φ╞╙ûªÄ╙÷╙.Φ▐╙ü╙a≤└╙1│ê£î£Ä£ï\î\▒┌╨w
r=r+r╖≥┘v□ì*Q╜Ñ3jî╓Z≤╤å¿5ΩÄ□ú▒h:Zöä□□□□□□□□¥□¥*□/ó3ó│ú≤□∩IVÅvÆvEùDùW□<║èt
Θz╥M≥Yó[+□▌□▌m┌▌□=h:□=□=n□╤-3Å₧ë₧Å^Ω═»Æ▐É┐_╤█╤{≥¥ö<▄¼!¡û▄█\╙\╫lj«í▄NΩm

7',Θµls□<

*nnm-╙▄Vaαµ
ñôI°5iÜgZ«6╧iεÇ.É*⌐╡y□Θ╥µ□═½+ñ┌╝║yC≤µ&o≤╢µ¥P╘Q┘╙╜┐B¡═çHÅÆ₧É┐⌡=°H╧V┤
·B≤σª╤═╫Ü.4▀l╛□cMwb╝ΘfL─□╞Ω¡■ÿ%□□ì·cQk>ûîσ┴û°╣─åôÄèìì╡□□{∩─&║█cSb╙c│bsc≤c
QYî╩▓□~Γ▒ε╪║╪╞╪û╪÷╪«╪▐╪□OO∞p∞X∞d∞t∞\∞"ΩWb╫c┐bwπJ\7ó½S≈Ä╫╞□Γ╓╕;-⌠═≈°π▒x║╥
π┼°╚°Φ°┐°°°ñ°╘°î°∞°╝xg╝+╛D2Ç'□_Ä│⌐r╩P▀«£╤±U±5«│≥┤ì»ÅoÆ°m|k|ç°Gv¡°n∩
µ°╛°ε°┴°æ°±xOⁿîgkⁿ|ⁿR|Rσ\÷fπW▌φ±□±█Æ%Γ≈\□
MóZ₧Θë□╙■D]┬t ┤M╪□^y~%┬▐ÄD"æEÑ£hàÄI┤▌?)□□□ô□╙□3□sÉw$□$□%û&V$V╦∩.▒6▒
!▒╢╥i▌[□█□;▒╧₧□·dΓPΓhΓDΓTΓlΓB╝6q9q-
q3q°à╡≡□╤2░Ñ╛┼╥Γö∩[ï_ε╙□⌡∞hIz╢╢Σ[å[»╦-▐2¬ùvá-
cI█√¿&┘2æt□Θt╥Y≥5┤╟%¥▀▓░e▒{obO╦2≈▒ûòÆFZ║=·└Ωûu-
□+y╦□╥φ≥,h┘%╗n╦«û╝≥□ût╤rÇ⌠░$ç@G╦▒ûôü

Ö╖£&▌ ╒r«σó╫█rÑσ:ê□\╤r½σnR⌐PD`╡ ╘ñ^j╥vo6ëO□«╓zo&□Æ╓╧ëΦÉÜt'â╓XσöOªô┼ΣH_29:9

è:*πôô*°|r*Θ

╥┘≥£J╫ô┌▓¥≥╫dWr ╫n£α═kô╥ô½ⁿè<╟ôkÆδôô¢ⁿ▒Σ╓Σ

□╠}%╣□°≤∞Σ>£┼x╧ô□IÅ╙≈!⌐O-O÷°µ'╧$╧√òΣÑΣU≈▒□à&o$o██Æ≈RÜTu¬&Uù2Ñ∞)o*£Jñ▓⌐r¬5
5&╒ûÜÉÜl>ùÜûÜIkµ`MGjü¡'╡(╡4╡"╡:╡6╡!╡9╡-
╡3╡'╡?u(u4u□;£J¥M]H]N]K▌L▌I3√╡4Oï⌠└t}┌Ævª²Θ¿c~:Ö╬°ç°GÑ╟6]N╖°'ª°Tªâ⌠⌠⌠,□O╧
M╧O/LO□ ;╙ï╙╤╥+╙▌Θu≥TMoLo⌐pxz{z□Θ^Φü⌠ß⌠▒⌠╔⌠Θ⌠╣⌠┼⌠ò⌠⌡⌠¡⌠▌îÆ╤gîÖ┌LC╞ÜqgéÖX&
]Ö@φ□2E{[e╥⌐╠□ÖæÖ╤Öqò)/3>3 :53├~M₧⌡Ö┘Ä-
ÖyÖ┘«╢Lgª+│$│<}.│*│&}▌vòV«╧l╩l=∞╙∞╬∞½╟Y╓uÖâ╓ε╟æ╟q░DWª╟╓Ö9ô9oδ─≤^▓╖e«fn▄□÷
╟φ╞═╝□ÜÆ▓Ül5&&z=┘Ül□╘ö╡g□╖Y╟ò┘D6¢-
g[⌡cL√σ;Ém╤N╙N«░èφ`vZvªc~vN╢├σ═.╙.╩.=«╙«╬«¡╟â┘
┘═┘m┘¥┘=┘²Æs▓ç▓G│'0Sc▓╬₧"=¢┘É╝\Öù║╫ñ6W╙∞│à*7σ│d∩H═1╫Σ□╖⌡ΣDnáφR«>g┴╟ï·7τ╟·
s╤▐<IÜù╝ö□^y'σ⌠Ü□E:V╛¬\{nbnJ«¥≥Θñ│rss≤□╔▄╥▄bL»ÿas╤r+s▌òë5╖Ät#Θ□gmn╖πnnWn∩
}ò3f`ü╘▄ü▄ß▄▒╩\Ö;Ö;¥;τîσ.µ«@QGσzεVe╞╟╡ôN'¥%).wWj^!╒τì·ZLÄÿ≤
yk▐ì╔□Sd>ÿÅσ╙÷k·b~$tt~□&─╢ⁿx[Åⁿ╢Σ'æN╡╫σgΣgττσ;□<▀ò_Γ╝¥_₧_σ≥µ╫Σ╫[╖≤¢≥[]□▒-
·□ε÷ⁿεⁿ>oçGq]F~0□$>:□<▀ô?ô?ƒ┐Σm╥_═▀≡X≤╖≤≈L√∩kAô2□¬
5à:¿ë╘

⌡□┬àD![(□Z
c≥7@∩4╙┤║□╨≤
□□ô
╙□3
s□□à□àEàÑq╝∞ƒR╜mà□à╒à╡à
₧%à═àm╨¥₧«┬-O¼░┐p¿p┤póp¬p╢píp╡p¡p│p°╚è╝(╝^⌐┼ü▓O□δ{g+╥óÑêô║Φ≈╬)Fï╓b╛8╝8¬8╢
╪^£X£R£^£U£[£_\X\

¥U\V\Y∞.«+n$▌R▄^▄U▄
=□=\<□9

=□9,{i±t±\±b±J±z±V±nI)ΘK╞Rm⌐íd-╣K┴RLv╤R║T,ì,ì.ì+ìw╬+M*M-
═(═.═k≥û:K]~%▒°┤$U.-
/¡*¡⌐£PRKδ²ô□ôK¢ⁿôJ[K;*Σ□□_┌]┌W:X:R:^Ω)¥)¥╖╠.]▓tû«ûnönùεò5àD╣║\S«+¢╩÷╨±▓╖
□.'╩╛r╣▄Z-Sn+O(O.O+╧|p╖≥£rGy□tQyiyEyuymyCysy[ygyOy□·P· ▒wεq:W█ ▌ƒ
M≤<<≤╠s¢qïÖ$╫q I≤\H╥ΣH□N$╖ù□%
I╚╤äJ¿âSsΣ°IÄ$iÆ□çrKR!GÆΣLÄKI%ë1~{╝┐╧╟Ö╘y╒ ┐▀╤╛╥°⌡]{φ╡╫▐{φ╡≈≈9_█≥>╠
█ô╖?∩P▐æ╛□y°≥╬F╡¿□¡□⌡EC╤Z╤·╤å╤f╤╓╤÷è╞ó¥ú·╤-╤▐╤~╤┴╤┬Φ╙Φ╪∞²╤
╤)╤Θ╤Ö╤9╤ΓΦ┬ΦÆΦ≥$-
ë«ë«wg╤□ôó¢ú█ú;ú{ú□óÑ╤c╤ô╤╙╤▓ÿ□KëÑ╞ⁿ▒H¼N,'╓(û█vitf¼M┤8╓!ûêuëuï□─nÅ⌡Å
ë▌□□□□┼αHlR¼(÷x√U▒╛
F╟µ┼µ+║(╢4╢"╢*÷VφNè╛¡Φ╓╪Ä╪ε╪╛╪┴╪ß╪W▒∩._□;□+Å[±j±┤x ₧□┐<▐
▐$▐2▐.₧□∩T?7▐5▐═▐3▐7>≡╧╓±í±□╫¥ëÅÄÅÅOÄOì╧h║9■tⁿ□e□¡─□─□╟ù┼W╞W╟╪e-
èoîo╦i□ 0°}|O|□ⁿPâ¼°æ°ë□MΓ°Γg□4I¿sz┬I╘H°□
%|W6L╘J╘O4L4½╗╗ßφë╓ë÷ëXós"?╤#╤;╤/18Qÿ□Ö□¢ÿÉÿ□▌Öÿ«╝ñhbª{ΩO╟I□'□&û$û'J${I¼æ
,%▒>▒9°$æ\qÆc╘¬π▐T\┤:N$∩□╞╡≤9▒3▒7q@÷≈D⌐£┴□╟▄9Θ▐

q╖╨=q2q:Qµfb□iÄ)èªv⌠τ,q9ε╜J√╖│╧vî╚ΩΦX╟=⌡w╠Θ╪¿c«Σ?Wì╓⌠≥?Z╫(┌█-¬hup5pKpK┴┌□
≤]à[└inσ
╡'üG)Ük╧□_□-□╛□|#╡□)┌$⌐≤□tNÉ°╙▀n5□jw□╓
□Wmi÷8E═
┤xß%Eσ▀║W╘╛_╤ò╘zN┤¥□ƒ_ïµ╓≡çü[╕╘Ω@[≈ü;├w±Ñα8°Z≡□Ñτ
▒·ⁿA╗'÷wáÅ«ⁿU╚ⁿ□√[ísÇ<┼║|≤{ív]Θ╤à□α_
¥¬h3dÜK┐□]HÅ╞ü]□é╬é∩╓]N+miσ.╝□╟ôC░□¢æin^Ç▀□ⁿ□□VGª3ⁿß╪│

√⌐e■
╛

|□ÆMΦπφH~åµ╧h²2ZW2τ?É±: ü²0ⁿ8|WOk⌠

û>é¢üs¡v╨╓hå□m□ j╞Φj{□¡╖CFp
s-╡6#9□═°╙Z□▌□~□ⁿ□╓ε□K╩≤¿╒□■ldZí╙¡;E╤ª╨µI?╘çvä╢Ñ╓@∞╣
·åª□à÷

M7ƒ□¢ì┬
Càzj* ·¬Pδ:íµ¢p□=□□C░S&X¿┬╗┴╗æ╔F&□▄ç║}□ù_¡≡□[═□τj╗ƒ`┌Ω □s¢pî

p-▒òg¼║÷□┴.□KåCk`╓#╬yæq-□,Üì╤ó╟□ä₧═╘□ìⁿzΣâ┤≥□Oâ<
╥»╟í_╗2╥□≤kz□ö╛(╝□╓ld▓ßƒ□=┤□ñ]□═Oí┘D≤^√□╒ε□{ñ`8+░ΣYZYü□╩╨╢ù^öím/┌èd,¼τ□ï"
Ω□Qw"u &₧▒ ,╘°¢<╒╝"»3v║╫σêñ■gΣ7Bänе╘÷ ╒╝⌠±#í□K7□U2b╒sB□_┘ªùC╝2╗t/□

dt₧├åü┤°î╪`╘□□┤r8πi≈04ù-╒ªG╣⌠(W┤□╣h╙ÿ{7≥Tπ⌐╞ê⌠á«fR°5í□K-ìZ
□¢□ÖK├ÿK□⌐╖╨~L╡╡A"ò▒╨σê-;î╢║à╥wà┼□
╤Sb?½Σ╖H─3JÉ/A~□≥%╚ÅC╛D|eÅ├o%Γ+àw#ôìL6|Ñ┴■ôâ₧≥M╩¬□─BÅüàá┐.│σ
│ÑnÆ#│┼┤□╘□¥u□±¢░í□┤«X½ùè╡µ¡□τ⌡Rⁿ\╩╚>═╙s╟ 4╤¼ƒπΘ9<y

ÖsI□□1¼□à□┼î╤½x╛□Kèi≈%<ÉJ╗┼x □□□πüT<Pî□R±@1≤·%<P,+]aEφ╢.çQ+╞█}h±yZ∞#-Ü
äπ╔┤┴p≡┐τ~τ/é]Ä┤¿8cU¡ûh□áí%6√Ö?epⁿ.ç^äá□╨
?æΩ□f√Qⁿ┐

/□┼KGæ╜□/□eu/c=╓│▐S╕ƒPú-Üδ9╖)┌D¿╣□┌□N=τ□8╫Ç□é□é½ü½ü{ü{ügQwû`<│┼R│┼~B¿▒à╣▓
□¢□αd┴ΘnIDzM¿╤▌σ`├W╪╨□ⁿ□╕╖╙êU│Dß:Φ╧E█□┤σèî╒N¿■□ⁿδàΩßìÅ╨╙□:├òíG3ä*\
\
■
■
,-^/-Ω1╙Φæ┘□═nÅ□[ Tφ╓□j

v9XR
¢╤cM─?3D¢5□√;`□╛îéu9cæO▌|Ω□¿█╧n⌐≡
kà`zæ"Æ·Thè╥í/╙æ_□φ□'àQ√P¿┬2jî¥┬2j/╨╙□¼ÜMOS─6²□4□█«ª⌡□i²j┌ΩK╗çàj_╗□
Z\

5ÿW á/□O≈ß▒╡p>íεZ°kæ?à¥këä□ ╓╫▓ô╓g╬,┬╧⌡±╓□╝╘□=□\

^ZêPts□╥GàÑ┐□░|8#□aDå#?□·Gáç⌐5£Zç⌐5\<úpCp5p5p/p/pü┬▀□5å█}□■Q¿1╝ⁿCeτub¡╙□¢oá
Åσαi╠□w∩(□□Ü»K■¼jφf□Æ┘°oⁿPÄ»^╟µ»àΩ»Sδud□"≤`╥ƒóy(┤ƒδCk⌐┬□ä□□°ΩJ|U@▀
Φu□Φ4·^@▀°╤≈□·>ì╛□╨≈i⌠╜Ç╛7á∩□▓óPts□□?|Ç□≡vä□C┤□!▓u□∞$ÿ□□]Ä┤δ¼╖w)|3=Ω%-
*╬bàÖ!µ2wv╤k╞╚|□¢δ╙GFGaY│¥▌╒ç╠JPtsna¥vf¥■d▌├ê▄#□m?9ÜΓîå■S¿■ô]*X¿┬[┴[┴/â_□ùü
╥╨╨□
¡□3K├ó▀ë╙V□?w╡╒¿Ö╧IFmtÑ▌3╓▌è╙I¿~╞σ`├dl╪□=□=c∩□I[¥┼⌠òx╒┴W ▓ !XΩ┌s¥┌□g█°ò-
«□jd╙«ë
Sà□&·W ╡éBU□╖U░Pà_□┐

.□ùüwé≤□3╟·██□▐(Toäσ_ZΩ

bn□¬□Y.æΣKZíE²K┤íSaΘ╥f·≥Ñ╪i.t¡┼?
─?·Ñ∞_=xzä|▓Ö┤ó¥│╫¬°3Σäó¥s9Φ▀O_-é╬ü╛-c°ΣÑ_ôä*,²zT¿┬╥»çàjd□┌9kçh□jΦ╥èë╧
¥╛ñ╥εBiWOu9╙f░║□ª┼□Xr£^î°□╟±╞q√□í°φ╕╘Rkí
6╙*~D₧jτ<╜%□
U|u╛3;╪σΩΘe¼Ä¿î¥}éqî2ª┘αuîi6~nü
┘╥k½àP#¢yRåτ□!·□us¿╗□NÄ╥Aâ□□ä₧□⌠

BO

3d□₧╟aå

┬ô9h-ä'sÿu3□╟□δä┬≈      5r╧▀Wm▌┴Zxƒv∩áG-
╗ûï'ò═°ö╝&°-≤□Ve!OK]╟î═éb°>Å°;x:□□╧πΘ-
<□L»█░6Wc[□dåé_e□╓╡»PxîÑ╬╘:√à^╫□¡Στ□╒▌=bû▌□?▀ëUB╖πƒ╞.u=îg□πÖl<╙□╧dπÖ╞╡ÿMd
╪.╘zTⁿúùóm╡Pπ£P{âï□
µ│╙╥-»dl·eï~┼æ°│ç║□äΩ{ÆδN8o@K░-#╣Γ─╢

j═╡ ╘┌▀8-TƒK¡╣h╛□╓╣╟≤╙¼εm▓ó╒╟ \±s╤δhrFû<╢□╣─≤B╧:N╡Ω,»╬Ç┬
┬·┌σH╢¼0τ,ß□Mä¬╙ôHû╕T8Ω$"≥%Γg□^*µi1OSσ⌐s□|?¡√yΩτ

■□9╣║_$□nG╝- □□'□╒Σ╘P@¼□N╘:J□+□□□äo╘□¬╥~□│□è╝%≥v□δ-
µg□#8£╤□■$ß+▀ûΓ█Ró▄p░┤R□·z╫.▓▄¢╓
╙□wæ1┬!g~2Öwφ┬Æ]ΣTRw

t╕pT6r3╣D
╒εgB╒~╧«╧╙i≥╘▌/|²0¡É½¿□≡□≡.░hX&°-█╟┘°-τáy.f╛▌└┌╢°φ□

'l┐á,,□j□u║¬║QG╡╫║3¿╗ï║▀ú│X¿Ö)╡¼Äéu°µ¢.F█▀DÅ⌡╜P╡ï²]a»P}□Æ□æî░Γ÷╥╓n┌Ω├y┐ÜP▌
╧ë╗┤║ïÖ╜-
ß⌠é╙2y▓û^╝$╘£!╘9ΘΓΣΘxÄ╞:íµò╓≤∩▀σ⌠*╘l%╢9□Φu+Σƒñwy⌠⌠I8yh{ùv≤╩7½v┐└■╧░▀ì½M≡╧
+╘j□τ
⌠Lå≤å{╓öô¼¥╩Θ{□┌╥▄,Ä╙┘≤£╬>τ<δτñ∞Ag:□<"Θ£ò¥±□╬zPts□£%▓èSz□7Pts&□¿ë≈.╚╝╨zƒ╤□&k▄
- √ù╒Vó«≤╡╚x6êî≤?Φ╓□□O'æqRê╞frÄ^-
°]τjεàVú¡î▌╡□τ╟τ▄□□±¬□µ.σc°Ä{«╝0HΘ┤dÑñ£ô-y,┌z┌:(6[≤e▄╤0EV║╟╥y÷>µ╓OBì¼≥╥e□
=·─ì|óPSe╓yí□ç╣s
□□Ü□□Üé├α0▒H8≤à*,Æ/BKäút6□ç┴┬»+□δ&°u╤□□ⁿbΣSß□#ƒè■ÿ ╧;□┴°τ
=ÜE/ÿ≤╞¼≥·╥□fi□'KIPts.o-÷sε~ûsw wb[└~ε□-
p∩□¥½T[⌡à□àσç5╣□öô╫□BìGα╘ΓPtsΩ□ⁿòB⌡s┬╫7░□>╥«≈á{├y~ƒó□<W*■6τy┴Dò

σÆùÄ╞åA╪0□■hù□~□╕:æ_¿ëåìh╪ï╡eX[äåτ╨PD⌠.BO□w□□╣E)*┐□Z¥▌A┤σΓ▒nHµ╥ó□╓ÿu»íYK≥
ò╝╓¡á│ô█Æë╥ó3LZ4'Rk!6l└åà.ç║
Ñ«5╩╜Y┬≤□æ╣N░q
Æ╖êñ^╧╚[σ⌐^Jt-│╝╞îj.8Ñ□╕□ƒK[iH▓¢(z/┤:√Z'¿α>▄)y╪┐·αÖ>"ο.□
₧L∞_└²▐□,Θîµ□□╢─{╧π=?╢qw¬⌡╙□gëP²(¡-
Ñ°7ÉS]□╝S¿╤□∞τn╣)3í¥⌡ëΓ▄f?°j¥b▐6F▓.┘╦m∞┐╫□╟ΘI¡kôÖττóS¿□)╧&┐²æÿ»¿₧çå□"»τ□┘
╛s┌╨9┼æò≥□¡┤án6δ·□┴÷□8∩╕·EÅ╩□∩#Oô□lôP}└·sBσ⌐±
2□ô½Å!□□Er□sè%┘╬+─└├□□═╧□╡╧Æsjb¢╩mT|3╓H-
╗çD<°½p∞□z╛!√²3┘QXr9'n╦║╗ß|)°HyzëGzæσ!sû╕°r□9□■âZε⌐mnyÜód╘╞s«
□±<╧`τN╓ε╘·±□93&⌐{╬□╝*I9¢□6ªBsä¬╢*+║□╓╞B□ûò^█═9æq·╗┼'µ□xfwy□yè²-░□╕²-
°╙ο□ƒù▒╗L░╒-

°□²»□⌐ö╡;╔»vbëα▌BU.⌠2·Å£¬Nêç╒ëFΦ*┤1▓Ω£"π;□╬ÿΣh╨XΣ╦Θ╒▐!g%ô█-
≤fN□=8)▄╠ê£ß∞ -
g 3─┤y╪3ù≤╘BNa├ÖuCh≈°≤ÆS5)ù{î«¼□ç╒Σ╕ÿ8□a=^Ω╞1óP□Ogà□□jq□6&ó3[Ng╩c7╙¬□¬µ¡K
½ô=J▌πx≥□V▀qiE¥µΣ□°Qí□╤└î▓;□%½Å▓□ƒÉ|█î║□&■\G□₧-
≥╓¥╟ÑÅ9#□Γ≤R≡□─ûypv└ÖG╗≤£°8□]□>□-┴╣fü°£□τa$□áa

}╧&:e╥ïRrZ?τÜsX╕□{▄ÿ╢□
'╨Γ-εCN@▀@µDr╬□@E -
╬ƒ╘╙áPuói┼·σ□▓σV╟□┴qÅDÇW□╓□-┤¢(■rn¿╕╫2Rß┤çô□'├╛^Θⁿ▐║N░{÷æ╖║µ╝╓?□#3╤ε";á²╕
`87úτ□⌠ⁿ□|å█░äΩ[äc

σΘ]vB=}╚Z/□²∩ ║├\.□²=8╥ÉF]≈ì╟-i╤N╨Γ╚ñ¥▓
Åò{Q=ï█╤┌.F├□yj£é│□y□·δäcv□∞

s▒{ô╓╙àΦ·╟╜╜─╫├bòΘ□lƒw1≥}Φπ-zΣπFq□u}d¡k▒ Q∞ □╓:H╓óε□Y¢╓W╓+é▌╗A╗ì£5¼□E{c≤τ
n╛╨φb+Φ□n█fΓ╒Yh>HÄù'7o)9▄∞
ü│□

7r

∩□¡┐èà÷ô▄□

â≤ú╝□▓┐'Sσ═æ}□ƒ∞□□¢,┌ÖBεz□Ö¬√µt9w}W╤═ñ²□w⌡▄H□π╕!£Φ▐?â»uowë█Å 9╟╜!╝
Öp₧╪ƒÆ├mφ^╔δRVÆú╢µ╞r6wÿ=╨▄M▐ÿ[oZo▓é:*=cô∩D□h≥╛CΦ╦╔≈□□8²╔╗╣ì╔╗w┴ÅBç
Uû(¬ù#·o°□≡°┴▀─¿╡□¬o┬KchδNlÿÅ
w&▀╔6ßä%Æ[α╘πµ│∩□å≤~!┬─
(7╪ΩäXMd∞¢%gs▀°ÉOvù▀EX□m»`óεZ[┼=+W8·Z8□a╓σ│:«s▀□¥?óh□-
Ná┼,Γ[□▒7□■□°╣≡;□y╛ä~D^7î°â▒m"╢σc≤σ≡≤⌐ò╧M∩□nñ≤╦»âVτ□-1¡

2

ëBkëHk□σ[yú▒6╔ù╢╕7vΩsÅZ_Z▒□I+fS4

á▌□┤;└σ╨ïßΣÉ!≈N₧-MCª□Ü_□Ä±5-~¥╓_w╘o>µ╙▌╥δ₧╤α∩░JΩ

Ñn□q╕ □√√A#£σ-
Γ□>Bû╪QΣ¥□□ ▌î=□□╓ó┴└'φyâs□■2$ α╬m,┘┬╬2åh□w² └m·□hG╢╜w╓;ùóa□sπRµ╧² ╟τ√eo%≈╗□
┌ÿ==□▄_¿╩┴Σ6{╝ ╒[ßì╓□à7╢╕ⁿ;÷8╓4.ÉW4#□√Æ\╬DC-(┌⌠╫╚□f╢ÿ¼`□£?╨b&Æ□.&□5$□╪
m╚£oj?J╟□
╟Σ«╒¬╟╙cde'¡aè╙╧·@p2°▌AÅv░'¬|UoEnπf8▄ Φ¡╨╨Ωé▄□z└£╟⌡O┴Oæ-╘ □y□■

85ß∞Çc╪?(:□j≡Ä E≥dδAδ+%·╢ΣffT┤9âä¬▄á-Tz╘R▌~Z7≥

┤>}τ═ùÖ □>é□□⌠e□£ì╓-
□├┘Nµ┐ï□~;╜{ÅPts╛ëσà╓¥N¼}□z#ⁿO╙»□╤£%T;!gX╜^y=E╗a├q<▄ìVªé□
█\#╘╚$τ£çPts□ΣÑOÉ╙nBC-
8o└9"□m□9φ□Φe╚╞&°φ□°ådî* W;╛±▓P²╧X>Ç£╢'#Xô~⌡tw.lX'╘¿├╙N<¡π■~□□┐□ç¢□{□£□2·
}╚;°a≥╟╧╥╙mdî&╨»╣σ5ΣΣE/▄▀ƒ<ê╡φ░vâ¢-░□&
┌ùI]δ3ε╛╪Z≈╓¡Äⁿ┌Jτlóf⌡âè╙ìQkhφQ÷╪v▒┬≤▄¢%∞╣

{₧'≈{åÖ┘□ƒ□τ²°□í* ö1ÜO_&Ü▓π╧G├□VD)+«Ö£┌∞V▓□¥Åσ<bqbr ▌□ùè¥v

Oïäcτ\P½╞£Kn≡/╔
LεÉ┌~─Ö□□|$πe~#2÷â╙|#½▐□#Oφú÷X┴╚îa6╛Få?□=□Ç≤9'ú?$o
σ₧d□≈c╝;╙□ΣÄσ,╖m?&o█ñ⌡■óYe√jV[╛$û·ÉÄ□■"k▀H°ò┌2j÷tµgm8aÖ▒╬M╟dⁿ╧ÿ-
>Zⁿ╠┌»8{àΩƒ╤bsδ]┌JuíFsdVPδ>µ├□8_K╖*τù[┘╓╠«|F¡5O┐e□k├·□╬C╪┘ææro□≈aτ{X▓/yNù
Z╖í¡□£-─└ò2[╠╖-╡S□□¡òÆM╔oⁿ

▌¿□ K~æRl~"□╬╙╘|Ä

≈äµ□xαƒ¼▄²⌠e□}┘OÄ1╚ì≡få`í¬w[9ïëî√~ □£ÅXk⌡α/à≤¼ïi7Ä²━4#

τj½Hq-□j|+╘╤\╟╙□φ╫╘╙□Bì□╥%√▓╨╨□
▄Ü¼tg

▌½Ö╟H╖▓kH╕╦¡-D¡-é▌7D⌠σ$==KLδ
.óVwj±½3□┴Dτ□«╖ΘW7╞b'QΓ)°;▒≤N₧f≤⌠N<VÅ°9ò¼╡▒¼ }│-
□Çw□·LV┘°╠|╞┌Z├¢¼7┴╝ƒU3D₧.□╧ó°πßp"╔╦░≡□y{ef╚█+s-∩t▐M╛
□#°WδSr*ëƒs╕±n(Tƒô£üró┘H□┐e=▐µ┤PV═σµ≡%·σc~Ä□▀këm]Θ⌡╜på┬╔â3□NW≈}t≥╜░
─║FαÄDåm⌠▌┴₧R°{É_l╦j:ODj□□╖─@πî╠jò□<╚╛/°□╞nBfôⁿ.╝-√ßh5┤Zè▐═ù~=·I²□²¼~┴H
Km¢z¡ⁿ;□╒"·╖□î□╞+╞Z·╩¼±ü±æ▒╤╪m∞5>7╛Éo╟□▀╙waM▌4M█t╟□≥uW·₧½┘@■╜□│╜┘┴╟Sτ≈□kⁿ
ì□□¿Pòæ¬îMû
ò8G□¼₧ÄThé6E¢«═╘µh┼┌Bmë╢\+╤╓hδ╡═┌vm°╡W;áòj╟┤ô┌i¡L7⌠□=U≈δ□╜Ä₧ú7╥s⌡6è
█í≥ù┌╒ô┐┬╛ªΓ≈╫F!∩=╛w▀▒p#°,æ≥YÅ╝»π═å╩░ä╙╠9«╝╒□|□·α
<φ╩√äzr«rO└·□▓Θ₧£M{:╖j╓Sª╬┘K?

╟┼▀█r╓I╙S□°¢h╓▀S╕┐2p□Má╓ìαv÷□-·╢Y═M(□≈□∩"µ5╡⌠r≈m░P5₧>┼Q-QX┐ñP╤±ÜÑy□7ñe)-
_í5╥Üjφ┤÷Z□-
íu╘n╘n╙zi╝╡╛┌@mêvƒ6J{@□°M╘■ñⁿ?K¢╦╖₧□k/¬1xC{S[½╝úm╘╣i{╘(|ó}ª²K√╖÷¥÷╝vF√I+W
caΩûnδÄε╤½ΘùΩùΘi║ⁿ
$╡╒╪╘╒δΘ
⌠n≥»3Θ}⌡;⌠~z□}░~ù^¿▀ú▀»Å╓╥'Φ□⌡I·├·d╝Hƒ¬O╙╙-
╫gΩ╧Φ»Θ½⌠╫⌡╒·□}╝╛A▀¼o╤▀╒▀╙╖;/ªΣ╙ σ&ÑaJôöµ)╣)-
S┌ÑtHë╩≈)S·Ñ▄Ö2@~□»■7^9┬╤kΦ>=ñl⌐»7╘¢Θ¡⌡÷è╞⌠╬z╛▐Cò▐╩₧┴╧ûå·H}¼▓dè>]╡=G/╓□ΩK
⌠σz
¡oVt╖╛S▀½₧φ╒□ΦÑ·1╡bNδeåañ□⌐å g□"F□#╟hdΣV■ict0□Fù*□║□□╞φF□cêÆ²╧ƒ╖ìQè
Ä3&□E╞π╞∞╩?≤î·j=.σ╧□ÑoòΓ╒1▐RΦmc½B;╘║∞ó■▐g□4

□ôTτsìvjfL╖σ6¼Ñuù─Rδ□□▌&╪°╨Æ╙─JíµwûⁿRh╖HjçàΩC¿╒Y¿╜N¿σ@¢Xr
╫□m▌╤│D÷t│□z╧D╞ΘA▌εn╖Bì}╨ä□æ□e□⌐╟╜ΩΘ`≡h°=àÜ#Lë·â□k;àΩìx║O¿φGf□tÖ·äó□□□ûN_
ä?

è6{Ä█"⌠□t□tÑ9B▐É□5□□5₧□╫▒╒Ö╤(□¼ƒÆ_╜+;ù)~
ßh[Mò¢i√à□o _█*≥v'Ω□á-╤╜└╒&2Fè)7

>sòd┬µB°óßä<U6ê╠□Φ °9╨3X╒

:Ij□=ô╓╨φƒ%╘┌+╘∞

]åm5┤Çf□Ü╤D34=╡(uªè¡ □┬ +_!¼«Θiú4-
m£*ôT)REφ∞i│╒▀≤TÖ»╧"Uû*▐□╦¢65mF┌╙┐QDµÖ┤□iï╙ûÑ¡L[¥╢εó"╝ìi█╥>TeO┌~╧í┤#i'~úê
╠╒┤│^=δxkT)≥▀>o╚[Kò·▐å▐f▐╓▐÷┐QD&µφ∞═≈÷≡÷VÑƒw0Ñ╨;╥;Vò
▐)αΘ▐Ö▐9▐b∩B∩□∩roëwMò" ╜▐╗┘╗▐╗╙╗≈7╨□o):ÄyORN{╦|å/┼ùÜ,é²╛ê»Nò" ¥πkΣ╦
U%'Y┌°:°□┐úê\□_7_ü∩v_ ï╨□▀▌╛Q╛q╛I□ò"▀π╛┘U╨<▀ⁿ▀U□·û·V°V·▐≥╜²½e½o□e╖o-
σá∩≡∩*_·╛≤¥±ò ╗ÆnÑWKOK□ⁿ«Æò~yzâ⌠&öûΘφ(yΘ¥(]╚╗≤w╧⌠╛Θ□╙çªÅH-
¥>>}≥/╨╘⌠□ΘO°?≤¢eA·Γ⌠eΘ+╙W°»úlL▀û■a·₧ƒò²Θç~QÄñf°Y9ò~÷≈□┐µwⁿ5ⁿ╛_öÉ┐û┐╛┐ß┼σ
b_√¢·[ «╥▐≤w÷τ√{°{ jæg²ⁿâ²à■æα▒■
┐½L±O ╡╢âÄÖ¬╟±□√□·ùⁿ«▓▄_Γ_π__Y6√╖W□y╛Sò╝■□αR 1 I i□YåA[□òîöîT■÷gD~│
╘╓╙╓höæ√│·m2:ⁿ¼$2║ⁿót╤(╚╕=ú□╞Éî╗3Feîπ∩I□E┐f╧□+□Åg╟╫ÿù1 □eQ╞╥î□□½~Q▐╨x√gek
╞Ä╩╪^%□W╞╧dî╤┼¥▒»"□e□╟8\5ÄT╬æ¬πÜ□ôJ-
}ò±]Ñ═g2╩½┌$▒$`⌐xóµcáÜ;/□□δW¡½@@ò¼└σ2▀□
Ti□hY1ƒ□φ╘▀┐¥@^áSáká{ágáo```h`D`┤∞/ü±ü╔┬ù╛╔-□ÿ□ÿ!{IαΘ└3ü□ü┼üeüòü╒üuüìümü□
□{êφ¬╧ü²üCü#ü□□ƒ□°□gâZ╨ ╓□·$□KL□_□C┴Z□;â⌡âΦ
6
╢

╢□╞é¥â·┴─┴▐┴~┴┴┴┬α╚α╪α□±/>U╛□□°ñ-□N□╬ö},8Gφ?I?□ïâ
â
âKD□╧û□Kékéδe▀┌▄g½ÄQR'z+÷öΣ^ v╓▐□▄□▄-▄□▄□<□,¡□gΣ╒╪╓╪□Å□O□O□╦BF(%ö□≥
/□Q{°Γdæ²zπEσÉ╗/çΩ¿}Y÷b┘â+÷b_▓¿·C▀.▐c
▌□╩±.æ┬■X▒»&K¿æ≈ñö╩=RJ┼▐Xu»¼║GV∞ô╓□╧U√á∞à▓≈⌐²0╘╞w╖□µ¡∞sEn
u□%d^å║ä║à□B╖â√çåäε

ìb╬¬°□□□Ü□*□=╬│┘íyⁿ=?┤(┤4┤B╓mhUΦ-YO⌠δφ╨╓╨Ä╨ε╨╛╨A╓Er□T╞E□KCçC_I£
}τ▀^▒FBgBσ□╖ñ~e

╝xm]╜«Æ±ÑbmíC┼═░□□□«□N□□+Ω#»╓[8□╬□_-n□n□n□n□╬
w□w□╗ëI¬□ßεß₧ß╛ßü╘√¡□ö┤+<╘ìπòⁿÖUdÆ6K_□□Å+b╖─ßè≥▀b▌□ë°ß□╓┐G½°9Σ?σ□q▓j¼ö°X□
#½─CæEÅ╚¿gΓâ≡°î■ß╓ß⌐2╞ß□ß°Ñƒßg┬
┬ï├╤┬+├½à/1+╛.╝1╛-ⁿ!·ïÜw"□▐□▐OÄí≥Ä≡í≡□≥ □╚┬'┬°Dª"'□ƒìh□'RC÷ ê/□ÆX□⌐□⌐/z#
#═ñ╚□ì┤Ä┤Å─"¥#·æ-□ç#╜#²"â#à□C%^FFF╞RwBdJe╛$9O2GAWRç<ïLÅ╟î╟ë□ï]òy]27H»□â)□
9L2≈□]ΦX□Y□Y-)í^▓╛╙ôo╚□+□ß□Θ█Ü╚zxÆ7Vöè<▒j·□╣
╢UΣtU≤║è"·\E╣8°½╚╤~%7ïlv╤oσfΣ^U≤/╔╣Æy╫╧≥,▒U╒E&Θôè╡□┘─┘╓▀{#□"Ñæc╟W╔yÆδ*r2r
:RûiH╔L╔L═⌠gF2δdµd6╩╞═lô┘üÆ╚∞Æ┘-
│áΩ|╧╝=│?eHµ▌▓╛¬|⌡zvµ╜╟·┐╢▐Σ|É╢ê/`╦≈»σδ╫;*╓[µε╟}òX╛é]±%lUdφU|□¢┐╙▓□□k0++δ
r╛ê▌2½]σ·SδJ╛é-÷╚╖▒%f╓w≈d∩⌐(ÆS&┐è=Z·╟7▒°f═É╪%±C╛ï-
{Jà<_╚«≥mlÖÅ╓∩c╦╫▒σ█╪g%/É/cï-±_≥
┘╡$-╦°W~';·¡l±╣°«fτÜ·5{≡Ñl·N÷Hë▌5╟╓£Çⁿ□╛ö=G╛Æ-9á|)╗"6╦□│+÷ÑΣù│w╩yD╛¥-
gèÜÑU┐₧]+UⁿX╤_+"τ□┘╗·å╢╥!▀ öqû/h╦║¬ⁿè╢|=√?_╟.Æ²¥gÅ╫Ü-
kN░╪W│σ¢┘+j¡□█+╛û-c^±┼lΘW≥½┘σ≥Ñlëò1W┼╔┌i╡□▓WV|3[╛Å-
±«Γ□┘2w┼_|/╗gφ╛2fe.╘-Φ~/[ⁿ(W¼□┌±%ÆµB¥>BσK□è│é°!8∩┬·□|□°╧<}I╙¡O£EB╦σ
Ω'₧B·╜¢p£ûp╞≤t<╕=╕╜ïE╥«.□╗║p∞Ωpj┬⌐)□≤▀é═□╗X₧Ü╦α,C>îd╪┼╘¡□°□£□pΓpΓ┬1.□l\εb
yj╜ï=∩┬Y╟╚┼.µi?₧÷ú⌡■┤▛┼Xr»KßîµΘh·5üZ□□;σ°í□{:bIG□c[]8uß▄□╛╦┼<□
g$:cΦî±⌠!°□┴□□■├.╞₧¡X▓□½║┬9
τ4£╙hnïå╢hkKO▀D╧¢┬±Σ ÷Σ╡ÿ°Cx:äV□Ç□╕□²╗╩┐
²╗É┐-Öδ╤ $m=ë|□°mÉ|ÅZ∩┴ƒ□□□ÿQ░□□ô║&u═₧α₧╚□#S

▌□▐└¿Ñ1éi≡∩ü□□||kα[╥åo├/â_å■K╤□)ÿYd2ï¼

Σ3□═╬îfg°[αo□╧□╧□∩□∩□ùéK⌐█ä║M╨□-²τæ9Ç╠□p68□□∞7▒▀|□ⁿ
2╠□ï·o╡□╖B t⌠Oço┴╖¿√>u▀□□□■-·□╚╖@■FΣoDª□2╜αoå┐□ⁿ□°
░□∞┴çS±ßTW□ú□'O∩□Ä■ú`²G₧Ü≡Mj▒Ω
V╜·OZⁿ°+/□î√xzÆYα,$╤ló┘Öî²ô╤|□═g┴7üo□≈□≈F~□≥δ╨╣□¥{╤┘□¥═æ-
üⁿ□d╝╚x┴°┴└□╞□,<□▌□□□zê$&æ─f┼┘εè{ ■Kαmαm`µâΘ╬□µí╓<tjag-
p□□D's█fn█╘╡⌐k]╧□|□½╥≡ >╓°c╚<µ╬□8╠□{║;+α░ªlwM□m,7┌<□~□╠<▒▄y≥)·?┼□"|φFµo▒
[°≈íÖ▒|╓Pw
╛□î»□âë'å□O□"3Éh├X{□kg(}-
èN|bπ□≤□┌·□ⁿ6°m⌠▄Ä₧█┴â└âÉ9Ä╞q°⌐≡S□°□□;SXw)─├□Γís%φ^I╖├iw8╢í╟â-O7p7Σ[╥_j┘╘
▓·1"╟╤┐ ═¢¿σ°ûÖ├XrÿVΩQ╖-|,▒░─ⁿ□╤┐é▀ ~'°Å└□□~5°╒α|"LwEîw≈J÷ΓrF-
µ╞y7₧CgSw6≥wÇ∩@╧*⌠¼é╧εi▒{:wπÖ╕□A÷□â}╟$╩ÖD9√Q·≥(ⁿv≡█! 5≥_úôQ╢□e╧□°Σ□°□|â╣□
h½□■(°ú▄XA┐■Äµ┐╗sÆ°╡]J[ïhk□OYY□+╥`µ□╞□τ»Φ +°9≡s╙░π□εÄ╧«d╬rú□φ6gòmåz±!+▌bÑ
[Wü»□3,w>á▀B┐}3■╣□■N°;ik

m═í¡▒╪0□█fb█L0┘ïC÷bÄC~□ÿ∞╥t│»/╨·□ⁿƒα □fφ¢¼²□Γp□q8à□6à□╓~□█^Ñ-
|σα+□½╒π«╓ÑΦ_è<+╬f┼9╘u¿k-@f□°□≡;`□∞`□Q┼$¬ÿ≈α╔«<φ□g□O□Ç┘_L÷□╗>-
╓çO|0ë□□^╡\»^
╛□O▐è'oà▀□~_p□p□⌠∞F╧n≡A≡AΩ

úε00≥å+□□2WÇ╔gL≥□â=╫`╧╡^D ïα□└7`?╣èM«b┐□~□<□<□=î ª┴h□x╥└ôµ1┌:&X?!X?ü

æ┴ 2□╠4âÖf╜B╗»Ç┘â,÷ +□Äó┃□¥%αÅ┴âùâùc[clk

°?á°zzÇ√Ç√ CåoÆß;Φw╨∩É≤8Σ<₧?2ú■ê~f╡═¼╢▒▀╞~ç|█!▀╢Ní ÿ}▄b-
7┐í¡oα3.πbÅA╧d╚lM2[
¥û¢├│╥MV║A£7ê≤µ!°çαôWΣ÷kΦ|
=K╨│╟╚╩▓▄╝kx≥Σ╔6y▓·:▀»L÷Pô·i║≤│ê║E╘²u °ùαôk≡╔5╘%¢Dcôòh▓M|e║╛║ƒ
║≈úô¿h╗Q±s°ƒ#╧°Üî»ç╝╦├▌mwG╛;≥o! ≥∞π&√╕y-°Z0╣ôIεΣ╝î¥/π├B|X&ΩD]
?[°┘b
Z¼Aï╣g1≈

ⁿl╕~f=ZεzΣ|ap╛p╙-□▓Gô╢L┌▓╙æ,7┐ó/ª█ù╒≡Wâë!╓ ▓≈=αU-U▐≤■═²√▌!╡╢
Ed□□Sd╙□╙4ï)Fd□□æR□╙,MiLüª4
ö"Eñÿ"Fî)Ñ)╤╥□üÑ)ÑH1RJ)═▓□□Y∞ç,≥± e▒RΣCd)ï□≈ε9┐Ö{ss
5╡┤▀τ⌠¢y╫yOµ¥93sµ╟Öyß²╜Q1□τ=[¥≈≡4g¬°9<O┘Ωy□g*
g*⌡ªû8/.±¢Zß⌡─∩ □ojì□_□_Ω⌠╛╓41]╘t√¡¡ƒë╜b_°w╖Nè╖─o;╜┴e°▐┌Ωùz_δ╬½▐╙zΣ¬≈│^J╜
¥╡╫n░┐c?i/╖╖┌/┘?╢w┘'φ╖∞╖φ
ⁿ┤/▐ñQ╛)NS¢□⌐=█□Pk1úÅ╤ƒ° cêQ¿Æ8
*1å□#u□cî7&□ôì*c║QK=Ög,4□□ì╞2Ω├*c¥▒┴╪ll5vΦT
z╪c╝«╙Aπêq▄8i£1╤□ùLa║f╙╟&⌐ù┘╫□`µæöo□Ö├╟2sT* s¼9┴,7+═j¥j╟:s

ò¢Å~R┐PÄεáUjçF·s╥□î!fⁿMïπ□~½(J|9▐▒29╟|
·ì£o╗\Æ-))╟Xüⁿ□µ╫~µ÷□Σ£├[Ñτí°□·□╕ûuÆe╗

e¬┴GA □h;▀±^ò┘└_⌠┤Bⁿvò┘é£G⌡╗QîÄ┬□UΓm╝o5║π¥)q)φ¡½
α╒╓9╝□8□oÉq╬jò▀±╛òeB├□p⌡nW½╒ê·□ΦC#▐i┌@≥□╝o╡
o?□B.┤^d
(ƒα|#G┐üE\╝ü≈¡□áß"sk<ΩÄ└□R%£cΣ╪+┘

¿□├;VâPª□|□⌠¿┍7W≡[råy#┐+G½/¢V▐╟i┍□`┍±ÜK«┤ΘΓ^Z]Å╤╩ZG½Ω□¼*^S╓ò⌠û8E½G╥║□ç□Së
□Si□ô\'/□[aâ□╥▀±ªδ┍∩║┤uz▀⌡Ω7^═Ä╖^Ω┐▐Φ□>╨□L£ $x□'a─k□ⁿEíq
~█x{é▀÷¢─3`╛╬2òf╣□wG@~□≥!ö£╟▓□çåü╕√/¿u
e■□e□ ƒÉ_□9□w]╙≈@■□Jû@█ F╓S╕;□≥A╙┘¬o╙9-_ì□«¢□>□·q.┌▀o╝lÜÿ╖O■╧╗mε□ù
w█hσ°≥∞-╛╜½R╛»╙7╟Wµ□Ñ╙X·m□R·U┌╥Wφ½╤┌╬7ç╥|■
⌡□┌▐╫└□c₧ ╢╝╓╣□|5 =y¥6RÖ½S¢o□┍o≈φ╥i»N√┴□□□φ"¥≡¥≥¥MÑf▀àTJΩm╛*]÷¢~ƒ▀KÑ¿┐╖N²
«J□}█ⁿâu*≡□S*Ñ~f$ □ h 8 D□□Ñ)HS²3ê╧≥ùª╥\ é╟Dv)ó┌ï¿□KtZ¬╙r□
Ñ┌D^□i¡□╜□S*U°╖$ôoö╓╝▌┐3#φ÷∩úÜ□RΘ░ □╥·.F²ª ┤ *q┌%■ï□)ε┐□░□ΩGRè┌ⁿ@ƒ@□Γâ┤v
Jü!ü┬@I□ix`d`L`<ⁿÑ)0
#ª□ÿ□¿≥/□L□╘□f□µuΦI╙╕╨┐┤├ƒ□ï□ìüe:¡╨i□√w`□<╖:░!░Ö}!░ò}&░â²#≡Ü□g`□F{1
≡zα zt□┌ÅÉ╛F÷ö└q╛∩█□8□8├V
£gδ□.▒Ñâ"Φ□C┴∞`»`_▀░α□ ▒`₧∩h0?XΣ½

û□G·Üâc⌐□
┴ ┴≥`e░:X□¼
╬
╬□╓□⌐/┴ª`spepu░5╕1╪□▄□l°-WS/Å□waì5□≈□≈□□□Å□[Iπëα)╥┼k□#B╔□¼□□Q≡¼oN≡B
≡r╚⌠σà|ö□ü╩ò╤Z┌□≥H□□èåz□∩□□□□□*□□çJ▒ûτ¿□□□□═ú
ì
M

Uäªäª╥jσ╘□Ü□Ü□Ü
°ûB
H▀"^ìí%DKC╥C-í╡í⌡íM╛Sí-╛|íφX]┤≥B;C╗╡°-□φ
□□□

╚╨¢╛Ü╨i┐Θo ¥
]$+»┼├v8ûΣ»%■cßX╕O╕┐ \@åQä
ßü█)a«╕\áä=åτ*╠uåçGå╟°╖ç╟╙¥╞≡ñ≡ΣpUxz╕╓
<;dåτàå√╝pcxj¼»»ú┤>~î|Çs7ç╖åwP½»à≈ä_°t0|$0ê▌;->>C╛:╥╫>-
╛Σo±√⌐τz!/█δσ⌡⌡xy^╛⌐WΣk⌡åæ?▓G«≡╧╝Q▐╪@-
yhú╗7üⁿΣéooáûV\o»£ⁿ░─?├½⌠¬²â╝Z±Sⁿ^┼ë▐£`Ö7ƒ|╞≤]≡Ω)Ä╠αuφ5xM■-
^│╖╥[▄ε╡Æ¡╗a¡¢êN▐FJ
\┬?╪w╓kúHE≤í<XùΣC│Γ5°ï╜m■⌡^╗»┘█E·[¿£G=h±÷ÆΣ≤÷{ç¿ÅG╜▐)∩¼w┴╗î(X19F|╧_
ëzç"╜æ·Q£¢Ñb]¿wd ⁿà<╪;Ω_╟╤îx
JDè#Ñæ╛S▐~╣8v-°╥-öC=
ÅîîÄîú41RÖΓ?ÖÖAij(-èGfEµrL≥òEE╕▌%æÑí7#╦²#-æ╡æ⌡æMüAæ-
æφtggd7Θ┌99╠+6r,≥fΣ┤»)t1r.r1ù╢

DNK)c▓Å∞/□╔!üÿ,$*æ├σH9FÄùôé
r▓¼≥╡╥!ßæ╛úr║öæM■~í╙╕S+g╥y╛□╣P.

╥á╥oú·□╥▀°÷≈ô;(⌐w╜□╨#■)xOlP░LPtsß╖εΣq▀)²□7▐□≤╧Φxo[

ΩQµ█δ»□⌠τ╖│ëO _òD≤hµw□□{öSl▄Γ╖╨±&Ypö┐Ñ╟| é-òíY=Ω╙▀╪

î∞▒R.╘∩mW°╜½═oC°▐╤

╙□X|<ƒZφ1|b┤║─ƒí'Çruε□ⁿ%▓ïÉ□â▀┼w═]£oE≡t2ÿsî■Ékíaêα»]αLk□ä╡±9.cσA╢Qr□Σª°S⌠
4f
<ƒ! ⌐°□╩┴I█Φà□□ⁿ(Z≈!□k£□□□í3ä╗π╤½▒,╙□ƒH₧└¡□qzv▒7!°□c┘

9ƒ≤═e╙iU#ìπΘ□╡6╨≤□=ÑA▐é□Gs»¿¡(ú}p«>é╗╒¿;□e&π□▐Ç>ö
<┼╞_å
·«ç≥s!π

o¼e¥╞^⌡ä╟▓z╓░□ßεyò□=1≡<-ú}\≡Sr>╩ⁿ□╜¬Púï?₧zªÿ┬φRïⁿD█_═□□▀Y∞Å3Rh□4ÅΓ╗b=j⌡b┘
iFδ╣,ô5□Q│□cÖâ╢ⁿ<:├ï?ü□≈"Y┤ín□∞v□≈J=q□;┴oΓÖ5V¿Q╟üß╤3╬z□┴V5╚)└\

î╖ú╖{xÄX6╖C─□ε□╧gN}╪

╣║□┌₧□┬≤□Å□▀⌠ □^ú╛g
^ä–╓≤▄Ö–░▐v┴▀– ·□luF·□≥OÇ∩┬ⁿ«B¡
Φß1≡ß╟]°ñSà£8ù╖Ä&□¡╪'qÜ°□@gìjEì□╧â□⌡SíôzBl□╧bε+ΣZY≡"{1x□╧ö[─w¥_rO£qëK≡ü□└
7αëÅδ▐á·├2YÅm√vΓ–ⁿo╧2<
óu╪│□ⁿm□z8□9│a½□╪╢□rI╝ö¡□»FÖj▄¡╟XΩY ò▀!τ$l╦□Bα#8╟ⁿ□╛≈Q

;ƒA╬«°\╠□ctOa╛Äí╝□ⁿc±7ê;°╨╔Jxr4╛□m╒┬ç□0N

╜□Φßçπî.¡Nⁿæ5`î¢0j?√ñ1□kñ□□8╞φ□[ò7r□□>Iπj╟┐Ç▓⌡□ Γ□Wæ
δ┤□u╧≤]w#δ7

p

┼├Z<k│%│Q7½5¢≤φJ▄━
ë□□╟}│╛□▀₧─3%.±╪╓□□` /ó$Åelⁿùαoá┼<°9δy8■═╘ek╫▒5ê?Å║GQ≈$ⁿƒWAo╢□·<░|╕{Gⁿm╚î
─│aφ¥(│□σ╫*

¢¼└║x□wƒÇå[0óoú¡[╘□ Yâà·56úⁿï╨□7·∞╙qëπg ╓╥

.c┴ô¥⌡ê'□(3□ÿφ┘░¡ ·wî>≡vⁿ√I╓a╜_≡╨≥t□▐éqφA⌠fK.Cδτæ?ZGoσ½□0^µôQª═╛B≡wmjßƒl╧█
áa,╧┤ó□Sæ3□σ≈j^ï┘»à┼

₧╘"ålA,Zì■3Æ≡∩α{¡╠ì&°├^1₧·/□c╒┐□σ#□□s▒□Q4;▐êÿ·□G□∞\├□□vB╧Z╚□┴≈óⁿ□n╤Y□^□Åφ
ì¥«Æ╦ôÅ▒τ╗hσ░╣╧3ÿ±e╪□ƒA4□╪M□ê┤╧ Vsⁿ?□▐åænD£êu╫ê≥█U-┤Rà■£┬

ûΩΦ=

÷ßV^ü%¢╘(á╟╟·┤√≤╝ B╘□╔φ║KE-V⌠Å┌┴?□□[¼╦hσ□óY-
╓╫□Φ▀└φ╥è■#Ve□▒:è╨ò·2°φ^¼w□½├F∞¥î≥□□├□╤ô├╪╖δ□ƒO}fy9φ▒╝/│5zaàÜ¼▀¬Ç²╖ó╓□1□τ
ƒ¢□▒·ï-%╨╝□e╬├

≈ón╜ε?|@E□╜τ≥■nX`■

2=ú|□÷)┬Φ

─□Ä▌ j□<φeî¥┐b6ü╣s□□├f
4,├Z«AN)∞╢ÿ╡Q╘z□╢Γ·=    ▌□_Üç|ë□│□₧0ùe±ƒ8-∞C╬<°@□ΘÜ═H╫≡╬≡Å □Θ·▀Φ_□Θ╘-'
       CNªk□]°█=°□°[4¥¿ûh÷5h₧ªàÜ□wô□ëûuA+4¡"Z╫M┌@┤Y╙VM;4╜ª»{ê^':Htñ
:Nt▓□tFδ8»ΘÆ¼Ω!2╙φéB□ö².¿□Q▀.h□Q^□öƒAE▌ñaDeDú«Ac5M╨T▐M¬$¬~□¬!¬δ&═!Ü»⌐^Sâª
&}m&ZI┤Ü¿╡
┌H╘╓
┌F╘N┤K╙^ó²□t¿
:ÜA'▐□¥":█□] ║▄□e╪:█∞&·ê<óΦ5ê∩⌡&Ω°σü▌ñ┴]√

t0□□□wôJëF0eÅ╞u\□%╦L╘╫□ó)DS╙┌J°□·:½□4ùhAF²E□┤ñ
ZJ┤£¿àh¡╛«┐F□«E¢ê╢tA█ëvvA╗3h_Z∞NÅ╖╔X⌐πX÷□Öè/┘çe°°æ≥æ⌠yM┌;iúci}~│sƒR±$▌7ôδ
W»¡∞╙□>ƒ}«│Oú¥ïDqYòc□□êñ┌_rb*có="°Å┌Kr·□
"□BTHTB4\╟v□o╬Hó1□±9g<╤$"▓□╟bÄΘlç£*ëxÖ3]Θ═í}/ç÷╕□┌▀r╕mj3ç█Zªφ½φ╔uyƒ╞Yí≈░¡
□vå«UJ□▀╦í:9□t┐2τ)cÄR√ë₧'╓┼{c

φs9╘F╬Ä┤ ·'╒▄±∩9┤τσ╨~ùC√]╬A]ª6ìµuAÖ√≥è.h]┌■Ü╛╟&Θ`□eε»z┐|O√d/┘y/□
;÷└┤².τê≥╦□┌╙sNjÖµ8τ╝÷Wè-
9┤oGà·=ΩΩ+╡□═Vδ6┌K¡'-Wö┌èR;╤Σ┌H«âd\ñX□═╫qn\╟□ë□⌐°┼⌡S10sme«½d|╤k
:<σ ╤a¬Å⌐·│╘zï╥₧□%²Q┌¢ú┤/G╦U┐□ôh

Q┌□ú╒║▐ƒè?□q╜╥2╔>w□Åô45ì«╒╓ƒè°-
□ö□'╙cσ÷┤□Ö-□=]wï╛╟6á□□¡Qs□¡Sπî╥╣"Jgèh╜╧τÿ□Ñ┃Dö╧□ò╨∩╕lö╧□|╞αr+⌡yéΓYt╡÷M}&
êR~tú┌ úm*╬E╖i╜φèxìFΘ\□ÑsEt┐è├Q«{T╟OnO╟╚(ƒ
ÆτÑ·Ä8□]Z□▀ï╥Y!zA≈+3

g─α╘□&┘F╗╓A18╫╘uÆ⌡█t|.╥{zâ□[«OτòÑ╤╪.¿;g┴:┘qªK?╫%iu□e₧ΘÆg┤≈r6;%;ƒ┐.╙ÄsW·^V
¡δ₧φ░Irmσj ╟Ñ3Tno┘qµ╤δ*ù╬S╣□5╤□╦Ñ{╣┤^rΘ¼ô;B╙hóq¥²=wóª□╡┘rΘ¼ôKk+ù╬5╣│║^o□□
sΘ,ôKgÖ\Zc╣K:╓[ε╥4y╣ª□E╜÷rΘⁿÆ╗^_7u¼┴\ZG╣┤╬rwª¡?Æswδ■∞S1+≈Ç┌{Æ─g╩\≥∩▄cz╠tf
╙=¡b□║Å▄sjOIû╧Ñ²?ù÷·□φ ▒Ç≥╟□┼└□φ 1┌¢c²╒╢ 6HΘa√┼h∩Å□¬x╟≤-
ú3@l╕ªæ╨µl╗□¥□bt□ê╤□ 6Y┼εXò.O{lî÷╙□φ√|□î═δê═1:□$≈Ñ□φò1┌+·y$F√%?S─hÅî╤^-
ú}2╢A┘1F{5?Å≡▐□█¬t─v¿yÄ╝ª╓UîΓ@î÷Ω□ì;F{Wî÷¡╪I╡┐π▐□╡µ s┐i-
ï╤■╒S¿┘≈ñ²½gH═yO┌┐z÷RπΩI√NO≥ü₧y*&ñb.=_O╜W÷ñ╢ΩI√J╧2□∩zR;=╟*▀e{▒▄sé÷g≥à₧Σ =
+ò□àæ╕└□k]□ÿô<□2qàQ□□o
□¥¬q⌐x_]úPü<╒ÿSα∞4┬TíJüàQ□Rà□UêQà□╒°╨□└±■░┬ü*□¿╞~*╝°Bz*╝□p¥□╤⌐░£□┐⌐□d□
ä7╪□*S#1±n╣┬]*¼ÑBY*|ÑBVjL%z¿qö@{i∞$É□□/⌐ÉÆ□#⌐╨æ□□⌐□æ□╗BA*ⁿúF>*╟úB;□□ñ□Ä□█
¿Qì└3j$#0î□╜□▄óB,*¼óF)□ƒ¿Éë□ô¿╨ê□ç¿□ê└─*╘íB□*îíB□*\í~O□oåk,!0G□?¿Éâ□3¿╨é└
*äá┬□*Tá┬□j$á┬□₧□□εO!■□╓Oúⁿ□┘O┘□6╘h>α°4éOa≈□:O!≥╩(□P╙;e□
□□╬N!∞□îEíΩ4₧N·□0t□=°ps□1°▒r@╓i|□Éq□□°╨p>╟öB└)∞¢B╝i╝□Én□▌ª░l□┼ª≡k□╣ª0k□¡ª
pj□íª░i□òª▒f@Ö)|ÖBû)LÖBô)□ÖBÉ)∞ÿBì)╝ÿBè)îÿBç)\ÿ┬é)ⁿùB~)╟ùB{i£ù┬┼¿u¬╓à┬m)─
û┬j)∞ëB□□ÖÑ0Y□iÑqXX√□{Ñ0∩└[iñ□fSú½□ΩYíó╨□àóR°)à£Rÿ)iûR8)
ñ□6Jßí4□□▐«╨O□≈ñ□O└:)öô╞7□⌐ñ1M@3i□ô╧W╪%D
àW╥H%`ö4:IßÆÇH╥X$⌠PßÅ□≥HcÄ□«Mßî□┬□╪"ì*Rx"á9□åHíç4n□ê!à□╥(!─
à

╥ÿ à╦Sÿ□à□┬□U¿à≈QH-
à±QΦ-àδQê-àσQ(-à▀╤╚□□yÇ╓Q8□ì╨Q╪□eOαq□□Gap□·Fπn□ΓF⌡□(□à»Q╚□à⌐Qh□àúQ□□ì¥Q¿□
─□àö╤□□°¬╞┼(D

¼°P0□ óÉ/□≤ó╨.□τó□.□█ó<DE-╡□⌐=□1Gä─:■??+.L±!π¼±{□òo□□äi\2■(l#a╥&i:ª+|f╨

ëÇ)═-"dµÜ1ßÖ╝═□ä4√¢□┘µ ≤□"j>e>%b╓(δ≤ó°Sτ╞□-
≥·y7ë□╝í▐□±I∩no¬╕╒½±-□ƒ≤fy_□_≡µ{▀□_÷Ω╝G┼m▐c▐╦Γv∩□oçXαφ÷~+□zg╝
╘┐ □{÷□!∞╛D□ê≥ê≥ëèêåÑ]╦⌠u□╤Xó DσDòD╒D5DuDsêµπ[□┬n
j"j&ZI┤ZS+╤Fó6ómDφD╖ê÷□φ╫m□":Jt"φz*φ≈│D□ê.
ßÉ □_┌╒#è□⌡Vσ·Ω⌠#□H4ÿ¿Ç¿8φZJ4éh4╤8óë║|□╤□ó⌐D3êf□═%Z@┤êh ╤RóσD-
Dkë╓π
#┬┘B┤¥h'╤nó}D□╘╕£├D╟⌠⌡═┤k▓ⁿieS\□Θz╒i≈╧□]$èôδ╪D□"┘qe√╕1ó>i╫■Dâ╥«Cê□;«▄g╖äh
8╤Hó1∩ε╨s╓ΘJ~ΓÄWφú?╓≥▒

ÜD4Y_'Θ╣O#╖èh║▓╖[K4;φ:Åhí°ü╡╟z▌:h□▒Ä['A│¡3╓yδÆ-l╫

┘┘v/╗»5▌-`τ┘·væ=╟.│GQ□kO░╦φJ╗┌«▒δ∞9÷|╗▐n░¢@═÷Jⁿ╛┌n╡7┌mámv╗╜╦▐K□≈ô┤║C÷Q√□Φö
}╓╛`_vL╟τxN╘Θm╫;²£ü╬`°└)vJ¥□╬hg£3╤⌐pª8Sφ6gå3╦Öδ,p□9K£Ñ╬r°┼Yδ¼w6□mA¥φ╬Ng╗│
╧9α□vÄ9o:°¥sáïN▄┤Ö▄Ç+A1╖□¿┐;╚□Bτ≤B¥JR□╦%εp¥FR□π□╗π)┐ÅNô▄╓Dô▄*J╙)╒║│▌yt□!h
1 σ^²╒·rgzΩ█⌡□╚àÉ
!□┌_│╤£Vê■▓}╝└y2⌡¡√□ö╝╔╛□≥□Pk0±!Z█m╨6ƒ∩ó|à=ê╣3-_µÖKrö□^±¿≤
±═(≤4╫╝□·╨+╨│□·≈¬^Θ╛òB≤L╚úÉ»Σ0Σ╧B■4Σ╗I╧□▄├+╟°∩eQoK1"U■Fö╢□╝²□t▐
·□╙╨╨≤╧btSQùσ¢¼□r>    ·
h□Γε(Σ▀□═Φ□ ╒DÆ#ÉoEÖ!h╝□¡D╨╨╧¡É?
Yò/B·jΓ·É≤!□≡╙"±"h@

°PΣ■

+▌∞▄ïVJPåσí╓r╘┌àÆ│áy5ΣUÉ≈A^□y;≈!>□σKæ│□w?□m¬V=±┐□è╣□j□

n□ö»Bªü┐─₧µ□bOs▀fOâ□Ç|□Σ¢ □║çæ┃□9□ □ï√αnÇ|□≥□▄σ·-╚Γ■

AN!┼g╓≡o(c├╗~╓▄╜╚>û┼zó.≈0Ü╒╠>╞□=>■tV+√□╥W^a9╛╨╕┴>û⌡;-EVO≡ï╟▌_│~%≤▀B$·□╚ç
d=
╣

≥T÷4τ'∞ijîε .∩╥f9+ÇÆù╤█í╨s□╨⌠╞]xNV1°0ΩF▄█

·"≥□"g°□pÄ∩»ás□ZW½π□d□e╛ =/└J□╨óë╓┐
·E╘e?╓W£√O6<╞·>□2╝Hτ□á□òÉoü|□4└ }□⌠□□²)P²qùí?;┘0Ω¢⌡¿çB≤](s□╧□□r1x□j▌□□½8
₧∞b╜Y_┬(╕òBü₧└oç║

εNélúò
¢|
9Cq≈'∞╜αC|,pσ·┐□ □J&É□□·╝
}

αDJkM┤□3ê▀Φît■╓·g»»Wα}╬□Θ} ▌□φ}┴□π}╤□τ}┘□∩▌ µM≡n≈&z□τM≥╛Γò{wx□ ▌¥^Ñwù7┼¢µM≈ε
⌡Ω╝Ö ▌\∩ao₧≈uoí≈Mo╖≈¬w·}╘|#┐,¿)Dö¡⌐ùª╛i2□¥□ì<-s╣|-□i□ªëvtâN□□¥
:A□σ║lÑ.╧y╒i┐╫Φk¥ª9im╬╫┐╫SoÅP:.Nè3Γ<ú╫
ü╘·/lΣÑ■╩å·□□}ì F9IòΓ<òσ┐┤Qù÷ù6µ□M<» Z▒┬Jⁿ□≥≤∞%α□ëg
□□|1»□}≈Yp─╛─\╚è≈□□

·¬εF╚□ím□°»É≤+╚êw╚–
è┐Kk]9□·f≡ë|≈□╟Φé+?çⁿ□ÿ╟°Çù#τ$ù╝╥╞}╙·□éc∩ëτBO.Σç┴oF¡m¿⌡∩É□é|¼┤+Oú¡ùÉ 3ûπ&
4 vΦ>£CÖú¿EkO4□G□1▄≡î¡ö┌)φ6

ÜK¡ú╔i█uå[°¡╙╬(:□ìr¬ΦYf.Ñàε□δ4¥O╞╗σn╣3▄□╫╡°Ω╘┌▓t╖£Ωσ║*Q.δαTσ╟M╒-
ƒ╥RG=□n▌nM▓┘b▌a▌i╒YO╨
≥nY%°╨□yf£!∩□▐Σ!9G~M╬ò_ù≤σ#ríⁿª¼ùÅ╨o╔┼≥█▓A~G~W6╨&╣T>.ƒÉ═≥)┘"ƒû½Σ│r╝
|NnÉ╧╥ì≥□rôⁿílô/╨ùΣ6·▓▄._æ;Σ½▓]ε□}dUb_bƒ£₧°ΕΓ□≥^≡┌─Å□?Æ□%Ä'Ä╦9ëmëm≥δë'□O╨
o □□°┬─ª─&·M≡FöiéP₧╟q≈i╚½└ƒü╬ì╙ □°&≡-
B V╚█áτe≡φα»Ç∩□▀╓·áíz┴W1êÆ)°B├:µΓ#╓φë╨DÑ╝;±╙─OσW╤r5°4≡√└gÇ?□^□>□ⁿkαs┴-
□»□□□ⁿ1≡eαOÇ7â?à╢Z└┐Å£╒α╧é»□□

|3x□°Kα»é╖3□7╥)ë∩&╛K}τ╤V±Φ:ì`¬°¿°¿-╟⌠─∩□┐°ybPts>ª√»□┘L≡Yiú|Φ¬▒>

>∩□πnBO╘Φù^├□ ê₧»□□□²○□□□uWü°█f□╞≥

·□╡¥6Ç?□■BÜσ~ÿf┐üo╣å-Mδv□□□δ╔─!èU╢⌡Eε□·FYó╟¬âƒNÇ≈□╥¥╛ó/▌íZ╓$≡»@╧□⌠09
□□□h□ÿOE╬4╚≈ü╧□┐cy□r□°LΣ╟□ƒì£9" □dyûτé?î╖≤└AN=°úαì(ƒ┤?τ,E╓eÉƒ□o□∩lg╬Y
╛

9╧B^□■□°□Φy-ⁿ□≡═╙o□ □r╢Ç┐ä£W┴█┴wr»°▀∞xåΘZùx-±Ü░Φ≈ΘëΘ⌠█║─:▓▌╞öh□░Ω'ê□□-
v;l╬5g&fè~tÑÖ ½»L¼$½3»K£N£ª≥Q□%½s√╒α╚┤°3ƒí-═╝N[ù·╫└τj[2»□□□ⁿ1m9µOÇ7â□-
|╡÷Dµδ┴ƒ╙Va▐ª-
┴ⁿU≡vµ4╛:φ+}·o┬╤x~┼√ú-»ΘÄß□╞m±□D«╚□∩τÅâ╜¼╙OΓ□ë?╝k=┘ö╕.=□╞╨┼□εuτç╓_J

RΓ║d╡⌠·□│□┐Nⁿ□2»╘k⌠;±□¥Mt□╪gÉ(▌≥┐yM°ε±-└╫▀&~¢¬╟>╫╒8┐m≥τNy╙□╙║56■í9G¼╤·;Θ*
áöQ÷¬╛d÷ï|-z┤□R}:ÿ8ÿÆi5%▌Jⁿ&±¢Σ8«╒┼wÜ+°jw~x╡g╓σƒ3ë3┌▀⌐/Ö·□ù□ùS≥Ñ─%\iïïORb
█²+%è□<Å□□ m╟█)Q▄à]à÷ùñ]╡}«Ω╤;ì_█δ¥~p¬α½n│╙=:òd╢┼Q∩¥⌠"*╥□Æ⌡ô≤tU9÷▀╞ƒñƒ$╤░
/|üRµOW>¥QW|åR║.ÄXaJ]⌡σ╒─½]σ°~╥╫2ùτ╣╧╙K▌δ«_·ä□W÷»k╟m'╝□k□?]╓╖F┐║╘┌┼□t│_╛C
Ö.∩e¼╒kû╙6a]⌐t1q□y∞/□Ω╟□azB┐C□k▓u°░eÑ╝G┐rÜ£&<zJ¿□□·á£'b⌠l≡□╤ù₧□Ü┼`╡\«□Er¡
ⁿ'±□z

╪$>K° v±□╣ôv≡»É╞aⁿ╠!BV╣UN┌∩ φ-iƒL≥¥╘åGm▄E{┘□9EX⌠<r╖╙ógÆ*j√½▓Z°Σ=╘â□⌠
çzPC√5=½╨▌√σ²┬OO,╡tryÇzûC={É°l9¢≥Æ□Q_τ╨I╚Σ°□ß╚ç┌▀AΩ≈|æ-
┐!□ÉµGΣ#ñç₧päΣg□╤C.ÆïH□=δP+⌠┤C#

ì3Jπⁿ.±F:'Ö≥{≥{╘█&:-
┘≥1·□ΘY*ùRïÅ╥╟⌐'╥Σ2╥≡ä|éε6ôuzÉuVP·□ö+it⌠┤Dúúτ%║√}▓Ü□V╥!½=G9⌠⌠Dz6Æ□□d┴-
R █d□⌡ⁿGr
⌡ⁿE·"⌡|½▄J╡^Æ/Q·mr□⌡£₧½¿□╢╡I⌐□╢

└╓A╪:□[□aδ╨U╢v╙l¥EOéS⌐
╢uû£.∩Ñ-░┼{└ΓY░╕□□≈├ΓYrª£E9l±,X<£f±,X▄□ïKX▄□ïg┴Γ-,₧EOòïI?█┌ü¡{└╓=«aδ,╪┌□[
√╔╓═öôi_7═╛Yr□¥L│σ3≥□□K½l%=lδ,╪┌□[√σ
≥□Æ7╤═ö╧v□├εY░╗□vù░╗-
v╧é▌=╪=K█=·═▓B┴∩'□Æ Ñ>Γ°╔Æ□ÉW╬ígΘF□K□ìüƒ¢ù±ô3▐d□H□dá□L{x1╛$X*Fïqbó¿□S─T1C
╠J╛∩b~ò±à·
aò╠□Ωδ\#⌐╜æ╕"□.pª0î▌ÉÅü≤YPúô═▀A>┴▄□╨£·▄_╪bæh°□X≥│Æó┐□+┐L╨╜S▐I±█□╥±;~╩¡öò≡
Ü»╥£▄#°ôìkΣ}ΣÑ≈╤□╙°f╨Öê
_#;=,□╡4▐oPîxD.□-
óy«□7╨╨ZL╧■□/>"W╘╡Γ□43 L÷≥(Σl□f□±∩F□q╛òF£7╬\l6Ü╦╠□µ*s¥╣┴▄ln5wÿ»Ö{╠╫═âµ□≤┐
y╥<c₧7/Y□□√8█Ωe⌡╡□XyV╛Ud
│╧¼Q╓Xk□¡öJ½┌¬▒ΩR≈τX≤¡z½┴j▓Ü¡ò╓j½╒┌h╡Y█¼vkù╡╫┌o□▓ÄZ'¼S╓Yδéu┘6¡□╢╧÷∞¿▌█εg□
┤□█□v▒]jÅ░G█π∞ëvà=┼₧j╧░g┘sφ□Vâ╜╙^b/╡ù█-
÷Z{╜╜ëJo▒╗█;Θ≈▌÷>√Ç}╪>f┐ifƒ6□┌τ∞ïv▄▒»y┌φ8□G:1+Σ⌠q·;â£!NíSb≈│÷:├¥æ╬□g╝3╔Ö∞T
9╙¥Zg╢3╧YΦ,v□⌐╞2gà│╨Yg_t68¢¥¡╬Äö²2φÿa═Σ╒)q^s÷ÿτô÷u^w

:╢s$iGτ╕s╥9π£O╬O≥╛s╓□l▀Σ┌u▌É¢φ÷J┘;├εn_wÇ¢τµ°«E÷□wÿ[ûiwwö;╓¥αû╗òn╡[π╓Ñ∞~
{╗s▄·n╜╣!ew}Mµ°«
nô█Lû╓≤Γ«tW[EnkjmΓ;
╞πα°Nëhé

─╢q;r■□8╨╥ΩK□F.r-□╧FN□╚K└±⌡æ─╖æ Gp|▕Aα½-t╛Γ□┼±e
q□|□°3α°JJ□êßä·2□╛Æ"-A╬[αê$&Fa<î╗ «╛├▒□ⁿ≤╚·18╛æ
□I─th└□VΓ╨l|□·ê?□□⌡□j□êB□╙o£óî□¼í┐░≥MΣ'└□ □_╢0f
□8~q/j}□m═b~□9ù∩├▌»Γε□αSá┘éå·╕{ □ú3₧□╟╫_─*≡┐B╬╤╖ ┐

°jK╝
·?□╟╖d─:≡Åâc╞□ÿδ+j□░ÿ@╧┼²╕{□·┐□
°+Φ█p╚hK╝è£/#□_g1≡┼□±SΣ├V╞≈└âαÿY⌡²□üÖ□ÿY│□·┐□┐□□-²4±]□□=7>î□î╘─wnî□
□ƒ

=≡Uq□ε ▐èⁿmαΦ¡x□·□}■!Σ?□«┘a□□4□â□|q┴\□■1Σ|□■fα[&Γ□°]╚┴£□|┼□□y
¼ç┘10¢ΓWαƒC>┘□ vÇ▀□Ä∩mêOπε°0w7úεùÉÅo ╨─╓<î|∞ò□▀╝□□"Gy┬ihÇMΓj╓■□╝□wa
±7□╫WÉ□□□ÜM⌠▄@╧
╡╓≡m-├A-╢6<R

┴Zτ╕1ô≈_9@~B

æƒÆE≥fY,oæ%≥╙≥6yG°²x¼▌Å□ZG>▌ìΓδ@□τâkkíûI╤▀v¬Wⁿ.Ω±If⌠{¬=*u2Ü-
µ!g╠□r¬α^╢╥¼½o╢v|»ò┐╓║ù~π/╢-□gI╛ .╙⌡É8ñ╛█J≈Ä≥╖[uΩg

ñ£C║|╗□e}╞`▒═(á▀è)»ìⁿc╡1éjòRIN'î╤Γö1Ä<□▀äe▌b□R7ò¢╙DmDI° ▌At─■9°╞Ä╙
fÑ¡ùDç╟è7╥|□±*1□▓è╜X}*╬ï'┴¢└
ⁿqÑ-æH╞□ü8/░û□je!µ□
½¥½εnN\&■b╨ƒ│□
y□□Ω┘e^┘╔gè⌠}ô╬Σ□d□═√m≥6Ü»;Σ▀ï~rë\">┌E▌□║╞(□ Y╡
Θ\█
⌐Σ╧¬ƒGσ≤⌡□¢¡Wπ□╜□□Xì┴.jE⌐do▓ö¬ò~ƒ·┌┤æ□╙╙JS┌╗╫│t□∩▄╥Üδ╨╥Ü½ƒ8╜¥□·ε(╠╥X~6·ô-
t╫èú⌡▄ì╙δ°·hσ□!∩ì4^{Σ⌡╤½ⁿ┤°═ª≥╓□óß∩Y
δy/Zè┤¥å□ò]□=¼Θ╜Φ)□«ΦâTJ4Γ=hz/3╡µ║╟╘Üδ2Sk«╙LeΩ·sgj=u¢⌐5]∞□▌╒░ó╤}π▌╓╬▄7▐m²
UT~╛▐7-"╣ç>Θ≡□╟└□╟┴□╟Å3N□g□□g£□╬8Q£qrq╞Θà3No£qn└┐ì⌡M¥t╓αñ≤£□*ƒ°≤╬-╘^D

öùâd₧╝Q■╡╜I□╧┐æÑ≥VY&+Σ▀SoU_U/w╨uüXIV·á√·╬╓|7ch°ÖZH⌐E∩N□Öú□
o¥á╜⌡ín╘εε╛T□O.□ubb╧>∩Ñ╫▌mwåÄ&Etv¥⌠ü╢\ä¿1¼█û╝z⌡N└l□ê≥δ╥∩½⌡O╘╤ÑPT╝O-
□c□Ñ:·╛;+t▐-
'j ß□ë╛╛}φ▄╬ñö┐≡▀≥x?[z∩╢Y≤□┘f═□f¢5i;┬J┌□■≥wä□ó┐Σ□ü┐q╓□7f□Q∩┐αq≡■SH3± f )"
·á≈ƒZ}■-
&Ü}¥÷Éε╢═;W□¡▐≈wτΓ·|┐v«ÄX╫¢ó▌√╫å╥_u¥|#3₧▐ú[*ç/░'TèΩδ>+¥█¬□Yx#°äê ╫°z╧Pτ╓&
Ωu²┴£
Æ|⌡~╢T₧╢n+üVy ┌z┐¡╖µ□|▐Ü□╨z▄û·WHⁿ?⌐>╜%╥▄.° □Ji□┤□.╓=σ╟╥⌠□ª□≥⌐ì□9□φ

CKƒí╢nO┼╪Σε:YNµ6⌐U□;¡à¥6
;m□;m□;¡─N█□;m

;mO∞┤╜▒╙÷┴N√a∞▒²≡┐□y°_ëOë₧B≡_□∞é\}╡⌡╒"2╙ε°╫s«íπ╦÷l{₧╜Éh1Iì÷2ó□D½HZgo░7█[ë
vÉ⌠Ü╜╟~▌>Htäñπ÷I√î}₧ΦÆ}╥□Äδä£ló^$⌡u□8yN>Q□I├£2gö3ûh□Q╣SΘT;5Du$═qµ;⌡N□Q□I═
╬Jg╡╙J┤æñ╢îz█£vg□╤^Æ÷;ç£ú╬
óS╬!√Æs╓╣α\vM╫τzε□▒≈5`R□τ║╔▌≤╖╦ⁿ÷t≈t»êH□"A."""□éê□╓è□\æ
□DD□D$□□W$ä□«□─═╩╤
□DbÉK□ìç¼êêH□òp A

Aé>ä □□ "□fµ╝⌡v╧2╗,▓èß‖\ªƒ≈½»┐ ·»■ΩδΩεÜ*├_□(□4□Z╚4□¡□φ□□ü.α‖╫L3╨+╨'P□

□780,0*0□ÿ□n□BO
<

╠ö±□│□│□≤Ç□α□□^□,
¼□╓Ç□□X□l□╢□╓·╖□╢□v□÷□√┴□□dⁿ□â>á0É╞²üáσ_
╝═V¬

ö□□□═dm□Fá(╪□h□0éφ²=â¥é]ü-αz╫ ,XÆ+u░$╪/808$8□□
n□rƒ□£

<─▄╤é╙âσ└□l·═┴9┴·└B»=w□ù□╘αR╖=â╤â½Ç╡

□□«□n□╢╩TP╓σ┴□Ç]▓╘à┴╜┴□└æπ⌡%$BüP8□≡m
Θ![jO¿
╨\jÅ╖π[[▀æPg_yM]Γ.p┼í₧■Vr≈7□YhPá)└k╦▌ó╞äF°;ä╞√├í2ε≥6%4□¿□Uro╖E■µ▄┴m
E`Rhu`□B¡□╝φ»

m□m□╢╔û»□»û₧╒:π╛n;B√Ä╫┴╨A╣□[Φ¿□╖▄├¡ªF╧¥▄é
σ«m▄¡¡╞Yá╡▄╗
ΦN=√<ì£┼▌▄z□ö‖:╚═▌□□

ô:X0¬`l┴äé▒ü5□ô□ª╓╓┴éçâ¢Ç¡9□,ÿ ╟╨Θ`┴l`-{£<[□,ûg□/σ⌠Sε┌F┐5└║£ƒ□O·□

▀─éì└□⌐□□█╢O█╬`¢é²╨Σ}rO╢ÇZΦô-!r▀.▌=mFKΩδ(⌐µ~≈τ¼3ßεÑ├»É□╫│W╕
è√¡Sφ╧≡ε∞é1⌠²□}·
T·□}9ƒAφKë√u≥^≥▌(τ7Mo-
□╬ûqτÑ╕sW4ε÷ár▀□à3g▄o⌐Ω%,mSåqg□∞ú»□å_Z▌┘;┬¥╣┴»·é≤[□╬1PYf┼-
≤═⌠σ7Y⌡Fª▄Ä)╗3□8K╟²V½Rεπⁿ□═▌w╚¥ƒ0ê<w°╤è□2H
[╒²┬δ╬²╨8k┬▌}(├ûT9╙Cπ,□w╫#⌡[⌠▌╧╕ⁿ┌½r▀-[L√_ö╗│□»Ñ£;]hε¼
ε□$▄=g8°BσεC□┐□+ε≈h~YV╣kôÅ│¢4ª¼p.ô╖□□[F╜àßƒ!_@·SΣ9╟I·=} @₧│t4w≈'╬╛p
┐\½£╫ñ╕_«gÉτ╬?Zè╛£}Σ═▀`□-
kíqåÅ7≈µ╟ö╗ΘsÄèÅ≤s▄╝î╝9□╝Γ¬╗°□g╤¿ε<.╖╓ E₧□Ñr╓Ñ∩yª└·0è½ßl1-
█D╗å□╞⌡╗√\qªè╧÷⌠=┬0╘4-
uUú╬¿╝^~╬z≥╣≤p¿Ö>╫╢rgl·ƒñä√÷╕;□y{U╝%iûφÅ^│□≥┼,≤"╓{;5╣≤U^cHû□=HåΣ£R⌠)≡┘Uö»
ú£3a╨¢d□£Wª^E╓àΣ/ærƒ;[i╗Σà;Km□}Y~w.ô╩ÖKΓ>%╬₧àÉ*»ú╚║□%îÉM)ß∞,ò²HíV╕3æ|£oú▒
□ö□╚SoUZ¢∞ô^∩Ée╬═ê╪ù¢╣ö]óD□≥V╞rw╨ó=╓╛ ≤□oH¬Rç}╘Uì╫Kσ

+═¥ÑF¢ú╞□ ╞▌ 7î≤⌠╝·]G╪

1Oô≈σfF⌐∞e¬█&╘a┐½ ▄?$≡□JF3╓□▓□■ôσyù╛3Θ╤Ü·╪
▄¥@□τëí/╤□▌╕,╣z1├╕:#█\├ÿv2┐B╣│╥□▄╙3δF├╪╗sⁿ;" M╨8æ¢≤t∩i*à*Kßσ^Ä<║╟{_ΣNO■ƒ ∞
ô_╢□╕□pX_╦╥╔kVΣò,τ#»ki□3┬ε¡W║·░s▀i;}╔°v₧VA-i[½⌠□8C╞¿□ó■uù▀□ΣS¡⌡%π╫.▌┼'-
▌╤°X║·╞W─TáL(^ε□NδWΩc╫░7╦"{½à□╚wWniraΩ≤μ≤TπτμQμ¼Σ}°╔>)±KÉ╗╞₧oσF╨°-
□U□í'╣û╣0^□mi□┐!~âh□∩□¥╣êmu15°]ui}b"

¢Ü╜á□öW^█±"7k√δ]VW□&40Mÿ╪Ç4í∞ Q/╧▌▒▄+╝¿A\ß\Ös╫yqâ╕╬╢Rτ·}┘Θ╛┌□ü╘┤=°┐

□τ┘öI █ eéWèë°Y∩{Sç□╙RΣ▀5NO9╛¼

╓R√±□Yä½W╞±∩┬□SεÇx□ú┬I<□ï▄?  □B╓U%@]▌A}òe╛ 4ò·δ7CSπ□EdÅ┘
Lⁿ ║5Σh╛□□$5ñ╣l□E■╟}τ□┌*_═∞□M∞¼n½3U□╛Üû≥ë]bù╥BZy┼τ=□ƒm½□i╒▒ûR╤ng█Ω≤
⌠j╖╪¡┤ö≈`▄╫T£M:▌Zƒs┐kí┤>½Y_ΦnΦô╜▒║╒╬Z«·┤Ö▀φòJ□»_*g{e╜4-
┐╤╢┌▓·Ω┌-▒G«│,▀K+!ñ╛GL>▌n'σπ╓µ¿U□Ub÷il▒»f.╢&^□╤°╓á

ßWë9
¿□ε58÷md□δ!τNùë═¿╒Θ╘▐□≈£yYº\²╘3«v>╛□┌╚w@òê▒E,?úΩºσ╒N≥gZ²▄□╢nΓ7═╛8ƒ,╢è□gT
5\┐-
yW≡â3µ□╓┤ó>jΩûΩ₧xª╓╙∩i∞VOgU▒πî╤╪┌W4┐₧▓o₧⌐5u ç(WLj*Σ╩□□╬vèògd]Uo□□9ε£'□|¡F
mu²ù¿!ò≈i┌√9b~⌡╕X«╓⌡u,q╛NΣZ║aûⁿiΩ╙▓╜□ⁿJ▒ª┴⌠▄║4^╓aY⌡ê╜í╫╞□í/»-□¼□k□t}┤╝┌H╛
í╫╟□ü»╚╕╥╥⌡□□rì╬□k≡⌠□e
₧>├¼┴╙gÿ5x·î|□ε□ZÄ½r∞□╔╡Q□Z}Ω□¡·¿y½¬{╥┘z}▌δ5Ök≤-δYδ─╞3óf*W3æ ⌠╨α÷≤╓4╣ +╙1
□╘u╢□7ñz·╜√╙ΩΩ╖dgè╢ƒ╓²╕╥¢E¡ê╣°▒F_t}#
φ>╧{'$τ,□□Kz¼}ƒ`╦V▒╝│└Ml@∩Pk╖√─Ωz(
╣□>╬v╧=Q)
Φ-
oφ½æ \X╓`δQ√ëP>C5╟Üh╕-+≤«H├{□¼i╡|╘░ò╒=Ñí╫╟∩iZn┤Ñ4└'█┌W¿µΦqRâ»╤±πF9╛j╙uÆ+╢
╡F║3╪□ó▄KµóπvôΘΩφàsZvíS&□èτ~-≥├⌠Qz*┼J_Ñ┐2H□¬îP╞(πò2eè2MÖíT(ò╩\σYeæ≥óR┼≤□╧
jσmeâ▓Y┘ª∞Pv+√öâ╩QUUCjT5╘"╡⌐┌Bm¡╢S;¬]╘εj/╡ÅZ¬

PV½â╒aΩ(u¼:A¥ñNU-
«□□gª:K¥¡╬S□¿ï╒ù╘eΩJuì║N▌¿nQ╖½;╒=Ω~⌡□╤ù+c«£ÇÜA9P^=°□╩2jq═╥□k=x.╤
h─╡6,╖Wv¡╜╓IB═h]se╫zh╜╡□¡-
╤╫rk□e╢╡!┌pû□É<1Z□'δóM╘▄Yæ"╖éöµ«iΣ«│2XRàδΓh¥╚╗δ╨\D:êö½4⌐εΩG▀τ ▐>bHw╫,«µΓε
¼Ñ÷a□ε□µεLÑ▌├≡;╔╗½Fq-
□╡£< ╜⌐▐MZ╩0▄{GφJ₧√Ve▌□í┐Nî╥û╛□I╢>ìVI9╫□r≈πRÿ▓╧]╖ê{C╢½@e╢Bî╩⌡«╝òo┐

ûΩ«¡┼ ░k▄c╩]9╔╟}╔▄□ÿ┤╣îσ╢├
ñï□åk╞hnxε╧µ«□Σπ║2▐Ü1+╚≤ƒÖ□sW╡
û∩╫öl ╧ò╖╘}Σ▌5É.í/WÑR╣╧¢╖╟¥╖FÄ╖□₧╖#û╩╡é4«xñ░╓Ü╖ZÅ[╬ C W╖Q╕S£Å√

╣δ-╣½"i\δKσ╒⌠╣+±p□,ìδf╣{öq⌡#we

yWv╫▌(□╟÷íRiG▄□M½C<□ΩÅ≈î□7▐+~mⁿûZßÜTç¢□ΩïwÅ_□∩□/«

σε₧)w□Ω'Ω▐¡+□R«□╘-Φö□y╞╡□:╤U±,╤ñ║Op╜Kà+□e╕èVåk<!∩V╕╧uσ┌:æjδYùφ¼k_╧êÉ Cù╨╝
ñ╗xæ«J╛èO3□ ».▒□6rå¿└□u.\w_¡║▌£□╒I▄□₧╗·8≈m/\nà╢B÷□^kε╘¥█EÉ╜;C□╬╖+ÉqO┼

u#s═▒~°6!╧□≡|\
╬▌·□φhú╛═╣B»Oj
⌠í□x½╬▌═«≥┤αÜj¡)╞╜≡□!W!□A(−,!╫B□!╞□â─xΦπ□1
5□□╫m┼g)ùφV╚═╠r│□dφ╪&i÷─

√ZÜ½═Ñ□Jh!╙\τ,├-Üµ╬uΘ□IÅ╛w¼}╥┤£□«yûf█f╕║[z▓╫>-
à▄╦QΩ┘τφ°vb┐0╥æ╫⌐─âΣσèc□╝s╡n┌pO>Z╚□W┬▄□╖□¿▄□╖□╪4εò┐□«½□▄τZ╔r%6wƒ4w╖╔,-
å╕sfÜ╕▌fTR«¿ùíµñGô?ƒö;If«°
æ½□╣+π╕½π<xÆzτ╔▒·ó▒□|⌐X╧yk
td\┴XôO□KàeΦ╩╪W±il▓ÿUcUÇ╔°Θ□æ[Ä□YJ□π╩S⌐█5u─■I╜c≈<Ñ╪▀═Ñ╪╜N)÷╡°□√û:bO⌐w∞ε°□
√ΩSè▌πöb□3vc«B`Σ┼«ÅÄ□o\αÄ
~*╣Z╧?5WÜ>÷□□f?□ µ□ü8⌡
°W█N╣╔â∞7B╙5"σÄ╕r─)≈□₧□╟≈dÆù½Z╙□£\ÑUÄ½Σ╩Åkæ╥T±░ÿ
□1□££Y╕ÿ hY╟9□δ8ùy{nù╘∞d»φ$╧;ïαê:╦□╕ka□«╨Θφf|ÿö╖αf≈{≈□╔s»]ßεc╞⌡;3□□
f╕bOÜ+wf╕.fÜúδ⌠$▐┼╕ÆbÜΘd▄╖-S╦p╦î₧g▐{-
K¢~▌│≤╥µ╧▌╬z▓□S┼□_╜┬▌ß⌠nªf±.⌐┬φ⌡òñ'▀'K□ùδm⌡■JR,b]}▐No┼°£ª╝v□╙m╬₧sc\ε¿
W╢öw█(í░▀╕·QΘΘü;║

h╫k7j²┤æ┌▌ ┌□╨□ Mp5τw╫bτy»ƒ□[E∩g2m÷┌DΓ□ë□BΦ╖Θw□U-
⌐Å□□I3┘Mÿ╔ε╔╛Γ¬di▓T⌠5▐06è□îM╞▀┼mf│Dⁿ╙∞k▐$╞¢╖¢╖ï2≤

≤N±Ç·á·[1┘«▓▀□╧9/8/êW£5╬¢b╣│┼┘é▒ûèæÅ|Bk├┤ ·4Qδùφêú2[Θò╦à▄Θ7`Dî6B1/7/□¬òMíⁿ
÷V{½P£¢□╘╨□σîÆß4╣Ñ╨-÷□ó@«
∩²╔Æf÷xG□□úq|ä□┐l$□╔□╬□╬6╬6╬∞┬!□+p

HJ╩.╞Q₧-╧□└▒□╟~□╙3╙│e┘▓ⁿ┤│F╓¿N[åæiçp ²ⁿ┤seb┌?┴qW÷.ª-
≤ö±^╩╝öK╗║ⁿm▓mÄ½Si╢4 ╜║▄Sp┤╧┤¬ßçrτ\^5□┐□GÑw─q4╟æ;»≥Ä·8╝+M□ç¼cìr□pH□Θn╬n╬╡
π╩#ε□≤≤Ä£┐tταÉΘ.─1737▀_╢Ölïjm╤╡≈A≈`<□╟╠Ü'j∩▄┤ön]╫2W∩Ωr
∩É·╚┤/╠^x▓┤Yw/mΘ/╧δJ¢φÅ┤3φq╚2m├▒□╟█8■Äc₧wHÖw-╙½╙½│Åαÿû¥ûÖüc-

Φê╘3Ωë*□ôù└□□ZÇ8-
└·┤□═h□┌í°╧□▌╟Öµ\Qb»D□┐ò=}${·]╥j#>ε═╔Kôùè@▓C≥□≡¥ôWé∩Æⁿ6°«╔«α┐ôⁿ

°n╚)ê£‖â^ìnTµ°2??≤≤3?
•═□}

╣ ·æδJ╪ü╫φ┍"d □%≡í□kD┗y□σPY

i█-åU╜^╛·╨`Ka□G□%1 1□⌠Ä─p╨æ )╡+1□⌠■D□Φñ─$Pi□□X─█ä_¬□ç]□íÅ□>X╟Qα∩╒-
□A²□·cá□z□Φ,}□Φ□}

Φ; ·6□╤ ª∩□ì`GS¿╒9╓s@╧M6□òφ□O▐ÿ□□■'╓ΘáO&τé>ù\"ó╓□ÆUα▀Jn□ßΣ?ôƒ┴□-
J-□a#häEÉv0f▄h⌠□ □πçó└□l

 cú

⌠g╞/
Öa╠□?╙(□}▄@⌐îτîàó╨Xd,□]b,□}┘x□T╢o┬°çü\î├╞aäL□iPÿ□□4┐e~K□╨µ□fg│3$]╠.á]═«á▌
L\3│╗ëkf÷0{Ç÷2{ü~╧ⁿ-Φ⌡µ⌡B5K╠□└≈7□□¥wëÇy'ε□¬9╥□ëöGÖúα{╖9□⌠i▄7Bµ□≤9□7□ÜKE╪ⁿ
â╡\─╟W═╫!_e╛ ~¡·□aÜ¢╠â"d]a}[□¡n□╨`u╖P□½╖╒□⌠:δ:╨δ¡∩âûZ7é▐d▌□z│u3Φ-╓-
áò╓2ñ ∩"▒╘□⌐N┬L]ù□

·π╘□╨'SòBK=ò·¥□Ñ▐O╜/□Ñ>I²□□Ve┐"th█δáo╪oÇ«▒╫Ç«╡1┬│╫█δE#{â╜□ⁿF{#Φ&{□Φf{3¿╝W
%φ≈∞≈äe∩░wê□{°╜□≥□φ□A?▓?□║□wtæpè¥b□t·8}@K£□╨╛N_□vJ¥RQα⌠sp▌y╧ï9╖;╖â┐╞╣□╛w:
w┬wñ3□□y/î9╦£σα_s^□ò╜"ε¼u▐B╙u╬:äⁿô≤'≡□£
α ∞ⁿ□a69¢└ ╒·+Σ[¥m"Σⁿ├┘□·╟╬'"TΣ□□a╠á*╢╝┐*╜òe"└7p_∞~·□

╪≥1o╛ c□Σσ▒□╟╪∞╪£¼«░ó?Ä-óç

+]1□çö╓░╥ûK;x4söa█ëvö?ö}êτ2¼îôKwPv□φ÷:□├q□eïΣ^O┌eÇ£«,ù,□εô┌aq╧í_~XUä8r□□9
□╤π█ë$√═┼□']□[)u┐=GHùR╙;"ÄN█ó┴╢

à=╣]□ks□φ╠¥HK±∞╠]·]□3Z
■n}

Σ≈Φc┴ H□■╟╙Oe~>µτg~□≤SÖƒ┬ⁿΣ□0E_╬

▓ö!Z4ò□Jú
≥3┐
{╕Å·□░G·┘ú□┘ú□┘ú□┘s|∞□□Ω«▀╣═╣M□RS²╬□g□F┤¬║□║Ñ¬□╡bΦû±Euδ½²Q╦D÷ô∞'╒özΘ╤*·╓┤
□┼ê┴ï□≤┬

Æ∩B]-æ:□·x÷q≈~ì

Σ□╓9╛Ñh·%≥u%╟≥═I<▌ejn╛«/╞□╣╝≥·zσ;3;|║F╡δ+j╘ù=εä⌡e:. òσ{ó·µ╡φ±⌡¡πè□┴v□¥q□╛
Q¡ÖÄ¢ù+╧□â°□⌠ò¢ä@ox
╓U>□┼!ìBZé╛·}≤√ΦS}╤¢□|╝□}¬-
Bk□]à1╬╥÷╦B╡ù┘╥└┐é▐ñ┌╥φσ≡}┍~□=kà╜□ⁿkH╫╟teo□■□z╗╬╤Mí>]ƒ.□aî3□÷µQ²Q≡3⌡Öα-
├¿╟°ùδσÉⁿ□cU□\□□╝□□⌐·□ΦOÇ□R□□|Ñ^ ■W·»└╧╓g#╓S·SÉ╚QÆO ╡■k≡s⌡╣αÑ}
p─$φτ7DêπúBÄÅ□9>*ñ⌡)α°¿É6(╟qP!╟>à□⌡╟□>π1π1î□╤1÷±□┐4~)□□□F□°╟ì╟!ƒà╤É
╧x▐x-r9÷ë□çîCα□□G ùq□a╥FF42à)@□S┴òPM
╜╧⌠â□ÿ┐≤ÖA3□■□|╜gwπ□□r|T╚±Q!╟Gà□-
□r|T╚±Q!╟Gàµ@îëb┤¢IÄÇ□═ëµDñ∞Äâ>3Q*≤░y□Üq─<□>mª┴gLY6<ÿα)XXB°,┼RE#K|P6+`ílV
╨□é□Y!≡□V□°B½□┬ç¡□$Q+□I╞èâOX:°ñò□oX)≡ù[¥└_au□ÜuÑu%°.V□≡▀╞h╤ou╡║B≥□î╣4δ*δ*
≡rΣÑYW[=└_c]□╛°┍□ⁿw¡∩é∩e⌡B¼k¡k!æú3┐⌡=δ{αï¡δ┴ùX%□≤}î╘ⁿV_½/Σ7X7Ç/╡J!┐□c7┐╒╧
Ω□·MV□≡?░□@~|5□ÆA╓ ╨Z?ä|░u+°!□╞Θ╓Pk(°█¡ßα_╡^E°╫¡╫A▀░▐□]m¡åⁿMδ-╨w¼w@ d²
t╜⌡□ß╗╤┬|å⌡«╡□tï⌡-¿╜ƒEx?K≡~ûα²,┴1`îc└□╟Ç1Ä□c□□□9□îq

□π□0╞1`î≈┐0╟Ç Ä□c□□╞8□îq

□πΦ»Éú┐ BÄ■□y┐

S¼τ ▌/├□┘ □:»:+╨█σ°«□Gp>τ]τ]<⌡$^{n}$═┘

■}τ}≡□wvΓ■·□τ□│
ú9┐≤í≤í╨£²╬?┴p

≡█àα;▀

▌ù□·ÄS□a9-┴8□#

9╥Éú□î&å╥±□,P□Θ;≥φî"≈  ê
¢╞OA/oí_□≈~╪æíLπ

╪Æ ·}░%░#Φσδ⌠?íùo╫▀G/τ□" ·y┐ΣαΣ¡ΦΘSÆ?ONC_»L>ò£â ▐╛□}²E< ²□╧Aç≡ⁿs

₧x□ú╖▀çτ£□xª·¥|èA?^m∞2÷□-
í□ú∩ó?û└Ü┬Æów▌ë┘╡ ╔ⁿo≤3h□'⌐╫x>╕IΩ□₧□₧╖^▒6α□_Çk√$╞√┐I¡H¡I╜□[√:«φ;╕Ü□p□7ßΩm┴
╡{╧▐Ä½╢╙■□╫¿□«M_î╚√9□£ü╬ \¥;0□-
Θ▄φ▄πⁿH╛çcÖ6□∩Γ9╡£¡T─6n,▄7╖??█R^KàÆ═ô▀¼╒Z¬░öµáMDï□φeΦ7¥m╖╧i7ML□╢"τâûï╨ΩûS
ER□Φàx6
δ1ß╫□║.□╨û&ε╜)╜HD⌠s⌡≤EB å▐\Xh█□┬╔/╥/□Ä~ë~⌐hó_ªw□τδWΦ¥┼7⌠.·╖┼7⌡k⌠₧Γ□
²Z╜╖╕P/╔»□▀╥K⌠┴Γ"τÉsTtq2E~qò┤□║q÷j}«û█J+╨µó╡P╜╣)?»n7Ew╬║▐τ┤^mï:φlky¡□¡«█╒
╔─(╧âµd{%R]àtW╫i]°¥╡«_░
δ▓┤╙╬Z┌□pσΩ▓║╙╬Z▌/╤Æεù·╕□├æ┐√□t(t□ÿ≈Ñd4z└□□╪┤·8}╜ê≡╜d□╡□!töt6g□□O·ài«Φ╞oK
r╖⌡µµEf□≤b│]Ω▒TyΩù⌐□√c√□√ƒ÷°÷g÷a√_v┌╬8èú:~'Φä£B°æ□qbN╥1¥öc;τ8ì¥≤£≤¥fNs∩ÖG
τ□a⌐□ √Kò ½,æ¬╙Y□>e╕▓_□Σ½ç· ╥Zªgf[fc║8▌═│K□╣█h·┘╠äl∩∞π┘¬l╤l7┘┐*;Ωⁿ╠╢πCª╖°
╖dfe&d╘⌠4ñ┤*;<╖□⌐Å╬∞Æoπ▓ïü▒□┘Må╠¿Ö╡Ö╣□╣,37╖'[Ö▌ô─æÖσ}⌡ò!+□Öµ□/Σ╨⌠·πC□□╣╕!
□q□u/ò┤ôE╔sôτ%╧ù#ÇdïΣ□╔
ô¡ômÆm½{G⌐yú|Of>m>c■&⌡Ij□Ωƒ⌐□⌐OS□SGSΘT&ò╡½╨□û┘»╪╤φWφ□÷kⁿ.)┐╚)H□:ê□0Æ@.τBr
^≥<╨≤ôτC╥<┘□ⁿ□╔
└_ÿ╜□┤u□6□╣╖□m¢l╤»[╣wv□▀┘·▌wv(═╙≡}╞|□⌠7µoäèÆ□□□╩öσ□╜╫°åε□⌐=Bæ·□┐╟R□"

²·□O╔ƒ╪ƒê ⌠H~u·╘■T□Bƒ>□□╨⌐├Bà^²
a╥vZ°φî¥□□⌐cBüû·±£□M├≤□íSêτⁿFN#Q□¥ïê$⌠.&tΦ^RDá□&$)'%Γ╨C[$ñ.Br₧s₧0ñFèÿ╙□Z□
e?o╟æV_»»╙·S□ZFi□çú°î╘GÖ╫K;┼/g¬≡-
▀½╤ôgèf∞╔r╢ûæδ/∞□q'ßΦu⌡□τ¢y·≈⌠≥□Σñ·k┤!é6$╬/┼M┐`₧ε□æóΩ7"╚½τú□xwäß·¥^╬╖╦□*«
[ì■^#ìµLπ□╟µ┤└▄▀j╙xA{ƒ}─>jg□═±9üΩ□T╨□╬□□båL]µ#┐⌠@ô_ü□┐V²

F╡?╞╡ ╒╝·<═╝□zT┘ü@
╔Xw±
æ"|^╔lÄ^┌y;äµ∞▀L·_$[▒┴█╖█≈#⌡□∞╟ä]d□Ñ─E╠gå²(S9V□═╧╪σ¼\a^╗┌h╤µ▐|┬╞¼O.»SK∩°╥
?v╥╓▀o?P+╗ⁿ÷¿]┬╟N□GU÷╔┘Θj@¥/b╕□╔πτ═Ç·|║╜[=□π□╧╕╨²▐É√ε
▌║╚9Ä╬¼├□x-_□,ù≤zpTf7╗3|j╞¬æ┬    ≥uK£]Gn¥╝╥╚//┘□H+╜Pδ▌t·?□/□~9Y╟√╞
╞r┐Ñ<σà┘|¼ñΣVσ╩/┐╖µΓ╗▀y╝»▓wßN▒□gU└-»□╒▀Ç╝T□≡kMeuδ╬»σ╧yO┘¢□¬¿û▀bJ╫╣╫F~¥û6%
▒4▒Bê─╩─ZQα\ΓtÆ_├E□ñKàÆ°CΓ□BMT%¬□εσ─╥┬ùXûX&ⁿëW□»ê@byb╣□&^M╝*Bë□HÑ□⌐¼D¼╫□»
#╔¬─*─z#±□b¡N¼□àë?&■(□%╓$╔êpΓ═─¢ê╡╔╦⌡□í:φ¥÷░∞ù:ù┬╩wp:└Ü_µ\□╗▀╤Θ(é╬σ╬σ"ΩtB
·Σî┴s┐d·^K╝år|^)□▒öaû2Æ_╩─[ë╖p╧⌐╗¼Qû5£W╓ÿ[Vτ□τ□╬j

├÷ió╜6J▌G≡4╒□#▓k⌠!Γ□[╡;ï¢∞.÷□D╣s¿()ûx≤"q¥┤n-pÅ╨èΘ*Z_∩ⁿ□T¡ I·|Ö¬
:N^3ⁿ╨j^µw<F└oL^▄±╡Γù╒Yû·ö≤dßΩ¬ôï)┴╜≤□ynE-╚┤pGò àc╗□ □┌╕εr╓┐ìkùív□?█@┌x╤
┌ß┼□ΣQu□·╔Γ□W≤è7+]▐≤ÄÉVÉr╒┐├+╙ Ä⌠u╥!ö□¿-□╢s├+╫ü╞$ƒ²Då□¡α8ªé &Æ⌠▐Ä
ô╓┐▒óöl□èz╗▀É4□û4█£Vá□· 0
Ob□qσ▐┌┤ÇE╕□□í)-B{XäÇ(▒Cv3qú}!¼├□Zç┌┤

≤ñ²HîJîé═╛;q╖ⁿ«-g

╥ë▀Ω╗╬2τ┐ ╘═□÷<‖╒□,}tùτ╓ä□▌{R>_ªD□£$¥#╟°ÿ8□&□¿□.□«3╧·öτdßΩ*╗¢°

╪yτMÄ╣▒µ5!╙è╡□┌zmùk┐»¬□δ.c¥φXú

╡█▒C□i╟╬u╫7╓φ□y⌠¼[~▓x╟«Åz0▄;\□ε□-□-□---□-□₧□₧

î
?□Lτyy°     ╕sÇ·└┬≡Æ≡R`yx□░$╝□X╧≤M□╖$╝□°□╪□▐□>□>□□ß#αÅD□@X≥□=|
bGÜÉo-iσ║@[ä▌
╖□ⁿ;D:G║ü∩□)ª□╧H▀≡▐H ╚á╚╨╚ê╚□á?0-(#?□≥iæ□Σ+"òtτ□╧"∞
╕ïα┐(≥bñ□ⁿè╚j·┐□┘□┐═æmæ□8▀
l□÷□□╔ì∞ê¬╤ÉΣú╤¿A╖□hè░█α╢Ç□ïhδh;≡□ú]Φ▀=┌+▓-
┌'Z□□□□□□□⌠□F□c╔OÇ|Rt¬Σ#□ó□S6□ÿà░Ñpg├□vt^t□°┼╤ùΦ┐8║

~+úkêu└F`ïw.▒=Å_□▌□▌ôw.▒▀sÑⁿP4□=□≤┼□ü8`┼|8o

4ï╡$▀□h□ 6▒N<∩□⌠□z╟Jx▐/60Üë
ë
□|▒╤└8`"0$6□x(6¥|9╨□■σ▒'x>□ÿ□,î-ß·╥╪rñ▒*╡ûX□l□╢zτ□Σ±δc╗□fU-÷µ┼=□;□□@
v$v -□⌠x□░y▐$▐□~9┤□┌╞;@▐□Φ□∩□/Ä≈□·□=πâÇí±□└□₧ÅÅù┴¥Γa□0#^□y%07■l|QⁿEá□X□<□

╜
lá╗□▓m└

`7²□┼≈□□üú≥<í&B±Eëh┬H□□Mü□@4╤□h□tñ█□▓ε@/á□²ìD)0□□╠≤a╕█□ë▒ë
⌡╞ñ─╘─├└╠─,≡│┴K╠J╠âl□░□x X□¼─H□□░□■+□n□1+▒□▓φ└N`□░8ö╚╘□'(¢ε╚
⌡8α╚-╝1·fα[R▐╞ô╖'O·
╚Θñw╒{□╜=╖D∩°□□åxnë>□εp£□τyφ≡ú⌡q·D`▓τ>□L□╧□Θ≈□Γ═□µ╚]¿/ü╗□X

<í»╥╫Ωδ⌡M└V║Φ╗αε□

□k⌡#Iæ

$├Çε╣6╨□h

H┐V╔╣⌡Bçdτd7ág▓□|q▓/╧√'□╤□□îÇ¼89&9>Y□LIN□?-
9âτ□╔J║süg!¢û\ö|▒^¿J«H«□▌□6□U╔═╔m^Yv□╗üm╙wƒù∩Aα(░/9═PìÉ□□

á□Pìª F
ú╡╤

Φ□┤0‖□▌Θ÷2·□Ñ└□╧═╟α:d├ÇΩΣ╟&;□□ÇI└Xc¬±0▌Ö₧;ò■5├╟Γ·l□≤ñk,□□□/ü_ª□7&□ôÇe╞Jπa
‖k<w%²kåY╟≤┘-µI╫╪□l1^□┐▌╪i∞□÷□█ìCFF‖ª╧,4πÇσ╣y0□-
/3□□═╚╖4█ÿφ═NfW@≥=╠▐╡PR═≈3□ÜCÇß└háƒ9Ä2ëëµ└H%▄╔└C<ƒ.eD╣·D-
öT≤s╠·µB@‖KÇÑµrs□░╘\k«ç╗╔▄j~□l2wÖ{ß-0ÅX┬<b-░□V□╝n┘@□½9▄VΣ█Z□Φv╢‖┴φi┘╕ÅÅ□□
╤□O~□∩√╕┐[┼æ□V▀╚êhkb¢tσ=□≈iò≈q▄»-
î□¼A└PKbî5-n□·)╓4‖3¼□╕ò└\δYk□≡óUe¡Ç╗:9▐z□╪`m╢╢┴▌aφ&÷Y□¡ú╓εöÜ□Ñó)ân□▄ª⌐□⌐╓
-$■

Φ□┐.@w½*╒
Éa√ñJS□Ç┴└0Åù□□î□&xⁿ$`*≡00□ÿσà¢¥ÜùZÉZ£z X□¼⌠xë5╘:`#░□╪

⌠□v□{Ç²^╕C⌐îφ▌
φ╕m┘iüf@K└G┤▒█{╝⌠δdw□z╪╜┴ùP▐╧-ê0═∞6æ■÷□î¡F`£UàqW□╞`U□»⌡ÅT┘├]ù~r▄åδfÅå|£<╖
'┌ôß╨q┌f√!îÑFD╖`|╡□╪─╟Y□c╡│°╖.²Σ8
╫5<□≥ryn?a╧ü«│=▀^h/▒ù┌╥üU└Z`>▒▐▐Σ±╥Θ.$╢é └;_é0╖□Y▀╜É□░Å8□□8aGwl°
xëµæ*°□╨6┌╬Θ□tåƒD7°°S∞⌠u·ç≈:²#=¥Aß#╬╨╚
á┬□ßîq╞G║9πúQ≥e╤RgJñâ3(:╪Ö□_Σ╟H□┤Θ▌`7╥<[,m¼┤±░▀╥f╩■!⌡_Ω╖╘_°┬nπTó□sQågφ⌡u?
╙ 4 U>╟ïO5·¥σ╙╟.╨╥°~ß2╔+╝□╚]□(«äd|≡j≡I≥[²[@▀╧▄+·Ç\▒lA≡□╥7≤7J

æ╬#}åt?π╢c:╖P□b°)Σ≈1à+)┘M╔sñ
àón≈²^(┌O3¡$∩

╛8√ñΣ□ö□4╥ä

ô~W·f6Rr-%□&»2àK(yàΘ▄GI'╨╗3□ö▄K*▀⌐l⌡╓òz╖fz░

rà╢¡┘G╚/'□D║êa□⌠MP≥□%!≥□Σ├ñ≥zmM─
ñ|(%Ññâ┘Æ□! □· fεm(╣âÆG□½'y╖e:╥╫a╛τR┬+Æ9ç|S≥τæ■î┤ÇqτÆτ⌡╩ⁿö□^à

δδ □■•

╢ë═v╕æφⁿS╢║₧E╔R≥□╢ß╥lτòö4ó∩^╨_c□τô□Ä≥aö┤öφ£NSr
i□h╘7⌠ètëΣ²kH□I□ÿJ:□·░╧≤}æ□h¼┌╧╖X«Hû·XRy]╘~■┐J□8ç⌠>·fI□@I_≥7ô■Éßƒ$□-
ΘDI╙█□≥"Jf0╠Zµ╡î╣□R≥!├┤$²□σE

?ÄÆ┴Σ╟3Σo╓□%}£Θ ïa«áΣ9å┘╟□╪╥□╕çu▄╨: ·I□O:□-p└⌡
┤□?T╛oSµf□H^╓
□m¿ⁿWf□Θuötòa2WôG·ò╤ö

öKy·0}»ùß╙ƒ"σÿ║°l╬«Æδ╙□dk─é!JtI3╖JI□║°^□□&·╟=

Ö!▌MJyα»⌠mI·□)léz│»7╥YÜ·□°É MI□q≥/Jy·c·Äô╓&²□σ≤d¡□úH╤□òî□

·üÆ·≈1à▀1û╝╛-□Rù╛¡²æÆo1àsIç╚⌡┬ⁿà╟σm·&ÖKoΘδ;─╘^'}öß□Θ
ö\╟öúîu)°╞,╣0âUT╟ddÅ(≈⌡ô+¡Q□╦y╒╩²7HIf□Θyö╝G·)├⌠ □╞}ƒ4╩□¿▒■¥ñ□Hùônb¼
╙wf°ò,├/e□do╥T∞╣6:;T≥┤iú3Ró□·ÉCÜó∩0╩ïàΓs|░3┌ò╛ëÆùφΘs□▓□o·>ô╡vkΣ?╩▄ □-
≡▒ö(-e□¬/-5°e└ÑKI»$]D*5│ùo□∩□ùô▐#S╚TÉoIz□Θmö╞▓)□·╛+█9√╕Σ²δ$
□░σ□J╔+ñY╥□Q▐-┤┬w;╦3\≥■°%ò≈□≡òñçHe-~δ√É╓1░╜┐╚╗Æ≥
kö⌠╧&òÑz0≤îΣâì╚≈b╚╧╚ ₧┤#K╒ÿσ|ä%I▓Σ┐by╩Θ;¥ⁿσΣ'ÉƒGz□╤0åeÉ╝≥□ ▀⌐ERç▀±²òÆâÆ╩>
«à╥σ╟²q╥gY*ïi*ñ:Θ□╞║ÿΘ□1¥!öⁿ□u▀□^┼Uφ;{÷₧ÖS89τΣⁿí'ê╚EÜRñêæG□kèêö/"╞□╙□1Dî□
)FDDñöª4"ì\îê╣y\îò□"≥0V□æ≥□□╣êH)□y□#Å"Eî□(ì╚┼$τ²╓oµä□iß}╓√>┐·╥┌k╓Z{φ?│≈₧=
k∩Ö□Kîv¿7wÄâ┤┴£□£æ1Ωπε;ê┐□ⁿ\W.Θ[H▀#5)qæ├i╝'µr─>D║ö8╩t√3W]╠╒

û.M║?ΘGê]j■æⁿ□'τqb)E"t+Ñ}y⌡╙─Å□╗─Å□≤ÄcN□╬Lò┌s▀Lî£δ[σ

k□W┌□Φæ╟y!s■□≤□"■ 1l┌C╟ û/%JkW     â·ë■Ä□⌡
·ªÜf╞╧□▄uëπ≡8▐CÑ

□┴□ □─▓ïñ╒+πⁿΣ-rxÅv┐J■□Σù╨┌0Zô▐±ö┴

=~Tμ3Ω)╤□û>Rapu╘Aμí┬·⌐╨]?□┌¡ªt0Ñ□Q┌@Θτ)╜□╓r═p┌ö;Q.GëGÑÅ╪╢ε'x▌σ-²εLäeΘ`½╪J
■Yμj□█X□Ñ□ä÷"Σö▒□cXó}─╧□┐Å□÷2«╟X├àú□╓┐B╬\┌/#=ÿ╓åÉ3OZùâ½¼▌╟□icμ□╥-
9J *â╗Æεδ<╧~tÅ\q□{1≤æ□é¼∩┬▄├╛╒Θ□╛·!{▀τ8¬□┴]C!═q~"úìi□¢Rô·w□
┐'Ü&μ╚╖┴_vE□NªÄ┤╟v^62¢▌Ω>└▒óÄ·╝ç8╬<ⁿæ¡┤åΘ▓Mè□u₧c╤y^ï≤ε╜─□╙9Nz□Θ≤ñ□─
I╢ä<iÖ]□R·]Zvh·=Σ╘╚Hδ∞eYεg,Åⁿ□─Σ8∞
╬7ê□□ Üû▀C╦r≈▀f╪□╖>═Y▄0÷□·╢≤z#3·⌡╬[ë□□\┼6VdΩ□k>c□▓╢┐@¥2Γ□╚i□■Üπ╧B9╖ö╝□□□□
ÿ│┘Öñ≤⌐s7Sτ|XfbÉ~àtôΣ╟~
╕╜s¢╪42«₧∩lÆT⌠□2ƒ!b⌠ekƒΦ¿"√/Σ╠#■<q-φ£g√·4G╝iB¢çÑ6∞¡¼
ß|Qw░┐°╥o1ç□□Oa~N□ú6╘/

╟1
ÄZÖævX$O=¬(#sâ□2□□√≤ ▐□
≤:5╫-
□£Mört▓Σï^]εç8sÉ{▀dÖ┼!╓\j■ÖW\÷{·δó╒z╓▀╡.zKΓWë#ûòz05╦□Ñ>¢·«□N=}δ□δ²ΘiΘi÷□<
╖~┬■j▐'≤□╪δ≤□µ=l 8∩X▐ ∞g╣^:3Ç*└╝
|5¿╚┬□╥=y*R}□;K{╨╦{Φ¼╝F»ß║i▐L~nñw╜╝√╨□X╫π|SÅ░Θ□□[Φ□æ□A▌eδ∩ì¬╟δτ±·⌡╪□«¡╠▌
$⌡╕∩5╩{≡5╥8≥□ⁿ□┼δ╛>÷oz╖⌠>■║p¬w[∩
╜/⌡εΦ})l□BßH8 □à√å□0╞□□□ä┬□8ùpT╕0<.\□(

ùå°å°çg□¬┬≤┬
□╒ßÑßσÇòÇ□@u╕□░

░ ╨□n□o□`7`σK├□□G□╟x~"|□ß┘≡┼≡e@W°bÄ h@N/@

╨'á√□□□□□å□F□zús╞□╞τL□î╬)AXå░□PÖ3+gN╬|└¼£EÇ%Çe9+p╛□░:τI└å ▄£≤t╬6└╬£=9√s

σ□=i=9□

σ£    □τ£╦i╧╢Æ╙-▒é╨ìä#q@:╥?τI└å ▄□□ö│
░32$τJdHdx□║æææ╗□c#□"ô□ô#S"□□g□f□µF□É┐□ⁿ┼æÜH-
Φ:B=°5□_▀ù¡ë¼E8□░Çτ>¼e8Öz┬▀╚≤-æ¡□□/l#▄N▐.└^─?Ç≡0á□p□qä□□ⁿS╨o□}□p
       y╪às□_▀ùu
╜6Φ╢!-╬ú6xmΣëmΦECá7R□A-┌$-≥╨å<┤Eô╤╛Ç□╤ⁿΦPä□ÇQÇ┬Φ8≥ï└/è□GKAO□LÅ╬□┐ÿαδ√▓¬Φ<
ä·Çq<_□(%Olï^5ΦÑ<_-]ëPΓ5□□□δó¢óM╤µΦÄΦεΦ>@□α
α□Θcαƒê₧&}6zæße@□t¢úM1□▌□3▒^9□@╟`□≥XƒX┐hs¼Wt7Çí╪CxBlênl
╬□Sw□twG□□□□`ƒ╪□>tF°!eÆ□ë;□ⁿ1Lk|l"h▒□≡b%▒▓X9∞V□J□│□säÄ═Åò╟□┼ûÉ^□[┴p□`u¼□q
JbOB■dlCl3Φ°c█(▀□█□┘□╨□ép□ΘE┤!║√q~ê║Gc{□n□»□P)v╚▀□k
┬m┤=ƒqOé>├≤s▒väb+αE¢"ô□ôcW"□□g□f□µF≈æ┐8║#▓8╓+R
║.┌ök┼·E&ß\Ç·ülM┤□ß

└>₧√╨╙p2φé□├≤-▒ëá□/6║¢╖
░□y8Ç≡0á□p□qä□□vOA┐
⌠□└%Σa□╬□¿-
╙:É^□t█□□τQ□╜6≥`¢zú#mHOd□ΣíMΓey¿┐╛Ç□╕₧C□□□F□□qmà_□~□«I)Φ⌐ÇΘ¿ ╛8□≡⌡}Y□«e)t
K□O╬□□J╓□█ów□⌠□₧/Å╡#öx□/°=╫═
□ΓÇtn ▄A╣.xCé░┐╙{ΩΣ┤GÅ±|x□#s1>µ▐
□¢;□τô□ô□SHWΣ╬`8;□+(□╡╬\₧/□□÷r□τ╓□ja».╥?<=╖-░&<=╥?wmεFå[ép¡╙»╤┘J▐ r╖Kÿ╗
░7≈□╬□τ╢Σ-□£□□╬m╤╝└≡R□╢Q■j¥

9Å█□ä$îGΓ≥₧j▀°Ç╙É\7₧□□èz□Æ█?^└pH□÷□·½uó╟x>╨□)/┬╖□¼┐x!`□áêtE╝ÿß∞
¼án╒:sy■j{5ÇZ┌+ìO□L□┬₧0≤:╜*└<╥
π╒ÇÑA╪□f^ç╖□░ÆtC╝1╛

░)Çªxs|□`7`_╜9Z□?□8□?□?□8□Ö□?
╕□Ö□┐╟░□aM╜¯
⌠╡pQ □□╜╤τàmä█π]╤d┬□zE≤□1ä}ó┼□ï□²@□□⌠
á□e╒áù□╜□@#á)~6┌ö□□▌æ□□·b¼WbX¼□∞6%F□Fâ╫σ□tD>:▐□□π≤▓t¼$╤ïP₧ê!∞□█ÇpCó-
ΦüÇ~□⌠ó∞$Φ3□/á□c□π□□□%Ç2@9á□0+1'1-
0-0□P□(□ö□*□╛|QbIbYb□`UbuΓ╔─å─f└╙ëmë¥ë=Ç²ëCëúë╓─╔─Ö─╢D{ΓJ╥J║╔p2₧L3□Φƒ□ö□Æ
□₧□Ö╜;969!9)999%Yæ£æ£¥£¢\É\î≤□╨╡╔║d}rMrmrcrKrk7□{rWr/⌠

$□'[Æ╟ô°Æm╔
╔K╔Äö¥□Ñ"⌐d¬oj@*?54UÉ□ò*Lì├y□ΦΓTijjjzjf¬*5/╡░¢_¥ZÜZ

=╟9en□;ë{└╔╚îT□╞≡ô□ïOF

ânK5F√□□RδóÑ⌐M╤f└┘h~¬ë≈Σï9□R═▒2└▓T#x╕╟àφ╪µ╘ª╙$└îΦ┼H-┬]Ç├á█R═░╒□-
□█╤Rδ ├┌eO∩.₧╖ÅX▌kö⌠R>╞5╟∞èñx□╫□O<EÖGäv>D»╤Zαy╬'□g>#X╝Uz₧¼$□□ó∩B╤wqL░▐E/□
╨k ≈.z]îx¡}╧≡y±*█╧ë□C╗Ö╟┼KΘ╘è╖G?C/╨}└╧s⌡ß╝¼P°╛/╡¡≤□J□ô₧Gz□-┐

ⁿ▬
╬!b╘¢z╤êWßEg3≡o¥□·Ä\+X°Ç╡▌b?≡8}äi╜ç□·╡─π□ZⁿK╧╙π⌠<=N╧╙╫⌠<=`╧╙π⌠<=N╧╙π4é½N□
u╜H,₧É□\uzsùx╜Γè╥□·<-
ΩΓ*░╘ÇU∩{╚╣□\)½□╓□d⌡═┌╫u¢ò⌡┤√^nzò¡⌡¥ #╗6¬□¥°¼└çl¡ù□Lp▀▄φë▓:e╡¿B«ú□*~¡└shë
e√\╫□▓╛&k»╚╓y±}uè°δk¥▀f*∩□∞╨┐T&t╓ôO□»¼╘□δ╬δΘ╦Z∩δ╗▓fz□5╫ïf░:|□╫y∩Ω|╩╩«□We▐
╬╝▌A<Éÿk╟\5╢:┐ne╫□¡╬'ÿ█~Vv═≈«╬F½{-
ÿ-6½πkW=l⌠_Uq=║è+╬U□Åtê8L\╓Æ^"]J<¥°QΓ≈□OΦ÷πU·-<«5Wq¡╣╩_SµèsU╞╖ⁿ&Γ╖□s5Ö+╤U
=╓ö½╕ª\┼5σ¬½½╓á}»α4╥□Y┘=□╛ç░Ω¬ç░°_

··Æ□xÇA ╒║║□/_æ╧«┬ «"■7δ┌U°ƒZWWß╡║□ß:½≡-
╡║╫▀âòw±F╢z│ëc╓U╧'c╓╨#0W╟□╧τ□µ≡√╘d║╥Å║╫
ε"µ>üNz╘3o%°û£²ñΘ│φ<H
o'╝ìv^&τ_ê∩$g3i·cÑw#ç▄□α■£▒Næ■É╒φ½öU6≡╓□/:8ⁿZBùî!■║╞zÅ╫╫≤u─│ÜÉ╡□p4ΘG□k;9¼
ô.╓╜¼╥ÇsÄ²σ%┌1Σ□╤3/□╓s╘-ΓÜNæ□]▄g╓□û£□%εC_+mzlKÆ□□-
┤╢^╡û□Ä┐╨╓╝èd╡å▌,□□YS≡W'□Æ□`zÿ²5)µñτΩRBzw░&╒cÑI%─Ü┐εiU╡┐æ■Φ╓>è╗Σné£
╥?å╓╥J1h╢^J½▄_sφ╓╢╤¬□t"»╝>u□ƒ6}╫jÜ]▀φ┘₧ª□i╓╩╥ƒëÑ
╫w~òÿ₧|εÑ□*□α·Σ□

°□Γ╥.□y*I_ΘÉv~┼τï»[▌Γ╙¥□□ôεxü╥≈Jy;₧#?!⌡#▐x²MΘ¢║╣k

pë╘-Φ█╙∩CLZr□²╟¿S╙;êî╒┐æ£α▌!W≤7¥▓¬╒.)┌QG╞≤û╟Xε"É;lïHφ2JW□┘7≥╦Ç>╬╢'½]Å
G=fZx╟□║▌╚╩∩╖ÿ├≈╔«!ôö¥Bá?E□cKτ|Y┴∩îKnA□Ch«ñ7╦,┬(ÖEÇ₧
zó¼╒Ω□Yƒ╒gd¡□┤∞ :╙·èh╧║*⌠e]u□WN┐╔u¿3µ9┴□┐É ⁿ□□w,±0Ωl□√£]4╦jÜÖ(6uë¼µ
¡□B█;ÿV□φ8┤3ìtD■ù'Ñs▐,Ñ□²)╥(¥cK)£;Ñ□áQ□güö┬╣SJßîùRÇ~°╨Æó3^Jßîù▄ÜëR□≤
)□t₧ú□ƒ H>JaNJ)ÇQ□')Ñp6I)£ñö┬┘─ÜiÆR ¡gàûR8Ii□░π╨╬4╥æ∞èI≈Z
i╟┐╦Å╣║┌"{0¼░╒▀▓¡|K╔g╜x

₧&ú±X</■╬t□□¢▀{ù┐Y
ε□y□┐τ╣ⁿëñƒ5╨≥▀═≈g°▓■^«¢□╤LR╒iY╗»πÄ8□╝f₧₧ w¡╫╙7û╤-
+⌡±╘╟¡□~C"Æ▌₧ùo┼≤□σ}╙z4o[▐Å¡o≤
▌O╓□M»╤□tYVoπç╫ÇΩ▌δåtO₧Ω□{};╜√⌠á√]╒Θ=≡□╜┴╫M≤f≥s#╜δσ▌O□╫░≈ê-τú»å╜╟╜□h
sà▐□²║╤╓▀□VÅ╫╧πuδ±Ue╕╢-K■IΩ▒∞5╩[■□iT^ƒ□├xW»╧z5Qò¬□5K═Sï╒2╡R¡Q[╘

uP╡¬s╢e≈▓ôv□{░]`▀mÅ╖ïφ)vÑ]e/░ù╪╓φz√I{ô╜╒▌i∩│□█¡÷i√é}E□□╤i=P□╒#│□]ñ'δr=S╧╒
ï⌠R]°Wδuzï▐ªwδ□·¿>í█t╕ε2!□7²L╛□nF¢qfÆ)3╙═l3▀T¢Z│╨4Üìµi│├∞5ç╟1s╨£3ù□█
;}£□╬□gäSΦLpJ£⌐╬

gÄ│╨┌qV8
╫Zg│╙∞∞r÷;G£π╫□τó╙ß║n╟φδ

rç╣║ú▄░εD╖ ╘¡pg╡║≤▄┼ε2wÑ╖ ╟▌ α6╡║█ =εA╖┼=ΘPtsu/yû╫╥Kz²╜┴^üw╖7 ▐+÷ªxò^ò╖└[Γ-
≈Ω╜'╜M▐Vo°╖╧;∞¥▓l»ƒ7□║├@┼╜┤7─□□*▀□è╪ú║Ñ#,█m≈å{#╜╗A╡A>╨+□╒σì≡Fc-,╥!"╖î─≤□╜
q^□R.⌡ªZ┌╖σ≤ ùT·{â╜░ü=φà┴□N▐□╚æ□Z□â╜O⌠J└aΦòyσ(╟,o

R[Ω.wWzòá□╣O╗█╜)áµ║
ÿ₧φ╬t□▌uêi╗σnÑ7╔┼╪∞Ä⌠≤â□ò┐┼▐hw:¿IÆ╛7Vñεlë□*Γ□I╩ÉÄu╦$>¿B──*σ═µ□╘□─₧,ΘJÜb□ö
Σ╩Å+2╓ò_□₧ñVÄ║D■A═@]═÷µé¬é▌Θ╞U%Ω╣îû+ ╧Jτy
╜jPïpφûy+@╒zu▐b»□╘ro%Σ
áVy½qU7ÇZδmD|æ«├Un≥ÜA=φmC²í╓p╒╖{╗╜╡ávx╗σèÇ┌Θφæ·□╡╦█δ□≡&Ç┌τ□⌠·bεj{√╜C▐QJ□#
nï□□u□▒ÅQz□╢║□y}□≈░╖╫=□Ω b∩pO1ε*∩É{□╘ü-╥#ü⌠□ΣOc

τK7║ç|⌐╖╓▌HΘQ╖┴md║-▐q╢╙□▐i╖□OF╢w╥█µ.t½□≈╕;G«/rä╥z;ÿ½V∩$Ñêτ╡╣e╞≤1∩ä;
□d▐□O≥╥µ]≡.╤≥E∩┤w┘ô╝┤{W ù╝\≥:B╢╖□TW╚@□╦!+Σzτ╝°})lKN/{]░-
9╜é╪ç╝U▌qk╕πVK\∩□b#°!;□□E≡┤dçzàbí>╥6B±É□□{╚i(□J".r□Y/─□□*

y┌□
*╔╗├EΩméφ|æΓ┌_± ·Y┤□Jzq╞φ□Ωçg7;ö

⌡Gk∩□╒74└□ε╢âΩ□□□□∞╢ü□□□Γ⌡sOÇ╩□

□╕GA
ú⌠□·Bô█∞εpwâ┌ë□□²

╘^w{¿Å╗E≥∞-ô~&yv╧╣½▄╒R"≈é[δ╓ür▌╤▐9w⌐ö□#╥)wæ╘□F°cε\╓ò
YεLP□Φ²{±▄,5ëvΘNµUêím□ü║α╡h□c@¥⌡"εpw$¿3₧q≤▌ íáNa⌠δτ

ö╢üæ0εªy}{ßΩGx⌡πNùk╪6·:φ╬□╢ì~N¢süφ¬┐s┬9
jÇù∩□uZAφ┴╪│┴9∞≈□°╤H ▌═▐p─oô░╛-
Tï▀▓@¥F╛╨▓ÿ½só□┤½¡∞eg╤▓╨>ñ╝╥vBa □·qσΩï▄┐╛~¢älè▀┌)¡÷û□╥²╥&┘6·zR≈ru┘┌y}╤ y}
ô╥ A□┤*?n□¡c╛╖êû/·ú□Z╒Yî93σ□I¢≤╧Ö+ÄYî+)GétK▓mR╞╗l╗□í╞C²Câdö□5□ûç░M÷B«□░Mª
Q□φí■~½s╖╢;²Vτn┴32π║ù┘&Q─┤¥v╦°vU$4X⌡U□T>8╟ë╒ü≤Éè( O» ┼>c=□-N■ÿM-
□≈≈ue≡De}□╝□Oi"5.Ñ?ô╝τv>=ï·ócåeΦ»Γ>╧Θ┬Q#╝[·╝(ⁿ├▄òY+ⁿ╨

±╟ä-òoU║u▓¢

╓Aδτ╝╧╨|□≈Ä=4+ü┐!4₧è≥á┐_ ╘R╖Tê5w▓y▄R▐}Ö □Θ□ΓQ6┐╤─$╖B█oôT£±▓█□uì°v≈v≤mpvèÄ
~╓┼ôƒ)=ê□·
Γ½VKàoτw<╧r╓^╤ⁿ╬|╥;σYôσ▌╨/zä─▓«æg2=âOfç╣╖}v└▀I>░y'su/≈î▀┐┐N□
₧∩¥'
«æ≤3≥Gk□°p ·

·□#╖ï╠GÑNd≈║╣ùv>I²╖□v₧á■√iíÉ£Bßê□─╛'α

á□y╞¡□
╬rzâ■ñ_Σ«Uy■^%ub╓╨«F5S▀F~XvëΩ@┐wF|0δd╫í²ε╬╤╤α╧# 3┬wåÖZ╪τ-
8□╥&τÿpx▌╫Θ!Σ▀╞Üç╛√²╠Oä╓kI?L╥╟h·□«c╚╠□■oÖ□╥≈ÆPtsA¥CáoòwA▄:▒

z/iö┼□æτf≈÷ ┼╕ΣPΩ═YÆ╡C ⁿ□r]L⌐╘òèh□⌠K≥∞«╢i±E□Æûl□┤□╔½÷;iu]▀□_□ΘAl·δ┤╘╞
Yï0ƒd¥[≥□n╬è&hßO5□ü╛úδ≈└ú⌠_x□┴1□¥b╨□b> ;▓¥»q□ :╜é5≤□K·
Σ□«xeLç∞╣v┐┬]║□╬#bGv⌡:
dW/≥·S╔â9ÇX≤y□╫i±╧╜`┤║#╧≤╗Σ√5□ö∞□«□┌>/φD□)αúUδ7□±UO$²□[ToæÜ▀ê╫╟ⁿ.╨ⁿ─
Φ┐╙s┐ô'□°ÿ⌡·□±╔Ö&╔¡╣â▒Üî╝╗│Q÷Åδ□╞¥'qφ°Θ╥y□≤Pεê»w□·?σ√L¥╘\FN╡x^⌡□·┐g⌐ ░Σ=Æ
▀æ/;ú▀%|√>±b┌ì|╧π\ÉC╤löÜ╫₧≤□q%φ▀╧X╛²□àoΣ═╢ç╚╛#□√□□gtëX╓ƒùò%╨ÉΩπ≥╬ôn

∞K▄wÖöîE□φê⌡0╜Y')¥E;_□

j□╡m╛.φ┘□,~#│ÿuRfΣ
¬Z-o╫□ëƒ▌~è╖\+D▀.π[□φ,iÖö╘~□╦X╦2╓▓,┐ûr□
²ö~àΘJ▄ úeO⌠/e∩┐■á╝ΘÑ¢□y+δA│GZÄ¼≤╕δñ-
⌐Ñr}╒wÑ¥t,╥□[:┌Ñ'v□æ■nY╥▀;╥6F┌╟AΘ╤╓>Θ╤□i╒.Ü▓ÄgY┬WCào}_z║¬ôPtsn╒K-
W∞ëj╖⌠k╗L·5¼σ3□YYjù:□ a┌ⁿ-
mPts□ £⌠q√Å╥╟A▀KZ▐□¢$╜□╣}å⌠^I]·╡z┐p2□Ñ□Z/HÅεzVztfì⌠¥┐nò□¡□HÅF■!═╝Eztµ□æv□ßÜ
ò%²┤δ%í3ÅKÅ╬<.╡j□╨ùV
Rδ□┬╓<#╜╪¬ö^ⁿ╫╚R□V╜⌠e╘Ç≡≤ñ/#? ╧╕▓#√è⌠eö·)ûÜ½j╥;╘n«╧T≥²ë▀J_╢>'+ô╨æ1·²▄°ƒα>
²{Σz┘e╘╕ñ_δ\▒-
jÉòÖ[*ñ╢▌╣l▒┐≈ⁿ?Φ╩□6Fa&b┘i<PZV·¡i±┘)knnUεg┼╙╫π█╬ƒë╧ß□N>□|σyQ|[ⁿ╟±φ±g²/='t
┬$□~æV╛B□|□Ö_|^É°"┐°ⁿìD=┐°ⁿ¥─F~±·gë=ë_&

$~ò°có-±°—+ë÷—ƒôvR'M╥K╛/y□rJ≥ú╔9╒/$O~9╣6·╜ΣÅ°□Φ]┘»▄□▀é>ƒzSΩ-
⌐□⌐ü⌐w±□,□ªf·□aI═N}:5'⌡┘╘τS
S□╦╖Öσ╗°⌐═╗■√¡▀L[Γ┼Tg^□µ¬CΩ¿jU'╒□uN╡½+╢Tö
□□□□αZ^□╨0(á»=□Ç□├□□ ╕;Ç▒□L□LΩ□N╛□L╣□▓·╥µ½"╙G6╟┬

L∞ £█□□□░8ÇÜ□─^–á

P□X□X
╪□≡%¡-Ç¡=┬φ╫└«k l m╜f≤╛7Ç□□□

@∞┤□⌡3!(╥±

lß□╩L╤ N█]Ö□+°#┤uWWç□ⁿ∩[╡
-
}Tf-½║°.-·E╘lσ£╕òq[╓ƒ£□E\A╝çδkBO≥1u>O|û╕Äⁿ:ƒ□√*ß█!~╠╟╘yî╥»0¡}Σ$ÿ╧V≥■!□|V∩
ΦÆ╖cv√y╦puÆⁿ█iτv╞*ó~Q□WNÖ├□·╒üT,LªeÄ□Ví5⌡oF□█r|Å║|#┌╧IoO∩╡óΘ□ΘsV?z╤∩æ■cÅy
Mx»Uc╒Zux-Xc¡╡6Z[¼¡╓Nk»u╚j▒NZg¼sV╖u□âì½┬*«╥¬┐□ñå¿ßjñ║[ìU□╘$5YMQ□jåÜ¡µ¬□j▒
¬Q╡¬N╒½5j¡┌¿╢¿¡j╖┌Ñ÷¬□Ω░jQ╟╒)╒ª.¿K¬╟║±╚□▒σ
┤δ╘&αFΓM¬⌐¢│\¡□^¬≡∞¡V¬åÇcT□tEC½Uj╡z□£ÑÉ4BC½%α¡□gÑ»à8e□O~¬Dß
OMT%└#□₧┤╒□5-x░□A

₧≡T?5ÿ£a└1 ╒□╕ R═□₧Ñµt╙cT%≡h┌,'▌Sg

Θ9Σ╧ó╬,ª[╓t+╒D╞□O<åy□M∞τaXw>╥¿_&·V□⌡'è╛╡£·π⌐?å·cÿτ╤rçÇ∞,±iΓ□Σ│ñ╓□ΓâÆ□₧'%¡
┴ñw□7□□│ÜPk├¼qV□8F
▓Fc╢3$█/±─"my□ g╛@h·2$9□Σ

ûτ; ▌┐╪ç⌠s■╙£┤}#□=┼s□Ñ∞Oµ

=!J{╙╬
\7úe□æ╢∩≡□╞╩ß¬á▀╫ì¼▓Üÿ▀½H╥□K±

B╥∞[^ê⌠ïLe□sRB²}╘□ë£Cö

"n!µ□╣¥Qñ7□ $H□⌐╗?$°â□ÄLµôéoΘδù□╪ ≥≥p╚k□¡U╥oπ╣±dⁿ╔x:₧□╧OτñcΘD:ÖNÑe/╟█«w╫ε
╛3g∩╝»≡^δ▀iσ.+w╪kε»⌐)⌐Åªªª>û*gÅWûï□Γ¡ΘAΘ█╥·Θ█â£╔5┌□ΣLvd

è╟π⌐x▀°p·/Gⁿ■xYⁿü°ö°GπSπïù╟°┼+ΓÅOOG╥╣Θ>ΘíΘ┬⌠=ΘIΘ□Ñï╙─
NùñΩKON□$]Ü╛?]ûû╖╔▌~▌·ç╞>0≤°çö╙ n╢╟»O⌠(╙δ┌÷?syeⁿ─
l╣V!εN≥u≡□╙╦1■□5╜╥╨O»J ╪□┬»â□╦ù¥σ}7╦Æ≈▌¼>≥╛¢5D╛u»+ aáoJo□`

`>`æe╥;□Z▐N}╡₧╤╥^□V▄@■F└*╥AΦÉ^▌═┐╢<C1╧¢╟c□fi3□z6fc
□z1á&ák□u□]□
╜&á╫□6□⌠□└╓Ç▐

╪□╨{□□□·0á□│(íO□┌□■□└%╗Chm√<□□⌠àÄ□ÆÇ╛┴·□¥»ç□tü-Ñ
Iì╙□ÇïH□δRα⌐W╙╒╙□}▒3SWe╙╥≤⌠B╥╒ÇÑü╬r╜R7□tú^°7□t□á9áw□v□⌠>└┴Ç>□8□╨'□°⌡Y╥□□ù
□~ù=┘□ƒoz·<□≤⌡I≈□⌠□

╬□¢afD@Å6c╟xR□M p□ΘrS <δj║fNá/vµ¢E┘┤╠□│î⌠□{2Gá═x₧£e=□∞bVÑ2_┤¬Çç╤ç╪«²¥└
─_ª▀╢ δß╒¢uM≈}%û□oï□▀%°%Σ4æ#^α+ó⌐╛
q╒P²□Θ<ε╙xÄsc'√⌠Φ~E≥α■O▄°öwƒ▄¢¥+Γ°0'Θ┼√│x□£;ΘüR▓ƒ╓¿Lï≡σ¢□^H₧ü⌡s□<═Ω┐ê╫Cƒ□
▀+ñ¡É■K
□o∞:╓í{^0tZä/╣2}3o!~Y8≥┤l·è_□°╕<c█┐å¥┘ΓW╡╛oτ└┬□{8Ñ╚Å╗ZvH║τ□;?ô=fε□Ωïñ╖2⌡ï
L╜E╝QR□NRj

┌/ÉSBNô`⌐▒╨□Ñ60/y»`⌐ì╨□╬TåHm╕[ñ6BoÆ┌pÅJΩ╬x⌐
g╝╪≈╛$╡□·üö╫ⁿUj├╣¢╗âεö▓C┌J■IyzùtC√Ö·¥¼╔iR□╬i⌐□`▒9MΩ┴9-⌡□∞╫C>µ□▀ôzp□êª⌡}u
∞îÉ┌pÆΓU ¡Éz□φ□î□Q□í▀K
á÷6IYäF-Zxù╧ mî

εê⌡┴▀KJ-ï□,□ö ▌ìgâæy√≥~a╜;∩X ▌1δ=|ÜXò ▌è╗ ╔Åpæ□ûv ▌Σ╝O±

╗□wX┘□;╖ç╜┐π√╒□GNFYeû∞q£≡ X▓□╙+`»

≈╓┴»c▀X≤0WK[╕vV═□YYo&o■sƒcσ½□╘╓■□Σ╟|εSⁿ≈Ä%≈w;
Φ√□□uSz□□·ÇíÇ□╦╢G□zΩΦ□ⁿ≤B╦╪π^□èn □#á□P□╨S╗·!+Ä½RêÜ¥eUú-
lFm_─╤e]T□╨K╬öⁿ∩╝┌j┼%iò°c╥xB╞ôT□╧╧r┤□Äô:E
M<ÄYù□₧ûg5k5Ä3Ç═Çs<█□n
ÄπÇ-ê)t¢╡
▄c╕ú╚Q
╪Mj9╕√╤;ΣXégv w╩ô□εD⌡7}W┌≈□≈&½√

┼æM7ïÅ┼╬τ╗□╗3□┴ß╕çQX|/[┼δ¥9╦╖BΩ(σzÿU/|}ä▀ë□'q⌡.í¡zY'Ç■Dz┤e]φδ\W½□∩6g╝₧Uáû
□3▐òh·½╥?┤åªƒA√ƒ╚□∩ç┘
Σ?Lû⌡□┤¿å╫□√□≥╫□}SzìÇuÇMǪ□Ω+╗·5┴╝Ä∞ì□□αvƒ∩Φµ_¢╟^□wG`d£c¡@K┌o¥AKù ╗ò¬⌐ÇΘj
ª¬R≤╘BU¡ûéZ-°h¬Θì)□.╞ß√aè┼_C╧N□b5¬□α□□δ□╗Ç1V·■□h$üô8$¥Θ□ÖN╓▓uI-
╡.□ÅRàj□┬"X/à4¢ôàV
¡╡í□f□█ùY█U╡╡□X╥ !lÉ3Z₧

Θ□5▌Z□∞╟^□K8δÄ╗□q□□/Gj□N□

ΘTI┘¬@

+Ç
á%aíα□÷┴l┐C/3údσ█|2≤□ε(□·qÖ▌□3├g0÷({¿û∩ⁿlß{FE╓□⌠Ü>□·~┌t▒`k·π£□ßzm½ⁿâ╞i╒∩□
┐▐>A»ª|æi¿╠□∞s□·f╬$Y}Wτeƒ9b=A²█¿□□£Ñ▓"Φ«ûⁿ╕3e┼□£ƒô≤29ΓO)e>?╠<≈ò]╨f;±╖Ö█"╔
¡¢f~^`~^□≥│àÑ□éⁿç%'╨yéR?⌡FJs┼ù─<|ïy°┤Σ□╥uΣ╝L╬□jÄ□F

C
δ~F~&╕ƒ~(op ▌□V    G

Ö⌐½⌠□îÆ□ε⌐ (P7Ñ╥□Ç·å¬□╘▌ä>µ$j═δ└┌□╚▀□╪hiå[z≡╣□╢ m□≡»ÇS╨□fX│1ç\ÇPtsRs□╜ß╡εEr
7½

ΦJY°û╒UτO─πe7ê│D╓w:╡}.█c∞|«G╟Θ}□[□y?i□/ τP□█┼}l□-
ß ⁿΩWΘsαµτσC≈□╥r÷²├└╛)╜âÇ#ÇcÇ□û╞╙ê@Vn«╤wΣ¢f╬┼╫ü╥7É┐□╨e╣□□ö|m,╥w╤≈▌╪▀ö±¡ÿ╙╧
@╗Xï·=qεÇ□à1□-╞µ&uP¥╡{┘âφ±vÑ╝─~╥▐i╖┌WtZÅ╘ô⌡\]°╖Φ□║=äL╛□g┘fª⌐2≤╠B-╒f⌐YnVÆn
0
!⌠9Sy¼□»╤¼#╜╓Làt:c□≥X□─□z╣)äT╬□t╛eí¬╠Ç@jw╛T¿□c√ΘΩS┘├□ëöTü>σ╟╒{│ç-
ùTâ▐□─▌ÿ=L─$M)ΘKz#ñ(¡«=-░\

⌐PSu-ñS═t=;{át□ªè⌠v=□╥R3NO╩-┤\Lj¬₧□Θ8Sñçw⌡z
ñB-╥├!□e□u<{ÿê▌

[B
╨qHçÜ□╗={ΦßóMj¼▌

i╛□j═─┤\@jú}□╥╛ ·Æ╜═▐f□□╓²⌐─ökcFlÆ║┼^m»6}□k┘4□□╩>c»╢l}▄╪÷"{æ
╢O"_□ä╥╡╢x□[⌠)╗▄.ç}`{Å╛á□≤|╢].q⌡b{

4╞≡Xñk⌠□R+∞1É-╨□÷@Φ□ΣQ«g@[¿9÷@H╖δ]╢╤{â╥?□φ≥□⌐□█╙_½⌠ZuZoW°σÇò╓z
⌐□Ω┤ⁿçLoT√▓ç=╟-□⌐P}□╞□⌐□╠ùéC»╡Ká-⌠eY□╙uÿât÷2─¡'}

┃#[/╞l⌐√@yjí/T╜¬ó┤°ΩaO┤√Φ□╥ÿúA:¢┃▓α░ç⌐╤·n╥ÿúA:û┃▓α░cΩÿ₧@□s4HG╩¼,{Φ0┌EÜT□`
╗╟\

ç:ó□┴□*_fg£àu-
¬YU█OÆ□╔∞î│░ε├>úª█KH╖╔∞î│░½╟)î□{H□α□┴M<□e╢▐∞}GOò□8µQy?▐□7▀╤▐k£╖╚,□u"o║»π╫
:wp?Iös╖0=U□Ö□╦;a⌠□═│2╘ùoUεûoIΩτΦ□╠ú╡ yÿÑ)g▒□äQ≤╝|++i?LM·ó≤l√iJùA:X|4É■ê╥√
)╜╠t1st?G╧╓r>╖φµ≈Mk%ƒεz╜□x+}çI╠□E□ªT▐╟═ô┘ ûwï<╔y∩û□≤n□ "Φ(≡@y{ █ ╔ò»ñ=¢oµ╒Æ
□□πè7±≈≥

"≥÷□mû╓J□▀É√-w√öë▀K√;¼-ò7 Qc≥□ⁿn╛□7ê≈⌠=╘<'q⌡e∩ì·□ƒ ╫I
¿:{ú∞sô/d¢┼Æ7╪yT÷│□:(⌐·7≥os«□□É₧╘█╕3≡â2Cpzso^à° L□ƒb□HZα╥█x□óc~╬²ô≈╨ⁿ┴▐AΘ
D·M/qTRvRí∞┬╞□Yτñ-£ëÆ+ò╘6k⌡□┬□√¿δ╧²_÷₧□0¬ΓΦ┘}∩BÇ□╟╕ww∩6ⁿ ñH□1"F─ê□("□"ó
"ED─ê?EEè□êH⌐EDîJ#"□DèH□□#""Rñ□□1RDè□□QæR>□ä╝of ▌╗╤%╢▄%åX√s¢¥╟φ∩∞∞∞∞╝╜╖╖╟%
:ôx?τ╧%Y≥Σ╥□Ñ.ye╫ñp≈╫₧t1╗╘│∩k╨«ö%K╩╛▀àZc?á5[kTìyY⌐tc╨ÅE?-²D╨jMe-
Ä╫╥ñ□╡-ëπg%ê?□■)≡α□□πsú┬±┘ó╓Γrφ;□ƒÇ°C-¼E]uXñê4╤M

F;eåX$╓ï]Γ¿⌠╩╣2
gòqrû\"7╩=h½ÿZ□¡°6\¢Ç┌|Ö╢□g■b▌┬╢╕╖>□g≈9·□╝@▀∩æ₧F₧╬₧~₧╤₧)₧╣₧╒₧mh╟I╧nO□╜┐Åε
¿τ$ gç1√=□=G<àΦN$□ gç1▀x

{╓╕εX□[â1T╓¦╫□è`°─α┘σ┘δÖµ‖²□1□╞1≈∞⌠îq]A□├□─┃╤┃┼3└u+"□Jñgëg╣°ïδµD░.□3╫ôτiµ
‖┼□

-(OÄg║'╓u╣□,        cªyfΦ▀8╬3;îΘ─¥ë·□}¢π<π├ÿ╛
c▓=wΩ½□τ□□╞t┤
<#<ú╤εcτ□□╞⌠╣□3P╧╒°ΦS<C╨□'╠q□╙Gƒ¼Å╓G{□á∩Iÿπ0ªà~□m«~₧6Φ;□µ8îQ·

╜IÿπO&E≈Ωì<┤ù«æ╟"Φ8î╤┤ú║⌠╘a3ó╕─₧D╗τ¿╛B█Å□j?┬"éÄ├ÿ²·□¡@?¿□
▄L╨q□│□¡╜□«;□┴≡9M▀¿o╤µ8No□╞4┤á⌠╒┌─░╙╫ûα□│□m-╫Θy%8╞Σh╜├Nƒ^éú%É«O╓╥□°O
c□Y□VëCï5éâö┼%N∩Zéc╠6■¡│H□Θ▌Σ!·∩8▓7╤╓Kù¢σf=□φ╠═açy÷╚}rÖδ╛ë`╥0µ□ûæπ║ú□

ƒ«Q□a√æ▄«□6┴─m╕ δ╛ ë` ├1 ª□╦δΘ║C□¼'╞∞┬'ù

«+î`□0fô,Éªδ╢G0┤áΣ:╡Q£p£╞□cΓ□≥m□Z~{─-m░╪#╫□µ8îΘ!vïìbú╓[löyä9

cÜ╔>bëXóÑë%r:aÄ├□□m╨YbûfëY≥v┬□ç□î□┘b£ªïqb£□K╨q□3L⌠□Cσ!1□ß`éÄπûzEû▄+▓□v#Φ8
î)□GE[╣]┤EÿF╨q□╚Yl□^╫⌡Ä`^îi!█ämH11╨óö╚í]a'U   Ä1I░>∞$öα
┼aX□vhuGp£=gê▌0├qó(îíô0╢─ëσQ▀¬⌡+o┤□)▐&╦Q╛Bo$╔¡⌠Kñ╗b±èΦÄ°2A°║ R├r┤d~kIiX/>
□▄ëy{╚Fê»!KD{êbßF±"ªΘOo»δ⌐□≥.┐╤□Rú□□>°□±ÿñ3□.µ_%²□ΓÖѲì,2. □▓Θ⌠!£≈□╬√□[s?
ê⌡HU1Q½uqδ║□CÄδd□═ß¥1à□~?╤á╒#¢T~╟φ□@o^πsσ~╬5□i8Dûæû╠6┌ïdù╔7h5□⌐Ñ█AV9wEÉ
%ï9o-è╒□znf£╬[▀ú}ï°5▄÷l>qπ□:ù□9╢□±ù⌠█╚╓÷\Do≈ô
ê¡>□± φÄ╥«$~Ω╝Öô[ëôr5╫▓êΘ□┤es╔═╣▐3ÖÆBzg\╗àW-┐Γ[□n!J°n∩$╚á1□₧≥oΣ░Ñ╓Å-
╡■d⌐±Θ□□□□Ég5σ╡JÑSΦ¢íOC▀

τ₧t÷óL:▌ô□╟g&ê?□>
<°▀├x»¿≡~Φ╦≤▒□⌠Ça0-f├R|û█
'E@t□╜─□q┐xJ,□[≡╣□ñÆ¥d9JNû╡rÑ▄*□h║╓D╦╨·c╧O╒µíf\ú¡G╖
g╥φ┌N╞╫k{4z^▀╞½/;┤▌h╟□`ⁿÉ│□úφ╒fá█»□╘Äh'□╫AO╥Pô`9c╤□hç╡cX□ß√┤o┤▒□Sñ
fw□²F╞

a
t□≈.¡□╗╜Φ'3╢╦ñ╡êφZ□╗¥Φ≈0FÑÑ±□G□╗▌Φ╟1F4ª`Lí<LN█à~
c╦╡ ╒≥0╤å│*:¡□}□c╤┤òr□¡j╚╡Σ┤uΦ█2╢Q╤òk1f╣╟#°-
D∩elë6Yµa╟\zvö╙╡<äG□[¼I□AR╦æ╖ô├□n╟Öé░·Zy;╞╟É□╔i│╤{□¢ú5Æ□1f¬∞JN{□ß"╞fiJvÑ╒
m□¡┐h╙□εblª▄╧k1□ÉÆdÖ¼▌Äp,cwπ¼¥î1╜┤1Γá8¿ì□□σ"╞╞╤□Γ ╞⌠╘Fèφb;╬¬█σ

╞å╓9é▐ò═□·Z□⌠-D>╬â⌠φ□9QΣc₧,1Oδà>Y╠├yp-bÖrñÿ╟∩)O╒:ú∩*ªΓ<ê▀É┬▐b*µi#▓╡

Φ;ël£□│□«ùΘ"¢µh£U╤ß□▌_¼e¼□╬î²╔R□□Σ┤&□≤□k%□"□c╝óI─M'¿□─I╤□c$╜╬π8ñÅ`}═□┤□□p
∞─@éZÆ╪ä %╧┬╓░□] ╧M¿M╖b╟□X□vó□A╣
ƒIWb╟å¿╒ÿy

□╨%╝□39╨□$ê6╫ ⁿ/aTö█NPµ┬-□u□g╒ε·²æÖ┤□ßb(²2
^ñ_□N╬ñ╒àôGΦ7üôKΘÖ╛°uz■åtÜ▌∞/iE□εú4╟ç╨╧═╒¥╬-&┌┴c┐╨OτCΘ9^┤áU□╤Ä╓o0σï┤╗╙┘k┼
'!·£XZ┐üM┤~#f╥·═⌐δ(%|F≤#å⌠ú│ûΦ='┘êgƒ
ë~,
k,Níy╟┘S%»íu□y□¡╙`°5\╦F▐G%h»□±J~Dδ□ê7α▌Ht▓ß'─üΓ·ö^^Φ¼╧≡ÖPCi=µ╘[╝â□°£╗□┤Æq
j□╡╖x;¡╟╪□┤-s╧`;Σ>µíÅ╥□gR.;âwAàêfΣ╪π▄«{x▀╒&▐UF╗▓╥▌╪□¿ì□k□Ißv.qΘ╘N.τAgG□ V
s□»Ä╝╞{╬F≡Θxùqx

çä° ▌ï□ε»□ª<ç╨wvëa□)Σ>Z}9⌡Og □Qr|□╡□╙;√┤ÿç┤εbgp·□▄∩'h▌□⌐jF▓çP├0Æ□zç*ùσΦI:#
)≥□P□≡□

□j□ªßç║F╨PP╧hl4çåFKú□ªL3╥└2┌□gA╚8█8□Ü□τ□□╜▄8▀Φ□-ìLπBhMo├/î╦î▐på╤╟Φ
┐4·□├í=Z□≈Aª⌡□Z□WàÄàN┬═íbσü1lA▄═┐Z▐╞░ÇΘñ╖╟·±
N;"öVⁿ╛╥$k□⌠╖-▓-é+┘:□└╓╔°╨1¼a┬╧┤ìéF}□─╓â%·EZΘ⌐¿ò▄Ü■?¢╓H□■DtP∩fr∩^╚╝
█ì{w$╙4èi║╡□oI;Yê═□ìDαg&δ□|?
□\w&╫}ë╦!τM╝≡o├i□ÄI╚□:≤Éx5≈g╞»°ök░□▌Jεoß₧□±∩B╗Ç<µ╣ä┼?S«#

□öí·íE,'√╨ª□¼ç,╪·ƒº¢K╖V

□⌠{EW━7Ç□² °ÑOÅû.┣e┌□(@!>-
¡}δg6□~f·‖□∩H√¡·≈╙~Ñ┌á≤zσZX═2α╪¿ n¡p╬+╒á□╨⌐╤½╤╓Xδ-□□□ΓêáS{i□φ6δ

δak□╨ΩZªδ│░ÑÖΦiÅw□╓æ   -|▐╥@ù□□3h╧*┬~00ⁿ4ë╨ß□⌡=êo├╧à°GO9□≥yI#Φ≈r{┤ΦFO-
|≈_□□□ΘTTj□╜g»YOX╧Xy£┐>╢á>>¿╚≤h╚ε┴≡□

¥9kÿ;geA Æ╬ox
ƒ□0RìFF
ú╡ænt6╬3║□□□▌ îKì^╞σF?π□π□cDêN°Nß¥àúìlπVcî9▄╝┴□a ▌h ▌dÄó▀â¥╜□µ;µ>▐[≡WΦ┴{
·±∞s1

σ'□τ=φ°½X3ûÄ5ÅFë╨6╞Çµ∩∩-
□I╝╤á╛ [s□╫ⁿ□□\sK¼y□┤τqF¡uZ■S╫ì¡ªwM#¡₧Se~_o<j╟4-3f□Å□9╞S╞<▌├□⌠Ñ ·□√Ü°Ü ·Ü·>≥
□ ·■µ√─w╟w▄w┬≈âÖj62□¢M═µfK│ò┘ ┌L3█ÿgÿm═_Ü□ó√╤ⁿ¡9┴╝■£i╬27ƒ4ƒ2_7╫ÿoÿk═7═πµ)╙

£Φ□╕ É□╕0pq
+╨;╨7pEá□``αΩ└á└uüδ□v□é"¿□=┴Z┴Σ`φ`¥`J░^░~░A╨□╝□z?⌠aΦ╦╨~zú▒<□ÿ3CQ"J8≤╟¬≈Ià
|╟±ß?Åδ□]¬Ä▄*╫æd▄d▄ä²~çq□□«Or}&╫╫Üδú╙α=µ=µ8≤^s╝⌡₧+δ├óΩ}ª╩╜^╧╪c|f∞5>7÷□□îâ
╛W|+|»·V·V√^K,qÑ╞┘▄╙*δÜ»;Å≡B□ßFΦ]□█-
cÄ░ƒó╓▓cδ┘ ╩▒□¢3╫öÆ√yUεìXσûî¬╥Ñ?wÜJÅ?åµƒµ1□¢sé÷□@□pV┌□₧°▀╟î≈î▌êú6ê-w■I■·╨
ÆG_w-}}y□□¡□·¡□½üeXA╦▓ÜX═¡

V║uÄ╒┘:╧Ωju┃.▓¼_Y=¡┴╓╡¬.╫i║u:oÖτU║&ñ═≈'▀rμP╒Ω$ε÷ÄΓεé*≈í0n3╞1WRì□╞

□╣|ì'0⌠IπI╨î°ì98ósìgQâ>g<□uî□ì┼╪#Kî%╨└Xj¼□»▒╥X□A≤1≤1□Ö|=□Pµ□s

46ƒ1ƒà&µ1≤□47Oÿ'íàYl□Ck?°kCÜ┐«┐─t⌠7≡7Ç│²
²!ΦΣ?█▀□.≡□╚ÇïBoå╓┴âí⌡í╖ßí╨╟í□≡phW¿□~□╤Q}ót╘□~¢%∞/5R□V╣┼

φ━~1o6o□α^>Ç²¢□╒╤ ·m□n
╢.□^¿▓ªlï-₧cⁿ╤ÿk╟7₧7^6û□»□½░%9╪égæµ-
╠ôf1╥+ⁿ║▀π»σOF¬Sⁿ⌡ÉΩå~├∩≈□ⁿû?ätw≥ƒπ∩î4»gZ±⌐╦°·P÷|o√6π│K4Ñ}ÿ╥EUªT7₧0□#wƒë[
·p.²┼*ù^ƒ╧N□□~║ß≤Sε4╞□w□≈□┤k¿╢⌡¥u▄·┴*

ëÉ'T+ö□¬├ú¼□∩)┬°3▒╨╤┼U¬uN)+d₧æg,0□□/□ïî?□»║s╪>πáq╪°°q─8µ□▀p▀
╛Q╛¢}ú}╖°n⌡ì±²╞7╓wù∩n▀│╛y╛τ|╧√≥|
x╢{▌╖╞╡`■ε█π√▄╡bN·è}6Z2a
µ

≤L≤\3├<╧∞b^└V╟M([ú═1h┼<└6

Y0O¢□4τÖ╧Ö/¢╦╚å±╫±╫/e┼86L╪é╔

▄□□□╕-p{α7ü√□□□ô□□□ª□ª□fáe□╢hΩ□}┴╘`ú`π`ô`≤α/ég□;□╧□ª□╧ ₧-
╝$ÿ□∞-∞□╝4╪7xu≡Üαáαααu┴□é╖□│â╖□╟□∩@«╫┬9≥b∞ ,▀□≡·□Γl▐□τ╦φ8╛□═/
¥g═ïy╓□┴Oⁿìúe DÎg□Yⁿ┐▌·Izï╞╬!δäu╥▓C□╥BIí┌Ñ√âvï■¿■¿π┌∞ì╤^°ôì╥|φ|┤"ÖΣÄ╒S4V#k
ª[πÅ⌐⌐₧┐╜┐â ,□z

-Ç╧/°╟r%Ç * ╒
²â≡·┼├╧/I¼!□¼ôh=%δ'»]≡╓_╘rτ6□┌□ÆLkqt╨öσ╡□╥IR□&ì│,zv╓├'lr^ZO¬∩«OΘHφ╡hc╥Z║4
WÖδ0□8ÜÅφfgu½T*π<δ□^'l─+W─√tñ╖□tß⌠tçáhH≥æä□
┬|^ñ╞╓Öf£╥⌡▄∞°s¡$╨óÅ)▓╕Fgv_J·
Z╤Kós<¡m╓ç╓vδ#≈\▒VLWIJ═⌐├═C{□╢aZ(WΩ╥æ□-*□·┌╘▀î≈`~□║Uh}é╥╝╟·□□─¿τσ□⌡h£□8°ç
s╓-
Wτ▓HKä╣┐\∞ƒKb²⌐n¡═╕w£°σ╞╟1æT║y&Ä╫≤â7░MDw)╤J▀¼2⌐ e½πσh╘▒╡□≥░#K⌐Cσ+□┘(ßgX*Æ
Y½Æ6²¥o║∩Q▀╟□╧X□°î!h⌡8║ⁿyWï ¥⌡≈?Hw│v&▌□¡Ö?f═ⁿY□═|&jß¢]φ√4j▄W╟W·)┐ÿ┤.┌Hà■»
╤ÜK Xü÷ü.«□ε╦zwhα□W≈Ndm√h
7≡τ└ε└?I╓Q╟₧Å·⌠åα│┴□é╦é½éƒ□┐□-□₧░«/'□»Fx┌╨□Y÷ⁿ\ ytj-
╬âo[□¼w¼ì£¢$╢□äƒrW2W□û┴ S¡ ≈╬)£3«
╠ê□π-8⌡*-¡
▒»»│ΩYÖ□∩[7ε,ùnu¼t■Qσ╥╝□│╝█Ö╬╢.ì¡8e~□)1□{Θ#£9Å╤*Mî£»W@3¡gô

` s∩□≈╫ß-┴■αR:$,E#ï╤□□δMΣ║╣╤ⁿÿs╢KÿSRN╓pß<òí┘╔±ÄY□#╟Ü□[Ö□═ƒê&-¥√ì□╣[
s ▌╖□mÆS(±□#∩┌□k÷`╬Æ|□╙;<u$≈═╕□¡Å▓▄□σ□Ñ╪=ï≡╝2r\║□Γòµ£╞▓□┬T┤ìób]à£÷αXjφδΣ≥
¢≥╡ï╨≈V∞^e┌¥s¼9oîv»ÅI)>m°⌐╓L╘LñòJk$╥x#Yτ▌□╓z¿¥H7æf┌╔Ü⌐□5S1δ"╥CñâH□-
≈»(úà.p╡╧ÿê╓y#░ì4□kù▓φîE¡4ngZπìƒ▓¡~;n½⌡░╬ì╤C□*ö/□Ñ·s▀□□Θjù
ùtk╚╩ªm□□□ú«w*¼½□Åó□4èp

⌡¡R^□╟□Ä□-yeΘîòK□_Γ╥╢±GΘ┤▓Ñ$^{n}$Ñ┬Q_Vg━jo┼╢kEk

╖╒□<N├y7┼╙[╛╫≡£!ís┬▄eδt≥╥/°4[vπS╘t╟C9hÅjR

½•=k□>;∞╡÷é▓è¼²Éèv□ÜdÑ|3┤c{B□Pε

æ▓°Uτún┘□[`□∞Ç▌P□□α□□àôX7¥╜%1v]□≥Y?ÿg□┬ÑΦ~*8-
¥t≈□U□å╧▐rnµß□x┤┘téíε╒εΓ[YεΓ█W║0∞╟╖¼∞f8>r¢J2▌■æ⌠à╛åßl▐ç²)τ²ûoW²üoGy&|/ès#
J⌠](·

‖ì[∩I≈Qx2xG⌡B ▌╖²&▌₧æ⌠è~O9[π▌□5v┘ö¢+ÿ□╩Å▌┐╞╤2‖±¿o
Ä┬πò╬τhUΘ√▄î5S┼╧π╙x□Äû:h56A¢qpÑksµ*ß
T╢.₧□╡□g─-qx!îÖ1,çX9□
"î╝□sH¼⌠%sêå┌□uGÑ)st¼Dì≤CÑ≤äy╔:□╧b╖fT$}Y╗0v
ú▄□╬«4┐óφHëuDk╩≈b╒┴-.╤¡Ñy□+G
Å%╬╥▒µΦX╣Dé9,v₧Q□Ö,█·╪ΘG2U■ÿⁿèS□ ÜY╓å,ê/_4│Ü3c⌠d¼|%Æ┐
F█▀»□┐╩╓▓5₧D╞lO¼□%□╢≡u»b-%RVZf>ê╦7□[$
ó4a█□y¥>Jrlh┤ ├·&╝öl╟╩□m│□Wn¬tì#Yâ`εÿ6{ⁿ□FÅè¬Σ·[╟τ╒╪⌠▒V⌠;┐9□µ√±╝@-
¡□ó╖φΦt┘L+┘¬
ù ]⌡ßW┤~□ùZ>+□ùY!½1⌠╡ÜZ═`Ç╒┬j□□¡3¼÷p╡uªu□

▒╬╣║:┴⌡╔╡║V□ⁿ┌Ωb¥□7╨I÷púu▒u ■du╖z└═╔Ñ╔
╕àWï&äèU□ÿñRöÅ≈=δæ□f∩├'╣□¼╟┴Γwⁿh┼╥□
□]□□□║:4(tmhHΦ║╨≡╨
í□C#C7çFçn eç╞än□▌□║3tOh□ƒ<O½bGαê≡é│è≥?-&3∩êg─+çGαJ□IΣ╣ □

æTL□τtZG*Φ╖╤J«╥W÷²ï╨)U7n_ö□[Ug·FYµæ?□╞EF~═╤□Ååh╣°░╞háτ╡# D!▌□
i°Y÷.2.A┘# l╦□╪Ä%░□K`Gû└ïX□{░□÷b
∞═□╪ç%░?Kα╒,üâX□□│□^╧□8£%p□KαHû└Q,üúY□│Y□╟░□>└□8à%≡▒ é1÷4>╓╟v²□⌠≤▄
°╦/t /ë□[-à»>═⌡à²Z⌠□ó╛oÄ·┐╡î□╖{^□^□<ê÷╤<(ⁿ□╢╗▓~╫ÅáÑäÅ9béÿînÜÿ!f;≈pëà□6□²□
▒\¼□k┼□─µê═□╛òπ□1▄╣½ou°╢>▒Z&╓□i`ⁿ
î].¡░├⌠□d□┘□a[┘Qvû]┼4y▒∞)√αw║{àv▀Sεar$╥0□C▓▒□┬Φ&û√1n□Γ╙ □
·µòΘ2Gµb∞|╣H□╩Ñ≥N

!≤1╘Æ31mÄ╟A:□"\ÄÑ¡C□τí_Äm<ü⌠□æ□σ□ñ!|─╤□·å▀╖ê²rï<$û`°QyRôZ2╥ï⌠╓ÄZ}─ε╘£▀Ç╨
▀Θ<éß#P⌐{£ü∩n□╛»ÖO□'╕å!ƒy+╚□□:#□±;9ⁿ□å╓□räq■MXΣa╓;(□íSN#╞ù3~7C~□H≥oΘ╝GÜwJ
#.9|+╤µöF√╒1∩à□;àC▓□τ╜□tΓ

╤*à?╧8∩Σ°█è□╬ H7è"⌠r□â!∩QwΦí│c□

`8ôΩ□□╕F°-
▌□.e╕₧ß□╬;æhv[=£¿%■`.µåtn;σ□□Z'å╜{M{åc?Γ= ┤f£«²Üw░┐├%□┬▒□R¼ñ7□╥σK□╦£æ7qÜW
xV□ìp=┐│╘>╬╠¥et7zTk■«□╗á=╢#Ñî7bäU╒[Φ¢£åró╦kà╛-
√╢0-&óƒè#j□┬⌐≡□╟Eƒ□ï1|□~°│,µ┬□XÅπo=□└v╪
{°&åoα0µ<åΘè1|□l□:╞□u▄S'"w»G▀Ñ^·□u║±□a□CÆτ6ts□4pB°t
αôÆÇo╨k├ßm▄p▐ƒΣ▄└╦wªâ=ÜßXN9É╦┘└!|┬à═∩╫p·`+N╙¢!∩L*>╠0□£√C4Φ╞o■$í-
sûq-┌□φ¡s╚║Σ≈S□ε=hCì□°=□├╜≤∞*k0P+&`╗i-
ΩΣ(|□ZªcÖ╫─Θë∞f!°□ƒï▄vB□#╟W║╕├±ë╞±pzΓⁿDµ╝□r□╣N_□Ñ)_

δ╥Γ╧¥7`□▌ F╢1å<□╓à┼+I7□□O{█ï?τ□ε¼uèW╥H┼pè=Å∞ ·Γ!┤°□≤BX»
▐/¼□ï┐çCa▌ëß+┬:»°ñⁿè╥▒¿□

/ å□⌡ç0L□┐)P7≥~@┘╖□4ñ;PΩ}æφ ┬7E*Cq┘wpPCûy□G@░ⁿ¢□æ□ε╬
▀δ[µ╫e╥÷û[&»%5£Do≤4ⁿ▓íìOF▌CùC(┤*┤□ZD▐ΓW«□°8AzëΘ╠¿▄┘æ░|Ü□□±╗Sì8╟▀□╔╩╚═#▄□;
□Σ╨╕l'ÅC╒'  ⌐j□%∩í∞¬
□ûí¼r□²ö╤╛Ä≤□4▄çy║å▓0╧╚╨J╖∞Φ╘╗9⌡░Jª■;°■u£╘Ñ{kOBZJ° ,!5Ñ╙∩M@OYi°<□=e╙□%á°l
·/□≥°¼┤φK╚í▓9╛L╚ú▓9÷'ΣRYë■*!ƒ╨µ8ÉÉSes|¥Ç¬≥úµ¢□Tò╧±m□¬╩τ8ÿ░□-
╓ê□·&ε█Ö▀%∞├≥y

%∞┼≥y■æ░-
¢╕╣d$╧ßä=Y>╧?□÷e·<G□÷fôH«≡[¡ ù░?╥τ9Ü░G╥τ·>□m║╥c'ù#□╟□╨□+╧±□┤┼╨s".mRƒB∩IΦ╗
<àÿ├□ò²ñù
░┐░ Ä≡²°┘è□ƒz□m╥ßS-
┬TÅ╟Oe ╒~çφ▌*}╨ó«D-┤ö±9┌-f'┘y÷h¼)╫>╫₧T.┘□╫╟ $N□□╤d»)▀FjAé▓□*ôèSfT&Eσx\╛F√
c√πΦ▄ iΩWÖzΦ╝╙rƒ≤╥à£Sq□(_kP╛□ò□╠⌐0·∩ü╬è¿á╝Γ╞°LD╧EQƒJ╚Wn▄□τá-
Γ╓□?U/z¬«≡soÖ∩σFm9║¿ç*û╫¼(<>]□íyñ╝Rƒ□□@@[ò¬Ü¬µ¬àj⌐Z⌐╓*M╡S□T║:[¥½2T□╒U]á.T
ù¿,╒C⌡T╜╘e¬╖║\⌡Q}╒□¬┐║R]Ñ□⌐k╒Pu╜□ªå½□╒═*[╤[≥g`╣⌐h├6UMAb·═A├:ZÇÄ⌡┤□□╓╒□Æ░╛
╓□¢ª╥á□╓█

Æ ε

P□δOç:H├┘P□Θ8□RÉû

îφó║@=ñ⌐+┬
╘□Pi╗□□ }ùÇ□i╠éåHg□0É╓₧X2╥ï╣z½▌αC¬/□□)∩□~ñ╛/╞^í«Ç□╢ó?╞^⌐«ä
╢µ*¼qÉ□□□╢ΩZ□a╦åé┬╓]ÅΘç⌐aÉè¡□Ä⌠▀¿näF╪┌¢▒ⁿlò
ì╒□lw2=WiWiWa╤□½╞ÿ°ë┬y^¥í╬└û╖Um1'╡SS□UGl?╡░ûΩñ:a)╘┬┌▄¬Z▄₧:▄₧║¬╗ΩÄφ°V╒SC╘□
l≤uΩ:l3QµeÜ□¬qj□┌≥▓i-═(═2Z⌠G)83æ─¥₧Å]XÑ╘k+¥t□╬□àvÄ╜□sæ ╞.░╫□F≤□├▄≡w■Fi6T▒
⌠àNΘΦ╛Ai╢ò┐╜oorK|⌐LΘò°=£½¿¬9*Uj□║B{S╕t;┐°√H▄╪╙R╟╫╚█\µp.û■□{╜SÅ□æ){Ü²«ï▒╞─
ßT"┌□□9f╒Æ#g≥Ö┌|√¡HX«}}□╧Å╞¬╓G÷°÷ÆHΘ·┼ç┬σ╪*ÆbVΘz∞%ò,╣αG≡ªyòsTßc□QΘñ4Ü╛F╛╗
6h┤-j┐Rm:□x⌠τ█;∞}Φw╪╢┼▀a╚

µ{D▌□G¡`oCì▒├~₧Γ╤v▄gW<WF╫PdÅ╡ƒî╚√¢ÄTù⌠o)K╨æ≈□╒mU□j╛⌐t╥□╚Aë|╨~╟═·VⁿΣUñc⌠Θ,
-
QΘQúrAàcµÆ*╒≡╡=+Jï}é≥9ùσ─▒Å□i¼┌ƒ:8ªx▀▐[UYE▐□Uåτ÷╢¬îª≥ÜáDGE░¢#!_ú?N╖─;Z:aΘ
ú□?yö╦│»╧9èjj÷½Ω╟▐\Ñ╘$
·,â·,3à«╢wdµ²╚°▀ä≤□ª¼\ sÄ╤σ$~P□█Ω"□█▄^┴zá+B£gφ╫□û-gε¿Üà□│ä▄x║
Ü□ÑGÉ═╣RÑ╟ò╚hï╞╡óÿäu|]Ω[□Ωå»├x□ ├±ò□I╒ ╪w█╧┌O─M±c∞Ñp▐╖φ█Γ&H°L□╖t|ε╢█╞MQ□
┌╖┘▒╓□N╙ç⌠q▄O⌡8≤aé°j≡à≤▀P╜ⁿq╦▐f'X√½fΘ□╟MP═╛□V¥▄ëKO─Öj⌡k¢□c╡Ü;┴║o╡>
∞15W8╧╠ok░⌠□÷@√φ□,┐≥+□U \É░÷ΩHd□╓3u¬QB£□÷Ω┤Ü)ÖK┐°µ╨□Γ

═M□rª┌·╝Æ+□?Ω╙┴^
5╫½o'ÿ¢zT½°kφU╒╧ ╙íF5p√Ü,□·╛½µJ╟≥╙Γ╞VK▐Q□╘ñ=│ó&9c□X÷╔ß4ù-
wµ½║₧╔¼╢╥Q"»«N■J╫â╢╢²A□V╟¬^·ª□-
²═Ü)¥fW{ÿï≤∩ε%δ╥°¡Ä□£°hu╫┼N;□J╩«ë□»□╝[▓ÜyÜK▀Qô⌠□»φOjL▌┐¼9yÅ¬Ñ⌠:δêΩ=S&¼¡Ü:
8n┘U|┐ª□f√c¡G₧╢╧jû/?n
╗Jσ╫□τ%Xjéz□@MR□é«-R┐âZΩ□⌡□ñ¿'╘╙`¿?¬╣□T≤╘sÉ¬₧W╤í▒Zí≥í╜Zú6B'⌡«*Ç
╒□ΩcΦ«v¬¥╨[φV╗ßrUñ÷A⌡¡·□«Pç╒aΦ»N⌐Spe¬ûΩü□⌐⌡Rδ┴╒┌┴╘ \ô┌<╡9

JmÖ┌□□°╢Im□╫"MâÉª ╘}Ω>ÉΩ~u?hΩ□ñRG*'üGMVô!I=ê□╫Bèéd5UMà┌Ωa⌡0╘Q╙╘4¿½~ç-
I┴û<□⌡╘╘á╛Üíf@□⌡¿z□╝jªÜ
╒c╪N□█·□°TÄ╧□S=⌐₧□┐zJ=□□⌡4╢?êφ #≥(WσBH=ú₧□Ñµ"GRæ#≤áæz

·╥□·≥<4Qy*□Ü¬□j□4S
╒Bh«^P/@
╡H-éûΩE⌡"┤Rï╒bh¡■ñ■□ijëZ□┐P/⌐ùáìZ¬û┬□Ωe⌡2┤U╦╘2°Ñ·|·3╞.GÄ╖CÄ»@Ä┐¬^à

jÑZ    g¬Uj5û·Üz
:¬|∞Å4∞Å5pûzC╜□Θj¡Z
g½7╒¢╪Cδ╘:8G╜Ñ▐é╬j╜Z□τ¬╖╒█Éí6¿
p₧zG╜□]╘F∞╦≤▒/▀à«j│┌

□¿┐¬-X■{Ω=╙T□n□□▌╘6╡
.R-
¬□ßb╡]mçK╘GΩ#╙Rà¬□%αcöâ-,□┐b9ΦërP□ù¬/╘□╨KφCÖ╕îeó7╥─σ,□}P&tΦ¢ΩA╔Φ╟Æq□KF□ûî
+Y2□░d\àÆ╤╤y¢E╖F╖□%âñDc)╤YJ<,%IjóÜêÆA▓Æ╠▓R[MQSP2HVΩ▓¼ñ░|╘S╙╒töî▀½▀údÉ¼xY>
□▓|□,-
>⌡╕z□%c╢ÜìÆAR╥ÉÑ$û|Éd(⌡¼z╢î|╠W≤Q>HJÜ▓□4π^oÄ╜╕□σâz¿%s|□≤«╡·T}è=║WφE°Ñ·□σ`┐
┌ÅR≥ò·□ÑΣΩ(%G╘□öÆ∩╒≈(╟╘1hƒZ'╡

ƒ[æ┬oÇNσS¿4∩% ▌╦□╝}╝}á┤║≈z∩⌡@g═z=╡=╡1tÿw□□∩p∩ì ╜7yGCÆ7█{+╘± ▌µ╜□R╝┐±■□□x∩≥▐
^∩o╜┐□┐≈>∩□□≡N≥NåÉwèw□ñz°z°A#∩-
╝9╨╘;║╟¢□gx□z▀éNP□Uï□dö°∩óîùε ~b□x┐ª│[SZ┤33╝/╚▌òI{2ç╔æ2█┘□)°╙Θrª╟q├si╫Ñ\╩√
-╫╤N╩¿}öç┬√&Θ╛bMi═┤4¡¥û«ehÖZû╓Kδ°
╘åh├╡Q┌□m¼6^fk□╡⌐Φ-a|û÷ö6W╦╙□╚
╓|âwyΦö□╛╫{□▀£M╖r□δ║^G≈Ω□-
Moñ╖□□⌡6z□qDN∩ñw╤╗Θ=⌠▐z□╖╜╤¡Än;C²□}¿>┬ßâ>Z┐]┐▌i╡>Af¼Os°µå;|`¿╧╨gδs\₧DqFƒ°/
╘ù╕p╣╛Z_□═□}â╛Y▀¬□Ω╗⌠╜.g╨±D▀»-
╘Å╕£aΦä╕≡ä□d□çc₧$Oèç≈\ΓGh|ª┐|êá░□▀┴≡W□≥□πO1¼┼!ô□o└°²□ß□ç|└!ç□ÄµÉ∩□

σ.S,`Φ£Γ2â±}

»τ□▐ƒ%ï□▐╨ß 2╛àß·□≥*Cτ-éz□m¥c/π≡|å¥8ñ5π□□vµû&q°]□Γ╨÷□å├8d'ùv-
┴b·▌P£║à├≈3╜ÜkΣ6jés▌╬ß□□□ü!S%f2▄└Θ_τ2·.□·□C▐i r8û≈╒╪╠╓bç╢╗9÷
«à≤j╧0ö

√rÜ⫤

38ä≈□Iε□±□π╠a╔□□╠am

ç|╨É[í¥b|)CG□╕≈σ□åN ·╧□÷πr6sÜ+8d>π╣

δ□╘;p°?9d□├ ç<╞╨Θ┴ü\╫VÄσ□σm□≥□πiî?┴É╣!/f£9 .d╚╥╚7├ √□N≤kå╟□q'π

□X÷D□├ π\▀p.G║□yε╚▒ïÖ-µëd9æ▌ 8äΘæLÅ£╞)┐gx□ùû┬{~╞±■Hë║ⁿW?ë÷ªv.a▀/µ)ê[J╬A□R$
ï·┬□J4□ió¥H□□"Sdë^«3E?1P

□├┼(1□□ë▒b<ƒÉÿ╧e«□□aúÿ□[─#░□ □Aæÿ┼≈╚□Oí¢╤Ñτë┼X║S÷2,=□ß2▒□kY□╓,:≈Ñµî□û□-
Öß□å<«Σ\å,æ□sY{□"#\╤b8ÿ!δ&φ@╓(udT;┴p^╓¿╚□ûî.ìσ╠≤6π≈2U=□▓∞j\╖~□C▐┼Ñ│F╚X□Φ¼
Ytû□²9

■├≡8├ª

□òH°╞úZ;R"y□δ#¥GæεñƒΣ╨□Θ▀╗a□φ ≡÷■I$êze□K╧╧█╣╥c7√□p*╓8[╟#m□Ä╡-|ΩΦ!─w8c

πv≤╕ s□Ä>

┘ßª_ç≤-û'┌A╛H╟o□□╢Bd┬|æà╣2·╝R,[⌠é□8>√╡gÜb┘░-
\6ª□H-δ0y╧□s□x÷ü≈□▓Vç7□≥,□,│└ú□xε□-7└▓ <7┘┐a|□C╓èα╚Θô

g2ⁿêßπNi╢ì≡]╞
□≥xσ≈ƒ□\╩#`"π<·ü√╥^nƒD╕*¬_;0╖⌐_1─{□÷π(∞┼╗╝w{ƒ±╬⌡>Å²▒╚╦'B`
)Φ╙á□·4HG£ε□═Bƒ-ßC□«à╡□∩╜□╝KC╪ç□╟≡wnφt_τxÿå╕µmφφΩ╜Φ'æ,£ⁿ░g╔
⌠²DO╤G

□â┼01Rdï;┼8q┐ÿ"ªïÖ"GΣè·bæX*Vê|▒

┐S▄F▒El□;8|╖(Γ≡□ΓÉ8*N≥≈ERóOû⌡Ñ)òl&╙d;╬çe╚tÖ!3EN)ƒ%{╔~r °í2ú╦╟0╨□ªO

qi

╙□╬â^

ùúΣ□9VÄù□╣.ⁿ-Iπ╥-°:e╦GΣ,≥Loÿvùnæï┤ùí¢╦{J╬
τv□]XVñτ□±Ösï□¥Ç≡>π∩3╛ö±Ñî┐╓°╝îÅe|,π{□▀├°╝î█î▀┼°σî□╞°gîτ3₧╧°x╞ù╖0àF□ß□)v
□çob|1π#□ƒ─°$╞□0|ìßô□■$π3□ƒ╓°Gî□─°πî?╬╤=°╖.ñÉ□╞
□□0■□A1æC~╦80■□╟╛╙x3ªy╣
)d□╟«□)σ∞□□+?3{ç⌠p

¢□ÄóeΓ╕d╫·
[=C─(╚╝c░□╟ó--Å▄Ö*-□│╪║yΣ █√□x¬╢÷ß╡╧>╟,°3░Oæ1$├>□╤@□è□äñ4ÖIåPå&NÑy-
╨äfMÆf═Rè╩òΣvδj╨Lö║4α[{φ□τv5╛╖≈▄▀∩w¡▀~¼²½╡╫^├│₧i▒-
ñ∩là<x□╘wÉNâZ?ü¥├□a█Wæ╬#IÉΓ╩ΦoG }$▓Mç─É▐Ä╤z,ΓÉ

≤6>î╤□,¡yè□&□A┌Ä@╢

C

┬╪á<╥Çh=Ö!Å≡Hδ┴□□╞hï╓□σcQ■6╩╙╓\9é■□ó·

║Mg□_!ê,)╞$I╧δP╧δP╧!d╪I· ╫╩╒□σa┤åOδτ│$²°Ω╧C°□□Ω
╙û⌠Ö*5ôî««ò╣H□╖åª¡/╔□è&⌠╨⌡UYÇ¥A□ΘëX.é┤5açFA╧ÿJ;□å· □
úφïµ┼φΘ≥É▓m%⌐├G║å┤·□öìZ°)[-
R╢¿u╟X $╨÷lúlë┼■≤Φ√□)□σ?G┘+┐â▓Ñ⌡□≤┐C┐·.Üª╝Äâæ~ú┬╔g□░
┘F∞εl3╢Ém┴ε═║e≈e□`ÅaÅe/■b╧¿╔╝~°··êÉg□C:□åtV∞b█ê□□hD¥%#ÆûBεl∩ cRΦ;¡n╨µå□'m
u'C½{1▄±+í╛B∩╒Të╒╝ ª¡¿⌡]p╫~┤║□#

p□ª□≡━τZ≈*φ□9ë M+┫_τw□╥□f!é┫≈à÷ïΣ

╥æ% ▌æ?P■y█-ú╛ !□±W□□·φ□ïÑ÷-²]ú□ö≈ª nàá.é┤O

Θμ

⌠v▄¥éL┤╞8φ/D>□□φ+┌Nf"Å□╛□ò╥^@Σ□c
δ¥ßÇ·ô╒╞k□wÑ╕□≥□α}□ñδç²KI▀CI8Γ|x□□iƒ'ó□□╤□εà0□□ñ╫ ·Qp$□$|· éê&hO

φ╥iíiqx█┌πêÅ╥>[┌#ì■?-c Üc"NL{Ω¿oìB8
A⌠vⁿ&¬□ë≥/□□î ó□&
┴┘□vDxW□CP√╥□▄ì·î╝ùî├¿öû'H^ßF□₧AÉ« ≥_J·-Jó=i8╜┐ô╤

╥‾?ÜO╜╛ΘUmⁿ G-[┌{FS□▐□A┤πi□□^)EIê?ß╙°Å»æóñ⌡möD»(╤óì□ÿHO`╥┤Ä ▐╬DÜ
□q,&≥ëα╙½é#«├D|ï□î
φδ╗!Es╜╢╜AS□═├≡°RööìPtsEú`╥⌡°J(ë·Γ╫b□±Kë¡╟ε╞6e[▓{░¡╪╓∞Pts∞^l¢ □uQ▀╧(√«δ□å□i /
Ä╬æ$RÆ>7@▐^▄
σæçÜ1□a╨ □Ä┤:@╥;·╠Σx□ └═□□

C°!mtå#╣å¡nπm-┤┐√W┤÷Φⁿ äAƒφ □□Ä<s□ 8φ1ù≥ÆKⁿα4▀ó²αH╙òx▒inτç┌D║□π□z;}

âΣ8Nƒ┬!▀:«╫╣╗p║■s)~÷╤□3a≈√□5■┘□╙ç╨□Ωv·H╧╙éÜ▐αV¥□y□□êÅ2┤QOΦ≤╖3□╥τè┤µ≈≡oΘ
╒□k°¢╥7ìa?\[iÇ^#c⌐yC╗¥µΦ°9□╣□Cƒ\9 ▄Öa"Jcós-&Æ£L⌠%x&Γâ╞«m▄ç>uÇ-
R□E*·÷□τ□=^─6G┌}ƒ?Θ≈
«A¥CV -
╫ îMî╙║□:MÆ£║"zÆ╚ó▐möM█ú8:π╞æÅZr╞JK□□)«ütbZ»┼}□D°½8¡╗á│N·£¥>╦ªy□â╓╙╨ë'Ä▐ê
#⌐êOÆΓ&t¢⌠Y□-
┴□I╧δP╧δP╧δZwHoäÖOÅé≥÷0╥□-¥tI<<┘T□µrä°9&¡)□Æ▐╓╡┌□■R6±GO╤□τ▄⌠□∞╘½Ö·+□□«□Xα
ls□¥Z<a:■ò⌠-u¿t⌡J□ M(}╤:|║f tƒ?ª┐{²7┤ô>⌐
yD╟Ä╤:B-├ αä□□'î□□Σ╣|ε□└τ┌s=Ç□╫ôδ <x□╝20éW╬{□□°«|70à∩┴□Γ°Aⁿ 0ô-
┬□□│°Iⁿ, &Ä□E`╖ WÉ
N□□□ù└)┴-┴-@┼è╘□ö¡╪□°SæZÇ⌠╧;⌠ÑF%N□'ÿ□╩Ö╟ëΓLßLà∩□

[₧□█f□□¬

└¿Z□R=Ç\Åá"°A□□P□Q┼□□`?╠δÇ┐ττ□∞√;░□J@cL□╙A╜~□▐A·≈

û,F≈0a╞s¡Θ□éTä□:Ä7□²╓çc░óπ┼â±p■□□ô□╚ □,N<g□□±R╬r
╥╢┬╣□gªÆs□(S▀é□r╘ùáü*w$╫
□¿°║á□w6w

PúΓδ□é╗₧╗□tªó╥□uεnε-└áóü□£[──é+{â{□(pδ╣⌡0▀╚m□□<Y₧,Φ└δ╠δ

Tx ▌<o╨æτ╦≤□□^,/□py╙x╙□Å7Å7□≡yT

9Y▐n▐ná┴╦µeCÿ║╦□è╝╚╝╙□□≡.Ç.╝'╝'@□┼ƒ╫Σ╜σ╜à⌡¢x=@û▀ô▀□«Æ□□□╨B4├Γç┬□c±'≤'□-┐£
_

δ<τ?ç░ü▀□dUòTò ∞Ñ┌□B□U□□G⌐Äép¼Ω8□7¿nÇ0G5□┬ô¬'!,U-à░E╡□¿¿□¿□□╢ÜïÜ
äδ╒╓□ûZ║Z:ÉU█í╢□┬3jg ,T+ä≡₧□£U"Å╚□ZD>æ□:□□D□ ê+ÉÄ┤ë□ó□ûû□Ñ□_FöA|9Q

╘ë□ó□α─-Γ□¼s¢╕
Tê;─□└&ε□w!ªè¿□<Γ□±□h□5D
╨□8
£üî└Uα□□□7ü□P□x□<Ç╝└Sα íù└
t□î□î□□□π□π□_0A0□h□é□A≡⌐□A□¼□*□à0L□□kN□Lå5ú□╤░µ□┴□á·s¿□#@H╔4╡{└d

□□/Ü┌OQt■ÖD╒2F; · _j ù┌ ~j?è¿╜□sàÉ =4▌ä7□5ù5°5φl┌┘|□Çû²□W╘∞╪b┌Æ┌2□ª
-√[¼╒ =·É▐tí9▓┘▓┘-╓tâ°í-□ÜéÜ¬Ü/╖XP_Üâ⌡å6ùQ □≤▒═µHX·ù6Aƒû□I¢

□⌐>□J ┏ `

ö╗£I£HH√Q£□á└Ö□Ñ«2ÆdlH/ô□□«Σ&@P□<┌)

ㅋ S□□

fñd`□|-ç╧Oé·0N8╟Gp"P\╪HÿÅB▓1□╢ï├víäΣ─┬╓që£

ób°Bi ƒE∩ávcg╛ ▀□û ·"ê]Ñ╢□(□╡D-
d□/ëù@û¿'Ωü□±åx□□ê□ó□╚□oë╖ÇA╝' ▌├:MD□`□═D3\?L└□ÿÇ%`□Eü¼@

0□□□□Ç
□□è@Aá,P□\üè@□p□\□□(
°□>─¿ ╘□[@□□╕╥¥□Ω□ú)╜□<ü╢@□¿□⌠□zá□∞Ñ+Z⌡ph╝2ü▄_╫□╬r5·Bσ}· █Z&┤╠ç⌐é·┐K⌠
2T

□╙σ□PªV□╬(£M8ùp-í╢□─□à│B¡ò┬╥╟;|é₧╙
ÿ°v8å₧ªµT□═#å╞îú13□l□□╚óæπhΣ8□9□ì£àFÄúæ╤áæ3╨╚□é«é«@÷□╞o□G□âh□²∩T≤fèB╤╕□E├
≥ò-z¿f╟ Σ°iφQÿ~¬=b┴□èWï╛δÄK⌠?□-□÷s"∞'▌C∞Km└y
K□

(□ⁿ□╞
Γí¿│p╓Ö(╛□â¿àúé{□▐Sÿ!0Qx&╤Lφ□□╡oìa
ç·*ú$▌□┘0· )▌D□□□╕├▐└¥⌡□ε(║□□▄7lAG□╡O°p~4⌐Öh}_ñCK┐□C▀╜Ñ"½╚□▓ä.íGt%⌠
□□Åfτ»x¼%-╨□□□□@□!Ä□m□ÅÑC╝Ω□)ó-
□I╥ûÿE¼□#P□ò□□E%□EòñófQqcçá°>ⁿoì╦$hó╟▌l√#OíHC▀ öµ□=╡Σ□₧┬~Φ)□èà⌠²s╕·┐≈Tï≈w
?□⌐úe è□⌡▐╧üΦûC╘s▀□┼
┼□ú₧[ï"I}▀s╘£D■╨Φ

²└Φ~ö▓□ÉÅ²╚j□ ╚~lƒ╤│┐ƒz-E┌□┼□ì"l╣□├%mR╤╝ÿ░=○┐2nÆ□╘D|Ç╢DΦ■╥QÑ⌠%-
·ú₧┤▐cπÜΩ tƒ╒t╢⌡₧┌}□ÑKí┤¥-╒:à±■□□5C{⌐□□┤

ßt&╘□□O

□□□□┘∩╙╪┘╢╨&Z┴┘┬┘

φí╖£{£ù£W£z╨□Γ□r├╕ß■╔ö
─]╞]

m␎<ε n9≈&≈□≈.≈−╖è√û█

¡ÖY╨r╔Ñl□~□╛□ □ ┤=₧≤□á╜A¿Ü@{├å▓0TCU├Uù¬.âvF

┤/JU□W¡'╞□╩□₧┌εe
µⁿâë≥ΣRq╠Γÿ╟ ¥┐ƒ?╠ÿ┼√w██□ ·+D≤Gqä≤φ╠□à▀╨

₧Γ□□("a{%□>[é╡[B»₧>Φ╫N□╥Çí▌K═úN╗□

φ*₧$╓╫ZΩ∩╞X╖
┤°÷□úè5░╡}□╨°¥÷1¿_Smwi≈óz²▒]{ûBk[z╙╢¥╢á¥!Θ⌐V╗□Cr╢%▌╧*╓Γ»╬@$'è-
ⁿc3Ç+I═└□╥>ò$φ3≈#₧EΘ╪-φ»3µ╤R±ÖÆX□æ▓┘Æbño╡S┬≡ja□□┌@δ¥8GP□GDì╔╛▌ïà╓éÇkL┘$z□
vA╤¬□Σ▒αPq0`□%⌐⌡ ≥J²£╖¬│·HΣm{╘Kß╫~□□░}<╥<┌½?»}<□╫▓▌·ßφ«╔
H□□8u6≥ {4u□┤\1□«□┤■µ├╡_D¼□║╚Γ3ª"δ0NH«s≡*öⁿ■╗«½Æ▀eR╕[R·╗□π╗-┴½ZΩ■ò╘∩╞O«Åc
-ö╧ îqK_╥πn■□╟²ì□╬ⁿü╛┤═#ëóτ-ûD╬ÑΓµ□óê╣□ε□èVⁿ□4B╪î11□îì⌐┬ñÄΘ└□u□bªÿ□╓
δç
ä╓□▌□ó4□s├╛░q□□yⁿoìHÄbs┐dΦ"°£èÅî]ç≡*¥G≡*#□σY(□îè°╠╤BAI═+□Zí╓«ú╥y□├ó0¿σ½□≤
ÿé□□éα□□□□_â₧=ìb□gRzÅ

èòî╖á:ò□6!XÅ`□Γf╘¿c0J□°9±┤i╧╒1□□åénP¬P□□≤ß╧□·°┬ç∩┐09@Eá■íg?█-□^¥╨e□ªó┐╤□â
·≡≈Rx¡□δ@□╪

÷Ç□α(╜Nü≤α2(□σÉ□εâ'P2<□»┴;¿r╔`JΦäÖ□e╥;¼
ªç□a$óù»╟╙Vi╤ÇP╨<é╧#¿ï OÆƒçΩ╕á:+P¥□□Å≥-
úPM:┐□σ▀á:oP²7□s□a((hí|î□╝□┤d!□K┴µc(/Å≥C□∞Å ⌡ö¿
²¥Ns>¬□å≥┼(O╥·L│m□□HY▀═║ªK▌││▐ª4¡□ƒïµ
╡«µ+ƒ-àO┐╖ⁿ├O□╡φ≈M_h[
╚╝╫o║⌡à□╘╪¬Üû□&Ä%*oHⁿJ·ü/öS²/ⁿ)Ñ╘╪╒┐▄│╞ú_)□÷σ≥□-_)W°╧╨¢
┐R₧≡û□2"┐╪┐ _)_·à≥ƒ╖▓ ïÑ?ù□⌐

%Ω7╨H□¡æ╙4RH▒╞*□yúd╟Σ□eLûæ!╓ÿ□Që

□*Æ≥2¼ε□pF'□ }d□║╤@□Dl┼└ÿ□εΣp╥X□ú╛╡Kó::□Ω□\Ç/ê□□`□□□1≡▓Ñ□⌐-
╒□ô□╫╝8jl╨1∩¡>□sK╟ìy·B▒k^åÿ□AèÖ·ñ□▀¥ü30□âK²çΘQó╧╬¢°┤□b╘ßúñrko1□∞W,Ω&>é)├e
îp□r╔Ä╘ì□WañOtpHxPLD╕ÉMváÉ▓\Y╖□ ░êp□a□R¥┬(p·├Bⁿó"ó#□c┤∞"ó"#ó|bBα□┌ñ&UÄsë╢
rÅÉ░□□≈□ƒ░H-W╗■d□5ea□▓'i%┤▓┤╢░□
o¡Ññ╔ñ▄ƒ╥3eRæ*WΣ2ç╣╕║
╙«⌠mùp╗É╙αÇ(-{w□-□wτ^V÷"□□s+kæ╔□ráò░+⌐KÅH╜▌□╡□DM
±
┼ÿÄ⌠

c,Çï1□□±□

1åüì¢/ö╕\╧╗║□
s╪□╣mç□n°φ(M;□P¢?{╘èn□▌L▌;~╨·□┬≤

A╒''□i╘╤╒[kHê«ïd╟╟═╗q╘╥▐y}-_ôiR88δ°ÉΓQòⁿ╔²ï╔□O╓á╘.gV╝^■|│±CíQ¼²+╡¢ò¥ÜvfÄ╝
5¬fσ=~ΘÄc>²δªe╠∞r╦*√xv╜ú∞0böy/╬─⌡¥╫┌«▒┘□yiτûù²{eƒ╛£╛qs□δu≈iEÜ█2╜⌡₧ì

Tè╣□─0¢╜≡□▒"╪─φ╠█≈6â╣O╖σù{ûT(Å-□6<┴╙n╠ú√3G{8-+⌡-
░°≥@□τäyå╪╝£∞í▄ù7▌□¢^{╨Γ`░X▄R□hvuτ╖3□8▄G[─ÿ<£□□⌐□°Tú□Sò╔[q:aε▌AδV\y╞Ö`c░D
qí╨YîhHCùIÉ¬ë<]ï╞_▐□F*╘⌠{?⌡}n≈²·û╣*ñ□UAô9î□B:e

╧pH╢
Äëë∞efµ□5╓4∞π:Ö·EäÖEååPX│╙¿□ )~1╤f¡╥H¡"ZDHòª░□Θ%#□7&ï%ïa╞íΣ`╥±π=╓Hε#yAlll
{/□ê·B╦1$ùΩoWª□⌐≡▒I\εô
ëSTóδùiÜóùy)α╕■Ω╧R_░zgî╫▓ë÷╖□7▌□z╨,-
°╓□r█□5ú7ïó¬"∩╞]Φ≈x▀πRòyüå·┼Å┬uë8▀▐╙ev4¼□¥▄|εåhz╪»~Ei□ç╛□≈╫

ì├÷Ñ█M°¡G╪v¼┌¡╦k
½Ö=□z=cNÿ°╦ôƒ«τ╨Æ▓\y╘·CΘeK‖φ□S╠Ü╢δ»ƒs├⌠H╫╕éSâÄ▒╬.ÿ:*uzà╔═□╗½║j,□r≤□╙èΘ
*□□?hef□¿$╙OåY∩≡?1Σ┘Ç≈U┐╜«¢₧┤╢[╨vP▄{åa▌╡□1□oδ⌠┼r{□ôΘ╓ô√╪÷∩rV¼σ}═╖j╒
□╙Ö┼□£

╡ó
C⌠f)Ñ╚□Ü&æb□

▓▒°Rl∞ⁿ╙□ì
I«O[□□;/=kèÉì=ⁿ)╟┬É╘°7╜ªt╣□Çû{HP8lòbdZ"R(D▄╟è┤□□E$L□47k╗%c~J $σ°g╧┐
╨ìµ/<¬ù/╗l]b< â■ä□Q≤ì▀╝▐Æ2□φ└#[è╞/0δen┌eE▄╗ΘYÜb∞PBQ°□°σü╧
╥□▐35^═Uh╤
╧|□dS`@<4╘|├\▌▀»·┴q■Γ□ε:╥▀¡#="zW∩uÉ'¥╬₧ZFª)□M╜╘□╜F5÷ùEy½/╩═╒¬Θ▓╙≥σΣ
swc└ÉàÑ╗W</Åk^≥n∩ä·6'Åiε≤M9]0'g·╛≥∞ε╫=▐[▐║2yσú.-╒ôCïf╔Mì╢╤-εX÷□□:

▌"k·pör╙⌠
àÅF?ÿ√ª|¥èµ╥φUs╘╬û_N╫└.69εα«4O╤v□5₧╙█

£r┐<;╝¢wR¡uxb}^5W±·Gnö□gd:═n║R∞ªU2□ò├Zw*.┼«è╨}τ\¢╨≤YK╨╣1Ñày╗ÅΣsSI7¬╕#□≥ó¡
âHçO%ì□)ónY▄ε"sÆ□è║√Yô□╛û□>&□=}-L,Dµ╔&╔µ=D&■╔û┬@-
æ╙╥"╨∩O,╨1▄ í+δ║xùÜòò╬í░¥ù°0╔|₧□╢╦í""ú□□äΣ□Θ□R1$`è~╟S└ä┤2!¡□
⌠æbü#H¿¡H▒@ç»╛α#□ⁿ┬+bH%¬π\

ka2H≡╔v╞┼

╙¿j■6≥£kí«╥µßq7k□¢«£╝qµσ█╬₧5εà!âX7╬□U▀ Éµ╝f|Gk├3,□ε▌u±≤O□ε■-
∩9cäε□□▌╕■a√□_é╤½╙□¬□╥»)Y°nOfmS╝x|É≈¢ε□ï╥ùyYσ;½gδ\f_⌐□││,δ÷Θ□.╙█₧┤¿╥@╝*Pc
ü¡i╥H|╪┘≡┘□óτ□s═\=╟╨Σ≡□□j°□ëVzP₧á»b┤╓açh╢φZ█æN▒║
Üs╪□□>öπ□/Φ>ZΦ▌sΓ┌¥[τç«5îxy~▀│ô

j┼╛╬Iç<:
ZÜ║─
∞L╕┴àF□═┬□¡,┼£ùW□╫¡╛?qc╙∞╟─7├┤Üτ▐h╔?Ü╥C╛┘åw6òùu&╣╕V|v≈□=;ΓÉπ▄╕ΣÆ╖Ñ□√□~σ-
x╝$=Xo~p∩¼ïë╬·Åσ┤ç·5mX┼─
f~╚sé╦═┴╟¼ù╢ÿ▐╔□┐╒.⌠R▄╡£╝╨e│'═ï┌⌡l█√⌠;¥╩{~≡┐□f+≈p·∞£╜'╢□ƒvm¡τ╓□»"╬
▀Rφ┌□}╬
□□╠l²╖YELpφ{─~╣KåΓóS3╜■╕□4╧τ╖M⌐τ
□□E

║w╞tuM╬√╔░ΩëN;ƒ«¥ZxRε|s∩7√ó¡d

x^□öσ╜Y}y₧·½─ëÿ╥ß╬I╤╢╫╜u·÷≥"*τ┐□:∩┤├∞v╫E6πJ¬-∞Wh£Xí4Ul[{╛┬$ô╔XΩ°╢÷

π□╛□□□Y(□ji!áαú□lüx┐·°*∞x─N□ΣWΩ/X⌡╨╪□¿ΓÉ□à□RφOH·Vbàd╪¥µ¢zm|╙─
"□2OH║!ü!~>1□Z²°─□GDà──S╟¥┤"-Hsí╚╥£∞
Ö╗Hên═IΩ÷ƒ╙í┐╞▀╙3'σT■µ╕╥hz¿⌐α▐╓√□□╥åδ║ε╜zçp╓Sy±╦Ä_åεì!╡:>ù╜ß▒å∩┤║≤Ç
ò√R╟É·╖@Φôi'½□╚¬4t`ª╓─(╓,2╫¢╖±╒δ
uπ□╙─╧╫x÷╪yKµY]≈╦K▐9\ô/□ù]▓□□s≤█φôV□▌4╝=╨}□r╔C├üª□{Æ]F╕)Uß╞∩'._Nå╧½─
En|7│<%≈ëv╩╠╞Rn╜▄□≈0╖â

╥╙□┴αAü□
║□εL⌐║.ô4x≤█9;:

Γ╓ï╙τ╘îêk╞╓i╕╩═□lr`═æ▀u□µ¥7±H╧ε□╫_□[╝╛▓≈∞UÖ>îC□╩9□╓└«Ω

±hy╤╩?°Ñ°æ┐∩å3▓âTiσ8,□ç┐ñ°y╗┌%┼╛5TÿLH□╔$[F^"□°□à□dR*=¢ôûôIK□y□÷ê'⌠≤4Hy╪ò√
┴Φ₧é√ÜQU[3²╢·ⁿt≥□│π≈¬f

╬╪╢wh┤╫kY«i□ΘJ
□'□╧í

╗î■╔}┐]/n-ÄéoñX9□□-R□┴æ□H┌K  □δ∩╤ë⌐q╪╤¡~ú>

τÜ¥▓0□

n▀π╬╙â{c□╗□?|□ûc□3┘;Lë╗√Ω⌐i╦ÄÜûq6/

≤=:ÆQΣ¼┼uM╗ô╨∩■╚╝l»uΩ≈4░Σ=yq»□òT≈╞^▄?╡LüU╕─±~¥; Ä╥εòUÅùL╝æx÷╤ΩW2fs±°+î⌠t"
■ ±í*.═T╣A÷~Σ
┬yπ╥Pà¿5G3{n□2)□▐ßÖ∩ÿ╛¬⌐ï┤·▐ùφ$z[,□<Uh╙═J▒≡YñM╦\□nσ9□ƒÑu7Å¬=w^4½└▓√
╕-°ƒƒÿí8`ZÖ{ö÷
≥r^\└□oLMü╫í⌠□/⌡Mƒcü^╣&fÅ▀╬M.-ε·dcΣΩI{z

-√#■⌠."┴╖[φµ⌡▌,db;·^▓Θ□ª)«S╝h£w═.≈ß█Ω□ç-l▌□cy╘╡`▓.G□¬b-
╗┼ôG□┤π¥╙═▌?,¿0}@Kb╝vΓ&>□°d□g\°┬M:┌%vO╗?={φXl\V!J□¬oΣ¿7~⌠3╧┌φ┐°m╝▄+ΓdÆAîL
╟□S╡O»□ƒ5≡8£3╤fAµTƒâßÖ▄φ°w
¬πD4-
□M:╨\9╝p▒εÑ└ô□5µqⁿ□6&┘úû□¡╥~xh e┐âq-¼▓■ª«{V∩▀□╖;7cφöN┐«£╟¥óc&┌)□₧ß╝╕δΘî┌9
ù╡╦ƒwq╢┤εà╙▌□, büΓî┬É┬Gß╧vñ\□vkΘPα=ªbXτ╠èwf¢·ÜÄP
╜━▌╥Dèe□H1╦≈ú(Φ░╝□ë□ⁿS3 i■Oa┼"Æñ7d╖o┘Ém□ü□è
k□i┘ô□□=╨¡Éñn qïE╠°½∞`P▓â□e□▄s╗δ▐E▒╒M≈Vä∩□│çY□□u╪K;}@gú╨°ú]w□ò▒ε─t:>+_⌐╦□
½╨
£□┼:δsi2√
{▐└x┬□╫□(╟√╧¢╣zé▐ñ∞MN□₧□Å+¡\∩~@┴8?√╫¼ε√□Σ│o«□uyB'╓╙└⌐ODn·□║╠╗σ\»σ┌-
□[q▐□ƒ▓;╕╛(¼╛╪ÿL╒╫□Å▀╡÷▀□εo□╖=├O┼Σz┐Uì□~ùU╛1&~¢S╖╟╨°2┐▒°V█╚╛□╨}4[sÿ°ßµä¿╗
£^G£╞U╘╧¡ÿ²δ┤□╙Æ; j¢│x∞ô□.s:╜╨4□U╡╝╖╓>s»é#╢═óδ╣┐M╬ü∞ò╓3K7&□┐q÷\ím┘5┐g╕ ,
≈π□T÷□tτ□╜>Ä'/i□_WΓvz±Ωy'╬h╟t□O□-.60┤εÜ┌spÅk╙sVεS╫▌æ□Xφú9±₧í╙╞±≤∩w□{]{ê¡█
·C#√Ωßu┐$x¢▌╨}□9Ve°└╪▄Fp∩─-åxⁿog°╣';ùì□≥┐gª╧S]°□─Q√Θ

Ug≤ú□εF=╓½<=0¡á÷£·╚▀f/⌐-µDε╪╜┤▓┌;=√├¥²ü≈╧ª$M½)»□≥╪⌐█

«ß÷□3é□-⌠ì□□└l╬═æ□╞£Ä54|Y□ûo╕╟xY?+ù⌡≈µ┌/8/?┤ál¢¥Y╟Üå≡╞8-/cε╪
k╓┘║ÿ╧╢╡□╛┌∩¢£_»▌□b`╞ñ╘╥╖σ≤□╖╩╬□(;ƒ╢#■┌äg╖vëá⌡□-â⌐╘E□╕â)└□╪ü■□û½□□╨
╥□OöI/åp╣▌1-╤·▐│□□à┐Φ.░ G╙┬ìrí║d

╥□Æ∞⌠]N╕oß«à¢╡╒(□OÜÅ□ëÉÿ□'%µ▄HW╥YJ╠
°61≈à÷c╚ñt¬≤Z╠ñ□2i5Ö┤óuÆLq2i6┘≈πδ□ÿ¬·╫╠, □┐h8▓É0ƒ¿x┐╚h╙αÿ0▓_k□

╥óïHK

□□ □°ÇI`<êD^σ□α□Γß]4êüx╩├□□»pÿ3╒╥h╧□
zò╝-⌡«G|'╙δ□1A:δ□╫v╜τ╖2m└┌□Ñ±J╦═□î75╣m╠Å·%lv≤⌐╛O□.≈>=(kK}╙o~°u,╖Ñî
ÿ⌡|╞óü«#*öVN/φ4D╜╛╧ÇEn%√¢B-
╪╨Üv[ ╙ª≤╢▓C□▒½{▐□Ω□╔▐&.A╖₧;c√≥ÿ┘K^□Θ3□□¥[╚╬█Ü┼RZ_□ⁿ.╪tMåQ_úP/'?M·É≡╤⌐k½f
┐>|¼~`≈▀?⌠.9iY□▐u▀├lâÜÆ;⌡□▓╙

SRçu░Q|%╖á\3_D▄»+0╣Ω╜ΘáSOà
□τ.∞▌≈≡└»┐±τ□w≡▓□M6Φ4+τ╡Aπ∩╞╝┤BR□îZ□□-▒πHL~?û╟v╟╚╨V▄ù;,P±Lε░7≈û═RÅα╧p╪1⌡a
?úÇ-·c▌|ô≤5ⁿzñ$W▐¬o|ÑÜ╣╬α▐òm)%/╞·⌡□α-╗a₧¡L¼╟/29S4y°||

╒▌╛╨Öy¬▓ ┼

å/~□0½N·#s╟┌□P₧9≡Σ¿ ·öm≥C□┘iëÜ%á[A╬ ·m}□b╗X^(▌╝9=!Aτ¥π□═▌∩□Θ&╛┘╘x:⌠╚Éö√╧°─u
¬~fò□O

i)╧╒
₧≥(√▌ çE╧□□ƒà⌠╬■@╓0ç.¡¼£□µ╥┬µùì₧╬.°□GΩd╞u□i'╘÷W╙Θ√~g±╓▒g3τ»-
9┘╚╧╤ß╟ÇKδ°z+$:å6┼°ƒ=□□6±Æ[4W9┴⌡èP╟╙▄OèÖ{□□F&¡·°□W√ε└╢├æîñ≤□≤æ□▒<.TÆ>yü╜h╗
S□v ÑK·ñn█âL!dm⌐┼ü!⌐í

∩□⌠╙_∞┃Y8━₧q₧K ·K=ó$⌠$=2î□
┴0┤▒¯óα□ú

o□ßv╥□-púQ¢.□Γ}`.□─gΩ'Ω}vg{─GF□E·D□╟k}"¢Öb

¿n╦IÅ┴f┼g<╕ □δ¢>c□╗Ö╖⌠b^╖┬╙[≈\|l^□T¥'R2╩?╢≈▓£ò‖ε9█Ñ½Å¥1>:╓q≈µÄâ□¼□╢Öà.ΩT□Ω
@-
⌡]ⁿ□╢┬%eYBÖ≤µ╦□·─M╙ò╙╩Üc!─│tF-M.8┤╟o▐╩τ▐wn═╡»W⌠X│"╓╪ⁿ╪u╫╛Ö├WëδOu┐}Jt█Ñ6Ω≡
Ñ┌±□╤▐╞π▓¬□╥^╒¬÷□┌_sJ\┤σ□½FL╗≡$╘┴╧Cl∫l°╨^ufPtsΩ▐╒□□-
√Oz;│[╟□²╤Q}c╢dU]□ë∩Åδ1╫Ω═╩FƒY{r╢Ptsz□□{π°êé╞e■√"□-VΦ<ƒ╠┤╕~░w╖╬UiuE□□▓BÜ2┼

C¿₧Φ╡ ¡æîP╟αCTGDÜK 1C╝²ô6)Ü□K□╥$⌐╪vbê┴ù╖û░ä*╚q▄Ch)□R?ú Bæ6#┤ª9»VNφ¿?¡ª▀εÖ!
÷╖¡YƒÿL□¡≡K²/□8Ægæ║igli╨er∞▌ü▒3K«╬╚;1}gßIRäà═r {╛dW¼▓hgPiyD╝░ƒ_Q╘
úÑYù╡
¼5ùds┬W⌠¢≤hΣ□;r╒╚╗G▌ù╜q:dⁿG±α╖Csî╫
□□? ╬á□[/□~⌡║`ü~ÑP1▄│°╜Ω┘ÿ╟ì

√{╕⌡½┌■.╝╙π¿k#G2.ò╕□₧{╣▓∞ù⌠┬R╝&≈ïV,╛ò£∞╟╧^╠┘8;┴═#┐≤┌√}-
╞G6█¢┐_┐╢ûi_R┤q╠m┐<Ñ□╤□▌╒ç⌠ï≥«-«=└|╙-ΓdXfà╠
├ge∩ïmO□╧e□╒▌7J≥rJ_ └p¥ô┼╣╢bσ≡Ö╒ΣαeYîå □6é □▓N¡+
□endstream
□endobj
□36 0 obj
□<</Type/XRef/Size 36/W[ 1 4 2] /Root 1 0 R/Info 16 0 R/ID[<DA5FCD1914B0FB43BD8A676E3A2F64ED><DA5FCD1914B0FB43BD8A676E3A2F64ED>] /Filter/FlateDecode/Length 133>>
□stream
□x£5══
éP□□αy ≥°Q!-⌠f□÷b□ä@□─□<x│
□pá bb□□ï└≈ft□√eô¥

```
αf₧ì█%αΘ─@╟â╪I«$=ï^╝Iv#·à¼qY∩╓╞ük╪┴èDñ"┐ù╟> ╥ê@äb!"▒tü≥╬óφ╓V#⌐ƒñ╓
I{╛¥═r
endstream
endobj
xref
0 37
0000000017 65535 f
0000000017 00000 n
0000000125 00000 n
0000000181 00000 n
0000000464 00000 n
0000002280 00000 n
0000002448 00000 n
0000002687 00000 n
0000002740 00000 n
0000002793 00000 n
0000002967 00000 n
0000003212 00000 n
0000003344 00000 n
0000003374 00000 n
0000003534 00000 n
0000003608 00000 n
0000003848 00000 n
0000000018 65535 f
0000000019 65535 f
0000000020 65535 f
0000000021 65535 f
0000000022 65535 f
0000000023 65535 f
0000000024 65535 f
0000000025 65535 f
0000000026 65535 f
0000000027 65535 f
0000000028 65535 f
0000000029 65535 f
0000000000 65535 f
0000004528 00000 n
0000004945 00000 n
0000221369 00000 n
0000221907 00000 n
0000222220 00000 n
0000222495 00000 n
0000419521 00000 n
trailer
<</Size 37/Root 1 0 R/Info 16 0
R/ID[<DA5FCD1914B0FB43BD8A676E3A2F64ED><DA5FCD1914B0FB43BD8A676E3A2F64ED>
] >>
startxref
419855
%%EOF
xref
0 0
trailer
```

<</Size 37/Root 1 0 R/Info 16 0
R/ID[<DA5FCD1914B0FB43BD8A676E3A2F64ED><DA5FCD1914B0FB43BD8A676E3A2F64ED>
] /Prev 419855/XRefStm 419521>>
startxref
420753
%%EOF
